# MiRNA-30d and miR-770-5p as potential clinical risk predictors of Vasoplegic Syndrome in Patients undergoing on-pump coronary artery bypass grafting

**DOI:** 10.1038/s41598-023-28978-2

**Published:** 2023-02-06

**Authors:** Omar Asdrúbal Vilca Mejia, Renato Cesar de Souza, Aritania S. Santos, Bianca Meneghini, Ana Carolina Carvalho Silva, Guilherme Visconde Brasil, Vagner Oliveira Carvalho Rigaud, Luís Roberto Palma Dallan, Luiz Felipe Pinho Moreira, Luiz Augusto Ferreira Lisboa, Luís Alberto Oliveira Dallan, Jorge Kalil, Edecio Cunha-Neto, Ludmila Rodrigues Pinto Ferreira, Fábio Biscegli Jatene

**Affiliations:** 1 Departamento de Cardiopneumologia, Instituto do Coração - InCor, Hospital das Clinicas HCFMUSP, Faculdade de Medicina, Universidade de Sao Paulo, São Paulo, SP 05403-900 Brazil; 2 Department of Cardiovascular Surgery, Hospital Samaritano Paulista, São Paulo, SP 01333-030 Brazil; 3 Instituto Estadual de Cardiologia Aloysio de Castro, Rio de Janeiro, RJ 22261-03 Brazil; 4 Marinha Do Brasil, Hospital Naval Marcílio Dias, Rio de Janeiro, RJ 20725-090 Brazil; 5 Laboratório de Carboidratos E Radioimunoensaios (LIM-18), Faculdade de Medicina FMUSP, Universidade de São Paulo, São Paulo, SP 01246-100 Brazil; 6 RNA Systems Biology Laboratory, Department of Morphology, Biological Sciences Institute, Federal University of Minas Gerais (UFMG), Av. Antônio Carlos, 6627 - Campus Pampulha. Bloco J4, Sala 158, Belo Horizonte, MG 31270-910 Brazil; 7 Laboratory of Immunology, Heart Institute, InCor, School of Medicine, University of São Paulo, São Paulo, SP 05403-900 Brazil; 8 Institute for Investigation in Immunology (Iii) INCT, São Paulo, Brazil; 9 National Institute of Science and Technology for Vaccines (INCTV), Belo Horizonte, MG Brazil; 10 Laboratório Cirúrgico de Pesquisa Cardiovascular (LIM-11), Faculdade de Medicina, Instituto Do Coração InCor, Hospital das Clínicas HCFMUSP, Universidade de São Paulo, São Paulo, 05403-900 Brazil; 11 Present Address: Center for Metabolic Disease Research, Temple University, Philadelphia, PA 19122 USA; 12 Departamento de Cirurgia, Faculdade de Medicina, Universidade Federal de Goiás (UFG), Goiânia, GO Brazil

**Keywords:** Molecular biology, Transcriptomics, Cellular signalling networks, RNA, Circulation, Cardiovascular diseases, Cardiomyopathies, Hypertension, Vascular diseases

## Abstract

The aims of this study were to perform pre-surgery miRNA profiling of patients who develop Vasoplegic syndrome (VS) after coronary artery bypass grafting (CABG) and identify those miRNAs that could be used as VS prognostic tools and biomarkers. The levels of 754 microRNAs (miRNAs) were measured in whole blood samples from a cohort of patients collected right before the coronary artery bypass grafting (CABG) surgery. We compared the miRNA levels of those who developed VS (VASO group) with those who did not (NONVASO group) after surgery. Six miRNAs (hsa-miR-548c-3p, -199b-5p, -383-5p -571 -183-3p, -30d-5p) were increased and two (hsa-1236-3p, and hsa-miR770-5p) were decreased in blood of VASO compared to NONVASO groups. Receiver Operating Characteristic (ROC) curve analysis revealed that a combination of the miRNAs, hsa-miR-30d-5p and hsa-miR-770-5p can be used as VS predictors (AUC = 0.9615, *p* < 0.0001). The computational and functional analyses were performed to gain insights into the potential role of these dysregulated miRNAs in VS and have identified the “Apelin Liver Signaling Pathway” as the canonical pathway containing the most target genes regulated by these miRNAs. The expression of the combined miRNAs hsa-miR-30d and hsa-miR-770-5p allowed the ability to distinguish between patients who could and could not develop VS, representing a potential predictive biomarker of VS.

## Introduction

Coronary artery bypass grafting (CABG) remains the most frequently performed cardiac surgical procedure worldwide, and mortality rates have been decreasing over the years^1^. The utility of employing score risks for patients undergoing cardiac surgery is also evolving, and several risk-scoring systems have developed during the last decades, like STS Risk Score and EUROSCORE II^2^. They are able to predict a patient's chance of having postoperative complications in cardiac surgery, such as mortality, renal failure, permanent stroke, prolonged mechanical ventilation, deep wound infection and long hospital stay. Predicting the risk of morbidity and mortality after cardiac surgery based on comorbidities and other clinical issues provides reference values for measuring quality performance^2–4^. However, these clinical scores cannot be used to predict vasoplegic syndrome (VS). This common complication occurs in 9–44% of the patients after cardiac surgery and is characterized as a high-output shock state with poor systemic vascular resistance. Mortality rate in this situation can be as high as 25% in this group of patients^4^.

Presently, when the medicine of the P4s focuses on systems that include predictive, personalized, preventive, and participative aspects, one of the biggest challenges is to use score risks to identify the low or high-risk patients undergoing cardiac surgery that could evolve to the seriousness or even death ^2,5^. For that reason, there is an urgent need to develop innovative tools to predict VS in patients submitted to coronary bypass surgery. A biomarker is a valuable tool in all fields of medicine, especially in cardiovascular disease when the patient has to undergo invasive surgery as on-pump CABG. MicroRNAs (miRNAs), a class of small, non-coding RNA molecules, are responsible for post-transcriptional regulation of gene expression, playing also a critical role in the physiology and also in the pathogenesis of various diseases^6,7^. In addition, miRNAs have been extensively reported as stable and predictive non-invasive biomarkers in cancer, cardiovascular and inflammatory diseases ^5,8–12^. In the present study, we screened 754 miRNAs by real-time PCR array in whole blood samples collected from patients before elective on-pump CABG and compared the miRNA profiles from patients who evolved (VASO) vs did not evolve with VS (NONVASO) after surgery. Furthermore, we performed computational analyses to assess the potential role and link between miRNAs and the pathophysiology of VS. We have identified that a combination of two miRNAs (hsa-miR-30d-5p and hsa-miR-770-5p) involved in the Apelin Liver Signaling Pathway can strongly distinguish patients who could and could not develop VS, representing a potential predictor biomarker of VS.

## Methods

### A Ethical approval and patient consent

The study was approved by the Ethical Committee of Heart Institute of the School of Medicine, University of São Paulo (HCFMUSP) (Cappesq 4435/16/101–15,380) and conducted following the guidelines of the Declaration of Helsinki. Informed consent was obtained from all patients.

### The coronary artery bypass graft surgery

In coronary artery bypass graft surgery, the patient is put under general anesthesia. The left internal thoracic artery (internal mammary artery) and the saphenous vein are used to perform a by-pass in the coronary arteries that are obstructed by the atherosclerosis process. To perform these by-passes, it is necessary to deviate the patient's blood, which arrives through the right atrium, to a cardiopulmonary bypass machine. This machine performs the function of oxygenating and pumping the blood back into the patient's aorta. In this way it is possible to perform the cardiac arrest of the patient for the confection of the sutures of the vessels, without stopping the patient's blood circulation.

### Patients’ inclusion and exclusion criteria, follow-up and diagnosis of VS

This was a case–control study nested in a cohort where 87 patients consecutively submitted to on-pump coronary artery bypass graft surgery were initially selected. Exclusion criteria were off-pump coronary artery by-pass graft surgery, patients requiring vasoactive drugs, need for intra-aortic balloon pump, presence of collagen diseases, neoplasms, or recent acute myocardial infarction (< 21 days). Of the 76 patients who passed the exclusion criteria, 15 developed VS (19.7%), the same ones who were compared with 15 controls who had undergone the same type of surgery in the same week at the institution. Therefore, a cohort of 30 samples was used for the miRNA profiling, divided into patients who developed (VASO group, n = 15) and did not (NONVASO group, n = 15) VS after surgery. The groups were also compared concerning peri, intra and postoperative variables. (Table 1).

**Table 1:** A cohort of 30 samples was divided into patients who developed (VASO group, N = 15) and did not (NONVASO group, N = 15) VS after surgery. The groups were compared concerning peri, intra and postoperative variables. Data are presented as number of patients and percentage in brackets for dichotomous variable or median and inter-quartile-range (IQR) or standard deviation (S.D). The symbols *** represent (*P* < 0.0001).

Clinical parameters of the patients under study		
Perioperative parameters	NONVASO group (N = 15)	VASO group (N = 15)
Median age (IQR)—yr	66.0 (49.0–76.0)	68.0 (48.0–76.0)
EuroSCORE—Mean (SD)	1.1 (0,4)	0.9 (0.4)
STS—Mean (SD)	0.5 (0.3)	0.7 (0.2)
Male—no.(%)	14 (93.3)	14 (93.3)
Body Mass Index (BMI), kg/m^2^—Mean (SD)	25.6 (2.2)	25.9 (3.5)
Diabetes mellitus—no. (%)	9 (60.0)	9 (60.0)
Hypertension—no. (%)	10 (66.7)	13 (86.7)
Family history of coronary heart disease—no. (%)	1 (6.6)	1 (6.6)
Renal failure—no. (%)	1 (6.6)	1 (6.6)
Peripheral artery disease—no. (%)	1 (6.6)	3 (20)
Previous stroke	1 (6.6)	3 (20)
Prior percutaneous coronary intervention (PCI)—no. (%)	3 (20)	2 (13.3)
Hemoglobin (mg / dL)—Mean (SD)	14.16 (1.7)	14.4 (1.3)
Hematocrit—% (SD)	42.9 (3.8)	42.3 (5.1)
Glycosylated Hemoglobin (mg / dL)—Mean (SD)	6.3 (1.0)	6.5 (1.2)
Glucose (mg/dL)—Mean (SD)	133.8 (41.1)	130.3 (47.8)
Creatinine, (mg / dL) – Mean (SD)	1.0 (0.26)	1.1 (0.18)
Coronary vessels involvement:		
Two-vessel disease—no. (%)	3 (20)	1 (6.7)
Tree-vessel disease—no. (%)	12 (80)	14 (93.3)
ACE inhibitor / ARB use (48 h before surgery)—no. (%)	9 (60)	7 (46,6)
Betablocker (24 h before surgery)	10 (67)	7 (46,6)
Calcium Chanel blocker (more than 2 weeks before surgery)	3 (20)	5 (33,3)
Ejection fraction—% (SD)	59 **(10.7**)	60.8 (6.1)

Intraoperative parameters	NONVASO group (N = 15)	VASO group (N = 15)
CPB time—minutes	101	85
Lower intraoperative hemoglobin (mg/dL)—Mean (SD)	9.3	8.4
Aortic cross clamp time (minutes)—Mean (SD)	69.93 (19.92)	75.60 (15.95)
Higher intraoperative blood glucose (mg/dL)—Mean (SD)	208,3**	173,8**
Transfusion of blood products during surgery—no. (%)	1(6.6)	1(6.6)

Postoperative parameters	NONVASO group (N = 15)	VASO group (N = 15)
Creatinine, (mg / dL)—Mean (SD)	1.5 (0.5)	1.3 (0.4)
ICU stay time (hours)—Mean (SD)	325.6 (257.9)	234.4 (165.7)
Hemoglobin (mg / dL)—Mean (SD)	10.8 (1.6)	10.3 (1.0)
CKMB peak (hours)—Mean (SD)	34.3 (28.5)	54.5 (47.5)
Troponin I peak (hours)—Mean (SD)	11.4 (11.8)	17.9 (16,1)

Two venous blood samples of 5 ml were collected from all the patients during surgery. The first one, after anesthetic induction, during the passage of the central venous catheter. The second, after leaving cardiopulmonary bypass, at least 15 min after complete reversal of heparin with protamine. At the end of the surgery, the patient was referred to the postoperative ICU still with a cardiac output monitor and followed daily by a member of the research group, to ascertain the clinical parameters and to diagnose VS in the first 48 h post-surgery. All patients were monitored, with a Flo Trac sensor attached to the EV1000 monitor, both from Edwards Lifescience, to monitor the cardiac index. The diagnostic criteria for VS adopted in this study was as previously described at VANCS Trial^13^. Briefly, VS was defined as hypotension (MAP < 65 mmHg) refractory to at least 1000 mL of intravenous crystalloid fluid infusion, associated with a cardiac index greater than 2.2 L/min2/m2, within the first 48 h after CPB. Other causes of shock needed to be ruled out such as cardiogenic shock, hypovolemic shock or cardiac tamponade. During follow-up, clinical and laboratory data of the included patients were collected for the Research Electronic Data Capture (REDCAP) database. During follow-up, clinical and laboratory data of the included patients were collected for the Research Electronic Data Capture (REDCAP) database.

### Whole blood RNA extraction and miRNA profiling

Whole blood for RNA extraction was collected from each patient in Tempus ™ Blood RNA tubes and stored at − 80℃ and RNA extracted according to the manufacturer's protocol, using the Tempus ™ 12-Port RNA Isolation kit (Thermofisher Scientific- USA). Total RNA integrity and concentration were accessed by using Nanodrop 2000 (Thermofisher Scientific, EUA) and Bioanalyzer equipment (Agilent, Germany). MiRNA profiling of the selected patients was performed on the AB7900 instrument (Applied Biosystems, USA) using microfluidic card TaqMan™ Array Human MicroRNA A + B Cards- TLDA- v2.0), which allows quantitative assessment of 754 miRNAs, including endogenous genes. Briefly, we used a multiplexed RT reaction (Megaplex™ RT stem-loop primers, Human Pool Set v3.0 kit) to produce cDNA from 100 ng isolated total RNA in a final volume of 7.5 μL, containing 3 μL of total RNA and 4.5μL of the RT mix (0.8 μL Megaplex ™ RT Primers (10x), 0.20 μL dNTPs (100 mM), 1.5μL MultiScribe TM Reverse Transcriptase enzyme (50U / μL), 0.8 μL RT buffer (10x), 0.9 μL MgCl2 (25 mM), 0.10 μL RNAse inhibitor (20U / μL) and 0.20 μL of nuclease-free water. Pre-amplification was performed with 2.5 µL of the reverse transcribed product and 22.5 µL of the pre-amplification mix (Thermofisher, USA), both steps were performed in a Veriti® Thermocycler equipment (Applied Biosystems, USA). The obtained pre-amplified product and Real-time PCR master mix (TaqMan Universal Master Mix II, no UNG, Thermofisher, USA) were loaded into pre-printed TaqMan Low-Density Arrays.

### Unsupervised analysis of miRNA profiling and statistical analysis for biomarker potential

To analyze the differences in miRNA levels between the two groups, we uploaded the real-time generated raw data files (extension.SDS) in a Thermofisher Cloud software v1.0. The data files were first pre-processed using automatic baseline corrections and manually checked for each assay if the Ct value corresponded to the midpoint of the logarithmic amplification curve. The comparative threshold cycle method was used to calculate the relative miRNA levels (∆Ct) after global mean normalization. MiRNAs with a mean Ct > 36 and detected in < 80% of all samples were considered below the detection level and excluded from further analysis. The miRNAs with threshold values of *P* ≤ 0.05 and absolute FC ≥ 1.5 were considered with dysregulated levels (increased or decreased). For analyses for individual miRNAs, the paired t-test was used for variables that followed the normal distribution (Anderson–Darling test), and for the others, the paired Mann–Whitney non-parametric test was used. To analyze the association between outcome and explanatory variables, simple and multiple logistic regression models were used, and, to evaluate this model, C-Statistics (AUC) was used. Prognostic measures of biomarker potential were calculated using the cutoff point by the Youden method evaluating sensitivity, specificity, accuracy, positive and negative predictive value. Two-tailed hypotheses were considered and a *P*-value < 0.05 was considered significant. GraphPad Prism software version 8.01 was used to perform individual analyses and ROC curves.

### miRNA target prediction and enrichment pathway analyses

Target prediction and identification of putative targets of the 7 dysregulated miRNAs in VASO vs NONVASO was performed using the software Ingenuity Pathways Analysis (IPA) (Qiagen, USA- www.ingenuity.com) which relies on three database algorithms (TargetScan, TarBase, and miRecords). IPA canonical pathways and network analyses use a graphical database of networks of interacting genes (Ingenuity Knowledge Base, IKB). A list containing the seven dysregulated miRNAs was uploaded in the IPA and analyzed based on the content of date 2022–07 using Fisher´s exact test to measure the significance of the association between each list and the enriched pathway. Molecules are represented as nodes, and the biological relationship between two nodes is represented as an edge (line). Chord diagrams were created to represent the miRNAs and their putative targets related to the apelin signaling pathways by using web-based software (Datasmith.org—Copyright 2021 Ben Peterson).

### Assessing the downstream effect of miRNAs on pathways and molecules

In order to predict the downstream effect of the activation or inhibition of molecules targeted by miRNAs in each canonical pathway, we used an IPA tool called “Molecule Activity Predictor” (MAP). First, we found the miRNA targets within each pathway and then, overlaid the fold change values of each dysregulated miRNA, or interactively specifying activation in silico, manually assigned, considering an inverse expression relationship between the miRNA and its targets. MAP enabled the visualization of the overall effect on the pathway producing results where molecules or functions that are predicted to be activated or inhibited are colored in shades of orange and blue, respectively, based on a calculated z-score. The z-score calculation algorithm is based on the IKB data as of July 2022, that divides the molecules in the pathways into two sets: set 1; molecules for which activity are colored in red and green when upregulated or downregulated, respectively, is known based on our overlayed data, e.g., dysregulated miRNAs or specified manually, e.g., putative miRNA targets and set 2; molecules for which activity is unknown. The software calculates the number of molecules with "unknown" activity that is connected to the molecules with "known" activity by certain types of relationships, and based on protein activity (expression, transcription, activation, inhibition, phosphorylation) it assigns its activity as increased or decreased.

## Results

### Characteristics of the study cohort

**Figure 1:**
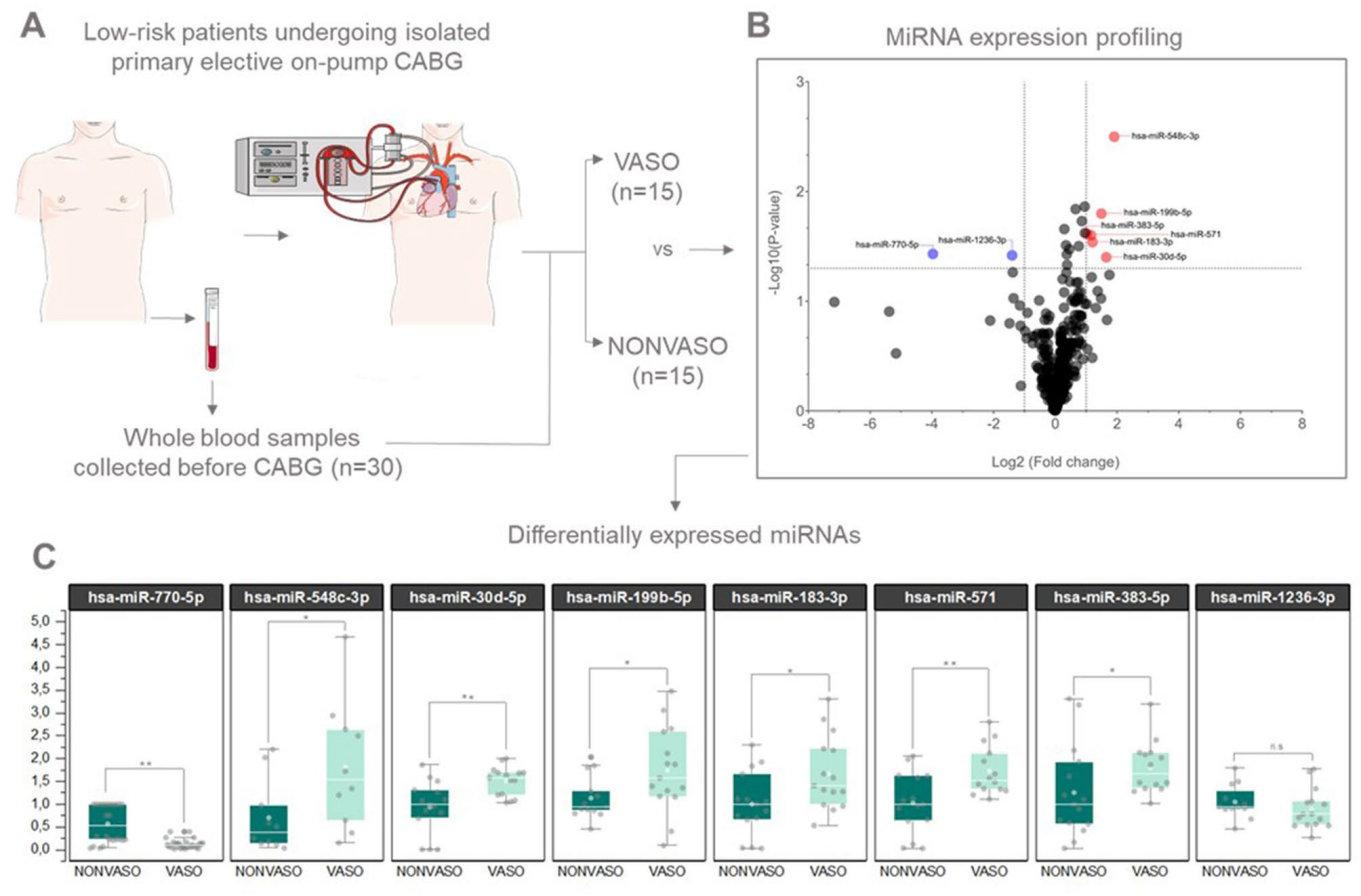
(**A**) Workflow of the study design: A cohort of 30 samples was used for the miRNA profile, divided into patients who developed (VASO group, N = 15) and did not (NONVASO group, N = 15) VS after surgery. (**B**) Volcano plot showing that among the 754 screened miRNAs, eight were differentially expressed in the VASO group compared to NONVASO, from those, six miRNAs were increased (red dots): hsa-miR-548c-3p, hsa-miR-30d-5p, hsa-miR-199b-5p, hsa-miR-183-3p, hsa-miR-571, hsa-miR-383-5p and two were decreased (blue dots): hsa-miR-1236-3p, hsa-miR-770-5p. (**C**) Mann–Whitney non-parametric test and, for outlier’s detection, the ROUT method (based on the False Discovery Rate—FDR) was used to compare both groups, using the values of ∆Ct after global mean normalization and calculation of the relative expression 2^-∆∆Ct^ (fold change) for each miRNA. Considering *P*-value ≤ 0.05 as statistically significant. Figure 1A was partly generated using Servier Medical Art, provided by Servier, licensed under a Creative Commons Attribution 3.0 unported license.

A workflow of the study design is shown in Fig. 1A. The clinical characteristics of the patients are summarized in Table 1, noting that there was no statistical difference in preoperative and post-operative variables comparing both groups. We also didn´t find significant differences in EuroSCORE II and STS values, usually employed to predict risk in heart surgery. There was statistically higher-level difference (*P* < 0.0047) in the higher intraoperative blood glucose parameters in the VASO compared to NONVASO groups depicted in Table 1.

### Circulating miRNAs dysregulated in blood samples of patients who underwent on-pump CABG and that developed versus failed to develop VS during the preoperative.

We identified among the 754 screened miRNAs, eight dysregulated miRNAs in the whole blood of VASO versus NONVASO patients, as shown in a volcano plot, (Fig. 1B). Six miRNAs were with increased levels (red dots): hsa-miR-548c-3p, hsa-miR-30d-5p, hsa-miR-199b-5p, hsa-miR-183-3p, hsa-miR-571, hsa-miR-383-5p and two were with decreased levels (blue dots): hsa-miR-1236-3p, hsa-miR-770-5p. Supplementary Table 1 (Table S1) shows the list of the dysregulated miRNAs, their symbol, miRbase ID, fold change, and p-values. As an aid to interpretation, we sorted the dysregulated miRNAs and represented them in grouped box plots (Fig. 1C). We performed paired Mann–Whitney non-parametric test and, for detection of outliers, the ROUT method (based on the False Discovery Rate—FDR). After these analyses we observed that seven out of eight miRNAs were statistically significant (*P* ≤ 0.05) comparing VASO versus NONVASO, hsa-miR-1236-3p didn´t pass the *P* -value cutoff after the Mann–Whitney non-parametric statistical analysis test.

### Combination of miR-30d-5p and hsa-miR-770-5p in whole blood as potential predictor of VS

**Figure 2:**
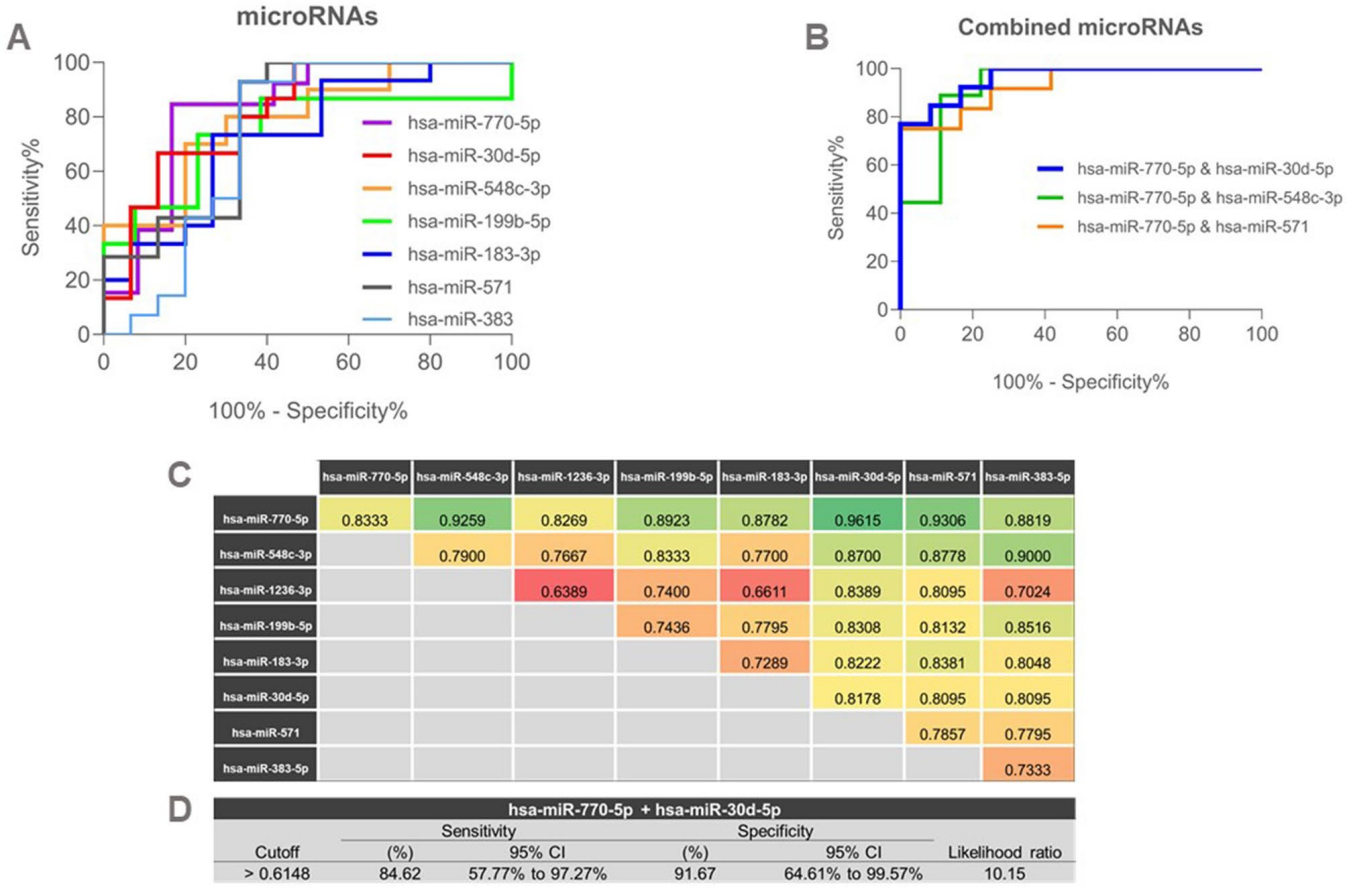
(**A, B**) ROC curve for each single miRNAs yielded the top 2 highest AUC values of 0.8333 [95% confidence interval (CI), 0.6622–1.000; *P*-value = 0.0047] and 0.8178 (95% CI, 0.6640–0.9715; *P*-value = 0.0030) for hsa-miR-770-5p and hsa-miR-30d-5p, respectively. (**C**) The combination of these two miRNAs yielded an AUC value of 0.9615 (95%CI, 0.973–0.999; *P* < 0.0001) (**D**) The combination showed 84.6% sensitivity and 91.67% specificity in distinguishing patients from VASO from NONVASO groups.

To explore the potential of miRNAs to predict VS before CABG, ROC curves were generated for single miRNAs and for all possible combinations between two miRNAs, to analyze their synergistic diagnostic value (Fig. 2A–C and Supplementary Table 2 (Table S2). ROC curves for each single miRNAs yielded the top 2 highest AUC values of 0.8333 [95% confidence interval (CI), 0.6622–1.000; *P*-value = 0.0047] and 0.8178 (95% CI, 0.6640–0.9715; *P*-value = 0.0030) for hsa-miR-770-5p and hsa-miR-30d-5p, respectively (Supplementary Table 2). The combination of these two miRNAs yielded an AUC value of 0.9615 (95%CI, 0.973–0.999; *P* < 0.0001) with 84.6% sensitivity and 91.67% specificity in distinguishing patients from VASO from NONVASO groups (Fig. 2D) showed a superior biomarker power to that of a single miRNA. We also performed ROC curve analyses using the combination of the two miRNAs, hsa-miR-770-5p and hsa-miR-30d-5p with the values of higher intraoperative blood glucose (HIG) parameters and we observed an AUC value increase compared to the one from the combination of the two miRNAs (from 0.9615 to 0.974) as shown in Supplementary Fig. 1.

### MiRNA target prediction and gene set enrichment analysis reveals canonical pathways potentially modulated in patients developing VS

**Figure 3:**
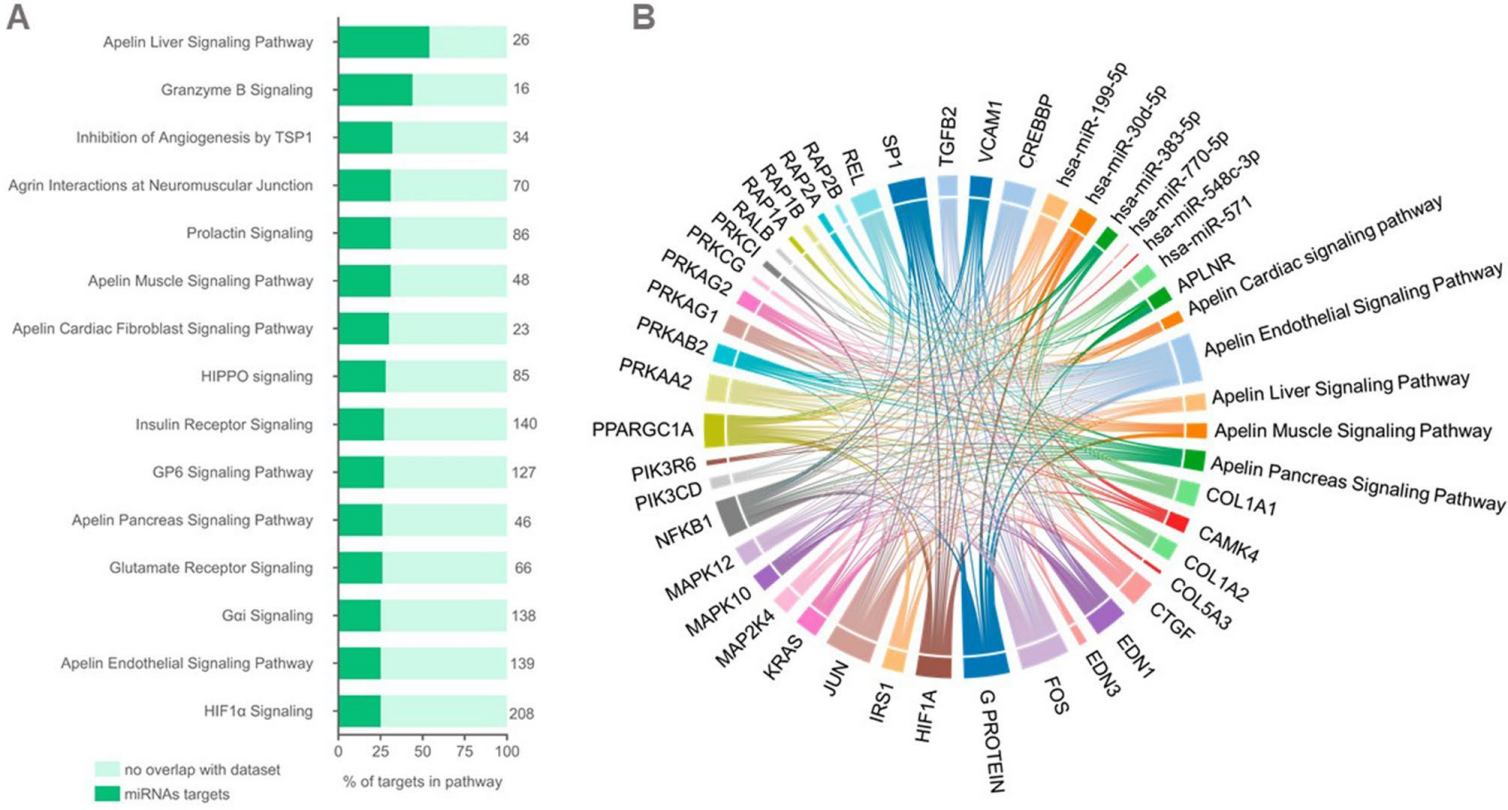
(**A**) Top 15 canonical pathways most significantly enriched represented as stacked bar charts. The total number of molecules within each pathway is at the top of each plotted bar. The log (*p*-values) were Benjamini–Hochberg corrected with a cutoff of 1.3. The top enriched pathway was “Apelin Liver Signaling Pathway” with 14 out of 26 molecules within the pathway (53.8%) containing the higher number of targets of the dysregulated miRNAs. (**B**) Chord diagram representing the amount of connection between the four canonical pathways related to the Apelin Signaling Pathway: in the liver, endothelial, muscle/cardiomyocyte, cardiac fibroblasts, and pancreas, with several entities or nodes (gene/protein/miRNAs) within these pathways or indirectly connected with the pathway nodes. The more interrelatedness and connections the node has more relevant it is and also represented by different colors. The size was proportional to the number of connections. In this particular diagram, the colors of the chords partially mixed with the colors of their connected entities.

We performed computational analyses to identify all potential targets of the seven dysregulated circulating miRNAs in VASO patients. Supplementary Table 3 contain the list of 3699 targets of the seven miRNAs. We obtained the targets list from three different target prediction databases, considering those predicted as moderate, high, or experimentally validated as targets. The genes predicted to be targets of the dysregulated miRNAs in VASO patients were significantly enriched in canonical pathways related to immune response, cellular stress, and injury and cardiovascular signaling. Figure 3A shows the top 15 canonical pathways most significantly enriched in stacked bar charts. The total number of molecules within each pathway is at the top of each plotted bar. In this analysis, we applied a Benjamini–Hochberg corrected-log (*P*-value) cutoff of 1.3. The top enriched pathway was “Apelin Liver Signaling Pathway” with 14 out of 26 molecules within the pathway (53.8%) containing the higher number of potential targets of the dysregulated miRNAs. In the list of most enriched pathways, we also identified another four pathways related to the apelinergic system e.g., Apelin signaling pathways in cardiac fibroblast, muscle, pancreas and endothelial cells. Figure 3B shows as a chord diagram the amount of connection between the four canonical pathways related to the apelin signaling pathways with several entities or nodes (gene/protein/miRNAs) within these pathways or indirectly connected with their nodes. The more interrelatedness and connections the node has more relevant it is and also represented by different colors. The size was proportional to the number of connections with the colors of the chords partially mixed with the colors of their connected entities. The IDs and names of all molecules shown in the chord diagram are listed in Supplementary Table 4.

### Key molecules from the apelin signaling pathways are the potential targets of the dysregulated circulating miRNAs in patients who evolved with VS.

**Figure 4:**
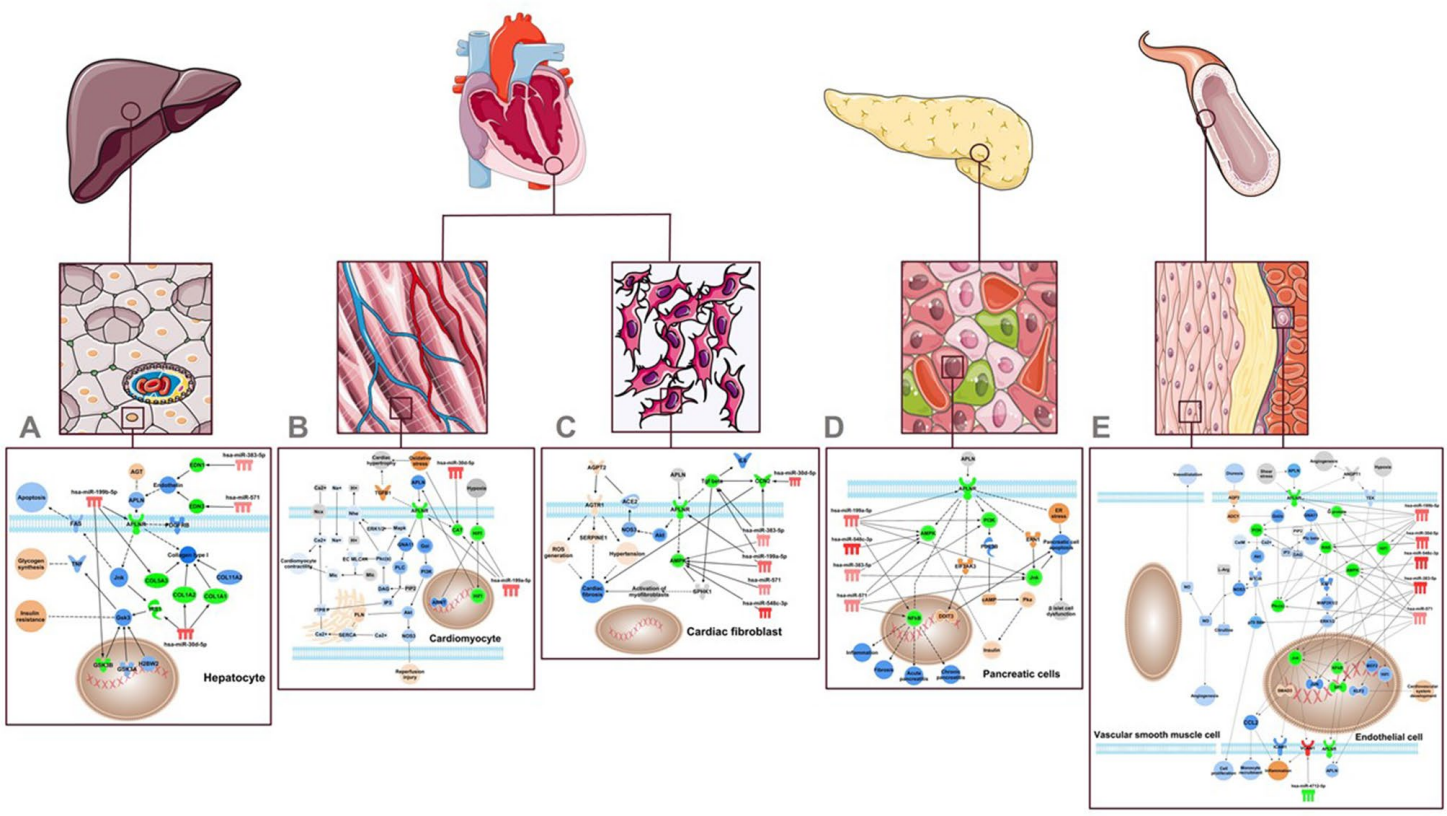
The Apelin signaling pathway represented in different organs/tissues/cells in the body: (**A**) hepatocytes, (**B**) cardiomyocytes, (**C**) cardiac fibroblasts, (**D**) pancreatic cells, (**E**) endothelial cells and vascular smooth muscle cells. Six miRNAs have potential targets in each apelin canonical pathway. Based on their expression, these molecules were colored in red and green when upregulated or downregulated, respectively. MAP enabled the visualization of the overall effect on the pathway producing results where molecules or functions that are predicted to be activated or inhibited are colored in shades of orange and blue, respectively. Figures 4A to E were partly generated using Servier Medical Art, provided by Servier, licensed under a Creative Commons Attribution 3.0 unported license.

A significant proportion of potential targets of the dysregulated miRNAs in VASO group belong to canonical apelin signaling pathways. Six miRNAs targeted the apelin canonical pathways, as Fig. 4A–E show e.g., the downregulated hsa-miR-770-5p and other five, upregulated, in VASO patients: hsa-miR-199a-5p, hsa-miR-30d-5p, hsa-miR-383-5p, hsa-miR-548c-3p, hsa-miR-571. This pathway is key in different organs/tissues/cells in the body e.g., hepatocytes (Fig. 4A), cardiomyocytes (Fig. 4B) and cardiac fibroblasts (Fig. 4C), pancreatic cells (Fig. 4D), endothelial cells, and vascular smooth muscle cells (Fig. 4E). Three nodes are present in all five pathways shown in Figs. 4A–E: Apelin (APLN), hsa-miR-199-5p and its predicted target Apelin receptor (APLNR). In hepatocytes (Fig. 4A), hsa-miR-199-5p has three predicted targets: the apelin receptor (APLNR), the collagen type 1 (COL5A3) and the glycogen synthase kinase 3 beta (GSK3b). Endothelin 1 (EDN1) and endothelin 3 (EDN3), are predicted targets of hsa-miR-383-5p and hsa-miR-571 respectively, both previously shown able to increase the expression of APLN. Angiotensin 2 (AGT) has an inhibitory effect in APLN. Hsa-miR-30d-5p has three predicted targets: insulin receptor substrate 1 (IRS1), collagen type I alpha chain 1 and 2 (COL1A1 and COL1A2). In cardiomyocytes (Fig. 4B), the hsa-miR-199-5p has three predicted targets: APLNR, the transcription factor hypoxia-inducible factor 1 subunit alpha (Hif1) and, the enzyme catalase (CAT) which is also a predicted target of hsa-miR-30d-5p. In cardiac fibroblasts (Fig. 4C), hsa-miR-199-5p and other three miRNAs: hsa-miR-30d-5p, hsa-miR-571 and hsa-miR-548c-5p have AMP-activated kinase (AMPK) as a target in common. These same four miRNAs have different targets within the Apelin signaling in pancreatic cells (Figs. 4D) in endothelial and vascular muscle cells (Fig. 4E) leading to a predicted increase in insulin resistance and decrease in vasodilatation, respectively. In endothelial cells, hsa-miR-770-5p is downregulated in the VASO group and has vascular cell adhesion molecule 1 (VCAM1) as a target, a cell surface sialoglycoprotein expressed by cytokine-activated endothelium.

## Discussion

In the present case–control study nested in a cohort of patients who developed VS we showed, for the first time, a possible link between miRNA and VS. Our results provide clues about miRNA signatures in this complication that affects 9–44% of patients after cardiac surgery and with a mortality rate of 25%. Patients with VS develop a systemic inflammatory response leading to endothelial dysregulation, systemic vasodilation refractory to vasopressors, hypotension and progressive death^13^. Despite the lack of information regarding miRNAs in VS, some inflammatory diseases, such as sepsis^14^, systemic inflammatory response syndrome^15^, and sepsis-related acute lung injury^16^, that have a similar mechanism as VS, were studied and also show a correlation with dysregulation levels of hsa-miR-30d-5p in body fluids^15–17^. Here we show that in comparison to healthy controls, the levels of miR-30d-5p are increased in whole blood of VS patients, suggesting a possible anti-inflammatory role for this miRNA^17^. A study in patients with heart failure and desynchrony (HF_DYS_), showed that miR-30d appears to be regulated by heart wall stress, consistent with its increased expression in the lateral wall in a canine model of HF_DYS_, which appears to play a functional role in cardiomyocyte biology, mediating cardiac hypertrophy with a distinct molecular signature and protecting against TNF-α–induced apoptosis and deleterious signaling^18^. This information could suggest that miRNA-30d might also play a similar role in VS, regulating the inflammatory response and endothelial gene expression dysregulation. MiRNA-770 was previously shown to be associated in regulation of apoptosis and cell proliferation^19^ and in different disease states as in diabetes^20^, dilated cardiomyopathy^21^, and different types of cancers^19^, such as non-small cell lung^22^, breast^23^ and gastric cancer^24^. Here, we show, by computational prediction analyses, that the dysregulated miRNAs have a potential role in controlling targets related to the apelinergic system, responsible for signaling events related to severe and refractory hypotension, a common outcome in patients who progress to VS^25^. One of the potential targets of the dysregulated miRNAs, described here, is the receptor of apelin (APLNR), a protein secreted from endothelial cells and induced by angiopoietin 1 (Ang1) which functions as regulator of vascular tone and blood pressure. Apelin/APLNR are highly expressed in endothelial cells ^26^ acting in the vasodilatation mechanism as well in the regulation of blood vessel diameter during angiogenesis^27^. Ishida et al., demonstrated that the acute administration of apelin significantly decreased the systolic blood pressure in wild type mice, but was unable to change the systolic blood pressure in mice deficient for APLNR. Interestingly, the hypotensive effect of apelin was also observed in chronic hypertensive model animals^27^. Studies in rats revellead that apelin/APLNR are localized within the endothelia of small arteries in several organs such as the liver, spleen, lung, pancreas, intestine, kidney, and adipose tissues^26^. Apelin causes endothelium-dependent vasorelaxation by triggering the release of nitric oxide (NO), and this effect can be almost completely abolished in the presence of endothelial NO synthase (eNOS) inhibitor, suggesting that the apelin/APLNR pathway exerts a vasorelaxation effect via activation of the eNOS pathway^28,29^. Although we obtained statistically significant results and we show here that the measured levels of miRNAs, hsa-miR-30d and hsa-miR-770-5p have a potential ability to distinguish patients who develop VS from those who didn´t, we acknowledge the main limitation of the present work, which is the small number of participants. However, the findings of this study offer new, potentially useful information to improve the prognostic outcome of VS and also opening vistas to a potential new clinical biomarker to help the management of patients referred to cardiac surgery. The computational analysis also points out to the importance in further investigate the miRNAs/apelin/APLNR signaling pathway in the refractory hypotension observed in patients who develop VS.

## Supplementary Information

Supplementary Information.

## Data Availability

All data generated or analyzed during this study are included in this published article (and its Supplementary Information files).

## Abbreviations

CABG: Coronary artery bypass grafting
VS: Vasoplegic syndrome
MiRNA: MicroRNA
EuroScore II: European System for Cardiac Operative Risk Evaluation
STS: Society of Thoracic Surgeons.
APLNR: Apelin receptor
COL5A3: Collagen type 1
GSK3b: Glycogen synthase kinase 3 beta
EDN1: Endothelin 1
EDN3: Endothelin 3
AGT: Angiotensin 2
IRS1: Insulin receptor substrate 1 and 2
COL1A1: Collagen type I alpha chain 1
COL1A2: Collagen type I alpha chain 2
Hif1: Hypoxia-inducible factor 1 subunit alpha
CAT: Catalase
AMPK: AMP-activated kinase
VCAM1: Vascular cell adhesion molecule 1
