# Supplementary material for: MiRNA-30d and miR-770-5p as potential clinical risk predictors of Vasoplegic Syndrome in Patients undergoing on-pump coronary artery bypass grafting
Source: Sci Rep. 2023 Feb 6;13:2084. doi: 10.1038/s41598-023-28978-2 (PMC9902624; doi:10.1038/s41598-023-28978-2)
Supplement: Supplementary file 1 — Supplementary Information. [file 41598_2023_28978_MOESM1_ESM.pdf]

Supplementary Figure 1

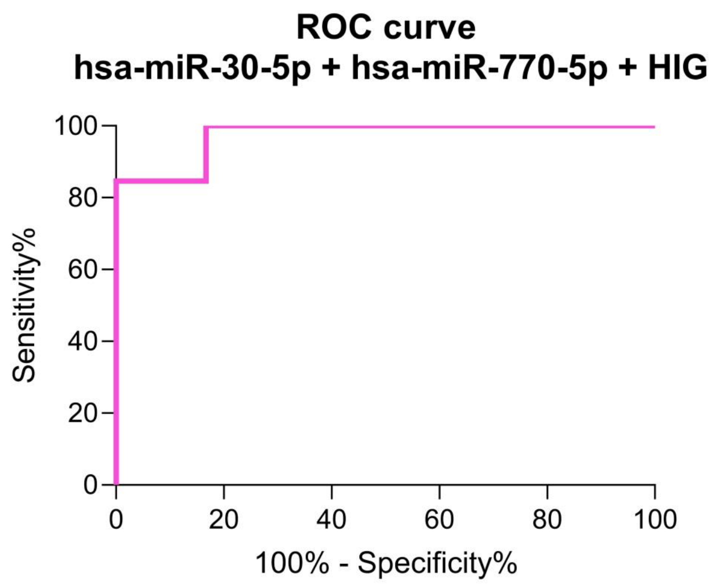


| Area under the ROC curve | |
|---|---|
| Area | 0.9744 |
| Std. Error | 0.02585 |
| 95% confidence interval | 0.9237 to 1.000 |
| P value | <0.0001 |

**Supplementary Figure 1.** ROC curve analyses using the combination of the two miRNAs, hsa-miR-770-5p and hsa-miR-30d-5p with the values of higher intraoperative blood glucose (HIG) parameters showed an AUC of 0.974 and *P*-value < 0.0001).

**Supplementary Table 1**. List of the dysregulated miRNAs, their symbol, miRbase ID, fold change, and p-values, considering $P<0.05$ as statistically significant.

| **Table-S1. List of differentially expressed circulating miRNAs (VASO vs NONVASO)** | | | |
|---|---|---|---|
| **miRNA symbol** | **miRbaseID** | **Fold Change** | **p-value** |
| hsa-miR-548c-3p | MIMAT0003285 | 3.7 | 0.00317 |
| hsa-miR-30d-5p | MIMAT0000245 | 3.1 | 0.03974 |
| hsa-miR-199b-5p | MIMAT0000263 | 2.8 | 0.01588 |
| hsa-miR-183-3p | MIMAT0004560 | 2.3 | 0.02891 |
| hsa-miR-571 | MIMAT0003236 | 2.2 | 0.02505 |
| hsa-miR-383-5p | MIMAT0000738 | 2.0 | 0.02417 |
| hsa-miR-1236-3p | MIMAT0005591 | -2.6 | 0.03820 |
| hsa-miR-770-5p | MIMAT0003948 | -15.6 | 0.03709 |

**Supplementary Table 2**. List of AUC and p-values for all possible combinations between two miRNAs, or miRNA alone.

**Table-S2. Area Under de Curve (AUC) and statistical significance (p<0.05) for each single miRNA and all possible combinations between two miRNAs**

| miRNA (s) | AUC | Std. Error | p-value |
|---|---|---|---|
| hsa-miR-770-5p + hsa-miR-30d-5p | 0.9615 | 0.03282 | <0.0001 |
| hsa-miR-770-5p + hsa-miR-571 | 0.9306 | 0.04953 | 0.0003 |
| hsa-miR-770-5p + hsa-miR-548c-3p | 0.9259 | 0.06610 | 0.0023 |
| hsa-miR-548c-3p + hsa-miR-383 | 0.9000 | 0.07438 | 0.0033 |
| hsa-miR-770-5p + hsa-miR-199b-5p | 0.8923 | 0.06608 | 0.0016 |
| hsa-miR-770-5p + hsa-miR-383 | 0.8819 | 0.07928 | 0.0015 |
| hsa-miR-770-5p + hsa-miR-183-3p | 0.8782 | 0.06802 | 0.0013 |
| hsa-miR-548c-3p + hsa-miR-571 | 0.8778 | 0.08617 | 0.0055 |
| hsa-miR-548c-3p + hsa-miR-30d-5p | 0.8700 | 0.09038 | 0.0052 |
| hsa-miR-199b-5p + hsa-miR-383 | 0.8516 | 0.07680 | 0.0019 |
| hsa-miR-1236-3p + hsa-miR-30d-5p | 0.8389 | 0.07519 | 0.0029 |
| hsa-miR-183-3p + hsa-miR-571 | 0.8381 | 0.07669 | 0.0019 |
| hsa-miR-770-5p | 0.8333 | 0.08729 | 0.0047 |
| hsa-miR-548c-3p + hsa-miR-199b-5p | 0.8333 | 0.09417 | 0.0143 |
| hsa-miR-199b-5p + hsa-miR-30d-5p | 0.8308 | 0.07644 | 0.0030 |
| hsa-miR-770-5p + hsa-1236-3p | 0.8269 | 0.08819 | 0.0055 |
| hsa-miR-183-3p + hsa-miR-30d-5p | 0.8222 | 0.08078 | 0.0026 |
| hsa-miR-30d-5p | 0.8178 | 0.07845 | 0.0030 |
| hsa-miR-199b-5p + hsa-miR-571 | 0.8132 | 0.08127 | 0.0057 |
| hsa-miR-1236-3p + hsa-miR-571 | 0.8095 | 0.08880 | 0.0075 |
| hsa-miR-30d-5p + hsa-miR-571 | 0.8095 | 0.08262 | 0.0046 |
| hsa-miR-30d-5p + hsa-miR-383 | 0.8095 | 0.08190 | 0.0046 |
| hsa-miR-183-3p + hsa-miR-383 | 0.8048 | 0.08081 | 0.0052 |
| hsa-miR-548c-3p | 0.7900 | 0.10230 | 0.0284 |
| hsa-miR-571 | 0.7857 | 0.08836 | 0.0088 |
| hsa-miR-199b-5p + hsa-miR-183-3p | 0.7795 | 0.08949 | 0.0121 |
| hsa-miR-571 + hsa-miR-383 | 0.7795 | 0.08649 | 0.0121 |
| hsa-miR-548c-3p + hsa-miR-183-3p | 0.7700 | 0.10640 | 0.0413 |
| hsa-miR-548c-3p + hsa-miR-1236-3p | 0.7667 | 0.11150 | 0.0500 |
| hsa-miR-199b-5p | 0.7436 | 0.09859 | 0.0287 |
| hsa-miR-1236-3p + hsa-miR-199b-5p | 0.7400 | 0.10150 | 0.0458 |
| hsa-miR-383 | 0.7333 | 0.10170 | 0.0325 |
| hsa-miR-183-3p | 0.7289 | 0.09323 | 0.0327 |
| hsa-miR-1236-3p + hsa-miR-383 | 0.7024 | 0.11060 | 0.0803 |
| hsa-miR-1236-3p + hsa-miR-183-3p | 0.6611 | 0.10570 | 0.1571 |
| hsa-miR-1236-3p | 0.6389 | 0.11030 | 0.2225 |

Sup. Table 3. List of 3699 targets of the seven dysregulated miRNAs obtained from three different target prediction databases,TargetScan TarBase and miRecords considering those predicted as moderate, high, or experimentally validated, respectively.

| miRbaseID | miRNA Symbol | Fold Change | Source | geting prediction confide | Gene target symbol | Entrez Gene target Name | Location | Molecule Type |
|---|---|---|---|---|---|---|---|---|
| MIMAT0003285 | miR-548c-3p (miRNAs w/seed AAAAAUC) | 3.760 | TargetScan Human | Moderate (predicted) | ANGPTL5 | angiopoietin like 5 | Extracellular Space | other |
| MIMAT0003285 | miR-548c-3p (miRNAs w/seed AAAAAUC) | 3.760 | TargetScan Human | Moderate (predicted) | APAF1 | apoptotic peptidase activating factor 1 | Cytoplasm | other |
| MIMAT0003285 | miR-548c-3p (miRNAs w/seed AAAAAUC) | 3.760 | TargetScan Human | Moderate (predicted) | ATG5 | autophagy related 5 | Cytoplasm | other |
| MIMAT0003285 | miR-548c-3p (miRNAs w/seed AAAAAUC) | 3.760 | TargetScan Human | Moderate (predicted) | ATPSCKMT | synthase c subunit lysine N-methyltransfe | Cytoplasm | enzyme |
| MIMAT0003285 | miR-548c-3p (miRNAs w/seed AAAAAUC) | 3.760 | TargetScan Human | Moderate (predicted) | C16orf87 | chromosome 16 open reading frame 87 | Other | other |
| MIMAT0003285 | miR-548c-3p (miRNAs w/seed AAAAAUC) | 3.760 | TargetScan Human | Moderate (predicted) | CAV1 | caveolin 1 | Plasma Membrane | transmembrane receptor |
| MIMAT0003285 | miR-548c-3p (miRNAs w/seed AAAAAUC) | 3.760 | TargetScan Human | Moderate (predicted) | CCL7 | C-C motif chemokine ligand 7 | Extracellular Space | cytokine |
| MIMAT0003285 | miR-548c-3p (miRNAs w/seed AAAAAUC) | 3.760 | TargetScan Human | Moderate (predicted) | CEBPZOS | CEBPZ opposite strand | Other | other |
| MIMAT0003285 | miR-548c-3p (miRNAs w/seed AAAAAUC) | 3.760 | TargetScan Human | Moderate (predicted) | CEP95 | centrosomal protein 95 | Cytoplasm | other |
| MIMAT0003285 | miR-548c-3p (miRNAs w/seed AAAAAUC) | 3.760 | TargetScan Human | Moderate (predicted) | CPSF2 | eavage and polyadenylation specific facto | Nucleus | other |
| MIMAT0003285 | miR-548c-3p (miRNAs w/seed AAAAAUC) | 3.760 | TargetScan Human | Moderate (predicted) | DCUN1D1 | ctive in cullin neddylation 1 domain contair | Nucleus | other |
| MIMAT0003285 | miR-548c-3p (miRNAs w/seed AAAAAUC) | 3.760 | TargetScan Human | Moderate (predicted) | DDX46 | DEAD-box helicase 46 | Nucleus | enzyme |
| MIMAT0003285 | miR-548c-3p (miRNAs w/seed AAAAAUC) | 3.760 | TargetScan Human | Moderate (predicted) | DTNBP1 | dystrobrevin binding protein 1 | Plasma Membrane | other |
| MIMAT0003285 | miR-548c-3p (miRNAs w/seed AAAAAUC) | 3.760 | TargetScan Human | Moderate (predicted) | E2F5 | E2F transcription factor 5 | Nucleus | transcription regulator |
| MIMAT0003285 | miR-548c-3p (miRNAs w/seed AAAAAUC) | 3.760 | TargetScan Human | Moderate (predicted) | FAM185A | mily with sequence similarity 185 member | Cytoplasm | other |
| MIMAT0003285 | miR-548c-3p (miRNAs w/seed AAAAAUC) | 3.760 | TargetScan Human | Moderate (predicted) | FUT9 | fucosyltransferase 9 | Cytoplasm | enzyme |
| MIMAT0003285 | miR-548c-3p (miRNAs w/seed AAAAAUC) | 3.760 | TargetScan Human | Moderate (predicted) | GABRA4 | -aminobutyric acid type A receptor subuni | Plasma Membrane | ion channel |
| MIMAT0003285 | miR-548c-3p (miRNAs w/seed AAAAAUC) | 3.760 | TargetScan Human | Moderate (predicted) | GAL | galanin and GMAP prepropeptide | Extracellular Space | other |
| MIMAT0003285 | miR-548c-3p (miRNAs w/seed AAAAAUC) | 3.760 | TargetScan Human | Moderate (predicted) | GIMAP7 | GTPase, IMAP family member 7 | Cytoplasm | enzyme |
| MIMAT0003285 | miR-548c-3p (miRNAs w/seed AAAAAUC) | 3.760 | TargetScan Human | Moderate (predicted) | GOT1 | glutamic-oxaloacetic transaminase 1 | Cytoplasm | enzyme |
| MIMAT0003285 | miR-548c-3p (miRNAs w/seed AAAAAUC) | 3.760 | TargetScan Human | Moderate (predicted) | HIPK2 | homeodomain interacting protein kinase 2 | Nucleus | kinase |
| MIMAT0003285 | miR-548c-3p (miRNAs w/seed AAAAAUC) | 3.760 | TargetScan Human | High (predicted) | HSPB11 | eat shock protein family B (small) member | Extracellular Space | other |
| MIMAT0003285 | miR-548c-3p (miRNAs w/seed AAAAAUC) | 3.760 | TargetScan Human | Moderate (predicted) | HSPB3 | eat shock protein family B (small) member | Cytoplasm | other |
| MIMAT0003285 | miR-548c-3p (miRNAs w/seed AAAAAUC) | 3.760 | TargetScan Human | Moderate (predicted) | HYPK | huntingtin interacting protein K | Cytoplasm | other |
| MIMAT0003285 | miR-548c-3p (miRNAs w/seed AAAAAUC) | 3.760 | TargetScan Human | Moderate (predicted) | IFNGR1 | interferon gamma receptor 1 | Plasma Membrane | transmembrane receptor |
| MIMAT0003285 | miR-548c-3p (miRNAs w/seed AAAAAUC) | 3.760 | TargetScan Human | Moderate (predicted) | KCNQ2 | um voltage-gated channel subfamily Q me | Plasma Membrane | ion channel |
| MIMAT0003285 | miR-548c-3p (miRNAs w/seed AAAAAUC) | 3.760 | TargetScan Human | Moderate (predicted) | KLRC2 | killer cell lectin like receptor C2 | Plasma Membrane | transmembrane receptor |
| MIMAT0003285 | miR-548c-3p (miRNAs w/seed AAAAAUC) | 3.760 | TargetScan Human | High (predicted) | KRTAP20-2 | keratin associated protein 20-2 | Cytoplasm | other |
| MIMAT0003285 | miR-548c-3p (miRNAs w/seed AAAAAUC) | 3.760 | TargetScan Human | Moderate (predicted) | KRTAP2-3/KRTAP2- | keratin associated protein 2-3 | Cytoplasm | other |
| MIMAT0003285 | miR-548c-3p (miRNAs w/seed AAAAAUC) | 3.760 | TargetScan Human | Moderate (predicted) | KYNU | kynureninase | Cytoplasm | enzyme |
| MIMAT0003285 | miR-548c-3p (miRNAs w/seed AAAAAUC) | 3.760 | TargetScan Human | Moderate (predicted) | LINC01465 | ng intergenic non-protein coding RNA 14 | Other | other |
| MIMAT0003285 | miR-548c-3p (miRNAs w/seed AAAAAUC) | 3.760 | TargetScan Human | Moderate (predicted) | LUZP6 | leucine zipper protein 6 | Other | other |
| MIMAT0003285 | miR-548c-3p (miRNAs w/seed AAAAAUC) | 3.760 | TargetScan Human | Moderate (predicted) | MAMDC2 | MAM domain containing 2 | Extracellular Space | other |
| MIMAT0003285 | miR-548c-3p (miRNAs w/seed AAAAAUC) | 3.760 | TargetScan Human | Moderate (predicted) | MED30 | mediator complex subunit 30 | Nucleus | transcription regulator |
| MIMAT0003285 | miR-548c-3p (miRNAs w/seed AAAAAUC) | 3.760 | TargetScan Human | Moderate (predicted) | METTL17 | methyltransferase like 17 | Nucleus | other |
| MIMAT0003285 | miR-548c-3p (miRNAs w/seed AAAAAUC) | 3.760 | TargetScan Human | Moderate (predicted) | MGST1 | microsomal glutathione S-transferase 1 | Cytoplasm | enzyme |
| MIMAT0003285 | miR-548c-3p (miRNAs w/seed AAAAAUC) | 3.760 | TargetScan Human | Moderate (predicted) | MRPS6 | mitochondrial ribosomal protein S6 | Cytoplasm | other |
| MIMAT0003285 | miR-548c-3p (miRNAs w/seed AAAAAUC) | 3.760 | TargetScan Human | Moderate (predicted) | MS4A6E | membrane spanning 4-domains A6E | Other | other |
| MIMAT0003285 | miR-548c-3p (miRNAs w/seed AAAAAUC) | 3.760 | TargetScan Human | Moderate (predicted) | MSMB | microseminoprotein beta | Extracellular Space | other |
| MIMAT0003285 | miR-548c-3p (miRNAs w/seed AAAAAUC) | 3.760 | TargetScan Human | Moderate (predicted) | MT1X | metallothionein 1X | Other | other |
| MIMAT0003285 | miR-548c-3p (miRNAs w/seed AAAAAUC) | 3.760 | TargetScan Human | Moderate (predicted) | MTERF3 | itochondrial transcription termination facto | Cytoplasm | other |
| MIMAT0003285 | miR-548c-3p (miRNAs w/seed AAAAAUC) | 3.760 | TargetScan Human | Moderate (predicted) | MTO1 | nitochondrial tRNA translation optimization | Cytoplasm | enzyme |
| MIMAT0003285 | miR-548c-3p (miRNAs w/seed AAAAAUC) | 3.760 | TargetScan Human | Moderate (predicted) | NAF1 | nuclear assembly factor 1 ribonucleoprotei | Cytoplasm | other |
| MIMAT0003285 | miR-548c-3p (miRNAs w/seed AAAAAUC) | 3.760 | TargetScan Human | Moderate (predicted) | NBEAL1 | neurobeachin like 1 | Other | other |
| MIMAT0003285 | miR-548c-3p (miRNAs w/seed AAAAAUC) | 3.760 | TargetScan Human | Moderate (predicted) | NDUFAF2 | iquinone oxidoreductase complex assemb | Cytoplasm | other |
| MIMAT0003285 | miR-548c-3p (miRNAs w/seed AAAAAUC) | 3.760 | TargetScan Human | Moderate (predicted) | OR4D1 | ctory receptor family 4 subfamily D memb | Plasma Membrane | G-protein coupled receptor |
| MIMAT0003285 | miR-548c-3p (miRNAs w/seed AAAAAUC) | 3.760 | TargetScan Human | High (predicted) | OSGIN2 | e stress induced growth inhibitor family m | Other | other |
| MIMAT0003285 | miR-548c-3p (miRNAs w/seed AAAAAUC) | 3.760 | TargetScan Human | Moderate (predicted) | OSTF1 | osteoclast stimulating factor 1 | Nucleus | transcription regulator |
| MIMAT0003285 | miR-548c-3p (miRNAs w/seed AAAAAUC) | 3.760 | TargetScan Human | High (predicted) | OXSM | 3-oxoacyl-ACP synthase, mitochondrial | Cytoplasm | kinase |
| MIMAT0003285 | miR-548c-3p (miRNAs w/seed AAAAAUC) | 3.760 | TargetScan Human | High (predicted) | PAK1 | p21 (RAC1) activated kinase 1 | Cytoplasm | kinase |
| MIMAT0003285 | miR-548c-3p (miRNAs w/seed AAAAAUC) | 3.760 | TargetScan Human | Moderate (predicted) | PHF10 | PHD finger protein 10 | Nucleus | other |
| MIMAT0003285 | miR-548c-3p (miRNAs w/seed AAAAAUC) | 3.760 | TargetScan Human | High (predicted) | PHF21A | PHD finger protein 21A | Nucleus | other |
| MIMAT0003285 | miR-548c-3p (miRNAs w/seed AAAAAUC) | 3.760 | TargetScan Human | Moderate (predicted) | POLR2H | RNA polymerase II, I and III subunit H | Nucleus | enzyme |
| MIMAT0003285 | miR-548c-3p (miRNAs w/seed AAAAAUC) | 3.760 | TargetScan Human | Moderate (predicted) | POP4 | OP4 homolog, ribonuclease P/MRP subu | Nucleus | enzyme |
| MIMAT0003285 | miR-548c-3p (miRNAs w/seed AAAAAUC) | 3.760 | TargetScan Human | Moderate (predicted) | PRKAA2 | n kinase AMP-activated catalytic subunit a | Cytoplasm | kinase |
| MIMAT0003285 | miR-548c-3p (miRNAs w/seed AAAAAUC) | 3.760 | TargetScan Human | Moderate (predicted) | PROK2 | prokineticin 2 | Extracellular Space | other |
| MIMAT0003285 | miR-548c-3p (miRNAs w/seed AAAAAUC) | 3.760 | TargetScan Human | Moderate (predicted) | PRSS22 | serine protease 22 | Extracellular Space | peptidase |
| MIMAT0003285 | miR-548c-3p (miRNAs w/seed AAAAAUC) | 3.760 | TargetScan Human | Moderate (predicted) | RFESD | Rieske Fe-S domain containing | Other | other |
| MIMAT0003285 | miR-548c-3p (miRNAs w/seed AAAAAUC) | 3.760 | TargetScan Human | Moderate (predicted) | RILPL2 | Rab interacting lysosomal protein like 2 | Cytoplasm | other |
| MIMAT0003285 | miR-548c-3p (miRNAs w/seed AAAAAUC) | 3.760 | TargetScan Human | Moderate (predicted) | RP11_219B46 | | Other | other |
| MIMAT0003285 | miR-548c-3p (miRNAs w/seed AAAAAUC) | 3.760 | TargetScan Human | Moderate (predicted) | RPL24 | ribosomal protein L24 | Cytoplasm | other |
| MIMAT0003285 | miR-548c-3p (miRNAs w/seed AAAAAUC) | 3.760 | TargetScan Human | Moderate (predicted) | RPL5 | ribosomal protein L5 | Cytoplasm | other |
| MIMAT0003285 | miR-548c-3p (miRNAs w/seed AAAAAUC) | 3.760 | TargetScan Human | Moderate (predicted) | RWDD3 | RWD domain containing 3 | Other | other |
| MIMAT0003285 | miR-548c-3p (miRNAs w/seed AAAAAUC) | 3.760 | TargetScan Human | Moderate (predicted) | RXYLT1 | ribitol xylosyltransferase 1 | Plasma Membrane | enzyme |
| MIMAT0003285 | miR-548c-3p (miRNAs w/seed AAAAAUC) | 3.760 | TargetScan Human | Moderate (predicted) | SCAF11 | SR-related CTD associated factor 11 | Nucleus | other |
| MIMAT0003285 | miR-548c-3p (miRNAs w/seed AAAAAUC) | 3.760 | TargetScan Human | High (predicted) | SI | sucrase-isomaltase | Cytoplasm | enzyme |
| MIMAT0003285 | miR-548c-3p (miRNAs w/seed AAAAAUC) | 3.760 | TargetScan Human | Moderate (predicted) | SLC39A12 | solute carrier family 39 member 12 | Plasma Membrane | transporter |
| MIMAT0003285 | miR-548c-3p (miRNAs w/seed AAAAAUC) | 3.760 | TargetScan Human | Moderate (predicted) | SMIM10 | small integral membrane protein 10 | Other | other |
| MIMAT0003285 | miR-548c-3p (miRNAs w/seed AAAAAUC) | 3.760 | TargetScan Human | Moderate (predicted) | SNCA | synuclein alpha | Cytoplasm | enzyme |
| MIMAT0003285 | miR-548c-3p (miRNAs w/seed AAAAAUC) | 3.760 | TargetScan Human | Moderate (predicted) | SPIN4 | spindlin family member 4 | Other | other |
| MIMAT0003285 | miR-548c-3p (miRNAs w/seed AAAAAUC) | 3.760 | TargetScan Human | Moderate (predicted) | STT3A | gosaccharyltransferase complex catalytic s | Plasma Membrane | enzyme |
| MIMAT0003285 | miR-548c-3p (miRNAs w/seed AAAAAUC) | 3.760 | TargetScan Human | Moderate (predicted) | TEX36 | testis expressed 36 | Other | other |
| MIMAT0003285 | miR-548c-3p (miRNAs w/seed AAAAAUC) | 3.760 | TargetScan Human | Moderate (predicted) | TMEM138 | transmembrane protein 138 | Extracellular Space | other |
| MIMAT0003285 | miR-548c-3p (miRNAs w/seed AAAAAUC) | 3.760 | TargetScan Human | Moderate (predicted) | TTC27 | tetratricopeptide repeat domain 27 | Other | other |
| MIMAT0003285 | miR-548c-3p (miRNAs w/seed AAAAAUC) | 3.760 | TargetScan Human | Moderate (predicted) | UBD | ubiquitin D | Nucleus | other |
| MIMAT0003285 | miR-548c-3p (miRNAs w/seed AAAAAUC) | 3.760 | TargetScan Human | Moderate (predicted) | UBN2 | ubinuclein 2 | Nucleus | other |
| MIMAT0003285 | miR-548c-3p (miRNAs w/seed AAAAAUC) | 3.760 | TargetScan Human | Moderate (predicted) | UFM1 | ubiquitin fold modifier 1 | Cytoplasm | other |
| MIMAT0003285 | miR-548c-3p (miRNAs w/seed AAAAAUC) | 3.760 | TargetScan Human | Moderate (predicted) | UMOD | uromodulin | Extracellular Space | other |
| MIMAT0003285 | miR-548c-3p (miRNAs w/seed AAAAAUC) | 3.760 | TargetScan Human | Moderate (predicted) | WTAP | WT1 associated protein | Nucleus | other |
| MIMAT0003285 | miR-548c-3p (miRNAs w/seed AAAAAUC) | 3.760 | TargetScan Human | Moderate (predicted) | XKR4 | XK related 4 | Plasma Membrane | other |
| MIMAT0003285 | miR-548c-3p (miRNAs w/seed AAAAAUC) | 3.760 | TargetScan Human | Moderate (predicted) | YBX1 | Y-box binding protein 1 | Nucleus | transcription regulator |
| MIMAT0003285 | miR-548c-3p (miRNAs w/seed AAAAAUC) | 3.760 | TargetScan Human | Moderate (predicted) | ZBTB37 | zinc finger and BTB domain containing 37 | Nucleus | transcription regulator |
| MIMAT0003285 | miR-548c-3p (miRNAs w/seed AAAAAUC) | 3.760 | TargetScan Human | Moderate (predicted) | ZC2HC1B | zinc finger C2HC-type containing 1B | Other | other |
| MIMAT0003285 | miR-548c-3p (miRNAs w/seed AAAAAUC) | 3.760 | TargetScan Human | Moderate (predicted) | ZNF264 | zinc finger protein 264 | Nucleus | transcription regulator |
| MIMAT0003285 | miR-548c-3p (miRNAs w/seed AAAAAUC) | 3.760 | TargetScan Human | Moderate (predicted) | ZNF460 | zinc finger protein 460 | Nucleus | transcription regulator |
| MIMAT0003285 | miR-548c-3p (miRNAs w/seed AAAAAUC) | 3.760 | TargetScan Human | Moderate (predicted) | ZNF506 | zinc finger protein 506 | Nucleus | transcription regulator |
| MIMAT0000245 | miR-30c-5p (and other miRNAs w/seed GUAAACA) | 3.150 | TargetScan Human | High (predicted) | ABHD10 | ydrolase domain containing 10, depalmito | Cytoplasm | enzyme |
| MIMAT0000245 | miR-30c-5p (and other miRNAs w/seed GUAAACA) | 3.150 | TargetScan Human | Moderate (predicted) | ABHD6 | drolase domain containing 6, acylglycerol | Cytoplasm | enzyme |
| MIMAT0000245 | miR-30c-5p (and other miRNAs w/seed GUAAACA) | 3.150 | TargetScan Human | Moderate (predicted) | ABI3BP | ABI family member 3 binding protein | Extracellular Space | other |

Sup. Table 3. List of 3699 targets of the seven dysregulated miRNAs obtained from three different target prediction databases,TargetScan TarBase and miRecords considering those predicted as moderate, high, or experimentally validated, respectively.

| | | | | | | | | |
|---|---|---|---|---|---|---|---|---|
| MIMAT0000245 | miR-30c-5p (and other miRNAs w/seed GUAAACA) | 3.150 | TargetScan Human | Moderate (predicted) | ABL1 | proto-oncogene 1, non-receptor tyrosine k | Nucleus | kinase |
| MIMAT0000245 | miR-30c-5p (and other miRNAs w/seed GUAAACA) | 3.150 | TargetScan Human | Moderate (predicted) | ACSBG1 | -CoA synthetase bubblegum family memb | Cytoplasm | enzyme |
| MIMAT0000245 | miR-30c-5p (and other miRNAs w/seed GUAAACA) | 3.150 | TargetScan Human | Moderate (predicted) | ACTBL2 | actin beta like 2 | Nucleus | other |
| MIMAT0000245 | miR-30c-5p (and other miRNAs w/seed GUAAACA) | 3.150 | TargetScan Human | High (predicted) | ACTC1 | actin alpha cardiac muscle 1 | Cytoplasm | enzyme |
| MIMAT0000245 | miR-30c-5p (and other miRNAs w/seed GUAAACA) | 3.150 | TargetScan Human | Moderate (predicted) | ACTN1 | actinin alpha 1 | Cytoplasm | transcription regulator |
| MIMAT0000245 | miR-30c-5p (and other miRNAs w/seed GUAAACA) | 3.150 | TargetScan Human | Moderate (predicted) | ACTR10 | actin related protein 10 | Cytoplasm | other |
| MIMAT0000245 | miR-30c-5p (and other miRNAs w/seed GUAAACA) | 3.150 | TargetScan Human | High (predicted) | ACTR3C | actin related protein 3C | Cytoplasm | other |
| MIMAT0000245 | miR-30c-5p (and other miRNAs w/seed GUAAACA) | 3.150 | rgetScan Human,miRec | ntally Observed,Moderate ( | ACVR1 | activin A receptor type 1 | Plasma Membrane | kinase |
| MIMAT0000245 | miR-30c-5p (and other miRNAs w/seed GUAAACA) | 3.150 | TargetScan Human | Moderate (predicted) | ADAM12 | ADAM metallopeptidase domain 12 | Plasma Membrane | peptidase |
| MIMAT0000245 | miR-30c-5p (and other miRNAs w/seed GUAAACA) | 3.150 | TargetScan Human | Moderate (predicted) | ADAM19 | ADAM metallopeptidase domain 19 | Plasma Membrane | peptidase |
| MIMAT0000245 | miR-30c-5p (and other miRNAs w/seed GUAAACA) | 3.150 | TargetScan Human | Moderate (predicted) | ADAM22 | ADAM metallopeptidase domain 22 | Plasma Membrane | peptidase |
| MIMAT0000245 | miR-30c-5p (and other miRNAs w/seed GUAAACA) | 3.150 | TargetScan Human | Moderate (predicted) | ADAM28 | ADAM metallopeptidase domain 28 | Plasma Membrane | peptidase |
| MIMAT0000245 | miR-30c-5p (and other miRNAs w/seed GUAAACA) | 3.150 | TargetScan Human | Moderate (predicted) | ADAM9 | ADAM metallopeptidase domain 9 | Plasma Membrane | peptidase |
| MIMAT0000245 | miR-30c-5p (and other miRNAs w/seed GUAAACA) | 3.150 | ngenuity Expert Finding | Experimentally Observed | ADAMTS14 | etallopeptidase with thrombospondin type | Extracellular Space | peptidase |
| MIMAT0000245 | miR-30c-5p (and other miRNAs w/seed GUAAACA) | 3.150 | ngenuity Expert Finding | Experimentally Observed | ADAMTS15 | etallopeptidase with thrombospondin type | Extracellular Space | peptidase |
| MIMAT0000245 | miR-30c-5p (and other miRNAs w/seed GUAAACA) | 3.150 | ngenuity Expert Finding | Experimentally Observed | ADAMTS2 | etallopeptidase with thrombospondin type | Extracellular Space | peptidase |
| MIMAT0000245 | miR-30c-5p (and other miRNAs w/seed GUAAACA) | 3.150 | TargetScan Human | Moderate (predicted) | ADAMTS3 | etallopeptidase with thrombospondin type | Extracellular Space | peptidase |
| MIMAT0000245 | miR-30c-5p (and other miRNAs w/seed GUAAACA) | 3.150 | TargetScan Human | Moderate (predicted) | ADGRA3 | adhesion G protein-coupled receptor A3 | Plasma Membrane | G-protein coupled receptor |
| MIMAT0000245 | miR-30c-5p (and other miRNAs w/seed GUAAACA) | 3.150 | TargetScan Human | Moderate (predicted) | ADO | 2-aminoethanethiol dioxygenase | Cytoplasm | enzyme |
| MIMAT0000245 | miR-30c-5p (and other miRNAs w/seed GUAAACA) | 3.150 | TarBase | Experimentally Observed | ADPGK | ADP dependent glucokinase | Plasma Membrane | kinase |
| MIMAT0000245 | miR-30c-5p (and other miRNAs w/seed GUAAACA) | 3.150 | TargetScan Human | Moderate (predicted) | ADRA1D | adrenoceptor alpha 1D | Plasma Membrane | G-protein coupled receptor |
| MIMAT0000245 | miR-30c-5p (and other miRNAs w/seed GUAAACA) | 3.150 | TargetScan Human | High (predicted) | ADRA2A | adrenoceptor alpha 2A | Plasma Membrane | G-protein coupled receptor |
| MIMAT0000245 | miR-30c-5p (and other miRNAs w/seed GUAAACA) | 3.150 | TargetScan Human | Moderate (predicted) | ADRB2 | adrenoceptor beta 2 | Plasma Membrane | G-protein coupled receptor |
| MIMAT0000245 | miR-30c-5p (and other miRNAs w/seed GUAAACA) | 3.150 | TargetScan Human | Moderate (predicted) | AIDA | axin interactor, dorsalization associated | Cytoplasm | other |
| MIMAT0000245 | miR-30c-5p (and other miRNAs w/seed GUAAACA) | 3.150 | TargetScan Human | Moderate (predicted) | AK4 | adenylate kinase 4 | Cytoplasm | kinase |
| MIMAT0000245 | miR-30c-5p (and other miRNAs w/seed GUAAACA) | 3.150 | TargetScan Human | Moderate (predicted) | AKAP10 | A-kinase anchoring protein 10 | Cytoplasm | other |
| MIMAT0000245 | miR-30c-5p (and other miRNAs w/seed GUAAACA) | 3.150 | TargetScan Human | High (predicted) | ALDH2 | aldehyde dehydrogenase 2 family membe | Cytoplasm | enzyme |
| MIMAT0000245 | miR-30c-5p (and other miRNAs w/seed GUAAACA) | 3.150 | TargetScan Human | Moderate (predicted) | ALG10 | ALG10 alpha-1,2-glucosyltransferase | Cytoplasm | enzyme |
| MIMAT0000245 | miR-30c-5p (and other miRNAs w/seed GUAAACA) | 3.150 | TargetScan Human | Moderate (predicted) | ALG10B | ALG10 alpha-1,2-glucosyltransferase B | Plasma Membrane | transporter |
| MIMAT0000245 | miR-30c-5p (and other miRNAs w/seed GUAAACA) | 3.150 | TargetScan Human | Moderate (predicted) | AMOTL2 | angiomotin like 2 | Plasma Membrane | other |
| MIMAT0000245 | miR-30c-5p (and other miRNAs w/seed GUAAACA) | 3.150 | TargetScan Human | High (predicted) | HD1/ANKHD1-EIF4E | nkyrin repeat and KH domain containing | Cytoplasm | other |
| MIMAT0000245 | miR-30c-5p (and other miRNAs w/seed GUAAACA) | 3.150 | TargetScan Human | High (predicted) | ANKRA2 | ankyrin repeat family A member 2 | Cytoplasm | transcription regulator |
| MIMAT0000245 | miR-30c-5p (and other miRNAs w/seed GUAAACA) | 3.150 | TargetScan Human | Moderate (predicted) | ANKRD17 | ankyrin repeat domain 17 | Nucleus | other |
| MIMAT0000245 | miR-30c-5p (and other miRNAs w/seed GUAAACA) | 3.150 | TargetScan Human | Moderate (predicted) | RD20A4P (includes o | repeat domain 20 family member A3, pse | Plasma Membrane | other |
| MIMAT0000245 | miR-30c-5p (and other miRNAs w/seed GUAAACA) | 3.150 | TargetScan Human | Moderate (predicted) | ANKRD22 | ankyrin repeat domain 22 | Nucleus | transcription regulator |
| MIMAT0000245 | miR-30c-5p (and other miRNAs w/seed GUAAACA) | 3.150 | TargetScan Human | Moderate (predicted) | ANKS4B | epeat and sterile alpha motif domain conta | Nucleus | transcription regulator |
| MIMAT0000245 | miR-30c-5p (and other miRNAs w/seed GUAAACA) | 3.150 | TargetScan Human | Moderate (predicted) | ANO4 | anoctamin 4 | Plasma Membrane | ion channel |
| MIMAT0000245 | miR-30c-5p (and other miRNAs w/seed GUAAACA) | 3.150 | TarBase | Experimentally Observed | ANPEP | alanyl aminopeptidase, membrane | Plasma Membrane | peptidase |
| MIMAT0000245 | miR-30c-5p (and other miRNAs w/seed GUAAACA) | 3.150 | arBase,TargetScan Hum | ntally Observed,Moderate ( | AP2A1 | ptor related protein complex 2 subunit alp | Cytoplasm | transporter |
| MIMAT0000245 | miR-30c-5p (and other miRNAs w/seed GUAAACA) | 3.150 | TargetScan Human | Moderate (predicted) | AP3S1 | ptor related protein complex 3 subunit sig | Cytoplasm | transporter |
| MIMAT0000245 | miR-30c-5p (and other miRNAs w/seed GUAAACA) | 3.150 | TargetScan Human | Moderate (predicted) | AP4E1 | ptor related protein complex 4 subunit epsi | Cytoplasm | other |
| MIMAT0000245 | miR-30c-5p (and other miRNAs w/seed GUAAACA) | 3.150 | TargetScan Human | Moderate (predicted) | ARF4 | ADP ribosylation factor 4 | Cytoplasm | enzyme |
| MIMAT0000245 | miR-30c-5p (and other miRNAs w/seed GUAAACA) | 3.150 | TargetScan Human | Moderate (predicted) | ARID4A | AT-rich interaction domain 4A | Nucleus | transcription regulator |
| MIMAT0000245 | miR-30c-5p (and other miRNAs w/seed GUAAACA) | 3.150 | TargetScan Human | Moderate (predicted) | ARL10 | ADP ribosylation factor like GTPase 10 | Other | other |
| MIMAT0000245 | miR-30c-5p (and other miRNAs w/seed GUAAACA) | 3.150 | TargetScan Human | Moderate (predicted) | ARL15 | ADP ribosylation factor like GTPase 15 | Cytoplasm | other |
| MIMAT0000245 | miR-30c-5p (and other miRNAs w/seed GUAAACA) | 3.150 | TargetScan Human | High (predicted) | ARL4A | ADP ribosylation factor like GTPase 4A | Nucleus | enzyme |
| MIMAT0000245 | miR-30c-5p (and other miRNAs w/seed GUAAACA) | 3.150 | TargetScan Human | Moderate (predicted) | ARL4C | ADP ribosylation factor like GTPase 4C | Nucleus | enzyme |
| MIMAT0000245 | miR-30c-5p (and other miRNAs w/seed GUAAACA) | 3.150 | TargetScan Human | Moderate (predicted) | ARL6IP6 | osylation factor like GTPase 6 interacting | Nucleus | other |
| MIMAT0000245 | miR-30c-5p (and other miRNAs w/seed GUAAACA) | 3.150 | TargetScan Human | Moderate (predicted) | ARPC5 | actin related protein 2/3 complex subunit 5 | Cytoplasm | other |
| MIMAT0000245 | miR-30c-5p (and other miRNAs w/seed GUAAACA) | 3.150 | TargetScan Human | Moderate (predicted) | ASB2 | ankyrin repeat and SOCS box containing 2 | Nucleus | transcription regulator |
| MIMAT0000245 | miR-30c-5p (and other miRNAs w/seed GUAAACA) | 3.150 | TargetScan Human | High (predicted) | ASB3/GPR75-ASB3 | ankyrin repeat and SOCS box containing 3 | Cytoplasm | transcription regulator |
| MIMAT0000245 | miR-30c-5p (and other miRNAs w/seed GUAAACA) | 3.150 | TargetScan Human | Moderate (predicted) | ASCL2 | haete-scute family bHLH transcription fact | Nucleus | transcription regulator |
| MIMAT0000245 | miR-30c-5p (and other miRNAs w/seed GUAAACA) | 3.150 | TargetScan Human | Moderate (predicted) | ATAD2B | ATPase family AAA domain containing 2B | Nucleus | other |
| MIMAT0000245 | miR-30c-5p (and other miRNAs w/seed GUAAACA) | 3.150 | TargetScan Human | Moderate (predicted) | ATF1 | activating transcription factor 1 | Nucleus | transcription regulator |
| MIMAT0000245 | miR-30c-5p (and other miRNAs w/seed GUAAACA) | 3.150 | TargetScan Human | High (predicted) | ATG12 | autophagy related 12 | Cytoplasm | other |
| MIMAT0000245 | miR-30c-5p (and other miRNAs w/seed GUAAACA) | 3.150 | TargetScan Human | High (predicted) | ATG5 | autophagy related 5 | Cytoplasm | other |
| MIMAT0000245 | miR-30c-5p (and other miRNAs w/seed GUAAACA) | 3.150 | TargetScan Human | Moderate (predicted) | ATL2 | atlastin GTPase 2 | Cytoplasm | other |
| MIMAT0000245 | miR-30c-5p (and other miRNAs w/seed GUAAACA) | 3.150 | TarBase | Experimentally Observed | ATP2A2 | coplasmic/endoplasmic reticulum Ca2+ tr | Cytoplasm | transporter |
| MIMAT0000245 | miR-30c-5p (and other miRNAs w/seed GUAAACA) | 3.150 | TargetScan Human | High (predicted) | ATP8A1 | ATPase phospholipid transporting 8A1 | Cytoplasm | transporter |
| MIMAT0000245 | miR-30c-5p (and other miRNAs w/seed GUAAACA) | 3.150 | TarBase | Experimentally Observed | ATRX | ATRX chromatin remodeler | Nucleus | transcription regulator |
| MIMAT0000245 | miR-30c-5p (and other miRNAs w/seed GUAAACA) | 3.150 | TargetScan Human | High (predicted) | AVEN | apoptosis and caspase activation inhibitor | Nucleus | other |
| MIMAT0000245 | miR-30c-5p (and other miRNAs w/seed GUAAACA) | 3.150 | TargetScan Human | Moderate (predicted) | AZIN1 | antizyme inhibitor 1 | Cytoplasm | enzyme |
| MIMAT0000245 | miR-30c-5p (and other miRNAs w/seed GUAAACA) | 3.150 | TargetScan Human | High (predicted) | B3GNT5 | c:betaGal beta-1,3-N-acetylglucosaminylt | Cytoplasm | enzyme |
| MIMAT0000245 | miR-30c-5p (and other miRNAs w/seed GUAAACA) | 3.150 | TargetScan Human | Moderate (predicted) | B4GALT4 | beta-1,4-galactosyltransferase 4 | Cytoplasm | enzyme |
| MIMAT0000245 | miR-30c-5p (and other miRNAs w/seed GUAAACA) | 3.150 | TargetScan Human | Moderate (predicted) | B4GALT6 | beta-1,4-galactosyltransferase 6 | Cytoplasm | enzyme |
| MIMAT0000245 | miR-30c-5p (and other miRNAs w/seed GUAAACA) | 3.150 | TargetScan Human | Moderate (predicted) | BCAP29 | B cell receptor associated protein 29 | Cytoplasm | transporter |
| MIMAT0000245 | miR-30c-5p (and other miRNAs w/seed GUAAACA) | 3.150 | TargetScan Human | Moderate (predicted) | BCHE | butyrylcholinesterase | Plasma Membrane | enzyme |
| MIMAT0000245 | miR-30c-5p (and other miRNAs w/seed GUAAACA) | 3.150 | TargetScan Human | Moderate (predicted) | BCL10 | BCL10 immune signaling adaptor | Cytoplasm | transcription regulator |
| MIMAT0000245 | miR-30c-5p (and other miRNAs w/seed GUAAACA) | 3.150 | TargetScan Human | Moderate (predicted) | BCL11B | chromatin remodeling complex subunit BC | Nucleus | transcription regulator |
| MIMAT0000245 | miR-30c-5p (and other miRNAs w/seed GUAAACA) | 3.150 | TargetScan Human | Moderate (predicted) | BCL2L11 | BCL2 like 11 | Cytoplasm | other |
| MIMAT0000245 | miR-30c-5p (and other miRNAs w/seed GUAAACA) | 3.150 | ngenuity Expert Finding | Experimentally Observed | BCL6 | BCL6 transcription repressor | Nucleus | transcription regulator |
| MIMAT0000245 | miR-30c-5p (and other miRNAs w/seed GUAAACA) | 3.150 | TargetScan Human | Moderate (predicted) | BCOR | BCL6 corepressor | Nucleus | transcription regulator |
| MIMAT0000245 | miR-30c-5p (and other miRNAs w/seed GUAAACA) | 3.150 | TargetScan Human | Moderate (predicted) | BDP1 | subunit of RNA polymerase III transcription | Nucleus | transcription regulator |
| MIMAT0000245 | miR-30c-5p (and other miRNAs w/seed GUAAACA) | 3.150 | rgetScan Human,miRec | entally Observed,High (pr | BECN1 | beclin 1 | Cytoplasm | other |
| MIMAT0000245 | miR-30c-5p (and other miRNAs w/seed GUAAACA) | 3.150 | TargetScan Human | Moderate (predicted) | BEND4 | BEN domain containing 4 | Other | other |
| MIMAT0000245 | miR-30c-5p (and other miRNAs w/seed GUAAACA) | 3.150 | TargetScan Human | Moderate (predicted) | BEND7 | BEN domain containing 7 | Cytoplasm | other |
| MIMAT0000245 | miR-30c-5p (and other miRNAs w/seed GUAAACA) | 3.150 | TargetScan Human | High (predicted) | BEST4 | bestrophin 4 | Plasma Membrane | other |
| MIMAT0000245 | miR-30c-5p (and other miRNAs w/seed GUAAACA) | 3.150 | TargetScan Human | Moderate (predicted) | BEX1 | brain expressed X-linked 1 | Other | other |
| MIMAT0000245 | miR-30c-5p (and other miRNAs w/seed GUAAACA) | 3.150 | TargetScan Human | Moderate (predicted) | BLOC1S6 | esis of lysosomal organelles complex 1 su | Cytoplasm | other |
| MIMAT0000245 | miR-30c-5p (and other miRNAs w/seed GUAAACA) | 3.150 | TargetScan Human | Moderate (predicted) | BNC1 | basonuclin 1 | Nucleus | transcription regulator |
| MIMAT0000245 | miR-30c-5p (and other miRNAs w/seed GUAAACA) | 3.150 | TargetScan Human | Moderate (predicted) | BRWD3 | nodomain and WD repeat domain contain | Other | other |
| MIMAT0000245 | miR-30c-5p (and other miRNAs w/seed GUAAACA) | 3.150 | TargetScan Human | Moderate (predicted) | BTBD10 | BTB domain containing 10 | Cytoplasm | other |
| MIMAT0000245 | miR-30c-5p (and other miRNAs w/seed GUAAACA) | 3.150 | TargetScan Human | Moderate (predicted) | BTBD7 | BTB domain containing 7 | Nucleus | other |
| MIMAT0000245 | miR-30c-5p (and other miRNAs w/seed GUAAACA) | 3.150 | TargetScan Human | Moderate (predicted) | C11orf21 | chromosome 11 open reading frame 21 | Cytoplasm | other |
| MIMAT0000245 | miR-30c-5p (and other miRNAs w/seed GUAAACA) | 3.150 | TargetScan Human | Moderate (predicted) | C14orf39 | chromosome 14 open reading frame 39 | Extracellular Space | other |
| MIMAT0000245 | miR-30c-5p (and other miRNAs w/seed GUAAACA) | 3.150 | TargetScan Human | Moderate (predicted) | C16orf87 | chromosome 16 open reading frame 87 | Other | other |
| MIMAT0000245 | miR-30c-5p (and other miRNAs w/seed GUAAACA) | 3.150 | TargetScan Human | Moderate (predicted) | C19orf12 | chromosome 19 open reading frame 12 | Cytoplasm | other |
| MIMAT0000245 | miR-30c-5p (and other miRNAs w/seed GUAAACA) | 3.150 | TargetScan Human | Moderate (predicted) | C1orf131 | chromosome 1 open reading frame 131 | Nucleus | other |
| MIMAT0000245 | miR-30c-5p (and other miRNAs w/seed GUAAACA) | 3.150 | TargetScan Human | Moderate (predicted) | C1orf174 | chromosome 1 open reading frame 174 | Nucleus | other |
| MIMAT0000245 | miR-30c-5p (and other miRNAs w/seed GUAAACA) | 3.150 | TargetScan Human | Moderate (predicted) | C2CD6 | C2 calcium dependent domain containing | Other | other |

Sup. Table 3. List of 3699 targets of the seven dysregulated miRNAs obtained from three different target prediction databases,TargetScan TarBase and miRecords considering those predicted as moderate, high, or experimentally validated, respectively.

| | | | | | | | | |
|---|---|---|---|---|---|---|---|---|
| MIMAT0000245 | miR-30c-5p (and other miRNAs w/seed GUAAACA) | 3.150 | TargetScan Human | Moderate (predicted) | C3orf38 | chromosome 3 open reading frame 38 | Nucleus | peptidase |
| MIMAT0000245 | miR-30c-5p (and other miRNAs w/seed GUAAACA) | 3.150 | TargetScan Human | Moderate (predicted) | C3orf56 | chromosome 3 open reading frame 56 | Other | other |
| MIMAT0000245 | miR-30c-5p (and other miRNAs w/seed GUAAACA) | 3.150 | TargetScan Human | Moderate (predicted) | C4orf19 | chromosome 4 open reading frame 19 | Nucleus | other |
| MIMAT0000245 | miR-30c-5p (and other miRNAs w/seed GUAAACA) | 3.150 | TargetScan Human | Moderate (predicted) | C4orf3 | chromosome 4 open reading frame 3 | Other | other |
| MIMAT0000245 | miR-30c-5p (and other miRNAs w/seed GUAAACA) | 3.150 | TargetScan Human | Moderate (predicted) | C6orf118 | chromosome 6 open reading frame 118 | Other | other |
| MIMAT0000245 | miR-30c-5p (and other miRNAs w/seed GUAAACA) | 3.150 | TargetScan Human | Moderate (predicted) | C6orf141 | chromosome 6 open reading frame 141 | Other | other |
| MIMAT0000245 | miR-30c-5p (and other miRNAs w/seed GUAAACA) | 3.150 | TargetScan Human | Moderate (predicted) | C7orf31 | chromosome 7 open reading frame 31 | Cytoplasm | transporter |
| MIMAT0000245 | miR-30c-5p (and other miRNAs w/seed GUAAACA) | 3.150 | TargetScan Human | Moderate (predicted) | C8orf44-SGK3/SGK3 | /glucocorticoid regulated kinase family me | Cytoplasm | kinase |
| MIMAT0000245 | miR-30c-5p (and other miRNAs w/seed GUAAACA) | 3.150 | TargetScan Human | Moderate (predicted) | C8orf76 | chromosome 8 open reading frame 76 | Other | other |
| MIMAT0000245 | miR-30c-5p (and other miRNAs w/seed GUAAACA) | 3.150 | TargetScan Human | High (predicted) | C9orf72 | C9orf72-SMCR8 complex subunit | Cytoplasm | other |
| MIMAT0000245 | miR-30c-5p (and other miRNAs w/seed GUAAACA) | 3.150 | TargetScan Human | Moderate (predicted) | CACHD1 | cache domain containing 1 | Other | other |
| MIMAT0000245 | miR-30c-5p (and other miRNAs w/seed GUAAACA) | 3.150 | TargetScan Human | Moderate (predicted) | CACNB2 | m voltage-gated channel auxiliary subunit | Plasma Membrane | ion channel |
| MIMAT0000245 | miR-30c-5p (and other miRNAs w/seed GUAAACA) | 3.150 | TargetScan Human | High (predicted) | CALB2 | calbindin 2 | Cytoplasm | other |
| MIMAT0000245 | miR-30c-5p (and other miRNAs w/seed GUAAACA) | 3.150 | TargetScan Human | Moderate (predicted) | CALCR | calcitonin receptor | Plasma Membrane | G-protein coupled receptor |
| MIMAT0000245 | miR-30c-5p (and other miRNAs w/seed GUAAACA) | 3.150 | TargetScan Human | Moderate (predicted) | CALHM4 | cium homeostasis modulator family memb | Other | other |
| MIMAT0000245 | miR-30c-5p (and other miRNAs w/seed GUAAACA) | 3.150 | TargetScan Human | Moderate (predicted) | CALU | calumenin | Cytoplasm | other |
| MIMAT0000245 | miR-30c-5p (and other miRNAs w/seed GUAAACA) | 3.150 | TargetScan Human | Moderate (predicted) | CAMK2D | um/calmodulin dependent protein kinase II | Cytoplasm | kinase |
| MIMAT0000245 | miR-30c-5p (and other miRNAs w/seed GUAAACA) | 3.150 | TargetScan Human | High (predicted) | CAMK2N1 | /calmodulin dependent protein kinase II in | Plasma Membrane | kinase |
| MIMAT0000245 | miR-30c-5p (and other miRNAs w/seed GUAAACA) | 3.150 | TargetScan Human | Moderate (predicted) | CAMK2N2 | /calmodulin dependent protein kinase II in | Nucleus | other |
| MIMAT0000245 | miR-30c-5p (and other miRNAs w/seed GUAAACA) | 3.150 | TargetScan Human | Moderate (predicted) | CAMK4 | cium/calmodulin dependent protein kinase | Nucleus | kinase |
| MIMAT0000245 | miR-30c-5p (and other miRNAs w/seed GUAAACA) | 3.150 | TargetScan Human | Moderate (predicted) | CAMLG | calcium modulating ligand | Cytoplasm | other |
| MIMAT0000245 | miR-30c-5p (and other miRNAs w/seed GUAAACA) | 3.150 | TargetScan Human | High (predicted) | CAPN5 | calpain 5 | Cytoplasm | peptidase |
| MIMAT0000245 | miR-30c-5p (and other miRNAs w/seed GUAAACA) | 3.150 | TargetScan Human | Moderate (predicted) | CAPZA1 | ng actin protein of muscle Z-line subunit a | Cytoplasm | other |
| MIMAT0000245 | miR-30c-5p (and other miRNAs w/seed GUAAACA) | 3.150 | TargetScan Human | Moderate (predicted) | CARF | calcium responsive transcription factor | Nucleus | transcription regulator |
| MIMAT0000245 | miR-30c-5p (and other miRNAs w/seed GUAAACA) | 3.150 | TargetScan Human | High (predicted) | CARS1 | cysteinyl-tRNA synthetase 1 | Cytoplasm | enzyme |
| MIMAT0000245 | miR-30c-5p (and other miRNAs w/seed GUAAACA) | 3.150 | TargetScan Human | High (predicted) | CASP3 | caspase 3 | Cytoplasm | peptidase |
| MIMAT0000245 | miR-30c-5p (and other miRNAs w/seed GUAAACA) | 3.150 | TargetScan Human | Moderate (predicted) | CAT | catalase | Cytoplasm | enzyme |
| MIMAT0000245 | miR-30c-5p (and other miRNAs w/seed GUAAACA) | 3.150 | arBase,TargetScan Hum | ntally Observed,Moderate ( | CBFB | core-binding factor subunit beta | Nucleus | transcription regulator |
| MIMAT0000245 | miR-30c-5p (and other miRNAs w/seed GUAAACA) | 3.150 | TargetScan Human | Moderate (predicted) | CBLB | Cbl proto-oncogene B | Nucleus | enzyme |
| MIMAT0000245 | miR-30c-5p (and other miRNAs w/seed GUAAACA) | 3.150 | TargetScan Human | Moderate (predicted) | CBX2 | chromobox 2 | Nucleus | transcription regulator |
| MIMAT0000245 | miR-30c-5p (and other miRNAs w/seed GUAAACA) | 3.150 | TargetScan Human | Moderate (predicted) | CBX3 | chromobox 3 | Nucleus | transcription regulator |
| MIMAT0000245 | miR-30c-5p (and other miRNAs w/seed GUAAACA) | 3.150 | TargetScan Human | High (predicted) | CCDC117 | coiled-coil domain containing 117 | Other | other |
| MIMAT0000245 | miR-30c-5p (and other miRNAs w/seed GUAAACA) | 3.150 | TargetScan Human | Moderate (predicted) | CCDC14 | coiled-coil domain containing 14 | Cytoplasm | other |
| MIMAT0000245 | miR-30c-5p (and other miRNAs w/seed GUAAACA) | 3.150 | TargetScan Human | Moderate (predicted) | CCDC148 | coiled-coil domain containing 148 | Other | other |
| MIMAT0000245 | miR-30c-5p (and other miRNAs w/seed GUAAACA) | 3.150 | TargetScan Human | Moderate (predicted) | CCDC178 | coiled-coil domain containing 178 | Cytoplasm | other |
| MIMAT0000245 | miR-30c-5p (and other miRNAs w/seed GUAAACA) | 3.150 | TargetScan Human | Moderate (predicted) | CCDC18 | coiled-coil domain containing 18 | Other | other |
| MIMAT0000245 | miR-30c-5p (and other miRNAs w/seed GUAAACA) | 3.150 | TargetScan Human | High (predicted) | CCDC38 | coiled-coil domain containing 38 | Cytoplasm | other |
| MIMAT0000245 | miR-30c-5p (and other miRNAs w/seed GUAAACA) | 3.150 | TargetScan Human | Moderate (predicted) | CCDC43 | coiled-coil domain containing 43 | Cytoplasm | other |
| MIMAT0000245 | miR-30c-5p (and other miRNAs w/seed GUAAACA) | 3.150 | TargetScan Human | Moderate (predicted) | CCDC50 | coiled-coil domain containing 50 | Cytoplasm | other |
| MIMAT0000245 | miR-30c-5p (and other miRNAs w/seed GUAAACA) | 3.150 | TargetScan Human | Moderate (predicted) | CCDC71L | coiled-coil domain containing 71 like | Other | other |
| MIMAT0000245 | miR-30c-5p (and other miRNAs w/seed GUAAACA) | 3.150 | TargetScan Human | Moderate (predicted) | CCDC97 | coiled-coil domain containing 97 | Other | other |
| MIMAT0000245 | miR-30c-5p (and other miRNAs w/seed GUAAACA) | 3.150 | TargetScan Human | Moderate (predicted) | CCL16 | C-C motif chemokine ligand 16 | Extracellular Space | cytokine |
| MIMAT0000245 | miR-30c-5p (and other miRNAs w/seed GUAAACA) | 3.150 | ngenuity Expert Finding | Experimentally Observed | CCN2 | cellular communication network factor 2 | Extracellular Space | growth factor |
| MIMAT0000245 | miR-30c-5p (and other miRNAs w/seed GUAAACA) | 3.150 | TargetScan Human | Moderate (predicted) | CCN3 | cellular communication network factor 3 | Extracellular Space | growth factor |
| MIMAT0000245 | miR-30c-5p (and other miRNAs w/seed GUAAACA) | 3.150 | TargetScan Human | High (predicted) | CCNA1 | cyclin A1 | Nucleus | other |
| MIMAT0000245 | miR-30c-5p (and other miRNAs w/seed GUAAACA) | 3.150 | TargetScan Human | High (predicted) | CCNE2 | cyclin E2 | Nucleus | other |
| MIMAT0000245 | miR-30c-5p (and other miRNAs w/seed GUAAACA) | 3.150 | TargetScan Human | Moderate (predicted) | CCNK | cyclin K | Nucleus | kinase |
| MIMAT0000245 | miR-30c-5p (and other miRNAs w/seed GUAAACA) | 3.150 | TargetScan Human | High (predicted) | CCNT2 | cyclin T2 | Nucleus | transcription regulator |
| MIMAT0000245 | miR-30c-5p (and other miRNAs w/seed GUAAACA) | 3.150 | TargetScan Human | Moderate (predicted) | CCNY | cyclin Y | Nucleus | other |
| MIMAT0000245 | miR-30c-5p (and other miRNAs w/seed GUAAACA) | 3.150 | TargetScan Human | Moderate (predicted) | CD80 | CD80 molecule | Plasma Membrane | transmembrane receptor |
| MIMAT0000245 | miR-30c-5p (and other miRNAs w/seed GUAAACA) | 3.150 | TargetScan Human | High (predicted) | CD99 | CD99 molecule (Xg blood group) | Plasma Membrane | other |
| MIMAT0000245 | miR-30c-5p (and other miRNAs w/seed GUAAACA) | 3.150 | TargetScan Human | Moderate (predicted) | CDC37L1 | cell division cycle 37 like 1 | Cytoplasm | other |
| MIMAT0000245 | miR-30c-5p (and other miRNAs w/seed GUAAACA) | 3.150 | TargetScan Human | Moderate (predicted) | CDC7 | cell division cycle 7 | Nucleus | kinase |
| MIMAT0000245 | miR-30c-5p (and other miRNAs w/seed GUAAACA) | 3.150 | TargetScan Human | Moderate (predicted) | CDCA7 | cell division cycle associated 7 | Nucleus | other |
| MIMAT0000245 | miR-30c-5p (and other miRNAs w/seed GUAAACA) | 3.150 | TarBase | Experimentally Observed | CDCP1 | CUB domain containing protein 1 | Plasma Membrane | other |
| MIMAT0000245 | miR-30c-5p (and other miRNAs w/seed GUAAACA) | 3.150 | TargetScan Human | Moderate (predicted) | CDH20 | cadherin 20 | Plasma Membrane | other |
| MIMAT0000245 | miR-30c-5p (and other miRNAs w/seed GUAAACA) | 3.150 | TargetScan Human | Moderate (predicted) | CELF3 | CUGBP Elav-like family member 3 | Nucleus | transcription regulator |
| MIMAT0000245 | miR-30c-5p (and other miRNAs w/seed GUAAACA) | 3.150 | TargetScan Human | Moderate (predicted) | CELSR3 | herin EGF LAG seven-pass G-type recept | Plasma Membrane | G-protein coupled receptor |
| MIMAT0000245 | miR-30c-5p (and other miRNAs w/seed GUAAACA) | 3.150 | TargetScan Human | Moderate (predicted) | CENPO | centromere protein O | Nucleus | other |
| MIMAT0000245 | miR-30c-5p (and other miRNAs w/seed GUAAACA) | 3.150 | TarBase | Experimentally Observed | CEP72 | centrosomal protein 72 | Cytoplasm | other |
| MIMAT0000245 | miR-30c-5p (and other miRNAs w/seed GUAAACA) | 3.150 | TargetScan Human | High (predicted) | CEP76 | centrosomal protein 76 | Cytoplasm | other |
| MIMAT0000245 | miR-30c-5p (and other miRNAs w/seed GUAAACA) | 3.150 | TargetScan Human | Moderate (predicted) | CERT1 | ceramide transporter 1 | Cytoplasm | kinase |
| MIMAT0000245 | miR-30c-5p (and other miRNAs w/seed GUAAACA) | 3.150 | TargetScan Human | High (predicted) | CFDP1 | craniofacial development protein 1 | Extracellular Space | other |
| MIMAT0000245 | miR-30c-5p (and other miRNAs w/seed GUAAACA) | 3.150 | TargetScan Human | Moderate (predicted) | CFL2 | cofilin 2 | Extracellular Space | other |
| MIMAT0000245 | miR-30c-5p (and other miRNAs w/seed GUAAACA) | 3.150 | arBase,TargetScan Hum | nentally Observed,High (pr | CHD1 | romodomain helicase DNA binding protei | Nucleus | enzyme |
| MIMAT0000245 | miR-30c-5p (and other miRNAs w/seed GUAAACA) | 3.150 | TargetScan Human | Moderate (predicted) | CHD7 | romodomain helicase DNA binding protei | Nucleus | enzyme |
| MIMAT0000245 | miR-30c-5p (and other miRNAs w/seed GUAAACA) | 3.150 | TargetScan Human | Moderate (predicted) | CHD9 | romodomain helicase DNA binding protei | Cytoplasm | other |
| MIMAT0000245 | miR-30c-5p (and other miRNAs w/seed GUAAACA) | 3.150 | TargetScan Human | Moderate (predicted) | CHI3L1 | chitinase 3 like 1 | Extracellular Space | enzyme |
| MIMAT0000245 | miR-30c-5p (and other miRNAs w/seed GUAAACA) | 3.150 | TargetScan Human | Moderate (predicted) | CHIC1 | cysteine rich hydrophobic domain 1 | Plasma Membrane | other |
| MIMAT0000245 | miR-30c-5p (and other miRNAs w/seed GUAAACA) | 3.150 | TargetScan Human | Moderate (predicted) | CHL1 | cell adhesion molecule L1 like | Plasma Membrane | other |
| MIMAT0000245 | miR-30c-5p (and other miRNAs w/seed GUAAACA) | 3.150 | TargetScan Human | Moderate (predicted) | CHORDC1 | steine and histidine rich domain containin | Other | other |
| MIMAT0000245 | miR-30c-5p (and other miRNAs w/seed GUAAACA) | 3.150 | TargetScan Human | Moderate (predicted) | CHST1 | carbohydrate sulfotransferase 1 | Cytoplasm | enzyme |
| MIMAT0000245 | miR-30c-5p (and other miRNAs w/seed GUAAACA) | 3.150 | TargetScan Human | Moderate (predicted) | CHST2 | carbohydrate sulfotransferase 2 | Cytoplasm | enzyme |
| MIMAT0000245 | miR-30c-5p (and other miRNAs w/seed GUAAACA) | 3.150 | TargetScan Human | Moderate (predicted) | CHURC1 | churchill domain containing 1 | Nucleus | transcription regulator |
| MIMAT0000245 | miR-30c-5p (and other miRNAs w/seed GUAAACA) | 3.150 | TargetScan Human | Moderate (predicted) | CISD2 | CDGSH iron sulfur domain 2 | Cytoplasm | other |
| MIMAT0000245 | miR-30c-5p (and other miRNAs w/seed GUAAACA) | 3.150 | TargetScan Human | Moderate (predicted) | CLDN12 | claudin 12 | Plasma Membrane | other |
| MIMAT0000245 | miR-30c-5p (and other miRNAs w/seed GUAAACA) | 3.150 | TargetScan Human | Moderate (predicted) | CLN8 | LN8 transmembrane ER and ERGIC prote | Cytoplasm | other |
| MIMAT0000245 | miR-30c-5p (and other miRNAs w/seed GUAAACA) | 3.150 | TargetScan Human | Moderate (predicted) | CLRN1 | clarin 1 | Plasma Membrane | other |
| MIMAT0000245 | miR-30c-5p (and other miRNAs w/seed GUAAACA) | 3.150 | TargetScan Human | Moderate (predicted) | CNKSR2 | nector enhancer of kinase suppressor of R | Plasma Membrane | other |
| MIMAT0000245 | miR-30c-5p (and other miRNAs w/seed GUAAACA) | 3.150 | TarBase | Experimentally Observed | CNOT9 | CCR4-NOT transcription complex subunit | Cytoplasm | transcription regulator |
| MIMAT0000245 | miR-30c-5p (and other miRNAs w/seed GUAAACA) | 3.150 | TargetScan Human | Moderate (predicted) | CNR1 | cannabinoid receptor 1 | Plasma Membrane | G-protein coupled receptor |
| MIMAT0000245 | miR-30c-5p (and other miRNAs w/seed GUAAACA) | 3.150 | TargetScan Human | High (predicted) | CNRIP1 | cannabinoid receptor interacting protein 1 | Extracellular Space | other |
| MIMAT0000245 | miR-30c-5p (and other miRNAs w/seed GUAAACA) | 3.150 | TargetScan Human | Moderate (predicted) | CNTN4 | contactin 4 | Plasma Membrane | enzyme |
| MIMAT0000245 | miR-30c-5p (and other miRNAs w/seed GUAAACA) | 3.150 | TargetScan Human | Moderate (predicted) | COG2 | component of oligomeric golgi complex 2 | Cytoplasm | other |
| MIMAT0000245 | miR-30c-5p (and other miRNAs w/seed GUAAACA) | 3.150 | TargetScan Human | High (predicted) | COL13A1 | collagen type XIII alpha 1 chain | Plasma Membrane | other |
| MIMAT0000245 | miR-30c-5p (and other miRNAs w/seed GUAAACA) | 3.150 | ngenuity Expert Finding | Experimentally Observed | COL1A1 | collagen type I alpha 1 chain | Extracellular Space | other |
| MIMAT0000245 | miR-30c-5p (and other miRNAs w/seed GUAAACA) | 3.150 | ngenuity Expert Finding | Experimentally Observed | COL1A2 | collagen type I alpha 2 chain | Extracellular Space | other |
| MIMAT0000245 | miR-30c-5p (and other miRNAs w/seed GUAAACA) | 3.150 | TargetScan Human | High (predicted) | COL25A1 | collagen type XXV alpha 1 chain | Cytoplasm | other |
| MIMAT0000245 | miR-30c-5p (and other miRNAs w/seed GUAAACA) | 3.150 | ngenuity Expert Finding | Experimentally Observed | COL4A1 | collagen type IV alpha 1 chain | Extracellular Space | other |
| MIMAT0000245 | miR-30c-5p (and other miRNAs w/seed GUAAACA) | 3.150 | ngenuity Expert Finding | Experimentally Observed | COL4A2 | collagen type IV alpha 2 chain | Extracellular Space | other |
| MIMAT0000245 | miR-30c-5p (and other miRNAs w/seed GUAAACA) | 3.150 | ngenuity Expert Finding | Experimentally Observed | COL4A5 | collagen type IV alpha 5 chain | Extracellular Space | other |
| MIMAT0000245 | miR-30c-5p (and other miRNAs w/seed GUAAACA) | 3.150 | ngenuity Expert Finding | Experimentally Observed | COL5A1 | collagen type V alpha 1 chain | Extracellular Space | other |
| MIMAT0000245 | miR-30c-5p (and other miRNAs w/seed GUAAACA) | 3.150 | ngenuity Expert Finding | Experimentally Observed | COL5A2 | collagen type V alpha 2 chain | Extracellular Space | other |
| MIMAT0000245 | miR-30c-5p (and other miRNAs w/seed GUAAACA) | 3.150 | Expert Findings,TargetSc | ntally Observed,Moderate ( | COL8A1 | collagen type VIII alpha 1 chain | Extracellular Space | other |
| MIMAT0000245 | miR-30c-5p (and other miRNAs w/seed GUAAACA) | 3.150 | TargetScan Human | Moderate (predicted) | COL9A3 | collagen type IX alpha 3 chain | Extracellular Space | other |
| MIMAT0000245 | miR-30c-5p (and other miRNAs w/seed GUAAACA) | 3.150 | TargetScan Human | Moderate (predicted) | COLCA2 | colorectal cancer associated 2 | Cytoplasm | other |
| MIMAT0000245 | miR-30c-5p (and other miRNAs w/seed GUAAACA) | 3.150 | TargetScan Human | Moderate (predicted) | COTL1 | coactosin like F-actin binding protein 1 | Cytoplasm | other |
| MIMAT0000245 | miR-30c-5p (and other miRNAs w/seed GUAAACA) | 3.150 | TargetScan Human | Moderate (predicted) | CPE | carboxypeptidase E | Cytoplasm | peptidase |
| MIMAT0000245 | miR-30c-5p (and other miRNAs w/seed GUAAACA) | 3.150 | TargetScan Human | Moderate (predicted) | CPEB3 | asmic polyadenylation element binding pr | Cytoplasm | translation regulator |
| MIMAT0000245 | miR-30c-5p (and other miRNAs w/seed GUAAACA) | 3.150 | TargetScan Human | Moderate (predicted) | CPEB4 | asmic polyadenylation element binding pr | Plasma Membrane | other |
| MIMAT0000245 | miR-30c-5p (and other miRNAs w/seed GUAAACA) | 3.150 | TargetScan Human | Moderate (predicted) | CPNE3 | copine 3 | Cytoplasm | kinase |
| MIMAT0000245 | miR-30c-5p (and other miRNAs w/seed GUAAACA) | 3.150 | arBase,TargetScan Hum | ntally Observed,Moderate ( | CPNE8 | copine 8 | Cytoplasm | other |
| MIMAT0000245 | miR-30c-5p (and other miRNAs w/seed GUAAACA) | 3.150 | TargetScan Human | Moderate (predicted) | CPOX | coproporphyrinogen oxidase | Cytoplasm | enzyme |
| MIMAT0000245 | miR-30c-5p (and other miRNAs w/seed GUAAACA) | 3.150 | TargetScan Human | Moderate (predicted) | CREG1 | ellular repressor of E1A stimulated genes | Nucleus | transcription regulator |
| MIMAT0000245 | miR-30c-5p (and other miRNAs w/seed GUAAACA) | 3.150 | TargetScan Human | Moderate (predicted) | CRK | CRK proto-oncogene, adaptor protein | Cytoplasm | other |
| MIMAT0000245 | miR-30c-5p (and other miRNAs w/seed GUAAACA) | 3.150 | ngenuity Expert Finding | Experimentally Observed | CRTAP | cartilage associated protein | Extracellular Space | other |
| MIMAT0000245 | miR-30c-5p (and other miRNAs w/seed GUAAACA) | 3.150 | TargetScan Human | High (predicted) | CSAD | cysteine sulfinic acid decarboxylase | Cytoplasm | enzyme |
| MIMAT0000245 | miR-30c-5p (and other miRNAs w/seed GUAAACA) | 3.150 | TargetScan Human | Moderate (predicted) | CSN1S1 | casein alpha s1 | Extracellular Space | other |
| MIMAT0000245 | miR-30c-5p (and other miRNAs w/seed GUAAACA) | 3.150 | TargetScan Human | Moderate (predicted) | CSNK1A1 | casein kinase 1 alpha 1 | Cytoplasm | kinase |
| MIMAT0000245 | miR-30c-5p (and other miRNAs w/seed GUAAACA) | 3.150 | TargetScan Human | Moderate (predicted) | CT83 | cancer/testis antigen 83 | Nucleus | other |
| MIMAT0000245 | miR-30c-5p (and other miRNAs w/seed GUAAACA) | 3.150 | TargetScan Human | Moderate (predicted) | CTD_2510F56 | | Other | other |
| MIMAT0000245 | miR-30c-5p (and other miRNAs w/seed GUAAACA) | 3.150 | TargetScan Human | Moderate (predicted) | CTHRC1 | collagen triple helix repeat containing 1 | Extracellular Space | other |
| MIMAT0000245 | miR-30c-5p (and other miRNAs w/seed GUAAACA) | 3.150 | TargetScan Human | Moderate (predicted) | CTNND2 | catenin delta 2 | Plasma Membrane | other |
| MIMAT0000245 | miR-30c-5p (and other miRNAs w/seed GUAAACA) | 3.150 | TargetScan Human | Moderate (predicted) | CTRL | chymotrypsin like | Extracellular Space | peptidase |
| MIMAT0000245 | miR-30c-5p (and other miRNAs w/seed GUAAACA) | 3.150 | TargetScan Human | Moderate (predicted) | CUL2 | cullin 2 | Nucleus | enzyme |
| MIMAT0000245 | miR-30c-5p (and other miRNAs w/seed GUAAACA) | 3.150 | TargetScan Human | Moderate (predicted) | CXCL11 | C-X-C motif chemokine ligand 11 | Extracellular Space | cytokine |
| MIMAT0000245 | miR-30c-5p (and other miRNAs w/seed GUAAACA) | 3.150 | TargetScan Human | Moderate (predicted) | CXXC4 | CXXC finger protein 4 | Cytoplasm | other |
| MIMAT0000245 | miR-30c-5p (and other miRNAs w/seed GUAAACA) | 3.150 | TargetScan Human | Moderate (predicted) | CYB561 | cytochrome b561 | Cytoplasm | enzyme |
| MIMAT0000245 | miR-30c-5p (and other miRNAs w/seed GUAAACA) | 3.150 | TargetScan Human | Moderate (predicted) | CYCS | cytochrome c, somatic | Cytoplasm | enzyme |
| MIMAT0000245 | miR-30c-5p (and other miRNAs w/seed GUAAACA) | 3.150 | TargetScan Human | High (predicted) | CYP24A1 | chrome P450 family 24 subfamily A meml | Cytoplasm | enzyme |
| MIMAT0000245 | miR-30c-5p (and other miRNAs w/seed GUAAACA) | 3.150 | TargetScan Human | High (predicted) | CYRIA | CYFIP related Rac1 interactor A | Other | other |
| MIMAT0000245 | miR-30c-5p (and other miRNAs w/seed GUAAACA) | 3.150 | TargetScan Human | Moderate (predicted) | CYSLTR1 | cysteinyl leukotriene receptor 1 | Plasma Membrane | G-protein coupled receptor |
| MIMAT0000245 | miR-30c-5p (and other miRNAs w/seed GUAAACA) | 3.150 | TargetScan Human | Moderate (predicted) | CYYR1 | cysteine and tyrosine rich 1 | Other | other |
| MIMAT0000245 | miR-30c-5p (and other miRNAs w/seed GUAAACA) | 3.150 | TargetScan Human | High (predicted) | DBF4 | DBF4 zinc finger | Nucleus | kinase |
| MIMAT0000245 | miR-30c-5p (and other miRNAs w/seed GUAAACA) | 3.150 | TargetScan Human | High (predicted) | DCBLD1 | scoidin, CUB and LCCL domain containin | Extracellular Space | other |
| MIMAT0000245 | miR-30c-5p (and other miRNAs w/seed GUAAACA) | 3.150 | TargetScan Human | Moderate (predicted) | DCUN1D1 | ctive in cullin neddylation 1 domain contair | Nucleus | other |
| MIMAT0000245 | miR-30c-5p (and other miRNAs w/seed GUAAACA) | 3.150 | TargetScan Human | Moderate (predicted) | DCUN1D3 | ctive in cullin neddylation 1 domain contair | Cytoplasm | other |
| MIMAT0000245 | miR-30c-5p (and other miRNAs w/seed GUAAACA) | 3.150 | TargetScan Human | Moderate (predicted) | DDAH1 | dimethylarginine dimethylaminohydrolase | Cytoplasm | enzyme |
| MIMAT0000245 | miR-30c-5p (and other miRNAs w/seed GUAAACA) | 3.150 | TargetScan Human | Moderate (predicted) | DDHD2 | DDHD domain containing 2 | Cytoplasm | enzyme |
| MIMAT0000245 | miR-30c-5p (and other miRNAs w/seed GUAAACA) | 3.150 | TargetScan Human | Moderate (predicted) | DDIT4 | DNA damage inducible transcript 4 | Cytoplasm | other |
| MIMAT0000245 | miR-30c-5p (and other miRNAs w/seed GUAAACA) | 3.150 | TargetScan Human | Moderate (predicted) | DENND2C | DENN domain containing 2C | Nucleus | other |
| MIMAT0000245 | miR-30c-5p (and other miRNAs w/seed GUAAACA) | 3.150 | TargetScan Human | High (predicted) | DEPDC4 | DEP domain containing 4 | Other | other |
| MIMAT0000245 | miR-30c-5p (and other miRNAs w/seed GUAAACA) | 3.150 | TargetScan Human | High (predicted) | DESI2 | desumoylating isopeptidase 2 | Cytoplasm | enzyme |
| MIMAT0000245 | miR-30c-5p (and other miRNAs w/seed GUAAACA) | 3.150 | TargetScan Human | Moderate (predicted) | DEXI | Dexi homolog | Other | other |
| MIMAT0000245 | miR-30c-5p (and other miRNAs w/seed GUAAACA) | 3.150 | TargetScan Human | Moderate (predicted) | DGKE | diacylglycerol kinase epsilon | Cytoplasm | kinase |
| MIMAT0000245 | miR-30c-5p (and other miRNAs w/seed GUAAACA) | 3.150 | TargetScan Human | Moderate (predicted) | DGKH | diacylglycerol kinase eta | Cytoplasm | kinase |
| MIMAT0000245 | miR-30c-5p (and other miRNAs w/seed GUAAACA) | 3.150 | TargetScan Human | Moderate (predicted) | DGKZ | diacylglycerol kinase zeta | Cytoplasm | kinase |
| MIMAT0000245 | miR-30c-5p (and other miRNAs w/seed GUAAACA) | 3.150 | TargetScan Human | Moderate (predicted) | DHX36 | DEAH-box helicase 36 | Cytoplasm | enzyme |
| MIMAT0000245 | miR-30c-5p (and other miRNAs w/seed GUAAACA) | 3.150 | TargetScan Human | Moderate (predicted) | DIP2B | disco interacting protein 2 homolog B | Cytoplasm | other |
| MIMAT0000245 | miR-30c-5p (and other miRNAs w/seed GUAAACA) | 3.150 | TargetScan Human | High (predicted) | DLGAP1 | DLG associated protein 1 | Plasma Membrane | other |
| MIMAT0000245 | miR-30c-5p (and other miRNAs w/seed GUAAACA) | 3.150 | TargetScan Human | Moderate (predicted) | DLGAP2 | DLG associated protein 2 | Plasma Membrane | other |
| MIMAT0000245 | miR-30c-5p (and other miRNAs w/seed GUAAACA) | 3.150 | TargetScan Human | Moderate (predicted) | DLL4 | delta like canonical Notch ligand 4 | Extracellular Space | other |
| MIMAT0000245 | miR-30c-5p (and other miRNAs w/seed GUAAACA) | 3.150 | TargetScan Human | Moderate (predicted) | DNAJC13 | heat shock protein family (Hsp40) membe | Cytoplasm | other |
| MIMAT0000245 | miR-30c-5p (and other miRNAs w/seed GUAAACA) | 3.150 | TargetScan Human | Moderate (predicted) | DNAJC25 | heat shock protein family (Hsp40) membe | Other | other |
| MIMAT0000245 | miR-30c-5p (and other miRNAs w/seed GUAAACA) | 3.150 | TargetScan Human | High (predicted) | DNAJC25-GNG10 | DNAJC25-GNG10 readthrough | Other | other |
| MIMAT0000245 | miR-30c-5p (and other miRNAs w/seed GUAAACA) | 3.150 | TargetScan Human | Moderate (predicted) | DNASE2B | deoxyribonuclease 2 beta | Cytoplasm | enzyme |

Sup. Table 3. List of 3699 targets of the seven dysregulated miRNAs obtained from three different target prediction databases,TargetScan TarBase and miRecords considering those predicted as moderate, high, or experimentally validated, respectively.

| | | | | | | | | |
|---|---|---|---|---|---|---|---|---|
| MIMAT0000245 | miR-30c-5p (and other miRNAs w/seed GUAACA) | 3.150 | TargetScan Human | Moderate (predicted) | DNMT3A | DNA methyltransferase 3 alpha | Nucleus | enzyme |
| MIMAT0000245 | miR-30c-5p (and other miRNAs w/seed GUAACA) | 3.150 | TargetScan Human | Moderate (predicted) | DOC2A | double C2 domain alpha | Cytoplasm | transporter |
| MIMAT0000245 | miR-30c-5p (and other miRNAs w/seed GUAACA) | 3.150 | arBase,TargetScan Hum | nentally Observed,High (pr | DOCK7 | dedicator of cytokinesis 7 | Plasma Membrane | other |
| MIMAT0000245 | miR-30c-5p (and other miRNAs w/seed GUAACA) | 3.150 | TargetScan Human | High (predicted) | DOK5 | docking protein 5 | Plasma Membrane | other |
| MIMAT0000245 | miR-30c-5p (and other miRNAs w/seed GUAACA) | 3.150 | TargetScan Human | High (predicted) | DOLPP1 | dolichyldiphosphatase 1 | Cytoplasm | enzyme |
| MIMAT0000245 | miR-30c-5p (and other miRNAs w/seed GUAACA) | 3.150 | TargetScan Human | Moderate (predicted) | DPY19L1 | dpy-19 like C-mannosyltransferase 1 | Other | other |
| MIMAT0000245 | miR-30c-5p (and other miRNAs w/seed GUAACA) | 3.150 | TargetScan Human | Moderate (predicted) | DPYSL2 | dihydropyrimidinase like 2 | Cytoplasm | enzyme |
| MIMAT0000245 | miR-30c-5p (and other miRNAs w/seed GUAACA) | 3.150 | TargetScan Human | Moderate (predicted) | DRD1 | dopamine receptor D1 | Plasma Membrane | G-protein coupled receptor |
| MIMAT0000245 | miR-30c-5p (and other miRNAs w/seed GUAACA) | 3.150 | ngenuity Expert Finding | Experimentally Observed | Dst | dystonin | Plasma Membrane | other |
| MIMAT0000245 | miR-30c-5p (and other miRNAs w/seed GUAACA) | 3.150 | TargetScan Human | Moderate (predicted) | DTD2 | D-aminoacyl-tRNA deacylase 2 | Other | enzyme |
| MIMAT0000245 | miR-30c-5p (and other miRNAs w/seed GUAACA) | 3.150 | TargetScan Human | Moderate (predicted) | DYNLT2 | dynein light chain Tctex-type 2 | Extracellular Space | other |
| MIMAT0000245 | miR-30c-5p (and other miRNAs w/seed GUAACA) | 3.150 | TargetScan Human | Moderate (predicted) | E2F3 | E2F transcription factor 3 | Nucleus | transcription regulator |
| MIMAT0000245 | miR-30c-5p (and other miRNAs w/seed GUAACA) | 3.150 | TargetScan Human | High (predicted) | E2F7 | E2F transcription factor 7 | Nucleus | transcription regulator |
| MIMAT0000245 | miR-30c-5p (and other miRNAs w/seed GUAACA) | 3.150 | TargetScan Human | Moderate (predicted) | EAF1 | ELL associated factor 1 | Nucleus | transcription regulator |
| MIMAT0000245 | miR-30c-5p (and other miRNAs w/seed GUAACA) | 3.150 | TargetScan Human | Moderate (predicted) | EDEM3 | adation enhancing alpha-mannosidase like | Cytoplasm | enzyme |
| MIMAT0000245 | miR-30c-5p (and other miRNAs w/seed GUAACA) | 3.150 | TargetScan Human | Moderate (predicted) | EDIL3 | EGF like repeats and discoidin domains 3 | Extracellular Space | other |
| MIMAT0000245 | miR-30c-5p (and other miRNAs w/seed GUAACA) | 3.150 | TargetScan Human | High (predicted) | EDNRA | endothelin receptor type A | Plasma Membrane | transmembrane receptor |
| MIMAT0000245 | miR-30c-5p (and other miRNAs w/seed GUAACA) | 3.150 | TargetScan Human | Moderate (predicted) | EEA1 | early endosome antigen 1 | Cytoplasm | other |
| MIMAT0000245 | miR-30c-5p (and other miRNAs w/seed GUAACA) | 3.150 | TargetScan Human | High (predicted) | EED | embryonic ectoderm development | Nucleus | transcription regulator |
| MIMAT0000245 | miR-30c-5p (and other miRNAs w/seed GUAACA) | 3.150 | TargetScan Human | Moderate (predicted) | EFR3A | EFR3 homolog A | Plasma Membrane | other |
| MIMAT0000245 | miR-30c-5p (and other miRNAs w/seed GUAACA) | 3.150 | TargetScan Human | Moderate (predicted) | EIF1AY | karyotic translation initiation factor 1A Y-lin | Other | translation regulator |
| MIMAT0000245 | miR-30c-5p (and other miRNAs w/seed GUAACA) | 3.150 | TargetScan Human | High (predicted) | ELAVL2 | ELAV like RNA binding protein 2 | Cytoplasm | other |
| MIMAT0000245 | miR-30c-5p (and other miRNAs w/seed GUAACA) | 3.150 | TargetScan Human | Moderate (predicted) | ELAVL4 | ELAV like RNA binding protein 4 | Cytoplasm | translation regulator |
| MIMAT0000245 | miR-30c-5p (and other miRNAs w/seed GUAACA) | 3.150 | TargetScan Human | Moderate (predicted) | ELL2 | elongation factor for RNA polymerase II 2 | Nucleus | transcription regulator |
| MIMAT0000245 | miR-30c-5p (and other miRNAs w/seed GUAACA) | 3.150 | TargetScan Human | Moderate (predicted) | ELMO1 | engulfment and cell motility 1 | Cytoplasm | other |
| MIMAT0000245 | miR-30c-5p (and other miRNAs w/seed GUAACA) | 3.150 | arBase,TargetScan Hum | nentally Observed,High (pr | ELMOD2 | ELMO domain containing 2 | Other | other |
| MIMAT0000245 | miR-30c-5p (and other miRNAs w/seed GUAACA) | 3.150 | TargetScan Human | Moderate (predicted) | ELOVL2 | ELOVL fatty acid elongase 2 | Cytoplasm | enzyme |
| MIMAT0000245 | miR-30c-5p (and other miRNAs w/seed GUAACA) | 3.150 | TargetScan Human | High (predicted) | ELOVL5 | ELOVL fatty acid elongase 5 | Cytoplasm | enzyme |
| MIMAT0000245 | miR-30c-5p (and other miRNAs w/seed GUAACA) | 3.150 | TargetScan Human | Moderate (predicted) | ELOVL7 | ELOVL fatty acid elongase 7 | Cytoplasm | enzyme |
| MIMAT0000245 | miR-30c-5p (and other miRNAs w/seed GUAACA) | 3.150 | TargetScan Human | Moderate (predicted) | EMCN | endomucin | Extracellular Space | other |
| MIMAT0000245 | miR-30c-5p (and other miRNAs w/seed GUAACA) | 3.150 | TargetScan Human | Moderate (predicted) | EML1 | EMAP like 1 | Cytoplasm | other |
| MIMAT0000245 | miR-30c-5p (and other miRNAs w/seed GUAACA) | 3.150 | TargetScan Human | Moderate (predicted) | ENPP5 | e pyrophosphatase/phosphodiesterase far | Extracellular Space | enzyme |
| MIMAT0000245 | miR-30c-5p (and other miRNAs w/seed GUAACA) | 3.150 | TargetScan Human | Moderate (predicted) | EPB41 | erythrocyte membrane protein band 4.1 | Plasma Membrane | other |
| MIMAT0000245 | miR-30c-5p (and other miRNAs w/seed GUAACA) | 3.150 | TargetScan Human | Moderate (predicted) | EPB41L2 | rythrocyte membrane protein band 4.1 like | Plasma Membrane | other |
| MIMAT0000245 | miR-30c-5p (and other miRNAs w/seed GUAACA) | 3.150 | TargetScan Human | Moderate (predicted) | EPC1 | enhancer of polycomb homolog 1 | Nucleus | transcription regulator |
| MIMAT0000245 | miR-30c-5p (and other miRNAs w/seed GUAACA) | 3.150 | TargetScan Human | Moderate (predicted) | EPC2 | enhancer of polycomb homolog 2 | Other | other |
| MIMAT0000245 | miR-30c-5p (and other miRNAs w/seed GUAACA) | 3.150 | TargetScan Human | Moderate (predicted) | EPDR1 | ependymin related 1 | Nucleus | other |
| MIMAT0000245 | miR-30c-5p (and other miRNAs w/seed GUAACA) | 3.150 | TargetScan Human | Moderate (predicted) | EPG5 | opic P-granules autophagy protein 5 hom | Cytoplasm | other |
| MIMAT0000245 | miR-30c-5p (and other miRNAs w/seed GUAACA) | 3.150 | TargetScan Human | Moderate (predicted) | ERG | ETS transcription factor ERG | Nucleus | transcription regulator |
| MIMAT0000245 | miR-30c-5p (and other miRNAs w/seed GUAACA) | 3.150 | TargetScan Human | Moderate (predicted) | ERICH3 | glutamate rich 3 | Other | other |
| MIMAT0000245 | miR-30c-5p (and other miRNAs w/seed GUAACA) | 3.150 | TargetScan Human | Moderate (predicted) | ERLIN1 | ER lipid raft associated 1 | Plasma Membrane | other |
| MIMAT0000245 | miR-30c-5p (and other miRNAs w/seed GUAACA) | 3.150 | TargetScan Human | Moderate (predicted) | ESCO1 | ent of sister chromatid cohesion N-acetyltr | Nucleus | enzyme |
| MIMAT0000245 | miR-30c-5p (and other miRNAs w/seed GUAACA) | 3.150 | TargetScan Human | Moderate (predicted) | ETFBKMT | nsfer flavoprotein subunit beta lysine meth | Cytoplasm | enzyme |
| MIMAT0000245 | miR-30c-5p (and other miRNAs w/seed GUAACA) | 3.150 | TargetScan Human | High (predicted) | EVX2 | even-skipped homeobox 2 | Nucleus | transcription regulator |
| MIMAT0000245 | miR-30c-5p (and other miRNAs w/seed GUAACA) | 3.150 | TargetScan Human | Moderate (predicted) | EXOC6 | exocyst complex component 6 | Plasma Membrane | transporter |
| MIMAT0000245 | miR-30c-5p (and other miRNAs w/seed GUAACA) | 3.150 | TargetScan Human | High (predicted) | EXTL2 | exostosin like glycosyltransferase 2 | Cytoplasm | enzyme |
| MIMAT0000245 | miR-30c-5p (and other miRNAs w/seed GUAACA) | 3.150 | TargetScan Human | Moderate (predicted) | EYA2 | A transcriptional coactivator and phosphata | Nucleus | phosphatase |
| MIMAT0000245 | miR-30c-5p (and other miRNAs w/seed GUAACA) | 3.150 | TarBase | Experimentally Observed | F2 | coagulation factor II, prothrombin | Extracellular Space | peptidase |
| MIMAT0000245 | miR-30c-5p (and other miRNAs w/seed GUAACA) | 3.150 | TargetScan Human | Moderate (predicted) | FAHD1 | arylacetoacetate hydrolase domain contain | Cytoplasm | enzyme |
| MIMAT0000245 | miR-30c-5p (and other miRNAs w/seed GUAACA) | 3.150 | TargetScan Human | Moderate (predicted) | FAM104A | mily with sequence similarity 104 member | Other | other |
| MIMAT0000245 | miR-30c-5p (and other miRNAs w/seed GUAACA) | 3.150 | TargetScan Human | Moderate (predicted) | FAM110B | mily with sequence similarity 110 member | Cytoplasm | other |
| MIMAT0000245 | miR-30c-5p (and other miRNAs w/seed GUAACA) | 3.150 | TargetScan Human | High (predicted) | FAM133A | mily with sequence similarity 133 member | Other | other |
| MIMAT0000245 | miR-30c-5p (and other miRNAs w/seed GUAACA) | 3.150 | TargetScan Human | Moderate (predicted) | FAM13C | amily with sequence similarity 13 member | Other | other |
| MIMAT0000245 | miR-30c-5p (and other miRNAs w/seed GUAACA) | 3.150 | TargetScan Human | Moderate (predicted) | FAM156A/FAM156B | mily with sequence similarity 156 member | Nucleus | other |
| MIMAT0000245 | miR-30c-5p (and other miRNAs w/seed GUAACA) | 3.150 | TargetScan Human | Moderate (predicted) | FAM189A2 | mily with sequence similarity 189 member | Other | other |
| MIMAT0000245 | miR-30c-5p (and other miRNAs w/seed GUAACA) | 3.150 | TargetScan Human | Moderate (predicted) | FAM214A | mily with sequence similarity 214 member | Other | other |
| MIMAT0000245 | miR-30c-5p (and other miRNAs w/seed GUAACA) | 3.150 | TargetScan Human | Moderate (predicted) | FAM217B | mily with sequence similarity 217 member | Other | other |
| MIMAT0000245 | miR-30c-5p (and other miRNAs w/seed GUAACA) | 3.150 | TargetScan Human | High (predicted) | FAM24A | amily with sequence similarity 24 member | Other | other |
| MIMAT0000245 | miR-30c-5p (and other miRNAs w/seed GUAACA) | 3.150 | TargetScan Human | High (predicted) | FAM43A | amily with sequence similarity 43 member | Other | other |
| MIMAT0000245 | miR-30c-5p (and other miRNAs w/seed GUAACA) | 3.150 | TargetScan Human | Moderate (predicted) | FAM72B | amily with sequence similarity 72 member | Cytoplasm | other |
| MIMAT0000245 | miR-30c-5p (and other miRNAs w/seed GUAACA) | 3.150 | TargetScan Human | Moderate (predicted) | FAM72C/FAM72D | amily with sequence similarity 72 member | Nucleus | other |
| MIMAT0000245 | miR-30c-5p (and other miRNAs w/seed GUAACA) | 3.150 | TargetScan Human | High (predicted) | FAM81A | amily with sequence similarity 81 member | Other | other |
| MIMAT0000245 | miR-30c-5p (and other miRNAs w/seed GUAACA) | 3.150 | TargetScan Human | Moderate (predicted) | FAM91A1 | mily with sequence similarity 91 member A | Cytoplasm | other |
| MIMAT0000245 | miR-30c-5p (and other miRNAs w/seed GUAACA) | 3.150 | TargetScan Human | Moderate (predicted) | FANCF | FA complementation group F | Nucleus | other |
| MIMAT0000245 | miR-30c-5p (and other miRNAs w/seed GUAACA) | 3.150 | TargetScan Human | High (predicted) | FAP | fibroblast activation protein alpha | Cytoplasm | peptidase |
| MIMAT0000245 | miR-30c-5p (and other miRNAs w/seed GUAACA) | 3.150 | TargetScan Human | Moderate (predicted) | FBXL14 | F-box and leucine rich repeat protein 14 | Cytoplasm | enzyme |
| MIMAT0000245 | miR-30c-5p (and other miRNAs w/seed GUAACA) | 3.150 | TargetScan Human | Moderate (predicted) | FBXL3 | F-box and leucine rich repeat protein 3 | Nucleus | enzyme |
| MIMAT0000245 | miR-30c-5p (and other miRNAs w/seed GUAACA) | 3.150 | TargetScan Human | Moderate (predicted) | FBXO32 | F-box protein 32 | Cytoplasm | enzyme |
| MIMAT0000245 | miR-30c-5p (and other miRNAs w/seed GUAACA) | 3.150 | TargetScan Human | Moderate (predicted) | FBXO39 | F-box protein 39 | Other | other |
| MIMAT0000245 | miR-30c-5p (and other miRNAs w/seed GUAACA) | 3.150 | TargetScan Human | High (predicted) | FBXO45 | F-box protein 45 | Plasma Membrane | other |
| MIMAT0000245 | miR-30c-5p (and other miRNAs w/seed GUAACA) | 3.150 | TargetScan Human | Moderate (predicted) | FCER1G | Fc fragment of IgE receptor Ig | Plasma Membrane | transmembrane receptor |
| MIMAT0000245 | miR-30c-5p (and other miRNAs w/seed GUAACA) | 3.150 | TargetScan Human | Moderate (predicted) | FCN1 | ficolin 1 | Extracellular Space | other |
| MIMAT0000245 | miR-30c-5p (and other miRNAs w/seed GUAACA) | 3.150 | TargetScan Human | Moderate (predicted) | FGF20 | fibroblast growth factor 20 | Extracellular Space | growth factor |
| MIMAT0000245 | miR-30c-5p (and other miRNAs w/seed GUAACA) | 3.150 | TargetScan Human | Moderate (predicted) | FHIP2A | complex subunit HOOK interacting protei | Other | other |
| MIMAT0000245 | miR-30c-5p (and other miRNAs w/seed GUAACA) | 3.150 | TargetScan Human | Moderate (predicted) | FIGN | fidgetin, microtubule severing factor | Nucleus | other |
| MIMAT0000245 | miR-30c-5p (and other miRNAs w/seed GUAACA) | 3.150 | TargetScan Human | Moderate (predicted) | FKBP3 | FKBP prolyl isomerase 3 | Nucleus | enzyme |
| MIMAT0000245 | miR-30c-5p (and other miRNAs w/seed GUAACA) | 3.150 | TargetScan Human | Moderate (predicted) | FLVCR1 | FLVCR heme transporter 1 | Plasma Membrane | transporter |
| MIMAT0000245 | miR-30c-5p (and other miRNAs w/seed GUAACA) | 3.150 | TargetScan Human | Moderate (predicted) | FLVCR2 | FLVCR heme transporter 2 | Plasma Membrane | transporter |
| MIMAT0000245 | miR-30c-5p (and other miRNAs w/seed GUAACA) | 3.150 | TargetScan Human | Moderate (predicted) | FMR1NB | FMR1 neighbor | Nucleus | other |
| MIMAT0000245 | miR-30c-5p (and other miRNAs w/seed GUAACA) | 3.150 | TargetScan Human | Moderate (predicted) | FNDC3A | fibronectin type III domain containing 3A | Cytoplasm | other |
| MIMAT0000245 | miR-30c-5p (and other miRNAs w/seed GUAACA) | 3.150 | TargetScan Human | Moderate (predicted) | FOSL2 | FOS like 2, AP-1 transcription factor subun | Nucleus | transcription regulator |
| MIMAT0000245 | miR-30c-5p (and other miRNAs w/seed GUAACA) | 3.150 | TargetScan Human | Moderate (predicted) | FOXA1 | forkhead box A1 | Nucleus | transcription regulator |
| MIMAT0000245 | miR-30c-5p (and other miRNAs w/seed GUAACA) | 3.150 | TargetScan Human | Moderate (predicted) | FOXB1 | forkhead box B1 | Nucleus | transcription regulator |
| MIMAT0000245 | miR-30c-5p (and other miRNAs w/seed GUAACA) | 3.150 | TargetScan Human | Moderate (predicted) | FOXD1 | forkhead box D1 | Nucleus | transcription regulator |
| MIMAT0000245 | miR-30c-5p (and other miRNAs w/seed GUAACA) | 3.150 | TargetScan Human | High (predicted) | FOXG1 | forkhead box G1 | Nucleus | transcription regulator |
| MIMAT0000245 | miR-30c-5p (and other miRNAs w/seed GUAACA) | 3.150 | TarBase | Experimentally Observed | FRG1 | FSHD region gene 1 | Nucleus | transporter |
| MIMAT0000245 | miR-30c-5p (and other miRNAs w/seed GUAACA) | 3.150 | TargetScan Human | Moderate (predicted) | FRMD6 | FERM domain containing 6 | Cytoplasm | other |
| MIMAT0000245 | miR-30c-5p (and other miRNAs w/seed GUAACA) | 3.150 | TargetScan Human | Moderate (predicted) | FRMPD1 | FERM and PDZ domain containing 1 | Cytoplasm | other |
| MIMAT0000245 | miR-30c-5p (and other miRNAs w/seed GUAACA) | 3.150 | TargetScan Human | Moderate (predicted) | FRZB | frizzled related protein | Extracellular Space | other |
| MIMAT0000245 | miR-30c-5p (and other miRNAs w/seed GUAACA) | 3.150 | TargetScan Human | Moderate (predicted) | FUCA1 | alpha-L-fucosidase 1 | Cytoplasm | enzyme |
| MIMAT0000245 | miR-30c-5p (and other miRNAs w/seed GUAACA) | 3.150 | TargetScan Human | Moderate (predicted) | FXR1 | FMR1 autosomal homolog 1 | Cytoplasm | other |
| MIMAT0000245 | miR-30c-5p (and other miRNAs w/seed GUAACA) | 3.150 | TarBase | Experimentally Observed | FXR2 | FMR1 autosomal homolog 2 | Cytoplasm | other |
| MIMAT0000245 | miR-30c-5p (and other miRNAs w/seed GUAACA) | 3.150 | TargetScan Human | High (predicted) | FZD3 | frizzled class receptor 3 | Plasma Membrane | G-protein coupled receptor |
| MIMAT0000245 | miR-30c-5p (and other miRNAs w/seed GUAACA) | 3.150 | TargetScan Human | Moderate (predicted) | GABRA5 | -aminobutyric acid type A receptor subuni | Plasma Membrane | ion channel |
| MIMAT0000245 | miR-30c-5p (and other miRNAs w/seed GUAACA) | 3.150 | TargetScan Human | High (predicted) | GABRB1 | a-aminobutyric acid type A receptor subun | Plasma Membrane | ion channel |
| MIMAT0000245 | miR-30c-5p (and other miRNAs w/seed GUAACA) | 3.150 | arBase,TargetScan Hum | ntally Observed,Moderate ( | GALNT1 | ypeptide N-acetylgalactosaminyltransferas | Cytoplasm | enzyme |
| MIMAT0000245 | miR-30c-5p (and other miRNAs w/seed GUAACA) | 3.150 | TargetScan Human | Moderate (predicted) | GALNT3 | ypeptide N-acetylgalactosaminyltransferas | Cytoplasm | enzyme |
| MIMAT0000245 | miR-30c-5p (and other miRNAs w/seed GUAACA) | 3.150 | arBase,TargetScan Hum | nentally Observed,High (pr | GALNT7 | ypeptide N-acetylgalactosaminyltransferas | Cytoplasm | enzyme |
| MIMAT0000245 | miR-30c-5p (and other miRNAs w/seed GUAACA) | 3.150 | TargetScan Human | Moderate (predicted) | GALR1 | galanin receptor 1 | Plasma Membrane | G-protein coupled receptor |
| MIMAT0000245 | miR-30c-5p (and other miRNAs w/seed GUAACA) | 3.150 | TargetScan Human | High (predicted) | GAN | gigaxonin | Cytoplasm | other |
| MIMAT0000245 | miR-30c-5p (and other miRNAs w/seed GUAACA) | 3.150 | TargetScan Human | Moderate (predicted) | GANC | glucosidase alpha, neutral C | Extracellular Space | enzyme |
| MIMAT0000245 | miR-30c-5p (and other miRNAs w/seed GUAACA) | 3.150 | TargetScan Human | Moderate (predicted) | GAS2L3 | growth arrest specific 2 like 3 | Other | other |
| MIMAT0000245 | miR-30c-5p (and other miRNAs w/seed GUAACA) | 3.150 | TargetScan Human | Moderate (predicted) | GATA5 | GATA binding protein 5 | Nucleus | transcription regulator |
| MIMAT0000245 | miR-30c-5p (and other miRNAs w/seed GUAACA) | 3.150 | TargetScan Human | Moderate (predicted) | GATM | glycine amidinotransferase | Cytoplasm | enzyme |
| MIMAT0000245 | miR-30c-5p (and other miRNAs w/seed GUAACA) | 3.150 | TargetScan Human | Moderate (predicted) | GCG | glucagon | Plasma Membrane | other |
| MIMAT0000245 | miR-30c-5p (and other miRNAs w/seed GUAACA) | 3.150 | TargetScan Human | Moderate (predicted) | GCLC | glutamate-cysteine ligase catalytic subuni | Cytoplasm | enzyme |
| MIMAT0000245 | miR-30c-5p (and other miRNAs w/seed GUAACA) | 3.150 | TargetScan Human | High (predicted) | GCSAM | minal center associated signaling and mo | Cytoplasm | other |
| MIMAT0000245 | miR-30c-5p (and other miRNAs w/seed GUAACA) | 3.150 | TargetScan Human | Moderate (predicted) | GFPT2 | tamine-fructose-6-phosphate transaminas | Cytoplasm | enzyme |
| MIMAT0000245 | miR-30c-5p (and other miRNAs w/seed GUAACA) | 3.150 | TargetScan Human | Moderate (predicted) | GJA1 | gap junction protein alpha 1 | Plasma Membrane | transporter |
| MIMAT0000245 | miR-30c-5p (and other miRNAs w/seed GUAACA) | 3.150 | TargetScan Human | Moderate (predicted) | GLCCI1 | glucocorticoid induced 1 | Cytoplasm | other |
| MIMAT0000245 | miR-30c-5p (and other miRNAs w/seed GUAACA) | 3.150 | TargetScan Human | Moderate (predicted) | GLDC | glycine decarboxylase | Cytoplasm | enzyme |
| MIMAT0000245 | miR-30c-5p (and other miRNAs w/seed GUAACA) | 3.150 | TargetScan Human | Moderate (predicted) | GLUD1 | glutamate dehydrogenase 1 | Cytoplasm | enzyme |
| MIMAT0000245 | miR-30c-5p (and other miRNAs w/seed GUAACA) | 3.150 | TargetScan Human | Moderate (predicted) | GLUD2 | glutamate dehydrogenase 2 | Cytoplasm | enzyme |
| MIMAT0000245 | miR-30c-5p (and other miRNAs w/seed GUAACA) | 3.150 | TargetScan Human | Moderate (predicted) | GM2A | GM2 ganglioside activator | Cytoplasm | enzyme |
| MIMAT0000245 | miR-30c-5p (and other miRNAs w/seed GUAACA) | 3.150 | TargetScan Human | High (predicted) | GMNC | geminin coiled-coil domain containing | Nucleus | other |
| MIMAT0000245 | miR-30c-5p (and other miRNAs w/seed GUAACA) | 3.150 | TargetScan Human | High (predicted) | GNA13 | G protein subunit alpha 13 | Plasma Membrane | enzyme |
| MIMAT0000245 | miR-30c-5p (and other miRNAs w/seed GUAACA) | 3.150 | e,TargetScan Human,mi | ntally Observed,Moderate ( | GNAI2 | G protein subunit alpha i2 | Plasma Membrane | enzyme |
| MIMAT0000245 | miR-30c-5p (and other miRNAs w/seed GUAACA) | 3.150 | TargetScan Human | Moderate (predicted) | GNAO1 | G protein subunit alpha o1 | Plasma Membrane | enzyme |
| MIMAT0000245 | miR-30c-5p (and other miRNAs w/seed GUAACA) | 3.150 | TargetScan Human | Moderate (predicted) | GNAQ | G protein subunit alpha q | Plasma Membrane | enzyme |
| MIMAT0000245 | miR-30c-5p (and other miRNAs w/seed GUAACA) | 3.150 | TargetScan Human | High (predicted) | GNG10 | G protein subunit gamma 10 | Plasma Membrane | enzyme |
| MIMAT0000245 | miR-30c-5p (and other miRNAs w/seed GUAACA) | 3.150 | TargetScan Human | High (predicted) | GNPDA1 | glucosamine-6-phosphate deaminase 1 | Cytoplasm | enzyme |
| MIMAT0000245 | miR-30c-5p (and other miRNAs w/seed GUAACA) | 3.150 | TargetScan Human | Moderate (predicted) | GOLGA1 | golgin A1 | Cytoplasm | other |
| MIMAT0000245 | miR-30c-5p (and other miRNAs w/seed GUAACA) | 3.150 | TargetScan Human | Moderate (predicted) | GOLGA4 | golgin A4 | Cytoplasm | other |
| MIMAT0000245 | miR-30c-5p (and other miRNAs w/seed GUAACA) | 3.150 | TargetScan Human | Moderate (predicted) | GORAB | golgin, RAB6 interacting | Cytoplasm | other |
| MIMAT0000245 | miR-30c-5p (and other miRNAs w/seed GUAACA) | 3.150 | TargetScan Human | Moderate (predicted) | GPCPD1 | lycerophosphocholine phosphodiesterase | Cytoplasm | enzyme |
| MIMAT0000245 | miR-30c-5p (and other miRNAs w/seed GUAACA) | 3.150 | TarBase | Experimentally Observed | GPD2 | glycerol-3-phosphate dehydrogenase 2 | Cytoplasm | enzyme |
| MIMAT0000245 | miR-30c-5p (and other miRNAs w/seed GUAACA) | 3.150 | TargetScan Human | Moderate (predicted) | GPR12 | G protein-coupled receptor 12 | Plasma Membrane | G-protein coupled receptor |
| MIMAT0000245 | miR-30c-5p (and other miRNAs w/seed GUAACA) | 3.150 | TargetScan Human | Moderate (predicted) | GPR150 | G protein-coupled receptor 150 | Plasma Membrane | G-protein coupled receptor |
| MIMAT0000245 | miR-30c-5p (and other miRNAs w/seed GUAACA) | 3.150 | TargetScan Human | Moderate (predicted) | GRAMD2A | GRAM domain containing 2A | Other | other |
| MIMAT0000245 | miR-30c-5p (and other miRNAs w/seed GUAACA) | 3.150 | TargetScan Human | Moderate (predicted) | GRB10 | growth factor receptor bound protein 10 | Cytoplasm | other |
| MIMAT0000245 | miR-30c-5p (and other miRNAs w/seed GUAACA) | 3.150 | TargetScan Human | Moderate (predicted) | GRHL2 | grainyhead like transcription factor 2 | Nucleus | transcription regulator |
| MIMAT0000245 | miR-30c-5p (and other miRNAs w/seed GUAACA) | 3.150 | TargetScan Human | High (predicted) | GSKIP | GSK3B interacting protein | Nucleus | other |
| MIMAT0000245 | miR-30c-5p (and other miRNAs w/seed GUAACA) | 3.150 | TargetScan Human | Moderate (predicted) | GTF2E2 | general transcription factor IIE subunit 2 | Nucleus | transcription regulator |
| MIMAT0000245 | miR-30c-5p (and other miRNAs w/seed GUAACA) | 3.150 | TargetScan Human | Moderate (predicted) | GTF2H1 | general transcription factor IIH subunit 1 | Nucleus | transcription regulator |
| MIMAT0000245 | miR-30c-5p (and other miRNAs w/seed GUAACA) | 3.150 | TargetScan Human | Moderate (predicted) | GUCY2C | guanylate cyclase 2C | Plasma Membrane | kinase |
| MIMAT0000245 | miR-30c-5p (and other miRNAs w/seed GUAACA) | 3.150 | TargetScan Human | Moderate (predicted) | H2AC15 | H2A clustered histone 15 | Nucleus | other |
| MIMAT0000245 | miR-30c-5p (and other miRNAs w/seed GUAACA) | 3.150 | TargetScan Human | Moderate (predicted) | H3-5 | H3.5 histone | Nucleus | other |
| MIMAT0000245 | miR-30c-5p (and other miRNAs w/seed GUAACA) | 3.150 | TargetScan Human | Moderate (predicted) | H3C4 | H3 clustered histone 4 | Nucleus | other |
| MIMAT0000245 | miR-30c-5p (and other miRNAs w/seed GUAACA) | 3.150 | TargetScan Human | Moderate (predicted) | H4C9 | H4 clustered histone 9 | Nucleus | other |
| MIMAT0000245 | miR-30c-5p (and other miRNAs w/seed GUAACA) | 3.150 | TargetScan Human | Moderate (predicted) | HAO1 | hydroxyacid oxidase 1 | Cytoplasm | enzyme |
| MIMAT0000245 | miR-30c-5p (and other miRNAs w/seed GUAACA) | 3.150 | TargetScan Human | High (predicted) | HECTD2 | HECT domain E3 ubiquitin protein ligase 2 | Cytoplasm | enzyme |
| MIMAT0000245 | miR-30c-5p (and other miRNAs w/seed GUAACA) | 3.150 | TargetScan Human | Moderate (predicted) | HEPHL1 | hephaestin like 1 | Other | enzyme |
| MIMAT0000245 | miR-30c-5p (and other miRNAs w/seed GUAACA) | 3.150 | TargetScan Human | Moderate (predicted) | HIPK2 | homeodomain interacting protein kinase 2 | Nucleus | kinase |
| MIMAT0000245 | miR-30c-5p (and other miRNAs w/seed GUAACA) | 3.150 | TargetScan Human | Moderate (predicted) | HIVEP1 | HIVEP zinc finger 1 | Nucleus | transcription regulator |
| MIMAT0000245 | miR-30c-5p (and other miRNAs w/seed GUAACA) | 3.150 | TargetScan Human | Moderate (predicted) | HLF | F transcription factor, PAR bZIP family mer | Nucleus | transcription regulator |
| MIMAT0000245 | miR-30c-5p (and other miRNAs w/seed GUAACA) | 3.150 | TargetScan Human | Moderate (predicted) | HLX | H2.0 like homeobox | Nucleus | transcription regulator |

Sup. Table 3. List of 3699 targets of the seven dysregulated miRNAs obtained from three different target prediction databases,TargetScan TarBase and miRecords considering those predicted as moderate, high, or experimentally validated, respectively.

| | | | | | | | | |
|---|---|---|---|---|---|---|---|---|
| MIMAT0000245 | miR-30c-5p (and other miRNAs w/seed GUAAACA) | 3.150 | TargetScan Human | Moderate (predicted) | HMGB3 | high mobility group box 3 | Nucleus | other |
| MIMAT0000245 | miR-30c-5p (and other miRNAs w/seed GUAAACA) | 3.150 | TargetScan Human | Moderate (predicted) | HNRNPA1 | heterogeneous nuclear ribonucleoprotein A | Nucleus | other |
| MIMAT0000245 | miR-30c-5p (and other miRNAs w/seed GUAAACA) | 3.150 | TarBase | Experimentally Observed | HNRNPM | heterogeneous nuclear ribonucleoprotein M | Nucleus | other |
| MIMAT0000245 | miR-30c-5p (and other miRNAs w/seed GUAAACA) | 3.150 | TargetScan Human | Moderate (predicted) | HORMAD1 | HORMA domain containing 1 | Nucleus | other |
| MIMAT0000245 | miR-30c-5p (and other miRNAs w/seed GUAAACA) | 3.150 | TargetScan Human | High (predicted) | HOXA1 | homeobox A1 | Nucleus | transcription regulator |
| MIMAT0000245 | miR-30c-5p (and other miRNAs w/seed GUAAACA) | 3.150 | TargetScan Human | Moderate (predicted) | HOXA11 | homeobox A11 | Nucleus | transcription regulator |
| MIMAT0000245 | miR-30c-5p (and other miRNAs w/seed GUAAACA) | 3.150 | TargetScan Human | Moderate (predicted) | HOXB8 | homeobox B8 | Nucleus | transcription regulator |
| MIMAT0000245 | miR-30c-5p (and other miRNAs w/seed GUAAACA) | 3.150 | TargetScan Human | Moderate (predicted) | HPS4 | genesis of lysosomal organelles complex 3 | Cytoplasm | other |
| MIMAT0000245 | miR-30c-5p (and other miRNAs w/seed GUAAACA) | 3.150 | TargetScan Human | Moderate (predicted) | HSFY1/HSFY2 | heat shock transcription factor Y-linked 1 | Nucleus | transcription regulator |
| MIMAT0000245 | miR-30c-5p (and other miRNAs w/seed GUAAACA) | 3.150 | TargetScan Human | High (predicted) | HTR1F | 5-hydroxytryptamine receptor 1F | Plasma Membrane | G-protein coupled receptor |
| MIMAT0000245 | miR-30c-5p (and other miRNAs w/seed GUAAACA) | 3.150 | TargetScan Human | Moderate (predicted) | HTRA3 | HtrA serine peptidase 3 | Extracellular Space | peptidase |
| MIMAT0000245 | miR-30c-5p (and other miRNAs w/seed GUAAACA) | 3.150 | arBase,TargetScan Hum | entally Observed,High (pr | IDH1 | isocitrate dehydrogenase (NADP(+)) 1 | Cytoplasm | enzyme |
| MIMAT0000245 | miR-30c-5p (and other miRNAs w/seed GUAAACA) | 3.150 | TargetScan Human | Moderate (predicted) | IER5 | immediate early response 5 | Nucleus | other |
| MIMAT0000245 | miR-30c-5p (and other miRNAs w/seed GUAAACA) | 3.150 | TarBase | Experimentally Observed | IFRD1 | nterferon related developmental regulator | Nucleus | other |
| MIMAT0000245 | miR-30c-5p (and other miRNAs w/seed GUAAACA) | 3.150 | TargetScan Human | Moderate (predicted) | IGF2BP2-AS1 | IGF2BP2 antisense RNA 1 | Other | other |
| MIMAT0000245 | miR-30c-5p (and other miRNAs w/seed GUAAACA) | 3.150 | TargetScan Human | Moderate (predicted) | IKZF2 | IKAROS family zinc finger 2 | Nucleus | transcription regulator |
| MIMAT0000245 | miR-30c-5p (and other miRNAs w/seed GUAAACA) | 3.150 | TargetScan Human | Moderate (predicted) | IL1A | interleukin 1 alpha | Extracellular Space | cytokine |
| MIMAT0000245 | miR-30c-5p (and other miRNAs w/seed GUAAACA) | 3.150 | TargetScan Human | High (predicted) | IL1RAPL2 | terleukin 1 receptor accessory protein like | Plasma Membrane | transmembrane receptor |
| MIMAT0000245 | miR-30c-5p (and other miRNAs w/seed GUAAACA) | 3.150 | TargetScan Human | Moderate (predicted) | IL25 | interleukin 25 | Extracellular Space | cytokine |
| MIMAT0000245 | miR-30c-5p (and other miRNAs w/seed GUAAACA) | 3.150 | TargetScan Human | Moderate (predicted) | IL2RA | interleukin 2 receptor subunit alpha | Plasma Membrane | transmembrane receptor |
| MIMAT0000245 | miR-30c-5p (and other miRNAs w/seed GUAAACA) | 3.150 | TargetScan Human | Moderate (predicted) | IL7 | interleukin 7 | Extracellular Space | cytokine |
| MIMAT0000245 | miR-30c-5p (and other miRNAs w/seed GUAAACA) | 3.150 | TargetScan Human | Moderate (predicted) | ING5 | inhibitor of growth family member 5 | Nucleus | other |
| MIMAT0000245 | miR-30c-5p (and other miRNAs w/seed GUAAACA) | 3.150 | TargetScan Human | High (predicted) | INO80D | INO80 complex subunit D | Nucleus | other |
| MIMAT0000245 | miR-30c-5p (and other miRNAs w/seed GUAAACA) | 3.150 | TargetScan Human | Moderate (predicted) | INSIG2 | insulin induced gene 2 | Cytoplasm | other |
| MIMAT0000245 | miR-30c-5p (and other miRNAs w/seed GUAAACA) | 3.150 | TargetScan Human | High (predicted) | IP6K3 | inositol hexakisphosphate kinase 3 | Cytoplasm | kinase |
| MIMAT0000245 | miR-30c-5p (and other miRNAs w/seed GUAAACA) | 3.150 | TargetScan Human | Moderate (predicted) | IQCB1 | IQ motif containing B1 | Extracellular Space | other |
| MIMAT0000245 | miR-30c-5p (and other miRNAs w/seed GUAAACA) | 3.150 | TargetScan Human | Moderate (predicted) | IQCG | IQ motif containing G | Cytoplasm | other |
| MIMAT0000245 | miR-30c-5p (and other miRNAs w/seed GUAAACA) | 3.150 | TargetScan Human | Moderate (predicted) | IRAK1BP1 | kin 1 receptor associated kinase 1 binding | Other | other |
| MIMAT0000245 | miR-30c-5p (and other miRNAs w/seed GUAAACA) | 3.150 | TargetScan Human | Moderate (predicted) | IRS1 | insulin receptor substrate 1 | Cytoplasm | enzyme |
| MIMAT0000245 | miR-30c-5p (and other miRNAs w/seed GUAAACA) | 3.150 | TargetScan Human | High (predicted) | IRS4 | insulin receptor substrate 4 | Plasma Membrane | other |
| MIMAT0000245 | miR-30c-5p (and other miRNAs w/seed GUAAACA) | 3.150 | TargetScan Human | Moderate (predicted) | IRX4 | iroquois homeobox 4 | Nucleus | transcription regulator |
| MIMAT0000245 | miR-30c-5p (and other miRNAs w/seed GUAAACA) | 3.150 | arBase,TargetScan Hum | ntally Observed,Moderate ( | ITGA2 | integrin subunit alpha 2 | Plasma Membrane | transmembrane receptor |
| MIMAT0000245 | miR-30c-5p (and other miRNAs w/seed GUAAACA) | 3.150 | TargetScan Human | Moderate (predicted) | ITGA4 | integrin subunit alpha 4 | Plasma Membrane | transmembrane receptor |
| MIMAT0000245 | miR-30c-5p (and other miRNAs w/seed GUAAACA) | 3.150 | TargetScan Human | Moderate (predicted) | ITGA6 | integrin subunit alpha 6 | Plasma Membrane | transmembrane receptor |
| MIMAT0000245 | miR-30c-5p (and other miRNAs w/seed GUAAACA) | 3.150 | TargetScan Human | High (predicted) | ITGA8 | integrin subunit alpha 8 | Plasma Membrane | other |
| MIMAT0000245 | miR-30c-5p (and other miRNAs w/seed GUAAACA) | 3.150 | TargetScan Human | Moderate (predicted) | ITPK1 | inositol-tetrakisphosphate 1-kinase | Cytoplasm | kinase |
| MIMAT0000245 | miR-30c-5p (and other miRNAs w/seed GUAAACA) | 3.150 | TargetScan Human | Moderate (predicted) | ITPRIPL1 | ITPRIP like 1 | Other | other |
| MIMAT0000245 | miR-30c-5p (and other miRNAs w/seed GUAAACA) | 3.150 | TargetScan Human | Moderate (predicted) | ITSN1 | intersectin 1 | Cytoplasm | other |
| MIMAT0000245 | miR-30c-5p (and other miRNAs w/seed GUAAACA) | 3.150 | TargetScan Human | Moderate (predicted) | JAK1 | Janus kinase 1 | Cytoplasm | kinase |
| MIMAT0000245 | miR-30c-5p (and other miRNAs w/seed GUAAACA) | 3.150 | TargetScan Human | Moderate (predicted) | JAKMIP2 | us kinase and microtubule interacting prote | Extracellular Space | other |
| MIMAT0000245 | miR-30c-5p (and other miRNAs w/seed GUAAACA) | 3.150 | TargetScan Human | Moderate (predicted) | JAKMIP3 | us kinase and microtubule interacting prot | Other | other |
| MIMAT0000245 | miR-30c-5p (and other miRNAs w/seed GUAAACA) | 3.150 | TargetScan Human | Moderate (predicted) | JARID2 | nji and AT-rich interaction domain contain | Nucleus | transcription regulator |
| MIMAT0000245 | miR-30c-5p (and other miRNAs w/seed GUAAACA) | 3.150 | TargetScan Human | Moderate (predicted) | JDP2 | Jun dimerization protein 2 | Nucleus | transcription regulator |
| MIMAT0000245 | miR-30c-5p (and other miRNAs w/seed GUAAACA) | 3.150 | TargetScan Human | Moderate (predicted) | JPH4 | junctophilin 4 | Cytoplasm | other |
| MIMAT0000245 | miR-30c-5p (and other miRNAs w/seed GUAAACA) | 3.150 | TarBase | Experimentally Observed | JUN | roto-oncogene, AP-1 transcription factor s | Nucleus | transcription regulator |
| MIMAT0000245 | miR-30c-5p (and other miRNAs w/seed GUAAACA) | 3.150 | TargetScan Human | Moderate (predicted) | JUNB | proto-oncogene, AP-1 transcription factor s | Nucleus | transcription regulator |
| MIMAT0000245 | miR-30c-5p (and other miRNAs w/seed GUAAACA) | 3.150 | TargetScan Human | Moderate (predicted) | KCNA4 | um voltage-gated channel subfamily A me | Plasma Membrane | ion channel |
| MIMAT0000245 | miR-30c-5p (and other miRNAs w/seed GUAAACA) | 3.150 | TargetScan Human | Moderate (predicted) | KCNIP4 | sium voltage-gated channel interacting pro | Plasma Membrane | ion channel |
| MIMAT0000245 | miR-30c-5p (and other miRNAs w/seed GUAAACA) | 3.150 | TargetScan Human | Moderate (predicted) | KCNJ3 | n inwardly rectifying channel subfamily J r | Plasma Membrane | ion channel |
| MIMAT0000245 | miR-30c-5p (and other miRNAs w/seed GUAAACA) | 3.150 | TargetScan Human | Moderate (predicted) | KCNMB2 | m-activated channel subfamily M regulato | Plasma Membrane | ion channel |
| MIMAT0000245 | miR-30c-5p (and other miRNAs w/seed GUAAACA) | 3.150 | TargetScan Human | Moderate (predicted) | KCTD16 | um channel tetramerization domain contai | Plasma Membrane | other |
| MIMAT0000245 | miR-30c-5p (and other miRNAs w/seed GUAAACA) | 3.150 | TargetScan Human | Moderate (predicted) | KCTD8 | ium channel tetramerization domain conta | Other | other |
| MIMAT0000245 | miR-30c-5p (and other miRNAs w/seed GUAAACA) | 3.150 | TargetScan Human | Moderate (predicted) | KCTD9 | ium channel tetramerization domain conta | Other | other |
| MIMAT0000245 | miR-30c-5p (and other miRNAs w/seed GUAAACA) | 3.150 | TargetScan Human | Moderate (predicted) | KDM3A | lysine demethylase 3A | Nucleus | transcription regulator |
| MIMAT0000245 | miR-30c-5p (and other miRNAs w/seed GUAAACA) | 3.150 | TargetScan Human | Moderate (predicted) | KIAA2026 | KIAA2026 | Other | other |
| MIMAT0000245 | miR-30c-5p (and other miRNAs w/seed GUAAACA) | 3.150 | TargetScan Human | Moderate (predicted) | KLF10 | Kruppel like factor 10 | Nucleus | transcription regulator |
| MIMAT0000245 | miR-30c-5p (and other miRNAs w/seed GUAAACA) | 3.150 | TargetScan Human | Moderate (predicted) | KLF11 | Kruppel like factor 11 | Nucleus | transcription regulator |
| MIMAT0000245 | miR-30c-5p (and other miRNAs w/seed GUAAACA) | 3.150 | TargetScan Human | Moderate (predicted) | KLF12 | Kruppel like factor 12 | Nucleus | transcription regulator |
| MIMAT0000245 | miR-30c-5p (and other miRNAs w/seed GUAAACA) | 3.150 | TargetScan Human | Moderate (predicted) | KLF9 | Kruppel like factor 9 | Nucleus | transcription regulator |
| MIMAT0000245 | miR-30c-5p (and other miRNAs w/seed GUAAACA) | 3.150 | TargetScan Human | Moderate (predicted) | KLHDC2 | kelch domain containing 2 | Cytoplasm | other |
| MIMAT0000245 | miR-30c-5p (and other miRNAs w/seed GUAAACA) | 3.150 | TargetScan Human | High (predicted) | KLHL20 | kelch like family member 20 | Plasma Membrane | enzyme |
| MIMAT0000245 | miR-30c-5p (and other miRNAs w/seed GUAAACA) | 3.150 | TargetScan Human | High (predicted) | KLHL28 | kelch like family member 28 | Other | other |
| MIMAT0000245 | miR-30c-5p (and other miRNAs w/seed GUAAACA) | 3.150 | TargetScan Human | Moderate (predicted) | KMT2C | lysine methyltransferase 2C | Nucleus | transcription regulator |
| MIMAT0000245 | miR-30c-5p (and other miRNAs w/seed GUAAACA) | 3.150 | TargetScan Human | Moderate (predicted) | KPNA3 | karyopherin subunit alpha 3 | Nucleus | transporter |
| MIMAT0000245 | miR-30c-5p (and other miRNAs w/seed GUAAACA) | 3.150 | miRecords | Experimentally Observed | KRT7 | keratin 7 | Cytoplasm | other |
| MIMAT0000245 | miR-30c-5p (and other miRNAs w/seed GUAAACA) | 3.150 | TarBase | Experimentally Observed | KRT85 | keratin 85 | Extracellular Space | other |
| MIMAT0000245 | miR-30c-5p (and other miRNAs w/seed GUAAACA) | 3.150 | TargetScan Human | Moderate (predicted) | KRTAP1-5 | keratin associated protein 1-5 | Cytoplasm | other |
| MIMAT0000245 | miR-30c-5p (and other miRNAs w/seed GUAAACA) | 3.150 | TargetScan Human | Moderate (predicted) | KRTAP26-1 | keratin associated protein 26-1 | Cytoplasm | other |
| MIMAT0000245 | miR-30c-5p (and other miRNAs w/seed GUAAACA) | 3.150 | TargetScan Human | High (predicted) | KXD1 | KxDL motif containing 1 | Cytoplasm | other |
| MIMAT0000245 | miR-30c-5p (and other miRNAs w/seed GUAAACA) | 3.150 | TargetScan Human | Moderate (predicted) | L3MBTL4 | MBTL histone methyl-lysine binding protei | Other | other |
| MIMAT0000245 | miR-30c-5p (and other miRNAs w/seed GUAAACA) | 3.150 | ngenuity Expert Finding | Experimentally Observed | LAMC1 | laminin subunit gamma 1 | Extracellular Space | other |
| MIMAT0000245 | miR-30c-5p (and other miRNAs w/seed GUAAACA) | 3.150 | TargetScan Human | Moderate (predicted) | LARGE1 | LARGE xylosyl- and glucuronyltransferase | Cytoplasm | enzyme |
| MIMAT0000245 | miR-30c-5p (and other miRNAs w/seed GUAAACA) | 3.150 | TargetScan Human | Moderate (predicted) | LCLAT1 | lysocardiolipin acyltransferase 1 | Cytoplasm | enzyme |
| MIMAT0000245 | miR-30c-5p (and other miRNAs w/seed GUAAACA) | 3.150 | TargetScan Human | Moderate (predicted) | LCOR | and dependent nuclear receptor corepress | Nucleus | transcription regulator |
| MIMAT0000245 | miR-30c-5p (and other miRNAs w/seed GUAAACA) | 3.150 | TargetScan Human | Moderate (predicted) | LCP1 | lymphocyte cytosolic protein 1 | Cytoplasm | other |
| MIMAT0000245 | miR-30c-5p (and other miRNAs w/seed GUAAACA) | 3.150 | TargetScan Human | Moderate (predicted) | LEFTY1 | left-right determination factor 1 | Extracellular Space | growth factor |
| MIMAT0000245 | miR-30c-5p (and other miRNAs w/seed GUAAACA) | 3.150 | TargetScan Human | High (predicted) | LGI1 | leucine rich glioma inactivated 1 | Plasma Membrane | other |
| MIMAT0000245 | miR-30c-5p (and other miRNAs w/seed GUAAACA) | 3.150 | TargetScan Human | High (predicted) | LHX1 | LIM homeobox 1 | Nucleus | transcription regulator |
| MIMAT0000245 | miR-30c-5p (and other miRNAs w/seed GUAAACA) | 3.150 | TargetScan Human | Moderate (predicted) | LHX5 | LIM homeobox 5 | Nucleus | transcription regulator |
| MIMAT0000245 | miR-30c-5p (and other miRNAs w/seed GUAAACA) | 3.150 | TargetScan Human | High (predicted) | LHX8 | LIM homeobox 8 | Nucleus | transcription regulator |
| MIMAT0000245 | miR-30c-5p (and other miRNAs w/seed GUAAACA) | 3.150 | TargetScan Human | High (predicted) | LHX9 | LIM homeobox 9 | Nucleus | transcription regulator |
| MIMAT0000245 | miR-30c-5p (and other miRNAs w/seed GUAAACA) | 3.150 | TargetScan Human | Moderate (predicted) | LIMCH1 | LIM and calponin homology domains 1 | Cytoplasm | other |
| MIMAT0000245 | miR-30c-5p (and other miRNAs w/seed GUAAACA) | 3.150 | TargetScan Human | Moderate (predicted) | LIMS1 | LIM zinc finger domain containing 1 | Plasma Membrane | other |
| MIMAT0000245 | miR-30c-5p (and other miRNAs w/seed GUAAACA) | 3.150 | TargetScan Human | High (predicted) | LIN28A | lin-28 homolog A | Cytoplasm | other |
| MIMAT0000245 | miR-30c-5p (and other miRNAs w/seed GUAAACA) | 3.150 | TargetScan Human | High (predicted) | LIN28B | lin-28 homolog B | Cytoplasm | other |
| MIMAT0000245 | miR-30c-5p (and other miRNAs w/seed GUAAACA) | 3.150 | TargetScan Human | Moderate (predicted) | LIN7C | molog C, crumbs cell polarity complex cor | Cytoplasm | other |
| MIMAT0000245 | miR-30c-5p (and other miRNAs w/seed GUAAACA) | 3.150 | TargetScan Human | Moderate (predicted) | LINC02656 | ng intergenic non-protein coding RNA 265 | Other | other |
| MIMAT0000245 | miR-30c-5p (and other miRNAs w/seed GUAAACA) | 3.150 | TargetScan Human | Moderate (predicted) | LMBR1 | limb development membrane protein 1 | Plasma Membrane | transmembrane receptor |
| MIMAT0000245 | miR-30c-5p (and other miRNAs w/seed GUAAACA) | 3.150 | TargetScan Human | High (predicted) | LMBR1L | limb development membrane protein 1 like | Plasma Membrane | transmembrane receptor |
| MIMAT0000245 | miR-30c-5p (and other miRNAs w/seed GUAAACA) | 3.150 | TarBase | Experimentally Observed | LMNB2 | lamin B2 | Nucleus | other |
| MIMAT0000245 | miR-30c-5p (and other miRNAs w/seed GUAAACA) | 3.150 | TargetScan Human | Moderate (predicted) | LNPK | lunapark, ER junction formation factor | Cytoplasm | other |
| MIMAT0000245 | miR-30c-5p (and other miRNAs w/seed GUAAACA) | 3.150 | TargetScan Human | Moderate (predicted) | LOC100996419 | uncharacterized LOC100996419 | Other | other |
| MIMAT0000245 | miR-30c-5p (and other miRNAs w/seed GUAAACA) | 3.150 | TargetScan Human | Moderate (predicted) | LONRF1 | peptidase N-terminal domain and ring fin | Cytoplasm | other |
| MIMAT0000245 | miR-30c-5p (and other miRNAs w/seed GUAAACA) | 3.150 | ngenuity Expert Finding | Experimentally Observed | LOXL2 | lysyl oxidase like 2 | Extracellular Space | enzyme |
| MIMAT0000245 | miR-30c-5p (and other miRNAs w/seed GUAAACA) | 3.150 | ngenuity Expert Finding | Experimentally Observed | LOXL4 | lysyl oxidase like 4 | Extracellular Space | enzyme |
| MIMAT0000245 | miR-30c-5p (and other miRNAs w/seed GUAAACA) | 3.150 | TargetScan Human | Moderate (predicted) | LPCAT2 | lysophosphatidylcholine acyltransferase 2 | Cytoplasm | enzyme |
| MIMAT0000245 | miR-30c-5p (and other miRNAs w/seed GUAAACA) | 3.150 | TargetScan Human | Moderate (predicted) | LPGAT1 | lysophosphatidylglycerol acyltransferase 1 | Cytoplasm | enzyme |
| MIMAT0000245 | miR-30c-5p (and other miRNAs w/seed GUAAACA) | 3.150 | TargetScan Human | Moderate (predicted) | LRFN2 | h repeat and fibronectin type III domain co | Plasma Membrane | other |
| MIMAT0000245 | miR-30c-5p (and other miRNAs w/seed GUAAACA) | 3.150 | TargetScan Human | High (predicted) | LRMDA | cine rich melanocyte differentiation associa | Other | other |
| MIMAT0000245 | miR-30c-5p (and other miRNAs w/seed GUAAACA) | 3.150 | TargetScan Human | High (predicted) | LRRC17 | leucine rich repeat containing 17 | Extracellular Space | other |
| MIMAT0000245 | miR-30c-5p (and other miRNAs w/seed GUAAACA) | 3.150 | TarBase | Experimentally Observed | LRRC8C | cine rich repeat containing 8 VRAC subun | Plasma Membrane | ion channel |
| MIMAT0000245 | miR-30c-5p (and other miRNAs w/seed GUAAACA) | 3.150 | TargetScan Human | High (predicted) | LRRC8D | cine rich repeat containing 8 VRAC subun | Plasma Membrane | G-protein coupled receptor |
| MIMAT0000245 | miR-30c-5p (and other miRNAs w/seed GUAAACA) | 3.150 | TargetScan Human | Moderate (predicted) | LSM1 | M1 homolog, mRNA degradation associat | Nucleus | other |
| MIMAT0000245 | miR-30c-5p (and other miRNAs w/seed GUAAACA) | 3.150 | TargetScan Human | Moderate (predicted) | LSM14B | LSM family member 14B | Other | other |
| MIMAT0000245 | miR-30c-5p (and other miRNAs w/seed GUAAACA) | 3.150 | TarBase | Experimentally Observed | LTN1 | listerin E3 ubiquitin protein ligase 1 | Nucleus | enzyme |
| MIMAT0000245 | miR-30c-5p (and other miRNAs w/seed GUAAACA) | 3.150 | TargetScan Human | Moderate (predicted) | LYN | N proto-oncogene, Src family tyrosine kina | Cytoplasm | kinase |
| MIMAT0000245 | miR-30c-5p (and other miRNAs w/seed GUAAACA) | 3.150 | TargetScan Human | Moderate (predicted) | LYSMD3 | LysM domain containing 3 | Other | other |
| MIMAT0000245 | miR-30c-5p (and other miRNAs w/seed GUAAACA) | 3.150 | TargetScan Human | Moderate (predicted) | M1AP | meiosis 1 associated protein | Cytoplasm | other |
| MIMAT0000245 | miR-30c-5p (and other miRNAs w/seed GUAAACA) | 3.150 | TargetScan Human | Moderate (predicted) | MAB21L1 | mab-21 like 1 | Nucleus | other |
| MIMAT0000245 | miR-30c-5p (and other miRNAs w/seed GUAAACA) | 3.150 | TargetScan Human | Moderate (predicted) | MAML1 | mastermind like transcriptional coactivator | Nucleus | transcription regulator |
| MIMAT0000245 | miR-30c-5p (and other miRNAs w/seed GUAAACA) | 3.150 | TargetScan Human | Moderate (predicted) | MAN1A2 | mannosidase alpha class 1A member 2 | Cytoplasm | enzyme |
| MIMAT0000245 | miR-30c-5p (and other miRNAs w/seed GUAAACA) | 3.150 | TargetScan Human | Moderate (predicted) | MAP3K1 | ogen-activated protein kinase kinase kinas | Cytoplasm | kinase |
| MIMAT0000245 | miR-30c-5p (and other miRNAs w/seed GUAAACA) | 3.150 | TargetScan Human | Moderate (predicted) | MAP3K12 | ogen-activated protein kinase kinase kinas | Cytoplasm | kinase |
| MIMAT0000245 | miR-30c-5p (and other miRNAs w/seed GUAAACA) | 3.150 | TargetScan Human | Moderate (predicted) | MAP3K15 | ogen-activated protein kinase kinase kinas | Other | other |
| MIMAT0000245 | miR-30c-5p (and other miRNAs w/seed GUAAACA) | 3.150 | TargetScan Human | Moderate (predicted) | MAP3K19 | ogen-activated protein kinase kinase kinas | Other | kinase |
| MIMAT0000245 | miR-30c-5p (and other miRNAs w/seed GUAAACA) | 3.150 | TargetScan Human | High (predicted) | MAP3K2 | ogen-activated protein kinase kinase kinas | Cytoplasm | kinase |
| MIMAT0000245 | miR-30c-5p (and other miRNAs w/seed GUAAACA) | 3.150 | TargetScan Human | Moderate (predicted) | MAP3K21 | ogen-activated protein kinase kinase kinas | Other | kinase |
| MIMAT0000245 | miR-30c-5p (and other miRNAs w/seed GUAAACA) | 3.150 | TargetScan Human | Moderate (predicted) | MAP3K5 | ogen-activated protein kinase kinase kinas | Cytoplasm | kinase |
| MIMAT0000245 | miR-30c-5p (and other miRNAs w/seed GUAAACA) | 3.150 | Expert Findings,TargetS | entally Observed,High (pr | MAP4K4 | n-activated protein kinase kinase kinase k | Cytoplasm | kinase |
| MIMAT0000245 | miR-30c-5p (and other miRNAs w/seed GUAAACA) | 3.150 | TargetScan Human | Moderate (predicted) | MARCHF4 | membrane associated ring-CH-type finger | Cytoplasm | enzyme |
| MIMAT0000245 | miR-30c-5p (and other miRNAs w/seed GUAAACA) | 3.150 | TarBase | Experimentally Observed | MAT2A | methionine adenosyltransferase 2A | Cytoplasm | enzyme |
| MIMAT0000245 | miR-30c-5p (and other miRNAs w/seed GUAAACA) | 3.150 | TargetScan Human | Moderate (predicted) | MATR3 | matrin 3 | Nucleus | other |
| MIMAT0000245 | miR-30c-5p (and other miRNAs w/seed GUAAACA) | 3.150 | arBase,TargetScan Hum | ntally Observed,Moderate ( | MBNL1 | muscleblind like splicing regulator 1 | Nucleus | other |
| MIMAT0000245 | miR-30c-5p (and other miRNAs w/seed GUAAACA) | 3.150 | TargetScan Human | Moderate (predicted) | MBNL2 | muscleblind like splicing regulator 2 | Nucleus | other |
| MIMAT0000245 | miR-30c-5p (and other miRNAs w/seed GUAAACA) | 3.150 | TargetScan Human | Moderate (predicted) | MBNL3 | muscleblind like splicing regulator 3 | Nucleus | other |
| MIMAT0000245 | miR-30c-5p (and other miRNAs w/seed GUAAACA) | 3.150 | TargetScan Human | Moderate (predicted) | MBOAT1 | ane bound O-acyltransferase domain cont | Cytoplasm | enzyme |
| MIMAT0000245 | miR-30c-5p (and other miRNAs w/seed GUAAACA) | 3.150 | TargetScan Human | Moderate (predicted) | MBTPS2 | brane bound transcription factor peptidase, | Cytoplasm | peptidase |
| MIMAT0000245 | miR-30c-5p (and other miRNAs w/seed GUAAACA) | 3.150 | TargetScan Human | Moderate (predicted) | MED6 | mediator complex subunit 6 | Nucleus | transcription regulator |
| MIMAT0000245 | miR-30c-5p (and other miRNAs w/seed GUAAACA) | 3.150 | TargetScan Human | High (predicted) | MEIOB | meiosis specific with OB-fold | Cytoplasm | enzyme |
| MIMAT0000245 | miR-30c-5p (and other miRNAs w/seed GUAAACA) | 3.150 | TargetScan Human | Moderate (predicted) | MEOX2 | mesenchyme homeobox 2 | Nucleus | transcription regulator |
| MIMAT0000245 | miR-30c-5p (and other miRNAs w/seed GUAAACA) | 3.150 | TargetScan Human | Moderate (predicted) | MESD | mesoderm development LRP chaperone | Extracellular Space | other |
| MIMAT0000245 | miR-30c-5p (and other miRNAs w/seed GUAAACA) | 3.150 | TarBase | Experimentally Observed | MET | ET proto-oncogene, receptor tyrosine kina | Plasma Membrane | kinase |
| MIMAT0000245 | miR-30c-5p (and other miRNAs w/seed GUAAACA) | 3.150 | TargetScan Human | Moderate (predicted) | METAP2 | methionyl aminopeptidase 2 | Cytoplasm | peptidase |
| MIMAT0000245 | miR-30c-5p (and other miRNAs w/seed GUAAACA) | 3.150 | TargetScan Human | High (predicted) | MEX3B | mex-3 RNA binding family member B | Cytoplasm | kinase |
| MIMAT0000245 | miR-30c-5p (and other miRNAs w/seed GUAAACA) | 3.150 | TargetScan Human | Moderate (predicted) | MEX3C | mex-3 RNA binding family member C | Cytoplasm | enzyme |
| MIMAT0000245 | miR-30c-5p (and other miRNAs w/seed GUAAACA) | 3.150 | TargetScan Human | Moderate (predicted) | MFAP3L | microfibril associated protein 3 like | Nucleus | other |
| MIMAT0000245 | miR-30c-5p (and other miRNAs w/seed GUAAACA) | 3.150 | TargetScan Human | Moderate (predicted) | MFSD11 | or facilitator superfamily domain containin | Other | other |
| MIMAT0000245 | miR-30c-5p (and other miRNAs w/seed GUAAACA) | 3.150 | TargetScan Human | Moderate (predicted) | MGC15885 | uncharacterized protein MGC15885 | Other | other |
| MIMAT0000245 | miR-30c-5p (and other miRNAs w/seed GUAAACA) | 3.150 | TargetScan Human | Moderate (predicted) | MIA3 | MIA SH3 domain ER export factor 3 | Cytoplasm | other |
| MIMAT0000245 | miR-30c-5p (and other miRNAs w/seed GUAAACA) | 3.150 | TargetScan Human | Moderate (predicted) | MICAL1 | ated monooxygenase, calponin and LIM d | Cytoplasm | enzyme |
| MIMAT0000245 | miR-30c-5p (and other miRNAs w/seed GUAAACA) | 3.150 | TargetScan Human | Moderate (predicted) | MICB | MHC class I polypeptide-related sequence | Plasma Membrane | transmembrane receptor |
| MIMAT0000245 | miR-30c-5p (and other miRNAs w/seed GUAAACA) | 3.150 | TargetScan Human | Moderate (predicted) | MICOS10 | l contact site and cristae organizing syste | Cytoplasm | other |
| MIMAT0000245 | miR-30c-5p (and other miRNAs w/seed GUAAACA) | 3.150 | TargetScan Human | High (predicted) | MIER3 | MIER family member 3 | Nucleus | transcription regulator |
| MIMAT0000245 | miR-30c-5p (and other miRNAs w/seed GUAAACA) | 3.150 | TargetScan Human | Moderate (predicted) | MIGA2 | mitoguardin 2 | Plasma Membrane | other |
| MIMAT0000245 | miR-30c-5p (and other miRNAs w/seed GUAAACA) | 3.150 | TargetScan Human | Moderate (predicted) | MINPP1 | ultiple inositol-polyphosphate phosphatase | Cytoplasm | phosphatase |
| MIMAT0000245 | miR-30c-5p (and other miRNAs w/seed GUAAACA) | 3.150 | TargetScan Human | Moderate (predicted) | MIS12 | MIS12 kinetochore complex component | Nucleus | other |

Sup. Table 3. List of 3699 targets of the seven dysregulated miRNAs obtained from three different target prediction databases,TargetScan TarBase and miRecords considering those predicted as moderate, high, or experimentally validated, respectively.

| | | | | | | | | |
|---|---|---|---|---|---|---|---|---|
| MIMAT0000245 | miR-30c-5p (and other miRNAs w/seed GUAAACA) | 3.150 | TargetScan Human | High (predicted) | MKRN3 | makorin ring finger protein 3 | Nucleus | enzyme |
| MIMAT0000245 | miR-30c-5p (and other miRNAs w/seed GUAAACA) | 3.150 | TarBase | Experimentally Observed | MLLT1 | MLLT1 super elongation complex subunit | Nucleus | transcription regulator |
| MIMAT0000245 | miR-30c-5p (and other miRNAs w/seed GUAAACA) | 3.150 | TarBase | Experimentally Observed | MLLT11 | MLLT11 transcription factor 7 cofactor | Cytoplasm | other |
| MIMAT0000245 | miR-30c-5p (and other miRNAs w/seed GUAAACA) | 3.150 | TargetScan Human | Moderate (predicted) | MMD | ocyte to macrophage differentiation assoc | Plasma Membrane | kinase |
| MIMAT0000245 | miR-30c-5p (and other miRNAs w/seed GUAAACA) | 3.150 | TargetScan Human | Moderate (predicted) | MMP21 | matrix metallopeptidase 21 | Extracellular Space | peptidase |
| MIMAT0000245 | miR-30c-5p (and other miRNAs w/seed GUAAACA) | 3.150 | TargetScan Human | Moderate (predicted) | MOCS3 | molybdenum cofactor synthesis 3 | Cytoplasm | enzyme |
| MIMAT0000245 | miR-30c-5p (and other miRNAs w/seed GUAAACA) | 3.150 | TargetScan Human | Moderate (predicted) | MOV10 | Mov10 RISC complex RNA helicase | Nucleus | enzyme |
| MIMAT0000245 | miR-30c-5p (and other miRNAs w/seed GUAAACA) | 3.150 | TarBase | Experimentally Observed | MPDU1 | mannose-P-dolichol utilization defect 1 | Cytoplasm | other |
| MIMAT0000245 | miR-30c-5p (and other miRNAs w/seed GUAAACA) | 3.150 | TargetScan Human | Moderate (predicted) | MS4A7 | membrane spanning 4-domains A7 | Cytoplasm | other |
| MIMAT0000245 | miR-30c-5p (and other miRNAs w/seed GUAAACA) | 3.150 | TargetScan Human | High (predicted) | MSANTD3-TMEFF1 | MSANTD3-TMEFF1 readthrough | Other | other |
| MIMAT0000245 | miR-30c-5p (and other miRNAs w/seed GUAAACA) | 3.150 | TargetScan Human | Moderate (predicted) | MSANTD4 | T DNA binding domain containing 4 with c | Nucleus | other |
| MIMAT0000245 | miR-30c-5p (and other miRNAs w/seed GUAAACA) | 3.150 | TargetScan Human | Moderate (predicted) | MTBP | MDM2 binding protein | Cytoplasm | other |
| MIMAT0000245 | miR-30c-5p (and other miRNAs w/seed GUAAACA) | 3.150 | TargetScan Human | High (predicted) | MTDH | metadherin | Cytoplasm | transcription regulator |
| MIMAT0000245 | miR-30c-5p (and other miRNAs w/seed GUAAACA) | 3.150 | TargetScan Human | Moderate (predicted) | MTRNR2L10 | MT-RNR2 like 10 | Other | other |
| MIMAT0000245 | miR-30c-5p (and other miRNAs w/seed GUAAACA) | 3.150 | TargetScan Human | Moderate (predicted) | MTTP | microsomal triglyceride transfer protein | Cytoplasm | transporter |
| MIMAT0000245 | miR-30c-5p (and other miRNAs w/seed GUAAACA) | 3.150 | TargetScan Human | Moderate (predicted) | MTX3 | metaxin 3 | Other | other |
| MIMAT0000245 | miR-30c-5p (and other miRNAs w/seed GUAAACA) | 3.150 | TargetScan Human | Moderate (predicted) | MYBL2 | MYB proto-oncogene like 2 | Nucleus | transcription regulator |
| MIMAT0000245 | miR-30c-5p (and other miRNAs w/seed GUAAACA) | 3.150 | TargetScan Human | Moderate (predicted) | MYD88 | )88 innate immune signal transduction ada | Plasma Membrane | other |
| MIMAT0000245 | miR-30c-5p (and other miRNAs w/seed GUAAACA) | 3.150 | TarBase | Experimentally Observed | MYO10 | myosin X | Cytoplasm | enzyme |
| MIMAT0000245 | miR-30c-5p (and other miRNAs w/seed GUAAACA) | 3.150 | TargetScan Human | Moderate (predicted) | MYOZ2 | myozenin 2 | Other | other |
| MIMAT0000245 | miR-30c-5p (and other miRNAs w/seed GUAAACA) | 3.150 | TargetScan Human | Moderate (predicted) | MZT1 | mitotic spindle organizing protein 1 | Cytoplasm | other |
| MIMAT0000245 | miR-30c-5p (and other miRNAs w/seed GUAAACA) | 3.150 | TargetScan Human | Moderate (predicted) | N4BP2 | NEDD4 binding protein 2 | Cytoplasm | enzyme |
| MIMAT0000245 | miR-30c-5p (and other miRNAs w/seed GUAAACA) | 3.150 | TargetScan Human | Moderate (predicted) | NAALADL2 | cetylated alpha-linked acidic dipeptidase li | Nucleus | other |
| MIMAT0000245 | miR-30c-5p (and other miRNAs w/seed GUAAACA) | 3.150 | TargetScan Human | Moderate (predicted) | NAGPA | samine-1-phosphodiester alpha-N-acetylgl | Cytoplasm | enzyme |
| MIMAT0000245 | miR-30c-5p (and other miRNAs w/seed GUAAACA) | 3.150 | TargetScan Human | Moderate (predicted) | NAP1L2 | nucleosome assembly protein 1 like 2 | Nucleus | other |
| MIMAT0000245 | miR-30c-5p (and other miRNAs w/seed GUAAACA) | 3.150 | TarBase | Experimentally Observed | NAPG | NSF attachment protein gamma | Cytoplasm | transporter |
| MIMAT0000245 | miR-30c-5p (and other miRNAs w/seed GUAAACA) | 3.150 | TargetScan Human | Moderate (predicted) | NAV3 | neuron navigator 3 | Nucleus | other |
| MIMAT0000245 | miR-30c-5p (and other miRNAs w/seed GUAAACA) | 3.150 | arBase,TargetScan Hum | ntally Observed,Moderate ( | NCEH1 | neutral cholesterol ester hydrolase 1 | Plasma Membrane | enzyme |
| MIMAT0000245 | miR-30c-5p (and other miRNAs w/seed GUAAACA) | 3.150 | TargetScan Human | Moderate (predicted) | NCK2 | NCK adaptor protein 2 | Cytoplasm | kinase |
| MIMAT0000245 | miR-30c-5p (and other miRNAs w/seed GUAAACA) | 3.150 | TarBase | Experimentally Observed | NCL | nucleolin | Nucleus | other |
| MIMAT0000245 | miR-30c-5p (and other miRNAs w/seed GUAAACA) | 3.150 | TargetScan Human | Moderate (predicted) | NDEL1 | nudE neurodevelopment protein 1 like 1 | Nucleus | peptidase |
| MIMAT0000245 | miR-30c-5p (and other miRNAs w/seed GUAAACA) | 3.150 | TargetScan Human | Moderate (predicted) | NDUFA12 | ADH:ubiquinone oxidoreductase subunit A | Cytoplasm | enzyme |
| MIMAT0000245 | miR-30c-5p (and other miRNAs w/seed GUAAACA) | 3.150 | TargetScan Human | High (predicted) | NECAP1 | NECAP endocytosis associated 1 | Plasma Membrane | other |
| MIMAT0000245 | miR-30c-5p (and other miRNAs w/seed GUAAACA) | 3.150 | TargetScan Human | Moderate (predicted) | NECTIN3 | nectin cell adhesion molecule 3 | Plasma Membrane | other |
| MIMAT0000245 | miR-30c-5p (and other miRNAs w/seed GUAAACA) | 3.150 | TargetScan Human | High (predicted) | NEDD4 | NEDD4 E3 ubiquitin protein ligase | Cytoplasm | enzyme |
| MIMAT0000245 | miR-30c-5p (and other miRNAs w/seed GUAAACA) | 3.150 | TargetScan Human | Moderate (predicted) | NEFM | neurofilament medium | Plasma Membrane | other |
| MIMAT0000245 | miR-30c-5p (and other miRNAs w/seed GUAAACA) | 3.150 | TargetScan Human | Moderate (predicted) | NEIL2 | nei like DNA glycosylase 2 | Nucleus | enzyme |
| MIMAT0000245 | miR-30c-5p (and other miRNAs w/seed GUAAACA) | 3.150 | rgetScan Human,miRec | ntally Observed,Moderate ( | NEUROD1 | neuronal differentiation 1 | Nucleus | transcription regulator |
| MIMAT0000245 | miR-30c-5p (and other miRNAs w/seed GUAAACA) | 3.150 | TargetScan Human | Moderate (predicted) | NEUROD6 | neuronal differentiation 6 | Nucleus | transcription regulator |
| MIMAT0000245 | miR-30c-5p (and other miRNAs w/seed GUAAACA) | 3.150 | TargetScan Human | Moderate (predicted) | NEUROG1 | neurogenin 1 | Nucleus | transcription regulator |
| MIMAT0000245 | miR-30c-5p (and other miRNAs w/seed GUAAACA) | 3.150 | TargetScan Human | Moderate (predicted) | NFAT5 | nuclear factor of activated T cells 5 | Nucleus | transcription regulator |
| MIMAT0000245 | miR-30c-5p (and other miRNAs w/seed GUAAACA) | 3.150 | TargetScan Human | Moderate (predicted) | NFATC3 | nuclear factor of activated T cells 3 | Nucleus | transcription regulator |
| MIMAT0000245 | miR-30c-5p (and other miRNAs w/seed GUAAACA) | 3.150 | TargetScan Human | High (predicted) | NFIB | nuclear factor I B | Nucleus | transcription regulator |
| MIMAT0000245 | miR-30c-5p (and other miRNAs w/seed GUAAACA) | 3.150 | TargetScan Human | Moderate (predicted) | NHLH2 | nescient helix-loop-helix 2 | Nucleus | transcription regulator |
| MIMAT0000245 | miR-30c-5p (and other miRNAs w/seed GUAAACA) | 3.150 | TargetScan Human | Moderate (predicted) | NHS | NHS actin remodeling regulator | Nucleus | other |
| MIMAT0000245 | miR-30c-5p (and other miRNAs w/seed GUAAACA) | 3.150 | ngenuity Expert Finding | Experimentally Observed | NID1 | nidogen 1 | Extracellular Space | other |
| MIMAT0000245 | miR-30c-5p (and other miRNAs w/seed GUAAACA) | 3.150 | ngenuity Expert Finding | Experimentally Observed | NID2 | nidogen 2 | Extracellular Space | other |
| MIMAT0000245 | miR-30c-5p (and other miRNAs w/seed GUAAACA) | 3.150 | TargetScan Human | Moderate (predicted) | NIF3L1 | NGG1 interacting factor 3 like 1 | Cytoplasm | other |
| MIMAT0000245 | miR-30c-5p (and other miRNAs w/seed GUAAACA) | 3.150 | TargetScan Human | Moderate (predicted) | NKAIN2 | ım/potassium transporting ATPase interac | Plasma Membrane | other |
| MIMAT0000245 | miR-30c-5p (and other miRNAs w/seed GUAAACA) | 3.150 | TargetScan Human | Moderate (predicted) | NKX2-2 | NK2 homeobox 2 | Nucleus | transcription regulator |
| MIMAT0000245 | miR-30c-5p (and other miRNAs w/seed GUAAACA) | 3.150 | TargetScan Human | Moderate (predicted) | NLGN1 | neuroligin 1 | Plasma Membrane | enzyme |
| MIMAT0000245 | miR-30c-5p (and other miRNAs w/seed GUAAACA) | 3.150 | TarBase | Experimentally Observed | NPR3 | natriuretic peptide receptor 3 | Plasma Membrane | G-protein coupled receptor |
| MIMAT0000245 | miR-30c-5p (and other miRNAs w/seed GUAAACA) | 3.150 | TargetScan Human | Moderate (predicted) | NR2F2 | clear receptor subfamily 2 group F membe | Nucleus | ligand-dependent nuclear receptor |
| MIMAT0000245 | miR-30c-5p (and other miRNAs w/seed GUAAACA) | 3.150 | TargetScan Human | High (predicted) | NR4A2 | clear receptor subfamily 4 group A membe | Nucleus | ligand-dependent nuclear receptor |
| MIMAT0000245 | miR-30c-5p (and other miRNAs w/seed GUAAACA) | 3.150 | TargetScan Human | Moderate (predicted) | NR5A2 | clear receptor subfamily 5 group A membe | Nucleus | ligand-dependent nuclear receptor |
| MIMAT0000245 | miR-30c-5p (and other miRNAs w/seed GUAAACA) | 3.150 | TargetScan Human | Moderate (predicted) | NR6A1 | clear receptor subfamily 6 group A membe | Nucleus | ligand-dependent nuclear receptor |
| MIMAT0000245 | miR-30c-5p (and other miRNAs w/seed GUAAACA) | 3.150 | TargetScan Human | Moderate (predicted) | NRBF2 | nuclear receptor binding factor 2 | Nucleus | transcription regulator |
| MIMAT0000245 | miR-30c-5p (and other miRNAs w/seed GUAAACA) | 3.150 | TargetScan Human | Moderate (predicted) | NRBP1 | nuclear receptor binding protein 1 | Nucleus | kinase |
| MIMAT0000245 | miR-30c-5p (and other miRNAs w/seed GUAAACA) | 3.150 | TargetScan Human | Moderate (predicted) | NRXN3 | neurexin 3 | Plasma Membrane | transporter |
| MIMAT0000245 | miR-30c-5p (and other miRNAs w/seed GUAAACA) | 3.150 | TargetScan Human | Moderate (predicted) | NSG1 | neuronal vesicle trafficking associated 1 | Cytoplasm | other |
| MIMAT0000245 | miR-30c-5p (and other miRNAs w/seed GUAAACA) | 3.150 | TarBase | Experimentally Observed | NT5C3A | 5'-nucleotidase, cytosolic IIIA | Cytoplasm | phosphatase |
| MIMAT0000245 | miR-30c-5p (and other miRNAs w/seed GUAAACA) | 3.150 | TargetScan Human | Moderate (predicted) | NT5DC3 | 5'-nucleotidase domain containing 3 | Cytoplasm | other |
| MIMAT0000245 | miR-30c-5p (and other miRNAs w/seed GUAAACA) | 3.150 | arBase,TargetScan Hum | ntally Observed,Moderate ( | NT5E | 5'-nucleotidase ecto | Plasma Membrane | phosphatase |
| MIMAT0000245 | miR-30c-5p (and other miRNAs w/seed GUAAACA) | 3.150 | TarBase | Experimentally Observed | NUCB1 | nucleobindin 1 | Cytoplasm | other |
| MIMAT0000245 | miR-30c-5p (and other miRNAs w/seed GUAAACA) | 3.150 | TarBase | Experimentally Observed | NUFIP2 | nuclear FMR1 interacting protein 2 | Cytoplasm | other |
| MIMAT0000245 | miR-30c-5p (and other miRNAs w/seed GUAAACA) | 3.150 | TargetScan Human | Moderate (predicted) | NUS1 | 1 dehydrodolichyl diphosphate synthase su | Cytoplasm | enzyme |
| MIMAT0000245 | miR-30c-5p (and other miRNAs w/seed GUAAACA) | 3.150 | TargetScan Human | High (predicted) | OMG | oligodendrocyte myelin glycoprotein | Plasma Membrane | G-protein coupled receptor |
| MIMAT0000245 | miR-30c-5p (and other miRNAs w/seed GUAAACA) | 3.150 | TargetScan Human | Moderate (predicted) | OR11A1 | ctory receptor family 11 subfamily A memb | Plasma Membrane | G-protein coupled receptor |
| MIMAT0000245 | miR-30c-5p (and other miRNAs w/seed GUAAACA) | 3.150 | TargetScan Human | Moderate (predicted) | OR12D3 | ctory receptor family 12 subfamily D memb | Plasma Membrane | G-protein coupled receptor |
| MIMAT0000245 | miR-30c-5p (and other miRNAs w/seed GUAAACA) | 3.150 | TargetScan Human | Moderate (predicted) | OR2B6 | ctory receptor family 2 subfamily B memb | Plasma Membrane | G-protein coupled receptor |
| MIMAT0000245 | miR-30c-5p (and other miRNAs w/seed GUAAACA) | 3.150 | TargetScan Human | Moderate (predicted) | ORC2 | origin recognition complex subunit 2 | Nucleus | other |
| MIMAT0000245 | miR-30c-5p (and other miRNAs w/seed GUAAACA) | 3.150 | TargetScan Human | Moderate (predicted) | OSTM1 | astogenesis associated transmembrane p | Cytoplasm | other |
| MIMAT0000245 | miR-30c-5p (and other miRNAs w/seed GUAAACA) | 3.150 | TargetScan Human | Moderate (predicted) | OTUD4 | OTU deubiquitinase 4 | Cytoplasm | peptidase |
| MIMAT0000245 | miR-30c-5p (and other miRNAs w/seed GUAAACA) | 3.150 | TargetScan Human | Moderate (predicted) | OVOL1 | ovo like transcriptional repressor 1 | Nucleus | transcription regulator |
| MIMAT0000245 | miR-30c-5p (and other miRNAs w/seed GUAAACA) | 3.150 | TargetScan Human | Moderate (predicted) | OXR1 | oxidation resistance 1 | Cytoplasm | enzyme |
| MIMAT0000245 | miR-30c-5p (and other miRNAs w/seed GUAAACA) | 3.150 | TargetScan Human | Moderate (predicted) | P2RY10 | P2Y receptor family member 10 | Plasma Membrane | G-protein coupled receptor |
| MIMAT0000245 | miR-30c-5p (and other miRNAs w/seed GUAAACA) | 3.150 | ngenuity Expert Finding | Experimentally Observed | P3H1 | prolyl 3-hydroxylase 1 | Nucleus | enzyme |
| MIMAT0000245 | miR-30c-5p (and other miRNAs w/seed GUAAACA) | 3.150 | TargetScan Human | Moderate (predicted) | P4HA1 | prolyl 4-hydroxylase subunit alpha 1 | Cytoplasm | enzyme |
| MIMAT0000245 | miR-30c-5p (and other miRNAs w/seed GUAAACA) | 3.150 | arBase,TargetScan Hum | ntally Observed,Moderate ( | P4HA2 | prolyl 4-hydroxylase subunit alpha 2 | Cytoplasm | transporter |
| MIMAT0000245 | miR-30c-5p (and other miRNAs w/seed GUAAACA) | 3.150 | TargetScan Human | High (predicted) | PAAF1 | proteasomal ATPase associated factor 1 | Other | other |
| MIMAT0000245 | miR-30c-5p (and other miRNAs w/seed GUAAACA) | 3.150 | TargetScan Human | Moderate (predicted) | PABPC1L2A | oly(A) binding protein cytoplasmic 1 like 2 | Cytoplasm | other |
| MIMAT0000245 | miR-30c-5p (and other miRNAs w/seed GUAAACA) | 3.150 | TargetScan Human | High (predicted) | PABPC1L2B | oly(A) binding protein cytoplasmic 1 like 2 | Cytoplasm | other |
| MIMAT0000245 | miR-30c-5p (and other miRNAs w/seed GUAAACA) | 3.150 | TarBase | Experimentally Observed | PAFAH1B2 | ctivating factor acetylhydrolase 1b catalytic | Cytoplasm | enzyme |
| MIMAT0000245 | miR-30c-5p (and other miRNAs w/seed GUAAACA) | 3.150 | TargetScan Human | Moderate (predicted) | PALM2AKAP2 | PALM2 and AKAP2 fusion | Plasma Membrane | other |
| MIMAT0000245 | miR-30c-5p (and other miRNAs w/seed GUAAACA) | 3.150 | TargetScan Human | Moderate (predicted) | PAPOLA | poly(A) polymerase alpha | Nucleus | enzyme |
| MIMAT0000245 | miR-30c-5p (and other miRNAs w/seed GUAAACA) | 3.150 | TargetScan Human | Moderate (predicted) | PAQR9 | ogestin and adipoQ receptor family membe | Plasma Membrane | other |
| MIMAT0000245 | miR-30c-5p (and other miRNAs w/seed GUAAACA) | 3.150 | TargetScan Human | Moderate (predicted) | PARP16 | y(ADP-ribose) polymerase family member | Cytoplasm | enzyme |
| MIMAT0000245 | miR-30c-5p (and other miRNAs w/seed GUAAACA) | 3.150 | TargetScan Human | Moderate (predicted) | PATL2 | PAT1 homolog 2 | Cytoplasm | translation regulator |
| MIMAT0000245 | miR-30c-5p (and other miRNAs w/seed GUAAACA) | 3.150 | TargetScan Human | Moderate (predicted) | PAWR | pro-apoptotic WT1 regulator | Nucleus | transcription regulator |
| MIMAT0000245 | miR-30c-5p (and other miRNAs w/seed GUAAACA) | 3.150 | TargetScan Human | Moderate (predicted) | PAX3 | paired box 3 | Nucleus | transcription regulator |
| MIMAT0000245 | miR-30c-5p (and other miRNAs w/seed GUAAACA) | 3.150 | TargetScan Human | High (predicted) | PBRM1 | polybromo 1 | Nucleus | other |
| MIMAT0000245 | miR-30c-5p (and other miRNAs w/seed GUAAACA) | 3.150 | TargetScan Human | Moderate (predicted) | PCDH17 | protocadherin 17 | Plasma Membrane | other |
| MIMAT0000245 | miR-30c-5p (and other miRNAs w/seed GUAAACA) | 3.150 | TargetScan Human | Moderate (predicted) | PCDH20 | protocadherin 20 | Other | other |
| MIMAT0000245 | miR-30c-5p (and other miRNAs w/seed GUAAACA) | 3.150 | TargetScan Human | High (predicted) | PCGF3 | polycomb group ring finger 3 | Nucleus | other |
| MIMAT0000245 | miR-30c-5p (and other miRNAs w/seed GUAAACA) | 3.150 | TargetScan Human | Moderate (predicted) | PCGF5 | polycomb group ring finger 5 | Cytoplasm | other |
| MIMAT0000245 | miR-30c-5p (and other miRNAs w/seed GUAAACA) | 3.150 | TargetScan Human | Moderate (predicted) | PCLAF | PCNA clamp associated factor | Nucleus | other |
| MIMAT0000245 | miR-30c-5p (and other miRNAs w/seed GUAAACA) | 3.150 | TargetScan Human | Moderate (predicted) | PCMTD2 | rtate (D-aspartate) O-methyltransferase dc | Cytoplasm | enzyme |
| MIMAT0000245 | miR-30c-5p (and other miRNAs w/seed GUAAACA) | 3.150 | TargetScan Human | Moderate (predicted) | PDAP1 | PDGFA associated protein 1 | Cytoplasm | other |
| MIMAT0000245 | miR-30c-5p (and other miRNAs w/seed GUAAACA) | 3.150 | TargetScan Human | Moderate (predicted) | PDCD10 | programmed cell death 10 | Cytoplasm | other |
| MIMAT0000245 | miR-30c-5p (and other miRNAs w/seed GUAAACA) | 3.150 | TargetScan Human | High (predicted) | PDCD2L | programmed cell death 2 like | Other | other |
| MIMAT0000245 | miR-30c-5p (and other miRNAs w/seed GUAAACA) | 3.150 | TargetScan Human | Moderate (predicted) | PDCL | phosducin like | Cytoplasm | enzyme |
| MIMAT0000245 | miR-30c-5p (and other miRNAs w/seed GUAAACA) | 3.150 | TargetScan Human | Moderate (predicted) | PDE4D | phosphodiesterase 4D | Cytoplasm | enzyme |
| MIMAT0000245 | miR-30c-5p (and other miRNAs w/seed GUAAACA) | 3.150 | TargetScan Human | Moderate (predicted) | PDE5A | phosphodiesterase 5A | Cytoplasm | enzyme |
| MIMAT0000245 | miR-30c-5p (and other miRNAs w/seed GUAAACA) | 3.150 | TargetScan Human | Moderate (predicted) | PDE6D | phosphodiesterase 6D | Cytoplasm | enzyme |
| MIMAT0000245 | miR-30c-5p (and other miRNAs w/seed GUAAACA) | 3.150 | TargetScan Human | High (predicted) | PDE7A | phosphodiesterase 7A | Cytoplasm | enzyme |
| MIMAT0000245 | miR-30c-5p (and other miRNAs w/seed GUAAACA) | 3.150 | ngenuity Expert Finding | Experimentally Observed | PDGFA | platelet derived growth factor subunit A | Extracellular Space | growth factor |
| MIMAT0000245 | miR-30c-5p (and other miRNAs w/seed GUAAACA) | 3.150 | ngenuity Expert Finding | Experimentally Observed | PDGFB | platelet derived growth factor subunit B | Extracellular Space | growth factor |
| MIMAT0000245 | miR-30c-5p (and other miRNAs w/seed GUAAACA) | 3.150 | TargetScan Human | Moderate (predicted) | PDSS1 | decaprenyl diphosphate synthase subunit | Cytoplasm | enzyme |
| MIMAT0000245 | miR-30c-5p (and other miRNAs w/seed GUAAACA) | 3.150 | TargetScan Human | Moderate (predicted) | PEG10 | paternally expressed 10 | Nucleus | other |
| MIMAT0000245 | miR-30c-5p (and other miRNAs w/seed GUAAACA) | 3.150 | TargetScan Human | Moderate (predicted) | PER2 | period circadian regulator 2 | Nucleus | transcription regulator |
| MIMAT0000245 | miR-30c-5p (and other miRNAs w/seed GUAAACA) | 3.150 | TargetScan Human | Moderate (predicted) | PET100 | PET100 cytochrome c oxidase chaperone | Other | other |
| MIMAT0000245 | miR-30c-5p (and other miRNAs w/seed GUAAACA) | 3.150 | TarBase | Experimentally Observed | PEX11B | peroxisomal biogenesis factor 11 beta | Cytoplasm | other |
| MIMAT0000245 | miR-30c-5p (and other miRNAs w/seed GUAAACA) | 3.150 | TargetScan Human | Moderate (predicted) | PEX5L | peroxisomal biogenesis factor 5 like | Cytoplasm | ion channel |
| MIMAT0000245 | miR-30c-5p (and other miRNAs w/seed GUAAACA) | 3.150 | TargetScan Human | High (predicted) | PFN2 | profilin 2 | Cytoplasm | enzyme |
| MIMAT0000245 | miR-30c-5p (and other miRNAs w/seed GUAAACA) | 3.150 | TargetScan Human | Moderate (predicted) | PGAP1 | GPI attachment to proteins inositol deacyl | Cytoplasm | enzyme |
| MIMAT0000245 | miR-30c-5p (and other miRNAs w/seed GUAAACA) | 3.150 | TargetScan Human | High (predicted) | PGBD2 | piggyBac transposable element derived 2 | Other | other |
| MIMAT0000245 | miR-30c-5p (and other miRNAs w/seed GUAAACA) | 3.150 | TargetScan Human | Moderate (predicted) | PGGT1B | in geranylgeranyltransferase type I subuni | Cytoplasm | enzyme |
| MIMAT0000245 | miR-30c-5p (and other miRNAs w/seed GUAAACA) | 3.150 | arBase,TargetScan Hum | nentally Observed,High (pr | PGM1 | phosphoglucomutase 1 | Cytoplasm | enzyme |
| MIMAT0000245 | miR-30c-5p (and other miRNAs w/seed GUAAACA) | 3.150 | TargetScan Human | Moderate (predicted) | PGM2L1 | phosphoglucomutase 2 like 1 | Cytoplasm | enzyme |
| MIMAT0000245 | miR-30c-5p (and other miRNAs w/seed GUAAACA) | 3.150 | TargetScan Human | High (predicted) | PGM3 | phosphoglucomutase 3 | Cytoplasm | enzyme |
| MIMAT0000245 | miR-30c-5p (and other miRNAs w/seed GUAAACA) | 3.150 | TargetScan Human | Moderate (predicted) | PGP | phosphoglycolate phosphatase | Cytoplasm | enzyme |
| MIMAT0000245 | miR-30c-5p (and other miRNAs w/seed GUAAACA) | 3.150 | TargetScan Human | Moderate (predicted) | PHACTR2 | phosphatase and actin regulator 2 | Other | other |
| MIMAT0000245 | miR-30c-5p (and other miRNAs w/seed GUAAACA) | 3.150 | TargetScan Human | Moderate (predicted) | PHETA1 | omain containing endocytic trafficking ada | Cytoplasm | other |
| MIMAT0000245 | miR-30c-5p (and other miRNAs w/seed GUAAACA) | 3.150 | TargetScan Human | Moderate (predicted) | PHETA2 | omain containing endocytic trafficking ada | Cytoplasm | other |
| MIMAT0000245 | miR-30c-5p (and other miRNAs w/seed GUAAACA) | 3.150 | TargetScan Human | Moderate (predicted) | PHIP | eckstrin homology domain interacting prot | Nucleus | other |
| MIMAT0000245 | miR-30c-5p (and other miRNAs w/seed GUAAACA) | 3.150 | TargetScan Human | Moderate (predicted) | PI4K2B | phosphatidylinositol 4-kinase type 2 beta | Cytoplasm | kinase |
| MIMAT0000245 | miR-30c-5p (and other miRNAs w/seed GUAAACA) | 3.150 | TargetScan Human | Moderate (predicted) | PICALM | phatidylinositol binding clathrin assembly p | Cytoplasm | other |
| MIMAT0000245 | miR-30c-5p (and other miRNAs w/seed GUAAACA) | 3.150 | TargetScan Human | Moderate (predicted) | PIGA | hatidylinositol glycan anchor biosynthesis | Cytoplasm | enzyme |
| MIMAT0000245 | miR-30c-5p (and other miRNAs w/seed GUAAACA) | 3.150 | TargetScan Human | Moderate (predicted) | PIGX | hatidylinositol glycan anchor biosynthesis | Cytoplasm | other |
| MIMAT0000245 | miR-30c-5p (and other miRNAs w/seed GUAAACA) | 3.150 | TargetScan Human | High (predicted) | PIP4K2A | hatidylinositol-5-phosphate 4-kinase type 2 | Cytoplasm | kinase |
| MIMAT0000245 | miR-30c-5p (and other miRNAs w/seed GUAAACA) | 3.150 | TargetScan Human | Moderate (predicted) | PIP4K2B | phatidylinositol-5-phosphate 4-kinase type | Cytoplasm | kinase |
| MIMAT0000245 | miR-30c-5p (and other miRNAs w/seed GUAAACA) | 3.150 | TargetScan Human | Moderate (predicted) | PITPNB | phosphatidylinositol transfer protein beta | Cytoplasm | transporter |
| MIMAT0000245 | miR-30c-5p (and other miRNAs w/seed GUAAACA) | 3.150 | TargetScan Human | Moderate (predicted) | PLA2G12A | phospholipase A2 group XIIA | Other | enzyme |
| MIMAT0000245 | miR-30c-5p (and other miRNAs w/seed GUAAACA) | 3.150 | TargetScan Human | Moderate (predicted) | PLA2G2C | phospholipase A2 group IIC | Extracellular Space | enzyme |
| MIMAT0000245 | miR-30c-5p (and other miRNAs w/seed GUAAACA) | 3.150 | TargetScan Human | High (predicted) | PLAGL2 | PLAG1 like zinc finger 2 | Nucleus | transcription regulator |
| MIMAT0000245 | miR-30c-5p (and other miRNAs w/seed GUAAACA) | 3.150 | ngenuity Expert Finding | Experimentally Observed | PLEC | plectin | Cytoplasm | other |
| MIMAT0000245 | miR-30c-5p (and other miRNAs w/seed GUAAACA) | 3.150 | TargetScan Human | Moderate (predicted) | PLEKHA8 | pleckstrin homology domain containing A8 | Extracellular Space | transporter |
| MIMAT0000245 | miR-30c-5p (and other miRNAs w/seed GUAAACA) | 3.150 | TargetScan Human | Moderate (predicted) | PLEKHO2 | pleckstrin homology domain containing O2 | Cytoplasm | other |
| MIMAT0000245 | miR-30c-5p (and other miRNAs w/seed GUAAACA) | 3.150 | TargetScan Human | Moderate (predicted) | PLIN3 | perilipin 3 | Cytoplasm | other |
| MIMAT0000245 | miR-30c-5p (and other miRNAs w/seed GUAAACA) | 3.150 | ngenuity Expert Finding | Experimentally Observed | PLOD2 | collagen-lysine,2-oxoglutarate 5-dioxygena | Cytoplasm | enzyme |
| MIMAT0000245 | miR-30c-5p (and other miRNAs w/seed GUAAACA) | 3.150 | TargetScan Human | Moderate (predicted) | PLPP6 | phospholipid phosphatase 6 | Plasma Membrane | phosphatase |
| MIMAT0000245 | miR-30c-5p (and other miRNAs w/seed GUAAACA) | 3.150 | TargetScan Human | Moderate (predicted) | PLPPR4 | phospholipid phosphatase related 4 | Plasma Membrane | phosphatase |
| MIMAT0000245 | miR-30c-5p (and other miRNAs w/seed GUAAACA) | 3.150 | TargetScan Human | Moderate (predicted) | PNMA1 | PNMA family member 1 | Nucleus | other |
| MIMAT0000245 | miR-30c-5p (and other miRNAs w/seed GUAAACA) | 3.150 | TarBase | Experimentally Observed | PNP | purine nucleoside phosphorylase | Nucleus | enzyme |
| MIMAT0000245 | miR-30c-5p (and other miRNAs w/seed GUAAACA) | 3.150 | TargetScan Human | Moderate (predicted) | PNPLA1 | tatin like phospholipase domain containin | Cytoplasm | enzyme |
| MIMAT0000245 | miR-30c-5p (and other miRNAs w/seed GUAAACA) | 3.150 | TargetScan Human | Moderate (predicted) | POC1B-GALNT4 | POC1B-GALNT4 readthrough | Other | other |

Sup. Table 3. List of 3699 targets of the seven dysregulated miRNAs obtained from three different target prediction databases,TargetScan TarBase and miRecords considering those predicted as moderate, high, or experimentally validated, respectively.

| | | | | | | | | |
|---|---|---|---|---|---|---|---|---|
| MIMAT0000245 | miR-30c-5p (and other miRNAs w/seed GUAAACA) | 3.150 | TarBase | Experimentally Observed | POGLUT3 | protein O-glucosyltransferase 3 | Other | enzyme |
| MIMAT0000245 | miR-30c-5p (and other miRNAs w/seed GUAAACA) | 3.150 | TargetScan Human | Moderate (predicted) | POLE3 | NA polymerase epsilon 3, accessory subu | Nucleus | enzyme |
| MIMAT0000245 | miR-30c-5p (and other miRNAs w/seed GUAAACA) | 3.150 | TargetScan Human | Moderate (predicted) | POLR1F | RNA polymerase I subunit F | Nucleus | other |
| MIMAT0000245 | miR-30c-5p (and other miRNAs w/seed GUAAACA) | 3.150 | TargetScan Human | Moderate (predicted) | POLR3G | RNA polymerase III subunit G | Nucleus | enzyme |
| MIMAT0000245 | miR-30c-5p (and other miRNAs w/seed GUAAACA) | 3.150 | TargetScan Human | Moderate (predicted) | PON2 | paraoxonase 2 | Plasma Membrane | enzyme |
| MIMAT0000245 | miR-30c-5p (and other miRNAs w/seed GUAAACA) | 3.150 | TargetScan Human | Moderate (predicted) | POP1 | OP1 homolog, ribonuclease P/MRP subui | Nucleus | enzyme |
| MIMAT0000245 | miR-30c-5p (and other miRNAs w/seed GUAAACA) | 3.150 | TargetScan Human | High (predicted) | PPARGC1A | PPARG coactivator 1 alpha | Nucleus | transcription regulator |
| MIMAT0000245 | miR-30c-5p (and other miRNAs w/seed GUAAACA) | 3.150 | TargetScan Human | High (predicted) | PPARGC1B | PPARG coactivator 1 beta | Nucleus | transcription regulator |
| MIMAT0000245 | miR-30c-5p (and other miRNAs w/seed GUAAACA) | 3.150 | TargetScan Human | High (predicted) | PPID | peptidylprolyl isomerase D | Cytoplasm | enzyme |
| MIMAT0000245 | miR-30c-5p (and other miRNAs w/seed GUAAACA) | 3.150 | TargetScan Human | High (predicted) | PPIL3 | peptidylprolyl isomerase like 3 | Cytoplasm | enzyme |
| MIMAT0000245 | miR-30c-5p (and other miRNAs w/seed GUAAACA) | 3.150 | TargetScan Human | Moderate (predicted) | PPP1R14C | n phosphatase 1 regulatory inhibitor subur | Cytoplasm | other |
| MIMAT0000245 | miR-30c-5p (and other miRNAs w/seed GUAAACA) | 3.150 | TargetScan Human | High (predicted) | PPP1R18 | rotein phosphatase 1 regulatory subunit 1 | Other | other |
| MIMAT0000245 | miR-30c-5p (and other miRNAs w/seed GUAAACA) | 3.150 | TargetScan Human | Moderate (predicted) | PPP1R1C | n phosphatase 1 regulatory inhibitor subu | Cytoplasm | phosphatase |
| MIMAT0000245 | miR-30c-5p (and other miRNAs w/seed GUAAACA) | 3.150 | TargetScan Human | High (predicted) | PPP1R2 | ein phosphatase 1 regulatory inhibitor subu | Cytoplasm | phosphatase |
| MIMAT0000245 | miR-30c-5p (and other miRNAs w/seed GUAAACA) | 3.150 | arBase,TargetScan Hum | ntally Observed,Moderate ( | PPP3CA | rotein phosphatase 3 catalytic subunit alpl | Cytoplasm | phosphatase |
| MIMAT0000245 | miR-30c-5p (and other miRNAs w/seed GUAAACA) | 3.150 | TargetScan Human | Moderate (predicted) | PPP3CB | rotein phosphatase 3 catalytic subunit bet | Plasma Membrane | phosphatase |
| MIMAT0000245 | miR-30c-5p (and other miRNAs w/seed GUAAACA) | 3.150 | TarBase | Experimentally Observed | PPP3R1 | ein phosphatase 3 regulatory subunit B, a | Cytoplasm | phosphatase |
| MIMAT0000245 | miR-30c-5p (and other miRNAs w/seed GUAAACA) | 3.150 | TargetScan Human | Moderate (predicted) | PPTC7 | protein phosphatase targeting COQ7 | Cytoplasm | phosphatase |
| MIMAT0000245 | miR-30c-5p (and other miRNAs w/seed GUAAACA) | 3.150 | TargetScan Human | High (predicted) | PRDM1 | PR/SET domain 1 | Nucleus | transcription regulator |
| MIMAT0000245 | miR-30c-5p (and other miRNAs w/seed GUAAACA) | 3.150 | TargetScan Human | Moderate (predicted) | PRDM13 | PR/SET domain 13 | Nucleus | enzyme |
| MIMAT0000245 | miR-30c-5p (and other miRNAs w/seed GUAAACA) | 3.150 | TargetScan Human | Moderate (predicted) | PRG4 | proteoglycan 4 | Extracellular Space | other |
| MIMAT0000245 | miR-30c-5p (and other miRNAs w/seed GUAAACA) | 3.150 | TargetScan Human | Moderate (predicted) | PRLR | prolactin receptor | Plasma Membrane | transmembrane receptor |
| MIMAT0000245 | miR-30c-5p (and other miRNAs w/seed GUAAACA) | 3.150 | TargetScan Human | Moderate (predicted) | PROX1 | prospero homeobox 1 | Nucleus | transcription regulator |
| MIMAT0000245 | miR-30c-5p (and other miRNAs w/seed GUAAACA) | 3.150 | arBase,TargetScan Hum | nentally Observed,High (pr | PRPF40A | pre-mRNA processing factor 40 homolog / | Nucleus | other |
| MIMAT0000245 | miR-30c-5p (and other miRNAs w/seed GUAAACA) | 3.150 | TargetScan Human | Moderate (predicted) | PRRG1 | proline rich and Gla domain 1 | Plasma Membrane | other |
| MIMAT0000245 | miR-30c-5p (and other miRNAs w/seed GUAAACA) | 3.150 | TargetScan Human | Moderate (predicted) | PRRX1 | paired related homeobox 1 | Nucleus | transcription regulator |
| MIMAT0000245 | miR-30c-5p (and other miRNAs w/seed GUAAACA) | 3.150 | TargetScan Human | Moderate (predicted) | PSEN2 | presenilin 2 | Cytoplasm | peptidase |
| MIMAT0000245 | miR-30c-5p (and other miRNAs w/seed GUAAACA) | 3.150 | TargetScan Human | Moderate (predicted) | PSMD7 | proteasome 26S subunit, non-ATPase 7 | Cytoplasm | other |
| MIMAT0000245 | miR-30c-5p (and other miRNAs w/seed GUAAACA) | 3.150 | TargetScan Human | High (predicted) | PSTK | phosphoseryl-tRNA kinase | Cytoplasm | kinase |
| MIMAT0000245 | miR-30c-5p (and other miRNAs w/seed GUAAACA) | 3.150 | TargetScan Human | Moderate (predicted) | PSTPIP2 | serine-threonine phosphatase interacting | Cytoplasm | other |
| MIMAT0000245 | miR-30c-5p (and other miRNAs w/seed GUAAACA) | 3.150 | TargetScan Human | Moderate (predicted) | PTAR1 | prenyltransferase alpha subunit repeat con | Other | other |
| MIMAT0000245 | miR-30c-5p (and other miRNAs w/seed GUAAACA) | 3.150 | TargetScan Human | Moderate (predicted) | PTGER3 | prostaglandin E receptor 3 | Plasma Membrane | G-protein coupled receptor |
| MIMAT0000245 | miR-30c-5p (and other miRNAs w/seed GUAAACA) | 3.150 | arBase,TargetScan Hum | ntally Observed,Moderate ( | PTGFRN | prostaglandin F2 receptor inhibitor | Plasma Membrane | other |
| MIMAT0000245 | miR-30c-5p (and other miRNAs w/seed GUAAACA) | 3.150 | TargetScan Human | High (predicted) | PTP4A1 | protein tyrosine phosphatase 4A1 | Cytoplasm | phosphatase |
| MIMAT0000245 | miR-30c-5p (and other miRNAs w/seed GUAAACA) | 3.150 | TarBase | Experimentally Observed | PTPA | rotein phosphatase 2 phosphatase activat | Cytoplasm | phosphatase |
| MIMAT0000245 | miR-30c-5p (and other miRNAs w/seed GUAAACA) | 3.150 | TargetScan Human | High (predicted) | PTPN13 | ein tyrosine phosphatase non-receptor typ | Cytoplasm | phosphatase |
| MIMAT0000245 | miR-30c-5p (and other miRNAs w/seed GUAAACA) | 3.150 | TargetScan Human | High (predicted) | PTPN20 | ein tyrosine phosphatase non-receptor typ | Extracellular Space | phosphatase |
| MIMAT0000245 | miR-30c-5p (and other miRNAs w/seed GUAAACA) | 3.150 | TarBase | Experimentally Observed | PTPRK | rotein tyrosine phosphatase receptor type | Plasma Membrane | phosphatase |
| MIMAT0000245 | miR-30c-5p (and other miRNAs w/seed GUAAACA) | 3.150 | TarBase | Experimentally Observed | PTRH1 | peptidyl-tRNA hydrolase 1 homolog | Cytoplasm | enzyme |
| MIMAT0000245 | miR-30c-5p (and other miRNAs w/seed GUAAACA) | 3.150 | TargetScan Human | Moderate (predicted) | QKI | QKI, KH domain containing RNA binding | Nucleus | other |
| MIMAT0000245 | miR-30c-5p (and other miRNAs w/seed GUAAACA) | 3.150 | TargetScan Human | Moderate (predicted) | QRFPR | pyroglutamylated RFamide peptide recept | Plasma Membrane | G-protein coupled receptor |
| MIMAT0000245 | miR-30c-5p (and other miRNAs w/seed GUAAACA) | 3.150 | TargetScan Human | High (predicted) | R3HDM1 | R3H domain containing 1 | Other | other |
| MIMAT0000245 | miR-30c-5p (and other miRNAs w/seed GUAAACA) | 3.150 | TargetScan Human | Moderate (predicted) | RAB15 | RAB15, member RAS oncogene family | Cytoplasm | other |
| MIMAT0000245 | miR-30c-5p (and other miRNAs w/seed GUAAACA) | 3.150 | TargetScan Human | Moderate (predicted) | RAB22A | RAB22A, member RAS oncogene family | Cytoplasm | enzyme |
| MIMAT0000245 | miR-30c-5p (and other miRNAs w/seed GUAAACA) | 3.150 | TargetScan Human | Moderate (predicted) | RAB23 | RAB23, member RAS oncogene family | Cytoplasm | enzyme |
| MIMAT0000245 | miR-30c-5p (and other miRNAs w/seed GUAAACA) | 3.150 | arBase,TargetScan Hum | nentally Observed,High (pr | RAB27B | RAB27B, member RAS oncogene family | Cytoplasm | enzyme |
| MIMAT0000245 | miR-30c-5p (and other miRNAs w/seed GUAAACA) | 3.150 | TargetScan Human | High (predicted) | RAB32 | RAB32, member RAS oncogene family | Cytoplasm | enzyme |
| MIMAT0000245 | miR-30c-5p (and other miRNAs w/seed GUAAACA) | 3.150 | TargetScan Human | High (predicted) | RAB38 | RAB38, member RAS oncogene family | Cytoplasm | enzyme |
| MIMAT0000245 | miR-30c-5p (and other miRNAs w/seed GUAAACA) | 3.150 | TargetScan Human | Moderate (predicted) | RAB3D | RAB3D, member RAS oncogene family | Cytoplasm | enzyme |
| MIMAT0000245 | miR-30c-5p (and other miRNAs w/seed GUAAACA) | 3.150 | TargetScan Human | Moderate (predicted) | RAB41 | RAB41, member RAS oncogene family | Cytoplasm | other |
| MIMAT0000245 | miR-30c-5p (and other miRNAs w/seed GUAAACA) | 3.150 | TargetScan Human | Moderate (predicted) | RAB4B | RAB4B, member RAS oncogene family | Plasma Membrane | enzyme |
| MIMAT0000245 | miR-30c-5p (and other miRNAs w/seed GUAAACA) | 3.150 | TargetScan Human | Moderate (predicted) | RAB7A | RAB7A, member RAS oncogene family | Cytoplasm | enzyme |
| MIMAT0000245 | miR-30c-5p (and other miRNAs w/seed GUAAACA) | 3.150 | TargetScan Human | Moderate (predicted) | RAB8A | RAB8A, member RAS oncogene family | Plasma Membrane | enzyme |
| MIMAT0000245 | miR-30c-5p (and other miRNAs w/seed GUAAACA) | 3.150 | TargetScan Human | Moderate (predicted) | RABGAP1L | RAB GTPase activating protein 1 like | Cytoplasm | other |
| MIMAT0000245 | miR-30c-5p (and other miRNAs w/seed GUAAACA) | 3.150 | arBase,TargetScan Hum | nentally Observed,High (pr | RAD23B | 23 homolog B, nucleotide excision repair p | Nucleus | other |
| MIMAT0000245 | miR-30c-5p (and other miRNAs w/seed GUAAACA) | 3.150 | TargetScan Human | Moderate (predicted) | RAD54B | RAD54 homolog B | Nucleus | enzyme |
| MIMAT0000245 | miR-30c-5p (and other miRNAs w/seed GUAAACA) | 3.150 | TargetScan Human | Moderate (predicted) | RALGDS | l guanine nucleotide dissociation stimulat | Cytoplasm | other |
| MIMAT0000245 | miR-30c-5p (and other miRNAs w/seed GUAAACA) | 3.150 | TargetScan Human | Moderate (predicted) | RANBP9 | RAN binding protein 9 | Nucleus | other |
| MIMAT0000245 | miR-30c-5p (and other miRNAs w/seed GUAAACA) | 3.150 | TargetScan Human | High (predicted) | RAP1B | RAP1B, member of RAS oncogene family | Cytoplasm | enzyme |
| MIMAT0000245 | miR-30c-5p (and other miRNAs w/seed GUAAACA) | 3.150 | TargetScan Human | Moderate (predicted) | RAP2B | RAP2B, member of RAS oncogene family | Plasma Membrane | enzyme |
| MIMAT0000245 | miR-30c-5p (and other miRNAs w/seed GUAAACA) | 3.150 | TargetScan Human | Moderate (predicted) | RAP2C | RAP2C, member of RAS oncogene family | Cytoplasm | enzyme |
| MIMAT0000245 | miR-30c-5p (and other miRNAs w/seed GUAAACA) | 3.150 | TargetScan Human | Moderate (predicted) | RAPGEF4 | Rap guanine nucleotide exchange factor 4 | Cytoplasm | other |
| MIMAT0000245 | miR-30c-5p (and other miRNAs w/seed GUAAACA) | 3.150 | TargetScan Human | High (predicted) | RARB | retinoic acid receptor beta | Nucleus | ligand-dependent nuclear receptor |
| MIMAT0000245 | miR-30c-5p (and other miRNAs w/seed GUAAACA) | 3.150 | TargetScan Human | High (predicted) | RARG | retinoic acid receptor gamma | Nucleus | ligand-dependent nuclear receptor |
| MIMAT0000245 | miR-30c-5p (and other miRNAs w/seed GUAAACA) | 3.150 | TargetScan Human | High (predicted) | RARRES1 | retinoic acid receptor responder 1 | Plasma Membrane | other |
| MIMAT0000245 | miR-30c-5p (and other miRNAs w/seed GUAAACA) | 3.150 | TargetScan Human | High (predicted) | RASA1 | RAS p21 protein activator 1 | Cytoplasm | transporter |
| MIMAT0000245 | miR-30c-5p (and other miRNAs w/seed GUAAACA) | 3.150 | TargetScan Human | Moderate (predicted) | RASAL2 | RAS protein activator like 2 | Extracellular Space | other |
| MIMAT0000245 | miR-30c-5p (and other miRNAs w/seed GUAAACA) | 3.150 | TargetScan Human | Moderate (predicted) | RASEF | RAS and EF-hand domain containing | Cytoplasm | other |
| MIMAT0000245 | miR-30c-5p (and other miRNAs w/seed GUAAACA) | 3.150 | TargetScan Human | Moderate (predicted) | RASSF4 | Ras association domain family member 4 | Other | other |
| MIMAT0000245 | miR-30c-5p (and other miRNAs w/seed GUAAACA) | 3.150 | TargetScan Human | Moderate (predicted) | RAVER2 | ribonucleoprotein, PTB binding 2 | Nucleus | other |
| MIMAT0000245 | miR-30c-5p (and other miRNAs w/seed GUAAACA) | 3.150 | TarBase | Experimentally Observed | RBMS1 | binding motif single stranded interacting pr | Nucleus | other |
| MIMAT0000245 | miR-30c-5p (and other miRNAs w/seed GUAAACA) | 3.150 | TargetScan Human | Moderate (predicted) | RCBTB1 | RCC1 and BTB domain containing protein | Nucleus | other |
| MIMAT0000245 | miR-30c-5p (and other miRNAs w/seed GUAAACA) | 3.150 | TargetScan Human | Moderate (predicted) | RECQL | RecQ like helicase | Nucleus | enzyme |
| MIMAT0000245 | miR-30c-5p (and other miRNAs w/seed GUAAACA) | 3.150 | TargetScan Human | Moderate (predicted) | REEP1 | receptor accessory protein 1 | Cytoplasm | other |
| MIMAT0000245 | miR-30c-5p (and other miRNAs w/seed GUAAACA) | 3.150 | TargetScan Human | High (predicted) | REEP3 | receptor accessory protein 3 | Other | other |
| MIMAT0000245 | miR-30c-5p (and other miRNAs w/seed GUAAACA) | 3.150 | TargetScan Human | High (predicted) | REV1 | REV1 DNA directed polymerase | Nucleus | enzyme |
| MIMAT0000245 | miR-30c-5p (and other miRNAs w/seed GUAAACA) | 3.150 | TargetScan Human | Moderate (predicted) | RFTN2 | raftlin family member 2 | Other | other |
| MIMAT0000245 | miR-30c-5p (and other miRNAs w/seed GUAAACA) | 3.150 | TargetScan Human | Moderate (predicted) | RFX2 | regulatory factor X2 | Nucleus | transcription regulator |
| MIMAT0000245 | miR-30c-5p (and other miRNAs w/seed GUAAACA) | 3.150 | TargetScan Human | High (predicted) | RFX6 | regulatory factor X6 | Nucleus | enzyme |
| MIMAT0000245 | miR-30c-5p (and other miRNAs w/seed GUAAACA) | 3.150 | TargetScan Human | Moderate (predicted) | RFX7 | regulatory factor X7 | Nucleus | transcription regulator |
| MIMAT0000245 | miR-30c-5p (and other miRNAs w/seed GUAAACA) | 3.150 | TargetScan Human | High (predicted) | GPD4 (includes other | RANBP2 like and GRIP domain containing | Nucleus | enzyme |
| MIMAT0000245 | miR-30c-5p (and other miRNAs w/seed GUAAACA) | 3.150 | TargetScan Human | Moderate (predicted) | RGS1 | regulator of G protein signaling 1 | Plasma Membrane | enzyme |
| MIMAT0000245 | miR-30c-5p (and other miRNAs w/seed GUAAACA) | 3.150 | TargetScan Human | Moderate (predicted) | RGS17 | regulator of G protein signaling 17 | Cytoplasm | enzyme |
| MIMAT0000245 | miR-30c-5p (and other miRNAs w/seed GUAAACA) | 3.150 | TargetScan Human | Moderate (predicted) | RGS22 | regulator of G protein signaling 22 | Cytoplasm | other |
| MIMAT0000245 | miR-30c-5p (and other miRNAs w/seed GUAAACA) | 3.150 | TargetScan Human | High (predicted) | RHEBL1 | RHEB like 1 | Cytoplasm | enzyme |
| MIMAT0000245 | miR-30c-5p (and other miRNAs w/seed GUAAACA) | 3.150 | TargetScan Human | Moderate (predicted) | RLIM | ring finger protein, LIM domain interacting | Nucleus | enzyme |
| MIMAT0000245 | miR-30c-5p (and other miRNAs w/seed GUAAACA) | 3.150 | TargetScan Human | Moderate (predicted) | RNF122 | ring finger protein 122 | Cytoplasm | enzyme |
| MIMAT0000245 | miR-30c-5p (and other miRNAs w/seed GUAAACA) | 3.150 | TargetScan Human | Moderate (predicted) | RNF138 | ring finger protein 138 | Nucleus | enzyme |
| MIMAT0000245 | miR-30c-5p (and other miRNAs w/seed GUAAACA) | 3.150 | TargetScan Human | Moderate (predicted) | RNF146 | ring finger protein 146 | Cytoplasm | enzyme |
| MIMAT0000245 | miR-30c-5p (and other miRNAs w/seed GUAAACA) | 3.150 | TargetScan Human | Moderate (predicted) | RNF157 | ring finger protein 157 | Cytoplasm | enzyme |
| MIMAT0000245 | miR-30c-5p (and other miRNAs w/seed GUAAACA) | 3.150 | TargetScan Human | Moderate (predicted) | RNF220 | ring finger protein 220 | Cytoplasm | enzyme |
| MIMAT0000245 | miR-30c-5p (and other miRNAs w/seed GUAAACA) | 3.150 | TargetScan Human | Moderate (predicted) | RNF34 | ring finger protein 34 | Cytoplasm | enzyme |
| MIMAT0000245 | miR-30c-5p (and other miRNAs w/seed GUAAACA) | 3.150 | TargetScan Human | Moderate (predicted) | ROR1 | ceptor tyrosine kinase like orphan recepto | Plasma Membrane | kinase |
| MIMAT0000245 | miR-30c-5p (and other miRNAs w/seed GUAAACA) | 3.150 | TargetScan Human | High (predicted) | RORA | RAR related orphan receptor A | Nucleus | ligand-dependent nuclear receptor |
| MIMAT0000245 | miR-30c-5p (and other miRNAs w/seed GUAAACA) | 3.150 | TargetScan Human | High (predicted) | RP11 160N110 | | Other | other |
| MIMAT0000245 | miR-30c-5p (and other miRNAs w/seed GUAAACA) | 3.150 | TargetScan Human | High (predicted) | RP11 497E192 | | Other | other |
| MIMAT0000245 | miR-30c-5p (and other miRNAs w/seed GUAAACA) | 3.150 | TargetScan Human | High (predicted) | RP11 674E164 | | Other | other |
| MIMAT0000245 | miR-30c-5p (and other miRNAs w/seed GUAAACA) | 3.150 | TargetScan Human | High (predicted) | RP11 738G52 | | Other | other |
| MIMAT0000245 | miR-30c-5p (and other miRNAs w/seed GUAAACA) | 3.150 | TargetScan Human | Moderate (predicted) | RPA2 | replication protein A2 | Nucleus | other |
| MIMAT0000245 | miR-30c-5p (and other miRNAs w/seed GUAAACA) | 3.150 | TargetScan Human | Moderate (predicted) | RPL10L | ribosomal protein L10 like | Nucleus | other |
| MIMAT0000245 | miR-30c-5p (and other miRNAs w/seed GUAAACA) | 3.150 | TargetScan Human | Moderate (predicted) | RPL17 | ribosomal protein L17 | Cytoplasm | other |
| MIMAT0000245 | miR-30c-5p (and other miRNAs w/seed GUAAACA) | 3.150 | TargetScan Human | Moderate (predicted) | RPL37 | ribosomal protein L37 | Cytoplasm | other |
| MIMAT0000245 | miR-30c-5p (and other miRNAs w/seed GUAAACA) | 3.150 | TargetScan Human | Moderate (predicted) | RPL7 | ribosomal protein L7 | Nucleus | transcription regulator |
| MIMAT0000245 | miR-30c-5p (and other miRNAs w/seed GUAAACA) | 3.150 | TargetScan Human | Moderate (predicted) | RPP40 | ribonuclease P/MRP subunit p40 | Nucleus | enzyme |
| MIMAT0000245 | miR-30c-5p (and other miRNAs w/seed GUAAACA) | 3.150 | TargetScan Human | Moderate (predicted) | RPRD1A | ation of nuclear pre-mRNA domain contain | Extracellular Space | other |
| MIMAT0000245 | miR-30c-5p (and other miRNAs w/seed GUAAACA) | 3.150 | TargetScan Human | High (predicted) | RRAD | elated glycolysis inhibitor and calcium cha | Cytoplasm | enzyme |
| MIMAT0000245 | miR-30c-5p (and other miRNAs w/seed GUAAACA) | 3.150 | TargetScan Human | Moderate (predicted) | RRP1B | ribosomal RNA processing 1B | Nucleus | transcription regulator |
| MIMAT0000245 | miR-30c-5p (and other miRNAs w/seed GUAAACA) | 3.150 | TargetScan Human | Moderate (predicted) | RTCB | RNA 2',3'-cyclic phosphate and 5'-OH ligas | Cytoplasm | enzyme |
| MIMAT0000245 | miR-30c-5p (and other miRNAs w/seed GUAAACA) | 3.150 | TargetScan Human | Moderate (predicted) | RTKN2 | rhotekin 2 | Plasma Membrane | other |
| MIMAT0000245 | miR-30c-5p (and other miRNAs w/seed GUAAACA) | 3.150 | TargetScan Human | Moderate (predicted) | RTN4R | reticulon 4 receptor | Plasma Membrane | transmembrane receptor |
| MIMAT0000245 | miR-30c-5p (and other miRNAs w/seed GUAAACA) | 3.150 | TargetScan Human | Moderate (predicted) | RUBCNL | rubicon like autophagy enhancer | Other | other |
| MIMAT0000245 | miR-30c-5p (and other miRNAs w/seed GUAAACA) | 3.150 | TargetScan Human | Moderate (predicted) | RUNDC3B | RUN domain containing 3B | Other | other |
| MIMAT0000245 | miR-30c-5p (and other miRNAs w/seed GUAAACA) | 3.150 | Expert Findings,TargetSc | nentally Observed,High (pr | RUNX2 | RUNX family transcription factor 2 | Nucleus | transcription regulator |
| MIMAT0000245 | miR-30c-5p (and other miRNAs w/seed GUAAACA) | 3.150 | TargetScan Human | Moderate (predicted) | RWDD4 | RWD domain containing 4 | Other | other |
| MIMAT0000245 | miR-30c-5p (and other miRNAs w/seed GUAAACA) | 3.150 | TargetScan Human | High (predicted) | S100PBP | S100P binding protein | Nucleus | other |
| MIMAT0000245 | miR-30c-5p (and other miRNAs w/seed GUAAACA) | 3.150 | TargetScan Human | Moderate (predicted) | SALL4 | spalt like transcription factor 4 | Nucleus | transcription regulator |
| MIMAT0000245 | miR-30c-5p (and other miRNAs w/seed GUAAACA) | 3.150 | TargetScan Human | Moderate (predicted) | SAMHD1 | containing deoxynucleoside triphosphate | Nucleus | enzyme |
| MIMAT0000245 | miR-30c-5p (and other miRNAs w/seed GUAAACA) | 3.150 | TargetScan Human | Moderate (predicted) | SAP30 | Sin3A associated protein 30 | Nucleus | transcription regulator |
| MIMAT0000245 | miR-30c-5p (and other miRNAs w/seed GUAAACA) | 3.150 | TargetScan Human | Moderate (predicted) | SCAF4 | SR-related CTD associated factor 4 | Nucleus | other |
| MIMAT0000245 | miR-30c-5p (and other miRNAs w/seed GUAAACA) | 3.150 | TargetScan Human | Moderate (predicted) | SCAMP1 | secretory carrier membrane protein 1 | Cytoplasm | transporter |
| MIMAT0000245 | miR-30c-5p (and other miRNAs w/seed GUAAACA) | 3.150 | TargetScan Human | Moderate (predicted) | SCEL | sciellin | Cytoplasm | other |
| MIMAT0000245 | miR-30c-5p (and other miRNAs w/seed GUAAACA) | 3.150 | TargetScan Human | High (predicted) | SCML1 | Scm polycomb group protein like 1 | Nucleus | transcription regulator |
| MIMAT0000245 | miR-30c-5p (and other miRNAs w/seed GUAAACA) | 3.150 | TargetScan Human | Moderate (predicted) | SCN1A | odium voltage-gated channel alpha subuni | Plasma Membrane | ion channel |
| MIMAT0000245 | miR-30c-5p (and other miRNAs w/seed GUAAACA) | 3.150 | TargetScan Human | High (predicted) | SCN2A | odium voltage-gated channel alpha subuni | Plasma Membrane | ion channel |
| MIMAT0000245 | miR-30c-5p (and other miRNAs w/seed GUAAACA) | 3.150 | TargetScan Human | Moderate (predicted) | SCN3A | odium voltage-gated channel alpha subuni | Plasma Membrane | ion channel |
| MIMAT0000245 | miR-30c-5p (and other miRNAs w/seed GUAAACA) | 3.150 | TargetScan Human | High (predicted) | SCN9A | odium voltage-gated channel alpha subuni | Plasma Membrane | ion channel |
| MIMAT0000245 | miR-30c-5p (and other miRNAs w/seed GUAAACA) | 3.150 | TargetScan Human | Moderate (predicted) | SCYL3 | SCY1 like pseudokinase 3 | Cytoplasm | kinase |
| MIMAT0000245 | miR-30c-5p (and other miRNAs w/seed GUAAACA) | 3.150 | TargetScan Human | High (predicted) | SDAD1 | SDA1 domain containing 1 | Nucleus | other |
| MIMAT0000245 | miR-30c-5p (and other miRNAs w/seed GUAAACA) | 3.150 | TargetScan Human | Moderate (predicted) | SEC22C | EC22 homolog C, vesicle trafficking prote | Cytoplasm | other |
| MIMAT0000245 | miR-30c-5p (and other miRNAs w/seed GUAAACA) | 3.150 | arBase,TargetScan Hum | nentally Observed,High (pr | SEC23A | 23 homolog A, COPII coat complex compo | Cytoplasm | transporter |
| MIMAT0000245 | miR-30c-5p (and other miRNAs w/seed GUAAACA) | 3.150 | TargetScan Human | Moderate (predicted) | SEC61A2 | SEC61 translocon subunit alpha 2 | Cytoplasm | transporter |
| MIMAT0000245 | miR-30c-5p (and other miRNAs w/seed GUAAACA) | 3.150 | TarBase | Experimentally Observed | SEC62 | C62 homolog, preprotein translocation fac | Cytoplasm | transporter |
| MIMAT0000245 | miR-30c-5p (and other miRNAs w/seed GUAAACA) | 3.150 | TargetScan Human | Moderate (predicted) | SELENOS | selenoprotein S | Cytoplasm | other |
| MIMAT0000245 | miR-30c-5p (and other miRNAs w/seed GUAAACA) | 3.150 | TargetScan Human | Moderate (predicted) | SEMA3A | semaphorin 3A | Extracellular Space | other |
| MIMAT0000245 | miR-30c-5p (and other miRNAs w/seed GUAAACA) | 3.150 | TargetScan Human | Moderate (predicted) | SEMA6B | semaphorin 6B | Plasma Membrane | other |
| MIMAT0000245 | miR-30c-5p (and other miRNAs w/seed GUAAACA) | 3.150 | TargetScan Human | Moderate (predicted) | SEPTIN7 | septin 7 | Cytoplasm | other |
| MIMAT0000245 | miR-30c-5p (and other miRNAs w/seed GUAAACA) | 3.150 | TargetScan Human | Moderate (predicted) | SERINC3 | serine incorporator 3 | Cytoplasm | transporter |
| MIMAT0000245 | miR-30c-5p (and other miRNAs w/seed GUAAACA) | 3.150 | TargetScan Human | Moderate (predicted) | SETD3 | nain containing 3, actin histidine methyltra | Nucleus | enzyme |
| MIMAT0000245 | miR-30c-5p (and other miRNAs w/seed GUAAACA) | 3.150 | TargetScan Human | Moderate (predicted) | SGMS2 | sphingomyelin synthase 2 | Plasma Membrane | enzyme |
| MIMAT0000245 | miR-30c-5p (and other miRNAs w/seed GUAAACA) | 3.150 | TargetScan Human | Moderate (predicted) | SH2B3 | SH2B adaptor protein 3 | Plasma Membrane | other |
| MIMAT0000245 | miR-30c-5p (and other miRNAs w/seed GUAAACA) | 3.150 | TargetScan Human | Moderate (predicted) | SH3RF1 | SH3 domain containing ring finger 1 | Cytoplasm | enzyme |
| MIMAT0000245 | miR-30c-5p (and other miRNAs w/seed GUAAACA) | 3.150 | TargetScan Human | Moderate (predicted) | SH3YL1 | SH3 and SYLF domain containing 1 | Plasma Membrane | other |
| MIMAT0000245 | miR-30c-5p (and other miRNAs w/seed GUAAACA) | 3.150 | TargetScan Human | Moderate (predicted) | SHISA3 | shisa family member 3 | Other | other |
| MIMAT0000245 | miR-30c-5p (and other miRNAs w/seed GUAAACA) | 3.150 | TargetScan Human | Moderate (predicted) | SHOC2 | SHOC2 leucine rich repeat scaffold protei | Cytoplasm | other |
| MIMAT0000245 | miR-30c-5p (and other miRNAs w/seed GUAAACA) | 3.150 | TargetScan Human | Moderate (predicted) | SIGMAR1 | sigma non-opioid intracellular receptor 1 | Plasma Membrane | transmembrane receptor |
| MIMAT0000245 | miR-30c-5p (and other miRNAs w/seed GUAAACA) | 3.150 | TargetScan Human | Moderate (predicted) | SIRT1 | sirtuin 1 | Nucleus | transcription regulator |

Sup. Table 3. List of 3699 targets of the seven dysregulated miRNAs obtained from three different target prediction databases,TargetScan TarBase and miRecords considering those predicted as moderate, high, or experimentally validated, respectively.

| | | | | | | | | |
|---|---|---|---|---|---|---|---|---|
| MIMAT0000245 | miR-30c-5p (and other miRNAs w/seed GUAAACA) | 3.150 | TargetScan Human | Moderate (predicted) | SIX1 | SIX homeobox 1 | Nucleus | transcription regulator |
| MIMAT0000245 | miR-30c-5p (and other miRNAs w/seed GUAAACA) | 3.150 | TargetScan Human | Moderate (predicted) | SIX4 | SIX homeobox 4 | Nucleus | transcription regulator |
| MIMAT0000245 | miR-30c-5p (and other miRNAs w/seed GUAAACA) | 3.150 | TargetScan Human | Moderate (predicted) | SKIL | SKI like proto-oncogene | Nucleus | transcription regulator |
| MIMAT0000245 | miR-30c-5p (and other miRNAs w/seed GUAAACA) | 3.150 | TargetScan Human | Moderate (predicted) | SKP2 | S-phase kinase associated protein 2 | Nucleus | other |
| MIMAT0000245 | miR-30c-5p (and other miRNAs w/seed GUAAACA) | 3.150 | TarBase | Experimentally Observed | SLC12A4 | solute carrier family 12 member 4 | Plasma Membrane | transporter |
| MIMAT0000245 | miR-30c-5p (and other miRNAs w/seed GUAAACA) | 3.150 | TargetScan Human | High (predicted) | SLC1A2 | solute carrier family 1 member 2 | Plasma Membrane | transporter |
| MIMAT0000245 | miR-30c-5p (and other miRNAs w/seed GUAAACA) | 3.150 | TargetScan Human | Moderate (predicted) | SLC22A5 | solute carrier family 22 member 5 | Plasma Membrane | transporter |
| MIMAT0000245 | miR-30c-5p (and other miRNAs w/seed GUAAACA) | 3.150 | TargetScan Human | Moderate (predicted) | SLC25A34 | solute carrier family 25 member 34 | Cytoplasm | other |
| MIMAT0000245 | miR-30c-5p (and other miRNAs w/seed GUAAACA) | 3.150 | TargetScan Human | Moderate (predicted) | SLC29A3 | solute carrier family 29 member 3 | Plasma Membrane | transporter |
| MIMAT0000245 | miR-30c-5p (and other miRNAs w/seed GUAAACA) | 3.150 | TargetScan Human | Moderate (predicted) | SLC35A3 | solute carrier family 35 member A3 | Cytoplasm | transporter |
| MIMAT0000245 | miR-30c-5p (and other miRNAs w/seed GUAAACA) | 3.150 | TargetScan Human | Moderate (predicted) | SLC35A5 | solute carrier family 35 member A5 | Other | transporter |
| MIMAT0000245 | miR-30c-5p (and other miRNAs w/seed GUAAACA) | 3.150 | TargetScan Human | High (predicted) | SLC35B4 | solute carrier family 35 member B4 | Cytoplasm | transporter |
| MIMAT0000245 | miR-30c-5p (and other miRNAs w/seed GUAAACA) | 3.150 | TargetScan Human | High (predicted) | SLC35C1 | solute carrier family 35 member C1 | Cytoplasm | transporter |
| MIMAT0000245 | miR-30c-5p (and other miRNAs w/seed GUAAACA) | 3.150 | TargetScan Human | Moderate (predicted) | SLC35D3 | solute carrier family 35 member D3 | Cytoplasm | other |
| MIMAT0000245 | miR-30c-5p (and other miRNAs w/seed GUAAACA) | 3.150 | TargetScan Human | High (predicted) | SLC35F1 | solute carrier family 35 member F1 | Other | other |
| MIMAT0000245 | miR-30c-5p (and other miRNAs w/seed GUAAACA) | 3.150 | TargetScan Human | Moderate (predicted) | SLC35F3 | solute carrier family 35 member F3 | Other | other |
| MIMAT0000245 | miR-30c-5p (and other miRNAs w/seed GUAAACA) | 3.150 | TargetScan Human | Moderate (predicted) | SLC35F4 | solute carrier family 35 member F4 | Other | other |
| MIMAT0000245 | miR-30c-5p (and other miRNAs w/seed GUAAACA) | 3.150 | TargetScan Human | Moderate (predicted) | SLC35G2 | solute carrier family 35 member G2 | Cytoplasm | other |
| MIMAT0000245 | miR-30c-5p (and other miRNAs w/seed GUAAACA) | 3.150 | arBase,TargetScan Hum | ntally Observed,Moderate ( | SLC38A1 | solute carrier family 38 member 1 | Plasma Membrane | transporter |
| MIMAT0000245 | miR-30c-5p (and other miRNAs w/seed GUAAACA) | 3.150 | TarBase | Experimentally Observed | SLC38A2 | solute carrier family 38 member 2 | Plasma Membrane | transporter |
| MIMAT0000245 | miR-30c-5p (and other miRNAs w/seed GUAAACA) | 3.150 | TargetScan Human | Moderate (predicted) | SLC41A2 | solute carrier family 41 member 2 | Plasma Membrane | transporter |
| MIMAT0000245 | miR-30c-5p (and other miRNAs w/seed GUAAACA) | 3.150 | TargetScan Human | Moderate (predicted) | SLC44A5 | solute carrier family 44 member 5 | Plasma Membrane | transporter |
| MIMAT0000245 | miR-30c-5p (and other miRNAs w/seed GUAAACA) | 3.150 | TarBase | Experimentally Observed | SLC4A10 | solute carrier family 4 member 10 | Plasma Membrane | transporter |
| MIMAT0000245 | miR-30c-5p (and other miRNAs w/seed GUAAACA) | 3.150 | TarBase | Experimentally Observed | SLC4A7 | solute carrier family 4 member 7 | Plasma Membrane | transporter |
| MIMAT0000245 | miR-30c-5p (and other miRNAs w/seed GUAAACA) | 3.150 | TargetScan Human | Moderate (predicted) | SLC5A11 | solute carrier family 5 member 11 | Plasma Membrane | transporter |
| MIMAT0000245 | miR-30c-5p (and other miRNAs w/seed GUAAACA) | 3.150 | TargetScan Human | Moderate (predicted) | SLC5A3 | solute carrier family 5 member 3 | Plasma Membrane | transporter |
| MIMAT0000245 | miR-30c-5p (and other miRNAs w/seed GUAAACA) | 3.150 | TargetScan Human | Moderate (predicted) | SLC6A6 | solute carrier family 6 member 6 | Plasma Membrane | transporter |
| MIMAT0000245 | miR-30c-5p (and other miRNAs w/seed GUAAACA) | 3.150 | TargetScan Human | Moderate (predicted) | SLC6A9 | solute carrier family 6 member 9 | Plasma Membrane | transporter |
| MIMAT0000245 | miR-30c-5p (and other miRNAs w/seed GUAAACA) | 3.150 | TarBase | Experimentally Observed | SLC7A1 | solute carrier family 7 member 1 | Plasma Membrane | transporter |
| MIMAT0000245 | miR-30c-5p (and other miRNAs w/seed GUAAACA) | 3.150 | TargetScan Human | High (predicted) | SLC7A10 | solute carrier family 7 member 10 | Plasma Membrane | transporter |
| MIMAT0000245 | miR-30c-5p (and other miRNAs w/seed GUAAACA) | 3.150 | TarBase | Experimentally Observed | SLC7A11 | solute carrier family 7 member 11 | Plasma Membrane | transporter |
| MIMAT0000245 | miR-30c-5p (and other miRNAs w/seed GUAAACA) | 3.150 | TarBase | Experimentally Observed | SLC9A3R2 | SLC9A3 regulator 2 | Plasma Membrane | transporter |
| MIMAT0000245 | miR-30c-5p (and other miRNAs w/seed GUAAACA) | 3.150 | TargetScan Human | Moderate (predicted) | SLC9B2 | solute carrier family 9 member B2 | Plasma Membrane | transporter |
| MIMAT0000245 | miR-30c-5p (and other miRNAs w/seed GUAAACA) | 3.150 | TargetScan Human | High (predicted) | SLCO6A1 | arrier organic anion transporter family men | Plasma Membrane | transporter |
| MIMAT0000245 | miR-30c-5p (and other miRNAs w/seed GUAAACA) | 3.150 | TargetScan Human | High (predicted) | SMAD1 | SMAD family member 1 | Nucleus | transcription regulator |
| MIMAT0000245 | miR-30c-5p (and other miRNAs w/seed GUAAACA) | 3.150 | TargetScan Human | High (predicted) | SMAP1 | small ArfGAP 1 | Cytoplasm | other |
| MIMAT0000245 | miR-30c-5p (and other miRNAs w/seed GUAAACA) | 3.150 | TargetScan Human | Moderate (predicted) | SMIM14 | small integral membrane protein 14 | Cytoplasm | other |
| MIMAT0000245 | miR-30c-5p (and other miRNAs w/seed GUAAACA) | 3.150 | TargetScan Human | High (predicted) | SNAI1 | snail family transcriptional repressor 1 | Nucleus | transcription regulator |
| MIMAT0000245 | miR-30c-5p (and other miRNAs w/seed GUAAACA) | 3.150 | TargetScan Human | Moderate (predicted) | SNAPIN | SNAP associated protein | Plasma Membrane | other |
| MIMAT0000245 | miR-30c-5p (and other miRNAs w/seed GUAAACA) | 3.150 | TargetScan Human | Moderate (predicted) | SNX10 | sorting nexin 10 | Cytoplasm | transporter |
| MIMAT0000245 | miR-30c-5p (and other miRNAs w/seed GUAAACA) | 3.150 | TargetScan Human | High (predicted) | SNX16 | sorting nexin 16 | Cytoplasm | transporter |
| MIMAT0000245 | miR-30c-5p (and other miRNAs w/seed GUAAACA) | 3.150 | TargetScan Human | Moderate (predicted) | SNX18 | sorting nexin 18 | Cytoplasm | transporter |
| MIMAT0000245 | miR-30c-5p (and other miRNAs w/seed GUAAACA) | 3.150 | TargetScan Human | High (predicted) | SOCS1 | suppressor of cytokine signaling 1 | Cytoplasm | other |
| MIMAT0000245 | miR-30c-5p (and other miRNAs w/seed GUAAACA) | 3.150 | TargetScan Human | High (predicted) | SOCS3 | suppressor of cytokine signaling 3 | Cytoplasm | phosphatase |
| MIMAT0000245 | miR-30c-5p (and other miRNAs w/seed GUAAACA) | 3.150 | TargetScan Human | Moderate (predicted) | SOCS6 | suppressor of cytokine signaling 6 | Cytoplasm | other |
| MIMAT0000245 | miR-30c-5p (and other miRNAs w/seed GUAAACA) | 3.150 | TargetScan Human | Moderate (predicted) | SORD | sorbitol dehydrogenase | Cytoplasm | enzyme |
| MIMAT0000245 | miR-30c-5p (and other miRNAs w/seed GUAAACA) | 3.150 | TargetScan Human | Moderate (predicted) | SOS1 | Ras/Rac guanine nucleotide exchange fa | Cytoplasm | other |
| MIMAT0000245 | miR-30c-5p (and other miRNAs w/seed GUAAACA) | 3.150 | TargetScan Human | Moderate (predicted) | SOX4 | SRY-box transcription factor 4 | Nucleus | transcription regulator |
| MIMAT0000245 | miR-30c-5p (and other miRNAs w/seed GUAAACA) | 3.150 | TargetScan Human | Moderate (predicted) | SOX9 | SRY-box transcription factor 9 | Nucleus | transcription regulator |
| MIMAT0000245 | miR-30c-5p (and other miRNAs w/seed GUAAACA) | 3.150 | TargetScan Human | Moderate (predicted) | SP4 | Sp4 transcription factor | Nucleus | transcription regulator |
| MIMAT0000245 | miR-30c-5p (and other miRNAs w/seed GUAAACA) | 3.150 | ngenuity Expert Finding | Experimentally Observed | SPARC | secreted protein acidic and cysteine rich | Extracellular Space | other |
| MIMAT0000245 | miR-30c-5p (and other miRNAs w/seed GUAAACA) | 3.150 | TargetScan Human | Moderate (predicted) | SPAST | spastin | Nucleus | enzyme |
| MIMAT0000245 | miR-30c-5p (and other miRNAs w/seed GUAAACA) | 3.150 | TargetScan Human | Moderate (predicted) | SPATA9 | spermatogenesis associated 9 | Other | other |
| MIMAT0000245 | miR-30c-5p (and other miRNAs w/seed GUAAACA) | 3.150 | TargetScan Human | Moderate (predicted) | SPEN | spen family transcriptional repressor | Nucleus | transcription regulator |
| MIMAT0000245 | miR-30c-5p (and other miRNAs w/seed GUAAACA) | 3.150 | TargetScan Human | Moderate (predicted) | SPHKAP | SPHK1 interactor, AKAP domain containin | Cytoplasm | other |
| MIMAT0000245 | miR-30c-5p (and other miRNAs w/seed GUAAACA) | 3.150 | TargetScan Human | Moderate (predicted) | SPOCK3 | eonectin), cwcv and kazal like domains pr | Extracellular Space | other |
| MIMAT0000245 | miR-30c-5p (and other miRNAs w/seed GUAAACA) | 3.150 | TargetScan Human | Moderate (predicted) | SPTLC3 | e palmitoyltransferase long chain base sub | Cytoplasm | enzyme |
| MIMAT0000245 | miR-30c-5p (and other miRNAs w/seed GUAAACA) | 3.150 | TargetScan Human | High (predicted) | SRSF10 | serine and arginine rich splicing factor 10 | Nucleus | other |
| MIMAT0000245 | miR-30c-5p (and other miRNAs w/seed GUAAACA) | 3.150 | TargetScan Human | High (predicted) | SRSF7 | serine and arginine rich splicing factor 7 | Nucleus | other |
| MIMAT0000245 | miR-30c-5p (and other miRNAs w/seed GUAAACA) | 3.150 | TargetScan Human | Moderate (predicted) | SSBP2 | single stranded DNA binding protein 2 | Nucleus | transcription regulator |
| MIMAT0000245 | miR-30c-5p (and other miRNAs w/seed GUAAACA) | 3.150 | TargetScan Human | Moderate (predicted) | SSR3 | signal sequence receptor subunit 3 | Cytoplasm | other |
| MIMAT0000245 | miR-30c-5p (and other miRNAs w/seed GUAAACA) | 3.150 | TargetScan Human | Moderate (predicted) | SSTR2 | somatostatin receptor 2 | Plasma Membrane | G-protein coupled receptor |
| MIMAT0000245 | miR-30c-5p (and other miRNAs w/seed GUAAACA) | 3.150 | TargetScan Human | Moderate (predicted) | ST8SIA1 | a-N-acetyl-neuraminide alpha-2,8-sialyltra | Cytoplasm | enzyme |
| MIMAT0000245 | miR-30c-5p (and other miRNAs w/seed GUAAACA) | 3.150 | TargetScan Human | Moderate (predicted) | ST8SIA4 | a-N-acetyl-neuraminide alpha-2,8-sialyltra | Cytoplasm | enzyme |
| MIMAT0000245 | miR-30c-5p (and other miRNAs w/seed GUAAACA) | 3.150 | TargetScan Human | High (predicted) | STAC | SH3 and cysteine rich domain | Cytoplasm | other |
| MIMAT0000245 | miR-30c-5p (and other miRNAs w/seed GUAAACA) | 3.150 | TargetScan Human | Moderate (predicted) | STARD6 | AR related lipid transfer domain containin | Other | transporter |
| MIMAT0000245 | miR-30c-5p (and other miRNAs w/seed GUAAACA) | 3.150 | TargetScan Human | High (predicted) | STIM2 | stromal interaction molecule 2 | Plasma Membrane | transporter |
| MIMAT0000245 | miR-30c-5p (and other miRNAs w/seed GUAAACA) | 3.150 | TargetScan Human | Moderate (predicted) | STK35 | serine/threonine kinase 35 | Cytoplasm | kinase |
| MIMAT0000245 | miR-30c-5p (and other miRNAs w/seed GUAAACA) | 3.150 | TargetScan Human | Moderate (predicted) | STK39 | serine/threonine kinase 39 | Nucleus | kinase |
| MIMAT0000245 | miR-30c-5p (and other miRNAs w/seed GUAAACA) | 3.150 | TargetScan Human | Moderate (predicted) | STMND1 | stathmin domain containing 1 | Other | other |
| MIMAT0000245 | miR-30c-5p (and other miRNAs w/seed GUAAACA) | 3.150 | TargetScan Human | High (predicted) | STOX2 | storkhead box 2 | Other | other |
| MIMAT0000245 | miR-30c-5p (and other miRNAs w/seed GUAAACA) | 3.150 | TargetScan Human | Moderate (predicted) | STRIP1 | striatin interacting protein 1 | Nucleus | other |
| MIMAT0000245 | miR-30c-5p (and other miRNAs w/seed GUAAACA) | 3.150 | TarBase | Experimentally Observed | STRN | striatin | Cytoplasm | other |
| MIMAT0000245 | miR-30c-5p (and other miRNAs w/seed GUAAACA) | 3.150 | TargetScan Human | Moderate (predicted) | STX16 | syntaxin 16 | Cytoplasm | transporter |
| MIMAT0000245 | miR-30c-5p (and other miRNAs w/seed GUAAACA) | 3.150 | miRecords | Experimentally Observed | STX1A | syntaxin 1A | Cytoplasm | transporter |
| MIMAT0000245 | miR-30c-5p (and other miRNAs w/seed GUAAACA) | 3.150 | TargetScan Human | High (predicted) | STX2 | syntaxin 2 | Cytoplasm | transporter |
| MIMAT0000245 | miR-30c-5p (and other miRNAs w/seed GUAAACA) | 3.150 | TarBase | Experimentally Observed | STX7 | syntaxin 7 | Plasma Membrane | transporter |
| MIMAT0000245 | miR-30c-5p (and other miRNAs w/seed GUAAACA) | 3.150 | TargetScan Human | Moderate (predicted) | STXBP5 | syntaxin binding protein 5 | Plasma Membrane | other |
| MIMAT0000245 | miR-30c-5p (and other miRNAs w/seed GUAAACA) | 3.150 | TargetScan Human | Moderate (predicted) | SURF4 | surfeit 4 | Cytoplasm | other |
| MIMAT0000245 | miR-30c-5p (and other miRNAs w/seed GUAAACA) | 3.150 | TargetScan Human | Moderate (predicted) | SYBU | syntabulin | Cytoplasm | other |
| MIMAT0000245 | miR-30c-5p (and other miRNAs w/seed GUAAACA) | 3.150 | TargetScan Human | Moderate (predicted) | SYCE2 | naptonemal complex central element prote | Nucleus | other |
| MIMAT0000245 | miR-30c-5p (and other miRNAs w/seed GUAAACA) | 3.150 | TargetScan Human | High (predicted) | SYNGR3 | synaptogyrin 3 | Plasma Membrane | other |
| MIMAT0000245 | miR-30c-5p (and other miRNAs w/seed GUAAACA) | 3.150 | TarBase | Experimentally Observed | SYPL1 | synaptophysin like 1 | Plasma Membrane | transporter |
| MIMAT0000245 | miR-30c-5p (and other miRNAs w/seed GUAAACA) | 3.150 | miRecords | Experimentally Observed | SYT4 | synaptotagmin 4 | Cytoplasm | transporter |
| MIMAT0000245 | miR-30c-5p (and other miRNAs w/seed GUAAACA) | 3.150 | TargetScan Human | Moderate (predicted) | TAF4B | ATA-box binding protein associated factor | Nucleus | transcription regulator |
| MIMAT0000245 | miR-30c-5p (and other miRNAs w/seed GUAAACA) | 3.150 | TargetScan Human | Moderate (predicted) | TAOK1 | TAO kinase 1 | Cytoplasm | kinase |
| MIMAT0000245 | miR-30c-5p (and other miRNAs w/seed GUAAACA) | 3.150 | TargetScan Human | Moderate (predicted) | TASP1 | taspase 1 | Nucleus | peptidase |
| MIMAT0000245 | miR-30c-5p (and other miRNAs w/seed GUAAACA) | 3.150 | TargetScan Human | Moderate (predicted) | TBC1D10B | TBC1 domain family member 10B | Plasma Membrane | enzyme |
| MIMAT0000245 | miR-30c-5p (and other miRNAs w/seed GUAAACA) | 3.150 | TargetScan Human | Moderate (predicted) | TBPL1 | TATA-box binding protein like 1 | Nucleus | transcription regulator |
| MIMAT0000245 | miR-30c-5p (and other miRNAs w/seed GUAAACA) | 3.150 | TargetScan Human | Moderate (predicted) | TCF24 | transcription factor 24 | Other | other |
| MIMAT0000245 | miR-30c-5p (and other miRNAs w/seed GUAAACA) | 3.150 | TargetScan Human | Moderate (predicted) | TCF7 | transcription factor 7 | Nucleus | transcription regulator |
| MIMAT0000245 | miR-30c-5p (and other miRNAs w/seed GUAAACA) | 3.150 | TargetScan Human | Moderate (predicted) | TCIM | nscriptional and immune response regula | Nucleus | other |
| MIMAT0000245 | miR-30c-5p (and other miRNAs w/seed GUAAACA) | 3.150 | TargetScan Human | Moderate (predicted) | TDRP | testis development related protein | Cytoplasm | other |
| MIMAT0000245 | miR-30c-5p (and other miRNAs w/seed GUAAACA) | 3.150 | TargetScan Human | Moderate (predicted) | TEAD1 | TEA domain transcription factor 1 | Nucleus | transcription regulator |
| MIMAT0000245 | miR-30c-5p (and other miRNAs w/seed GUAAACA) | 3.150 | TargetScan Human | High (predicted) | TENT2 | terminal nucleotidyltransferase 2 | Nucleus | enzyme |
| MIMAT0000245 | miR-30c-5p (and other miRNAs w/seed GUAAACA) | 3.150 | TargetScan Human | Moderate (predicted) | TENT5A | terminal nucleotidyltransferase 5A | Other | other |
| MIMAT0000245 | miR-30c-5p (and other miRNAs w/seed GUAAACA) | 3.150 | TargetScan Human | Moderate (predicted) | TEPSIN | adaptor related protein complex 4 accesso | Nucleus | other |
| MIMAT0000245 | miR-30c-5p (and other miRNAs w/seed GUAAACA) | 3.150 | TargetScan Human | Moderate (predicted) | TEX2 | testis expressed 2 | Other | other |
| MIMAT0000245 | miR-30c-5p (and other miRNAs w/seed GUAAACA) | 3.150 | TargetScan Human | Moderate (predicted) | TFDP1 | transcription factor Dp-1 | Nucleus | transcription regulator |
| MIMAT0000245 | miR-30c-5p (and other miRNAs w/seed GUAAACA) | 3.150 | TargetScan Human | Moderate (predicted) | TFRC | transferrin receptor | Plasma Membrane | transporter |
| MIMAT0000245 | miR-30c-5p (and other miRNAs w/seed GUAAACA) | 3.150 | TargetScan Human | Moderate (predicted) | TGDS | TDP-glucose 4,6-dehydratase | Extracellular Space | enzyme |
| MIMAT0000245 | miR-30c-5p (and other miRNAs w/seed GUAAACA) | 3.150 | ngenuity Expert Finding | Experimentally Observed | TGFBR1 | transforming growth factor beta receptor 1 | Plasma Membrane | kinase |
| MIMAT0000245 | miR-30c-5p (and other miRNAs w/seed GUAAACA) | 3.150 | ngenuity Expert Finding | Experimentally Observed | TGFBR2 | transforming growth factor beta receptor 2 | Plasma Membrane | kinase |
| MIMAT0000245 | miR-30c-5p (and other miRNAs w/seed GUAAACA) | 3.150 | TargetScan Human | Moderate (predicted) | THAP12 | THAP domain containing 12 | Nucleus | other |
| MIMAT0000245 | miR-30c-5p (and other miRNAs w/seed GUAAACA) | 3.150 | TarBase | Experimentally Observed | THEM4 | thioesterase superfamily member 4 | Plasma Membrane | enzyme |
| MIMAT0000245 | miR-30c-5p (and other miRNAs w/seed GUAAACA) | 3.150 | TargetScan Human | High (predicted) | TIA1 | cytotoxic granule associated RNA binding | Nucleus | other |
| MIMAT0000245 | miR-30c-5p (and other miRNAs w/seed GUAAACA) | 3.150 | TargetScan Human | Moderate (predicted) | TICAM1 | toll like receptor adaptor molecule 1 | Nucleus | other |
| MIMAT0000245 | miR-30c-5p (and other miRNAs w/seed GUAAACA) | 3.150 | TargetScan Human | Moderate (predicted) | TIGD6 | tigger transposable element derived 6 | Nucleus | other |
| MIMAT0000245 | miR-30c-5p (and other miRNAs w/seed GUAAACA) | 3.150 | TargetScan Human | Moderate (predicted) | TLL2 | tolloid like 2 | Extracellular Space | peptidase |
| MIMAT0000245 | miR-30c-5p (and other miRNAs w/seed GUAAACA) | 3.150 | TargetScan Human | High (predicted) | TM4SF20 | transmembrane 4 L six family member 20 | Cytoplasm | other |
| MIMAT0000245 | miR-30c-5p (and other miRNAs w/seed GUAAACA) | 3.150 | TargetScan Human | Moderate (predicted) | TMCC1 | nsmembrane and coiled-coil domain famil | Cytoplasm | other |
| MIMAT0000245 | miR-30c-5p (and other miRNAs w/seed GUAAACA) | 3.150 | TarBase | Experimentally Observed | TMCO1 | transmembrane and coiled-coil domains 1 | Cytoplasm | ion channel |
| MIMAT0000245 | miR-30c-5p (and other miRNAs w/seed GUAAACA) | 3.150 | arBase,TargetScan Hum | ntally Observed,Moderate ( | TMED10 | transmembrane p24 trafficking protein 10 | Cytoplasm | transporter |
| MIMAT0000245 | miR-30c-5p (and other miRNAs w/seed GUAAACA) | 3.150 | arBase,TargetScan Hum | ntally Observed,Moderate ( | TMED2 | transmembrane p24 trafficking protein 2 | Cytoplasm | transporter |
| MIMAT0000245 | miR-30c-5p (and other miRNAs w/seed GUAAACA) | 3.150 | TarBase | Experimentally Observed | TMED3 | transmembrane p24 trafficking protein 3 | Cytoplasm | transporter |
| MIMAT0000245 | miR-30c-5p (and other miRNAs w/seed GUAAACA) | 3.150 | TarBase | Experimentally Observed | TMED7 | transmembrane p24 trafficking protein 7 | Cytoplasm | transporter |
| MIMAT0000245 | miR-30c-5p (and other miRNAs w/seed GUAAACA) | 3.150 | TargetScan Human | High (predicted) | TMEFF1 | ne protein with EGF like and two follistatin | Plasma Membrane | other |
| MIMAT0000245 | miR-30c-5p (and other miRNAs w/seed GUAAACA) | 3.150 | TargetScan Human | Moderate (predicted) | TMEM106B | transmembrane protein 106B | Cytoplasm | other |
| MIMAT0000245 | miR-30c-5p (and other miRNAs w/seed GUAAACA) | 3.150 | TargetScan Human | High (predicted) | TMEM121 | transmembrane protein 121 | Other | other |
| MIMAT0000245 | miR-30c-5p (and other miRNAs w/seed GUAAACA) | 3.150 | TargetScan Human | High (predicted) | TMEM170B | transmembrane protein 170B | Plasma Membrane | other |
| MIMAT0000245 | miR-30c-5p (and other miRNAs w/seed GUAAACA) | 3.150 | TargetScan Human | High (predicted) | TMEM181 | transmembrane protein 181 | Other | other |
| MIMAT0000245 | miR-30c-5p (and other miRNAs w/seed GUAAACA) | 3.150 | TargetScan Human | Moderate (predicted) | TMEM229A | transmembrane protein 229A | Other | other |
| MIMAT0000245 | miR-30c-5p (and other miRNAs w/seed GUAAACA) | 3.150 | TargetScan Human | Moderate (predicted) | TMEM30B | transmembrane protein 30B | Cytoplasm | transporter |
| MIMAT0000245 | miR-30c-5p (and other miRNAs w/seed GUAAACA) | 3.150 | TargetScan Human | Moderate (predicted) | TMEM35A | transmembrane protein 35A | Cytoplasm | other |
| MIMAT0000245 | miR-30c-5p (and other miRNAs w/seed GUAAACA) | 3.150 | TarBase | Experimentally Observed | TMEM41B | transmembrane protein 41B | Cytoplasm | other |
| MIMAT0000245 | miR-30c-5p (and other miRNAs w/seed GUAAACA) | 3.150 | TarBase | Experimentally Observed | TMEM59 | transmembrane protein 59 | Plasma Membrane | peptidase |
| MIMAT0000245 | miR-30c-5p (and other miRNAs w/seed GUAAACA) | 3.150 | arBase,TargetScan Hum | ntally Observed,Moderate ( | TMEM87A | transmembrane protein 87A | Cytoplasm | other |
| MIMAT0000245 | miR-30c-5p (and other miRNAs w/seed GUAAACA) | 3.150 | TargetScan Human | Moderate (predicted) | TMOD2 | tropomodulin 2 | Cytoplasm | other |
| MIMAT0000245 | miR-30c-5p (and other miRNAs w/seed GUAAACA) | 3.150 | TargetScan Human | Moderate (predicted) | TMTC3 | brane O-mannosyltransferase targeting ca | Cytoplasm | enzyme |
| MIMAT0000245 | miR-30c-5p (and other miRNAs w/seed GUAAACA) | 3.150 | TarBase | Experimentally Observed | TNFAIP2 | TNF alpha induced protein 2 | Extracellular Space | other |
| MIMAT0000245 | miR-30c-5p (and other miRNAs w/seed GUAAACA) | 3.150 | arBase,TargetScan Hum | ntally Observed,Moderate ( | TNFRSF10B | TNF receptor superfamily member 10b | Plasma Membrane | transmembrane receptor |
| MIMAT0000245 | miR-30c-5p (and other miRNAs w/seed GUAAACA) | 3.150 | TargetScan Human | Moderate (predicted) | TNFRSF10D | TNF receptor superfamily member 10d | Plasma Membrane | transmembrane receptor |
| MIMAT0000245 | miR-30c-5p (and other miRNAs w/seed GUAAACA) | 3.150 | TargetScan Human | High (predicted) | TNFSF13B | TNF superfamily member 13b | Extracellular Space | cytokine |
| MIMAT0000245 | miR-30c-5p (and other miRNAs w/seed GUAAACA) | 3.150 | TargetScan Human | Moderate (predicted) | TNPO1 | transportin 1 | Nucleus | transporter |
| MIMAT0000245 | miR-30c-5p (and other miRNAs w/seed GUAAACA) | 3.150 | rgetScan Human,miRec | nentally Observed,High (pr | TNRC6A | trinucleotide repeat containing adaptor 6A | Nucleus | other |
| MIMAT0000245 | miR-30c-5p (and other miRNAs w/seed GUAAACA) | 3.150 | TargetScan Human | Moderate (predicted) | TOMM20 | nslocase of outer mitochondrial membrane | Cytoplasm | transporter |
| MIMAT0000245 | miR-30c-5p (and other miRNAs w/seed GUAAACA) | 3.150 | TargetScan Human | Moderate (predicted) | TOMM5 | nslocase of outer mitochondrial membran | Cytoplasm | other |
| MIMAT0000245 | miR-30c-5p (and other miRNAs w/seed GUAAACA) | 3.150 | miRecords | Experimentally Observed | TP53 | tumor protein p53 | Nucleus | transcription regulator |
| MIMAT0000245 | miR-30c-5p (and other miRNAs w/seed GUAAACA) | 3.150 | ngenuity Expert Finding | Experimentally Observed | Tpm1 | tropomyosin 1, alpha | Plasma Membrane | other |
| MIMAT0000245 | miR-30c-5p (and other miRNAs w/seed GUAAACA) | 3.150 | TargetScan Human | Moderate (predicted) | TPRX1 | tetrapeptide repeat homeobox 1 | Nucleus | transcription regulator |
| MIMAT0000245 | miR-30c-5p (and other miRNAs w/seed GUAAACA) | 3.150 | TargetScan Human | Moderate (predicted) | TRAF3IP2 | TRAF3 interacting protein 2 | Cytoplasm | enzyme |
| MIMAT0000245 | miR-30c-5p (and other miRNAs w/seed GUAAACA) | 3.150 | TargetScan Human | Moderate (predicted) | TRAPPC13 | afficking protein particle complex subunit 1 | Cytoplasm | other |
| MIMAT0000245 | miR-30c-5p (and other miRNAs w/seed GUAAACA) | 3.150 | TargetScan Human | High (predicted) | TRAPPC14 | afficking protein particle complex subunit 1 | Cytoplasm | other |
| MIMAT0000245 | miR-30c-5p (and other miRNAs w/seed GUAAACA) | 3.150 | TargetScan Human | Moderate (predicted) | TRAPPC6B | afficking protein particle complex subunit 6 | Cytoplasm | other |
| MIMAT0000245 | miR-30c-5p (and other miRNAs w/seed GUAAACA) | 3.150 | TargetScan Human | Moderate (predicted) | TRDN | triadin | Cytoplasm | other |
| MIMAT0000245 | miR-30c-5p (and other miRNAs w/seed GUAAACA) | 3.150 | TargetScan Human | Moderate (predicted) | TRIM23 | tripartite motif containing 23 | Nucleus | enzyme |

Sup. Table 3. List of 3699 targets of the seven dysregulated miRNAs obtained from three different target prediction databases,TargetScan TarBase and miRecords considering those predicted as moderate, high, or experimentally validated, respectively.

| | | | | | | | | |
|---|---|---|---|---|---|---|---|---|
| MIMAT0000245 | miR-30c-5p (and other miRNAs w/seed GUAAACA) | 3.150 | TargetScan Human | Moderate (predicted) | TRIP6 | thyroid hormone receptor interactor 6 | Extracellular Space | cytokine |
| MIMAT0000245 | miR-30c-5p (and other miRNAs w/seed GUAAACA) | 3.150 | TargetScan Human | Moderate (predicted) | TRIQK | triple QxxK/R motif containing | Cytoplasm | other |
| MIMAT0000245 | miR-30c-5p (and other miRNAs w/seed GUAAACA) | 3.150 | TargetScan Human | Moderate (predicted) | TRMT10C | thyltransferase 10C, mitochondrial RNase | Cytoplasm | enzyme |
| MIMAT0000245 | miR-30c-5p (and other miRNAs w/seed GUAAACA) | 3.150 | TargetScan Human | Moderate (predicted) | TRO | trophinin | Plasma Membrane | other |
| MIMAT0000245 | miR-30c-5p (and other miRNAs w/seed GUAAACA) | 3.150 | TargetScan Human | Moderate (predicted) | TRPM7 | ceptor potential cation channel subfamily M | Plasma Membrane | kinase |
| MIMAT0000245 | miR-30c-5p (and other miRNAs w/seed GUAAACA) | 3.150 | ngenuity Expert Findings | Experimentally Observed | TRPS1 | transcriptional repressor GATA binding 1 | Nucleus | transcription regulator |
| MIMAT0000245 | miR-30c-5p (and other miRNAs w/seed GUAAACA) | 3.150 | TargetScan Human | Moderate (predicted) | TSEN15 | tRNA splicing endonuclease subunit 15 | Nucleus | enzyme |
| MIMAT0000245 | miR-30c-5p (and other miRNAs w/seed GUAAACA) | 3.150 | TargetScan Human | Moderate (predicted) | TTC39A | tetratricopeptide repeat domain 39A | Cytoplasm | other |
| MIMAT0000245 | miR-30c-5p (and other miRNAs w/seed GUAAACA) | 3.150 | TargetScan Human | Moderate (predicted) | TTC8 | tetratricopeptide repeat domain 8 | Cytoplasm | other |
| MIMAT0000245 | miR-30c-5p (and other miRNAs w/seed GUAAACA) | 3.150 | TargetScan Human | High (predicted) | TTLL7 | tubulin tyrosine ligase like 7 | Cytoplasm | enzyme |
| MIMAT0000245 | miR-30c-5p (and other miRNAs w/seed GUAAACA) | 3.150 | TargetScan Human | Moderate (predicted) | TTPA | alpha tocopherol transfer protein | Cytoplasm | transporter |
| MIMAT0000245 | miR-30c-5p (and other miRNAs w/seed GUAAACA) | 3.150 | TargetScan Human | Moderate (predicted) | TUBGCP3 | ubulin gamma complex associated protein | Cytoplasm | other |
| MIMAT0000245 | miR-30c-5p (and other miRNAs w/seed GUAAACA) | 3.150 | TargetScan Human | Moderate (predicted) | TUSC3 | tumor suppressor candidate 3 | Plasma Membrane | transporter |
| MIMAT0000245 | miR-30c-5p (and other miRNAs w/seed GUAAACA) | 3.150 | TargetScan Human | Moderate (predicted) | TVP23B | ns-golgi network vesicle protein 23 homolo | Other | other |
| MIMAT0000245 | miR-30c-5p (and other miRNAs w/seed GUAAACA) | 3.150 | TargetScan Human | High (predicted) | TWF1 | twinfilin actin binding protein 1 | Cytoplasm | kinase |
| MIMAT0000245 | miR-30c-5p (and other miRNAs w/seed GUAAACA) | 3.150 | TargetScan Human | Moderate (predicted) | TXNDC5 | thioredoxin domain containing 5 | Cytoplasm | enzyme |
| MIMAT0000245 | miR-30c-5p (and other miRNAs w/seed GUAAACA) | 3.150 | TarBase | Experimentally Observed | UAP1 | )P-N-acetylglucosamine pyrophosphorylas | Nucleus | enzyme |
| MIMAT0000245 | miR-30c-5p (and other miRNAs w/seed GUAAACA) | 3.150 | TargetScan Human | Moderate (predicted) | UBAC1 | UBA domain containing 1 | Nucleus | other |
| MIMAT0000245 | miR-30c-5p (and other miRNAs w/seed GUAAACA) | 3.150 | TargetScan Human | Moderate (predicted) | UBE2D2 | ubiquitin conjugating enzyme E2 D2 | Cytoplasm | enzyme |
| MIMAT0000245 | miR-30c-5p (and other miRNAs w/seed GUAAACA) | 3.150 | TargetScan Human | Moderate (predicted) | UBE2D3 | ubiquitin conjugating enzyme E2 D3 | Cytoplasm | enzyme |
| MIMAT0000245 | miR-30c-5p (and other miRNAs w/seed GUAAACA) | 3.150 | TargetScan Human | Moderate (predicted) | UBE2F | biquitin conjugating enzyme E2 F (putativ | Cytoplasm | enzyme |
| MIMAT0000245 | miR-30c-5p (and other miRNAs w/seed GUAAACA) | 3.150 | rgetScan Human,miRec | nentally Observed,High (pr | UBE2I | ubiquitin conjugating enzyme E2 I | Nucleus | enzyme |
| MIMAT0000245 | miR-30c-5p (and other miRNAs w/seed GUAAACA) | 3.150 | TargetScan Human | Moderate (predicted) | UBE2J1 | ubiquitin conjugating enzyme E2 J1 | Cytoplasm | enzyme |
| MIMAT0000245 | miR-30c-5p (and other miRNAs w/seed GUAAACA) | 3.150 | TargetScan Human | High (predicted) | UBE2V2 | ubiquitin conjugating enzyme E2 V2 | Cytoplasm | enzyme |
| MIMAT0000245 | miR-30c-5p (and other miRNAs w/seed GUAAACA) | 3.150 | TargetScan Human | Moderate (predicted) | UBE3C | ubiquitin protein ligase E3C | Nucleus | enzyme |
| MIMAT0000245 | miR-30c-5p (and other miRNAs w/seed GUAAACA) | 3.150 | TargetScan Human | High (predicted) | UCP3 | uncoupling protein 3 | Cytoplasm | transporter |
| MIMAT0000245 | miR-30c-5p (and other miRNAs w/seed GUAAACA) | 3.150 | TargetScan Human | Moderate (predicted) | UGCG | UDP-glucose ceramide glucosyltransferas | Cytoplasm | enzyme |
| MIMAT0000245 | miR-30c-5p (and other miRNAs w/seed GUAAACA) | 3.150 | TargetScan Human | High (predicted) | UGT2A3 | P glucuronosyltransferase family 2 membe | Other | enzyme |
| MIMAT0000245 | miR-30c-5p (and other miRNAs w/seed GUAAACA) | 3.150 | TargetScan Human | Moderate (predicted) | UHMK1 | U2AF homology motif kinase 1 | Nucleus | kinase |
| MIMAT0000245 | miR-30c-5p (and other miRNAs w/seed GUAAACA) | 3.150 | TargetScan Human | Moderate (predicted) | UPRT | uracil phosphoribosyltransferase homolog | Nucleus | enzyme |
| MIMAT0000245 | miR-30c-5p (and other miRNAs w/seed GUAAACA) | 3.150 | TargetScan Human | Moderate (predicted) | USO1 | USO1 vesicle transport factor | Cytoplasm | other |
| MIMAT0000245 | miR-30c-5p (and other miRNAs w/seed GUAAACA) | 3.150 | TargetScan Human | Moderate (predicted) | USP2 | ubiquitin specific peptidase 2 | Cytoplasm | peptidase |
| MIMAT0000245 | miR-30c-5p (and other miRNAs w/seed GUAAACA) | 3.150 | TargetScan Human | Moderate (predicted) | USP22 | ubiquitin specific peptidase 22 | Nucleus | peptidase |
| MIMAT0000245 | miR-30c-5p (and other miRNAs w/seed GUAAACA) | 3.150 | TargetScan Human | High (predicted) | USP45 | ubiquitin specific peptidase 45 | Nucleus | peptidase |
| MIMAT0000245 | miR-30c-5p (and other miRNAs w/seed GUAAACA) | 3.150 | TargetScan Human | Moderate (predicted) | USP48 | ubiquitin specific peptidase 48 | Plasma Membrane | peptidase |
| MIMAT0000245 | miR-30c-5p (and other miRNAs w/seed GUAAACA) | 3.150 | TargetScan Human | Moderate (predicted) | VAMP3 | vesicle associated membrane protein 3 | Plasma Membrane | other |
| MIMAT0000245 | miR-30c-5p (and other miRNAs w/seed GUAAACA) | 3.150 | TargetScan Human | Moderate (predicted) | VAPA | VAMP associated protein A | Plasma Membrane | other |
| MIMAT0000245 | miR-30c-5p (and other miRNAs w/seed GUAAACA) | 3.150 | TargetScan Human | Moderate (predicted) | VAT1L | vesicle amine transport 1 like | Other | enzyme |
| MIMAT0000245 | miR-30c-5p (and other miRNAs w/seed GUAAACA) | 3.150 | ngenuity Expert Findings | Experimentally Observed | VCAN | versican | Extracellular Space | other |
| MIMAT0000245 | miR-30c-5p (and other miRNAs w/seed GUAAACA) | 3.150 | TargetScan Human | Moderate (predicted) | VENTX | VENT homeobox | Nucleus | transcription regulator |
| MIMAT0000245 | miR-30c-5p (and other miRNAs w/seed GUAAACA) | 3.150 | TargetScan Human | High (predicted) | VIM | vimentin | Cytoplasm | other |
| MIMAT0000245 | miR-30c-5p (and other miRNAs w/seed GUAAACA) | 3.150 | TargetScan Human | Moderate (predicted) | VIP | vasoactive intestinal peptide | Extracellular Space | other |
| MIMAT0000245 | miR-30c-5p (and other miRNAs w/seed GUAAACA) | 3.150 | TargetScan Human | Moderate (predicted) | VKORC1L1 | in K epoxide reductase complex subunit 1 | Cytoplasm | enzyme |
| MIMAT0000245 | miR-30c-5p (and other miRNAs w/seed GUAAACA) | 3.150 | TargetScan Human | Moderate (predicted) | VOPP1 | VOPP1 WW domain binding protein | Nucleus | other |
| MIMAT0000245 | miR-30c-5p (and other miRNAs w/seed GUAAACA) | 3.150 | TargetScan Human | Moderate (predicted) | VPS26B | VPS26, retromer complex component B | Cytoplasm | transporter |
| MIMAT0000245 | miR-30c-5p (and other miRNAs w/seed GUAAACA) | 3.150 | TargetScan Human | High (predicted) | VWC2 | on Willebrand factor C domain containing | Extracellular Space | other |
| MIMAT0000245 | miR-30c-5p (and other miRNAs w/seed GUAAACA) | 3.150 | TargetScan Human | Moderate (predicted) | WDR44 | WD repeat domain 44 | Cytoplasm | other |
| MIMAT0000245 | miR-30c-5p (and other miRNAs w/seed GUAAACA) | 3.150 | TargetScan Human | Moderate (predicted) | WDR7 | WD repeat domain 7 | Cytoplasm | other |
| MIMAT0000245 | miR-30c-5p (and other miRNAs w/seed GUAAACA) | 3.150 | TargetScan Human | High (predicted) | WDR82 | WD repeat domain 82 | Nucleus | enzyme |
| MIMAT0000245 | miR-30c-5p (and other miRNAs w/seed GUAAACA) | 3.150 | TargetScan Human | High (predicted) | WNK3 | WNK lysine deficient protein kinase 3 | Plasma Membrane | kinase |
| MIMAT0000245 | miR-30c-5p (and other miRNAs w/seed GUAAACA) | 3.150 | TarBase | Experimentally Observed | WNT5A | Wnt family member 5A | Extracellular Space | cytokine |
| MIMAT0000245 | miR-30c-5p (and other miRNAs w/seed GUAAACA) | 3.150 | TargetScan Human | Moderate (predicted) | WWP1 | domain containing E3 ubiquitin protein lig | Cytoplasm | enzyme |
| MIMAT0000245 | miR-30c-5p (and other miRNAs w/seed GUAAACA) | 3.150 | TargetScan Human | Moderate (predicted) | XKR4 | XK related 4 | Plasma Membrane | other |
| MIMAT0000245 | miR-30c-5p (and other miRNAs w/seed GUAAACA) | 3.150 | TargetScan Human | Moderate (predicted) | XPO1 | exportin 1 | Nucleus | transporter |
| MIMAT0000245 | miR-30c-5p (and other miRNAs w/seed GUAAACA) | 3.150 | TargetScan Human | Moderate (predicted) | YAF2 | YY1 associated factor 2 | Nucleus | transcription regulator |
| MIMAT0000245 | miR-30c-5p (and other miRNAs w/seed GUAAACA) | 3.150 | TargetScan Human | Moderate (predicted) | YBX1 | Y-box binding protein 1 | Nucleus | transcription regulator |
| MIMAT0000245 | miR-30c-5p (and other miRNAs w/seed GUAAACA) | 3.150 | TargetScan Human | Moderate (predicted) | YEATS4 | YEATS domain containing 4 | Nucleus | transcription regulator |
| MIMAT0000245 | miR-30c-5p (and other miRNAs w/seed GUAAACA) | 3.150 | TargetScan Human | Moderate (predicted) | YIPF7 | Yip1 domain family member 7 | Other | other |
| MIMAT0000245 | miR-30c-5p (and other miRNAs w/seed GUAAACA) | 3.150 | TargetScan Human | Moderate (predicted) | YOD1 | YOD1 deubiquitinase | Cytoplasm | enzyme |
| MIMAT0000245 | miR-30c-5p (and other miRNAs w/seed GUAAACA) | 3.150 | TargetScan Human | High (predicted) | YPEL2 | yippee like 2 | Nucleus | other |
| MIMAT0000245 | miR-30c-5p (and other miRNAs w/seed GUAAACA) | 3.150 | TargetScan Human | Moderate (predicted) | YPEL5 | yippee like 5 | Nucleus | other |
| MIMAT0000245 | miR-30c-5p (and other miRNAs w/seed GUAAACA) | 3.150 | TargetScan Human | High (predicted) | YTHDC1 | YTH domain containing 1 | Cytoplasm | other |
| MIMAT0000245 | miR-30c-5p (and other miRNAs w/seed GUAAACA) | 3.150 | TargetScan Human | Moderate (predicted) | YWHAZ | ygenase/tryptophan 5-monooxygenase act | Cytoplasm | enzyme |
| MIMAT0000245 | miR-30c-5p (and other miRNAs w/seed GUAAACA) | 3.150 | TargetScan Human | Moderate (predicted) | YY2 | YY2 transcription factor | Nucleus | transcription regulator |
| MIMAT0000245 | miR-30c-5p (and other miRNAs w/seed GUAAACA) | 3.150 | TargetScan Human | High (predicted) | ZBTB11 | zinc finger and BTB domain containing 11 | Nucleus | transcription regulator |
| MIMAT0000245 | miR-30c-5p (and other miRNAs w/seed GUAAACA) | 3.150 | TargetScan Human | Moderate (predicted) | ZBTB18 | zinc finger and BTB domain containing 18 | Nucleus | transcription regulator |
| MIMAT0000245 | miR-30c-5p (and other miRNAs w/seed GUAAACA) | 3.150 | TargetScan Human | Moderate (predicted) | ZBTB39 | zinc finger and BTB domain containing 39 | Nucleus | transcription regulator |
| MIMAT0000245 | miR-30c-5p (and other miRNAs w/seed GUAAACA) | 3.150 | TargetScan Human | High (predicted) | ZBTB41 | zinc finger and BTB domain containing 41 | Other | other |
| MIMAT0000245 | miR-30c-5p (and other miRNAs w/seed GUAAACA) | 3.150 | TargetScan Human | Moderate (predicted) | ZC3H8 | zinc finger CCCH-type containing 8 | Nucleus | transcription regulator |
| MIMAT0000245 | miR-30c-5p (and other miRNAs w/seed GUAAACA) | 3.150 | TargetScan Human | Moderate (predicted) | ZCCHC2 | zinc finger CCHC-type containing 2 | Cytoplasm | other |
| MIMAT0000245 | miR-30c-5p (and other miRNAs w/seed GUAAACA) | 3.150 | TargetScan Human | Moderate (predicted) | ZCCHC4 | zinc finger CCHC-type containing 4 | Nucleus | enzyme |
| MIMAT0000245 | miR-30c-5p (and other miRNAs w/seed GUAAACA) | 3.150 | TargetScan Human | High (predicted) | ZCRB1 | er CCHC-type and RNA binding motif con | Nucleus | other |
| MIMAT0000245 | miR-30c-5p (and other miRNAs w/seed GUAAACA) | 3.150 | TargetScan Human | High (predicted) | ZDHHC20 | nc finger DHHC-type palmitoyltransferase | Plasma Membrane | enzyme |
| MIMAT0000245 | miR-30c-5p (and other miRNAs w/seed GUAAACA) | 3.150 | TargetScan Human | Moderate (predicted) | ZDHHC21 | nc finger DHHC-type palmitoyltransferase | Plasma Membrane | enzyme |
| MIMAT0000245 | miR-30c-5p (and other miRNAs w/seed GUAAACA) | 3.150 | TargetScan Human | Moderate (predicted) | ZEB2 | zinc finger E-box binding homeobox 2 | Nucleus | transcription regulator |
| MIMAT0000245 | miR-30c-5p (and other miRNAs w/seed GUAAACA) | 3.150 | TargetScan Human | Moderate (predicted) | ZFAND1 | zinc finger AN1-type containing 1 | Cytoplasm | other |
| MIMAT0000245 | miR-30c-5p (and other miRNAs w/seed GUAAACA) | 3.150 | TargetScan Human | High (predicted) | ZFAND5 | zinc finger AN1-type containing 5 | Nucleus | other |
| MIMAT0000245 | miR-30c-5p (and other miRNAs w/seed GUAAACA) | 3.150 | TargetScan Human | Moderate (predicted) | ZFC3H1 | zinc finger C3H1-type containing | Extracellular Space | other |
| MIMAT0000245 | miR-30c-5p (and other miRNAs w/seed GUAAACA) | 3.150 | TargetScan Human | Moderate (predicted) | ZFP1 | ZFP1 zinc finger protein | Nucleus | transcription regulator |
| MIMAT0000245 | miR-30c-5p (and other miRNAs w/seed GUAAACA) | 3.150 | TargetScan Human | Moderate (predicted) | ZFP69B | ZFP69 zinc finger protein B | Nucleus | transcription regulator |
| MIMAT0000245 | miR-30c-5p (and other miRNAs w/seed GUAAACA) | 3.150 | TargetScan Human | Moderate (predicted) | ZFY | zinc finger protein Y-linked | Nucleus | transcription regulator |
| MIMAT0000245 | miR-30c-5p (and other miRNAs w/seed GUAAACA) | 3.150 | TargetScan Human | Moderate (predicted) | ZNF200 | zinc finger protein 200 | Nucleus | transcription regulator |
| MIMAT0000245 | miR-30c-5p (and other miRNAs w/seed GUAAACA) | 3.150 | TargetScan Human | Moderate (predicted) | ZNF22-AS1 | ZNF22 antisense RNA 1 | Other | other |
| MIMAT0000245 | miR-30c-5p (and other miRNAs w/seed GUAAACA) | 3.150 | TargetScan Human | Moderate (predicted) | ZNF260 | zinc finger protein 260 | Nucleus | transcription regulator |
| MIMAT0000245 | miR-30c-5p (and other miRNAs w/seed GUAAACA) | 3.150 | TargetScan Human | Moderate (predicted) | ZNF280B | zinc finger protein 280B | Nucleus | transcription regulator |
| MIMAT0000245 | miR-30c-5p (and other miRNAs w/seed GUAAACA) | 3.150 | TargetScan Human | Moderate (predicted) | ZNF382 | zinc finger protein 382 | Nucleus | transcription regulator |
| MIMAT0000245 | miR-30c-5p (and other miRNAs w/seed GUAAACA) | 3.150 | TargetScan Human | Moderate (predicted) | ZNF460 | zinc finger protein 460 | Nucleus | transcription regulator |
| MIMAT0000245 | miR-30c-5p (and other miRNAs w/seed GUAAACA) | 3.150 | TargetScan Human | Moderate (predicted) | ZNF521 | zinc finger protein 521 | Nucleus | transcription regulator |
| MIMAT0000245 | miR-30c-5p (and other miRNAs w/seed GUAAACA) | 3.150 | TargetScan Human | Moderate (predicted) | ZNF529 | zinc finger protein 529 | Other | other |
| MIMAT0000245 | miR-30c-5p (and other miRNAs w/seed GUAAACA) | 3.150 | TargetScan Human | Moderate (predicted) | ZNF608 | zinc finger protein 608 | Other | other |
| MIMAT0000245 | miR-30c-5p (and other miRNAs w/seed GUAAACA) | 3.150 | TargetScan Human | Moderate (predicted) | ZNF678 | zinc finger protein 678 | Nucleus | transcription regulator |
| MIMAT0000245 | miR-30c-5p (and other miRNAs w/seed GUAAACA) | 3.150 | TargetScan Human | Moderate (predicted) | ZNF711 | zinc finger protein 711 | Nucleus | transcription regulator |
| MIMAT0000245 | miR-30c-5p (and other miRNAs w/seed GUAAACA) | 3.150 | TargetScan Human | Moderate (predicted) | ZNF805 | zinc finger protein 805 | Other | other |
| MIMAT0000245 | miR-30c-5p (and other miRNAs w/seed GUAAACA) | 3.150 | TargetScan Human | High (predicted) | ZNRF1 | zinc and ring finger 1 | Cytoplasm | enzyme |
| MIMAT0000245 | miR-30c-5p (and other miRNAs w/seed GUAAACA) | 3.150 | TargetScan Human | High (predicted) | ZPBP2 | zona pellucida binding protein 2 | Extracellular Space | other |
| MIMAT0000245 | miR-30c-5p (and other miRNAs w/seed GUAAACA) | 3.150 | TargetScan Human | Moderate (predicted) | ZRANB2 | zinc finger RANBP2-type containing 2 | Nucleus | other |
| MIMAT0000263 | miR-199a-5p (and other miRNAs w/seed CCAGUGU) | 2.810 | TargetScan Human | Moderate (predicted) | AADAT | aminoadipate aminotransferase | Cytoplasm | enzyme |
| MIMAT0000263 | miR-199a-5p (and other miRNAs w/seed CCAGUGU) | 2.810 | TargetScan Human | Moderate (predicted) | ABCC1 | ATP binding cassette subfamily C member | Plasma Membrane | transporter |
| MIMAT0000263 | miR-199a-5p (and other miRNAs w/seed CCAGUGU) | 2.810 | TargetScan Human | High (predicted) | ABHD17C | drolase domain containing 17C, depalmitc | Plasma Membrane | enzyme |
| MIMAT0000263 | miR-199a-5p (and other miRNAs w/seed CCAGUGU) | 2.810 | TargetScan Human | Moderate (predicted) | ABL2 | proto-oncogene 2, non-receptor tyrosine k | Cytoplasm | kinase |
| MIMAT0000263 | miR-199a-5p (and other miRNAs w/seed CCAGUGU) | 2.810 | TargetScan Human | Moderate (predicted) | ACER2 | alkaline ceramidase 2 | Cytoplasm | enzyme |
| MIMAT0000263 | miR-199a-5p (and other miRNAs w/seed CCAGUGU) | 2.810 | TargetScan Human | Moderate (predicted) | ACHE | cetylcholinesterase (Cartwright blood grou | Plasma Membrane | enzyme |
| MIMAT0000263 | miR-199a-5p (and other miRNAs w/seed CCAGUGU) | 2.810 | TargetScan Human | High (predicted) | ACP4 | acid phosphatase 4 | Plasma Membrane | phosphatase |
| MIMAT0000263 | miR-199a-5p (and other miRNAs w/seed CCAGUGU) | 2.810 | TargetScan Human | Moderate (predicted) | ACTG1 | actin gamma 1 | Cytoplasm | other |
| MIMAT0000263 | miR-199a-5p (and other miRNAs w/seed CCAGUGU) | 2.810 | TargetScan Human | Moderate (predicted) | ACVR1B | activin A receptor type 1B | Plasma Membrane | kinase |
| MIMAT0000263 | miR-199a-5p (and other miRNAs w/seed CCAGUGU) | 2.810 | TargetScan Human | Moderate (predicted) | ADAP2 | ArfGAP with dual PH domains 2 | Cytoplasm | other |
| MIMAT0000263 | miR-199a-5p (and other miRNAs w/seed CCAGUGU) | 2.810 | TargetScan Human | Moderate (predicted) | ADAT2 | adenosine deaminase tRNA specific 2 | Nucleus | enzyme |
| MIMAT0000263 | miR-199a-5p (and other miRNAs w/seed CCAGUGU) | 2.810 | TargetScan Human | Moderate (predicted) | ADD3 | adducin 3 | Cytoplasm | other |
| MIMAT0000263 | miR-199a-5p (and other miRNAs w/seed CCAGUGU) | 2.810 | TargetScan Human | Moderate (predicted) | AF011889.5 | | Other | other |
| MIMAT0000263 | miR-199a-5p (and other miRNAs w/seed CCAGUGU) | 2.810 | TargetScan Human | Moderate (predicted) | AGAP1 | th GTPase domain, ankyrin repeat and PH | Cytoplasm | enzyme |
| MIMAT0000263 | miR-199a-5p (and other miRNAs w/seed CCAGUGU) | 2.810 | TargetScan Human | Moderate (predicted) | AGGF1 | ogenic factor with G-patch and FHA doma | Cytoplasm | other |
| MIMAT0000263 | miR-199a-5p (and other miRNAs w/seed CCAGUGU) | 2.810 | TargetScan Human | Moderate (predicted) | AIDA | axin interactor, dorsalization associated | Cytoplasm | other |
| MIMAT0000263 | miR-199a-5p (and other miRNAs w/seed CCAGUGU) | 2.810 | TargetScan Human | High (predicted) | AKAP1 | A-kinase anchoring protein 1 | Cytoplasm | other |
| MIMAT0000263 | miR-199a-5p (and other miRNAs w/seed CCAGUGU) | 2.810 | TargetScan Human | Moderate (predicted) | AKAP6 | A-kinase anchoring protein 6 | Nucleus | other |
| MIMAT0000263 | miR-199a-5p (and other miRNAs w/seed CCAGUGU) | 2.810 | TargetScan Human | Moderate (predicted) | AKAP7 | A-kinase anchoring protein 7 | Plasma Membrane | other |
| MIMAT0000263 | miR-199a-5p (and other miRNAs w/seed CCAGUGU) | 2.810 | miRecords | Experimentally Observed | ALOX5AP | achidonate 5-lipoxygenase activating prote | Plasma Membrane | enzyme |
| MIMAT0000263 | miR-199a-5p (and other miRNAs w/seed CCAGUGU) | 2.810 | TargetScan Human | High (predicted) | ALPK2 | alpha kinase 2 | Nucleus | kinase |
| MIMAT0000263 | miR-199a-5p (and other miRNAs w/seed CCAGUGU) | 2.810 | TargetScan Human | High (predicted) | ALS2 | Rho guanine nucleotide exchange factor A | Cytoplasm | other |
| MIMAT0000263 | miR-199a-5p (and other miRNAs w/seed CCAGUGU) | 2.810 | TargetScan Human | Moderate (predicted) | ANGEL1 | angel homolog 1 | Extracellular Space | other |
| MIMAT0000263 | miR-199a-5p (and other miRNAs w/seed CCAGUGU) | 2.810 | TargetScan Human | High (predicted) | ANGEL2 | angel homolog 2 | Nucleus | other |
| MIMAT0000263 | miR-199a-5p (and other miRNAs w/seed CCAGUGU) | 2.810 | TargetScan Human | Moderate (predicted) | ANK3 | ankyrin 3 | Plasma Membrane | other |
| MIMAT0000263 | miR-199a-5p (and other miRNAs w/seed CCAGUGU) | 2.810 | TargetScan Human | Moderate (predicted) | ANO10 | anoctamin 10 | Plasma Membrane | ion channel |
| MIMAT0000263 | miR-199a-5p (and other miRNAs w/seed CCAGUGU) | 2.810 | TargetScan Human | Moderate (predicted) | AOC1 | amine oxidase copper containing 1 | Extracellular Space | enzyme |
| MIMAT0000263 | miR-199a-5p (and other miRNAs w/seed CCAGUGU) | 2.810 | TargetScan Human | High (predicted) | AP1G1 | tor related protein complex 1 subunit gam | Cytoplasm | other |
| MIMAT0000263 | miR-199a-5p (and other miRNAs w/seed CCAGUGU) | 2.810 | TargetScan Human | High (predicted) | APBB1 | beta precursor protein binding family B m | Cytoplasm | transcription regulator |
| MIMAT0000263 | miR-199a-5p (and other miRNAs w/seed CCAGUGU) | 2.810 | TargetScan Human | Moderate (predicted) | APLNR | apelin receptor | Plasma Membrane | G-protein coupled receptor |
| MIMAT0000263 | miR-199a-5p (and other miRNAs w/seed CCAGUGU) | 2.810 | TargetScan Human | Moderate (predicted) | APMAP | ipocyte plasma membrane associated prot | Plasma Membrane | enzyme |
| MIMAT0000263 | miR-199a-5p (and other miRNAs w/seed CCAGUGU) | 2.810 | TargetScan Human | High (predicted) | APOL2 | apolipoprotein L2 | Cytoplasm | other |
| MIMAT0000263 | miR-199a-5p (and other miRNAs w/seed CCAGUGU) | 2.810 | TargetScan Human | Moderate (predicted) | APPBP2 | yloid beta precursor protein binding protei | Cytoplasm | other |
| MIMAT0000263 | miR-199a-5p (and other miRNAs w/seed CCAGUGU) | 2.810 | TargetScan Human | Moderate (predicted) | AQP11 | aquaporin 11 | Plasma Membrane | transporter |
| MIMAT0000263 | miR-199a-5p (and other miRNAs w/seed CCAGUGU) | 2.810 | TargetScan Human | Moderate (predicted) | ARCN1 | archain 1 | Cytoplasm | other |
| MIMAT0000263 | miR-199a-5p (and other miRNAs w/seed CCAGUGU) | 2.810 | TargetScan Human | High (predicted) | ARF6 | ADP ribosylation factor 6 | Plasma Membrane | transporter |
| MIMAT0000263 | miR-199a-5p (and other miRNAs w/seed CCAGUGU) | 2.810 | TargetScan Human | High (predicted) | ARHGAP12 | Rho GTPase activating protein 12 | Cytoplasm | other |
| MIMAT0000263 | miR-199a-5p (and other miRNAs w/seed CCAGUGU) | 2.810 | TargetScan Human | Moderate (predicted) | ARHGAP17 | Rho GTPase activating protein 17 | Cytoplasm | other |
| MIMAT0000263 | miR-199a-5p (and other miRNAs w/seed CCAGUGU) | 2.810 | TargetScan Human | High (predicted) | ARHGAP21 | Rho GTPase activating protein 21 | Cytoplasm | other |
| MIMAT0000263 | miR-199a-5p (and other miRNAs w/seed CCAGUGU) | 2.810 | TargetScan Human | Moderate (predicted) | ARHGEF10 | Rho guanine nucleotide exchange factor 1 | Cytoplasm | enzyme |
| MIMAT0000263 | miR-199a-5p (and other miRNAs w/seed CCAGUGU) | 2.810 | TargetScan Human | Moderate (predicted) | ARHGEF28 | Rho guanine nucleotide exchange factor 2 | Cytoplasm | other |
| MIMAT0000263 | miR-199a-5p (and other miRNAs w/seed CCAGUGU) | 2.810 | TargetScan Human | Moderate (predicted) | ARHGEF39 | Rho guanine nucleotide exchange factor 3 | Plasma Membrane | other |
| MIMAT0000263 | miR-199a-5p (and other miRNAs w/seed CCAGUGU) | 2.810 | TargetScan Human | Moderate (predicted) | ARL14EP | ibosylation factor like GTPase 14 effector | Other | other |
| MIMAT0000263 | miR-199a-5p (and other miRNAs w/seed CCAGUGU) | 2.810 | TargetScan Human | Moderate (predicted) | ARL4A | ADP ribosylation factor like GTPase 4A | Nucleus | enzyme |
| MIMAT0000263 | miR-199a-5p (and other miRNAs w/seed CCAGUGU) | 2.810 | TargetScan Human | Moderate (predicted) | ARRB2 | arrestin beta 2 | Cytoplasm | other |
| MIMAT0000263 | miR-199a-5p (and other miRNAs w/seed CCAGUGU) | 2.810 | TargetScan Human | Moderate (predicted) | ART3 | ADP-ribosyltransferase 3 (inactive) | Plasma Membrane | enzyme |
| MIMAT0000263 | miR-199a-5p (and other miRNAs w/seed CCAGUGU) | 2.810 | TargetScan Human | Moderate (predicted) | ART4 | osyltransferase 4 (inactive) (Dombrock bloo | Nucleus | enzyme |
| MIMAT0000263 | miR-199a-5p (and other miRNAs w/seed CCAGUGU) | 2.810 | TargetScan Human | High (predicted) | ASRGL1 | asparaginase and isoaspartyl peptidase 1 | Cytoplasm | enzyme |
| MIMAT0000263 | miR-199a-5p (and other miRNAs w/seed CCAGUGU) | 2.810 | TargetScan Human | Moderate (predicted) | ATF3 | activating transcription factor 3 | Nucleus | transcription regulator |
| MIMAT0000263 | miR-199a-5p (and other miRNAs w/seed CCAGUGU) | 2.810 | TargetScan Human | Moderate (predicted) | ATG4D | autophagy related 4D cysteine peptidase | Cytoplasm | peptidase |

Sup. Table 3. List of 3699 targets of the seven dysregulated miRNAs obtained from three different target prediction databases,TargetScan TarBase and miRecords considering those predicted as moderate, high, or experimentally validated, respectively.

| | | | | | | | | |
|---|---|---|---|---|---|---|---|---|
| MIMAT0000263 | miR-199a-5p (and other miRNAs w/seed CCAGUGU) | 2.810 | TargetScan Human | Moderate (predicted) | ATP13A2 | ATPase cation transporting 13A2 | Cytoplasm | transporter |
| MIMAT0000263 | miR-199a-5p (and other miRNAs w/seed CCAGUGU) | 2.810 | TargetScan Human | Moderate (predicted) | ATP1B3 | ATPase Na+/K+ transporting subunit beta | Plasma Membrane | transporter |
| MIMAT0000263 | miR-199a-5p (and other miRNAs w/seed CCAGUGU) | 2.810 | TargetScan Human | Moderate (predicted) | ATP6V1C2 | ATPase H+ transporting V1 subunit C2 | Cytoplasm | transporter |
| MIMAT0000263 | miR-199a-5p (and other miRNAs w/seed CCAGUGU) | 2.810 | TargetScan Human | Moderate (predicted) | ATP6V1D | ATPase H+ transporting V1 subunit D | Cytoplasm | transporter |
| MIMAT0000263 | miR-199a-5p (and other miRNAs w/seed CCAGUGU) | 2.810 | TargetScan Human | Moderate (predicted) | ATP9A | Pase phospholipid transporting 9A (putati | Cytoplasm | transporter |
| MIMAT0000263 | miR-199a-5p (and other miRNAs w/seed CCAGUGU) | 2.810 | TargetScan Human | Moderate (predicted) | ATXN7 | ataxin 7 | Nucleus | other |
| MIMAT0000263 | miR-199a-5p (and other miRNAs w/seed CCAGUGU) | 2.810 | TargetScan Human | Moderate (predicted) | ATXN7L1 | ataxin 7 like 1 | Other | other |
| MIMAT0000263 | miR-199a-5p (and other miRNAs w/seed CCAGUGU) | 2.810 | TargetScan Human | Moderate (predicted) | AVPR1B | arginine vasopressin receptor 1B | Plasma Membrane | G-protein coupled receptor |
| MIMAT0000263 | miR-199a-5p (and other miRNAs w/seed CCAGUGU) | 2.810 | TargetScan Human | Moderate (predicted) | B4GALT4 | beta-1,4-galactosyltransferase 4 | Cytoplasm | enzyme |
| MIMAT0000263 | miR-199a-5p (and other miRNAs w/seed CCAGUGU) | 2.810 | TargetScan Human | High (predicted) | B4GAT1 | beta-1,4-glucuronyltransferase 1 | Cytoplasm | enzyme |
| MIMAT0000263 | miR-199a-5p (and other miRNAs w/seed CCAGUGU) | 2.810 | TargetScan Human | High (predicted) | BAAT | bile acid-CoA:amino acid N-acyltransferas | Cytoplasm | enzyme |
| MIMAT0000263 | miR-199a-5p (and other miRNAs w/seed CCAGUGU) | 2.810 | TargetScan Human | Moderate (predicted) | BAG1 | BAG cochaperone 1 | Cytoplasm | other |
| MIMAT0000263 | miR-199a-5p (and other miRNAs w/seed CCAGUGU) | 2.810 | TargetScan Human | High (predicted) | BCAM | cell adhesion molecule (Lutheran blood g | Plasma Membrane | transmembrane receptor |
| MIMAT0000263 | miR-199a-5p (and other miRNAs w/seed CCAGUGU) | 2.810 | TargetScan Human | Moderate (predicted) | BCAP29 | B cell receptor associated protein 29 | Cytoplasm | transporter |
| MIMAT0000263 | miR-199a-5p (and other miRNAs w/seed CCAGUGU) | 2.810 | TargetScan Human | Moderate (predicted) | BCL2 | BCL2 apoptosis regulator | Cytoplasm | transporter |
| MIMAT0000263 | miR-199a-5p (and other miRNAs w/seed CCAGUGU) | 2.810 | TargetScan Human | Moderate (predicted) | BECN1 | beclin 1 | Cytoplasm | other |
| MIMAT0000263 | miR-199a-5p (and other miRNAs w/seed CCAGUGU) | 2.810 | TargetScan Human | Moderate (predicted) | BEND3 | BEN domain containing 3 | Nucleus | other |
| MIMAT0000263 | miR-199a-5p (and other miRNAs w/seed CCAGUGU) | 2.810 | TargetScan Human | High (predicted) | BEND7 | BEN domain containing 7 | Cytoplasm | other |
| MIMAT0000263 | miR-199a-5p (and other miRNAs w/seed CCAGUGU) | 2.810 | TargetScan Human | Moderate (predicted) | BHMT | betaine--homocysteine S-methyltransferas | Cytoplasm | enzyme |
| MIMAT0000263 | miR-199a-5p (and other miRNAs w/seed CCAGUGU) | 2.810 | TargetScan Human | High (predicted) | BICC1 | BicC family RNA binding protein 1 | Other | other |
| MIMAT0000263 | miR-199a-5p (and other miRNAs w/seed CCAGUGU) | 2.810 | TargetScan Human | Moderate (predicted) | BICD1 | BICD cargo adaptor 1 | Cytoplasm | other |
| MIMAT0000263 | miR-199a-5p (and other miRNAs w/seed CCAGUGU) | 2.810 | TargetScan Human | High (predicted) | BPIFC | BPI fold containing family C | Extracellular Space | transporter |
| MIMAT0000263 | miR-199a-5p (and other miRNAs w/seed CCAGUGU) | 2.810 | TargetScan Human | Moderate (predicted) | BROX | BRO1 domain and CAAX motif containing | Cytoplasm | other |
| MIMAT0000263 | miR-199a-5p (and other miRNAs w/seed CCAGUGU) | 2.810 | TargetScan Human | Moderate (predicted) | BTBD3 | BTB domain containing 3 | Other | other |
| MIMAT0000263 | miR-199a-5p (and other miRNAs w/seed CCAGUGU) | 2.810 | TargetScan Human | Moderate (predicted) | BTC | betacellulin | Extracellular Space | growth factor |
| MIMAT0000263 | miR-199a-5p (and other miRNAs w/seed CCAGUGU) | 2.810 | TargetScan Human | Moderate (predicted) | BTLA | B and T lymphocyte associated | Plasma Membrane | other |
| MIMAT0000263 | miR-199a-5p (and other miRNAs w/seed CCAGUGU) | 2.810 | TargetScan Human | Moderate (predicted) | BTRC | sducin repeat containing E3 ubiquitin prot | Cytoplasm | enzyme |
| MIMAT0000263 | miR-199a-5p (and other miRNAs w/seed CCAGUGU) | 2.810 | TargetScan Human | Moderate (predicted) | C11orf54 | chromosome 11 open reading frame 54 | Nucleus | enzyme |
| MIMAT0000263 | miR-199a-5p (and other miRNAs w/seed CCAGUGU) | 2.810 | TargetScan Human | Moderate (predicted) | C17orf102 | chromosome 17 open reading frame 102 | Other | other |
| MIMAT0000263 | miR-199a-5p (and other miRNAs w/seed CCAGUGU) | 2.810 | TargetScan Human | High (predicted) | C1GALT1 | coprotein-N-acetylgalactosamine 3-beta-ga | Plasma Membrane | enzyme |
| MIMAT0000263 | miR-199a-5p (and other miRNAs w/seed CCAGUGU) | 2.810 | TargetScan Human | Moderate (predicted) | C1orf105 | chromosome 1 open reading frame 105 | Other | other |
| MIMAT0000263 | miR-199a-5p (and other miRNAs w/seed CCAGUGU) | 2.810 | TargetScan Human | Moderate (predicted) | C1orf21 | chromosome 1 open reading frame 21 | Other | other |
| MIMAT0000263 | miR-199a-5p (and other miRNAs w/seed CCAGUGU) | 2.810 | TargetScan Human | High (predicted) | C1QL3 | complement C1q like 3 | Extracellular Space | other |
| MIMAT0000263 | miR-199a-5p (and other miRNAs w/seed CCAGUGU) | 2.810 | TargetScan Human | Moderate (predicted) | C1QTNF6 | C1q and TNF related 6 | Extracellular Space | other |
| MIMAT0000263 | miR-199a-5p (and other miRNAs w/seed CCAGUGU) | 2.810 | TargetScan Human | Moderate (predicted) | C20orf173 | chromosome 20 open reading frame 173 | Other | other |
| MIMAT0000263 | miR-199a-5p (and other miRNAs w/seed CCAGUGU) | 2.810 | TargetScan Human | Moderate (predicted) | C20orf197 | omosome 20 putative open reading frame | Other | other |
| MIMAT0000263 | miR-199a-5p (and other miRNAs w/seed CCAGUGU) | 2.810 | TargetScan Human | Moderate (predicted) | C5orf64 | romosome 5 putative open reading frame | Other | other |
| MIMAT0000263 | miR-199a-5p (and other miRNAs w/seed CCAGUGU) | 2.810 | TargetScan Human | High (predicted) | C5orf67 | romosome 5 putative open reading frame | Other | other |
| MIMAT0000263 | miR-199a-5p (and other miRNAs w/seed CCAGUGU) | 2.810 | TargetScan Human | Moderate (predicted) | C8orf34-AS1 | C8orf34 antisense RNA 1 | Other | other |
| MIMAT0000263 | miR-199a-5p (and other miRNAs w/seed CCAGUGU) | 2.810 | TargetScan Human | High (predicted) | C8orf88 | chromosome 8 open reading frame 88 | Other | other |
| MIMAT0000263 | miR-199a-5p (and other miRNAs w/seed CCAGUGU) | 2.810 | TargetScan Human | Moderate (predicted) | CABLES2 | Cdk5 and Abl enzyme substrate 2 | Other | other |
| MIMAT0000263 | miR-199a-5p (and other miRNAs w/seed CCAGUGU) | 2.810 | TargetScan Human | Moderate (predicted) | CACNA1S | cium voltage-gated channel subunit alpha | Plasma Membrane | ion channel |
| MIMAT0000263 | miR-199a-5p (and other miRNAs w/seed CCAGUGU) | 2.810 | TargetScan Human | High (predicted) | CACNB2 | m voltage-gated channel auxiliary subunit | Plasma Membrane | ion channel |
| MIMAT0000263 | miR-199a-5p (and other miRNAs w/seed CCAGUGU) | 2.810 | TargetScan Human | High (predicted) | CACUL1 | CDK2 associated cullin domain 1 | Other | other |
| MIMAT0000263 | miR-199a-5p (and other miRNAs w/seed CCAGUGU) | 2.810 | TargetScan Human | Moderate (predicted) | CAP1 | associated actin cytoskeleton regulatory p | Plasma Membrane | other |
| MIMAT0000263 | miR-199a-5p (and other miRNAs w/seed CCAGUGU) | 2.810 | TargetScan Human | Moderate (predicted) | CAPRIN1 | cell cycle associated protein 1 | Plasma Membrane | other |
| MIMAT0000263 | miR-199a-5p (and other miRNAs w/seed CCAGUGU) | 2.810 | TargetScan Human | Moderate (predicted) | CARHSP1 | calcium regulated heat stable protein 1 | Cytoplasm | transcription regulator |
| MIMAT0000263 | miR-199a-5p (and other miRNAs w/seed CCAGUGU) | 2.810 | TargetScan Human | Moderate (predicted) | CARNMT1 | carnosine N-methyltransferase 1 | Cytoplasm | enzyme |
| MIMAT0000263 | miR-199a-5p (and other miRNAs w/seed CCAGUGU) | 2.810 | TargetScan Human | Moderate (predicted) | CAT | catalase | Cytoplasm | enzyme |
| MIMAT0000263 | miR-199a-5p (and other miRNAs w/seed CCAGUGU) | 2.810 | TargetScan Human | Moderate (predicted) | CBLIF | cobalamin binding intrinsic factor | Extracellular Space | transporter |
| MIMAT0000263 | miR-199a-5p (and other miRNAs w/seed CCAGUGU) | 2.810 | TargetScan Human | Moderate (predicted) | CCDC103 | coiled-coil domain containing 103 | Cytoplasm | other |
| MIMAT0000263 | miR-199a-5p (and other miRNAs w/seed CCAGUGU) | 2.810 | TargetScan Human | High (predicted) | CCDC120 | coiled-coil domain containing 120 | Plasma Membrane | other |
| MIMAT0000263 | miR-199a-5p (and other miRNAs w/seed CCAGUGU) | 2.810 | TargetScan Human | Moderate (predicted) | CCDC15 | coiled-coil domain containing 15 | Cytoplasm | other |
| MIMAT0000263 | miR-199a-5p (and other miRNAs w/seed CCAGUGU) | 2.810 | TargetScan Human | High (predicted) | CCDC152 | coiled-coil domain containing 152 | Other | other |
| MIMAT0000263 | miR-199a-5p (and other miRNAs w/seed CCAGUGU) | 2.810 | TargetScan Human | Moderate (predicted) | CCDC32 | coiled-coil domain containing 32 | Other | other |
| MIMAT0000263 | miR-199a-5p (and other miRNAs w/seed CCAGUGU) | 2.810 | TargetScan Human | High (predicted) | CCDC43 | coiled-coil domain containing 43 | Cytoplasm | other |
| MIMAT0000263 | miR-199a-5p (and other miRNAs w/seed CCAGUGU) | 2.810 | TargetScan Human | Moderate (predicted) | CCDC88C | coiled-coil domain containing 88C | Cytoplasm | other |
| MIMAT0000263 | miR-199a-5p (and other miRNAs w/seed CCAGUGU) | 2.810 | TargetScan Human | Moderate (predicted) | CCDC91 | coiled-coil domain containing 91 | Cytoplasm | other |
| MIMAT0000263 | miR-199a-5p (and other miRNAs w/seed CCAGUGU) | 2.810 | TargetScan Human | Moderate (predicted) | CCL20 | C-C motif chemokine ligand 20 | Extracellular Space | cytokine |
| MIMAT0000263 | miR-199a-5p (and other miRNAs w/seed CCAGUGU) | 2.810 | TargetScan Human | Moderate (predicted) | CCL28 | C-C motif chemokine ligand 28 | Extracellular Space | cytokine |
| MIMAT0000263 | miR-199a-5p (and other miRNAs w/seed CCAGUGU) | 2.810 | TargetScan Human | High (predicted) | CCNJ | cyclin J | Nucleus | other |
| MIMAT0000263 | miR-199a-5p (and other miRNAs w/seed CCAGUGU) | 2.810 | TargetScan Human | High (predicted) | CCNL1 | cyclin L1 | Nucleus | other |
| MIMAT0000263 | miR-199a-5p (and other miRNAs w/seed CCAGUGU) | 2.810 | TargetScan Human | High (predicted) | CCSAP | entriole, cilia and spindle associated prote | Cytoplasm | other |
| MIMAT0000263 | miR-199a-5p (and other miRNAs w/seed CCAGUGU) | 2.810 | TargetScan Human | Moderate (predicted) | CCT6A | chaperonin containing TCP1 subunit 6A | Cytoplasm | other |
| MIMAT0000263 | miR-199a-5p (and other miRNAs w/seed CCAGUGU) | 2.810 | TargetScan Human | Moderate (predicted) | CCZ1/CCZ1B | B, vacuolar protein trafficking and biogen | Cytoplasm | other |
| MIMAT0000263 | miR-199a-5p (and other miRNAs w/seed CCAGUGU) | 2.810 | TargetScan Human | Moderate (predicted) | CD207 | CD207 molecule | Plasma Membrane | other |
| MIMAT0000263 | miR-199a-5p (and other miRNAs w/seed CCAGUGU) | 2.810 | TargetScan Human | High (predicted) | CD22 | CD22 molecule | Plasma Membrane | transmembrane receptor |
| MIMAT0000263 | miR-199a-5p (and other miRNAs w/seed CCAGUGU) | 2.810 | TargetScan Human | Moderate (predicted) | CD274 | CD274 molecule | Plasma Membrane | enzyme |
| MIMAT0000263 | miR-199a-5p (and other miRNAs w/seed CCAGUGU) | 2.810 | TargetScan Human | Moderate (predicted) | CD5L | CD5 molecule like | Plasma Membrane | transmembrane receptor |
| MIMAT0000263 | miR-199a-5p (and other miRNAs w/seed CCAGUGU) | 2.810 | TargetScan Human | High (predicted) | CD83 | CD83 molecule | Plasma Membrane | transmembrane receptor |
| MIMAT0000263 | miR-199a-5p (and other miRNAs w/seed CCAGUGU) | 2.810 | TargetScan Human | Moderate (predicted) | CD99L2 | CD99 molecule like 2 | Plasma Membrane | other |
| MIMAT0000263 | miR-199a-5p (and other miRNAs w/seed CCAGUGU) | 2.810 | TargetScan Human | Moderate (predicted) | CDC7 | cell division cycle 7 | Nucleus | kinase |
| MIMAT0000263 | miR-199a-5p (and other miRNAs w/seed CCAGUGU) | 2.810 | TargetScan Human | High (predicted) | CDCA7L | cell division cycle associated 7 like | Nucleus | other |
| MIMAT0000263 | miR-199a-5p (and other miRNAs w/seed CCAGUGU) | 2.810 | TargetScan Human | Moderate (predicted) | CDH17 | cadherin 17 | Plasma Membrane | transporter |
| MIMAT0000263 | miR-199a-5p (and other miRNAs w/seed CCAGUGU) | 2.810 | TargetScan Human | Moderate (predicted) | CDK14 | cyclin dependent kinase 14 | Nucleus | kinase |
| MIMAT0000263 | miR-199a-5p (and other miRNAs w/seed CCAGUGU) | 2.810 | TargetScan Human | Moderate (predicted) | CDKN1B | cyclin dependent kinase inhibitor 1B | Nucleus | kinase |
| MIMAT0000263 | miR-199a-5p (and other miRNAs w/seed CCAGUGU) | 2.810 | TargetScan Human | Moderate (predicted) | CDKN1C | cyclin dependent kinase inhibitor 1C | Nucleus | other |
| MIMAT0000263 | miR-199a-5p (and other miRNAs w/seed CCAGUGU) | 2.810 | TargetScan Human | High (predicted) | CDKN2AIP | CDKN2A interacting protein | Nucleus | transcription regulator |
| MIMAT0000263 | miR-199a-5p (and other miRNAs w/seed CCAGUGU) | 2.810 | TargetScan Human | Moderate (predicted) | CEACAM3 | CEA cell adhesion molecule 3 | Other | other |
| MIMAT0000263 | miR-199a-5p (and other miRNAs w/seed CCAGUGU) | 2.810 | TargetScan Human | Moderate (predicted) | CECR2 | CECR2 histone acetyl-lysine reader | Nucleus | other |
| MIMAT0000263 | miR-199a-5p (and other miRNAs w/seed CCAGUGU) | 2.810 | TargetScan Human | Moderate (predicted) | CELF2 | CUGBP Elav-like family member 2 | Nucleus | other |
| MIMAT0000263 | miR-199a-5p (and other miRNAs w/seed CCAGUGU) | 2.810 | TargetScan Human | High (predicted) | CELSR1 | herin EGF LAG seven-pass G-type recept | Plasma Membrane | G-protein coupled receptor |
| MIMAT0000263 | miR-199a-5p (and other miRNAs w/seed CCAGUGU) | 2.810 | TargetScan Human | High (predicted) | CENPI | centromere protein I | Nucleus | other |
| MIMAT0000263 | miR-199a-5p (and other miRNAs w/seed CCAGUGU) | 2.810 | TargetScan Human | Moderate (predicted) | CFI | complement factor I | Extracellular Space | peptidase |
| MIMAT0000263 | miR-199a-5p (and other miRNAs w/seed CCAGUGU) | 2.810 | TargetScan Human | Moderate (predicted) | CHCHD4 | d-coil-helix-coiled-coil-helix domain contair | Cytoplasm | enzyme |
| MIMAT0000263 | miR-199a-5p (and other miRNAs w/seed CCAGUGU) | 2.810 | TargetScan Human | Moderate (predicted) | CHN2 | chimerin 2 | Cytoplasm | other |
| MIMAT0000263 | miR-199a-5p (and other miRNAs w/seed CCAGUGU) | 2.810 | TargetScan Human | Moderate (predicted) | CHRM1 | cholinergic receptor muscarinic 1 | Plasma Membrane | G-protein coupled receptor |
| MIMAT0000263 | miR-199a-5p (and other miRNAs w/seed CCAGUGU) | 2.810 | TargetScan Human | Moderate (predicted) | CLCN3 | chloride voltage-gated channel 3 | Plasma Membrane | ion channel |
| MIMAT0000263 | miR-199a-5p (and other miRNAs w/seed CCAGUGU) | 2.810 | TargetScan Human | High (predicted) | CLDN22 | claudin 22 | Plasma Membrane | other |
| MIMAT0000263 | miR-199a-5p (and other miRNAs w/seed CCAGUGU) | 2.810 | TargetScan Human | Moderate (predicted) | CLEC1B | C-type lectin domain family 1 member B | Plasma Membrane | transmembrane receptor |
| MIMAT0000263 | miR-199a-5p (and other miRNAs w/seed CCAGUGU) | 2.810 | TargetScan Human | Moderate (predicted) | CLEC2A | C-type lectin domain family 2 member A | Plasma Membrane | other |
| MIMAT0000263 | miR-199a-5p (and other miRNAs w/seed CCAGUGU) | 2.810 | TargetScan Human | Moderate (predicted) | CLEC2D | C-type lectin domain family 2 member D | Plasma Membrane | transmembrane receptor |
| MIMAT0000263 | miR-199a-5p (and other miRNAs w/seed CCAGUGU) | 2.810 | TargetScan Human | Moderate (predicted) | CLEC5A | C-type lectin domain containing 5A | Plasma Membrane | other |
| MIMAT0000263 | miR-199a-5p (and other miRNAs w/seed CCAGUGU) | 2.810 | TargetScan Human | Moderate (predicted) | CLECL1 | C-type lectin like 1 | Plasma Membrane | other |
| MIMAT0000263 | miR-199a-5p (and other miRNAs w/seed CCAGUGU) | 2.810 | TargetScan Human | Moderate (predicted) | CLIP1 | CAP-Gly domain containing linker protein | Cytoplasm | other |
| MIMAT0000263 | miR-199a-5p (and other miRNAs w/seed CCAGUGU) | 2.810 | TargetScan Human | Moderate (predicted) | CLK2 | CDC like kinase 2 | Nucleus | kinase |
| MIMAT0000263 | miR-199a-5p (and other miRNAs w/seed CCAGUGU) | 2.810 | TargetScan Human | Moderate (predicted) | CLOCK | clock circadian regulator | Nucleus | transcription regulator |
| MIMAT0000263 | miR-199a-5p (and other miRNAs w/seed CCAGUGU) | 2.810 | TargetScan Human | High (predicted) | CLTC | clathrin heavy chain | Plasma Membrane | other |
| MIMAT0000263 | miR-199a-5p (and other miRNAs w/seed CCAGUGU) | 2.810 | TargetScan Human | Moderate (predicted) | CMSS1 | cms1 ribosomal small subunit homolog | Other | other |
| MIMAT0000263 | miR-199a-5p (and other miRNAs w/seed CCAGUGU) | 2.810 | TargetScan Human | Moderate (predicted) | CNN1 | calponin 1 | Cytoplasm | other |
| MIMAT0000263 | miR-199a-5p (and other miRNAs w/seed CCAGUGU) | 2.810 | TargetScan Human | Moderate (predicted) | CNPY2 | canopy FGF signaling regulator 2 | Plasma Membrane | other |
| MIMAT0000263 | miR-199a-5p (and other miRNAs w/seed CCAGUGU) | 2.810 | TargetScan Human | Moderate (predicted) | CNPY4 | canopy FGF signaling regulator 4 | Other | other |
| MIMAT0000263 | miR-199a-5p (and other miRNAs w/seed CCAGUGU) | 2.810 | TargetScan Human | Moderate (predicted) | COL5A3 | collagen type V alpha 3 chain | Extracellular Space | other |
| MIMAT0000263 | miR-199a-5p (and other miRNAs w/seed CCAGUGU) | 2.810 | TargetScan Human | High (predicted) | COMT | catechol-O-methyltransferase | Cytoplasm | enzyme |
| MIMAT0000263 | miR-199a-5p (and other miRNAs w/seed CCAGUGU) | 2.810 | TargetScan Human | Moderate (predicted) | COX15 | ochrome c oxidase assembly homolog CO | Cytoplasm | enzyme |
| MIMAT0000263 | miR-199a-5p (and other miRNAs w/seed CCAGUGU) | 2.810 | TargetScan Human | Moderate (predicted) | CRB1 | crumbs cell polarity complex component 1 | Plasma Membrane | other |
| MIMAT0000263 | miR-199a-5p (and other miRNAs w/seed CCAGUGU) | 2.810 | TargetScan Human | High (predicted) | CRCT1 | cysteine rich C-terminal 1 | Other | other |
| MIMAT0000263 | miR-199a-5p (and other miRNAs w/seed CCAGUGU) | 2.810 | TargetScan Human | Moderate (predicted) | CREBRF | CREB3 regulatory factor | Nucleus | transcription regulator |
| MIMAT0000263 | miR-199a-5p (and other miRNAs w/seed CCAGUGU) | 2.810 | TargetScan Human | Moderate (predicted) | CRP | C-reactive protein | Extracellular Space | other |
| MIMAT0000263 | miR-199a-5p (and other miRNAs w/seed CCAGUGU) | 2.810 | TargetScan Human | High (predicted) | CRYBG3 | crystallin beta-gamma domain containing 3 | Other | other |
| MIMAT0000263 | miR-199a-5p (and other miRNAs w/seed CCAGUGU) | 2.810 | TargetScan Human | Moderate (predicted) | CSDC2 | cold shock domain containing C2 | Cytoplasm | other |
| MIMAT0000263 | miR-199a-5p (and other miRNAs w/seed CCAGUGU) | 2.810 | TargetScan Human | High (predicted) | CSGALNACT1 | roitin sulfate N-acetylgalactosaminyltransfe | Cytoplasm | enzyme |
| MIMAT0000263 | miR-199a-5p (and other miRNAs w/seed CCAGUGU) | 2.810 | TargetScan Human | High (predicted) | CT55 | cancer/testis antigen 55 | Other | other |
| MIMAT0000263 | miR-199a-5p (and other miRNAs w/seed CCAGUGU) | 2.810 | TargetScan Human | High (predicted) | CTD-2006C1.13 | | Other | other |
| MIMAT0000263 | miR-199a-5p (and other miRNAs w/seed CCAGUGU) | 2.810 | TargetScan Human | Moderate (predicted) | CTSL | cathepsin L | Cytoplasm | peptidase |
| MIMAT0000263 | miR-199a-5p (and other miRNAs w/seed CCAGUGU) | 2.810 | TargetScan Human | High (predicted) | CTU2 | cytosolic thiouridylase subunit 2 | Cytoplasm | other |
| MIMAT0000263 | miR-199a-5p (and other miRNAs w/seed CCAGUGU) | 2.810 | TargetScan Human | Moderate (predicted) | CXCL16 | C-X-C motif chemokine ligand 16 | Extracellular Space | cytokine |
| MIMAT0000263 | miR-199a-5p (and other miRNAs w/seed CCAGUGU) | 2.810 | TargetScan Human | Moderate (predicted) | CXCL6 | C-X-C motif chemokine ligand 6 | Extracellular Space | cytokine |
| MIMAT0000263 | miR-199a-5p (and other miRNAs w/seed CCAGUGU) | 2.810 | TargetScan Human | High (predicted) | CYB561 | cytochrome b561 | Cytoplasm | enzyme |
| MIMAT0000263 | miR-199a-5p (and other miRNAs w/seed CCAGUGU) | 2.810 | TargetScan Human | Moderate (predicted) | CYLC2 | cylicin 2 | Other | other |
| MIMAT0000263 | miR-199a-5p (and other miRNAs w/seed CCAGUGU) | 2.810 | TargetScan Human | Moderate (predicted) | CYP2U1 | ochrome P450 family 2 subfamily U memb | Cytoplasm | enzyme |
| MIMAT0000263 | miR-199a-5p (and other miRNAs w/seed CCAGUGU) | 2.810 | TargetScan Human | High (predicted) | CYP51A1 | chrome P450 family 51 subfamily A memb | Cytoplasm | enzyme |
| MIMAT0000263 | miR-199a-5p (and other miRNAs w/seed CCAGUGU) | 2.810 | TargetScan Human | High (predicted) | CYP7B1 | ochrome P450 family 7 subfamily B memb | Cytoplasm | enzyme |
| MIMAT0000263 | miR-199a-5p (and other miRNAs w/seed CCAGUGU) | 2.810 | TargetScan Human | Moderate (predicted) | CYREN | cell cycle regulator of NHEJ | Nucleus | other |
| MIMAT0000263 | miR-199a-5p (and other miRNAs w/seed CCAGUGU) | 2.810 | TargetScan Human | Moderate (predicted) | DAW1 | dynein assembly factor with WD repeats 1 | Extracellular Space | other |
| MIMAT0000263 | miR-199a-5p (and other miRNAs w/seed CCAGUGU) | 2.810 | TargetScan Human | High (predicted) | DAZ1/DAZ4 | deleted in azoospermia 1 | Cytoplasm | translation regulator |
| MIMAT0000263 | miR-199a-5p (and other miRNAs w/seed CCAGUGU) | 2.810 | TargetScan Human | High (predicted) | DCAF8L1 | DDB1 and CUL4 associated factor 8 like 1 | Other | other |
| MIMAT0000263 | miR-199a-5p (and other miRNAs w/seed CCAGUGU) | 2.810 | TargetScan Human | Moderate (predicted) | DDI2 | DNA damage inducible 1 homolog 2 | Plasma Membrane | transporter |
| MIMAT0000263 | miR-199a-5p (and other miRNAs w/seed CCAGUGU) | 2.810 | TargetScan Human | High (predicted) | DDR1 | discoidin domain receptor tyrosine kinase | Plasma Membrane | kinase |
| MIMAT0000263 | miR-199a-5p (and other miRNAs w/seed CCAGUGU) | 2.810 | TargetScan Human | Moderate (predicted) | DDX3Y | DEAD-box helicase 3 Y-linked | Cytoplasm | enzyme |
| MIMAT0000263 | miR-199a-5p (and other miRNAs w/seed CCAGUGU) | 2.810 | TargetScan Human | Moderate (predicted) | DENND2C | DENN domain containing 2C | Nucleus | other |
| MIMAT0000263 | miR-199a-5p (and other miRNAs w/seed CCAGUGU) | 2.810 | TargetScan Human | Moderate (predicted) | DENND6A | DENN domain containing 6A | Cytoplasm | other |
| MIMAT0000263 | miR-199a-5p (and other miRNAs w/seed CCAGUGU) | 2.810 | TargetScan Human | Moderate (predicted) | DEPDC1B | DEP domain containing 1B | Cytoplasm | other |
| MIMAT0000263 | miR-199a-5p (and other miRNAs w/seed CCAGUGU) | 2.810 | TargetScan Human | Moderate (predicted) | DERL2 | derlin 2 | Cytoplasm | other |
| MIMAT0000263 | miR-199a-5p (and other miRNAs w/seed CCAGUGU) | 2.810 | TargetScan Human | Moderate (predicted) | GCR6/LOC1027247 | DiGeorge syndrome critical region gene 6 | Nucleus | other |
| MIMAT0000263 | miR-199a-5p (and other miRNAs w/seed CCAGUGU) | 2.810 | TargetScan Human | Moderate (predicted) | DGLUCY | D-glutamate cyclase | Cytoplasm | enzyme |
| MIMAT0000263 | miR-199a-5p (and other miRNAs w/seed CCAGUGU) | 2.810 | TargetScan Human | Moderate (predicted) | DHRS7 | dehydrogenase/reductase 7 | Other | enzyme |
| MIMAT0000263 | miR-199a-5p (and other miRNAs w/seed CCAGUGU) | 2.810 | TargetScan Human | Moderate (predicted) | DMRT1 | blesex and mab-3 related transcription fac | Nucleus | transcription regulator |
| MIMAT0000263 | miR-199a-5p (and other miRNAs w/seed CCAGUGU) | 2.810 | TargetScan Human | High (predicted) | DNAH7 | dynein axonemal heavy chain 7 | Cytoplasm | other |
| MIMAT0000263 | miR-199a-5p (and other miRNAs w/seed CCAGUGU) | 2.810 | TargetScan Human | Moderate (predicted) | DNAJB5 | J heat shock protein family (Hsp40) memb | Cytoplasm | other |
| MIMAT0000263 | miR-199a-5p (and other miRNAs w/seed CCAGUGU) | 2.810 | TargetScan Human | Moderate (predicted) | DNAJC3 | J heat shock protein family (Hsp40) memb | Cytoplasm | other |
| MIMAT0000263 | miR-199a-5p (and other miRNAs w/seed CCAGUGU) | 2.810 | TargetScan Human | High (predicted) | DOCK8-AS1 | DOCK8 antisense RNA 1 | Other | other |
| MIMAT0000263 | miR-199a-5p (and other miRNAs w/seed CCAGUGU) | 2.810 | TargetScan Human | High (predicted) | DPP8 | dipeptidyl peptidase 8 | Cytoplasm | peptidase |
| MIMAT0000263 | miR-199a-5p (and other miRNAs w/seed CCAGUGU) | 2.810 | TargetScan Human | Moderate (predicted) | DRAM1 | NA damage regulated autophagy modulato | Cytoplasm | other |
| MIMAT0000263 | miR-199a-5p (and other miRNAs w/seed CCAGUGU) | 2.810 | TargetScan Human | High (predicted) | DTNBP1 | dystrobrevin binding protein 1 | Plasma Membrane | other |
| MIMAT0000263 | miR-199a-5p (and other miRNAs w/seed CCAGUGU) | 2.810 | TargetScan Human | Moderate (predicted) | DUSP14 | dual specificity phosphatase 14 | Other | phosphatase |

Sup. Table 3. List of 3699 targets of the seven dysregulated miRNAs obtained from three different target prediction databases,TargetScan TarBase and miRecords considering those predicted as moderate, high, or experimentally validated, respectively.

| | | | | | | | | |
|---|---|---|---|---|---|---|---|---|
| MIMAT0000263 | miR-199a-5p (and other miRNAs w/seed CCAGUGU) | 2.810 | TargetScan Human | Moderate (predicted) | DYNLRB2 | dynein light chain roadblock-type 2 | Cytoplasm | other |
| MIMAT0000263 | miR-199a-5p (and other miRNAs w/seed CCAGUGU) | 2.810 | Expert Findings,TargetSc | ntally Observed,Moderate ( | DYRK1A | cificity tyrosine phosphorylation regulated k | Nucleus | kinase |
| MIMAT0000263 | miR-199a-5p (and other miRNAs w/seed CCAGUGU) | 2.810 | TargetScan Human | Moderate (predicted) | E2F6 | E2F transcription factor 6 | Nucleus | transcription regulator |
| MIMAT0000263 | miR-199a-5p (and other miRNAs w/seed CCAGUGU) | 2.810 | TargetScan Human | High (predicted) | ECE1 | endothelin converting enzyme 1 | Plasma Membrane | peptidase |
| MIMAT0000263 | miR-199a-5p (and other miRNAs w/seed CCAGUGU) | 2.810 | TargetScan Human | Moderate (predicted) | EEF1A1 | karyotic translation elongation factor 1 alph | Cytoplasm | translation regulator |
| MIMAT0000263 | miR-199a-5p (and other miRNAs w/seed CCAGUGU) | 2.810 | TargetScan Human | Moderate (predicted) | EGF | epidermal growth factor | Extracellular Space | growth factor |
| MIMAT0000263 | miR-199a-5p (and other miRNAs w/seed CCAGUGU) | 2.810 | TargetScan Human | Moderate (predicted) | EHD4 | EH domain containing 4 | Plasma Membrane | enzyme |
| MIMAT0000263 | miR-199a-5p (and other miRNAs w/seed CCAGUGU) | 2.810 | TargetScan Human | Moderate (predicted) | EIF4H | eukaryotic translation initiation factor 4H | Cytoplasm | translation regulator |
| MIMAT0000263 | miR-199a-5p (and other miRNAs w/seed CCAGUGU) | 2.810 | TargetScan Human | Moderate (predicted) | EIF5B | eukaryotic translation initiation factor 5B | Cytoplasm | translation regulator |
| MIMAT0000263 | miR-199a-5p (and other miRNAs w/seed CCAGUGU) | 2.810 | TargetScan Human | Moderate (predicted) | ELOVL7 | ELOVL fatty acid elongase 7 | Cytoplasm | enzyme |
| MIMAT0000263 | miR-199a-5p (and other miRNAs w/seed CCAGUGU) | 2.810 | TargetScan Human | Moderate (predicted) | EMC10 | ER membrane protein complex subunit 10 | Cytoplasm | other |
| MIMAT0000263 | miR-199a-5p (and other miRNAs w/seed CCAGUGU) | 2.810 | TargetScan Human | Moderate (predicted) | EMC7 | ER membrane protein complex subunit 7 | Cytoplasm | other |
| MIMAT0000263 | miR-199a-5p (and other miRNAs w/seed CCAGUGU) | 2.810 | TargetScan Human | Moderate (predicted) | EML5 | EMAP like 5 | Cytoplasm | other |
| MIMAT0000263 | miR-199a-5p (and other miRNAs w/seed CCAGUGU) | 2.810 | TargetScan Human | High (predicted) | ENO3 | enolase 3 | Cytoplasm | enzyme |
| MIMAT0000263 | miR-199a-5p (and other miRNAs w/seed CCAGUGU) | 2.810 | TargetScan Human | Moderate (predicted) | ENTPD7 | nucleoside triphosphate diphosphohydrola | Cytoplasm | enzyme |
| MIMAT0000263 | miR-199a-5p (and other miRNAs w/seed CCAGUGU) | 2.810 | TargetScan Human | Moderate (predicted) | EPAS1 | endothelial PAS domain protein 1 | Nucleus | transcription regulator |
| MIMAT0000263 | miR-199a-5p (and other miRNAs w/seed CCAGUGU) | 2.810 | TargetScan Human | High (predicted) | EPB41L1 | rythrocyte membrane protein band 4.1 like | Plasma Membrane | other |
| MIMAT0000263 | miR-199a-5p (and other miRNAs w/seed CCAGUGU) | 2.810 | TargetScan Human | Moderate (predicted) | EPB41L3 | rythrocyte membrane protein band 4.1 like | Plasma Membrane | other |
| MIMAT0000263 | miR-199a-5p (and other miRNAs w/seed CCAGUGU) | 2.810 | TargetScan Human | Moderate (predicted) | EPHA7 | EPH receptor A7 | Plasma Membrane | kinase |
| MIMAT0000263 | miR-199a-5p (and other miRNAs w/seed CCAGUGU) | 2.810 | TargetScan Human | High (predicted) | ERICH3 | glutamate rich 3 | Other | other |
| MIMAT0000263 | miR-199a-5p (and other miRNAs w/seed CCAGUGU) | 2.810 | TargetScan Human | Moderate (predicted) | ERLIN1 | ER lipid raft associated 1 | Plasma Membrane | other |
| MIMAT0000263 | miR-199a-5p (and other miRNAs w/seed CCAGUGU) | 2.810 | TargetScan Human | Moderate (predicted) | ERMP1 | endoplasmic reticulum metallopeptidase 1 | Cytoplasm | other |
| MIMAT0000263 | miR-199a-5p (and other miRNAs w/seed CCAGUGU) | 2.810 | TargetScan Human | Moderate (predicted) | ESPN | espin | Cytoplasm | other |
| MIMAT0000263 | miR-199a-5p (and other miRNAs w/seed CCAGUGU) | 2.810 | Expert Findings,TargetSc | ntally Observed,Moderate ( | ETS1 | ETS proto-oncogene 1, transcription facto | Nucleus | transcription regulator |
| MIMAT0000263 | miR-199a-5p (and other miRNAs w/seed CCAGUGU) | 2.810 | TargetScan Human | High (predicted) | EVX2 | even-skipped homeobox 2 | Nucleus | transcription regulator |
| MIMAT0000263 | miR-199a-5p (and other miRNAs w/seed CCAGUGU) | 2.810 | TargetScan Human | Moderate (predicted) | EXOC8 | exocyst complex component 8 | Plasma Membrane | other |
| MIMAT0000263 | miR-199a-5p (and other miRNAs w/seed CCAGUGU) | 2.810 | TargetScan Human | High (predicted) | FABP2 | fatty acid binding protein 2 | Cytoplasm | transporter |
| MIMAT0000263 | miR-199a-5p (and other miRNAs w/seed CCAGUGU) | 2.810 | TargetScan Human | Moderate (predicted) | FADS3 | fatty acid desaturase 3 | Plasma Membrane | enzyme |
| MIMAT0000263 | miR-199a-5p (and other miRNAs w/seed CCAGUGU) | 2.810 | TargetScan Human | Moderate (predicted) | FAM107A | mily with sequence similarity 107 member | Nucleus | other |
| MIMAT0000263 | miR-199a-5p (and other miRNAs w/seed CCAGUGU) | 2.810 | TargetScan Human | High (predicted) | FAM107B | mily with sequence similarity 107 member | Other | other |
| MIMAT0000263 | miR-199a-5p (and other miRNAs w/seed CCAGUGU) | 2.810 | TargetScan Human | Moderate (predicted) | FAM161B | FAM161 centrosomal protein B | Cytoplasm | other |
| MIMAT0000263 | miR-199a-5p (and other miRNAs w/seed CCAGUGU) | 2.810 | TargetScan Human | Moderate (predicted) | FAM169A | mily with sequence similarity 169 member | Other | other |
| MIMAT0000263 | miR-199a-5p (and other miRNAs w/seed CCAGUGU) | 2.810 | TargetScan Human | High (predicted) | FAM216B | mily with sequence similarity 216 member | Other | other |
| MIMAT0000263 | miR-199a-5p (and other miRNAs w/seed CCAGUGU) | 2.810 | TargetScan Human | High (predicted) | FAM222B | mily with sequence similarity 222 member | Nucleus | other |
| MIMAT0000263 | miR-199a-5p (and other miRNAs w/seed CCAGUGU) | 2.810 | TargetScan Human | Moderate (predicted) | FAM234A | mily with sequence similarity 234 member | Plasma Membrane | other |
| MIMAT0000263 | miR-199a-5p (and other miRNAs w/seed CCAGUGU) | 2.810 | TargetScan Human | Moderate (predicted) | FAM27C | amily with sequence similarity 27 member | Other | other |
| MIMAT0000263 | miR-199a-5p (and other miRNAs w/seed CCAGUGU) | 2.810 | TargetScan Human | Moderate (predicted) | FARP2 | M, ARH/RhoGEF and pleckstrin domain pr | Cytoplasm | other |
| MIMAT0000263 | miR-199a-5p (and other miRNAs w/seed CCAGUGU) | 2.810 | TargetScan Human | Moderate (predicted) | FBXO17 | F-box protein 17 | Cytoplasm | enzyme |
| MIMAT0000263 | miR-199a-5p (and other miRNAs w/seed CCAGUGU) | 2.810 | TargetScan Human | Moderate (predicted) | FBXO30 | F-box protein 30 | Extracellular Space | enzyme |
| MIMAT0000263 | miR-199a-5p (and other miRNAs w/seed CCAGUGU) | 2.810 | TargetScan Human | Moderate (predicted) | FBXO33 | F-box protein 33 | Other | other |
| MIMAT0000263 | miR-199a-5p (and other miRNAs w/seed CCAGUGU) | 2.810 | TargetScan Human | High (predicted) | FCAMR | Fc fragment of IgA and IgM receptor | Plasma Membrane | transmembrane receptor |
| MIMAT0000263 | miR-199a-5p (and other miRNAs w/seed CCAGUGU) | 2.810 | TargetScan Human | Moderate (predicted) | FCRL6 | Fc receptor like 6 | Plasma Membrane | other |
| MIMAT0000263 | miR-199a-5p (and other miRNAs w/seed CCAGUGU) | 2.810 | TargetScan Human | Moderate (predicted) | FEN1 | flap structure-specific endonuclease 1 | Nucleus | enzyme |
| MIMAT0000263 | miR-199a-5p (and other miRNAs w/seed CCAGUGU) | 2.810 | TargetScan Human | Moderate (predicted) | FER | FER tyrosine kinase | Cytoplasm | kinase |
| MIMAT0000263 | miR-199a-5p (and other miRNAs w/seed CCAGUGU) | 2.810 | TargetScan Human | Moderate (predicted) | FGD3 | YVE, RhoGEF and PH domain containing | Cytoplasm | other |
| MIMAT0000263 | miR-199a-5p (and other miRNAs w/seed CCAGUGU) | 2.810 | TargetScan Human | High (predicted) | FGF11 | fibroblast growth factor 11 | Extracellular Space | growth factor |
| MIMAT0000263 | miR-199a-5p (and other miRNAs w/seed CCAGUGU) | 2.810 | TargetScan Human | High (predicted) | FGFBP2 | fibroblast growth factor binding protein 2 | Extracellular Space | other |
| MIMAT0000263 | miR-199a-5p (and other miRNAs w/seed CCAGUGU) | 2.810 | TargetScan Human | Moderate (predicted) | FIGN | fidgetin, microtubule severing factor | Nucleus | other |
| MIMAT0000263 | miR-199a-5p (and other miRNAs w/seed CCAGUGU) | 2.810 | TargetScan Human | Moderate (predicted) | FKBP5 | FKBP prolyl isomerase 5 | Nucleus | enzyme |
| MIMAT0000263 | miR-199a-5p (and other miRNAs w/seed CCAGUGU) | 2.810 | TargetScan Human | High (predicted) | FLRT3 | onectin leucine rich transmembrane prote | Plasma Membrane | other |
| MIMAT0000263 | miR-199a-5p (and other miRNAs w/seed CCAGUGU) | 2.810 | TargetScan Human | Moderate (predicted) | FNTB | farnesyltransferase, CAAX box, beta | Cytoplasm | enzyme |
| MIMAT0000263 | miR-199a-5p (and other miRNAs w/seed CCAGUGU) | 2.810 | TargetScan Human | Moderate (predicted) | FOXC2 | forkhead box C2 | Nucleus | transcription regulator |
| MIMAT0000263 | miR-199a-5p (and other miRNAs w/seed CCAGUGU) | 2.810 | TargetScan Human | Moderate (predicted) | FOXI1 | forkhead box I1 | Nucleus | transcription regulator |
| MIMAT0000263 | miR-199a-5p (and other miRNAs w/seed CCAGUGU) | 2.810 | TargetScan Human | Moderate (predicted) | FPGS | folylpolyglutamate synthase | Cytoplasm | enzyme |
| MIMAT0000263 | miR-199a-5p (and other miRNAs w/seed CCAGUGU) | 2.810 | TargetScan Human | Moderate (predicted) | FRK | fyn related Src family tyrosine kinase | Nucleus | kinase |
| MIMAT0000263 | miR-199a-5p (and other miRNAs w/seed CCAGUGU) | 2.810 | TargetScan Human | Moderate (predicted) | FSD2 | onectin type III and SPRY domain containi | Other | other |
| MIMAT0000263 | miR-199a-5p (and other miRNAs w/seed CCAGUGU) | 2.810 | TargetScan Human | Moderate (predicted) | FSTL4 | follistatin like 4 | Cytoplasm | other |
| MIMAT0000263 | miR-199a-5p (and other miRNAs w/seed CCAGUGU) | 2.810 | TargetScan Human | Moderate (predicted) | FUT10 | fucosyltransferase 10 | Cytoplasm | enzyme |
| MIMAT0000263 | miR-199a-5p (and other miRNAs w/seed CCAGUGU) | 2.810 | TargetScan Human | Moderate (predicted) | FUT9 | fucosyltransferase 9 | Cytoplasm | enzyme |
| MIMAT0000263 | miR-199a-5p (and other miRNAs w/seed CCAGUGU) | 2.810 | TargetScan Human | Moderate (predicted) | FXR1 | FMR1 autosomal homolog 1 | Cytoplasm | other |
| MIMAT0000263 | miR-199a-5p (and other miRNAs w/seed CCAGUGU) | 2.810 | TargetScan Human | Moderate (predicted) | FXYD4 | YD domain containing ion transport regulat | Plasma Membrane | ion channel |
| MIMAT0000263 | miR-199a-5p (and other miRNAs w/seed CCAGUGU) | 2.810 | TargetScan Human | High (predicted) | FZD4 | frizzled class receptor 4 | Plasma Membrane | G-protein coupled receptor |
| MIMAT0000263 | miR-199a-5p (and other miRNAs w/seed CCAGUGU) | 2.810 | TargetScan Human | High (predicted) | FZD6 | frizzled class receptor 6 | Plasma Membrane | G-protein coupled receptor |
| MIMAT0000263 | miR-199a-5p (and other miRNAs w/seed CCAGUGU) | 2.810 | TargetScan Human | Moderate (predicted) | GABRA4 | -aminobutyric acid type A receptor subuni | Plasma Membrane | ion channel |
| MIMAT0000263 | miR-199a-5p (and other miRNAs w/seed CCAGUGU) | 2.810 | TargetScan Human | High (predicted) | GABRE | -aminobutyric acid type A receptor subunit | Plasma Membrane | ion channel |
| MIMAT0000263 | miR-199a-5p (and other miRNAs w/seed CCAGUGU) | 2.810 | TargetScan Human | High (predicted) | GAS2L2 | growth arrest specific 2 like 2 | Cytoplasm | other |
| MIMAT0000263 | miR-199a-5p (and other miRNAs w/seed CCAGUGU) | 2.810 | TargetScan Human | High (predicted) | GBGT1 | ,3-N-acetylgalactosaminyltransferase 1 (F | Other | enzyme |
| MIMAT0000263 | miR-199a-5p (and other miRNAs w/seed CCAGUGU) | 2.810 | TargetScan Human | High (predicted) | GCNT2 | osaminyl (N-acetyl) transferase 2 (I blood g | Cytoplasm | enzyme |
| MIMAT0000263 | miR-199a-5p (and other miRNAs w/seed CCAGUGU) | 2.810 | TargetScan Human | Moderate (predicted) | GDPD1 | osphodiester phosphodiesterase domain c | Cytoplasm | enzyme |
| MIMAT0000263 | miR-199a-5p (and other miRNAs w/seed CCAGUGU) | 2.810 | TargetScan Human | High (predicted) | GFRAL | GDNF family receptor alpha like | Plasma Membrane | transmembrane receptor |
| MIMAT0000263 | miR-199a-5p (and other miRNAs w/seed CCAGUGU) | 2.810 | TargetScan Human | Moderate (predicted) | GIT1 | GIT ArfGAP 1 | Nucleus | kinase |
| MIMAT0000263 | miR-199a-5p (and other miRNAs w/seed CCAGUGU) | 2.810 | TargetScan Human | High (predicted) | GJA5 | gap junction protein alpha 5 | Plasma Membrane | transporter |
| MIMAT0000263 | miR-199a-5p (and other miRNAs w/seed CCAGUGU) | 2.810 | TargetScan Human | Moderate (predicted) | GJB3 | gap junction protein beta 3 | Plasma Membrane | transporter |
| MIMAT0000263 | miR-199a-5p (and other miRNAs w/seed CCAGUGU) | 2.810 | TargetScan Human | Moderate (predicted) | GMCL1 | erm cell-less 1, spermatogenesis associate | Nucleus | other |
| MIMAT0000263 | miR-199a-5p (and other miRNAs w/seed CCAGUGU) | 2.810 | TargetScan Human | Moderate (predicted) | GMEB1 | ocorticoid modulatory element binding prot | Nucleus | transcription regulator |
| MIMAT0000263 | miR-199a-5p (and other miRNAs w/seed CCAGUGU) | 2.810 | TargetScan Human | Moderate (predicted) | GMFB | glia maturation factor beta | Cytoplasm | growth factor |
| MIMAT0000263 | miR-199a-5p (and other miRNAs w/seed CCAGUGU) | 2.810 | TargetScan Human | High (predicted) | GNG5 | G protein subunit gamma 5 | Plasma Membrane | other |
| MIMAT0000263 | miR-199a-5p (and other miRNAs w/seed CCAGUGU) | 2.810 | TargetScan Human | Moderate (predicted) | GNG7 | G protein subunit gamma 7 | Plasma Membrane | enzyme |
| MIMAT0000263 | miR-199a-5p (and other miRNAs w/seed CCAGUGU) | 2.810 | TargetScan Human | Moderate (predicted) | GOSR1 | golgi SNAP receptor complex member 1 | Cytoplasm | transporter |
| MIMAT0000263 | miR-199a-5p (and other miRNAs w/seed CCAGUGU) | 2.810 | TargetScan Human | Moderate (predicted) | GPD2 | glycerol-3-phosphate dehydrogenase 2 | Cytoplasm | enzyme |
| MIMAT0000263 | miR-199a-5p (and other miRNAs w/seed CCAGUGU) | 2.810 | TargetScan Human | Moderate (predicted) | GPR180 | G protein-coupled receptor 180 | Cytoplasm | other |
| MIMAT0000263 | miR-199a-5p (and other miRNAs w/seed CCAGUGU) | 2.810 | TargetScan Human | Moderate (predicted) | GPR39 | G protein-coupled receptor 39 | Plasma Membrane | G-protein coupled receptor |
| MIMAT0000263 | miR-199a-5p (and other miRNAs w/seed CCAGUGU) | 2.810 | TargetScan Human | High (predicted) | GPR63 | G protein-coupled receptor 63 | Plasma Membrane | G-protein coupled receptor |
| MIMAT0000263 | miR-199a-5p (and other miRNAs w/seed CCAGUGU) | 2.810 | TargetScan Human | High (predicted) | GPR89A/GPR89B | G protein-coupled receptor 89B | Cytoplasm | ion channel |
| MIMAT0000263 | miR-199a-5p (and other miRNAs w/seed CCAGUGU) | 2.810 | TargetScan Human | High (predicted) | GPRC5A | tein-coupled receptor class C group 5 mer | Plasma Membrane | G-protein coupled receptor |
| MIMAT0000263 | miR-199a-5p (and other miRNAs w/seed CCAGUGU) | 2.810 | TargetScan Human | Moderate (predicted) | GRAMD1B | GRAM domain containing 1B | Cytoplasm | transporter |
| MIMAT0000263 | miR-199a-5p (and other miRNAs w/seed CCAGUGU) | 2.810 | TargetScan Human | High (predicted) | GRB10 | growth factor receptor bound protein 10 | Cytoplasm | other |
| MIMAT0000263 | miR-199a-5p (and other miRNAs w/seed CCAGUGU) | 2.810 | TargetScan Human | High (predicted) | GRIP1 | glutamate receptor interacting protein 1 | Plasma Membrane | transcription regulator |
| MIMAT0000263 | miR-199a-5p (and other miRNAs w/seed CCAGUGU) | 2.810 | TargetScan Human | High (predicted) | GRTP1 | growth hormone regulated TBC protein 1 | Other | other |
| MIMAT0000263 | miR-199a-5p (and other miRNAs w/seed CCAGUGU) | 2.810 | TargetScan Human | Moderate (predicted) | GSK3B | glycogen synthase kinase 3 beta | Nucleus | kinase |
| MIMAT0000263 | miR-199a-5p (and other miRNAs w/seed CCAGUGU) | 2.810 | TargetScan Human | Moderate (predicted) | GUCY1A2 | guanylate cyclase 1 soluble subunit alpha | Cytoplasm | enzyme |
| MIMAT0000263 | miR-199a-5p (and other miRNAs w/seed CCAGUGU) | 2.810 | TargetScan Human | Moderate (predicted) | GZMB | granzyme B | Cytoplasm | peptidase |
| MIMAT0000263 | miR-199a-5p (and other miRNAs w/seed CCAGUGU) | 2.810 | TargetScan Human | Moderate (predicted) | H4C15 | H4 clustered histone 15 | Nucleus | other |
| MIMAT0000263 | miR-199a-5p (and other miRNAs w/seed CCAGUGU) | 2.810 | TargetScan Human | High (predicted) | HAPLN1 | hyaluronan and proteoglycan link protein 1 | Extracellular Space | other |
| MIMAT0000263 | miR-199a-5p (and other miRNAs w/seed CCAGUGU) | 2.810 | TargetScan Human | Moderate (predicted) | HAVCR1 | hepatitis A virus cellular receptor 1 | Plasma Membrane | other |
| MIMAT0000263 | miR-199a-5p (and other miRNAs w/seed CCAGUGU) | 2.810 | TargetScan Human | Moderate (predicted) | HCCS | holocytochrome c synthase | Cytoplasm | enzyme |
| MIMAT0000263 | miR-199a-5p (and other miRNAs w/seed CCAGUGU) | 2.810 | TargetScan Human | High (predicted) | HDDC2 | HD domain containing 2 | Cytoplasm | other |
| MIMAT0000263 | miR-199a-5p (and other miRNAs w/seed CCAGUGU) | 2.810 | TargetScan Human | Moderate (predicted) | HECTD2 | HECT domain E3 ubiquitin protein ligase 2 | Cytoplasm | enzyme |
| MIMAT0000263 | miR-199a-5p (and other miRNAs w/seed CCAGUGU) | 2.810 | TargetScan Human | Moderate (predicted) | HECTD3 | HECT domain E3 ubiquitin protein ligase 3 | Cytoplasm | enzyme |
| MIMAT0000263 | miR-199a-5p (and other miRNAs w/seed CCAGUGU) | 2.810 | TargetScan Human | Moderate (predicted) | HGF | hepatocyte growth factor | Extracellular Space | growth factor |
| MIMAT0000263 | miR-199a-5p (and other miRNAs w/seed CCAGUGU) | 2.810 | TargetScan Human | Moderate (predicted) | HHIPL2 | HHIP like 2 | Other | other |
| MIMAT0000263 | miR-199a-5p (and other miRNAs w/seed CCAGUGU) | 2.810 | Findings,TargetScan Hu | ntally Observed,Moderate ( | HIF1A | hypoxia inducible factor 1 subunit alpha | Nucleus | transcription regulator |
| MIMAT0000263 | miR-199a-5p (and other miRNAs w/seed CCAGUGU) | 2.810 | TargetScan Human | High (predicted) | HIPK2 | homeodomain interacting protein kinase 2 | Nucleus | kinase |
| MIMAT0000263 | miR-199a-5p (and other miRNAs w/seed CCAGUGU) | 2.810 | TargetScan Human | Moderate (predicted) | HK2 | hexokinase 2 | Cytoplasm | kinase |
| MIMAT0000263 | miR-199a-5p (and other miRNAs w/seed CCAGUGU) | 2.810 | TargetScan Human | Moderate (predicted) | HLF | F transcription factor, PAR bZIP family mer | Nucleus | transcription regulator |
| MIMAT0000263 | miR-199a-5p (and other miRNAs w/seed CCAGUGU) | 2.810 | TargetScan Human | High (predicted) | HMCN1 | hemicentin 1 | Extracellular Space | other |
| MIMAT0000263 | miR-199a-5p (and other miRNAs w/seed CCAGUGU) | 2.810 | TargetScan Human | Moderate (predicted) | HMGN1 | n mobility group nucleosome binding doma | Nucleus | transcription regulator |
| MIMAT0000263 | miR-199a-5p (and other miRNAs w/seed CCAGUGU) | 2.810 | TargetScan Human | Moderate (predicted) | HOXA7 | homeobox A7 | Nucleus | transcription regulator |
| MIMAT0000263 | miR-199a-5p (and other miRNAs w/seed CCAGUGU) | 2.810 | TargetScan Human | Moderate (predicted) | HRCT1 | histidine rich carboxyl terminus 1 | Other | other |
| MIMAT0000263 | miR-199a-5p (and other miRNAs w/seed CCAGUGU) | 2.810 | TargetScan Human | Moderate (predicted) | HSPA12A | t shock protein family A (Hsp70) member | Cytoplasm | other |
| MIMAT0000263 | miR-199a-5p (and other miRNAs w/seed CCAGUGU) | 2.810 | TargetScan Human | Moderate (predicted) | HSPA4 | eat shock protein family A (Hsp70) membe | Cytoplasm | other |
| MIMAT0000263 | miR-199a-5p (and other miRNAs w/seed CCAGUGU) | 2.810 | TargetScan Human | High (predicted) | HSPA5 | eat shock protein family A (Hsp70) membe | Cytoplasm | enzyme |
| MIMAT0000263 | miR-199a-5p (and other miRNAs w/seed CCAGUGU) | 2.810 | TargetScan Human | Moderate (predicted) | HSPBAP1 | HSPB1 associated protein 1 | Other | other |
| MIMAT0000263 | miR-199a-5p (and other miRNAs w/seed CCAGUGU) | 2.810 | TargetScan Human | Moderate (predicted) | HSPE1-MOB4 | HSPE1-MOB4 readthrough | Other | other |
| MIMAT0000263 | miR-199a-5p (and other miRNAs w/seed CCAGUGU) | 2.810 | TargetScan Human | Moderate (predicted) | IFI35 | interferon induced protein 35 | Nucleus | other |
| MIMAT0000263 | miR-199a-5p (and other miRNAs w/seed CCAGUGU) | 2.810 | TargetScan Human | High (predicted) | IGFBP1 | insulin like growth factor binding protein 1 | Extracellular Space | other |
| MIMAT0000263 | miR-199a-5p (and other miRNAs w/seed CCAGUGU) | 2.810 | TargetScan Human | Moderate (predicted) | IGLL1/IGLL5 | immunoglobulin lambda like polypeptide 1 | Plasma Membrane | other |
| MIMAT0000263 | miR-199a-5p (and other miRNAs w/seed CCAGUGU) | 2.810 | TargetScan Human | Moderate (predicted) | IKBKB | tor of nuclear factor kappa B kinase subun | Cytoplasm | kinase |
| MIMAT0000263 | miR-199a-5p (and other miRNAs w/seed CCAGUGU) | 2.810 | TargetScan Human | Moderate (predicted) | IL13 | interleukin 13 | Extracellular Space | cytokine |
| MIMAT0000263 | miR-199a-5p (and other miRNAs w/seed CCAGUGU) | 2.810 | TargetScan Human | High (predicted) | IL23R | interleukin 23 receptor | Plasma Membrane | transmembrane receptor |
| MIMAT0000263 | miR-199a-5p (and other miRNAs w/seed CCAGUGU) | 2.810 | TargetScan Human | Moderate (predicted) | IL24 | interleukin 24 | Extracellular Space | cytokine |
| MIMAT0000263 | miR-199a-5p (and other miRNAs w/seed CCAGUGU) | 2.810 | TargetScan Human | High (predicted) | IL36B | interleukin 36 beta | Extracellular Space | cytokine |
| MIMAT0000263 | miR-199a-5p (and other miRNAs w/seed CCAGUGU) | 2.810 | TargetScan Human | Moderate (predicted) | INTS2 | integrator complex subunit 2 | Nucleus | other |
| MIMAT0000263 | miR-199a-5p (and other miRNAs w/seed CCAGUGU) | 2.810 | TargetScan Human | Moderate (predicted) | IP6K3 | inositol hexakisphosphate kinase 3 | Cytoplasm | kinase |
| MIMAT0000263 | miR-199a-5p (and other miRNAs w/seed CCAGUGU) | 2.810 | TargetScan Human | High (predicted) | IPO8 | importin 8 | Nucleus | transporter |
| MIMAT0000263 | miR-199a-5p (and other miRNAs w/seed CCAGUGU) | 2.810 | TargetScan Human | Moderate (predicted) | IRS4 | insulin receptor substrate 4 | Plasma Membrane | other |
| MIMAT0000263 | miR-199a-5p (and other miRNAs w/seed CCAGUGU) | 2.810 | TargetScan Human | Moderate (predicted) | ITGA3 | integrin subunit alpha 3 | Plasma Membrane | other |
| MIMAT0000263 | miR-199a-5p (and other miRNAs w/seed CCAGUGU) | 2.810 | TargetScan Human | Moderate (predicted) | ITGA8 | integrin subunit alpha 8 | Plasma Membrane | other |
| MIMAT0000263 | miR-199a-5p (and other miRNAs w/seed CCAGUGU) | 2.810 | TargetScan Human | High (predicted) | JKAMP | NK1/MAPK8 associated membrane protei | Cytoplasm | other |
| MIMAT0000263 | miR-199a-5p (and other miRNAs w/seed CCAGUGU) | 2.810 | TargetScan Human | Moderate (predicted) | JPH3 | junctophilin 3 | Plasma Membrane | other |
| MIMAT0000263 | miR-199a-5p (and other miRNAs w/seed CCAGUGU) | 2.810 | TargetScan Human | Moderate (predicted) | JUNB | proto-oncogene, AP-1 transcription factor s | Nucleus | transcription regulator |
| MIMAT0000263 | miR-199a-5p (and other miRNAs w/seed CCAGUGU) | 2.810 | TargetScan Human | Moderate (predicted) | KANSL1L | KAT8 regulatory NSL complex subunit 1 lik | Other | other |
| MIMAT0000263 | miR-199a-5p (and other miRNAs w/seed CCAGUGU) | 2.810 | TargetScan Human | Moderate (predicted) | KCND2 | um voltage-gated channel subfamily D me | Plasma Membrane | ion channel |
| MIMAT0000263 | miR-199a-5p (and other miRNAs w/seed CCAGUGU) | 2.810 | TargetScan Human | High (predicted) | KCND3 | um voltage-gated channel subfamily D me | Plasma Membrane | ion channel |
| MIMAT0000263 | miR-199a-5p (and other miRNAs w/seed CCAGUGU) | 2.810 | TargetScan Human | Moderate (predicted) | KCNE1 | oltage-gated channel subfamily E regulato | Plasma Membrane | ion channel |
| MIMAT0000263 | miR-199a-5p (and other miRNAs w/seed CCAGUGU) | 2.810 | TargetScan Human | Moderate (predicted) | KCNIP1 | sium voltage-gated channel interacting pro | Plasma Membrane | ion channel |
| MIMAT0000263 | miR-199a-5p (and other miRNAs w/seed CCAGUGU) | 2.810 | TargetScan Human | Moderate (predicted) | KCNK15 | m two pore domain channel subfamily K m | Plasma Membrane | ion channel |
| MIMAT0000263 | miR-199a-5p (and other miRNAs w/seed CCAGUGU) | 2.810 | TargetScan Human | Moderate (predicted) | KCNU1 | m calcium-activated channel subfamily U r | Plasma Membrane | ion channel |
| MIMAT0000263 | miR-199a-5p (and other miRNAs w/seed CCAGUGU) | 2.810 | TargetScan Human | Moderate (predicted) | KCNV1 | voltage-gated channel modifier subfamily V | Plasma Membrane | ion channel |
| MIMAT0000263 | miR-199a-5p (and other miRNAs w/seed CCAGUGU) | 2.810 | TargetScan Human | Moderate (predicted) | KDM1B | lysine demethylase 1B | Nucleus | enzyme |
| MIMAT0000263 | miR-199a-5p (and other miRNAs w/seed CCAGUGU) | 2.810 | TargetScan Human | Moderate (predicted) | KDM3B | lysine demethylase 3B | Nucleus | enzyme |
| MIMAT0000263 | miR-199a-5p (and other miRNAs w/seed CCAGUGU) | 2.810 | TargetScan Human | Moderate (predicted) | KIAA0040 | KIAA0040 | Other | other |
| MIMAT0000263 | miR-199a-5p (and other miRNAs w/seed CCAGUGU) | 2.810 | TargetScan Human | Moderate (predicted) | KIAA0087 | KIAA0087 lncRNA | Other | other |
| MIMAT0000263 | miR-199a-5p (and other miRNAs w/seed CCAGUGU) | 2.810 | TargetScan Human | Moderate (predicted) | KIAA0753 | KIAA0753 | Cytoplasm | other |
| MIMAT0000263 | miR-199a-5p (and other miRNAs w/seed CCAGUGU) | 2.810 | TargetScan Human | Moderate (predicted) | KIAA1109 | KIAA1109 | Other | other |
| MIMAT0000263 | miR-199a-5p (and other miRNAs w/seed CCAGUGU) | 2.810 | TargetScan Human | Moderate (predicted) | KIAA1958 | KIAA1958 | Other | other |

Sup. Table 3. List of 3699 targets of the seven dysregulated miRNAs obtained from three different target prediction databases,TargetScan TarBase and miRecords considering those predicted as moderate, high, or experimentally validated, respectively.

| | | | | | | | | |
|---|---|---|---|---|---|---|---|---|
| MIMAT0000263 | miR-199a-5p (and other miRNAs w/seed CCAGUGU) | 2.810 | TargetScan Human | High (predicted) | KL | klotho | Extracellular Space | enzyme |
| MIMAT0000263 | miR-199a-5p (and other miRNAs w/seed CCAGUGU) | 2.810 | TargetScan Human | Moderate (predicted) | L23/PHOSPHO2-KLI | kelch like family member 23 | Extracellular Space | other |
| MIMAT0000263 | miR-199a-5p (and other miRNAs w/seed CCAGUGU) | 2.810 | TargetScan Human | Moderate (predicted) | KLHL29 | kelch like family member 29 | Other | other |
| MIMAT0000263 | miR-199a-5p (and other miRNAs w/seed CCAGUGU) | 2.810 | TargetScan Human | High (predicted) | KLHL32 | kelch like family member 32 | Other | other |
| MIMAT0000263 | miR-199a-5p (and other miRNAs w/seed CCAGUGU) | 2.810 | TargetScan Human | High (predicted) | KLK7 | kallikrein related peptidase 7 | Extracellular Space | peptidase |
| MIMAT0000263 | miR-199a-5p (and other miRNAs w/seed CCAGUGU) | 2.810 | TargetScan Human | Moderate (predicted) | KLLN | illin, p53 regulated DNA replication inhibit | Nucleus | other |
| MIMAT0000263 | miR-199a-5p (and other miRNAs w/seed CCAGUGU) | 2.810 | TargetScan Human | Moderate (predicted) | KLRF1 | killer cell lectin like receptor F1 | Plasma Membrane | transmembrane receptor |
| MIMAT0000263 | miR-199a-5p (and other miRNAs w/seed CCAGUGU) | 2.810 | TargetScan Human | Moderate (predicted) | KPNA4 | karyopherin subunit alpha 4 | Nucleus | other |
| MIMAT0000263 | miR-199a-5p (and other miRNAs w/seed CCAGUGU) | 2.810 | TargetScan Human | Moderate (predicted) | KRAS | KRAS proto-oncogene, GTPase | Cytoplasm | enzyme |
| MIMAT0000263 | miR-199a-5p (and other miRNAs w/seed CCAGUGU) | 2.810 | TargetScan Human | Moderate (predicted) | KRTAP4-5 | keratin associated protein 4-5 | Cytoplasm | other |
| MIMAT0000263 | miR-199a-5p (and other miRNAs w/seed CCAGUGU) | 2.810 | TargetScan Human | Moderate (predicted) | KYAT3 | kynurenine aminotransferase 3 | Cytoplasm | enzyme |
| MIMAT0000263 | miR-199a-5p (and other miRNAs w/seed CCAGUGU) | 2.810 | TargetScan Human | Moderate (predicted) | LAMC1 | laminin subunit gamma 1 | Extracellular Space | other |
| MIMAT0000263 | miR-199a-5p (and other miRNAs w/seed CCAGUGU) | 2.810 | TarBase,miRecords | Experimentally Observed | LAMC2 | laminin subunit gamma 2 | Extracellular Space | other |
| MIMAT0000263 | miR-199a-5p (and other miRNAs w/seed CCAGUGU) | 2.810 | TargetScan Human | Moderate (predicted) | LARGE1 | LARGE xylosyl- and glucuronyltransferase | Cytoplasm | enzyme |
| MIMAT0000263 | miR-199a-5p (and other miRNAs w/seed CCAGUGU) | 2.810 | TargetScan Human | Moderate (predicted) | LARP1 | a ribonucleoprotein 1, translational regulat | Cytoplasm | translation regulator |
| MIMAT0000263 | miR-199a-5p (and other miRNAs w/seed CCAGUGU) | 2.810 | TargetScan Human | Moderate (predicted) | LARP4 | La ribonucleoprotein 4 | Cytoplasm | other |
| MIMAT0000263 | miR-199a-5p (and other miRNAs w/seed CCAGUGU) | 2.810 | TargetScan Human | Moderate (predicted) | LCLAT1 | lysocardiolipin acyltransferase 1 | Cytoplasm | enzyme |
| MIMAT0000263 | miR-199a-5p (and other miRNAs w/seed CCAGUGU) | 2.810 | TargetScan Human | High (predicted) | LCOR | and dependent nuclear receptor corepress | Nucleus | transcription regulator |
| MIMAT0000263 | miR-199a-5p (and other miRNAs w/seed CCAGUGU) | 2.810 | TargetScan Human | Moderate (predicted) | LDLRAD3 | ity lipoprotein receptor class A domain cor | Plasma Membrane | other |
| MIMAT0000263 | miR-199a-5p (and other miRNAs w/seed CCAGUGU) | 2.810 | TargetScan Human | Moderate (predicted) | LEFTY1 | left-right determination factor 1 | Extracellular Space | growth factor |
| MIMAT0000263 | miR-199a-5p (and other miRNAs w/seed CCAGUGU) | 2.810 | TargetScan Human | Moderate (predicted) | LHFPL2 | LHFPL tetraspan subfamily member 2 | Other | enzyme |
| MIMAT0000263 | miR-199a-5p (and other miRNAs w/seed CCAGUGU) | 2.810 | TargetScan Human | Moderate (predicted) | LIMD1 | LIM domain containing 1 | Nucleus | transcription regulator |
| MIMAT0000263 | miR-199a-5p (and other miRNAs w/seed CCAGUGU) | 2.810 | TargetScan Human | High (predicted) | LIMS3/LIMS4 | LIM zinc finger domain containing 4 | Other | other |
| MIMAT0000263 | miR-199a-5p (and other miRNAs w/seed CCAGUGU) | 2.810 | TargetScan Human | High (predicted) | LIN7C | molog C, crumbs cell polarity complex cor | Cytoplasm | other |
| MIMAT0000263 | miR-199a-5p (and other miRNAs w/seed CCAGUGU) | 2.810 | TargetScan Human | Moderate (predicted) | LINC01553 | ng intergenic non-protein coding RNA 155 | Other | other |
| MIMAT0000263 | miR-199a-5p (and other miRNAs w/seed CCAGUGU) | 2.810 | TargetScan Human | Moderate (predicted) | LINC01555 | ng intergenic non-protein coding RNA 155 | Other | other |
| MIMAT0000263 | miR-199a-5p (and other miRNAs w/seed CCAGUGU) | 2.810 | TargetScan Human | High (predicted) | LINC01619 | ng intergenic non-protein coding RNA 161 | Other | other |
| MIMAT0000263 | miR-199a-5p (and other miRNAs w/seed CCAGUGU) | 2.810 | TargetScan Human | Moderate (predicted) | LINC02089 | ng intergenic non-protein coding RNA 208 | Other | other |
| MIMAT0000263 | miR-199a-5p (and other miRNAs w/seed CCAGUGU) | 2.810 | TargetScan Human | Moderate (predicted) | LINC02872 | ng intergenic non-protein coding RNA 287 | Other | other |
| MIMAT0000263 | miR-199a-5p (and other miRNAs w/seed CCAGUGU) | 2.810 | TargetScan Human | Moderate (predicted) | LINC02873 | ng intergenic non-protein coding RNA 287 | Other | other |
| MIMAT0000263 | miR-199a-5p (and other miRNAs w/seed CCAGUGU) | 2.810 | TargetScan Human | Moderate (predicted) | LINC02906 | ng intergenic non-protein coding RNA 290 | Other | other |
| MIMAT0000263 | miR-199a-5p (and other miRNAs w/seed CCAGUGU) | 2.810 | TargetScan Human | Moderate (predicted) | LIPG | lipase G, endothelial type | Extracellular Space | enzyme |
| MIMAT0000263 | miR-199a-5p (and other miRNAs w/seed CCAGUGU) | 2.810 | TargetScan Human | Moderate (predicted) | LIX1 | limb and CNS expressed 1 | Other | other |
| MIMAT0000263 | miR-199a-5p (and other miRNAs w/seed CCAGUGU) | 2.810 | TargetScan Human | Moderate (predicted) | LMAN2L | lectin, mannose binding 2 like | Cytoplasm | other |
| MIMAT0000263 | miR-199a-5p (and other miRNAs w/seed CCAGUGU) | 2.810 | TargetScan Human | Moderate (predicted) | LOC100132813 | uncharacterized LOC100132813 | Other | other |
| MIMAT0000263 | miR-199a-5p (and other miRNAs w/seed CCAGUGU) | 2.810 | TargetScan Human | Moderate (predicted) | LOC403312 | utative uncharacterized protein MGC3954 | Other | other |
| MIMAT0000263 | miR-199a-5p (and other miRNAs w/seed CCAGUGU) | 2.810 | TargetScan Human | Moderate (predicted) | LPXN | leupaxin | Cytoplasm | transcription regulator |
| MIMAT0000263 | miR-199a-5p (and other miRNAs w/seed CCAGUGU) | 2.810 | TargetScan Human | Moderate (predicted) | LRRC17 | leucine rich repeat containing 17 | Extracellular Space | other |
| MIMAT0000263 | miR-199a-5p (and other miRNAs w/seed CCAGUGU) | 2.810 | TargetScan Human | Moderate (predicted) | LRRC61 | leucine rich repeat containing 61 | Other | other |
| MIMAT0000263 | miR-199a-5p (and other miRNAs w/seed CCAGUGU) | 2.810 | TargetScan Human | Moderate (predicted) | LRRFIP1 | LRR binding FLII interacting protein 1 | Cytoplasm | transcription regulator |
| MIMAT0000263 | miR-199a-5p (and other miRNAs w/seed CCAGUGU) | 2.810 | TargetScan Human | Moderate (predicted) | LSM8 | M8 homolog, U6 small nuclear RNA associ | Nucleus | other |
| MIMAT0000263 | miR-199a-5p (and other miRNAs w/seed CCAGUGU) | 2.810 | TargetScan Human | Moderate (predicted) | LTB4R2 | leukotriene B4 receptor 2 | Plasma Membrane | G-protein coupled receptor |
| MIMAT0000263 | miR-199a-5p (and other miRNAs w/seed CCAGUGU) | 2.810 | TargetScan Human | Moderate (predicted) | LYRM2 | LYR motif containing 2 | Cytoplasm | other |
| MIMAT0000263 | miR-199a-5p (and other miRNAs w/seed CCAGUGU) | 2.810 | TargetScan Human | Moderate (predicted) | LYRM9 | LYR motif containing 9 | Other | other |
| MIMAT0000263 | miR-199a-5p (and other miRNAs w/seed CCAGUGU) | 2.810 | TargetScan Human | High (predicted) | LYSMD3 | LysM domain containing 3 | Other | other |
| MIMAT0000263 | miR-199a-5p (and other miRNAs w/seed CCAGUGU) | 2.810 | TargetScan Human | High (predicted) | MAB21L1 | mab-21 like 1 | Nucleus | other |
| MIMAT0000263 | miR-199a-5p (and other miRNAs w/seed CCAGUGU) | 2.810 | TargetScan Human | High (predicted) | MACC1 | MET transcriptional regulator MACC1 | Nucleus | transcription regulator |
| MIMAT0000263 | miR-199a-5p (and other miRNAs w/seed CCAGUGU) | 2.810 | TargetScan Human | High (predicted) | MAGT1 | magnesium transporter 1 | Plasma Membrane | enzyme |
| MIMAT0000263 | miR-199a-5p (and other miRNAs w/seed CCAGUGU) | 2.810 | TargetScan Human | Moderate (predicted) | MAP2K4 | mitogen-activated protein kinase kinase 4 | Cytoplasm | kinase |
| MIMAT0000263 | miR-199a-5p (and other miRNAs w/seed CCAGUGU) | 2.810 | TargetScan Human | High (predicted) | MAP3K11 | gen-activated protein kinase kinase kinas | Cytoplasm | kinase |
| MIMAT0000263 | miR-199a-5p (and other miRNAs w/seed CCAGUGU) | 2.810 | TargetScan Human | Moderate (predicted) | MAP4K3 | n-activated protein kinase kinase kinase k | Plasma Membrane | kinase |
| MIMAT0000263 | miR-199a-5p (and other miRNAs w/seed CCAGUGU) | 2.810 | TargetScan Human | Moderate (predicted) | MAP7D2 | MAP7 domain containing 2 | Other | other |
| MIMAT0000263 | miR-199a-5p (and other miRNAs w/seed CCAGUGU) | 2.810 | TargetScan Human | Moderate (predicted) | MAPRE2 | ubule associated protein RP/EB family me | Cytoplasm | other |
| MIMAT0000263 | miR-199a-5p (and other miRNAs w/seed CCAGUGU) | 2.810 | TargetScan Human | Moderate (predicted) | MARCHF7 | nembrane associated ring-CH-type finger | Extracellular Space | other |
| MIMAT0000263 | miR-199a-5p (and other miRNAs w/seed CCAGUGU) | 2.810 | TargetScan Human | High (predicted) | MARCHF8 | nembrane associated ring-CH-type finger | Cytoplasm | enzyme |
| MIMAT0000263 | miR-199a-5p (and other miRNAs w/seed CCAGUGU) | 2.810 | TargetScan Human | Moderate (predicted) | MARK4 | microtubule affinity regulating kinase 4 | Cytoplasm | kinase |
| MIMAT0000263 | miR-199a-5p (and other miRNAs w/seed CCAGUGU) | 2.810 | TargetScan Human | Moderate (predicted) | MATN2 | matrilin 2 | Extracellular Space | other |
| MIMAT0000263 | miR-199a-5p (and other miRNAs w/seed CCAGUGU) | 2.810 | TargetScan Human | High (predicted) | MBP | myelin basic protein | Extracellular Space | other |
| MIMAT0000263 | miR-199a-5p (and other miRNAs w/seed CCAGUGU) | 2.810 | TargetScan Human | Moderate (predicted) | MCFD2 | tion factor deficiency 2, ER cargo receptor | Cytoplasm | other |
| MIMAT0000263 | miR-199a-5p (and other miRNAs w/seed CCAGUGU) | 2.810 | TargetScan Human | High (predicted) | MCIDAS | rentiation and DNA synthesis associated c | Nucleus | other |
| MIMAT0000263 | miR-199a-5p (and other miRNAs w/seed CCAGUGU) | 2.810 | TargetScan Human | High (predicted) | MCTP1 | ble C2 and transmembrane domain contai | Cytoplasm | other |
| MIMAT0000263 | miR-199a-5p (and other miRNAs w/seed CCAGUGU) | 2.810 | TargetScan Human | Moderate (predicted) | MED4 | mediator complex subunit 4 | Nucleus | transcription regulator |
| MIMAT0000263 | miR-199a-5p (and other miRNAs w/seed CCAGUGU) | 2.810 | TargetScan Human | Moderate (predicted) | MED9 | mediator complex subunit 9 | Nucleus | other |
| MIMAT0000263 | miR-199a-5p (and other miRNAs w/seed CCAGUGU) | 2.810 | TargetScan Human | Moderate (predicted) | MEIS2 | Meis homeobox 2 | Nucleus | transcription regulator |
| MIMAT0000263 | miR-199a-5p (and other miRNAs w/seed CCAGUGU) | 2.810 | TargetScan Human | High (predicted) | METTL21A | methyltransferase like 21A | Nucleus | enzyme |
| MIMAT0000263 | miR-199a-5p (and other miRNAs w/seed CCAGUGU) | 2.810 | TargetScan Human | Moderate (predicted) | MEX3C | mex-3 RNA binding family member C | Cytoplasm | enzyme |
| MIMAT0000263 | miR-199a-5p (and other miRNAs w/seed CCAGUGU) | 2.810 | TargetScan Human | Moderate (predicted) | MFSD6 | jor facilitator superfamily domain containir | Plasma Membrane | transmembrane receptor |
| MIMAT0000263 | miR-199a-5p (and other miRNAs w/seed CCAGUGU) | 2.810 | TargetScan Human | High (predicted) | MGAT4B | osyl-glycoprotein 4-beta-N-acetylglucosam | Cytoplasm | enzyme |
| MIMAT0000263 | miR-199a-5p (and other miRNAs w/seed CCAGUGU) | 2.810 | TargetScan Human | Moderate (predicted) | MGLL | monoglyceride lipase | Plasma Membrane | enzyme |
| MIMAT0000263 | miR-199a-5p (and other miRNAs w/seed CCAGUGU) | 2.810 | TargetScan Human | High (predicted) | MICAL3 | ated monooxygenase, calponin and LIM d | Cytoplasm | enzyme |
| MIMAT0000263 | miR-199a-5p (and other miRNAs w/seed CCAGUGU) | 2.810 | TargetScan Human | Moderate (predicted) | MIER3 | MIER family member 3 | Nucleus | transcription regulator |
| MIMAT0000263 | miR-199a-5p (and other miRNAs w/seed CCAGUGU) | 2.810 | TargetScan Human | High (predicted) | MINDY3 | MINDY lysine 48 deubiquitinase 3 | Nucleus | enzyme |
| MIMAT0000263 | miR-199a-5p (and other miRNAs w/seed CCAGUGU) | 2.810 | TargetScan Human | Moderate (predicted) | MINK1 | misshapen like kinase 1 | Cytoplasm | kinase |
| MIMAT0000263 | miR-199a-5p (and other miRNAs w/seed CCAGUGU) | 2.810 | TargetScan Human | Moderate (predicted) | MINPP1 | ultiple inositol-polyphosphate phosphatase | Cytoplasm | phosphatase |
| MIMAT0000263 | miR-199a-5p (and other miRNAs w/seed CCAGUGU) | 2.810 | TargetScan Human | High (predicted) | MLNR | motilin receptor | Plasma Membrane | G-protein coupled receptor |
| MIMAT0000263 | miR-199a-5p (and other miRNAs w/seed CCAGUGU) | 2.810 | TargetScan Human | Moderate (predicted) | MN1 | N1 proto-oncogene, transcriptional regula | Nucleus | other |
| MIMAT0000263 | miR-199a-5p (and other miRNAs w/seed CCAGUGU) | 2.810 | TargetScan Human | Moderate (predicted) | MOB1B | MOB kinase activator 1B | Cytoplasm | other |
| MIMAT0000263 | miR-199a-5p (and other miRNAs w/seed CCAGUGU) | 2.810 | TargetScan Human | Moderate (predicted) | MOB4 | MOB family member 4, phocein | Cytoplasm | other |
| MIMAT0000263 | miR-199a-5p (and other miRNAs w/seed CCAGUGU) | 2.810 | TargetScan Human | Moderate (predicted) | MOSMO | modulator of smoothened | Other | other |
| MIMAT0000263 | miR-199a-5p (and other miRNAs w/seed CCAGUGU) | 2.810 | TargetScan Human | Moderate (predicted) | MRAP2 | nelanocortin 2 receptor accessory protein | Cytoplasm | other |
| MIMAT0000263 | miR-199a-5p (and other miRNAs w/seed CCAGUGU) | 2.810 | TargetScan Human | Moderate (predicted) | MRPL14 | mitochondrial ribosomal protein L14 | Cytoplasm | other |
| MIMAT0000263 | miR-199a-5p (and other miRNAs w/seed CCAGUGU) | 2.810 | TargetScan Human | High (predicted) | MRPL30 | mitochondrial ribosomal protein L30 | Cytoplasm | other |
| MIMAT0000263 | miR-199a-5p (and other miRNAs w/seed CCAGUGU) | 2.810 | TargetScan Human | Moderate (predicted) | MRPS35 | mitochondrial ribosomal protein S35 | Cytoplasm | other |
| MIMAT0000263 | miR-199a-5p (and other miRNAs w/seed CCAGUGU) | 2.810 | TargetScan Human | Moderate (predicted) | MS4A6A | membrane spanning 4-domains A6A | Cytoplasm | other |
| MIMAT0000263 | miR-199a-5p (and other miRNAs w/seed CCAGUGU) | 2.810 | TargetScan Human | Moderate (predicted) | MTMR2 | myotubularin related protein 2 | Cytoplasm | phosphatase |
| MIMAT0000263 | miR-199a-5p (and other miRNAs w/seed CCAGUGU) | 2.810 | TargetScan Human | Moderate (predicted) | MUC21 | mucin 21, cell surface associated | Cytoplasm | other |
| MIMAT0000263 | miR-199a-5p (and other miRNAs w/seed CCAGUGU) | 2.810 | TargetScan Human | Moderate (predicted) | MYCBP | MYC binding protein | Nucleus | transcription regulator |
| MIMAT0000263 | miR-199a-5p (and other miRNAs w/seed CCAGUGU) | 2.810 | TargetScan Human | High (predicted) | MYEF2 | myelin expression factor 2 | Nucleus | transcription regulator |
| MIMAT0000263 | miR-199a-5p (and other miRNAs w/seed CCAGUGU) | 2.810 | ngenuity Expert Finding | Experimentally Observed | MYH9 | myosin heavy chain 9 | Cytoplasm | enzyme |
| MIMAT0000263 | miR-199a-5p (and other miRNAs w/seed CCAGUGU) | 2.810 | TargetScan Human | High (predicted) | MYRF | myelin regulatory factor | Nucleus | transcription regulator |
| MIMAT0000263 | miR-199a-5p (and other miRNAs w/seed CCAGUGU) | 2.810 | TargetScan Human | High (predicted) | NAA40 | pha-acetyltransferase 40, NatD catalytic su | Cytoplasm | enzyme |
| MIMAT0000263 | miR-199a-5p (and other miRNAs w/seed CCAGUGU) | 2.810 | TargetScan Human | Moderate (predicted) | NAALADL2 | cetylated alpha-linked acidic dipeptidase li | Nucleus | other |
| MIMAT0000263 | miR-199a-5p (and other miRNAs w/seed CCAGUGU) | 2.810 | TargetScan Human | Moderate (predicted) | NAB2 | NGFI-A binding protein 2 | Nucleus | transcription regulator |
| MIMAT0000263 | miR-199a-5p (and other miRNAs w/seed CCAGUGU) | 2.810 | TargetScan Human | Moderate (predicted) | NAGK | N-acetylglucosamine kinase | Cytoplasm | kinase |
| MIMAT0000263 | miR-199a-5p (and other miRNAs w/seed CCAGUGU) | 2.810 | TargetScan Human | Moderate (predicted) | BPF10 (includes othe | NBPF member 19 | Other | other |
| MIMAT0000263 | miR-199a-5p (and other miRNAs w/seed CCAGUGU) | 2.810 | TargetScan Human | Moderate (predicted) | NCEH1 | neutral cholesterol ester hydrolase 1 | Plasma Membrane | enzyme |
| MIMAT0000263 | miR-199a-5p (and other miRNAs w/seed CCAGUGU) | 2.810 | TargetScan Human | Moderate (predicted) | NCSTN | nicastrin | Plasma Membrane | peptidase |
| MIMAT0000263 | miR-199a-5p (and other miRNAs w/seed CCAGUGU) | 2.810 | TargetScan Human | Moderate (predicted) | NDUFS5 | ADH:ubiquinone oxidoreductase subunit S | Cytoplasm | enzyme |
| MIMAT0000263 | miR-199a-5p (and other miRNAs w/seed CCAGUGU) | 2.810 | TargetScan Human | Moderate (predicted) | NECTIN1 | nectin cell adhesion molecule 1 | Plasma Membrane | other |
| MIMAT0000263 | miR-199a-5p (and other miRNAs w/seed CCAGUGU) | 2.810 | TargetScan Human | High (predicted) | NECTIN2 | nectin cell adhesion molecule 2 | Plasma Membrane | transmembrane receptor |
| MIMAT0000263 | miR-199a-5p (and other miRNAs w/seed CCAGUGU) | 2.810 | TargetScan Human | High (predicted) | NEU3 | neuraminidase 3 | Plasma Membrane | enzyme |
| MIMAT0000263 | miR-199a-5p (and other miRNAs w/seed CCAGUGU) | 2.810 | TargetScan Human | Moderate (predicted) | NFATC2IP | ar factor of activated T cells 2 interacting p | Nucleus | other |
| MIMAT0000263 | miR-199a-5p (and other miRNAs w/seed CCAGUGU) | 2.810 | TargetScan Human | Moderate (predicted) | NFIL3 | nuclear factor, interleukin 3 regulated | Nucleus | transcription regulator |
| MIMAT0000263 | miR-199a-5p (and other miRNAs w/seed CCAGUGU) | 2.810 | TargetScan Human | Moderate (predicted) | NFKB1 | nuclear factor kappa B subunit 1 | Nucleus | transcription regulator |
| MIMAT0000263 | miR-199a-5p (and other miRNAs w/seed CCAGUGU) | 2.810 | TargetScan Human | Moderate (predicted) | NGB | neuroglobin | Cytoplasm | transporter |
| MIMAT0000263 | miR-199a-5p (and other miRNAs w/seed CCAGUGU) | 2.810 | TargetScan Human | Moderate (predicted) | NHLH2 | nescient helix-loop-helix 2 | Nucleus | transcription regulator |
| MIMAT0000263 | miR-199a-5p (and other miRNAs w/seed CCAGUGU) | 2.810 | TargetScan Human | High (predicted) | NINL | ninein like | Cytoplasm | other |
| MIMAT0000263 | miR-199a-5p (and other miRNAs w/seed CCAGUGU) | 2.810 | TargetScan Human | High (predicted) | NKAIN3 | m/potassium transporting ATPase interac | Other | other |
| MIMAT0000263 | miR-199a-5p (and other miRNAs w/seed CCAGUGU) | 2.810 | TargetScan Human | Moderate (predicted) | NLK | nemo like kinase | Nucleus | kinase |
| MIMAT0000263 | miR-199a-5p (and other miRNAs w/seed CCAGUGU) | 2.810 | TargetScan Human | Moderate (predicted) | NLRC3 | NLR family CARD domain containing 3 | Cytoplasm | other |
| MIMAT0000263 | miR-199a-5p (and other miRNAs w/seed CCAGUGU) | 2.810 | TargetScan Human | Moderate (predicted) | NLRP7 | NLR family pyrin domain containing 7 | Extracellular Space | other |
| MIMAT0000263 | miR-199a-5p (and other miRNAs w/seed CCAGUGU) | 2.810 | TargetScan Human | Moderate (predicted) | NMNAT3 | cotinamide nucleotide adenylyltransferase | Cytoplasm | enzyme |
| MIMAT0000263 | miR-199a-5p (and other miRNAs w/seed CCAGUGU) | 2.810 | TargetScan Human | Moderate (predicted) | NPAS2 | neuronal PAS domain protein 2 | Nucleus | transcription regulator |
| MIMAT0000263 | miR-199a-5p (and other miRNAs w/seed CCAGUGU) | 2.810 | TargetScan Human | High (predicted) | NPY2R | neuropeptide Y receptor Y2 | Plasma Membrane | G-protein coupled receptor |
| MIMAT0000263 | miR-199a-5p (and other miRNAs w/seed CCAGUGU) | 2.810 | TargetScan Human | High (predicted) | NR1I3 | clear receptor subfamily 1 group I membe | Nucleus | ligand-dependent nuclear receptor |
| MIMAT0000263 | miR-199a-5p (and other miRNAs w/seed CCAGUGU) | 2.810 | TargetScan Human | Moderate (predicted) | NREP | neuronal regeneration related protein | Cytoplasm | other |
| MIMAT0000263 | miR-199a-5p (and other miRNAs w/seed CCAGUGU) | 2.810 | TargetScan Human | Moderate (predicted) | NRSN1 | neurensin 1 | Plasma Membrane | other |
| MIMAT0000263 | miR-199a-5p (and other miRNAs w/seed CCAGUGU) | 2.810 | TargetScan Human | High (predicted) | NSG1 | neuronal vesicle trafficking associated 1 | Cytoplasm | other |
| MIMAT0000263 | miR-199a-5p (and other miRNAs w/seed CCAGUGU) | 2.810 | TargetScan Human | Moderate (predicted) | NSMF | r synaptonuclear signaling and neuronal r | Extracellular Space | other |
| MIMAT0000263 | miR-199a-5p (and other miRNAs w/seed CCAGUGU) | 2.810 | TargetScan Human | High (predicted) | NT5C1A | 5'-nucleotidase, cytosolic IA | Cytoplasm | phosphatase |
| MIMAT0000263 | miR-199a-5p (and other miRNAs w/seed CCAGUGU) | 2.810 | TargetScan Human | Moderate (predicted) | NT5C3A | 5'-nucleotidase, cytosolic IIIA | Cytoplasm | phosphatase |
| MIMAT0000263 | miR-199a-5p (and other miRNAs w/seed CCAGUGU) | 2.810 | TargetScan Human | Moderate (predicted) | NT5DC1 | 5'-nucleotidase domain containing 1 | Other | other |
| MIMAT0000263 | miR-199a-5p (and other miRNAs w/seed CCAGUGU) | 2.810 | TargetScan Human | High (predicted) | NTNG1 | netrin G1 | Extracellular Space | other |
| MIMAT0000263 | miR-199a-5p (and other miRNAs w/seed CCAGUGU) | 2.810 | TargetScan Human | High (predicted) | NUDT5 | nudix hydrolase 5 | Cytoplasm | phosphatase |
| MIMAT0000263 | miR-199a-5p (and other miRNAs w/seed CCAGUGU) | 2.810 | TargetScan Human | Moderate (predicted) | NUP210 | nucleoporin 210 | Nucleus | transporter |
| MIMAT0000263 | miR-199a-5p (and other miRNAs w/seed CCAGUGU) | 2.810 | TargetScan Human | Moderate (predicted) | NXT2 | nuclear transport factor 2 like export factor | Nucleus | transporter |
| MIMAT0000263 | miR-199a-5p (and other miRNAs w/seed CCAGUGU) | 2.810 | TargetScan Human | Moderate (predicted) | OMD | osteomodulin | Extracellular Space | other |
| MIMAT0000263 | miR-199a-5p (and other miRNAs w/seed CCAGUGU) | 2.810 | TargetScan Human | Moderate (predicted) | OPA3 | chondrial membrane lipid metabolism regu | Cytoplasm | other |
| MIMAT0000263 | miR-199a-5p (and other miRNAs w/seed CCAGUGU) | 2.810 | TargetScan Human | Moderate (predicted) | OR1K1 | ctory receptor family 1 subfamily K memb | Plasma Membrane | G-protein coupled receptor |
| MIMAT0000263 | miR-199a-5p (and other miRNAs w/seed CCAGUGU) | 2.810 | TargetScan Human | Moderate (predicted) | OSGIN2 | e stress induced growth inhibitor family m | Other | other |
| MIMAT0000263 | miR-199a-5p (and other miRNAs w/seed CCAGUGU) | 2.810 | TargetScan Human | Moderate (predicted) | OSR1 | odd-skipped related transcription factor 1 | Nucleus | transcription regulator |
| MIMAT0000263 | miR-199a-5p (and other miRNAs w/seed CCAGUGU) | 2.810 | TargetScan Human | High (predicted) | OTULINL | deubiquitinase with linear linkage specifici | Other | other |
| MIMAT0000263 | miR-199a-5p (and other miRNAs w/seed CCAGUGU) | 2.810 | TargetScan Human | Moderate (predicted) | OTX1 | orthodenticle homeobox 1 | Nucleus | transcription regulator |
| MIMAT0000263 | miR-199a-5p (and other miRNAs w/seed CCAGUGU) | 2.810 | TargetScan Human | High (predicted) | P3H2 | prolyl 3-hydroxylase 2 | Cytoplasm | enzyme |
| MIMAT0000263 | miR-199a-5p (and other miRNAs w/seed CCAGUGU) | 2.810 | TargetScan Human | Moderate (predicted) | PAFAH1B1 | tivating factor acetylhydrolase 1b regulator | Cytoplasm | enzyme |
| MIMAT0000263 | miR-199a-5p (and other miRNAs w/seed CCAGUGU) | 2.810 | TargetScan Human | Moderate (predicted) | PAG1 | membrane anchor with glycosphingolipid | Plasma Membrane | other |
| MIMAT0000263 | miR-199a-5p (and other miRNAs w/seed CCAGUGU) | 2.810 | TargetScan Human | Moderate (predicted) | PAN3 | poly(A) specific ribonuclease subunit PAN | Cytoplasm | enzyme |
| MIMAT0000263 | miR-199a-5p (and other miRNAs w/seed CCAGUGU) | 2.810 | TargetScan Human | Moderate (predicted) | PAQR9 | ogestin and adipoQ receptor family membe | Plasma Membrane | other |
| MIMAT0000263 | miR-199a-5p (and other miRNAs w/seed CCAGUGU) | 2.810 | TargetScan Human | Moderate (predicted) | PARP12 | y(ADP-ribose) polymerase family member | Nucleus | enzyme |
| MIMAT0000263 | miR-199a-5p (and other miRNAs w/seed CCAGUGU) | 2.810 | TargetScan Human | Moderate (predicted) | PAX3 | paired box 3 | Nucleus | transcription regulator |
| MIMAT0000263 | miR-199a-5p (and other miRNAs w/seed CCAGUGU) | 2.810 | TargetScan Human | Moderate (predicted) | PAXBP1 | PAX3 and PAX7 binding protein 1 | Nucleus | other |
| MIMAT0000263 | miR-199a-5p (and other miRNAs w/seed CCAGUGU) | 2.810 | TargetScan Human | Moderate (predicted) | PCBD2 | pterin-4 alpha-carbinolamine dehydratase | Nucleus | enzyme |
| MIMAT0000263 | miR-199a-5p (and other miRNAs w/seed CCAGUGU) | 2.810 | TargetScan Human | Moderate (predicted) | PCNX4 | pecanex 4 | Other | other |
| MIMAT0000263 | miR-199a-5p (and other miRNAs w/seed CCAGUGU) | 2.810 | TargetScan Human | Moderate (predicted) | PDE4D | phosphodiesterase 4D | Cytoplasm | enzyme |
| MIMAT0000263 | miR-199a-5p (and other miRNAs w/seed CCAGUGU) | 2.810 | TargetScan Human | Moderate (predicted) | PDE5A | phosphodiesterase 5A | Cytoplasm | enzyme |
| MIMAT0000263 | miR-199a-5p (and other miRNAs w/seed CCAGUGU) | 2.810 | TargetScan Human | Moderate (predicted) | PDIK1L | PDLIM1 interacting kinase 1 like | Nucleus | kinase |

Sup. Table 3. List of 3699 targets of the seven dysregulated miRNAs obtained from three different target prediction databases,TargetScan TarBase and miRecords considering those predicted as moderate, high, or experimentally validated, respectively.

| | | | | | | | | |
|---|---|---|---|---|---|---|---|---|
| MIMAT0000263 | miR-199a-5p (and other miRNAs w/seed CCAGUGU) | 2.810 | TargetScan Human | High (predicted) | PDPN | podoplanin | Plasma Membrane | other |
| MIMAT0000263 | miR-199a-5p (and other miRNAs w/seed CCAGUGU) | 2.810 | TargetScan Human | Moderate (predicted) | PEX2 | peroxisomal biogenesis factor 2 | Cytoplasm | other |
| MIMAT0000263 | miR-199a-5p (and other miRNAs w/seed CCAGUGU) | 2.810 | TargetScan Human | Moderate (predicted) | PEX3 | peroxisomal biogenesis factor 3 | Cytoplasm | other |
| MIMAT0000263 | miR-199a-5p (and other miRNAs w/seed CCAGUGU) | 2.810 | TargetScan Human | Moderate (predicted) | PFN4 | profilin family member 4 | Other | other |
| MIMAT0000263 | miR-199a-5p (and other miRNAs w/seed CCAGUGU) | 2.810 | TargetScan Human | Moderate (predicted) | PGD | phosphogluconate dehydrogenase | Cytoplasm | enzyme |
| MIMAT0000263 | miR-199a-5p (and other miRNAs w/seed CCAGUGU) | 2.810 | TargetScan Human | Moderate (predicted) | PHACTR4 | phosphatase and actin regulator 4 | Plasma Membrane | other |
| MIMAT0000263 | miR-199a-5p (and other miRNAs w/seed CCAGUGU) | 2.810 | TargetScan Human | Moderate (predicted) | PI4KA | phosphatidylinositol 4-kinase alpha | Cytoplasm | kinase |
| MIMAT0000263 | miR-199a-5p (and other miRNAs w/seed CCAGUGU) | 2.810 | TargetScan Human | Moderate (predicted) | PIGB | hatidylinositol glycan anchor biosynthesis | Cytoplasm | enzyme |
| MIMAT0000263 | miR-199a-5p (and other miRNAs w/seed CCAGUGU) | 2.810 | TargetScan Human | High (predicted) | PIGG | hatidylinositol glycan anchor biosynthesis | Cytoplasm | enzyme |
| MIMAT0000263 | miR-199a-5p (and other miRNAs w/seed CCAGUGU) | 2.810 | TargetScan Human | Moderate (predicted) | PIGS | hatidylinositol glycan anchor biosynthesis | Cytoplasm | enzyme |
| MIMAT0000263 | miR-199a-5p (and other miRNAs w/seed CCAGUGU) | 2.810 | TargetScan Human | Moderate (predicted) | PIGZ | hatidylinositol glycan anchor biosynthesis | Cytoplasm | enzyme |
| MIMAT0000263 | miR-199a-5p (and other miRNAs w/seed CCAGUGU) | 2.810 | TargetScan Human | Moderate (predicted) | PIK3CD | hositol-4,5-bisphosphate 3-kinase catalytic | Cytoplasm | kinase |
| MIMAT0000263 | miR-199a-5p (and other miRNAs w/seed CCAGUGU) | 2.810 | TargetScan Human | Moderate (predicted) | PKN2 | protein kinase N2 | Cytoplasm | kinase |
| MIMAT0000263 | miR-199a-5p (and other miRNAs w/seed CCAGUGU) | 2.810 | TargetScan Human | Moderate (predicted) | PLA2G15 | phospholipase A2 group XV | Cytoplasm | enzyme |
| MIMAT0000263 | miR-199a-5p (and other miRNAs w/seed CCAGUGU) | 2.810 | TargetScan Human | Moderate (predicted) | PLCD4 | phospholipase C delta 4 | Cytoplasm | enzyme |
| MIMAT0000263 | miR-199a-5p (and other miRNAs w/seed CCAGUGU) | 2.810 | TargetScan Human | Moderate (predicted) | PLEK2 | pleckstrin 2 | Plasma Membrane | other |
| MIMAT0000263 | miR-199a-5p (and other miRNAs w/seed CCAGUGU) | 2.810 | TargetScan Human | Moderate (predicted) | PLEKHF2 | kstrin homology and FYVE domain contain | Cytoplasm | other |
| MIMAT0000263 | miR-199a-5p (and other miRNAs w/seed CCAGUGU) | 2.810 | TargetScan Human | Moderate (predicted) | PLXNA2 | plexin A2 | Plasma Membrane | transmembrane receptor |
| MIMAT0000263 | miR-199a-5p (and other miRNAs w/seed CCAGUGU) | 2.810 | TargetScan Human | Moderate (predicted) | PLXNC1 | plexin C1 | Plasma Membrane | transmembrane receptor |
| MIMAT0000263 | miR-199a-5p (and other miRNAs w/seed CCAGUGU) | 2.810 | TargetScan Human | Moderate (predicted) | PMP22 | peripheral myelin protein 22 | Plasma Membrane | other |
| MIMAT0000263 | miR-199a-5p (and other miRNAs w/seed CCAGUGU) | 2.810 | TargetScan Human | Moderate (predicted) | PNPLA6 | tatin like phospholipase domain containin | Cytoplasm | enzyme |
| MIMAT0000263 | miR-199a-5p (and other miRNAs w/seed CCAGUGU) | 2.810 | TargetScan Human | High (predicted) | PODXL | podocalyxin like | Plasma Membrane | kinase |
| MIMAT0000263 | miR-199a-5p (and other miRNAs w/seed CCAGUGU) | 2.810 | TargetScan Human | Moderate (predicted) | POLDIP3 | DNA polymerase delta interacting protein 3 | Nucleus | other |
| MIMAT0000263 | miR-199a-5p (and other miRNAs w/seed CCAGUGU) | 2.810 | TargetScan Human | Moderate (predicted) | POLR1G | RNA polymerase I subunit G | Nucleus | other |
| MIMAT0000263 | miR-199a-5p (and other miRNAs w/seed CCAGUGU) | 2.810 | TargetScan Human | Moderate (predicted) | POMGNT1 | d mannose N-acetylglucosaminyltransfera | Cytoplasm | enzyme |
| MIMAT0000263 | miR-199a-5p (and other miRNAs w/seed CCAGUGU) | 2.810 | TargetScan Human | Moderate (predicted) | POP4 | OP4 homolog, ribonuclease P/MRP subu | Nucleus | enzyme |
| MIMAT0000263 | miR-199a-5p (and other miRNAs w/seed CCAGUGU) | 2.810 | TargetScan Human | High (predicted) | POTEE/POTEF | POTE ankyrin domain family member E | Extracellular Space | other |
| MIMAT0000263 | miR-199a-5p (and other miRNAs w/seed CCAGUGU) | 2.810 | TargetScan Human | High (predicted) | OTEM (includes other | POTE ankyrin domain family member M | Other | other |
| MIMAT0000263 | miR-199a-5p (and other miRNAs w/seed CCAGUGU) | 2.810 | TargetScan Human | Moderate (predicted) | POU3F2 | POU class 3 homeobox 2 | Nucleus | transcription regulator |
| MIMAT0000263 | miR-199a-5p (and other miRNAs w/seed CCAGUGU) | 2.810 | TargetScan Human | High (predicted) | PPARGC1A | PPARG coactivator 1 alpha | Nucleus | transcription regulator |
| MIMAT0000263 | miR-199a-5p (and other miRNAs w/seed CCAGUGU) | 2.810 | TargetScan Human | Moderate (predicted) | PPFIBP1 | PPFIA binding protein 1 | Plasma Membrane | other |
| MIMAT0000263 | miR-199a-5p (and other miRNAs w/seed CCAGUGU) | 2.810 | TargetScan Human | High (predicted) | PPP1R2 | ein phosphatase 1 regulatory inhibitor subu | Cytoplasm | phosphatase |
| MIMAT0000263 | miR-199a-5p (and other miRNAs w/seed CCAGUGU) | 2.810 | TargetScan Human | High (predicted) | PPP1R9A | rotein phosphatase 1 regulatory subunit 9 | Plasma Membrane | other |
| MIMAT0000263 | miR-199a-5p (and other miRNAs w/seed CCAGUGU) | 2.810 | TargetScan Human | Moderate (predicted) | PPP2R1A | otein phosphatase 2 scaffold subunit Aalp | Cytoplasm | phosphatase |
| MIMAT0000263 | miR-199a-5p (and other miRNAs w/seed CCAGUGU) | 2.810 | TargetScan Human | Moderate (predicted) | PRDM5 | PR/SET domain 5 | Nucleus | transcription regulator |
| MIMAT0000263 | miR-199a-5p (and other miRNAs w/seed CCAGUGU) | 2.810 | TargetScan Human | Moderate (predicted) | PREPL | prolyl endopeptidase like | Cytoplasm | peptidase |
| MIMAT0000263 | miR-199a-5p (and other miRNAs w/seed CCAGUGU) | 2.810 | TargetScan Human | Moderate (predicted) | PRKAG2 | nase AMP-activated non-catalytic subunit | Cytoplasm | kinase |
| MIMAT0000263 | miR-199a-5p (and other miRNAs w/seed CCAGUGU) | 2.810 | TargetScan Human | Moderate (predicted) | PRKCI | protein kinase C iota | Cytoplasm | kinase |
| MIMAT0000263 | miR-199a-5p (and other miRNAs w/seed CCAGUGU) | 2.810 | TargetScan Human | Moderate (predicted) | PRPF40A | pre-mRNA processing factor 40 homolog A | Nucleus | other |
| MIMAT0000263 | miR-199a-5p (and other miRNAs w/seed CCAGUGU) | 2.810 | TargetScan Human | Moderate (predicted) | PRR35 | proline rich 35 | Other | other |
| MIMAT0000263 | miR-199a-5p (and other miRNAs w/seed CCAGUGU) | 2.810 | TargetScan Human | Moderate (predicted) | PRSS54 | serine protease 54 | Cytoplasm | other |
| MIMAT0000263 | miR-199a-5p (and other miRNAs w/seed CCAGUGU) | 2.810 | TargetScan Human | Moderate (predicted) | PTCHD1 | patched domain containing 1 | Plasma Membrane | other |
| MIMAT0000263 | miR-199a-5p (and other miRNAs w/seed CCAGUGU) | 2.810 | TargetScan Human | Moderate (predicted) | PUDP | pseudouridine 5'-phosphatase | Cytoplasm | enzyme |
| MIMAT0000263 | miR-199a-5p (and other miRNAs w/seed CCAGUGU) | 2.810 | TargetScan Human | Moderate (predicted) | PXN | paxillin | Cytoplasm | other |
| MIMAT0000263 | miR-199a-5p (and other miRNAs w/seed CCAGUGU) | 2.810 | TargetScan Human | High (predicted) | R3HDM2 | R3H domain containing 2 | Nucleus | other |
| MIMAT0000263 | miR-199a-5p (and other miRNAs w/seed CCAGUGU) | 2.810 | TargetScan Human | Moderate (predicted) | RAB12 | RAB12, member RAS oncogene family | Cytoplasm | enzyme |
| MIMAT0000263 | miR-199a-5p (and other miRNAs w/seed CCAGUGU) | 2.810 | TargetScan Human | Moderate (predicted) | RAB21 | RAB21, member RAS oncogene family | Cytoplasm | enzyme |
| MIMAT0000263 | miR-199a-5p (and other miRNAs w/seed CCAGUGU) | 2.810 | TargetScan Human | Moderate (predicted) | RAB27B | RAB27B, member RAS oncogene family | Cytoplasm | enzyme |
| MIMAT0000263 | miR-199a-5p (and other miRNAs w/seed CCAGUGU) | 2.810 | TargetScan Human | Moderate (predicted) | RAB33A | RAB33A, member RAS oncogene family | Cytoplasm | enzyme |
| MIMAT0000263 | miR-199a-5p (and other miRNAs w/seed CCAGUGU) | 2.810 | TargetScan Human | High (predicted) | RAB9B | RAB9B, member RAS oncogene family | Plasma Membrane | enzyme |
| MIMAT0000263 | miR-199a-5p (and other miRNAs w/seed CCAGUGU) | 2.810 | TargetScan Human | Moderate (predicted) | RAD21L1 | RAD21 cohesin complex component like 1 | Nucleus | other |
| MIMAT0000263 | miR-199a-5p (and other miRNAs w/seed CCAGUGU) | 2.810 | TargetScan Human | High (predicted) | RAD23B | 23 homolog B, nucleotide excision repair p | Nucleus | other |
| MIMAT0000263 | miR-199a-5p (and other miRNAs w/seed CCAGUGU) | 2.810 | TargetScan Human | High (predicted) | RALGAPA1 | TPase activating protein catalytic subunit a | Cytoplasm | other |
| MIMAT0000263 | miR-199a-5p (and other miRNAs w/seed CCAGUGU) | 2.810 | TargetScan Human | Moderate (predicted) | RANBP2 | RAN binding protein 2 | Nucleus | enzyme |
| MIMAT0000263 | miR-199a-5p (and other miRNAs w/seed CCAGUGU) | 2.810 | TargetScan Human | Moderate (predicted) | RASL10A | RAS like family 10 member A | Plasma Membrane | enzyme |
| MIMAT0000263 | miR-199a-5p (and other miRNAs w/seed CCAGUGU) | 2.810 | TargetScan Human | High (predicted) | RASSF2 | Ras association domain family member 2 | Nucleus | kinase |
| MIMAT0000263 | miR-199a-5p (and other miRNAs w/seed CCAGUGU) | 2.810 | TargetScan Human | Moderate (predicted) | RASSF3 | Ras association domain family member 3 | Other | other |
| MIMAT0000263 | miR-199a-5p (and other miRNAs w/seed CCAGUGU) | 2.810 | TargetScan Human | Moderate (predicted) | RAX | retina and anterior neural fold homeobox | Nucleus | transcription regulator |
| MIMAT0000263 | miR-199a-5p (and other miRNAs w/seed CCAGUGU) | 2.810 | TargetScan Human | Moderate (predicted) | RBBP4 | binding protein 4, chromatin remodeling fa | Nucleus | enzyme |
| MIMAT0000263 | miR-199a-5p (and other miRNAs w/seed CCAGUGU) | 2.810 | TargetScan Human | Moderate (predicted) | RBM23 | RNA binding motif protein 23 | Nucleus | other |
| MIMAT0000263 | miR-199a-5p (and other miRNAs w/seed CCAGUGU) | 2.810 | TargetScan Human | Moderate (predicted) | RBM33 | RNA binding motif protein 33 | Other | other |
| MIMAT0000263 | miR-199a-5p (and other miRNAs w/seed CCAGUGU) | 2.810 | TargetScan Human | High (predicted) | RBM47 | RNA binding motif protein 47 | Nucleus | other |
| MIMAT0000263 | miR-199a-5p (and other miRNAs w/seed CCAGUGU) | 2.810 | TargetScan Human | Moderate (predicted) | RBMXL1 | RBMX like 1 | Nucleus | other |
| MIMAT0000263 | miR-199a-5p (and other miRNAs w/seed CCAGUGU) | 2.810 | TargetScan Human | Moderate (predicted) | RBPMS | NA binding protein, mRNA processing fac | Cytoplasm | transcription regulator |
| MIMAT0000263 | miR-199a-5p (and other miRNAs w/seed CCAGUGU) | 2.810 | TargetScan Human | Moderate (predicted) | RCHY1 | finger and CHY zinc finger domain contain | Nucleus | enzyme |
| MIMAT0000263 | miR-199a-5p (and other miRNAs w/seed CCAGUGU) | 2.810 | TargetScan Human | High (predicted) | RCSD1 | RCSD domain containing 1 | Other | other |
| MIMAT0000263 | miR-199a-5p (and other miRNAs w/seed CCAGUGU) | 2.810 | TargetScan Human | Moderate (predicted) | REG4 | regenerating family member 4 | Extracellular Space | other |
| MIMAT0000263 | miR-199a-5p (and other miRNAs w/seed CCAGUGU) | 2.810 | TargetScan Human | Moderate (predicted) | RFX3 | regulatory factor X3 | Nucleus | transcription regulator |
| MIMAT0000263 | miR-199a-5p (and other miRNAs w/seed CCAGUGU) | 2.810 | TargetScan Human | High (predicted) | RGMA | pulsive guidance molecule BMP co-recept | Plasma Membrane | other |
| MIMAT0000263 | miR-199a-5p (and other miRNAs w/seed CCAGUGU) | 2.810 | TargetScan Human | Moderate (predicted) | RGS16 | regulator of G protein signaling 16 | Cytoplasm | enzyme |
| MIMAT0000263 | miR-199a-5p (and other miRNAs w/seed CCAGUGU) | 2.810 | TargetScan Human | Moderate (predicted) | RGS17 | regulator of G protein signaling 17 | Cytoplasm | enzyme |
| MIMAT0000263 | miR-199a-5p (and other miRNAs w/seed CCAGUGU) | 2.810 | TargetScan Human | Moderate (predicted) | RGS6 | regulator of G protein signaling 6 | Cytoplasm | enzyme |
| MIMAT0000263 | miR-199a-5p (and other miRNAs w/seed CCAGUGU) | 2.810 | TargetScan Human | Moderate (predicted) | RHEB | Ras homolog, mTORC1 binding | Cytoplasm | enzyme |
| MIMAT0000263 | miR-199a-5p (and other miRNAs w/seed CCAGUGU) | 2.810 | TargetScan Human | Moderate (predicted) | RHOQ | ras homolog family member Q | Plasma Membrane | enzyme |
| MIMAT0000263 | miR-199a-5p (and other miRNAs w/seed CCAGUGU) | 2.810 | TargetScan Human | Moderate (predicted) | RIC8A | RIC8 guanine nucleotide exchange factor A | Cytoplasm | other |
| MIMAT0000263 | miR-199a-5p (and other miRNAs w/seed CCAGUGU) | 2.810 | TargetScan Human | Moderate (predicted) | RLIM | ring finger protein, LIM domain interacting | Nucleus | enzyme |
| MIMAT0000263 | miR-199a-5p (and other miRNAs w/seed CCAGUGU) | 2.810 | TargetScan Human | High (predicted) | RND1 | Rho family GTPase 1 | Cytoplasm | enzyme |
| MIMAT0000263 | miR-199a-5p (and other miRNAs w/seed CCAGUGU) | 2.810 | TargetScan Human | Moderate (predicted) | RNF11 | ring finger protein 11 | Cytoplasm | enzyme |
| MIMAT0000263 | miR-199a-5p (and other miRNAs w/seed CCAGUGU) | 2.810 | TargetScan Human | Moderate (predicted) | RNF113B | ring finger protein 113B | Other | other |
| MIMAT0000263 | miR-199a-5p (and other miRNAs w/seed CCAGUGU) | 2.810 | TargetScan Human | Moderate (predicted) | RNF114 | ring finger protein 114 | Cytoplasm | transcription regulator |
| MIMAT0000263 | miR-199a-5p (and other miRNAs w/seed CCAGUGU) | 2.810 | TargetScan Human | Moderate (predicted) | RNF183 | ring finger protein 183 | Cytoplasm | enzyme |
| MIMAT0000263 | miR-199a-5p (and other miRNAs w/seed CCAGUGU) | 2.810 | TargetScan Human | Moderate (predicted) | RNF207 | ring finger protein 207 | Other | other |
| MIMAT0000263 | miR-199a-5p (and other miRNAs w/seed CCAGUGU) | 2.810 | TargetScan Human | Moderate (predicted) | RNF38 | ring finger protein 38 | Nucleus | enzyme |
| MIMAT0000263 | miR-199a-5p (and other miRNAs w/seed CCAGUGU) | 2.810 | TargetScan Human | Moderate (predicted) | ROGDI | rogdi atypical leucine zipper | Nucleus | other |
| MIMAT0000263 | miR-199a-5p (and other miRNAs w/seed CCAGUGU) | 2.810 | TargetScan Human | Moderate (predicted) | RORB | RAR related orphan receptor B | Nucleus | ligand-dependent nuclear receptor |
| MIMAT0000263 | miR-199a-5p (and other miRNAs w/seed CCAGUGU) | 2.810 | TargetScan Human | Moderate (predicted) | RP11 126H74 | | Other | other |
| MIMAT0000263 | miR-199a-5p (and other miRNAs w/seed CCAGUGU) | 2.810 | TargetScan Human | High (predicted) | RP11 763F81 | | Other | other |
| MIMAT0000263 | miR-199a-5p (and other miRNAs w/seed CCAGUGU) | 2.810 | TargetScan Human | Moderate (predicted) | RP11 94B194 | | Other | other |
| MIMAT0000263 | miR-199a-5p (and other miRNAs w/seed CCAGUGU) | 2.810 | TargetScan Human | High (predicted) | RP11-111H13.1 | | Other | other |
| MIMAT0000263 | miR-199a-5p (and other miRNAs w/seed CCAGUGU) | 2.810 | TargetScan Human | Moderate (predicted) | RPL37 | ribosomal protein L37 | Cytoplasm | other |
| MIMAT0000263 | miR-199a-5p (and other miRNAs w/seed CCAGUGU) | 2.810 | TargetScan Human | High (predicted) | RPUSD3 | RNA pseudouridine synthase D3 | Cytoplasm | other |
| MIMAT0000263 | miR-199a-5p (and other miRNAs w/seed CCAGUGU) | 2.810 | TargetScan Human | High (predicted) | RSPH4A | radial spoke head component 4A | Nucleus | other |
| MIMAT0000263 | miR-199a-5p (and other miRNAs w/seed CCAGUGU) | 2.810 | TargetScan Human | Moderate (predicted) | RSPRY1 | ring finger and SPRY domain containing 1 | Other | other |
| MIMAT0000263 | miR-199a-5p (and other miRNAs w/seed CCAGUGU) | 2.810 | TargetScan Human | Moderate (predicted) | RTL8C | retrotransposon Gag like 8C | Plasma Membrane | other |
| MIMAT0000263 | miR-199a-5p (and other miRNAs w/seed CCAGUGU) | 2.810 | TargetScan Human | Moderate (predicted) | RTN4RL2 | reticulon 4 receptor like 2 | Plasma Membrane | other |
| MIMAT0000263 | miR-199a-5p (and other miRNAs w/seed CCAGUGU) | 2.810 | TargetScan Human | Moderate (predicted) | RTP2 | receptor transporter protein 2 | Plasma Membrane | other |
| MIMAT0000263 | miR-199a-5p (and other miRNAs w/seed CCAGUGU) | 2.810 | TargetScan Human | Moderate (predicted) | RTP4 | receptor transporter protein 4 | Plasma Membrane | other |
| MIMAT0000263 | miR-199a-5p (and other miRNAs w/seed CCAGUGU) | 2.810 | TargetScan Human | Moderate (predicted) | RUNX1T1 | UNX1 partner transcriptional co-repressor | Nucleus | transcription regulator |
| MIMAT0000263 | miR-199a-5p (and other miRNAs w/seed CCAGUGU) | 2.810 | TargetScan Human | Moderate (predicted) | RXRB | retinoid X receptor beta | Nucleus | ligand-dependent nuclear receptor |
| MIMAT0000263 | miR-199a-5p (and other miRNAs w/seed CCAGUGU) | 2.810 | TargetScan Human | Moderate (predicted) | S100G | S100 calcium binding protein G | Cytoplasm | other |
| MIMAT0000263 | miR-199a-5p (and other miRNAs w/seed CCAGUGU) | 2.810 | TargetScan Human | Moderate (predicted) | SACS | sacsin molecular chaperone | Plasma Membrane | other |
| MIMAT0000263 | miR-199a-5p (and other miRNAs w/seed CCAGUGU) | 2.810 | TargetScan Human | High (predicted) | SARAF | perated calcium entry associated regulator | Cytoplasm | other |
| MIMAT0000263 | miR-199a-5p (and other miRNAs w/seed CCAGUGU) | 2.810 | TargetScan Human | Moderate (predicted) | SAT1 | permidine/spermine N1-acetyltransferase | Cytoplasm | enzyme |
| MIMAT0000263 | miR-199a-5p (and other miRNAs w/seed CCAGUGU) | 2.810 | TargetScan Human | High (predicted) | SCGN | cretagogin, EF-hand calcium binding prot | Cytoplasm | other |
| MIMAT0000263 | miR-199a-5p (and other miRNAs w/seed CCAGUGU) | 2.810 | TargetScan Human | Moderate (predicted) | SCNN1G | sodium channel epithelial 1 subunit gamm | Plasma Membrane | ion channel |
| MIMAT0000263 | miR-199a-5p (and other miRNAs w/seed CCAGUGU) | 2.810 | TargetScan Human | Moderate (predicted) | SDCBP | syndecan binding protein | Plasma Membrane | enzyme |
| MIMAT0000263 | miR-199a-5p (and other miRNAs w/seed CCAGUGU) | 2.810 | TargetScan Human | Moderate (predicted) | SEC22C | EC22 homolog C, vesicle trafficking prote | Cytoplasm | other |
| MIMAT0000263 | miR-199a-5p (and other miRNAs w/seed CCAGUGU) | 2.810 | TargetScan Human | Moderate (predicted) | SEC24C | 24 homolog C, COPII coat complex comp | Cytoplasm | transporter |
| MIMAT0000263 | miR-199a-5p (and other miRNAs w/seed CCAGUGU) | 2.810 | TargetScan Human | Moderate (predicted) | SEMA3F | semaphorin 3F | Extracellular Space | other |
| MIMAT0000263 | miR-199a-5p (and other miRNAs w/seed CCAGUGU) | 2.810 | TargetScan Human | Moderate (predicted) | SEMA4D | semaphorin 4D | Plasma Membrane | transmembrane receptor |
| MIMAT0000263 | miR-199a-5p (and other miRNAs w/seed CCAGUGU) | 2.810 | TargetScan Human | Moderate (predicted) | SERBP1 | SERPINE1 mRNA binding protein 1 | Cytoplasm | other |
| MIMAT0000263 | miR-199a-5p (and other miRNAs w/seed CCAGUGU) | 2.810 | TargetScan Human | Moderate (predicted) | SERINC3 | serine incorporator 3 | Cytoplasm | transporter |
| MIMAT0000263 | miR-199a-5p (and other miRNAs w/seed CCAGUGU) | 2.810 | TargetScan Human | Moderate (predicted) | SESN1 | sestrin 1 | Nucleus | other |
| MIMAT0000263 | miR-199a-5p (and other miRNAs w/seed CCAGUGU) | 2.810 | getScan Human,miRec | ntally Observed,Moderate ( | SET | SET nuclear proto-oncogene | Nucleus | phosphatase |
| MIMAT0000263 | miR-199a-5p (and other miRNAs w/seed CCAGUGU) | 2.810 | TargetScan Human | Moderate (predicted) | SGMS2 | sphingomyelin synthase 2 | Plasma Membrane | enzyme |
| MIMAT0000263 | miR-199a-5p (and other miRNAs w/seed CCAGUGU) | 2.810 | TargetScan Human | Moderate (predicted) | SGPP2 | sphingosine-1-phosphate phosphatase 2 | Cytoplasm | phosphatase |
| MIMAT0000263 | miR-199a-5p (and other miRNAs w/seed CCAGUGU) | 2.810 | TargetScan Human | Moderate (predicted) | SGSM2 | small G protein signaling modulator 2 | Cytoplasm | other |
| MIMAT0000263 | miR-199a-5p (and other miRNAs w/seed CCAGUGU) | 2.810 | TargetScan Human | High (predicted) | SHOC2 | SHOC2 leucine rich repeat scaffold protein | Cytoplasm | other |
| MIMAT0000263 | miR-199a-5p (and other miRNAs w/seed CCAGUGU) | 2.810 | TargetScan Human | Moderate (predicted) | SIRPB2 | signal regulatory protein beta 2 | Other | other |
| MIMAT0000263 | miR-199a-5p (and other miRNAs w/seed CCAGUGU) | 2.810 | getScan Human,miRec | ntally Observed,Moderate ( | SIRT1 | sirtuin 1 | Nucleus | transcription regulator |
| MIMAT0000263 | miR-199a-5p (and other miRNAs w/seed CCAGUGU) | 2.810 | TargetScan Human | High (predicted) | SLC10A4 | solute carrier family 10 member 4 | Plasma Membrane | transporter |
| MIMAT0000263 | miR-199a-5p (and other miRNAs w/seed CCAGUGU) | 2.810 | TargetScan Human | High (predicted) | SLC1A5 | solute carrier family 1 member 5 | Plasma Membrane | transporter |
| MIMAT0000263 | miR-199a-5p (and other miRNAs w/seed CCAGUGU) | 2.810 | TargetScan Human | High (predicted) | SLC24A3 | solute carrier family 24 member 3 | Plasma Membrane | transporter |
| MIMAT0000263 | miR-199a-5p (and other miRNAs w/seed CCAGUGU) | 2.810 | TargetScan Human | Moderate (predicted) | SLC24A4 | solute carrier family 24 member 4 | Plasma Membrane | transporter |
| MIMAT0000263 | miR-199a-5p (and other miRNAs w/seed CCAGUGU) | 2.810 | TargetScan Human | Moderate (predicted) | SLC25A13 | solute carrier family 25 member 13 | Cytoplasm | transporter |
| MIMAT0000263 | miR-199a-5p (and other miRNAs w/seed CCAGUGU) | 2.810 | TargetScan Human | High (predicted) | SLC25A23 | solute carrier family 25 member 23 | Cytoplasm | transporter |
| MIMAT0000263 | miR-199a-5p (and other miRNAs w/seed CCAGUGU) | 2.810 | TargetScan Human | Moderate (predicted) | SLC35A2 | solute carrier family 35 member A2 | Cytoplasm | enzyme |
| MIMAT0000263 | miR-199a-5p (and other miRNAs w/seed CCAGUGU) | 2.810 | TargetScan Human | Moderate (predicted) | SLC35B3 | solute carrier family 35 member B3 | Cytoplasm | transporter |
| MIMAT0000263 | miR-199a-5p (and other miRNAs w/seed CCAGUGU) | 2.810 | TargetScan Human | Moderate (predicted) | SLC35D1 | solute carrier family 35 member D1 | Cytoplasm | transporter |
| MIMAT0000263 | miR-199a-5p (and other miRNAs w/seed CCAGUGU) | 2.810 | TargetScan Human | Moderate (predicted) | SLC35E1 | solute carrier family 35 member E1 | Cytoplasm | other |
| MIMAT0000263 | miR-199a-5p (and other miRNAs w/seed CCAGUGU) | 2.810 | TargetScan Human | Moderate (predicted) | SLC35F1 | solute carrier family 35 member F1 | Other | other |
| MIMAT0000263 | miR-199a-5p (and other miRNAs w/seed CCAGUGU) | 2.810 | TargetScan Human | Moderate (predicted) | SLC3A1 | solute carrier family 3 member 1 | Plasma Membrane | transporter |
| MIMAT0000263 | miR-199a-5p (and other miRNAs w/seed CCAGUGU) | 2.810 | TargetScan Human | Moderate (predicted) | SLC9A8 | solute carrier family 9 member A8 | Cytoplasm | transporter |
| MIMAT0000263 | miR-199a-5p (and other miRNAs w/seed CCAGUGU) | 2.810 | TargetScan Human | Moderate (predicted) | SLF2 | SMC5-SMC6 complex localization factor 2 | Extracellular Space | other |
| MIMAT0000263 | miR-199a-5p (and other miRNAs w/seed CCAGUGU) | 2.810 | TargetScan Human | Moderate (predicted) | SMARCAD1 | actin-dependent regulator of chromatin, su | Nucleus | enzyme |
| MIMAT0000263 | miR-199a-5p (and other miRNAs w/seed CCAGUGU) | 2.810 | TargetScan Human | Moderate (predicted) | SMARCD1 | ociated, actin dependent regulator of chror | Nucleus | transcription regulator |
| MIMAT0000263 | miR-199a-5p (and other miRNAs w/seed CCAGUGU) | 2.810 | TargetScan Human | Moderate (predicted) | SMIM12 | small integral membrane protein 12 | Other | other |
| MIMAT0000263 | miR-199a-5p (and other miRNAs w/seed CCAGUGU) | 2.810 | TargetScan Human | Moderate (predicted) | SMKR1 | small lysine rich protein 1 | Other | other |
| MIMAT0000263 | miR-199a-5p (and other miRNAs w/seed CCAGUGU) | 2.810 | TargetScan Human | Moderate (predicted) | SMLR1 | small leucine rich protein 1 | Other | other |
| MIMAT0000263 | miR-199a-5p (and other miRNAs w/seed CCAGUGU) | 2.810 | TargetScan Human | High (predicted) | SNAI1 | snail family transcriptional repressor 1 | Nucleus | transcription regulator |
| MIMAT0000263 | miR-199a-5p (and other miRNAs w/seed CCAGUGU) | 2.810 | TargetScan Human | Moderate (predicted) | SNAPIN | SNAP associated protein | Plasma Membrane | other |
| MIMAT0000263 | miR-199a-5p (and other miRNAs w/seed CCAGUGU) | 2.810 | TargetScan Human | Moderate (predicted) | SNX6 | sorting nexin 6 | Cytoplasm | transporter |
| MIMAT0000263 | miR-199a-5p (and other miRNAs w/seed CCAGUGU) | 2.810 | TargetScan Human | Moderate (predicted) | SORCS3 | in related VPS10 domain containing recep | Nucleus | transporter |
| MIMAT0000263 | miR-199a-5p (and other miRNAs w/seed CCAGUGU) | 2.810 | TargetScan Human | High (predicted) | SOS2 | Ras/Rho guanine nucleotide exchange fa | Cytoplasm | other |
| MIMAT0000263 | miR-199a-5p (and other miRNAs w/seed CCAGUGU) | 2.810 | TargetScan Human | Moderate (predicted) | SPATA12 | spermatogenesis associated 12 | Other | other |
| MIMAT0000263 | miR-199a-5p (and other miRNAs w/seed CCAGUGU) | 2.810 | TargetScan Human | Moderate (predicted) | SPCS2 | signal peptidase complex subunit 2 | Cytoplasm | other |

Sup. Table 3. List of 3699 targets of the seven dysregulated miRNAs obtained from three different target prediction databases,TargetScan TarBase and miRecords considering those predicted as moderate, high, or experimentally validated, respectively.

| | | | | | | | | |
|---|---|---|---|---|---|---|---|---|
| MIMAT0000263 | miR-199a-5p (and other miRNAs w/seed CCAGUGU) | 2.810 | TargetScan Human | Moderate (predicted) | SRGAP3 | LIT-ROBO Rho GTPase activating protein | Cytoplasm | other |
| MIMAT0000263 | miR-199a-5p (and other miRNAs w/seed CCAGUGU) | 2.810 | TargetScan Human | High (predicted) | SRRM1 | serine and arginine repetitive matrix 1 | Nucleus | other |
| MIMAT0000263 | miR-199a-5p (and other miRNAs w/seed CCAGUGU) | 2.810 | TargetScan Human | Moderate (predicted) | ST7-OT4 | ST7 overlapping transcript 4 | Other | other |
| MIMAT0000263 | miR-199a-5p (and other miRNAs w/seed CCAGUGU) | 2.810 | TargetScan Human | Moderate (predicted) | STAG1 | stromal antigen 1 | Nucleus | other |
| MIMAT0000263 | miR-199a-5p (and other miRNAs w/seed CCAGUGU) | 2.810 | TargetScan Human | Moderate (predicted) | STK4 | serine/threonine kinase 4 | Cytoplasm | kinase |
| MIMAT0000263 | miR-199a-5p (and other miRNAs w/seed CCAGUGU) | 2.810 | TargetScan Human | Moderate (predicted) | STRADB | STE20 related adaptor beta | Cytoplasm | kinase |
| MIMAT0000263 | miR-199a-5p (and other miRNAs w/seed CCAGUGU) | 2.810 | TargetScan Human | Moderate (predicted) | STX12 | syntaxin 12 | Cytoplasm | other |
| MIMAT0000263 | miR-199a-5p (and other miRNAs w/seed CCAGUGU) | 2.810 | TargetScan Human | Moderate (predicted) | STX4 | syntaxin 4 | Plasma Membrane | transporter |
| MIMAT0000263 | miR-199a-5p (and other miRNAs w/seed CCAGUGU) | 2.810 | TargetScan Human | Moderate (predicted) | STXBP5L | syntaxin binding protein 5 like | Cytoplasm | other |
| MIMAT0000263 | miR-199a-5p (and other miRNAs w/seed CCAGUGU) | 2.810 | TargetScan Human | Moderate (predicted) | SUCLG1 | ate-CoA ligase GDP/ADP-forming subuni | Cytoplasm | enzyme |
| MIMAT0000263 | miR-199a-5p (and other miRNAs w/seed CCAGUGU) | 2.810 | TargetScan Human | Moderate (predicted) | SUCNR1 | succinate receptor 1 | Plasma Membrane | G-protein coupled receptor |
| MIMAT0000263 | miR-199a-5p (and other miRNAs w/seed CCAGUGU) | 2.810 | TargetScan Human | Moderate (predicted) | SUCO | SUN domain containing ossification factor | Other | other |
| MIMAT0000263 | miR-199a-5p (and other miRNAs w/seed CCAGUGU) | 2.810 | TargetScan Human | Moderate (predicted) | SUGCT | succinyl-CoA:glutarate-CoA transferase | Cytoplasm | enzyme |
| MIMAT0000263 | miR-199a-5p (and other miRNAs w/seed CCAGUGU) | 2.810 | TargetScan Human | High (predicted) | SULF1 | sulfatase 1 | Cytoplasm | enzyme |
| MIMAT0000263 | miR-199a-5p (and other miRNAs w/seed CCAGUGU) | 2.810 | TargetScan Human | Moderate (predicted) | SULT4A1 | sulfotransferase family 4A member 1 | Cytoplasm | enzyme |
| MIMAT0000263 | miR-199a-5p (and other miRNAs w/seed CCAGUGU) | 2.810 | TargetScan Human | High (predicted) | SUN1 | Sad1 and UNC84 domain containing 1 | Nucleus | other |
| MIMAT0000263 | miR-199a-5p (and other miRNAs w/seed CCAGUGU) | 2.810 | TargetScan Human | Moderate (predicted) | SYT11 | synaptotagmin 11 | Cytoplasm | transporter |
| MIMAT0000263 | miR-199a-5p (and other miRNAs w/seed CCAGUGU) | 2.810 | TargetScan Human | Moderate (predicted) | SYTL4 | synaptotagmin like 4 | Cytoplasm | transporter |
| MIMAT0000263 | miR-199a-5p (and other miRNAs w/seed CCAGUGU) | 2.810 | TargetScan Human | Moderate (predicted) | TAB3 | eta activated kinase 1 (MAP3K7) binding p | Cytoplasm | other |
| MIMAT0000263 | miR-199a-5p (and other miRNAs w/seed CCAGUGU) | 2.810 | TargetScan Human | Moderate (predicted) | TAF8 | ATA-box binding protein associated factor | Nucleus | transcription regulator |
| MIMAT0000263 | miR-199a-5p (and other miRNAs w/seed CCAGUGU) | 2.810 | TargetScan Human | High (predicted) | TAF9B | ATA-box binding protein associated factor | Nucleus | transcription regulator |
| MIMAT0000263 | miR-199a-5p (and other miRNAs w/seed CCAGUGU) | 2.810 | TargetScan Human | Moderate (predicted) | TAFA2 | TAFA chemokine like family member 2 | Cytoplasm | other |
| MIMAT0000263 | miR-199a-5p (and other miRNAs w/seed CCAGUGU) | 2.810 | TargetScan Human | Moderate (predicted) | TAOK1 | TAO kinase 1 | Cytoplasm | kinase |
| MIMAT0000263 | miR-199a-5p (and other miRNAs w/seed CCAGUGU) | 2.810 | TargetScan Human | Moderate (predicted) | TAT | tyrosine aminotransferase | Cytoplasm | enzyme |
| MIMAT0000263 | miR-199a-5p (and other miRNAs w/seed CCAGUGU) | 2.810 | TargetScan Human | Moderate (predicted) | TBC1D14 | TBC1 domain family member 14 | Extracellular Space | other |
| MIMAT0000263 | miR-199a-5p (and other miRNAs w/seed CCAGUGU) | 2.810 | TargetScan Human | Moderate (predicted) | TBC1D26 | TBC1 domain family member 26 | Other | other |
| MIMAT0000263 | miR-199a-5p (and other miRNAs w/seed CCAGUGU) | 2.810 | TargetScan Human | Moderate (predicted) | TBC1D5 | TBC1 domain family member 5 | Extracellular Space | other |
| MIMAT0000263 | miR-199a-5p (and other miRNAs w/seed CCAGUGU) | 2.810 | TargetScan Human | Moderate (predicted) | TBC1D8 | TBC1 domain family member 8 | Plasma Membrane | other |
| MIMAT0000263 | miR-199a-5p (and other miRNAs w/seed CCAGUGU) | 2.810 | TargetScan Human | Moderate (predicted) | TBCE | tubulin folding cofactor E | Cytoplasm | other |
| MIMAT0000263 | miR-199a-5p (and other miRNAs w/seed CCAGUGU) | 2.810 | TargetScan Human | Moderate (predicted) | TCP10L | t-complex 10 like | Nucleus | transcription regulator |
| MIMAT0000263 | miR-199a-5p (and other miRNAs w/seed CCAGUGU) | 2.810 | TargetScan Human | Moderate (predicted) | TCTA | T cell leukemia translocation altered | Other | other |
| MIMAT0000263 | miR-199a-5p (and other miRNAs w/seed CCAGUGU) | 2.810 | TargetScan Human | Moderate (predicted) | TCTE1 | t-complex-associated-testis-expressed 1 | Other | other |
| MIMAT0000263 | miR-199a-5p (and other miRNAs w/seed CCAGUGU) | 2.810 | TargetScan Human | Moderate (predicted) | TEP1 | telomerase associated protein 1 | Nucleus | enzyme |
| MIMAT0000263 | miR-199a-5p (and other miRNAs w/seed CCAGUGU) | 2.810 | TargetScan Human | Moderate (predicted) | TERB1 | here repeat binding bouquet formation pro | Nucleus | peptidase |
| MIMAT0000263 | miR-199a-5p (and other miRNAs w/seed CCAGUGU) | 2.810 | TargetScan Human | High (predicted) | TESPA1 | ocyte expressed, positive selection associ | Cytoplasm | other |
| MIMAT0000263 | miR-199a-5p (and other miRNAs w/seed CCAGUGU) | 2.810 | TargetScan Human | Moderate (predicted) | TET2 | tet methylcytosine dioxygenase 2 | Nucleus | enzyme |
| MIMAT0000263 | miR-199a-5p (and other miRNAs w/seed CCAGUGU) | 2.810 | TargetScan Human | High (predicted) | TFPI | tissue factor pathway inhibitor | Extracellular Space | other |
| MIMAT0000263 | miR-199a-5p (and other miRNAs w/seed CCAGUGU) | 2.810 | TargetScan Human | High (predicted) | TGFB2 | transforming growth factor beta 2 | Extracellular Space | growth factor |
| MIMAT0000263 | miR-199a-5p (and other miRNAs w/seed CCAGUGU) | 2.810 | TargetScan Human | Moderate (predicted) | THUMPD3 | THUMP domain containing 3 | Other | other |
| MIMAT0000263 | miR-199a-5p (and other miRNAs w/seed CCAGUGU) | 2.810 | TargetScan Human | Moderate (predicted) | TIGD5 | tigger transposable element derived 5 | Other | other |
| MIMAT0000263 | miR-199a-5p (and other miRNAs w/seed CCAGUGU) | 2.810 | TargetScan Human | Moderate (predicted) | TIMM29 | nslocase of inner mitochondrial membrane | Cytoplasm | transporter |
| MIMAT0000263 | miR-199a-5p (and other miRNAs w/seed CCAGUGU) | 2.810 | TargetScan Human | Moderate (predicted) | TIMMDC1 | of inner mitochondrial membrane domain | Cytoplasm | other |
| MIMAT0000263 | miR-199a-5p (and other miRNAs w/seed CCAGUGU) | 2.810 | TargetScan Human | Moderate (predicted) | TLCD1 | TLC domain containing 1 | Plasma Membrane | other |
| MIMAT0000263 | miR-199a-5p (and other miRNAs w/seed CCAGUGU) | 2.810 | TargetScan Human | High (predicted) | TLDC2 | TBC/LysM-associated domain containing 2 | Other | other |
| MIMAT0000263 | miR-199a-5p (and other miRNAs w/seed CCAGUGU) | 2.810 | TargetScan Human | Moderate (predicted) | TM9SF3 | transmembrane 9 superfamily member 3 | Cytoplasm | transporter |
| MIMAT0000263 | miR-199a-5p (and other miRNAs w/seed CCAGUGU) | 2.810 | TargetScan Human | Moderate (predicted) | TMED8 | embrane p24 trafficking protein family me | Other | other |
| MIMAT0000263 | miR-199a-5p (and other miRNAs w/seed CCAGUGU) | 2.810 | TargetScan Human | Moderate (predicted) | TMEM135 | transmembrane protein 135 | Cytoplasm | other |
| MIMAT0000263 | miR-199a-5p (and other miRNAs w/seed CCAGUGU) | 2.810 | TargetScan Human | Moderate (predicted) | TMEM211 | transmembrane protein 211 | Other | other |
| MIMAT0000263 | miR-199a-5p (and other miRNAs w/seed CCAGUGU) | 2.810 | TargetScan Human | Moderate (predicted) | TMEM215 | transmembrane protein 215 | Other | other |
| MIMAT0000263 | miR-199a-5p (and other miRNAs w/seed CCAGUGU) | 2.810 | TargetScan Human | Moderate (predicted) | TMEM229A | transmembrane protein 229A | Other | other |
| MIMAT0000263 | miR-199a-5p (and other miRNAs w/seed CCAGUGU) | 2.810 | TargetScan Human | Moderate (predicted) | TMEM245 | transmembrane protein 245 | Other | other |
| MIMAT0000263 | miR-199a-5p (and other miRNAs w/seed CCAGUGU) | 2.810 | TargetScan Human | Moderate (predicted) | TMEM255A | transmembrane protein 255A | Extracellular Space | other |
| MIMAT0000263 | miR-199a-5p (and other miRNAs w/seed CCAGUGU) | 2.810 | TargetScan Human | Moderate (predicted) | TMEM273 | transmembrane protein 273 | Other | other |
| MIMAT0000263 | miR-199a-5p (and other miRNAs w/seed CCAGUGU) | 2.810 | TargetScan Human | Moderate (predicted) | TMEM44 | transmembrane protein 44 | Other | other |
| MIMAT0000263 | miR-199a-5p (and other miRNAs w/seed CCAGUGU) | 2.810 | TargetScan Human | Moderate (predicted) | TMEM63B | transmembrane protein 63B | Plasma Membrane | ion channel |
| MIMAT0000263 | miR-199a-5p (and other miRNAs w/seed CCAGUGU) | 2.810 | TargetScan Human | Moderate (predicted) | TMEM97 | transmembrane protein 97 | Extracellular Space | other |
| MIMAT0000263 | miR-199a-5p (and other miRNAs w/seed CCAGUGU) | 2.810 | TargetScan Human | Moderate (predicted) | TMOD2 | tropomodulin 2 | Cytoplasm | other |
| MIMAT0000263 | miR-199a-5p (and other miRNAs w/seed CCAGUGU) | 2.810 | TargetScan Human | High (predicted) | TMPRSS5 | transmembrane serine protease 5 | Plasma Membrane | peptidase |
| MIMAT0000263 | miR-199a-5p (and other miRNAs w/seed CCAGUGU) | 2.810 | TargetScan Human | Moderate (predicted) | TNFRSF21 | TNF receptor superfamily member 21 | Plasma Membrane | transmembrane receptor |
| MIMAT0000263 | miR-199a-5p (and other miRNAs w/seed CCAGUGU) | 2.810 | TargetScan Human | High (predicted) | TNK1 | tyrosine kinase non receptor 1 | Cytoplasm | kinase |
| MIMAT0000263 | miR-199a-5p (and other miRNAs w/seed CCAGUGU) | 2.810 | TargetScan Human | Moderate (predicted) | TOX3 | OX high mobility group box family membe | Nucleus | transcription regulator |
| MIMAT0000263 | miR-199a-5p (and other miRNAs w/seed CCAGUGU) | 2.810 | TargetScan Human | Moderate (predicted) | TPRN | taperin | Extracellular Space | other |
| MIMAT0000263 | miR-199a-5p (and other miRNAs w/seed CCAGUGU) | 2.810 | TargetScan Human | Moderate (predicted) | TRAF3 | TNF receptor associated factor 3 | Cytoplasm | enzyme |
| MIMAT0000263 | miR-199a-5p (and other miRNAs w/seed CCAGUGU) | 2.810 | TargetScan Human | Moderate (predicted) | TREML1 | ering receptor expressed on myeloid cells | Plasma Membrane | other |
| MIMAT0000263 | miR-199a-5p (and other miRNAs w/seed CCAGUGU) | 2.810 | TargetScan Human | Moderate (predicted) | TRIM35 | tripartite motif containing 35 | Cytoplasm | other |
| MIMAT0000263 | miR-199a-5p (and other miRNAs w/seed CCAGUGU) | 2.810 | TargetScan Human | Moderate (predicted) | TRMT1L | tRNA methyltransferase 1 like | Other | enzyme |
| MIMAT0000263 | miR-199a-5p (and other miRNAs w/seed CCAGUGU) | 2.810 | TargetScan Human | Moderate (predicted) | TRMT44 | tRNA methyltransferase 44 homolog | Other | other |
| MIMAT0000263 | miR-199a-5p (and other miRNAs w/seed CCAGUGU) | 2.810 | TargetScan Human | Moderate (predicted) | TRPM4 | ceptor potential cation channel subfamily M | Plasma Membrane | ion channel |
| MIMAT0000263 | miR-199a-5p (and other miRNAs w/seed CCAGUGU) | 2.810 | TargetScan Human | Moderate (predicted) | TSPAN3 | tetraspanin 3 | Plasma Membrane | other |
| MIMAT0000263 | miR-199a-5p (and other miRNAs w/seed CCAGUGU) | 2.810 | TargetScan Human | Moderate (predicted) | TSPAN5 | tetraspanin 5 | Plasma Membrane | other |
| MIMAT0000263 | miR-199a-5p (and other miRNAs w/seed CCAGUGU) | 2.810 | TargetScan Human | High (predicted) | TSPAN6 | tetraspanin 6 | Plasma Membrane | other |
| MIMAT0000263 | miR-199a-5p (and other miRNAs w/seed CCAGUGU) | 2.810 | TargetScan Human | Moderate (predicted) | TSPYL6 | TSPY like 6 | Other | other |
| MIMAT0000263 | miR-199a-5p (and other miRNAs w/seed CCAGUGU) | 2.810 | TargetScan Human | High (predicted) | TST | thiosulfate sulfurtransferase | Cytoplasm | enzyme |
| MIMAT0000263 | miR-199a-5p (and other miRNAs w/seed CCAGUGU) | 2.810 | TargetScan Human | Moderate (predicted) | TTC9 | tetratricopeptide repeat domain 9 | Extracellular Space | other |
| MIMAT0000263 | miR-199a-5p (and other miRNAs w/seed CCAGUGU) | 2.810 | TargetScan Human | Moderate (predicted) | TTLL9 | tubulin tyrosine ligase like 9 | Cytoplasm | other |
| MIMAT0000263 | miR-199a-5p (and other miRNAs w/seed CCAGUGU) | 2.810 | TargetScan Human | Moderate (predicted) | TUBG1 | tubulin gamma 1 | Cytoplasm | other |
| MIMAT0000263 | miR-199a-5p (and other miRNAs w/seed CCAGUGU) | 2.810 | TargetScan Human | High (predicted) | TVP23C-CDRT4 | TVP23C-CDRT4 readthrough | Other | other |
| MIMAT0000263 | miR-199a-5p (and other miRNAs w/seed CCAGUGU) | 2.810 | TargetScan Human | Moderate (predicted) | TWNK | twinkle mtDNA helicase | Cytoplasm | enzyme |
| MIMAT0000263 | miR-199a-5p (and other miRNAs w/seed CCAGUGU) | 2.810 | TargetScan Human | Moderate (predicted) | TXLNB | taxilin beta | Cytoplasm | other |
| MIMAT0000263 | miR-199a-5p (and other miRNAs w/seed CCAGUGU) | 2.810 | TargetScan Human | High (predicted) | TXNDC12 | thioredoxin domain containing 12 | Cytoplasm | enzyme |
| MIMAT0000263 | miR-199a-5p (and other miRNAs w/seed CCAGUGU) | 2.810 | TargetScan Human | Moderate (predicted) | UBALD2 | UBA like domain containing 2 | Other | other |
| MIMAT0000263 | miR-199a-5p (and other miRNAs w/seed CCAGUGU) | 2.810 | TargetScan Human | Moderate (predicted) | UBAP1 | ubiquitin associated protein 1 | Cytoplasm | other |
| MIMAT0000263 | miR-199a-5p (and other miRNAs w/seed CCAGUGU) | 2.810 | TargetScan Human | High (predicted) | UBE2D4 | biquitin conjugating enzyme E2 D4 (putativ | Other | enzyme |
| MIMAT0000263 | miR-199a-5p (and other miRNAs w/seed CCAGUGU) | 2.810 | TargetScan Human | Moderate (predicted) | UBE2G1 | ubiquitin conjugating enzyme E2 G1 | Cytoplasm | enzyme |
| MIMAT0000263 | miR-199a-5p (and other miRNAs w/seed CCAGUGU) | 2.810 | TargetScan Human | Moderate (predicted) | UBE2Q1 | ubiquitin conjugating enzyme E2 Q1 | Other | enzyme |
| MIMAT0000263 | miR-199a-5p (and other miRNAs w/seed CCAGUGU) | 2.810 | TargetScan Human | High (predicted) | UBL3 | ubiquitin like 3 | Cytoplasm | other |
| MIMAT0000263 | miR-199a-5p (and other miRNAs w/seed CCAGUGU) | 2.810 | TargetScan Human | Moderate (predicted) | UBL5 | ubiquitin like 5 | Cytoplasm | other |
| MIMAT0000263 | miR-199a-5p (and other miRNAs w/seed CCAGUGU) | 2.810 | TargetScan Human | Moderate (predicted) | UHMK1 | U2AF homology motif kinase 1 | Nucleus | kinase |
| MIMAT0000263 | miR-199a-5p (and other miRNAs w/seed CCAGUGU) | 2.810 | TargetScan Human | High (predicted) | UNG | uracil DNA glycosylase | Nucleus | enzyme |
| MIMAT0000263 | miR-199a-5p (and other miRNAs w/seed CCAGUGU) | 2.810 | TargetScan Human | Moderate (predicted) | USP27X | ubiquitin specific peptidase 27 X-linked | Other | peptidase |
| MIMAT0000263 | miR-199a-5p (and other miRNAs w/seed CCAGUGU) | 2.810 | TargetScan Human | Moderate (predicted) | USP29 | ubiquitin specific peptidase 29 | Other | peptidase |
| MIMAT0000263 | miR-199a-5p (and other miRNAs w/seed CCAGUGU) | 2.810 | TargetScan Human | Moderate (predicted) | USP37 | ubiquitin specific peptidase 37 | Nucleus | peptidase |
| MIMAT0000263 | miR-199a-5p (and other miRNAs w/seed CCAGUGU) | 2.810 | TargetScan Human | Moderate (predicted) | USP44 | ubiquitin specific peptidase 44 | Nucleus | peptidase |
| MIMAT0000263 | miR-199a-5p (and other miRNAs w/seed CCAGUGU) | 2.810 | TargetScan Human | High (predicted) | USPL1 | ubiquitin specific peptidase like 1 | Cytoplasm | peptidase |
| MIMAT0000263 | miR-199a-5p (and other miRNAs w/seed CCAGUGU) | 2.810 | TargetScan Human | Moderate (predicted) | UTS2 | urotensin 2 | Extracellular Space | other |
| MIMAT0000263 | miR-199a-5p (and other miRNAs w/seed CCAGUGU) | 2.810 | TargetScan Human | Moderate (predicted) | VEGFA | vascular endothelial growth factor A | Extracellular Space | growth factor |
| MIMAT0000263 | miR-199a-5p (and other miRNAs w/seed CCAGUGU) | 2.810 | TargetScan Human | Moderate (predicted) | VGLL2 | vestigial like family member 2 | Nucleus | transcription regulator |
| MIMAT0000263 | miR-199a-5p (and other miRNAs w/seed CCAGUGU) | 2.810 | TargetScan Human | Moderate (predicted) | VMA21 | vacuolar ATPase assembly factor VMA21 | Cytoplasm | other |
| MIMAT0000263 | miR-199a-5p (and other miRNAs w/seed CCAGUGU) | 2.810 | TargetScan Human | High (predicted) | VPS26A | VPS26, retromer complex component A | Cytoplasm | transporter |
| MIMAT0000263 | miR-199a-5p (and other miRNAs w/seed CCAGUGU) | 2.810 | TargetScan Human | Moderate (predicted) | VSIG4 | set and immunoglobulin domain containin | Plasma Membrane | other |
| MIMAT0000263 | miR-199a-5p (and other miRNAs w/seed CCAGUGU) | 2.810 | TargetScan Human | Moderate (predicted) | VWC2L | Willebrand factor C domain containing 2 | Extracellular Space | other |
| MIMAT0000263 | miR-199a-5p (and other miRNAs w/seed CCAGUGU) | 2.810 | TargetScan Human | High (predicted) | WDCP | WD repeat and coiled coil containing | Other | other |
| MIMAT0000263 | miR-199a-5p (and other miRNAs w/seed CCAGUGU) | 2.810 | TargetScan Human | Moderate (predicted) | WDR31 | WD repeat domain 31 | Other | peptidase |
| MIMAT0000263 | miR-199a-5p (and other miRNAs w/seed CCAGUGU) | 2.810 | TargetScan Human | Moderate (predicted) | WDR44 | WD repeat domain 44 | Cytoplasm | other |
| MIMAT0000263 | miR-199a-5p (and other miRNAs w/seed CCAGUGU) | 2.810 | TargetScan Human | Moderate (predicted) | WDTC1 | WD and tetratricopeptide repeats 1 | Nucleus | other |
| MIMAT0000263 | miR-199a-5p (and other miRNAs w/seed CCAGUGU) | 2.810 | TargetScan Human | Moderate (predicted) | WHAMM | associated with actin, golgi membranes a | Cytoplasm | other |
| MIMAT0000263 | miR-199a-5p (and other miRNAs w/seed CCAGUGU) | 2.810 | TargetScan Human | Moderate (predicted) | WIPI2 | repeat domain, phosphoinositide interacti | Cytoplasm | other |
| MIMAT0000263 | miR-199a-5p (and other miRNAs w/seed CCAGUGU) | 2.810 | TargetScan Human | High (predicted) | WNT2 | Wnt family member 2 | Extracellular Space | cytokine |
| MIMAT0000263 | miR-199a-5p (and other miRNAs w/seed CCAGUGU) | 2.810 | TargetScan Human | Moderate (predicted) | WT1 | WT1 transcription factor | Nucleus | transcription regulator |
| MIMAT0000263 | miR-199a-5p (and other miRNAs w/seed CCAGUGU) | 2.810 | TargetScan Human | Moderate (predicted) | XAF1 | XIAP associated factor 1 | Nucleus | other |
| MIMAT0000263 | miR-199a-5p (and other miRNAs w/seed CCAGUGU) | 2.810 | TargetScan Human | Moderate (predicted) | XPOT | exportin for tRNA | Nucleus | other |
| MIMAT0000263 | miR-199a-5p (and other miRNAs w/seed CCAGUGU) | 2.810 | TargetScan Human | Moderate (predicted) | XRCC4 | X-ray repair cross complementing 4 | Nucleus | enzyme |
| MIMAT0000263 | miR-199a-5p (and other miRNAs w/seed CCAGUGU) | 2.810 | TargetScan Human | Moderate (predicted) | XYLT1 | xylosyltransferase 1 | Cytoplasm | enzyme |
| MIMAT0000263 | miR-199a-5p (and other miRNAs w/seed CCAGUGU) | 2.810 | TargetScan Human | Moderate (predicted) | ZBTB18 | zinc finger and BTB domain containing 18 | Nucleus | transcription regulator |
| MIMAT0000263 | miR-199a-5p (and other miRNAs w/seed CCAGUGU) | 2.810 | TargetScan Human | Moderate (predicted) | ZBTB37 | zinc finger and BTB domain containing 37 | Nucleus | transcription regulator |
| MIMAT0000263 | miR-199a-5p (and other miRNAs w/seed CCAGUGU) | 2.810 | TargetScan Human | High (predicted) | ZBTB42 | zinc finger and BTB domain containing 42 | Cytoplasm | transcription regulator |
| MIMAT0000263 | miR-199a-5p (and other miRNAs w/seed CCAGUGU) | 2.810 | TargetScan Human | Moderate (predicted) | ZCCHC2 | zinc finger CCHC-type containing 2 | Cytoplasm | other |
| MIMAT0000263 | miR-199a-5p (and other miRNAs w/seed CCAGUGU) | 2.810 | TargetScan Human | Moderate (predicted) | ZCCHC4 | zinc finger CCHC-type containing 4 | Nucleus | enzyme |
| MIMAT0000263 | miR-199a-5p (and other miRNAs w/seed CCAGUGU) | 2.810 | TargetScan Human | Moderate (predicted) | ZFAND2A | zinc finger AN1-type containing 2A | Other | other |
| MIMAT0000263 | miR-199a-5p (and other miRNAs w/seed CCAGUGU) | 2.810 | TargetScan Human | High (predicted) | ZFAND4 | zinc finger AN1-type containing 4 | Other | other |
| MIMAT0000263 | miR-199a-5p (and other miRNAs w/seed CCAGUGU) | 2.810 | TargetScan Human | High (predicted) | ZFP2 | ZFP2 zinc finger protein | Nucleus | transcription regulator |
| MIMAT0000263 | miR-199a-5p (and other miRNAs w/seed CCAGUGU) | 2.810 | TargetScan Human | Moderate (predicted) | ZFP90 | ZFP90 zinc finger protein | Nucleus | transcription regulator |
| MIMAT0000263 | miR-199a-5p (and other miRNAs w/seed CCAGUGU) | 2.810 | TargetScan Human | High (predicted) | ZFYVE27 | zinc finger FYVE-type containing 27 | Plasma Membrane | other |
| MIMAT0000263 | miR-199a-5p (and other miRNAs w/seed CCAGUGU) | 2.810 | TargetScan Human | High (predicted) | ZIK1 | inc finger protein interacting with K protein | Other | other |
| MIMAT0000263 | miR-199a-5p (and other miRNAs w/seed CCAGUGU) | 2.810 | TargetScan Human | Moderate (predicted) | ZKSCAN7 | inc finger with KRAB and SCAN domains | Nucleus | transcription regulator |
| MIMAT0000263 | miR-199a-5p (and other miRNAs w/seed CCAGUGU) | 2.810 | TargetScan Human | Moderate (predicted) | ZMAT2 | zinc finger matrin-type 2 | Nucleus | other |
| MIMAT0000263 | miR-199a-5p (and other miRNAs w/seed CCAGUGU) | 2.810 | TargetScan Human | High (predicted) | ZNF101 | zinc finger protein 101 | Nucleus | transcription regulator |
| MIMAT0000263 | miR-199a-5p (and other miRNAs w/seed CCAGUGU) | 2.810 | TargetScan Human | Moderate (predicted) | ZNF117 | zinc finger protein 117 | Nucleus | transcription regulator |
| MIMAT0000263 | miR-199a-5p (and other miRNAs w/seed CCAGUGU) | 2.810 | TargetScan Human | Moderate (predicted) | ZNF124 | zinc finger protein 124 | Nucleus | transcription regulator |
| MIMAT0000263 | miR-199a-5p (and other miRNAs w/seed CCAGUGU) | 2.810 | TargetScan Human | High (predicted) | ZNF132 | zinc finger protein 132 | Nucleus | transcription regulator |
| MIMAT0000263 | miR-199a-5p (and other miRNAs w/seed CCAGUGU) | 2.810 | TargetScan Human | High (predicted) | ZNF195 | zinc finger protein 195 | Nucleus | transcription regulator |
| MIMAT0000263 | miR-199a-5p (and other miRNAs w/seed CCAGUGU) | 2.810 | TargetScan Human | Moderate (predicted) | ZNF208 | zinc finger protein 208 | Nucleus | transcription regulator |
| MIMAT0000263 | miR-199a-5p (and other miRNAs w/seed CCAGUGU) | 2.810 | TargetScan Human | High (predicted) | ZNF225 | zinc finger protein 225 | Nucleus | transcription regulator |
| MIMAT0000263 | miR-199a-5p (and other miRNAs w/seed CCAGUGU) | 2.810 | TargetScan Human | High (predicted) | ZNF23 | zinc finger protein 23 | Nucleus | transcription regulator |
| MIMAT0000263 | miR-199a-5p (and other miRNAs w/seed CCAGUGU) | 2.810 | TargetScan Human | High (predicted) | ZNF233 | zinc finger protein 233 | Nucleus | transcription regulator |
| MIMAT0000263 | miR-199a-5p (and other miRNAs w/seed CCAGUGU) | 2.810 | TargetScan Human | Moderate (predicted) | ZNF24 | zinc finger protein 24 | Nucleus | transcription regulator |
| MIMAT0000263 | miR-199a-5p (and other miRNAs w/seed CCAGUGU) | 2.810 | TargetScan Human | High (predicted) | ZNF256 | zinc finger protein 256 | Nucleus | transcription regulator |
| MIMAT0000263 | miR-199a-5p (and other miRNAs w/seed CCAGUGU) | 2.810 | TargetScan Human | High (predicted) | ZNF266 | zinc finger protein 266 | Nucleus | transcription regulator |
| MIMAT0000263 | miR-199a-5p (and other miRNAs w/seed CCAGUGU) | 2.810 | TargetScan Human | High (predicted) | ZNF28 | zinc finger protein 28 | Nucleus | transcription regulator |
| MIMAT0000263 | miR-199a-5p (and other miRNAs w/seed CCAGUGU) | 2.810 | TargetScan Human | High (predicted) | ZNF284 | zinc finger protein 284 | Other | other |
| MIMAT0000263 | miR-199a-5p (and other miRNAs w/seed CCAGUGU) | 2.810 | TargetScan Human | Moderate (predicted) | ZNF285 | zinc finger protein 285 | Nucleus | transcription regulator |
| MIMAT0000263 | miR-199a-5p (and other miRNAs w/seed CCAGUGU) | 2.810 | TargetScan Human | Moderate (predicted) | ZNF286A | zinc finger protein 286A | Nucleus | transcription regulator |
| MIMAT0000263 | miR-199a-5p (and other miRNAs w/seed CCAGUGU) | 2.810 | TargetScan Human | High (predicted) | ZNF30 | zinc finger protein 30 | Nucleus | transcription regulator |
| MIMAT0000263 | miR-199a-5p (and other miRNAs w/seed CCAGUGU) | 2.810 | TargetScan Human | Moderate (predicted) | ZNF300 | zinc finger protein 300 | Nucleus | transcription regulator |
| MIMAT0000263 | miR-199a-5p (and other miRNAs w/seed CCAGUGU) | 2.810 | TargetScan Human | High (predicted) | ZNF329 | zinc finger protein 329 | Other | other |
| MIMAT0000263 | miR-199a-5p (and other miRNAs w/seed CCAGUGU) | 2.810 | TargetScan Human | Moderate (predicted) | ZNF347 | zinc finger protein 347 | Nucleus | transcription regulator |

Sup. Table 3. List of 3699 targets of the seven dysregulated miRNAs obtained from three different target prediction databases,TargetScan TarBase and miRecords considering those predicted as moderate, high, or experimentally validated, respectively.

| | | | | | | | | |
|---|---|---|---|---|---|---|---|---|
| MIMAT0000263 | miR-199a-5p (and other miRNAs w/seed CCAGUGU) | 2.810 | TargetScan Human | Moderate (predicted) | ZNF37A | zinc finger protein 37A | Nucleus | transcription regulator |
| MIMAT0000263 | miR-199a-5p (and other miRNAs w/seed CCAGUGU) | 2.810 | TargetScan Human | Moderate (predicted) | ZNF41 | zinc finger protein 41 | Nucleus | transcription regulator |
| MIMAT0000263 | miR-199a-5p (and other miRNAs w/seed CCAGUGU) | 2.810 | TargetScan Human | Moderate (predicted) | ZNF417/ZNF587 | zinc finger protein 417 | Nucleus | transcription regulator |
| MIMAT0000263 | miR-199a-5p (and other miRNAs w/seed CCAGUGU) | 2.810 | TargetScan Human | High (predicted) | ZNF418 | zinc finger protein 418 | Nucleus | transcription regulator |
| MIMAT0000263 | miR-199a-5p (and other miRNAs w/seed CCAGUGU) | 2.810 | TargetScan Human | High (predicted) | ZNF419 | zinc finger protein 419 | Nucleus | transcription regulator |
| MIMAT0000263 | miR-199a-5p (and other miRNAs w/seed CCAGUGU) | 2.810 | TargetScan Human | Moderate (predicted) | ZNF425 | zinc finger protein 425 | Nucleus | other |
| MIMAT0000263 | miR-199a-5p (and other miRNAs w/seed CCAGUGU) | 2.810 | TargetScan Human | High (predicted) | ZNF426 | zinc finger protein 426 | Nucleus | transcription regulator |
| MIMAT0000263 | miR-199a-5p (and other miRNAs w/seed CCAGUGU) | 2.810 | TargetScan Human | High (predicted) | ZNF439 | zinc finger protein 439 | Nucleus | transcription regulator |
| MIMAT0000263 | miR-199a-5p (and other miRNAs w/seed CCAGUGU) | 2.810 | TargetScan Human | High (predicted) | ZNF440 | zinc finger protein 440 | Nucleus | transcription regulator |
| MIMAT0000263 | miR-199a-5p (and other miRNAs w/seed CCAGUGU) | 2.810 | TargetScan Human | High (predicted) | ZNF442 | zinc finger protein 442 | Nucleus | transcription regulator |
| MIMAT0000263 | miR-199a-5p (and other miRNAs w/seed CCAGUGU) | 2.810 | TargetScan Human | High (predicted) | ZNF468 | zinc finger protein 468 | Other | other |
| MIMAT0000263 | miR-199a-5p (and other miRNAs w/seed CCAGUGU) | 2.810 | TargetScan Human | High (predicted) | ZNF470 | zinc finger protein 470 | Nucleus | other |
| MIMAT0000263 | miR-199a-5p (and other miRNAs w/seed CCAGUGU) | 2.810 | TargetScan Human | Moderate (predicted) | ZNF473 | zinc finger protein 473 | Nucleus | other |
| MIMAT0000263 | miR-199a-5p (and other miRNAs w/seed CCAGUGU) | 2.810 | TargetScan Human | High (predicted) | ZNF479 | zinc finger protein 479 | Nucleus | transcription regulator |
| MIMAT0000263 | miR-199a-5p (and other miRNAs w/seed CCAGUGU) | 2.810 | TargetScan Human | Moderate (predicted) | ZNF488 | zinc finger protein 488 | Nucleus | transcription regulator |
| MIMAT0000263 | miR-199a-5p (and other miRNAs w/seed CCAGUGU) | 2.810 | TargetScan Human | High (predicted) | ZNF490 | zinc finger protein 490 | Nucleus | transcription regulator |
| MIMAT0000263 | miR-199a-5p (and other miRNAs w/seed CCAGUGU) | 2.810 | TargetScan Human | (predicted),Moderate (pred | ZNF492/ZNF98 | zinc finger protein 492 | Nucleus | transcription regulator |
| MIMAT0000263 | miR-199a-5p (and other miRNAs w/seed CCAGUGU) | 2.810 | TargetScan Human | High (predicted) | ZNF514 | zinc finger protein 514 | Nucleus | transcription regulator |
| MIMAT0000263 | miR-199a-5p (and other miRNAs w/seed CCAGUGU) | 2.810 | TargetScan Human | Moderate (predicted) | ZNF516 | zinc finger protein 516 | Nucleus | transcription regulator |
| MIMAT0000263 | miR-199a-5p (and other miRNAs w/seed CCAGUGU) | 2.810 | TargetScan Human | Moderate (predicted) | ZNF525 | zinc finger protein 525 | Nucleus | transcription regulator |
| MIMAT0000263 | miR-199a-5p (and other miRNAs w/seed CCAGUGU) | 2.810 | TargetScan Human | High (predicted) | ZNF528 | zinc finger protein 528 | Other | other |
| MIMAT0000263 | miR-199a-5p (and other miRNAs w/seed CCAGUGU) | 2.810 | TargetScan Human | High (predicted) | ZNF544 | zinc finger protein 544 | Nucleus | transcription regulator |
| MIMAT0000263 | miR-199a-5p (and other miRNAs w/seed CCAGUGU) | 2.810 | TargetScan Human | High (predicted) | ZNF547 | zinc finger protein 547 | Nucleus | transcription regulator |
| MIMAT0000263 | miR-199a-5p (and other miRNAs w/seed CCAGUGU) | 2.810 | TargetScan Human | High (predicted) | ZNF551 | zinc finger protein 551 | Nucleus | transcription regulator |
| MIMAT0000263 | miR-199a-5p (and other miRNAs w/seed CCAGUGU) | 2.810 | TargetScan Human | Moderate (predicted) | ZNF555 | zinc finger protein 555 | Other | other |
| MIMAT0000263 | miR-199a-5p (and other miRNAs w/seed CCAGUGU) | 2.810 | TargetScan Human | High (predicted) | ZNF558 | zinc finger protein 558 | Nucleus | transcription regulator |
| MIMAT0000263 | miR-199a-5p (and other miRNAs w/seed CCAGUGU) | 2.810 | TargetScan Human | Moderate (predicted) | ZNF560 | zinc finger protein 560 | Nucleus | transcription regulator |
| MIMAT0000263 | miR-199a-5p (and other miRNAs w/seed CCAGUGU) | 2.810 | TargetScan Human | High (predicted) | ZNF561 | zinc finger protein 561 | Nucleus | transcription regulator |
| MIMAT0000263 | miR-199a-5p (and other miRNAs w/seed CCAGUGU) | 2.810 | TargetScan Human | Moderate (predicted) | ZNF562 | zinc finger protein 562 | Nucleus | transcription regulator |
| MIMAT0000263 | miR-199a-5p (and other miRNAs w/seed CCAGUGU) | 2.810 | TargetScan Human | High (predicted) | ZNF563 | zinc finger protein 563 | Nucleus | transcription regulator |
| MIMAT0000263 | miR-199a-5p (and other miRNAs w/seed CCAGUGU) | 2.810 | TargetScan Human | High (predicted) | ZNF579 | zinc finger protein 579 | Nucleus | transcription regulator |
| MIMAT0000263 | miR-199a-5p (and other miRNAs w/seed CCAGUGU) | 2.810 | TargetScan Human | High (predicted) | ZNF582 | zinc finger protein 582 | Other | other |
| MIMAT0000263 | miR-199a-5p (and other miRNAs w/seed CCAGUGU) | 2.810 | TargetScan Human | High (predicted) | ZNF584 | zinc finger protein 584 | Nucleus | transcription regulator |
| MIMAT0000263 | miR-199a-5p (and other miRNAs w/seed CCAGUGU) | 2.810 | TargetScan Human | High (predicted) | ZNF586 | zinc finger protein 586 | Other | other |
| MIMAT0000263 | miR-199a-5p (and other miRNAs w/seed CCAGUGU) | 2.810 | TargetScan Human | Moderate (predicted) | ZNF594 | zinc finger protein 594 | Nucleus | transcription regulator |
| MIMAT0000263 | miR-199a-5p (and other miRNAs w/seed CCAGUGU) | 2.810 | TargetScan Human | High (predicted) | ZNF615 | zinc finger protein 615 | Nucleus | transcription regulator |
| MIMAT0000263 | miR-199a-5p (and other miRNAs w/seed CCAGUGU) | 2.810 | TargetScan Human | High (predicted) | ZNF616 | zinc finger protein 616 | Nucleus | transcription regulator |
| MIMAT0000263 | miR-199a-5p (and other miRNAs w/seed CCAGUGU) | 2.810 | TargetScan Human | Moderate (predicted) | ZNF618 | zinc finger protein 618 | Nucleus | other |
| MIMAT0000263 | miR-199a-5p (and other miRNAs w/seed CCAGUGU) | 2.810 | TargetScan Human | Moderate (predicted) | ZNF619 | zinc finger protein 619 | Other | other |
| MIMAT0000263 | miR-199a-5p (and other miRNAs w/seed CCAGUGU) | 2.810 | TargetScan Human | High (predicted) | ZNF625 | zinc finger protein 625 | Other | other |
| MIMAT0000263 | miR-199a-5p (and other miRNAs w/seed CCAGUGU) | 2.810 | TargetScan Human | High (predicted) | ZNF627 | zinc finger protein 627 | Other | other |
| MIMAT0000263 | miR-199a-5p (and other miRNAs w/seed CCAGUGU) | 2.810 | TargetScan Human | Moderate (predicted) | ZNF629 | zinc finger protein 629 | Nucleus | transcription regulator |
| MIMAT0000263 | miR-199a-5p (and other miRNAs w/seed CCAGUGU) | 2.810 | TargetScan Human | Moderate (predicted) | ZNF641 | zinc finger protein 641 | Other | other |
| MIMAT0000263 | miR-199a-5p (and other miRNAs w/seed CCAGUGU) | 2.810 | TargetScan Human | Moderate (predicted) | ZNF652 | zinc finger protein 652 | Other | other |
| MIMAT0000263 | miR-199a-5p (and other miRNAs w/seed CCAGUGU) | 2.810 | TargetScan Human | Moderate (predicted) | ZNF654 | zinc finger protein 654 | Other | other |
| MIMAT0000263 | miR-199a-5p (and other miRNAs w/seed CCAGUGU) | 2.810 | TargetScan Human | Moderate (predicted) | ZNF667 | zinc finger protein 667 | Nucleus | transcription regulator |
| MIMAT0000263 | miR-199a-5p (and other miRNAs w/seed CCAGUGU) | 2.810 | TargetScan Human | High (predicted) | ZNF669 | zinc finger protein 669 | Other | other |
| MIMAT0000263 | miR-199a-5p (and other miRNAs w/seed CCAGUGU) | 2.810 | TargetScan Human | High (predicted) | ZNF700 | zinc finger protein 700 | Nucleus | transcription regulator |
| MIMAT0000263 | miR-199a-5p (and other miRNAs w/seed CCAGUGU) | 2.810 | TargetScan Human | High (predicted) | ZNF701 | zinc finger protein 701 | Nucleus | transcription regulator |
| MIMAT0000263 | miR-199a-5p (and other miRNAs w/seed CCAGUGU) | 2.810 | TargetScan Human | High (predicted) | ZNF705A | zinc finger protein 705A | Nucleus | transcription regulator |
| MIMAT0000263 | miR-199a-5p (and other miRNAs w/seed CCAGUGU) | 2.810 | TargetScan Human | High (predicted) | ZNF705B/ZNF705D | zinc finger protein 705B | Other | other |
| MIMAT0000263 | miR-199a-5p (and other miRNAs w/seed CCAGUGU) | 2.810 | TargetScan Human | High (predicted) | ZNF705G | zinc finger protein 705G | Other | other |
| MIMAT0000263 | miR-199a-5p (and other miRNAs w/seed CCAGUGU) | 2.810 | TargetScan Human | High (predicted) | ZNF709 | zinc finger protein 709 | Other | other |
| MIMAT0000263 | miR-199a-5p (and other miRNAs w/seed CCAGUGU) | 2.810 | TargetScan Human | High (predicted) | ZNF772 | zinc finger protein 772 | Other | other |
| MIMAT0000263 | miR-199a-5p (and other miRNAs w/seed CCAGUGU) | 2.810 | TargetScan Human | High (predicted) | ZNF773 | zinc finger protein 773 | Other | other |
| MIMAT0000263 | miR-199a-5p (and other miRNAs w/seed CCAGUGU) | 2.810 | TargetScan Human | High (predicted) | ZNF776 | zinc finger protein 776 | Other | other |
| MIMAT0000263 | miR-199a-5p (and other miRNAs w/seed CCAGUGU) | 2.810 | TargetScan Human | Moderate (predicted) | ZNF778 | zinc finger protein 778 | Nucleus | transcription regulator |
| MIMAT0000263 | miR-199a-5p (and other miRNAs w/seed CCAGUGU) | 2.810 | TargetScan Human | Moderate (predicted) | ZNF780A | zinc finger protein 780A | Other | other |
| MIMAT0000263 | miR-199a-5p (and other miRNAs w/seed CCAGUGU) | 2.810 | TargetScan Human | Moderate (predicted) | ZNF781 | zinc finger protein 781 | Other | other |
| MIMAT0000263 | miR-199a-5p (and other miRNAs w/seed CCAGUGU) | 2.810 | TargetScan Human | Moderate (predicted) | ZNF784 | zinc finger protein 784 | Other | other |
| MIMAT0000263 | miR-199a-5p (and other miRNAs w/seed CCAGUGU) | 2.810 | TargetScan Human | High (predicted) | ZNF791 | zinc finger protein 791 | Other | other |
| MIMAT0000263 | miR-199a-5p (and other miRNAs w/seed CCAGUGU) | 2.810 | TargetScan Human | High (predicted) | ZNF81 | zinc finger protein 81 | Nucleus | transcription regulator |
| MIMAT0000263 | miR-199a-5p (and other miRNAs w/seed CCAGUGU) | 2.810 | TargetScan Human | Moderate (predicted) | ZNF813 | zinc finger protein 813 | Other | other |
| MIMAT0000263 | miR-199a-5p (and other miRNAs w/seed CCAGUGU) | 2.810 | TargetScan Human | High (predicted) | ZNF823 | zinc finger protein 823 | Nucleus | transcription regulator |
| MIMAT0000263 | miR-199a-5p (and other miRNAs w/seed CCAGUGU) | 2.810 | TargetScan Human | High (predicted) | ZNF83 | zinc finger protein 83 | Nucleus | transcription regulator |
| MIMAT0000263 | miR-199a-5p (and other miRNAs w/seed CCAGUGU) | 2.810 | TargetScan Human | Moderate (predicted) | ZNF84 | zinc finger protein 84 | Nucleus | transcription regulator |
| MIMAT0000263 | miR-199a-5p (and other miRNAs w/seed CCAGUGU) | 2.810 | TargetScan Human | High (predicted) | ZNF844 | zinc finger protein 844 | Other | other |
| MIMAT0000263 | miR-199a-5p (and other miRNAs w/seed CCAGUGU) | 2.810 | TargetScan Human | Moderate (predicted) | ZNF845 | zinc finger protein 845 | Other | other |
| MIMAT0000263 | miR-199a-5p (and other miRNAs w/seed CCAGUGU) | 2.810 | TargetScan Human | High (predicted) | ZNF846 | zinc finger protein 846 | Other | other |
| MIMAT0000263 | miR-199a-5p (and other miRNAs w/seed CCAGUGU) | 2.810 | TargetScan Human | Moderate (predicted) | ZNF852 | zinc finger protein 852 | Other | other |
| MIMAT0000263 | miR-199a-5p (and other miRNAs w/seed CCAGUGU) | 2.810 | TargetScan Human | High (predicted) | ZNF878 | zinc finger protein 878 | Other | other |
| MIMAT0000263 | miR-199a-5p (and other miRNAs w/seed CCAGUGU) | 2.810 | TargetScan Human | Moderate (predicted) | ZSCAN29 | zinc finger and SCAN domain containing 2 | Nucleus | transcription regulator |
| MIMAT0004560 | miR-183-3p (miRNAs w/seed UGAAUUA) | 2.300 | TargetScan Human | Moderate (predicted) | ABCB4 | ATP binding cassette subfamily B member | Plasma Membrane | transporter |
| MIMAT0004560 | miR-183-3p (miRNAs w/seed UGAAUUA) | 2.300 | TargetScan Human | Moderate (predicted) | ABHD10 | ydrolase domain containing 10, depalmito | Cytoplasm | enzyme |
| MIMAT0004560 | miR-183-3p (miRNAs w/seed UGAAUUA) | 2.300 | TargetScan Human | Moderate (predicted) | AKIRIN1 | akirin 1 | Cytoplasm | enzyme |
| MIMAT0004560 | miR-183-3p (miRNAs w/seed UGAAUUA) | 2.300 | TargetScan Human | High (predicted) | AMTN | amelotin | Extracellular Space | other |
| MIMAT0004560 | miR-183-3p (miRNAs w/seed UGAAUUA) | 2.300 | TargetScan Human | Moderate (predicted) | AP1S3 | ptor related protein complex 1 subunit sigr | Cytoplasm | transporter |
| MIMAT0004560 | miR-183-3p (miRNAs w/seed UGAAUUA) | 2.300 | TargetScan Human | Moderate (predicted) | ARMC9 | armadillo repeat containing 9 | Cytoplasm | other |
| MIMAT0004560 | miR-183-3p (miRNAs w/seed UGAAUUA) | 2.300 | TargetScan Human | Moderate (predicted) | ART3 | ADP-ribosyltransferase 3 (inactive) | Plasma Membrane | enzyme |
| MIMAT0004560 | miR-183-3p (miRNAs w/seed UGAAUUA) | 2.300 | TargetScan Human | High (predicted) | ATP5MK | ATP synthase membrane subunit k | Cytoplasm | other |
| MIMAT0004560 | miR-183-3p (miRNAs w/seed UGAAUUA) | 2.300 | TargetScan Human | Moderate (predicted) | B3GALT2 | beta-1,3-galactosyltransferase 2 | Cytoplasm | enzyme |
| MIMAT0004560 | miR-183-3p (miRNAs w/seed UGAAUUA) | 2.300 | TargetScan Human | Moderate (predicted) | B4GALT6 | beta-1,4-galactosyltransferase 6 | Cytoplasm | enzyme |
| MIMAT0004560 | miR-183-3p (miRNAs w/seed UGAAUUA) | 2.300 | TargetScan Human | Moderate (predicted) | BMP3 | bone morphogenetic protein 3 | Extracellular Space | growth factor |
| MIMAT0004560 | miR-183-3p (miRNAs w/seed UGAAUUA) | 2.300 | TargetScan Human | Moderate (predicted) | C2orf73 | chromosome 2 open reading frame 73 | Other | other |
| MIMAT0004560 | miR-183-3p (miRNAs w/seed UGAAUUA) | 2.300 | TargetScan Human | Moderate (predicted) | CA1 | carbonic anhydrase 1 | Cytoplasm | enzyme |
| MIMAT0004560 | miR-183-3p (miRNAs w/seed UGAAUUA) | 2.300 | TargetScan Human | Moderate (predicted) | CAMK2N1 | /calmodulin dependent protein kinase II in | Plasma Membrane | kinase |
| MIMAT0004560 | miR-183-3p (miRNAs w/seed UGAAUUA) | 2.300 | TargetScan Human | Moderate (predicted) | CCDC91 | coiled-coil domain containing 91 | Cytoplasm | other |
| MIMAT0004560 | miR-183-3p (miRNAs w/seed UGAAUUA) | 2.300 | TargetScan Human | Moderate (predicted) | CCNI | cyclin I | Other | other |
| MIMAT0004560 | miR-183-3p (miRNAs w/seed UGAAUUA) | 2.300 | TargetScan Human | Moderate (predicted) | CDX4 | caudal type homeobox 4 | Nucleus | transcription regulator |
| MIMAT0004560 | miR-183-3p (miRNAs w/seed UGAAUUA) | 2.300 | TargetScan Human | Moderate (predicted) | CLDN22 | claudin 22 | Plasma Membrane | other |
| MIMAT0004560 | miR-183-3p (miRNAs w/seed UGAAUUA) | 2.300 | TargetScan Human | Moderate (predicted) | CLU | clusterin | Cytoplasm | other |
| MIMAT0004560 | miR-183-3p (miRNAs w/seed UGAAUUA) | 2.300 | TargetScan Human | Moderate (predicted) | CLVS1 | clavesin 1 | Cytoplasm | other |
| MIMAT0004560 | miR-183-3p (miRNAs w/seed UGAAUUA) | 2.300 | TargetScan Human | Moderate (predicted) | COMMD2 | COMM domain containing 2 | Other | other |
| MIMAT0004560 | miR-183-3p (miRNAs w/seed UGAAUUA) | 2.300 | TargetScan Human | Moderate (predicted) | COQ10B | coenzyme Q10B | Cytoplasm | other |
| MIMAT0004560 | miR-183-3p (miRNAs w/seed UGAAUUA) | 2.300 | TargetScan Human | Moderate (predicted) | CSN2 | casein beta | Extracellular Space | kinase |
| MIMAT0004560 | miR-183-3p (miRNAs w/seed UGAAUUA) | 2.300 | TargetScan Human | Moderate (predicted) | CTSC | cathepsin C | Cytoplasm | peptidase |
| MIMAT0004560 | miR-183-3p (miRNAs w/seed UGAAUUA) | 2.300 | TargetScan Human | Moderate (predicted) | CTXN3 | cortexin 3 | Other | other |
| MIMAT0004560 | miR-183-3p (miRNAs w/seed UGAAUUA) | 2.300 | TargetScan Human | Moderate (predicted) | CYLC2 | cylicin 2 | Other | other |
| MIMAT0004560 | miR-183-3p (miRNAs w/seed UGAAUUA) | 2.300 | TargetScan Human | Moderate (predicted) | CYP2C19 | chrome P450 family 2 subfamily C membe | Cytoplasm | enzyme |
| MIMAT0004560 | miR-183-3p (miRNAs w/seed UGAAUUA) | 2.300 | TargetScan Human | Moderate (predicted) | CYP2C9 | ochrome P450 family 2 subfamily C memb | Cytoplasm | enzyme |
| MIMAT0004560 | miR-183-3p (miRNAs w/seed UGAAUUA) | 2.300 | TargetScan Human | Moderate (predicted) | DAOA | D-amino acid oxidase activator | Cytoplasm | other |
| MIMAT0004560 | miR-183-3p (miRNAs w/seed UGAAUUA) | 2.300 | TargetScan Human | Moderate (predicted) | DAZAP1 | DAZ associated protein 1 | Other | other |
| MIMAT0004560 | miR-183-3p (miRNAs w/seed UGAAUUA) | 2.300 | TargetScan Human | Moderate (predicted) | DIS3 | g, exosome endoribonuclease and 3'-5' ex | Nucleus | enzyme |
| MIMAT0004560 | miR-183-3p (miRNAs w/seed UGAAUUA) | 2.300 | TargetScan Human | Moderate (predicted) | EFCAB10 | EF-hand calcium binding domain 10 | Other | other |
| MIMAT0004560 | miR-183-3p (miRNAs w/seed UGAAUUA) | 2.300 | TargetScan Human | Moderate (predicted) | EIF5A | eukaryotic translation initiation factor 5A | Cytoplasm | translation regulator |
| MIMAT0004560 | miR-183-3p (miRNAs w/seed UGAAUUA) | 2.300 | TargetScan Human | Moderate (predicted) | EXOSC5 | exosome component 5 | Nucleus | enzyme |
| MIMAT0004560 | miR-183-3p (miRNAs w/seed UGAAUUA) | 2.300 | TargetScan Human | Moderate (predicted) | GLT8D1 | glycosyltransferase 8 domain containing 1 | Cytoplasm | enzyme |
| MIMAT0004560 | miR-183-3p (miRNAs w/seed UGAAUUA) | 2.300 | TargetScan Human | Moderate (predicted) | GLYATL2 | glycine-N-acyltransferase like 2 | Cytoplasm | enzyme |
| MIMAT0004560 | miR-183-3p (miRNAs w/seed UGAAUUA) | 2.300 | TargetScan Human | Moderate (predicted) | GOLM2 | golgi membrane protein 2 | Cytoplasm | other |
| MIMAT0004560 | miR-183-3p (miRNAs w/seed UGAAUUA) | 2.300 | TargetScan Human | Moderate (predicted) | GSX2 | GS homeobox 2 | Nucleus | transcription regulator |
| MIMAT0004560 | miR-183-3p (miRNAs w/seed UGAAUUA) | 2.300 | TargetScan Human | Moderate (predicted) | GTF2A1L | general transcription factor IIA subunit 1 lik | Nucleus | transcription regulator |
| MIMAT0004560 | miR-183-3p (miRNAs w/seed UGAAUUA) | 2.300 | TargetScan Human | Moderate (predicted) | GZMK | granzyme K | Cytoplasm | peptidase |
| MIMAT0004560 | miR-183-3p (miRNAs w/seed UGAAUUA) | 2.300 | TargetScan Human | Moderate (predicted) | HENMT1 | HEN methyltransferase 1 | Cytoplasm | enzyme |
| MIMAT0004560 | miR-183-3p (miRNAs w/seed UGAAUUA) | 2.300 | TargetScan Human | Moderate (predicted) | IFT43 | intraflagellar transport 43 | Cytoplasm | other |
| MIMAT0004560 | miR-183-3p (miRNAs w/seed UGAAUUA) | 2.300 | TargetScan Human | Moderate (predicted) | IL1R2 | interleukin 1 receptor type 2 | Plasma Membrane | transmembrane receptor |
| MIMAT0004560 | miR-183-3p (miRNAs w/seed UGAAUUA) | 2.300 | TargetScan Human | Moderate (predicted) | INTS13 | integrator complex subunit 13 | Cytoplasm | other |
| MIMAT0004560 | miR-183-3p (miRNAs w/seed UGAAUUA) | 2.300 | TargetScan Human | Moderate (predicted) | IRX5 | iroquois homeobox 5 | Nucleus | transcription regulator |
| MIMAT0004560 | miR-183-3p (miRNAs w/seed UGAAUUA) | 2.300 | TargetScan Human | Moderate (predicted) | ITM2B | integral membrane protein 2B | Plasma Membrane | other |
| MIMAT0004560 | miR-183-3p (miRNAs w/seed UGAAUUA) | 2.300 | TargetScan Human | Moderate (predicted) | KCNE2 | oltage-gated channel subfamily E regulato | Plasma Membrane | ion channel |
| MIMAT0004560 | miR-183-3p (miRNAs w/seed UGAAUUA) | 2.300 | TargetScan Human | Moderate (predicted) | KIF20B | kinesin family member 20B | Nucleus | enzyme |
| MIMAT0004560 | miR-183-3p (miRNAs w/seed UGAAUUA) | 2.300 | TargetScan Human | Moderate (predicted) | KRTAP4-3 | keratin associated protein 4-3 | Cytoplasm | other |
| MIMAT0004560 | miR-183-3p (miRNAs w/seed UGAAUUA) | 2.300 | TargetScan Human | Moderate (predicted) | LAPTM5 | lysosomal protein transmembrane 5 | Plasma Membrane | other |
| MIMAT0004560 | miR-183-3p (miRNAs w/seed UGAAUUA) | 2.300 | TargetScan Human | Moderate (predicted) | LINC01546 | ng intergenic non-protein coding RNA 154 | Other | other |
| MIMAT0004560 | miR-183-3p (miRNAs w/seed UGAAUUA) | 2.300 | TargetScan Human | Moderate (predicted) | LOC100129603 | uncharacterized LOC100129603 | Other | other |
| MIMAT0004560 | miR-183-3p (miRNAs w/seed UGAAUUA) | 2.300 | TargetScan Human | Moderate (predicted) | LPL | lipoprotein lipase | Cytoplasm | enzyme |
| MIMAT0004560 | miR-183-3p (miRNAs w/seed UGAAUUA) | 2.300 | TargetScan Human | Moderate (predicted) | LRRC39 | leucine rich repeat containing 39 | Other | other |
| MIMAT0004560 | miR-183-3p (miRNAs w/seed UGAAUUA) | 2.300 | TargetScan Human | Moderate (predicted) | LSMEM1 | eucine rich single-pass membrane protein | Cytoplasm | other |
| MIMAT0004560 | miR-183-3p (miRNAs w/seed UGAAUUA) | 2.300 | TargetScan Human | Moderate (predicted) | MANSC1 | MANSC domain containing 1 | Other | other |
| MIMAT0004560 | miR-183-3p (miRNAs w/seed UGAAUUA) | 2.300 | TargetScan Human | Moderate (predicted) | MBIP | MAP3K12 binding inhibitory protein 1 | Nucleus | other |
| MIMAT0004560 | miR-183-3p (miRNAs w/seed UGAAUUA) | 2.300 | TargetScan Human | Moderate (predicted) | MSH4 | mutS homolog 4 | Nucleus | enzyme |
| MIMAT0004560 | miR-183-3p (miRNAs w/seed UGAAUUA) | 2.300 | TargetScan Human | Moderate (predicted) | MT-ATP6 | ATP synthase F0 subunit 6 | Cytoplasm | transporter |
| MIMAT0004560 | miR-183-3p (miRNAs w/seed UGAAUUA) | 2.300 | TargetScan Human | Moderate (predicted) | MTRNR2L10 | MT-RNR2 like 10 | Other | other |
| MIMAT0004560 | miR-183-3p (miRNAs w/seed UGAAUUA) | 2.300 | TargetScan Human | Moderate (predicted) | MTRNR2L11 | MT-RNR2 like 11 | Other | other |
| MIMAT0004560 | miR-183-3p (miRNAs w/seed UGAAUUA) | 2.300 | TargetScan Human | High (predicted) | MTRNR2L13 | MT-RNR2 like 13 | Other | other |
| MIMAT0004560 | miR-183-3p (miRNAs w/seed UGAAUUA) | 2.300 | TargetScan Human | High (predicted) | MTRNR2L3 | MT-RNR2 like 3 | Other | other |
| MIMAT0004560 | miR-183-3p (miRNAs w/seed UGAAUUA) | 2.300 | TargetScan Human | High (predicted) | MTRNR2L4 | MT-RNR2 like 4 | Other | other |
| MIMAT0004560 | miR-183-3p (miRNAs w/seed UGAAUUA) | 2.300 | TargetScan Human | Moderate (predicted) | MTRNR2L5 | MT-RNR2 like 5 | Other | other |
| MIMAT0004560 | miR-183-3p (miRNAs w/seed UGAAUUA) | 2.300 | TargetScan Human | High (predicted) | MTRNR2L7 | MT-RNR2 like 7 | Other | other |
| MIMAT0004560 | miR-183-3p (miRNAs w/seed UGAAUUA) | 2.300 | TargetScan Human | Moderate (predicted) | MTRNR2L8 | MT-RNR2 like 8 | Other | other |
| MIMAT0004560 | miR-183-3p (miRNAs w/seed UGAAUUA) | 2.300 | TargetScan Human | High (predicted) | MUC15 | mucin 15, cell surface associated | Extracellular Space | other |
| MIMAT0004560 | miR-183-3p (miRNAs w/seed UGAAUUA) | 2.300 | TargetScan Human | High (predicted) | NBEAL1 | neurobeachin like 1 | Other | other |
| MIMAT0004560 | miR-183-3p (miRNAs w/seed UGAAUUA) | 2.300 | TargetScan Human | High (predicted) | NDUFA12 | ADH:ubiquinone oxidoreductase subunit A | Cytoplasm | enzyme |
| MIMAT0004560 | miR-183-3p (miRNAs w/seed UGAAUUA) | 2.300 | TargetScan Human | Moderate (predicted) | NEUROD1 | neuronal differentiation 1 | Nucleus | transcription regulator |
| MIMAT0004560 | miR-183-3p (miRNAs w/seed UGAAUUA) | 2.300 | TargetScan Human | High (predicted) | NOX4 | NADPH oxidase 4 | Cytoplasm | enzyme |
| MIMAT0004560 | miR-183-3p (miRNAs w/seed UGAAUUA) | 2.300 | TargetScan Human | Moderate (predicted) | NXPH1 | neurexophilin 1 | Extracellular Space | other |
| MIMAT0004560 | miR-183-3p (miRNAs w/seed UGAAUUA) | 2.300 | TargetScan Human | Moderate (predicted) | NXPH2 | neurexophilin 2 | Extracellular Space | other |

Sup. Table 3. List of 3699 targets of the seven dysregulated miRNAs obtained from three different target prediction databases,TargetScan TarBase and miRecords considering those predicted as moderate, high, or experimentally validated, respectively.

| | | | | | | | | |
|---|---|---|---|---|---|---|---|---|
| MIMAT0004560 | miR-183-3p (miRNAs w/seed UGAAUUA) | 2.300 | TargetScan Human | Moderate (predicted) | OOSP2 | oocyte secreted protein 2 | Other | other |
| MIMAT0004560 | miR-183-3p (miRNAs w/seed UGAAUUA) | 2.300 | TargetScan Human | Moderate (predicted) | OR2AE1 | ctory receptor family 2 subfamily AE memb | Plasma Membrane | other |
| MIMAT0004560 | miR-183-3p (miRNAs w/seed UGAAUUA) | 2.300 | TargetScan Human | Moderate (predicted) | OR2L13 | ctory receptor family 2 subfamily L membe | Plasma Membrane | G-protein coupled receptor |
| MIMAT0004560 | miR-183-3p (miRNAs w/seed UGAAUUA) | 2.300 | TargetScan Human | Moderate (predicted) | PAPPA-AS1 | PAPPA antisense RNA 1 | Other | other |
| MIMAT0004560 | miR-183-3p (miRNAs w/seed UGAAUUA) | 2.300 | TargetScan Human | Moderate (predicted) | PCP4 | Purkinje cell protein 4 | Cytoplasm | other |
| MIMAT0004560 | miR-183-3p (miRNAs w/seed UGAAUUA) | 2.300 | TargetScan Human | Moderate (predicted) | PKIB | AMP-dependent protein kinase inhibitor be | Other | other |
| MIMAT0004560 | miR-183-3p (miRNAs w/seed UGAAUUA) | 2.300 | TargetScan Human | (predicted),Moderate (pred | PLGLB1/PLGLB2 | plasminogen like B2 | Extracellular Space | peptidase |
| MIMAT0004560 | miR-183-3p (miRNAs w/seed UGAAUUA) | 2.300 | TargetScan Human | Moderate (predicted) | PLIN2 | perilipin 2 | Plasma Membrane | other |
| MIMAT0004560 | miR-183-3p (miRNAs w/seed UGAAUUA) | 2.300 | TargetScan Human | High (predicted) | PRB4 | proline rich protein BstNI subfamily 4 | Extracellular Space | other |
| MIMAT0004560 | miR-183-3p (miRNAs w/seed UGAAUUA) | 2.300 | TargetScan Human | Moderate (predicted) | PSMC6 | proteasome 26S subunit, ATPase 6 | Nucleus | peptidase |
| MIMAT0004560 | miR-183-3p (miRNAs w/seed UGAAUUA) | 2.300 | TargetScan Human | Moderate (predicted) | PSMG2 | proteasome assembly chaperone 2 | Nucleus | other |
| MIMAT0004560 | miR-183-3p (miRNAs w/seed UGAAUUA) | 2.300 | TargetScan Human | Moderate (predicted) | PTH | parathyroid hormone | Extracellular Space | other |
| MIMAT0004560 | miR-183-3p (miRNAs w/seed UGAAUUA) | 2.300 | TargetScan Human | Moderate (predicted) | RAB5C | RAB5C, member RAS oncogene family | Cytoplasm | enzyme |
| MIMAT0004560 | miR-183-3p (miRNAs w/seed UGAAUUA) | 2.300 | TargetScan Human | Moderate (predicted) | RGS1 | regulator of G protein signaling 1 | Plasma Membrane | enzyme |
| MIMAT0004560 | miR-183-3p (miRNAs w/seed UGAAUUA) | 2.300 | TargetScan Human | High (predicted) | RP11_67H21 | | Other | other |
| MIMAT0004560 | miR-183-3p (miRNAs w/seed UGAAUUA) | 2.300 | TargetScan Human | Moderate (predicted) | RP4_598P131 | | Other | other |
| MIMAT0004560 | miR-183-3p (miRNAs w/seed UGAAUUA) | 2.300 | TargetScan Human | Moderate (predicted) | SDHAF3 | nate dehydrogenase complex assembly fa | Cytoplasm | other |
| MIMAT0004560 | miR-183-3p (miRNAs w/seed UGAAUUA) | 2.300 | TargetScan Human | Moderate (predicted) | SEC61B | SEC61 translocon subunit beta | Cytoplasm | transporter |
| MIMAT0004560 | miR-183-3p (miRNAs w/seed UGAAUUA) | 2.300 | TargetScan Human | Moderate (predicted) | SLN | sarcolipin | Cytoplasm | other |
| MIMAT0004560 | miR-183-3p (miRNAs w/seed UGAAUUA) | 2.300 | TargetScan Human | Moderate (predicted) | SPCS1 | signal peptidase complex subunit 1 | Cytoplasm | peptidase |
| MIMAT0004560 | miR-183-3p (miRNAs w/seed UGAAUUA) | 2.300 | TargetScan Human | Moderate (predicted) | SPINK6 | serine peptidase inhibitor Kazal type 6 | Extracellular Space | other |
| MIMAT0004560 | miR-183-3p (miRNAs w/seed UGAAUUA) | 2.300 | TargetScan Human | Moderate (predicted) | SRSF1 | serine and arginine rich splicing factor 1 | Nucleus | other |
| MIMAT0004560 | miR-183-3p (miRNAs w/seed UGAAUUA) | 2.300 | TargetScan Human | Moderate (predicted) | SRSF7 | serine and arginine rich splicing factor 7 | Nucleus | other |
| MIMAT0004560 | miR-183-3p (miRNAs w/seed UGAAUUA) | 2.300 | TargetScan Human | Moderate (predicted) | STON1-GTF2A1L | STON1-GTF2A1L readthrough | Cytoplasm | other |
| MIMAT0004560 | miR-183-3p (miRNAs w/seed UGAAUUA) | 2.300 | TargetScan Human | High (predicted) | SULT6B1 | sulfotransferase family 6B member 1 | Cytoplasm | enzyme |
| MIMAT0004560 | miR-183-3p (miRNAs w/seed UGAAUUA) | 2.300 | TargetScan Human | Moderate (predicted) | TMEM126B | transmembrane protein 126B | Cytoplasm | other |
| MIMAT0004560 | miR-183-3p (miRNAs w/seed UGAAUUA) | 2.300 | TargetScan Human | Moderate (predicted) | TMEM185A | transmembrane protein 185A | Nucleus | other |
| MIMAT0004560 | miR-183-3p (miRNAs w/seed UGAAUUA) | 2.300 | TargetScan Human | Moderate (predicted) | TMEM97 | transmembrane protein 97 | Extracellular Space | other |
| MIMAT0004560 | miR-183-3p (miRNAs w/seed UGAAUUA) | 2.300 | TargetScan Human | Moderate (predicted) | TRIQK | triple QxxK/R motif containing | Cytoplasm | other |
| MIMAT0004560 | miR-183-3p (miRNAs w/seed UGAAUUA) | 2.300 | TargetScan Human | Moderate (predicted) | UBXN4 | UBX domain protein 4 | Extracellular Space | other |
| MIMAT0004560 | miR-183-3p (miRNAs w/seed UGAAUUA) | 2.300 | TargetScan Human | Moderate (predicted) | UGT2B28 | glucuronosyltransferase family 2 member | Cytoplasm | enzyme |
| MIMAT0004560 | miR-183-3p (miRNAs w/seed UGAAUUA) | 2.300 | TargetScan Human | Moderate (predicted) | VIP | vasoactive intestinal peptide | Extracellular Space | other |
| MIMAT0004560 | miR-183-3p (miRNAs w/seed UGAAUUA) | 2.300 | TargetScan Human | Moderate (predicted) | ZNF138 | zinc finger protein 138 | Nucleus | transcription regulator |
| MIMAT0004560 | miR-183-3p (miRNAs w/seed UGAAUUA) | 2.300 | TargetScan Human | Moderate (predicted) | ZNF30 | zinc finger protein 30 | Nucleus | transcription regulator |
| MIMAT0004560 | miR-183-3p (miRNAs w/seed UGAAUUA) | 2.300 | TargetScan Human | Moderate (predicted) | ZNF519 | zinc finger protein 519 | Nucleus | transcription regulator |
| MIMAT0004560 | miR-183-3p (miRNAs w/seed UGAAUUA) | 2.300 | TargetScan Human | High (predicted) | ZNF99 | zinc finger protein 99 | Other | other |
| MIMAT0003236 | miR-571 (miRNAs w/seed GAGUUGG) | 2.230 | TargetScan Human | Moderate (predicted) | AADACL3 | arylacetamide deacetylase like 3 | Other | other |
| MIMAT0003236 | miR-571 (miRNAs w/seed GAGUUGG) | 2.230 | TargetScan Human | Moderate (predicted) | ABCG1 | TP binding cassette subfamily G member | Plasma Membrane | transporter |
| MIMAT0003236 | miR-571 (miRNAs w/seed GAGUUGG) | 2.230 | TargetScan Human | High (predicted) | ABRACL | ABRA C-terminal like | Other | other |
| MIMAT0003236 | miR-571 (miRNAs w/seed GAGUUGG) | 2.230 | TargetScan Human | Moderate (predicted) | ACAA1 | acetyl-CoA acyltransferase 1 | Cytoplasm | enzyme |
| MIMAT0003236 | miR-571 (miRNAs w/seed GAGUUGG) | 2.230 | TargetScan Human | Moderate (predicted) | ACRV1 | acrosomal vesicle protein 1 | Cytoplasm | other |
| MIMAT0003236 | miR-571 (miRNAs w/seed GAGUUGG) | 2.230 | TargetScan Human | Moderate (predicted) | ACSBG1 | -CoA synthetase bubblegum family memb | Cytoplasm | enzyme |
| MIMAT0003236 | miR-571 (miRNAs w/seed GAGUUGG) | 2.230 | TargetScan Human | Moderate (predicted) | ACSL1 | l-CoA synthetase long chain family memb | Cytoplasm | enzyme |
| MIMAT0003236 | miR-571 (miRNAs w/seed GAGUUGG) | 2.230 | TargetScan Human | Moderate (predicted) | ACSM5 | CoA synthetase medium chain family mem | Other | enzyme |
| MIMAT0003236 | miR-571 (miRNAs w/seed GAGUUGG) | 2.230 | TargetScan Human | Moderate (predicted) | ACTL6A | actin like 6A | Nucleus | other |
| MIMAT0003236 | miR-571 (miRNAs w/seed GAGUUGG) | 2.230 | TargetScan Human | Moderate (predicted) | ACTR1A | actin related protein 1A | Cytoplasm | other |
| MIMAT0003236 | miR-571 (miRNAs w/seed GAGUUGG) | 2.230 | TargetScan Human | Moderate (predicted) | ADA2 | adenosine deaminase 2 | Extracellular Space | enzyme |
| MIMAT0003236 | miR-571 (miRNAs w/seed GAGUUGG) | 2.230 | TargetScan Human | Moderate (predicted) | ADAM12 | ADAM metallopeptidase domain 12 | Plasma Membrane | peptidase |
| MIMAT0003236 | miR-571 (miRNAs w/seed GAGUUGG) | 2.230 | TargetScan Human | Moderate (predicted) | ADAMTS3 | etallopeptidase with thrombospondin type | Extracellular Space | peptidase |
| MIMAT0003236 | miR-571 (miRNAs w/seed GAGUUGG) | 2.230 | TargetScan Human | Moderate (predicted) | ADD2 | adducin 2 | Cytoplasm | other |
| MIMAT0003236 | miR-571 (miRNAs w/seed GAGUUGG) | 2.230 | TargetScan Human | Moderate (predicted) | ADH6 | alcohol dehydrogenase 6 (class V) | Cytoplasm | enzyme |
| MIMAT0003236 | miR-571 (miRNAs w/seed GAGUUGG) | 2.230 | TargetScan Human | Moderate (predicted) | ADM2 | adrenomedullin 2 | Extracellular Space | other |
| MIMAT0003236 | miR-571 (miRNAs w/seed GAGUUGG) | 2.230 | TargetScan Human | Moderate (predicted) | ADPRH | ADP-ribosylarginine hydrolase | Other | enzyme |
| MIMAT0003236 | miR-571 (miRNAs w/seed GAGUUGG) | 2.230 | TargetScan Human | Moderate (predicted) | ADRA2A | adrenoceptor alpha 2A | Plasma Membrane | G-protein coupled receptor |
| MIMAT0003236 | miR-571 (miRNAs w/seed GAGUUGG) | 2.230 | TargetScan Human | High (predicted) | AEBP1 | AE binding protein 1 | Nucleus | peptidase |
| MIMAT0003236 | miR-571 (miRNAs w/seed GAGUUGG) | 2.230 | TargetScan Human | Moderate (predicted) | AFAP1L2 | actin filament associated protein 1 like 2 | Cytoplasm | other |
| MIMAT0003236 | miR-571 (miRNAs w/seed GAGUUGG) | 2.230 | TargetScan Human | High (predicted) | ALDH3B2 | dehyde dehydrogenase 3 family member | Cytoplasm | enzyme |
| MIMAT0003236 | miR-571 (miRNAs w/seed GAGUUGG) | 2.230 | TargetScan Human | Moderate (predicted) | ALOX15B | arachidonate 15-lipoxygenase type B | Cytoplasm | enzyme |
| MIMAT0003236 | miR-571 (miRNAs w/seed GAGUUGG) | 2.230 | TargetScan Human | Moderate (predicted) | ALOXE3 | arachidonate lipoxygenase 3 | Extracellular Space | enzyme |
| MIMAT0003236 | miR-571 (miRNAs w/seed GAGUUGG) | 2.230 | TargetScan Human | Moderate (predicted) | ALPK1 | alpha kinase 1 | Cytoplasm | kinase |
| MIMAT0003236 | miR-571 (miRNAs w/seed GAGUUGG) | 2.230 | TargetScan Human | Moderate (predicted) | ANKIB1 | nkyrin repeat and IBR domain containing | Nucleus | transcription regulator |
| MIMAT0003236 | miR-571 (miRNAs w/seed GAGUUGG) | 2.230 | TargetScan Human | Moderate (predicted) | ANKZF1 | repeat and zinc finger peptidyl tRNA hyd | Nucleus | transcription regulator |
| MIMAT0003236 | miR-571 (miRNAs w/seed GAGUUGG) | 2.230 | TargetScan Human | Moderate (predicted) | ANXA8/ANXA8L1 | annexin A8 like 1 | Plasma Membrane | other |
| MIMAT0003236 | miR-571 (miRNAs w/seed GAGUUGG) | 2.230 | TargetScan Human | Moderate (predicted) | APOBEC3H | rotein B mRNA editing enzyme catalytic su | Cytoplasm | enzyme |
| MIMAT0003236 | miR-571 (miRNAs w/seed GAGUUGG) | 2.230 | TargetScan Human | Moderate (predicted) | APOC2 | apolipoprotein C2 | Extracellular Space | transporter |
| MIMAT0003236 | miR-571 (miRNAs w/seed GAGUUGG) | 2.230 | TargetScan Human | Moderate (predicted) | APOC4 | apolipoprotein C4 | Extracellular Space | transporter |
| MIMAT0003236 | miR-571 (miRNAs w/seed GAGUUGG) | 2.230 | TargetScan Human | High (predicted) | APOC4-APOC2 | POC4-APOC2 readthrough (NMD candida | Other | other |
| MIMAT0003236 | miR-571 (miRNAs w/seed GAGUUGG) | 2.230 | TargetScan Human | Moderate (predicted) | ARF3 | ADP ribosylation factor 3 | Cytoplasm | enzyme |
| MIMAT0003236 | miR-571 (miRNAs w/seed GAGUUGG) | 2.230 | TargetScan Human | Moderate (predicted) | ARHGAP25 | Rho GTPase activating protein 25 | Cytoplasm | other |
| MIMAT0003236 | miR-571 (miRNAs w/seed GAGUUGG) | 2.230 | TargetScan Human | Moderate (predicted) | ARHGAP32 | Rho GTPase activating protein 32 | Cytoplasm | other |
| MIMAT0003236 | miR-571 (miRNAs w/seed GAGUUGG) | 2.230 | TargetScan Human | Moderate (predicted) | ARHGAP5 | Rho GTPase activating protein 5 | Cytoplasm | enzyme |
| MIMAT0003236 | miR-571 (miRNAs w/seed GAGUUGG) | 2.230 | TargetScan Human | Moderate (predicted) | ARHGEF3 | Rho guanine nucleotide exchange factor 3 | Cytoplasm | other |
| MIMAT0003236 | miR-571 (miRNAs w/seed GAGUUGG) | 2.230 | TargetScan Human | High (predicted) | ARHGEF39 | Rho guanine nucleotide exchange factor 3 | Plasma Membrane | other |
| MIMAT0003236 | miR-571 (miRNAs w/seed GAGUUGG) | 2.230 | TargetScan Human | Moderate (predicted) | ARL4A | ADP ribosylation factor like GTPase 4A | Nucleus | enzyme |
| MIMAT0003236 | miR-571 (miRNAs w/seed GAGUUGG) | 2.230 | TargetScan Human | Moderate (predicted) | ARX | aristaless related homeobox | Nucleus | transcription regulator |
| MIMAT0003236 | miR-571 (miRNAs w/seed GAGUUGG) | 2.230 | TargetScan Human | High (predicted) | ASAP3 | with SH3 domain, ankyrin repeat and PH d | Plasma Membrane | other |
| MIMAT0003236 | miR-571 (miRNAs w/seed GAGUUGG) | 2.230 | TargetScan Human | Moderate (predicted) | ASB13 | nkyrin repeat and SOCS box containing 1 | Cytoplasm | other |
| MIMAT0003236 | miR-571 (miRNAs w/seed GAGUUGG) | 2.230 | TargetScan Human | Moderate (predicted) | ASB6 | ankyrin repeat and SOCS box containing 6 | Nucleus | transcription regulator |
| MIMAT0003236 | miR-571 (miRNAs w/seed GAGUUGG) | 2.230 | TargetScan Human | Moderate (predicted) | ASNS | paragine synthetase (glutamine-hydrolyzi | Cytoplasm | enzyme |
| MIMAT0003236 | miR-571 (miRNAs w/seed GAGUUGG) | 2.230 | TargetScan Human | Moderate (predicted) | ATAD2 | ATPase family AAA domain containing 2 | Nucleus | enzyme |
| MIMAT0003236 | miR-571 (miRNAs w/seed GAGUUGG) | 2.230 | TargetScan Human | Moderate (predicted) | ATF5 | activating transcription factor 5 | Nucleus | transcription regulator |
| MIMAT0003236 | miR-571 (miRNAs w/seed GAGUUGG) | 2.230 | TargetScan Human | Moderate (predicted) | ATG12 | autophagy related 12 | Cytoplasm | other |
| MIMAT0003236 | miR-571 (miRNAs w/seed GAGUUGG) | 2.230 | TargetScan Human | High (predicted) | ATP5MK | ATP synthase membrane subunit k | Cytoplasm | other |
| MIMAT0003236 | miR-571 (miRNAs w/seed GAGUUGG) | 2.230 | TargetScan Human | Moderate (predicted) | ATXN2 | ataxin 2 | Nucleus | other |
| MIMAT0003236 | miR-571 (miRNAs w/seed GAGUUGG) | 2.230 | TargetScan Human | High (predicted) | AUP1 | 1 lipid droplet regulating VLDL assembly f | Cytoplasm | other |
| MIMAT0003236 | miR-571 (miRNAs w/seed GAGUUGG) | 2.230 | TargetScan Human | Moderate (predicted) | BAALC | BAALC binder of MAP3K1 and KLF4 | Cytoplasm | other |
| MIMAT0003236 | miR-571 (miRNAs w/seed GAGUUGG) | 2.230 | TargetScan Human | Moderate (predicted) | BBLN | bublin coiled coil protein | Other | other |
| MIMAT0003236 | miR-571 (miRNAs w/seed GAGUUGG) | 2.230 | TargetScan Human | Moderate (predicted) | BCL2L11 | BCL2 like 11 | Cytoplasm | other |
| MIMAT0003236 | miR-571 (miRNAs w/seed GAGUUGG) | 2.230 | TargetScan Human | Moderate (predicted) | BCO2 | beta-carotene oxygenase 2 | Cytoplasm | enzyme |
| MIMAT0003236 | miR-571 (miRNAs w/seed GAGUUGG) | 2.230 | TargetScan Human | Moderate (predicted) | BDKRB2 | bradykinin receptor B2 | Plasma Membrane | G-protein coupled receptor |
| MIMAT0003236 | miR-571 (miRNAs w/seed GAGUUGG) | 2.230 | TargetScan Human | Moderate (predicted) | BICD1 | BICD cargo adaptor 1 | Cytoplasm | other |
| MIMAT0003236 | miR-571 (miRNAs w/seed GAGUUGG) | 2.230 | TargetScan Human | Moderate (predicted) | BORCS6 | BLOC-1 related complex subunit 6 | Cytoplasm | other |
| MIMAT0003236 | miR-571 (miRNAs w/seed GAGUUGG) | 2.230 | TargetScan Human | Moderate (predicted) | BRI3 | brain protein I3 | Cytoplasm | other |
| MIMAT0003236 | miR-571 (miRNAs w/seed GAGUUGG) | 2.230 | TargetScan Human | Moderate (predicted) | C10orf53 | chromosome 10 open reading frame 53 | Other | other |
| MIMAT0003236 | miR-571 (miRNAs w/seed GAGUUGG) | 2.230 | TargetScan Human | Moderate (predicted) | C16orf54 | chromosome 16 open reading frame 54 | Extracellular Space | other |
| MIMAT0003236 | miR-571 (miRNAs w/seed GAGUUGG) | 2.230 | TargetScan Human | High (predicted) | C2orf73 | chromosome 2 open reading frame 73 | Other | other |
| MIMAT0003236 | miR-571 (miRNAs w/seed GAGUUGG) | 2.230 | TargetScan Human | Moderate (predicted) | C6 | complement C6 | Extracellular Space | other |
| MIMAT0003236 | miR-571 (miRNAs w/seed GAGUUGG) | 2.230 | TargetScan Human | High (predicted) | C9orf57 | chromosome 9 open reading frame 57 | Other | other |
| MIMAT0003236 | miR-571 (miRNAs w/seed GAGUUGG) | 2.230 | TargetScan Human | Moderate (predicted) | CA13 | carbonic anhydrase 13 | Cytoplasm | enzyme |
| MIMAT0003236 | miR-571 (miRNAs w/seed GAGUUGG) | 2.230 | TargetScan Human | Moderate (predicted) | CACNG6 | voltage-gated channel auxiliary subunit g | Plasma Membrane | ion channel |
| MIMAT0003236 | miR-571 (miRNAs w/seed GAGUUGG) | 2.230 | TargetScan Human | Moderate (predicted) | CACNG7 | voltage-gated channel auxiliary subunit g | Plasma Membrane | ion channel |
| MIMAT0003236 | miR-571 (miRNAs w/seed GAGUUGG) | 2.230 | TargetScan Human | High (predicted) | CALU | calumenin | Cytoplasm | other |
| MIMAT0003236 | miR-571 (miRNAs w/seed GAGUUGG) | 2.230 | TargetScan Human | High (predicted) | CAPN3 | calpain 3 | Cytoplasm | peptidase |
| MIMAT0003236 | miR-571 (miRNAs w/seed GAGUUGG) | 2.230 | TargetScan Human | Moderate (predicted) | CASP14 | caspase 14 | Cytoplasm | peptidase |
| MIMAT0003236 | miR-571 (miRNAs w/seed GAGUUGG) | 2.230 | TargetScan Human | High (predicted) | CASP8 | caspase 8 | Nucleus | peptidase |
| MIMAT0003236 | miR-571 (miRNAs w/seed GAGUUGG) | 2.230 | TargetScan Human | Moderate (predicted) | CBLB | Cbl proto-oncogene B | Nucleus | enzyme |
| MIMAT0003236 | miR-571 (miRNAs w/seed GAGUUGG) | 2.230 | TargetScan Human | Moderate (predicted) | CCDC106 | coiled-coil domain containing 106 | Extracellular Space | other |
| MIMAT0003236 | miR-571 (miRNAs w/seed GAGUUGG) | 2.230 | TargetScan Human | High (predicted) | CCDC38 | coiled-coil domain containing 38 | Cytoplasm | other |
| MIMAT0003236 | miR-571 (miRNAs w/seed GAGUUGG) | 2.230 | TargetScan Human | Moderate (predicted) | CCND3 | cyclin D3 | Nucleus | kinase |
| MIMAT0003236 | miR-571 (miRNAs w/seed GAGUUGG) | 2.230 | TargetScan Human | Moderate (predicted) | CD160 | CD160 molecule | Plasma Membrane | transmembrane receptor |
| MIMAT0003236 | miR-571 (miRNAs w/seed GAGUUGG) | 2.230 | TargetScan Human | Moderate (predicted) | CD1E | CD1e molecule | Cytoplasm | other |
| MIMAT0003236 | miR-571 (miRNAs w/seed GAGUUGG) | 2.230 | TargetScan Human | Moderate (predicted) | CD209 | CD209 molecule | Plasma Membrane | other |
| MIMAT0003236 | miR-571 (miRNAs w/seed GAGUUGG) | 2.230 | TargetScan Human | Moderate (predicted) | CD300LB | CD300 molecule like family member b | Plasma Membrane | other |
| MIMAT0003236 | miR-571 (miRNAs w/seed GAGUUGG) | 2.230 | TargetScan Human | Moderate (predicted) | CD4 | CD4 molecule | Plasma Membrane | transmembrane receptor |
| MIMAT0003236 | miR-571 (miRNAs w/seed GAGUUGG) | 2.230 | TargetScan Human | Moderate (predicted) | CD46 | CD46 molecule | Plasma Membrane | transmembrane receptor |
| MIMAT0003236 | miR-571 (miRNAs w/seed GAGUUGG) | 2.230 | TargetScan Human | Moderate (predicted) | CD84 | CD84 molecule | Plasma Membrane | other |
| MIMAT0003236 | miR-571 (miRNAs w/seed GAGUUGG) | 2.230 | TargetScan Human | Moderate (predicted) | CD99L2 | CD99 molecule like 2 | Plasma Membrane | other |
| MIMAT0003236 | miR-571 (miRNAs w/seed GAGUUGG) | 2.230 | TargetScan Human | High (predicted) | CDRT1 | CMT1A duplicated region transcript 1 | Other | other |
| MIMAT0003236 | miR-571 (miRNAs w/seed GAGUUGG) | 2.230 | TargetScan Human | Moderate (predicted) | CDX4 | caudal type homeobox 4 | Nucleus | transcription regulator |
| MIMAT0003236 | miR-571 (miRNAs w/seed GAGUUGG) | 2.230 | TargetScan Human | Moderate (predicted) | CEACAM19 | CEA cell adhesion molecule 19 | Other | other |
| MIMAT0003236 | miR-571 (miRNAs w/seed GAGUUGG) | 2.230 | TargetScan Human | Moderate (predicted) | CEACAM6 | CEA cell adhesion molecule 6 | Plasma Membrane | other |
| MIMAT0003236 | miR-571 (miRNAs w/seed GAGUUGG) | 2.230 | TargetScan Human | Moderate (predicted) | CEMP1 | cementum protein 1 | Nucleus | other |
| MIMAT0003236 | miR-571 (miRNAs w/seed GAGUUGG) | 2.230 | TargetScan Human | Moderate (predicted) | CERS1 | ceramide synthase 1 | Cytoplasm | enzyme |
| MIMAT0003236 | miR-571 (miRNAs w/seed GAGUUGG) | 2.230 | TargetScan Human | Moderate (predicted) | CHAD | chondroadherin | Extracellular Space | other |
| MIMAT0003236 | miR-571 (miRNAs w/seed GAGUUGG) | 2.230 | TargetScan Human | Moderate (predicted) | CHL1 | cell adhesion molecule L1 like | Plasma Membrane | other |
| MIMAT0003236 | miR-571 (miRNAs w/seed GAGUUGG) | 2.230 | TargetScan Human | Moderate (predicted) | CHP1 | calcineurin like EF-hand protein 1 | Cytoplasm | other |
| MIMAT0003236 | miR-571 (miRNAs w/seed GAGUUGG) | 2.230 | TargetScan Human | High (predicted) | CHP2 | calcineurin like EF-hand protein 2 | Cytoplasm | other |
| MIMAT0003236 | miR-571 (miRNAs w/seed GAGUUGG) | 2.230 | TargetScan Human | Moderate (predicted) | CHTF8 | chromosome transmission fidelity factor 8 | Cytoplasm | other |
| MIMAT0003236 | miR-571 (miRNAs w/seed GAGUUGG) | 2.230 | TargetScan Human | Moderate (predicted) | CIAPIN1 | cytokine induced apoptosis inhibitor 1 | Cytoplasm | other |
| MIMAT0003236 | miR-571 (miRNAs w/seed GAGUUGG) | 2.230 | TargetScan Human | Moderate (predicted) | CIRBP | cold inducible RNA binding protein | Nucleus | translation regulator |
| MIMAT0003236 | miR-571 (miRNAs w/seed GAGUUGG) | 2.230 | TargetScan Human | Moderate (predicted) | CIT | itron rho-interacting serine/threonine kinas | Cytoplasm | kinase |
| MIMAT0003236 | miR-571 (miRNAs w/seed GAGUUGG) | 2.230 | TargetScan Human | Moderate (predicted) | CLDN7 | claudin 7 | Plasma Membrane | other |
| MIMAT0003236 | miR-571 (miRNAs w/seed GAGUUGG) | 2.230 | TargetScan Human | High (predicted) | CLIC2 | chloride intracellular channel 2 | Cytoplasm | enzyme |
| MIMAT0003236 | miR-571 (miRNAs w/seed GAGUUGG) | 2.230 | TargetScan Human | Moderate (predicted) | CLIC5 | chloride intracellular channel 5 | Cytoplasm | ion channel |
| MIMAT0003236 | miR-571 (miRNAs w/seed GAGUUGG) | 2.230 | TargetScan Human | Moderate (predicted) | CNKSR2 | hector enhancer of kinase suppressor of R | Plasma Membrane | other |
| MIMAT0003236 | miR-571 (miRNAs w/seed GAGUUGG) | 2.230 | TargetScan Human | Moderate (predicted) | CNR2 | cannabinoid receptor 2 | Plasma Membrane | G-protein coupled receptor |
| MIMAT0003236 | miR-571 (miRNAs w/seed GAGUUGG) | 2.230 | TargetScan Human | Moderate (predicted) | CNTD1 | cyclin N-terminal domain containing 1 | Other | other |
| MIMAT0003236 | miR-571 (miRNAs w/seed GAGUUGG) | 2.230 | TargetScan Human | Moderate (predicted) | COL20A1 | collagen type XX alpha 1 chain | Extracellular Space | other |
| MIMAT0003236 | miR-571 (miRNAs w/seed GAGUUGG) | 2.230 | TargetScan Human | Moderate (predicted) | COP1 | COP1 E3 ubiquitin ligase | Cytoplasm | enzyme |
| MIMAT0003236 | miR-571 (miRNAs w/seed GAGUUGG) | 2.230 | TargetScan Human | Moderate (predicted) | COPA | COPI coat complex subunit alpha | Cytoplasm | transporter |
| MIMAT0003236 | miR-571 (miRNAs w/seed GAGUUGG) | 2.230 | TargetScan Human | Moderate (predicted) | COPZ1 | COPI coat complex subunit zeta 1 | Cytoplasm | transporter |
| MIMAT0003236 | miR-571 (miRNAs w/seed GAGUUGG) | 2.230 | TargetScan Human | High (predicted) | COX7C | cytochrome c oxidase subunit 7C | Cytoplasm | enzyme |
| MIMAT0003236 | miR-571 (miRNAs w/seed GAGUUGG) | 2.230 | TargetScan Human | Moderate (predicted) | CRAT | carnitine O-acetyltransferase | Cytoplasm | enzyme |
| MIMAT0003236 | miR-571 (miRNAs w/seed GAGUUGG) | 2.230 | TargetScan Human | Moderate (predicted) | CREBBP | CREB binding protein | Nucleus | transcription regulator |
| MIMAT0003236 | miR-571 (miRNAs w/seed GAGUUGG) | 2.230 | TargetScan Human | Moderate (predicted) | CREBRF | CREB3 regulatory factor | Nucleus | transcription regulator |
| MIMAT0003236 | miR-571 (miRNAs w/seed GAGUUGG) | 2.230 | TargetScan Human | Moderate (predicted) | CREBZF | CREB/ATF bZIP transcription factor | Nucleus | transcription regulator |

Sup. Table 3. List of 3699 targets of the seven dysregulated miRNAs obtained from three different target prediction databases,TargetScan TarBase and miRecords considering those predicted as moderate, high, or experimentally validated, respectively.

| | | | | | | | | |
|---|---|---|---|---|---|---|---|---|
| MIMAT0003236 | miR-571 (miRNAs w/seed GAGUUGG) | 2.230 | TargetScan Human | Moderate (predicted) | CREG2 | ellular repressor of E1A stimulated genes | Extracellular Space | enzyme |
| MIMAT0003236 | miR-571 (miRNAs w/seed GAGUUGG) | 2.230 | TargetScan Human | Moderate (predicted) | CRISPLD1 | e rich secretory protein LCCL domain cont | Cytoplasm | other |
| MIMAT0003236 | miR-571 (miRNAs w/seed GAGUUGG) | 2.230 | TargetScan Human | High (predicted) | CSNK2A1 | casein kinase 2 alpha 1 | Nucleus | kinase |
| MIMAT0003236 | miR-571 (miRNAs w/seed GAGUUGG) | 2.230 | TargetScan Human | High (predicted) | CSRNP3 | cysteine and serine rich nuclear protein 3 | Nucleus | transcription regulator |
| MIMAT0003236 | miR-571 (miRNAs w/seed GAGUUGG) | 2.230 | TargetScan Human | Moderate (predicted) | CST3 | cystatin C | Extracellular Space | other |
| MIMAT0003236 | miR-571 (miRNAs w/seed GAGUUGG) | 2.230 | TargetScan Human | Moderate (predicted) | CTDSPL | CTD small phosphatase like | Nucleus | other |
| MIMAT0003236 | miR-571 (miRNAs w/seed GAGUUGG) | 2.230 | TargetScan Human | High (predicted) | CX3CR1 | C-X3-C motif chemokine receptor 1 | Plasma Membrane | G-protein coupled receptor |
| MIMAT0003236 | miR-571 (miRNAs w/seed GAGUUGG) | 2.230 | TargetScan Human | Moderate (predicted) | CYB5D1 | cytochrome b5 domain containing 1 | Other | other |
| MIMAT0003236 | miR-571 (miRNAs w/seed GAGUUGG) | 2.230 | TargetScan Human | High (predicted) | CYB5D2 | cytochrome b5 domain containing 2 | Extracellular Space | other |
| MIMAT0003236 | miR-571 (miRNAs w/seed GAGUUGG) | 2.230 | TargetScan Human | Moderate (predicted) | CYP11B2 | chrome P450 family 11 subfamily B memb | Cytoplasm | enzyme |
| MIMAT0003236 | miR-571 (miRNAs w/seed GAGUUGG) | 2.230 | TargetScan Human | High (predicted) | CYP24A1 | chrome P450 family 24 subfamily A memb | Cytoplasm | enzyme |
| MIMAT0003236 | miR-571 (miRNAs w/seed GAGUUGG) | 2.230 | TargetScan Human | Moderate (predicted) | CYP2S1 | ochrome P450 family 2 subfamily S memb | Cytoplasm | enzyme |
| MIMAT0003236 | miR-571 (miRNAs w/seed GAGUUGG) | 2.230 | TargetScan Human | Moderate (predicted) | CYSLTR1 | cysteinyl leukotriene receptor 1 | Plasma Membrane | G-protein coupled receptor |
| MIMAT0003236 | miR-571 (miRNAs w/seed GAGUUGG) | 2.230 | TargetScan Human | Moderate (predicted) | CYTH1 | cytohesin 1 | Cytoplasm | other |
| MIMAT0003236 | miR-571 (miRNAs w/seed GAGUUGG) | 2.230 | TargetScan Human | High (predicted) | DAGLB | diacylglycerol lipase beta | Plasma Membrane | enzyme |
| MIMAT0003236 | miR-571 (miRNAs w/seed GAGUUGG) | 2.230 | TargetScan Human | High (predicted) | DBF4B | DBF4 zinc finger B | Nucleus | other |
| MIMAT0003236 | miR-571 (miRNAs w/seed GAGUUGG) | 2.230 | TargetScan Human | Moderate (predicted) | DCAKD | dephospho-CoA kinase domain containing | Cytoplasm | other |
| MIMAT0003236 | miR-571 (miRNAs w/seed GAGUUGG) | 2.230 | TargetScan Human | Moderate (predicted) | DCTN2 | dynactin subunit 2 | Cytoplasm | other |
| MIMAT0003236 | miR-571 (miRNAs w/seed GAGUUGG) | 2.230 | TargetScan Human | Moderate (predicted) | DCTN3 | dynactin subunit 3 | Nucleus | other |
| MIMAT0003236 | miR-571 (miRNAs w/seed GAGUUGG) | 2.230 | TargetScan Human | Moderate (predicted) | DCTN6 | dynactin subunit 6 | Cytoplasm | enzyme |
| MIMAT0003236 | miR-571 (miRNAs w/seed GAGUUGG) | 2.230 | TargetScan Human | Moderate (predicted) | DCTPP1 | dCTP pyrophosphatase 1 | Cytoplasm | enzyme |
| MIMAT0003236 | miR-571 (miRNAs w/seed GAGUUGG) | 2.230 | TargetScan Human | High (predicted) | DEFB132 | defensin beta 132 | Extracellular Space | other |
| MIMAT0003236 | miR-571 (miRNAs w/seed GAGUUGG) | 2.230 | TargetScan Human | Moderate (predicted) | DENND1B | DENN domain containing 1B | Other | other |
| MIMAT0003236 | miR-571 (miRNAs w/seed GAGUUGG) | 2.230 | TargetScan Human | Moderate (predicted) | DGAT2L6 | diacylglycerol O-acyltransferase 2 like 6 | Cytoplasm | enzyme |
| MIMAT0003236 | miR-571 (miRNAs w/seed GAGUUGG) | 2.230 | TargetScan Human | Moderate (predicted) | DGKB | diacylglycerol kinase beta | Cytoplasm | kinase |
| MIMAT0003236 | miR-571 (miRNAs w/seed GAGUUGG) | 2.230 | TargetScan Human | Moderate (predicted) | DIS3L2 | DIS3 like 3'-5' exoribonuclease 2 | Cytoplasm | enzyme |
| MIMAT0003236 | miR-571 (miRNAs w/seed GAGUUGG) | 2.230 | TargetScan Human | Moderate (predicted) | DKK1 | dickkopf WNT signaling pathway inhibitor | Extracellular Space | growth factor |
| MIMAT0003236 | miR-571 (miRNAs w/seed GAGUUGG) | 2.230 | TargetScan Human | Moderate (predicted) | DMBT1 | deleted in malignant brain tumors 1 | Plasma Membrane | transmembrane receptor |
| MIMAT0003236 | miR-571 (miRNAs w/seed GAGUUGG) | 2.230 | TargetScan Human | Moderate (predicted) | DNAH17-AS1 | DNAH17 antisense RNA 1 | Other | other |
| MIMAT0003236 | miR-571 (miRNAs w/seed GAGUUGG) | 2.230 | TargetScan Human | Moderate (predicted) | DPYSL5 | dihydropyrimidinase like 5 | Cytoplasm | enzyme |
| MIMAT0003236 | miR-571 (miRNAs w/seed GAGUUGG) | 2.230 | TargetScan Human | High (predicted) | DSCAML1 | DS cell adhesion molecule like 1 | Plasma Membrane | other |
| MIMAT0003236 | miR-571 (miRNAs w/seed GAGUUGG) | 2.230 | TargetScan Human | Moderate (predicted) | DTNA | dystrobrevin alpha | Plasma Membrane | other |
| MIMAT0003236 | miR-571 (miRNAs w/seed GAGUUGG) | 2.230 | TargetScan Human | Moderate (predicted) | DUSP18 | dual specificity phosphatase 18 | Cytoplasm | phosphatase |
| MIMAT0003236 | miR-571 (miRNAs w/seed GAGUUGG) | 2.230 | TargetScan Human | High (predicted) | DUSP22 | dual specificity phosphatase 22 | Cytoplasm | phosphatase |
| MIMAT0003236 | miR-571 (miRNAs w/seed GAGUUGG) | 2.230 | TargetScan Human | Moderate (predicted) | DUSP3 | dual specificity phosphatase 3 | Cytoplasm | phosphatase |
| MIMAT0003236 | miR-571 (miRNAs w/seed GAGUUGG) | 2.230 | TargetScan Human | High (predicted) | DYNAP | dynactin associated protein | Other | other |
| MIMAT0003236 | miR-571 (miRNAs w/seed GAGUUGG) | 2.230 | TargetScan Human | Moderate (predicted) | EDN3 | endothelin 3 | Extracellular Space | other |
| MIMAT0003236 | miR-571 (miRNAs w/seed GAGUUGG) | 2.230 | TargetScan Human | Moderate (predicted) | EGR4 | early growth response 4 | Nucleus | transcription regulator |
| MIMAT0003236 | miR-571 (miRNAs w/seed GAGUUGG) | 2.230 | TargetScan Human | Moderate (predicted) | EIF3L | karyotic translation initiation factor 3 subur | Cytoplasm | translation regulator |
| MIMAT0003236 | miR-571 (miRNAs w/seed GAGUUGG) | 2.230 | TargetScan Human | Moderate (predicted) | EIF4E3 | otic translation initiation factor 4E family me | Cytoplasm | translation regulator |
| MIMAT0003236 | miR-571 (miRNAs w/seed GAGUUGG) | 2.230 | TargetScan Human | Moderate (predicted) | ELP5 | ongator acetyltransferase complex subuni | Cytoplasm | other |
| MIMAT0003236 | miR-571 (miRNAs w/seed GAGUUGG) | 2.230 | TargetScan Human | Moderate (predicted) | ENDOD1 | endonuclease domain containing 1 | Extracellular Space | enzyme |
| MIMAT0003236 | miR-571 (miRNAs w/seed GAGUUGG) | 2.230 | TargetScan Human | Moderate (predicted) | ENTPD1 | nucleoside triphosphate diphosphohydrola | Plasma Membrane | enzyme |
| MIMAT0003236 | miR-571 (miRNAs w/seed GAGUUGG) | 2.230 | TargetScan Human | Moderate (predicted) | EPB41 | erythrocyte membrane protein band 4.1 | Plasma Membrane | other |
| MIMAT0003236 | miR-571 (miRNAs w/seed GAGUUGG) | 2.230 | TargetScan Human | Moderate (predicted) | EPHB2 | EPH receptor B2 | Plasma Membrane | kinase |
| MIMAT0003236 | miR-571 (miRNAs w/seed GAGUUGG) | 2.230 | TargetScan Human | Moderate (predicted) | EPHX1 | epoxide hydrolase 1 | Cytoplasm | peptidase |
| MIMAT0003236 | miR-571 (miRNAs w/seed GAGUUGG) | 2.230 | TargetScan Human | Moderate (predicted) | EREG | epiregulin | Extracellular Space | growth factor |
| MIMAT0003236 | miR-571 (miRNAs w/seed GAGUUGG) | 2.230 | TargetScan Human | Moderate (predicted) | ERG | ETS transcription factor ERG | Nucleus | transcription regulator |
| MIMAT0003236 | miR-571 (miRNAs w/seed GAGUUGG) | 2.230 | TargetScan Human | High (predicted) | ERLIN2 | ER lipid raft associated 2 | Cytoplasm | other |
| MIMAT0003236 | miR-571 (miRNAs w/seed GAGUUGG) | 2.230 | TargetScan Human | High (predicted) | ERP27 | endoplasmic reticulum protein 27 | Other | other |
| MIMAT0003236 | miR-571 (miRNAs w/seed GAGUUGG) | 2.230 | TargetScan Human | Moderate (predicted) | ERVV-1 | genous retrovirus group V member 1, env | Other | other |
| MIMAT0003236 | miR-571 (miRNAs w/seed GAGUUGG) | 2.230 | TargetScan Human | High (predicted) | ESYT1 | extended synaptotagmin 1 | Cytoplasm | other |
| MIMAT0003236 | miR-571 (miRNAs w/seed GAGUUGG) | 2.230 | TargetScan Human | Moderate (predicted) | ETNK2 | ethanolamine kinase 2 | Cytoplasm | kinase |
| MIMAT0003236 | miR-571 (miRNAs w/seed GAGUUGG) | 2.230 | TargetScan Human | Moderate (predicted) | ETS1 | ETS proto-oncogene 1, transcription facto | Nucleus | transcription regulator |
| MIMAT0003236 | miR-571 (miRNAs w/seed GAGUUGG) | 2.230 | TargetScan Human | Moderate (predicted) | ETV3 | ETS variant transcription factor 3 | Nucleus | transcription regulator |
| MIMAT0003236 | miR-571 (miRNAs w/seed GAGUUGG) | 2.230 | TargetScan Human | High (predicted) | EYA1 | A transcriptional coactivator and phosphata | Nucleus | phosphatase |
| MIMAT0003236 | miR-571 (miRNAs w/seed GAGUUGG) | 2.230 | TargetScan Human | Moderate (predicted) | F2RL2 | coagulation factor II thrombin receptor like | Plasma Membrane | G-protein coupled receptor |
| MIMAT0003236 | miR-571 (miRNAs w/seed GAGUUGG) | 2.230 | TargetScan Human | Moderate (predicted) | FAM131B | mily with sequence similarity 131 member | Other | other |
| MIMAT0003236 | miR-571 (miRNAs w/seed GAGUUGG) | 2.230 | TargetScan Human | Moderate (predicted) | FAM131C | mily with sequence similarity 131 member | Other | other |
| MIMAT0003236 | miR-571 (miRNAs w/seed GAGUUGG) | 2.230 | TargetScan Human | High (predicted) | FAM163A | mily with sequence similarity 163 member | Other | other |
| MIMAT0003236 | miR-571 (miRNAs w/seed GAGUUGG) | 2.230 | TargetScan Human | Moderate (predicted) | FAM180B | mily with sequence similarity 180 member | Other | other |
| MIMAT0003236 | miR-571 (miRNAs w/seed GAGUUGG) | 2.230 | TargetScan Human | Moderate (predicted) | FAM189A1 | mily with sequence similarity 189 member | Other | other |
| MIMAT0003236 | miR-571 (miRNAs w/seed GAGUUGG) | 2.230 | TargetScan Human | Moderate (predicted) | FAM98B | amily with sequence similarity 98 member | Nucleus | enzyme |
| MIMAT0003236 | miR-571 (miRNAs w/seed GAGUUGG) | 2.230 | TargetScan Human | High (predicted) | FASLG | Fas ligand | Extracellular Space | cytokine |
| MIMAT0003236 | miR-571 (miRNAs w/seed GAGUUGG) | 2.230 | TargetScan Human | High (predicted) | FAU | ubiquitin like and ribosomal protein S30 fu | Cytoplasm | other |
| MIMAT0003236 | miR-571 (miRNAs w/seed GAGUUGG) | 2.230 | TargetScan Human | Moderate (predicted) | FBXW8 | F-box and WD repeat domain containing 8 | Cytoplasm | enzyme |
| MIMAT0003236 | miR-571 (miRNAs w/seed GAGUUGG) | 2.230 | TargetScan Human | High (predicted) | FCAMR | Fc fragment of IgA and IgM receptor | Plasma Membrane | transmembrane receptor |
| MIMAT0003236 | miR-571 (miRNAs w/seed GAGUUGG) | 2.230 | TargetScan Human | Moderate (predicted) | FCER1G | Fc fragment of IgE receptor Ig | Plasma Membrane | transmembrane receptor |
| MIMAT0003236 | miR-571 (miRNAs w/seed GAGUUGG) | 2.230 | TargetScan Human | Moderate (predicted) | FCGR3A/FCGR3B | Fc fragment of IgG receptor IIIa | Plasma Membrane | transmembrane receptor |
| MIMAT0003236 | miR-571 (miRNAs w/seed GAGUUGG) | 2.230 | TargetScan Human | Moderate (predicted) | FCRL4 | Fc receptor like 4 | Cytoplasm | other |
| MIMAT0003236 | miR-571 (miRNAs w/seed GAGUUGG) | 2.230 | TargetScan Human | Moderate (predicted) | FGF17 | fibroblast growth factor 17 | Extracellular Space | growth factor |
| MIMAT0003236 | miR-571 (miRNAs w/seed GAGUUGG) | 2.230 | TargetScan Human | Moderate (predicted) | FGF23 | fibroblast growth factor 23 | Extracellular Space | growth factor |
| MIMAT0003236 | miR-571 (miRNAs w/seed GAGUUGG) | 2.230 | TargetScan Human | High (predicted) | FGF4 | fibroblast growth factor 4 | Extracellular Space | growth factor |
| MIMAT0003236 | miR-571 (miRNAs w/seed GAGUUGG) | 2.230 | TargetScan Human | High (predicted) | FGF5 | fibroblast growth factor 5 | Extracellular Space | growth factor |
| MIMAT0003236 | miR-571 (miRNAs w/seed GAGUUGG) | 2.230 | TargetScan Human | Moderate (predicted) | FHIT | gile histidine triad diadenosine triphosphat | Cytoplasm | enzyme |
| MIMAT0003236 | miR-571 (miRNAs w/seed GAGUUGG) | 2.230 | TargetScan Human | Moderate (predicted) | FHL1 | four and a half LIM domains 1 | Cytoplasm | other |
| MIMAT0003236 | miR-571 (miRNAs w/seed GAGUUGG) | 2.230 | TargetScan Human | Moderate (predicted) | FKBP5 | FKBP prolyl isomerase 5 | Nucleus | enzyme |
| MIMAT0003236 | miR-571 (miRNAs w/seed GAGUUGG) | 2.230 | TargetScan Human | Moderate (predicted) | FLII | FLII actin remodeling protein | Nucleus | other |
| MIMAT0003236 | miR-571 (miRNAs w/seed GAGUUGG) | 2.230 | TargetScan Human | Moderate (predicted) | FLOT1 | flotillin 1 | Plasma Membrane | other |
| MIMAT0003236 | miR-571 (miRNAs w/seed GAGUUGG) | 2.230 | TargetScan Human | Moderate (predicted) | FLT3LG | ms related receptor tyrosine kinase 3 ligan | Extracellular Space | cytokine |
| MIMAT0003236 | miR-571 (miRNAs w/seed GAGUUGG) | 2.230 | TargetScan Human | Moderate (predicted) | FLVCR2 | FLVCR heme transporter 2 | Plasma Membrane | transporter |
| MIMAT0003236 | miR-571 (miRNAs w/seed GAGUUGG) | 2.230 | TargetScan Human | Moderate (predicted) | FOSL1 | FOS like 1, AP-1 transcription factor subun | Nucleus | transcription regulator |
| MIMAT0003236 | miR-571 (miRNAs w/seed GAGUUGG) | 2.230 | TargetScan Human | Moderate (predicted) | FOXA3 | forkhead box A3 | Nucleus | transcription regulator |
| MIMAT0003236 | miR-571 (miRNAs w/seed GAGUUGG) | 2.230 | TargetScan Human | Moderate (predicted) | FSD2 | onectin type III and SPRY domain containi | Other | other |
| MIMAT0003236 | miR-571 (miRNAs w/seed GAGUUGG) | 2.230 | TargetScan Human | Moderate (predicted) | FUBP3 | far upstream element binding protein 3 | Nucleus | transcription regulator |
| MIMAT0003236 | miR-571 (miRNAs w/seed GAGUUGG) | 2.230 | TargetScan Human | Moderate (predicted) | FUT1 | fucosyltransferase 1 (H blood group) | Cytoplasm | enzyme |
| MIMAT0003236 | miR-571 (miRNAs w/seed GAGUUGG) | 2.230 | TargetScan Human | Moderate (predicted) | FXYD2 | YD domain containing ion transport regulat | Plasma Membrane | ion channel |
| MIMAT0003236 | miR-571 (miRNAs w/seed GAGUUGG) | 2.230 | TargetScan Human | Moderate (predicted) | FXYD6-FXYD2 | FXYD6-FXYD2 readthrough | Other | other |
| MIMAT0003236 | miR-571 (miRNAs w/seed GAGUUGG) | 2.230 | TargetScan Human | Moderate (predicted) | FYB1 | FYN binding protein 1 | Nucleus | other |
| MIMAT0003236 | miR-571 (miRNAs w/seed GAGUUGG) | 2.230 | TargetScan Human | Moderate (predicted) | FZD1 | frizzled class receptor 1 | Plasma Membrane | G-protein coupled receptor |
| MIMAT0003236 | miR-571 (miRNAs w/seed GAGUUGG) | 2.230 | TargetScan Human | Moderate (predicted) | G6PC2 | glucose-6-phosphatase catalytic subunit 2 | Cytoplasm | phosphatase |
| MIMAT0003236 | miR-571 (miRNAs w/seed GAGUUGG) | 2.230 | TargetScan Human | Moderate (predicted) | G6PD | glucose-6-phosphate dehydrogenase | Cytoplasm | enzyme |
| MIMAT0003236 | miR-571 (miRNAs w/seed GAGUUGG) | 2.230 | TargetScan Human | Moderate (predicted) | GALNT9 | ypeptide N-acetylgalactosaminyltransferas | Cytoplasm | enzyme |
| MIMAT0003236 | miR-571 (miRNAs w/seed GAGUUGG) | 2.230 | TargetScan Human | Moderate (predicted) | GANAB | glucosidase II alpha subunit | Cytoplasm | enzyme |
| MIMAT0003236 | miR-571 (miRNAs w/seed GAGUUGG) | 2.230 | TargetScan Human | Moderate (predicted) | GAS1 | growth arrest specific 1 | Plasma Membrane | other |
| MIMAT0003236 | miR-571 (miRNAs w/seed GAGUUGG) | 2.230 | TargetScan Human | Moderate (predicted) | GAS2L2 | growth arrest specific 2 like 2 | Cytoplasm | other |
| MIMAT0003236 | miR-571 (miRNAs w/seed GAGUUGG) | 2.230 | TargetScan Human | Moderate (predicted) | GATC | glutamyl-tRNA amidotransferase subunit C | Nucleus | enzyme |
| MIMAT0003236 | miR-571 (miRNAs w/seed GAGUUGG) | 2.230 | TargetScan Human | Moderate (predicted) | GBP4 | guanylate binding protein 4 | Cytoplasm | enzyme |
| MIMAT0003236 | miR-571 (miRNAs w/seed GAGUUGG) | 2.230 | TargetScan Human | Moderate (predicted) | GGPS1 | geranylgeranyl diphosphate synthase 1 | Cytoplasm | enzyme |
| MIMAT0003236 | miR-571 (miRNAs w/seed GAGUUGG) | 2.230 | TargetScan Human | Moderate (predicted) | GINS2 | GINS complex subunit 2 | Nucleus | other |
| MIMAT0003236 | miR-571 (miRNAs w/seed GAGUUGG) | 2.230 | TargetScan Human | High (predicted) | GINS4 | GINS complex subunit 4 | Nucleus | other |
| MIMAT0003236 | miR-571 (miRNAs w/seed GAGUUGG) | 2.230 | TargetScan Human | Moderate (predicted) | GIPC3 | PC PDZ domain containing family membe | Other | other |
| MIMAT0003236 | miR-571 (miRNAs w/seed GAGUUGG) | 2.230 | TargetScan Human | Moderate (predicted) | GJB5 | gap junction protein beta 5 | Plasma Membrane | transporter |
| MIMAT0003236 | miR-571 (miRNAs w/seed GAGUUGG) | 2.230 | TargetScan Human | Moderate (predicted) | GLG1 | golgi glycoprotein 1 | Cytoplasm | other |
| MIMAT0003236 | miR-571 (miRNAs w/seed GAGUUGG) | 2.230 | TargetScan Human | Moderate (predicted) | GLUL | glutamate-ammonia ligase | Cytoplasm | enzyme |
| MIMAT0003236 | miR-571 (miRNAs w/seed GAGUUGG) | 2.230 | TargetScan Human | Moderate (predicted) | GMNN | geminin DNA replication inhibitor | Nucleus | transcription regulator |
| MIMAT0003236 | miR-571 (miRNAs w/seed GAGUUGG) | 2.230 | TargetScan Human | High (predicted) | GNB2 | G protein subunit beta 2 | Plasma Membrane | enzyme |
| MIMAT0003236 | miR-571 (miRNAs w/seed GAGUUGG) | 2.230 | TargetScan Human | High (predicted) | GNPDA2 | glucosamine-6-phosphate deaminase 2 | Nucleus | enzyme |
| MIMAT0003236 | miR-571 (miRNAs w/seed GAGUUGG) | 2.230 | TargetScan Human | Moderate (predicted) | GNS | glucosamine (N-acetyl)-6-sulfatase | Cytoplasm | enzyme |
| MIMAT0003236 | miR-571 (miRNAs w/seed GAGUUGG) | 2.230 | TargetScan Human | Moderate (predicted) | GOLGB1 | golgin B1 | Cytoplasm | other |
| MIMAT0003236 | miR-571 (miRNAs w/seed GAGUUGG) | 2.230 | TargetScan Human | Moderate (predicted) | GPAT3 | glycerol-3-phosphate acyltransferase 3 | Cytoplasm | enzyme |
| MIMAT0003236 | miR-571 (miRNAs w/seed GAGUUGG) | 2.230 | TargetScan Human | High (predicted) | GPC6 | glypican 6 | Plasma Membrane | transmembrane receptor |
| MIMAT0003236 | miR-571 (miRNAs w/seed GAGUUGG) | 2.230 | TargetScan Human | Moderate (predicted) | GPR171 | G protein-coupled receptor 171 | Plasma Membrane | G-protein coupled receptor |
| MIMAT0003236 | miR-571 (miRNAs w/seed GAGUUGG) | 2.230 | TargetScan Human | Moderate (predicted) | GPR183 | G protein-coupled receptor 183 | Plasma Membrane | G-protein coupled receptor |
| MIMAT0003236 | miR-571 (miRNAs w/seed GAGUUGG) | 2.230 | TargetScan Human | Moderate (predicted) | GPR26 | G protein-coupled receptor 26 | Plasma Membrane | G-protein coupled receptor |
| MIMAT0003236 | miR-571 (miRNAs w/seed GAGUUGG) | 2.230 | TargetScan Human | High (predicted) | GPR3 | G protein-coupled receptor 3 | Plasma Membrane | G-protein coupled receptor |
| MIMAT0003236 | miR-571 (miRNAs w/seed GAGUUGG) | 2.230 | TargetScan Human | Moderate (predicted) | GPR31 | G protein-coupled receptor 31 | Plasma Membrane | G-protein coupled receptor |
| MIMAT0003236 | miR-571 (miRNAs w/seed GAGUUGG) | 2.230 | TargetScan Human | Moderate (predicted) | GPRASP1 | ein-coupled receptor associated sorting pr | Cytoplasm | transporter |
| MIMAT0003236 | miR-571 (miRNAs w/seed GAGUUGG) | 2.230 | TargetScan Human | Moderate (predicted) | GPX6 | glutathione peroxidase 6 | Extracellular Space | enzyme |
| MIMAT0003236 | miR-571 (miRNAs w/seed GAGUUGG) | 2.230 | TargetScan Human | Moderate (predicted) | GREM1 | gremlin 1, DAN family BMP antagonist | Extracellular Space | other |
| MIMAT0003236 | miR-571 (miRNAs w/seed GAGUUGG) | 2.230 | TargetScan Human | Moderate (predicted) | GRIA1 | amate ionotropic receptor AMPA type subu | Plasma Membrane | ion channel |
| MIMAT0003236 | miR-571 (miRNAs w/seed GAGUUGG) | 2.230 | TargetScan Human | Moderate (predicted) | GRIPAP1 | GRIP1 associated protein 1 | Plasma Membrane | other |
| MIMAT0003236 | miR-571 (miRNAs w/seed GAGUUGG) | 2.230 | TargetScan Human | High (predicted) | GRK4 | G protein-coupled receptor kinase 4 | Plasma Membrane | kinase |
| MIMAT0003236 | miR-571 (miRNAs w/seed GAGUUGG) | 2.230 | TargetScan Human | Moderate (predicted) | GSX2 | GS homeobox 2 | Nucleus | transcription regulator |
| MIMAT0003236 | miR-571 (miRNAs w/seed GAGUUGG) | 2.230 | TargetScan Human | Moderate (predicted) | GTDC1 | lycosyltransferase like domain containing | Other | other |
| MIMAT0003236 | miR-571 (miRNAs w/seed GAGUUGG) | 2.230 | TargetScan Human | Moderate (predicted) | GTF2H1 | general transcription factor IIH subunit 1 | Nucleus | transcription regulator |
| MIMAT0003236 | miR-571 (miRNAs w/seed GAGUUGG) | 2.230 | TargetScan Human | Moderate (predicted) | GTF3C2 | general transcription factor IIIC subunit 2 | Nucleus | transcription regulator |
| MIMAT0003236 | miR-571 (miRNAs w/seed GAGUUGG) | 2.230 | TargetScan Human | Moderate (predicted) | GYS2 | glycogen synthase 2 | Cytoplasm | enzyme |
| MIMAT0003236 | miR-571 (miRNAs w/seed GAGUUGG) | 2.230 | TargetScan Human | Moderate (predicted) | H2AC13 | H2A clustered histone 13 | Nucleus | other |
| MIMAT0003236 | miR-571 (miRNAs w/seed GAGUUGG) | 2.230 | TargetScan Human | Moderate (predicted) | H2BC18 | H2B clustered histone 18 | Nucleus | other |
| MIMAT0003236 | miR-571 (miRNAs w/seed GAGUUGG) | 2.230 | TargetScan Human | High (predicted) | H2BC5 | H2B clustered histone 5 | Nucleus | other |
| MIMAT0003236 | miR-571 (miRNAs w/seed GAGUUGG) | 2.230 | TargetScan Human | Moderate (predicted) | HCAR1 | hydroxycarboxylic acid receptor 1 | Plasma Membrane | G-protein coupled receptor |
| MIMAT0003236 | miR-571 (miRNAs w/seed GAGUUGG) | 2.230 | TargetScan Human | High (predicted) | HDHD2 | l dehalogenase like hydrolase domain con | Cytoplasm | other |
| MIMAT0003236 | miR-571 (miRNAs w/seed GAGUUGG) | 2.230 | TargetScan Human | Moderate (predicted) | HECTD3 | HECT domain E3 ubiquitin protein ligase 3 | Cytoplasm | enzyme |
| MIMAT0003236 | miR-571 (miRNAs w/seed GAGUUGG) | 2.230 | TargetScan Human | Moderate (predicted) | HEYL | family bHLH transcription factor with YRP | Nucleus | transcription regulator |
| MIMAT0003236 | miR-571 (miRNAs w/seed GAGUUGG) | 2.230 | TargetScan Human | Moderate (predicted) | HHAT | hedgehog acyltransferase | Cytoplasm | enzyme |
| MIMAT0003236 | miR-571 (miRNAs w/seed GAGUUGG) | 2.230 | TargetScan Human | High (predicted) | HHLA2 | HERV-H LTR-associating 2 | Other | other |
| MIMAT0003236 | miR-571 (miRNAs w/seed GAGUUGG) | 2.230 | TargetScan Human | Moderate (predicted) | HIBADH | 3-hydroxyisobutyrate dehydrogenase | Cytoplasm | enzyme |
| MIMAT0003236 | miR-571 (miRNAs w/seed GAGUUGG) | 2.230 | TargetScan Human | Moderate (predicted) | HIGD1B | 1 hypoxia inducible domain family membe | Other | other |
| MIMAT0003236 | miR-571 (miRNAs w/seed GAGUUGG) | 2.230 | TargetScan Human | Moderate (predicted) | HIP1 | huntingtin interacting protein 1 | Cytoplasm | other |
| MIMAT0003236 | miR-571 (miRNAs w/seed GAGUUGG) | 2.230 | TargetScan Human | Moderate (predicted) | HMGA2 | high mobility group AT-hook 2 | Nucleus | enzyme |
| MIMAT0003236 | miR-571 (miRNAs w/seed GAGUUGG) | 2.230 | TargetScan Human | Moderate (predicted) | HOATZ | HOATZ cilia and flagella associated protei | Other | other |
| MIMAT0003236 | miR-571 (miRNAs w/seed GAGUUGG) | 2.230 | TargetScan Human | Moderate (predicted) | HOXB5 | homeobox B5 | Nucleus | transcription regulator |
| MIMAT0003236 | miR-571 (miRNAs w/seed GAGUUGG) | 2.230 | TargetScan Human | Moderate (predicted) | HOXC6 | homeobox C6 | Nucleus | transcription regulator |
| MIMAT0003236 | miR-571 (miRNAs w/seed GAGUUGG) | 2.230 | TargetScan Human | High (predicted) | HOXC8 | homeobox C8 | Nucleus | transcription regulator |
| MIMAT0003236 | miR-571 (miRNAs w/seed GAGUUGG) | 2.230 | TargetScan Human | Moderate (predicted) | HRH3 | histamine receptor H3 | Plasma Membrane | G-protein coupled receptor |

Sup. Table 3. List of 3699 targets of the seven dysregulated miRNAs obtained from three different target prediction databases,TargetScan TarBase and miRecords considering those predicted as moderate, high, or experimentally validated, respectively.

| | | | | | | | | |
|---|---|---|---|---|---|---|---|---|
| MIMAT0003236 | miR-571 (miRNAs w/seed GAGUUGG) | 2.230 | TargetScan Human | High (predicted) | HS3ST1 | aran sulfate-glucosamine 3-sulfotransfera | Cytoplasm | enzyme |
| MIMAT0003236 | miR-571 (miRNAs w/seed GAGUUGG) | 2.230 | TargetScan Human | Moderate (predicted) | HS6ST3 | heparan sulfate 6-O-sulfotransferase 3 | Other | enzyme |
| MIMAT0003236 | miR-571 (miRNAs w/seed GAGUUGG) | 2.230 | TargetScan Human | Moderate (predicted) | HSD11B1L | ydroxysteroid 11-beta dehydrogenase 1 lil | Nucleus | other |
| MIMAT0003236 | miR-571 (miRNAs w/seed GAGUUGG) | 2.230 | TargetScan Human | High (predicted) | HSPB6 | eat shock protein family B (small) member | Cytoplasm | other |
| MIMAT0003236 | miR-571 (miRNAs w/seed GAGUUGG) | 2.230 | TargetScan Human | Moderate (predicted) | HTR3A | 5-hydroxytryptamine receptor 3A | Plasma Membrane | ion channel |
| MIMAT0003236 | miR-571 (miRNAs w/seed GAGUUGG) | 2.230 | TargetScan Human | Moderate (predicted) | HUWE1 | nd WWE domain containing E3 ubiquitin p | Nucleus | transcription regulator |
| MIMAT0003236 | miR-571 (miRNAs w/seed GAGUUGG) | 2.230 | TargetScan Human | Moderate (predicted) | IFI6 | interferon alpha inducible protein 6 | Cytoplasm | other |
| MIMAT0003236 | miR-571 (miRNAs w/seed GAGUUGG) | 2.230 | TargetScan Human | Moderate (predicted) | IGHMBP2 | immunoglobulin mu DNA binding protein 2 | Nucleus | enzyme |
| MIMAT0003236 | miR-571 (miRNAs w/seed GAGUUGG) | 2.230 | TargetScan Human | Moderate (predicted) | IGLL1/IGLL5 | immunoglobulin lambda like polypeptide 1 | Plasma Membrane | other |
| MIMAT0003236 | miR-571 (miRNAs w/seed GAGUUGG) | 2.230 | TargetScan Human | High (predicted) | IGSF6 | immunoglobulin superfamily member 6 | Plasma Membrane | transmembrane receptor |
| MIMAT0003236 | miR-571 (miRNAs w/seed GAGUUGG) | 2.230 | TargetScan Human | Moderate (predicted) | IHH | Indian hedgehog signaling molecule | Extracellular Space | enzyme |
| MIMAT0003236 | miR-571 (miRNAs w/seed GAGUUGG) | 2.230 | TargetScan Human | Moderate (predicted) | IL2RB | interleukin 2 receptor subunit beta | Plasma Membrane | transmembrane receptor |
| MIMAT0003236 | miR-571 (miRNAs w/seed GAGUUGG) | 2.230 | TargetScan Human | Moderate (predicted) | IL6R | interleukin 6 receptor | Plasma Membrane | transmembrane receptor |
| MIMAT0003236 | miR-571 (miRNAs w/seed GAGUUGG) | 2.230 | TargetScan Human | Moderate (predicted) | ILF2 | interleukin enhancer binding factor 2 | Nucleus | transcription regulator |
| MIMAT0003236 | miR-571 (miRNAs w/seed GAGUUGG) | 2.230 | TargetScan Human | Moderate (predicted) | INIP | INTS3 and NABP interacting protein | Nucleus | other |
| MIMAT0003236 | miR-571 (miRNAs w/seed GAGUUGG) | 2.230 | TargetScan Human | Moderate (predicted) | INKA2 | inka box actin regulator 2 | Nucleus | other |
| MIMAT0003236 | miR-571 (miRNAs w/seed GAGUUGG) | 2.230 | TargetScan Human | Moderate (predicted) | INTS6L | integrator complex subunit 6 like | Nucleus | other |
| MIMAT0003236 | miR-571 (miRNAs w/seed GAGUUGG) | 2.230 | TargetScan Human | Moderate (predicted) | IRF9 | interferon regulatory factor 9 | Nucleus | transcription regulator |
| MIMAT0003236 | miR-571 (miRNAs w/seed GAGUUGG) | 2.230 | TargetScan Human | Moderate (predicted) | ITGA6 | integrin subunit alpha 6 | Plasma Membrane | transmembrane receptor |
| MIMAT0003236 | miR-571 (miRNAs w/seed GAGUUGG) | 2.230 | TargetScan Human | Moderate (predicted) | IYD | iodotyrosine deiodinase | Plasma Membrane | enzyme |
| MIMAT0003236 | miR-571 (miRNAs w/seed GAGUUGG) | 2.230 | TargetScan Human | Moderate (predicted) | JCHAIN | joining chain of multimeric IgA and IgM | Extracellular Space | other |
| MIMAT0003236 | miR-571 (miRNAs w/seed GAGUUGG) | 2.230 | TargetScan Human | Moderate (predicted) | KAT5 | lysine acetyltransferase 5 | Nucleus | transcription regulator |
| MIMAT0003236 | miR-571 (miRNAs w/seed GAGUUGG) | 2.230 | TargetScan Human | Moderate (predicted) | KCNB1 | um voltage-gated channel subfamily B me | Plasma Membrane | ion channel |
| MIMAT0003236 | miR-571 (miRNAs w/seed GAGUUGG) | 2.230 | TargetScan Human | Moderate (predicted) | KCNE5 | oltage-gated channel subfamily E regulato | Plasma Membrane | ion channel |
| MIMAT0003236 | miR-571 (miRNAs w/seed GAGUUGG) | 2.230 | TargetScan Human | Moderate (predicted) | KCNH6 | um voltage-gated channel subfamily H me | Plasma Membrane | ion channel |
| MIMAT0003236 | miR-571 (miRNAs w/seed GAGUUGG) | 2.230 | TargetScan Human | High (predicted) | KCNH7 | um voltage-gated channel subfamily H me | Plasma Membrane | ion channel |
| MIMAT0003236 | miR-571 (miRNAs w/seed GAGUUGG) | 2.230 | TargetScan Human | High (predicted) | KCNIP1 | sium voltage-gated channel interacting pro | Plasma Membrane | ion channel |
| MIMAT0003236 | miR-571 (miRNAs w/seed GAGUUGG) | 2.230 | TargetScan Human | Moderate (predicted) | KCNJ10 | n inwardly rectifying channel subfamily J m | Plasma Membrane | ion channel |
| MIMAT0003236 | miR-571 (miRNAs w/seed GAGUUGG) | 2.230 | TargetScan Human | Moderate (predicted) | KCNJ11 | n inwardly rectifying channel subfamily J m | Plasma Membrane | ion channel |
| MIMAT0003236 | miR-571 (miRNAs w/seed GAGUUGG) | 2.230 | TargetScan Human | Moderate (predicted) | KCNJ13 | n inwardly rectifying channel subfamily J m | Plasma Membrane | ion channel |
| MIMAT0003236 | miR-571 (miRNAs w/seed GAGUUGG) | 2.230 | TargetScan Human | Moderate (predicted) | KCNN3 | n calcium-activated channel subfamily N r | Plasma Membrane | ion channel |
| MIMAT0003236 | miR-571 (miRNAs w/seed GAGUUGG) | 2.230 | TargetScan Human | Moderate (predicted) | KIAA0232 | KIAA0232 | Extracellular Space | other |
| MIMAT0003236 | miR-571 (miRNAs w/seed GAGUUGG) | 2.230 | TargetScan Human | Moderate (predicted) | KIAA0408 | KIAA0408 | Other | other |
| MIMAT0003236 | miR-571 (miRNAs w/seed GAGUUGG) | 2.230 | TargetScan Human | High (predicted) | KIAA1143 | KIAA1143 | Other | other |
| MIMAT0003236 | miR-571 (miRNAs w/seed GAGUUGG) | 2.230 | TargetScan Human | Moderate (predicted) | KLHDC10 | kelch domain containing 10 | Nucleus | other |
| MIMAT0003236 | miR-571 (miRNAs w/seed GAGUUGG) | 2.230 | TargetScan Human | High (predicted) | KLHDC2 | kelch domain containing 2 | Cytoplasm | other |
| MIMAT0003236 | miR-571 (miRNAs w/seed GAGUUGG) | 2.230 | TargetScan Human | Moderate (predicted) | KLHL7 | kelch like family member 7 | Nucleus | other |
| MIMAT0003236 | miR-571 (miRNAs w/seed GAGUUGG) | 2.230 | TargetScan Human | Moderate (predicted) | KRT222 | keratin 222 | Other | other |
| MIMAT0003236 | miR-571 (miRNAs w/seed GAGUUGG) | 2.230 | TargetScan Human | Moderate (predicted) | KRT72 | keratin 72 | Cytoplasm | other |
| MIMAT0003236 | miR-571 (miRNAs w/seed GAGUUGG) | 2.230 | TargetScan Human | Moderate (predicted) | KRTAP4-6 | keratin associated protein 4-6 | Cytoplasm | other |
| MIMAT0003236 | miR-571 (miRNAs w/seed GAGUUGG) | 2.230 | TargetScan Human | High (predicted) | KRTAP9-6 | keratin associated protein 9-6 | Cytoplasm | other |
| MIMAT0003236 | miR-571 (miRNAs w/seed GAGUUGG) | 2.230 | TargetScan Human | High (predicted) | KTN1 | kinectin 1 | Plasma Membrane | transmembrane receptor |
| MIMAT0003236 | miR-571 (miRNAs w/seed GAGUUGG) | 2.230 | TargetScan Human | Moderate (predicted) | KYAT3 | kynurenine aminotransferase 3 | Cytoplasm | enzyme |
| MIMAT0003236 | miR-571 (miRNAs w/seed GAGUUGG) | 2.230 | TargetScan Human | High (predicted) | LAMA1 | laminin subunit alpha 1 | Extracellular Space | other |
| MIMAT0003236 | miR-571 (miRNAs w/seed GAGUUGG) | 2.230 | TargetScan Human | Moderate (predicted) | LAS1L | LAS1 like ribosome biogenesis factor | Nucleus | other |
| MIMAT0003236 | miR-571 (miRNAs w/seed GAGUUGG) | 2.230 | TargetScan Human | Moderate (predicted) | LBH | LBH regulator of WNT signaling pathway | Nucleus | transcription regulator |
| MIMAT0003236 | miR-571 (miRNAs w/seed GAGUUGG) | 2.230 | TargetScan Human | Moderate (predicted) | LDAH | lipid droplet associated hydrolase | Cytoplasm | enzyme |
| MIMAT0003236 | miR-571 (miRNAs w/seed GAGUUGG) | 2.230 | TargetScan Human | Moderate (predicted) | LDLRAD3 | ity lipoprotein receptor class A domain cor | Plasma Membrane | other |
| MIMAT0003236 | miR-571 (miRNAs w/seed GAGUUGG) | 2.230 | TargetScan Human | Moderate (predicted) | LETMD1 | LETM1 domain containing 1 | Plasma Membrane | other |
| MIMAT0003236 | miR-571 (miRNAs w/seed GAGUUGG) | 2.230 | TargetScan Human | Moderate (predicted) | LIMK2 | LIM domain kinase 2 | Cytoplasm | kinase |
| MIMAT0003236 | miR-571 (miRNAs w/seed GAGUUGG) | 2.230 | TargetScan Human | High (predicted) | LIN52 | -52 DREAM MuvB core complex compone | Nucleus | other |
| MIMAT0003236 | miR-571 (miRNAs w/seed GAGUUGG) | 2.230 | TargetScan Human | High (predicted) | LINC01555 | ng intergenic non-protein coding RNA 155 | Other | other |
| MIMAT0003236 | miR-571 (miRNAs w/seed GAGUUGG) | 2.230 | TargetScan Human | Moderate (predicted) | LINC02873 | ng intergenic non-protein coding RNA 287 | Other | other |
| MIMAT0003236 | miR-571 (miRNAs w/seed GAGUUGG) | 2.230 | TargetScan Human | Moderate (predicted) | LINC02897 | ng intergenic non-protein coding RNA 289 | Other | other |
| MIMAT0003236 | miR-571 (miRNAs w/seed GAGUUGG) | 2.230 | TargetScan Human | Moderate (predicted) | LMO3 | LIM domain only 3 | Nucleus | other |
| MIMAT0003236 | miR-571 (miRNAs w/seed GAGUUGG) | 2.230 | TargetScan Human | Moderate (predicted) | LMX1A | LIM homeobox transcription factor 1 alpha | Nucleus | transcription regulator |
| MIMAT0003236 | miR-571 (miRNAs w/seed GAGUUGG) | 2.230 | TargetScan Human | Moderate (predicted) | LNP1 | leukemia NUP98 fusion partner 1 | Other | other |
| MIMAT0003236 | miR-571 (miRNAs w/seed GAGUUGG) | 2.230 | TargetScan Human | Moderate (predicted) | LOC100131635 | hCG1645011-like | Other | other |
| MIMAT0003236 | miR-571 (miRNAs w/seed GAGUUGG) | 2.230 | TargetScan Human | High (predicted) | LOC150935 | uncharacterized LOC150935 | Other | other |
| MIMAT0003236 | miR-571 (miRNAs w/seed GAGUUGG) | 2.230 | TargetScan Human | High (predicted) | LOC401589 | uncharacterized LOC401589 | Other | other |
| MIMAT0003236 | miR-571 (miRNAs w/seed GAGUUGG) | 2.230 | TargetScan Human | Moderate (predicted) | LOH12CR2 | of heterozygosity on chromosome 12, reg | Other | other |
| MIMAT0003236 | miR-571 (miRNAs w/seed GAGUUGG) | 2.230 | TargetScan Human | Moderate (predicted) | LOXL4 | lysyl oxidase like 4 | Extracellular Space | enzyme |
| MIMAT0003236 | miR-571 (miRNAs w/seed GAGUUGG) | 2.230 | TargetScan Human | Moderate (predicted) | LPAR1 | lysophosphatidic acid receptor 1 | Plasma Membrane | G-protein coupled receptor |
| MIMAT0003236 | miR-571 (miRNAs w/seed GAGUUGG) | 2.230 | TargetScan Human | Moderate (predicted) | LPGAT1 | lysophosphatidylglycerol acyltransferase 1 | Cytoplasm | enzyme |
| MIMAT0003236 | miR-571 (miRNAs w/seed GAGUUGG) | 2.230 | TargetScan Human | Moderate (predicted) | LRRC20 | leucine rich repeat containing 20 | Other | other |
| MIMAT0003236 | miR-571 (miRNAs w/seed GAGUUGG) | 2.230 | TargetScan Human | Moderate (predicted) | LRRC3 | leucine rich repeat containing 3 | Other | other |
| MIMAT0003236 | miR-571 (miRNAs w/seed GAGUUGG) | 2.230 | TargetScan Human | Moderate (predicted) | LRRC32 | leucine rich repeat containing 32 | Plasma Membrane | other |
| MIMAT0003236 | miR-571 (miRNAs w/seed GAGUUGG) | 2.230 | TargetScan Human | Moderate (predicted) | LRRD1 | ine rich repeats and death domain contain | Other | other |
| MIMAT0003236 | miR-571 (miRNAs w/seed GAGUUGG) | 2.230 | TargetScan Human | Moderate (predicted) | LSG1 | arge 60S subunit nuclear export GTPase | Cytoplasm | enzyme |
| MIMAT0003236 | miR-571 (miRNAs w/seed GAGUUGG) | 2.230 | TargetScan Human | Moderate (predicted) | LSM10 | LSM10, U7 small nuclear RNA associated | Nucleus | other |
| MIMAT0003236 | miR-571 (miRNAs w/seed GAGUUGG) | 2.230 | TargetScan Human | Moderate (predicted) | LYAR | Ly1 antibody reactive | Plasma Membrane | other |
| MIMAT0003236 | miR-571 (miRNAs w/seed GAGUUGG) | 2.230 | TargetScan Human | High (predicted) | LYRM7 | LYR motif containing 7 | Cytoplasm | other |
| MIMAT0003236 | miR-571 (miRNAs w/seed GAGUUGG) | 2.230 | TargetScan Human | Moderate (predicted) | MAOA | monoamine oxidase A | Cytoplasm | enzyme |
| MIMAT0003236 | miR-571 (miRNAs w/seed GAGUUGG) | 2.230 | TargetScan Human | Moderate (predicted) | MAOB | monoamine oxidase B | Cytoplasm | enzyme |
| MIMAT0003236 | miR-571 (miRNAs w/seed GAGUUGG) | 2.230 | TargetScan Human | Moderate (predicted) | MAP1A | microtubule associated protein 1A | Cytoplasm | other |
| MIMAT0003236 | miR-571 (miRNAs w/seed GAGUUGG) | 2.230 | TargetScan Human | Moderate (predicted) | MAPK12 | mitogen-activated protein kinase 12 | Cytoplasm | kinase |
| MIMAT0003236 | miR-571 (miRNAs w/seed GAGUUGG) | 2.230 | TargetScan Human | High (predicted) | MARCHF4 | nembrane associated ring-CH-type finger | Cytoplasm | enzyme |
| MIMAT0003236 | miR-571 (miRNAs w/seed GAGUUGG) | 2.230 | TargetScan Human | Moderate (predicted) | MASP2 | MBL associated serine protease 2 | Extracellular Space | peptidase |
| MIMAT0003236 | miR-571 (miRNAs w/seed GAGUUGG) | 2.230 | TargetScan Human | Moderate (predicted) | MAZ | MYC associated zinc finger protein | Nucleus | transcription regulator |
| MIMAT0003236 | miR-571 (miRNAs w/seed GAGUUGG) | 2.230 | TargetScan Human | Moderate (predicted) | MDM2 | MDM2 proto-oncogene | Nucleus | transcription regulator |
| MIMAT0003236 | miR-571 (miRNAs w/seed GAGUUGG) | 2.230 | TargetScan Human | Moderate (predicted) | MEMO1 | mediator of cell motility 1 | Cytoplasm | other |
| MIMAT0003236 | miR-571 (miRNAs w/seed GAGUUGG) | 2.230 | TargetScan Human | High (predicted) | METTL9 | methyltransferase like 9 | Cytoplasm | enzyme |
| MIMAT0003236 | miR-571 (miRNAs w/seed GAGUUGG) | 2.230 | TargetScan Human | Moderate (predicted) | MEX3C | mex-3 RNA binding family member C | Cytoplasm | enzyme |
| MIMAT0003236 | miR-571 (miRNAs w/seed GAGUUGG) | 2.230 | TargetScan Human | Moderate (predicted) | MFAP4 | microfibril associated protein 4 | Extracellular Space | other |
| MIMAT0003236 | miR-571 (miRNAs w/seed GAGUUGG) | 2.230 | TargetScan Human | Moderate (predicted) | MFSD14B | or facilitator superfamily domain containing | Other | other |
| MIMAT0003236 | miR-571 (miRNAs w/seed GAGUUGG) | 2.230 | TargetScan Human | Moderate (predicted) | MFSD6 | jor facilitator superfamily domain containin | Plasma Membrane | transmembrane receptor |
| MIMAT0003236 | miR-571 (miRNAs w/seed GAGUUGG) | 2.230 | TargetScan Human | Moderate (predicted) | MGST1 | microsomal glutathione S-transferase 1 | Cytoplasm | enzyme |
| MIMAT0003236 | miR-571 (miRNAs w/seed GAGUUGG) | 2.230 | TargetScan Human | Moderate (predicted) | MICAL2 | ated monooxygenase, calponin and LIM d | Cytoplasm | enzyme |
| MIMAT0003236 | miR-571 (miRNAs w/seed GAGUUGG) | 2.230 | TargetScan Human | High (predicted) | MICB | MHC class I polypeptide-related sequence | Plasma Membrane | transmembrane receptor |
| MIMAT0003236 | miR-571 (miRNAs w/seed GAGUUGG) | 2.230 | TargetScan Human | Moderate (predicted) | MIEF1 | mitochondrial elongation factor 1 | Cytoplasm | other |
| MIMAT0003236 | miR-571 (miRNAs w/seed GAGUUGG) | 2.230 | TargetScan Human | High (predicted) | MMP19 | matrix metallopeptidase 19 | Extracellular Space | peptidase |
| MIMAT0003236 | miR-571 (miRNAs w/seed GAGUUGG) | 2.230 | TargetScan Human | Moderate (predicted) | MOCS1 | molybdenum cofactor synthesis 1 | Cytoplasm | other |
| MIMAT0003236 | miR-571 (miRNAs w/seed GAGUUGG) | 2.230 | TargetScan Human | Moderate (predicted) | MORC3 | MORC family CW-type zinc finger 3 | Nucleus | other |
| MIMAT0003236 | miR-571 (miRNAs w/seed GAGUUGG) | 2.230 | TargetScan Human | Moderate (predicted) | MPC1 | mitochondrial pyruvate carrier 1 | Cytoplasm | transporter |
| MIMAT0003236 | miR-571 (miRNAs w/seed GAGUUGG) | 2.230 | TargetScan Human | Moderate (predicted) | MPI | mannose phosphate isomerase | Cytoplasm | enzyme |
| MIMAT0003236 | miR-571 (miRNAs w/seed GAGUUGG) | 2.230 | TargetScan Human | Moderate (predicted) | MRGPRX2 | MAS related GPR family member X2 | Plasma Membrane | G-protein coupled receptor |
| MIMAT0003236 | miR-571 (miRNAs w/seed GAGUUGG) | 2.230 | TargetScan Human | Moderate (predicted) | MRPL11 | mitochondrial ribosomal protein L11 | Cytoplasm | other |
| MIMAT0003236 | miR-571 (miRNAs w/seed GAGUUGG) | 2.230 | TargetScan Human | Moderate (predicted) | MRPL35 | mitochondrial ribosomal protein L35 | Cytoplasm | other |
| MIMAT0003236 | miR-571 (miRNAs w/seed GAGUUGG) | 2.230 | TargetScan Human | High (predicted) | MRPS18B | mitochondrial ribosomal protein S18B | Cytoplasm | other |
| MIMAT0003236 | miR-571 (miRNAs w/seed GAGUUGG) | 2.230 | TargetScan Human | Moderate (predicted) | MRPS34 | mitochondrial ribosomal protein S34 | Cytoplasm | other |
| MIMAT0003236 | miR-571 (miRNAs w/seed GAGUUGG) | 2.230 | TargetScan Human | Moderate (predicted) | MTMR11 | myotubularin related protein 11 | Cytoplasm | other |
| MIMAT0003236 | miR-571 (miRNAs w/seed GAGUUGG) | 2.230 | TargetScan Human | Moderate (predicted) | MTNR1B | melatonin receptor 1B | Plasma Membrane | G-protein coupled receptor |
| MIMAT0003236 | miR-571 (miRNAs w/seed GAGUUGG) | 2.230 | TargetScan Human | Moderate (predicted) | MTPN | myotrophin | Nucleus | transcription regulator |
| MIMAT0003236 | miR-571 (miRNAs w/seed GAGUUGG) | 2.230 | TargetScan Human | Moderate (predicted) | MYADML2 | yeloid associated differentiation marker like | Cytoplasm | other |
| MIMAT0003236 | miR-571 (miRNAs w/seed GAGUUGG) | 2.230 | TargetScan Human | Moderate (predicted) | MYEOV | myeloma overexpressed | Nucleus | other |
| MIMAT0003236 | miR-571 (miRNAs w/seed GAGUUGG) | 2.230 | TargetScan Human | High (predicted) | MYOZ1 | myozenin 1 | Cytoplasm | other |
| MIMAT0003236 | miR-571 (miRNAs w/seed GAGUUGG) | 2.230 | TargetScan Human | High (predicted) | NAA35 | pha-acetyltransferase 35, NatC auxiliary su | Cytoplasm | other |
| MIMAT0003236 | miR-571 (miRNAs w/seed GAGUUGG) | 2.230 | TargetScan Human | High (predicted) | NAALADL1 | cetylated alpha-linked acidic dipeptidase li | Plasma Membrane | peptidase |
| MIMAT0003236 | miR-571 (miRNAs w/seed GAGUUGG) | 2.230 | TargetScan Human | High (predicted) | NAT2 | N-acetyltransferase 2 | Cytoplasm | enzyme |
| MIMAT0003236 | miR-571 (miRNAs w/seed GAGUUGG) | 2.230 | TargetScan Human | Moderate (predicted) | NBPF4/NBPF6 | NBPF member 6 | Other | other |
| MIMAT0003236 | miR-571 (miRNAs w/seed GAGUUGG) | 2.230 | TargetScan Human | High (predicted) | NDUFB5 | ADH:ubiquinone oxidoreductase subunit B | Cytoplasm | enzyme |
| MIMAT0003236 | miR-571 (miRNAs w/seed GAGUUGG) | 2.230 | TargetScan Human | High (predicted) | NECAB1 | -terminal EF-hand calcium binding protein | Cytoplasm | other |
| MIMAT0003236 | miR-571 (miRNAs w/seed GAGUUGG) | 2.230 | TargetScan Human | Moderate (predicted) | NFIC | nuclear factor I C | Nucleus | transcription regulator |
| MIMAT0003236 | miR-571 (miRNAs w/seed GAGUUGG) | 2.230 | TargetScan Human | Moderate (predicted) | NHLH1 | nescient helix-loop-helix 1 | Nucleus | transcription regulator |
| MIMAT0003236 | miR-571 (miRNAs w/seed GAGUUGG) | 2.230 | TargetScan Human | High (predicted) | NIPAL2 | NIPA like domain containing 2 | Other | other |
| MIMAT0003236 | miR-571 (miRNAs w/seed GAGUUGG) | 2.230 | TargetScan Human | Moderate (predicted) | NKAIN1 | um/potassium transporting ATPase interac | Plasma Membrane | other |
| MIMAT0003236 | miR-571 (miRNAs w/seed GAGUUGG) | 2.230 | TargetScan Human | High (predicted) | NLGN3 | neuroligin 3 | Plasma Membrane | enzyme |
| MIMAT0003236 | miR-571 (miRNAs w/seed GAGUUGG) | 2.230 | TargetScan Human | Moderate (predicted) | NPAS3 | neuronal PAS domain protein 3 | Nucleus | transcription regulator |
| MIMAT0003236 | miR-571 (miRNAs w/seed GAGUUGG) | 2.230 | TargetScan Human | High (predicted) | NPIPA9 | pore complex interacting protein family, me | Other | other |
| MIMAT0003236 | miR-571 (miRNAs w/seed GAGUUGG) | 2.230 | TargetScan Human | Moderate (predicted) | NPL | N-acetylneuraminate pyruvate lyase | Cytoplasm | enzyme |
| MIMAT0003236 | miR-571 (miRNAs w/seed GAGUUGG) | 2.230 | TargetScan Human | Moderate (predicted) | NPM2 | nucleophosmin/nucleoplasmin 2 | Nucleus | other |
| MIMAT0003236 | miR-571 (miRNAs w/seed GAGUUGG) | 2.230 | TargetScan Human | High (predicted) | NRBP2 | nuclear receptor binding protein 2 | Cytoplasm | kinase |
| MIMAT0003236 | miR-571 (miRNAs w/seed GAGUUGG) | 2.230 | TargetScan Human | Moderate (predicted) | NRSN2 | neurensin 2 | Plasma Membrane | other |
| MIMAT0003236 | miR-571 (miRNAs w/seed GAGUUGG) | 2.230 | TargetScan Human | High (predicted) | NSUN4 | NOP2/Sun RNA methyltransferase 4 | Cytoplasm | enzyme |
| MIMAT0003236 | miR-571 (miRNAs w/seed GAGUUGG) | 2.230 | TargetScan Human | Moderate (predicted) | NSUN7 | 2/Sun RNA methyltransferase family mem | Other | other |
| MIMAT0003236 | miR-571 (miRNAs w/seed GAGUUGG) | 2.230 | TargetScan Human | Moderate (predicted) | NXF3 | nuclear RNA export factor 3 | Nucleus | transporter |
| MIMAT0003236 | miR-571 (miRNAs w/seed GAGUUGG) | 2.230 | TargetScan Human | Moderate (predicted) | NYAP2 | ine-phosphorylated phosphoinositide-3-ki | Cytoplasm | other |
| MIMAT0003236 | miR-571 (miRNAs w/seed GAGUUGG) | 2.230 | TargetScan Human | High (predicted) | OCIAD1 | OCIA domain containing 1 | Cytoplasm | other |
| MIMAT0003236 | miR-571 (miRNAs w/seed GAGUUGG) | 2.230 | TargetScan Human | High (predicted) | OIT3 | oncoprotein induced transcript 3 | Nucleus | other |
| MIMAT0003236 | miR-571 (miRNAs w/seed GAGUUGG) | 2.230 | TargetScan Human | Moderate (predicted) | OPALIN | ndrocytic myelin paranodal and inner loop | Cytoplasm | other |
| MIMAT0003236 | miR-571 (miRNAs w/seed GAGUUGG) | 2.230 | TargetScan Human | Moderate (predicted) | OSGEPL1 | O-sialoglycoprotein endopeptidase like 1 | Cytoplasm | peptidase |
| MIMAT0003236 | miR-571 (miRNAs w/seed GAGUUGG) | 2.230 | TargetScan Human | Moderate (predicted) | OTUD3 | OTU deubiquitinase 3 | Cytoplasm | peptidase |
| MIMAT0003236 | miR-571 (miRNAs w/seed GAGUUGG) | 2.230 | TargetScan Human | Moderate (predicted) | OTX1 | orthodenticle homeobox 1 | Nucleus | transcription regulator |
| MIMAT0003236 | miR-571 (miRNAs w/seed GAGUUGG) | 2.230 | TargetScan Human | Moderate (predicted) | PAK1 | p21 (RAC1) activated kinase 1 | Cytoplasm | kinase |
| MIMAT0003236 | miR-571 (miRNAs w/seed GAGUUGG) | 2.230 | TargetScan Human | Moderate (predicted) | PARP1 | poly(ADP-ribose) polymerase 1 | Nucleus | enzyme |
| MIMAT0003236 | miR-571 (miRNAs w/seed GAGUUGG) | 2.230 | TargetScan Human | Moderate (predicted) | PARS2 | prolyl-tRNA synthetase 2, mitochondrial | Cytoplasm | enzyme |
| MIMAT0003236 | miR-571 (miRNAs w/seed GAGUUGG) | 2.230 | TargetScan Human | Moderate (predicted) | PAX7 | paired box 7 | Nucleus | transcription regulator |
| MIMAT0003236 | miR-571 (miRNAs w/seed GAGUUGG) | 2.230 | TargetScan Human | High (predicted) | PAX8 | paired box 8 | Nucleus | transcription regulator |
| MIMAT0003236 | miR-571 (miRNAs w/seed GAGUUGG) | 2.230 | TargetScan Human | Moderate (predicted) | PCDH10 | protocadherin 10 | Plasma Membrane | other |
| MIMAT0003236 | miR-571 (miRNAs w/seed GAGUUGG) | 2.230 | TargetScan Human | Moderate (predicted) | PCNT | pericentrin | Cytoplasm | other |
| MIMAT0003236 | miR-571 (miRNAs w/seed GAGUUGG) | 2.230 | TargetScan Human | Moderate (predicted) | PCP4L1 | Purkinje cell protein 4 like 1 | Other | other |
| MIMAT0003236 | miR-571 (miRNAs w/seed GAGUUGG) | 2.230 | TargetScan Human | Moderate (predicted) | PDHA2 | yruvate dehydrogenase E1 subunit alpha | Nucleus | enzyme |
| MIMAT0003236 | miR-571 (miRNAs w/seed GAGUUGG) | 2.230 | TargetScan Human | Moderate (predicted) | PEG10 | paternally expressed 10 | Nucleus | other |
| MIMAT0003236 | miR-571 (miRNAs w/seed GAGUUGG) | 2.230 | TargetScan Human | Moderate (predicted) | PFKFB3 | sphofructo-2-kinase/fructose-2,6-biphosph | Cytoplasm | kinase |
| MIMAT0003236 | miR-571 (miRNAs w/seed GAGUUGG) | 2.230 | TargetScan Human | Moderate (predicted) | PGM3 | phosphoglucomutase 3 | Cytoplasm | enzyme |
| MIMAT0003236 | miR-571 (miRNAs w/seed GAGUUGG) | 2.230 | TargetScan Human | High (predicted) | PIANP | PILR alpha associated neural protein | Plasma Membrane | other |

Sup. Table 3. List of 3699 targets of the seven dysregulated miRNAs obtained from three different target prediction databases,TargetScan TarBase and miRecords considering those predicted as moderate, high, or experimentally validated, respectively.

| | | | | | | | | |
|---|---|---|---|---|---|---|---|---|
| MIMAT0003236 | miR-571 (miRNAs w/seed GAGUUGG) | 2.230 | TargetScan Human | Moderate (predicted) | PIK3R6 | osphoinositide-3-kinase regulatory subun | Cytoplasm | kinase |
| MIMAT0003236 | miR-571 (miRNAs w/seed GAGUUGG) | 2.230 | TargetScan Human | Moderate (predicted) | PIN4 | dylprolyl cis/trans isomerase, NIMA-interac | Nucleus | enzyme |
| MIMAT0003236 | miR-571 (miRNAs w/seed GAGUUGG) | 2.230 | TargetScan Human | Moderate (predicted) | PINX1 | N2 (TERF1) interacting telomerase inhibitc | Nucleus | other |
| MIMAT0003236 | miR-571 (miRNAs w/seed GAGUUGG) | 2.230 | TargetScan Human | Moderate (predicted) | PIP4K2A | hatidylinositol-5-phosphate 4-kinase type 2 | Cytoplasm | kinase |
| MIMAT0003236 | miR-571 (miRNAs w/seed GAGUUGG) | 2.230 | TargetScan Human | Moderate (predicted) | PIRT | interacting regulator of transient receptor | Plasma Membrane | other |
| MIMAT0003236 | miR-571 (miRNAs w/seed GAGUUGG) | 2.230 | TargetScan Human | Moderate (predicted) | PITX3 | paired like homeodomain 3 | Nucleus | transcription regulator |
| MIMAT0003236 | miR-571 (miRNAs w/seed GAGUUGG) | 2.230 | TargetScan Human | Moderate (predicted) | PKHD1 | ciliary IPT domain containing fibrocystin/p | Plasma Membrane | other |
| MIMAT0003236 | miR-571 (miRNAs w/seed GAGUUGG) | 2.230 | TargetScan Human | High (predicted) | PLA2G5 | phospholipase A2 group V | Extracellular Space | enzyme |
| MIMAT0003236 | miR-571 (miRNAs w/seed GAGUUGG) | 2.230 | TargetScan Human | Moderate (predicted) | PLD1 | phospholipase D1 | Cytoplasm | enzyme |
| MIMAT0003236 | miR-571 (miRNAs w/seed GAGUUGG) | 2.230 | TargetScan Human | Moderate (predicted) | PLEKHO1 | pleckstrin homology domain containing O1 | Plasma Membrane | other |
| MIMAT0003236 | miR-571 (miRNAs w/seed GAGUUGG) | 2.230 | TargetScan Human | Moderate (predicted) | PLP2 | proteolipid protein 2 | Cytoplasm | transporter |
| MIMAT0003236 | miR-571 (miRNAs w/seed GAGUUGG) | 2.230 | TargetScan Human | Moderate (predicted) | PLXDC1 | plexin domain containing 1 | Plasma Membrane | other |
| MIMAT0003236 | miR-571 (miRNAs w/seed GAGUUGG) | 2.230 | TargetScan Human | Moderate (predicted) | PM20D2 | peptidase M20 domain containing 2 | Nucleus | peptidase |
| MIMAT0003236 | miR-571 (miRNAs w/seed GAGUUGG) | 2.230 | TargetScan Human | High (predicted) | PMAIP1 | rbol-12-myristate-13-acetate-induced prote | Cytoplasm | other |
| MIMAT0003236 | miR-571 (miRNAs w/seed GAGUUGG) | 2.230 | TargetScan Human | Moderate (predicted) | PMP2 | peripheral myelin protein 2 | Cytoplasm | other |
| MIMAT0003236 | miR-571 (miRNAs w/seed GAGUUGG) | 2.230 | TargetScan Human | Moderate (predicted) | PMP22 | peripheral myelin protein 22 | Plasma Membrane | other |
| MIMAT0003236 | miR-571 (miRNAs w/seed GAGUUGG) | 2.230 | TargetScan Human | Moderate (predicted) | POC1B | POC1 centriolar protein B | Cytoplasm | other |
| MIMAT0003236 | miR-571 (miRNAs w/seed GAGUUGG) | 2.230 | TargetScan Human | High (predicted) | POGLUT1 | protein O-glucosyltransferase 1 | Extracellular Space | enzyme |
| MIMAT0003236 | miR-571 (miRNAs w/seed GAGUUGG) | 2.230 | TargetScan Human | Moderate (predicted) | POLR2G | RNA polymerase II subunit G | Nucleus | enzyme |
| MIMAT0003236 | miR-571 (miRNAs w/seed GAGUUGG) | 2.230 | TargetScan Human | High (predicted) | POU1F1 | POU class 1 homeobox 1 | Nucleus | transcription regulator |
| MIMAT0003236 | miR-571 (miRNAs w/seed GAGUUGG) | 2.230 | TargetScan Human | Moderate (predicted) | POU2F3 | POU class 2 homeobox 3 | Nucleus | transcription regulator |
| MIMAT0003236 | miR-571 (miRNAs w/seed GAGUUGG) | 2.230 | TargetScan Human | Moderate (predicted) | PP7080 | uncharacterized LOC25845 | Other | other |
| MIMAT0003236 | miR-571 (miRNAs w/seed GAGUUGG) | 2.230 | TargetScan Human | Moderate (predicted) | PPM1L | tein phosphatase, Mg2+/Mn2+ dependent | Cytoplasm | phosphatase |
| MIMAT0003236 | miR-571 (miRNAs w/seed GAGUUGG) | 2.230 | TargetScan Human | Moderate (predicted) | PPP3CC | otein phosphatase 3 catalytic subunit gam | Cytoplasm | phosphatase |
| MIMAT0003236 | miR-571 (miRNAs w/seed GAGUUGG) | 2.230 | TargetScan Human | High (predicted) | PPP3R2 | tein phosphatase 3 regulatory subunit B, b | Cytoplasm | phosphatase |
| MIMAT0003236 | miR-571 (miRNAs w/seed GAGUUGG) | 2.230 | TargetScan Human | Moderate (predicted) | PPT2 | palmitoyl-protein thioesterase 2 | Cytoplasm | enzyme |
| MIMAT0003236 | miR-571 (miRNAs w/seed GAGUUGG) | 2.230 | TargetScan Human | Moderate (predicted) | PRDM4 | PR/SET domain 4 | Nucleus | transcription regulator |
| MIMAT0003236 | miR-571 (miRNAs w/seed GAGUUGG) | 2.230 | TargetScan Human | Moderate (predicted) | PRDM5 | PR/SET domain 5 | Nucleus | transcription regulator |
| MIMAT0003236 | miR-571 (miRNAs w/seed GAGUUGG) | 2.230 | TargetScan Human | Moderate (predicted) | PRKAB2 | kinase AMP-activated non-catalytic subun | Cytoplasm | kinase |
| MIMAT0003236 | miR-571 (miRNAs w/seed GAGUUGG) | 2.230 | TargetScan Human | High (predicted) | PRKAG1 | nase AMP-activated non-catalytic subunit | Nucleus | kinase |
| MIMAT0003236 | miR-571 (miRNAs w/seed GAGUUGG) | 2.230 | TargetScan Human | Moderate (predicted) | PRKCG | protein kinase C gamma | Cytoplasm | kinase |
| MIMAT0003236 | miR-571 (miRNAs w/seed GAGUUGG) | 2.230 | TargetScan Human | Moderate (predicted) | PRPH2 | peripherin 2 | Plasma Membrane | transmembrane receptor |
| MIMAT0003236 | miR-571 (miRNAs w/seed GAGUUGG) | 2.230 | TargetScan Human | Moderate (predicted) | PRR27 | proline rich 27 | Cytoplasm | other |
| MIMAT0003236 | miR-571 (miRNAs w/seed GAGUUGG) | 2.230 | TargetScan Human | Moderate (predicted) | PRSS33 | serine protease 33 | Extracellular Space | peptidase |
| MIMAT0003236 | miR-571 (miRNAs w/seed GAGUUGG) | 2.230 | TargetScan Human | Moderate (predicted) | PRSS53 | serine protease 53 | Other | other |
| MIMAT0003236 | miR-571 (miRNAs w/seed GAGUUGG) | 2.230 | TargetScan Human | Moderate (predicted) | PRXL2B | peroxiredoxin like 2B | Cytoplasm | enzyme |
| MIMAT0003236 | miR-571 (miRNAs w/seed GAGUUGG) | 2.230 | TargetScan Human | Moderate (predicted) | PSMB7 | proteasome 20S subunit beta 7 | Cytoplasm | peptidase |
| MIMAT0003236 | miR-571 (miRNAs w/seed GAGUUGG) | 2.230 | TargetScan Human | Moderate (predicted) | PTGER3 | prostaglandin E receptor 3 | Plasma Membrane | G-protein coupled receptor |
| MIMAT0003236 | miR-571 (miRNAs w/seed GAGUUGG) | 2.230 | TargetScan Human | Moderate (predicted) | PUM1 | pumilio RNA binding family member 1 | Cytoplasm | other |
| MIMAT0003236 | miR-571 (miRNAs w/seed GAGUUGG) | 2.230 | TargetScan Human | Moderate (predicted) | PYROXD1 | e nucleotide-disulphide oxidoreductase dc | Nucleus | other |
| MIMAT0003236 | miR-571 (miRNAs w/seed GAGUUGG) | 2.230 | TargetScan Human | Moderate (predicted) | QPRT | quinolinate phosphoribosyltransferase | Cytoplasm | enzyme |
| MIMAT0003236 | miR-571 (miRNAs w/seed GAGUUGG) | 2.230 | TargetScan Human | Moderate (predicted) | RAB4B | RAB4B, member RAS oncogene family | Plasma Membrane | enzyme |
| MIMAT0003236 | miR-571 (miRNAs w/seed GAGUUGG) | 2.230 | TargetScan Human | High (predicted) | RAB9A | RAB9A, member RAS oncogene family | Cytoplasm | enzyme |
| MIMAT0003236 | miR-571 (miRNAs w/seed GAGUUGG) | 2.230 | TargetScan Human | Moderate (predicted) | RAB9B | RAB9B, member RAS oncogene family | Plasma Membrane | enzyme |
| MIMAT0003236 | miR-571 (miRNAs w/seed GAGUUGG) | 2.230 | TargetScan Human | Moderate (predicted) | RABEPK | Rab9 effector protein with kelch motifs | Cytoplasm | other |
| MIMAT0003236 | miR-571 (miRNAs w/seed GAGUUGG) | 2.230 | TargetScan Human | High (predicted) | RAC2 | Rac family small GTPase 2 | Cytoplasm | enzyme |
| MIMAT0003236 | miR-571 (miRNAs w/seed GAGUUGG) | 2.230 | TargetScan Human | High (predicted) | RANBP10 | RAN binding protein 10 | Cytoplasm | other |
| MIMAT0003236 | miR-571 (miRNAs w/seed GAGUUGG) | 2.230 | TargetScan Human | High (predicted) | RAP1A | RAP1A, member of RAS oncogene family | Cytoplasm | enzyme |
| MIMAT0003236 | miR-571 (miRNAs w/seed GAGUUGG) | 2.230 | TargetScan Human | Moderate (predicted) | RASGRP4 | RAS guanyl releasing protein 4 | Cytoplasm | other |
| MIMAT0003236 | miR-571 (miRNAs w/seed GAGUUGG) | 2.230 | TargetScan Human | Moderate (predicted) | RASSF1 | Ras association domain family member 1 | Nucleus | other |
| MIMAT0003236 | miR-571 (miRNAs w/seed GAGUUGG) | 2.230 | TargetScan Human | Moderate (predicted) | RASSF8 | Ras association domain family member 8 | Extracellular Space | other |
| MIMAT0003236 | miR-571 (miRNAs w/seed GAGUUGG) | 2.230 | TargetScan Human | Moderate (predicted) | RBM41 | RNA binding motif protein 41 | Other | other |
| MIMAT0003236 | miR-571 (miRNAs w/seed GAGUUGG) | 2.230 | TargetScan Human | Moderate (predicted) | RBMXL1 | RBMX like 1 | Nucleus | other |
| MIMAT0003236 | miR-571 (miRNAs w/seed GAGUUGG) | 2.230 | TargetScan Human | Moderate (predicted) | RDH12 | retinol dehydrogenase 12 | Cytoplasm | enzyme |
| MIMAT0003236 | miR-571 (miRNAs w/seed GAGUUGG) | 2.230 | TargetScan Human | High (predicted) | REG1A | regenerating family member 1 alpha | Extracellular Space | growth factor |
| MIMAT0003236 | miR-571 (miRNAs w/seed GAGUUGG) | 2.230 | TargetScan Human | High (predicted) | REG1B | regenerating family member 1 beta | Extracellular Space | other |
| MIMAT0003236 | miR-571 (miRNAs w/seed GAGUUGG) | 2.230 | TargetScan Human | Moderate (predicted) | REL | REL proto-oncogene, NF-kB subunit | Nucleus | transcription regulator |
| MIMAT0003236 | miR-571 (miRNAs w/seed GAGUUGG) | 2.230 | TargetScan Human | High (predicted) | RFPL1/RFPL3 | ret finger protein like 3 | Cytoplasm | other |
| MIMAT0003236 | miR-571 (miRNAs w/seed GAGUUGG) | 2.230 | TargetScan Human | Moderate (predicted) | GPD4 (includes other | ANBP2 like and GRIP domain containing | Nucleus | enzyme |
| MIMAT0003236 | miR-571 (miRNAs w/seed GAGUUGG) | 2.230 | TargetScan Human | High (predicted) | RHO | rhodopsin | Plasma Membrane | G-protein coupled receptor |
| MIMAT0003236 | miR-571 (miRNAs w/seed GAGUUGG) | 2.230 | TargetScan Human | Moderate (predicted) | RICTOR | DR independent companion of MTOR com | Cytoplasm | other |
| MIMAT0003236 | miR-571 (miRNAs w/seed GAGUUGG) | 2.230 | TargetScan Human | Moderate (predicted) | RLF | RLF zinc finger | Nucleus | transcription regulator |
| MIMAT0003236 | miR-571 (miRNAs w/seed GAGUUGG) | 2.230 | TargetScan Human | Moderate (predicted) | RNF11 | ring finger protein 11 | Cytoplasm | enzyme |
| MIMAT0003236 | miR-571 (miRNAs w/seed GAGUUGG) | 2.230 | TargetScan Human | Moderate (predicted) | RNF115 | ring finger protein 115 | Cytoplasm | enzyme |
| MIMAT0003236 | miR-571 (miRNAs w/seed GAGUUGG) | 2.230 | TargetScan Human | Moderate (predicted) | RNF146 | ring finger protein 146 | Cytoplasm | enzyme |
| MIMAT0003236 | miR-571 (miRNAs w/seed GAGUUGG) | 2.230 | TargetScan Human | Moderate (predicted) | RNF165 | ring finger protein 165 | Nucleus | enzyme |
| MIMAT0003236 | miR-571 (miRNAs w/seed GAGUUGG) | 2.230 | TargetScan Human | Moderate (predicted) | RNF7 | ring finger protein 7 | Nucleus | enzyme |
| MIMAT0003236 | miR-571 (miRNAs w/seed GAGUUGG) | 2.230 | TargetScan Human | High (predicted) | RORB | RAR related orphan receptor B | Nucleus | ligand-dependent nuclear receptor |
| MIMAT0003236 | miR-571 (miRNAs w/seed GAGUUGG) | 2.230 | TargetScan Human | Moderate (predicted) | RP11 116D171 | | Other | other |
| MIMAT0003236 | miR-571 (miRNAs w/seed GAGUUGG) | 2.230 | TargetScan Human | Moderate (predicted) | RP11 180I44 | | Other | other |
| MIMAT0003236 | miR-571 (miRNAs w/seed GAGUUGG) | 2.230 | TargetScan Human | Moderate (predicted) | RP11 215A192 | | Other | other |
| MIMAT0003236 | miR-571 (miRNAs w/seed GAGUUGG) | 2.230 | TargetScan Human | High (predicted) | RP11 363G102 | | Other | other |
| MIMAT0003236 | miR-571 (miRNAs w/seed GAGUUGG) | 2.230 | TargetScan Human | High (predicted) | RP11 76I237 | | Other | other |
| MIMAT0003236 | miR-571 (miRNAs w/seed GAGUUGG) | 2.230 | TargetScan Human | Moderate (predicted) | RP11 831H911 | | Other | other |
| MIMAT0003236 | miR-571 (miRNAs w/seed GAGUUGG) | 2.230 | TargetScan Human | Moderate (predicted) | RPAIN | RPA interacting protein | Nucleus | other |
| MIMAT0003236 | miR-571 (miRNAs w/seed GAGUUGG) | 2.230 | TargetScan Human | Moderate (predicted) | RPP25 | ribonuclease P and MRP subunit p25 | Nucleus | enzyme |
| MIMAT0003236 | miR-571 (miRNAs w/seed GAGUUGG) | 2.230 | TargetScan Human | Moderate (predicted) | RTBDN | retbindin | Extracellular Space | other |
| MIMAT0003236 | miR-571 (miRNAs w/seed GAGUUGG) | 2.230 | TargetScan Human | Moderate (predicted) | RTKN | rhotekin | Cytoplasm | other |
| MIMAT0003236 | miR-571 (miRNAs w/seed GAGUUGG) | 2.230 | TargetScan Human | Moderate (predicted) | RTP1 | receptor transporter protein 1 | Plasma Membrane | other |
| MIMAT0003236 | miR-571 (miRNAs w/seed GAGUUGG) | 2.230 | TargetScan Human | Moderate (predicted) | RUFY3 | RUN and FYVE domain containing 3 | Plasma Membrane | other |
| MIMAT0003236 | miR-571 (miRNAs w/seed GAGUUGG) | 2.230 | TargetScan Human | Moderate (predicted) | RXFP4 | relaxin family peptide/INSL5 receptor 4 | Plasma Membrane | G-protein coupled receptor |
| MIMAT0003236 | miR-571 (miRNAs w/seed GAGUUGG) | 2.230 | TargetScan Human | High (predicted) | SAMD12 | sterile alpha motif domain containing 12 | Other | other |
| MIMAT0003236 | miR-571 (miRNAs w/seed GAGUUGG) | 2.230 | TargetScan Human | Moderate (predicted) | SCAI | suppressor of cancer cell invasion | Nucleus | transcription regulator |
| MIMAT0003236 | miR-571 (miRNAs w/seed GAGUUGG) | 2.230 | TargetScan Human | Moderate (predicted) | SCN3B | odium voltage-gated channel beta subunit | Plasma Membrane | ion channel |
| MIMAT0003236 | miR-571 (miRNAs w/seed GAGUUGG) | 2.230 | TargetScan Human | High (predicted) | SCRT2 | scratch family transcriptional repressor 2 | Nucleus | transcription regulator |
| MIMAT0003236 | miR-571 (miRNAs w/seed GAGUUGG) | 2.230 | TargetScan Human | Moderate (predicted) | SCYL2 | SCY1 like pseudokinase 2 | Cytoplasm | other |
| MIMAT0003236 | miR-571 (miRNAs w/seed GAGUUGG) | 2.230 | TargetScan Human | Moderate (predicted) | SEC13 | olog, nuclear pore and COPII coat comple | Cytoplasm | transporter |
| MIMAT0003236 | miR-571 (miRNAs w/seed GAGUUGG) | 2.230 | TargetScan Human | Moderate (predicted) | SEC24B | 24 homolog B, COPII coat complex compo | Cytoplasm | transporter |
| MIMAT0003236 | miR-571 (miRNAs w/seed GAGUUGG) | 2.230 | TargetScan Human | Moderate (predicted) | SECISBP2L | SECIS binding protein 2 like | Other | other |
| MIMAT0003236 | miR-571 (miRNAs w/seed GAGUUGG) | 2.230 | TargetScan Human | Moderate (predicted) | SELENOV | selenoprotein V | Other | other |
| MIMAT0003236 | miR-571 (miRNAs w/seed GAGUUGG) | 2.230 | TargetScan Human | Moderate (predicted) | SERINC4 | serine incorporator 4 | Other | other |
| MIMAT0003236 | miR-571 (miRNAs w/seed GAGUUGG) | 2.230 | TargetScan Human | Moderate (predicted) | SERPINA7 | serpin family A member 7 | Extracellular Space | transporter |
| MIMAT0003236 | miR-571 (miRNAs w/seed GAGUUGG) | 2.230 | TargetScan Human | Moderate (predicted) | SETD7 | main containing 7, histone lysine methyltra | Nucleus | enzyme |
| MIMAT0003236 | miR-571 (miRNAs w/seed GAGUUGG) | 2.230 | TargetScan Human | Moderate (predicted) | SF3A3 | splicing factor 3a subunit 3 | Nucleus | other |
| MIMAT0003236 | miR-571 (miRNAs w/seed GAGUUGG) | 2.230 | TargetScan Human | Moderate (predicted) | SFTPA2 | surfactant protein A2 | Extracellular Space | other |
| MIMAT0003236 | miR-571 (miRNAs w/seed GAGUUGG) | 2.230 | TargetScan Human | Moderate (predicted) | SFTPB | surfactant protein B | Extracellular Space | other |
| MIMAT0003236 | miR-571 (miRNAs w/seed GAGUUGG) | 2.230 | TargetScan Human | Moderate (predicted) | SFXN3 | sideroflexin 3 | Cytoplasm | transporter |
| MIMAT0003236 | miR-571 (miRNAs w/seed GAGUUGG) | 2.230 | TargetScan Human | High (predicted) | SGTB | amine rich tetratricopeptide repeat co-chap | Other | other |
| MIMAT0003236 | miR-571 (miRNAs w/seed GAGUUGG) | 2.230 | TargetScan Human | Moderate (predicted) | SH3BGRL3 | 3 domain binding glutamate rich protein lik | Nucleus | other |
| MIMAT0003236 | miR-571 (miRNAs w/seed GAGUUGG) | 2.230 | TargetScan Human | Moderate (predicted) | SHISA9 | shisa family member 9 | Plasma Membrane | other |
| MIMAT0003236 | miR-571 (miRNAs w/seed GAGUUGG) | 2.230 | TargetScan Human | High (predicted) | SHTN1 | shootin 1 | Plasma Membrane | other |
| MIMAT0003236 | miR-571 (miRNAs w/seed GAGUUGG) | 2.230 | TargetScan Human | Moderate (predicted) | SIRPB1 | signal regulatory protein beta 1 | Plasma Membrane | other |
| MIMAT0003236 | miR-571 (miRNAs w/seed GAGUUGG) | 2.230 | TargetScan Human | Moderate (predicted) | SIRPB2 | signal regulatory protein beta 2 | Other | other |
| MIMAT0003236 | miR-571 (miRNAs w/seed GAGUUGG) | 2.230 | TargetScan Human | Moderate (predicted) | SIRPG | signal regulatory protein gamma | Plasma Membrane | other |
| MIMAT0003236 | miR-571 (miRNAs w/seed GAGUUGG) | 2.230 | TargetScan Human | Moderate (predicted) | SIX1 | SIX homeobox 1 | Nucleus | transcription regulator |
| MIMAT0003236 | miR-571 (miRNAs w/seed GAGUUGG) | 2.230 | TargetScan Human | Moderate (predicted) | SLAMF1 | g lymphocytic activation molecule family m | Plasma Membrane | transmembrane receptor |
| MIMAT0003236 | miR-571 (miRNAs w/seed GAGUUGG) | 2.230 | TargetScan Human | Moderate (predicted) | SLAMF6 | SLAM family member 6 | Plasma Membrane | transmembrane receptor |
| MIMAT0003236 | miR-571 (miRNAs w/seed GAGUUGG) | 2.230 | TargetScan Human | Moderate (predicted) | SLC17A7 | solute carrier family 17 member 7 | Plasma Membrane | transporter |
| MIMAT0003236 | miR-571 (miRNAs w/seed GAGUUGG) | 2.230 | TargetScan Human | High (predicted) | SLC18A2 | solute carrier family 18 member A2 | Plasma Membrane | transporter |
| MIMAT0003236 | miR-571 (miRNAs w/seed GAGUUGG) | 2.230 | TargetScan Human | Moderate (predicted) | SLC1A5 | solute carrier family 1 member 5 | Plasma Membrane | transporter |
| MIMAT0003236 | miR-571 (miRNAs w/seed GAGUUGG) | 2.230 | TargetScan Human | High (predicted) | SLC1A6 | solute carrier family 1 member 6 | Plasma Membrane | transporter |
| MIMAT0003236 | miR-571 (miRNAs w/seed GAGUUGG) | 2.230 | TargetScan Human | Moderate (predicted) | SLC25A3 | solute carrier family 25 member 3 | Cytoplasm | transporter |
| MIMAT0003236 | miR-571 (miRNAs w/seed GAGUUGG) | 2.230 | TargetScan Human | Moderate (predicted) | SLC28A2 | solute carrier family 28 member 2 | Plasma Membrane | transporter |
| MIMAT0003236 | miR-571 (miRNAs w/seed GAGUUGG) | 2.230 | TargetScan Human | Moderate (predicted) | SLC28A3 | solute carrier family 28 member 3 | Plasma Membrane | transporter |
| MIMAT0003236 | miR-571 (miRNAs w/seed GAGUUGG) | 2.230 | TargetScan Human | Moderate (predicted) | SLC29A1 | arrier family 29 member 1 (Augustine bloo | Plasma Membrane | transporter |
| MIMAT0003236 | miR-571 (miRNAs w/seed GAGUUGG) | 2.230 | TargetScan Human | Moderate (predicted) | SLC30A9 | solute carrier family 30 member 9 | Nucleus | transporter |
| MIMAT0003236 | miR-571 (miRNAs w/seed GAGUUGG) | 2.230 | TargetScan Human | Moderate (predicted) | SLC35D1 | solute carrier family 35 member D1 | Cytoplasm | transporter |
| MIMAT0003236 | miR-571 (miRNAs w/seed GAGUUGG) | 2.230 | TargetScan Human | Moderate (predicted) | SLC36A2 | solute carrier family 36 member 2 | Plasma Membrane | transporter |
| MIMAT0003236 | miR-571 (miRNAs w/seed GAGUUGG) | 2.230 | TargetScan Human | High (predicted) | SLC39A11 | solute carrier family 39 member 11 | Other | transporter |
| MIMAT0003236 | miR-571 (miRNAs w/seed GAGUUGG) | 2.230 | TargetScan Human | Moderate (predicted) | SLC39A4 | solute carrier family 39 member 4 | Plasma Membrane | transporter |
| MIMAT0003236 | miR-571 (miRNAs w/seed GAGUUGG) | 2.230 | TargetScan Human | Moderate (predicted) | SLC41A1 | solute carrier family 41 member 1 | Plasma Membrane | transporter |
| MIMAT0003236 | miR-571 (miRNAs w/seed GAGUUGG) | 2.230 | TargetScan Human | Moderate (predicted) | SLC52A3 | solute carrier family 52 member 3 | Plasma Membrane | transporter |
| MIMAT0003236 | miR-571 (miRNAs w/seed GAGUUGG) | 2.230 | TargetScan Human | Moderate (predicted) | SLC5A9 | solute carrier family 5 member 9 | Plasma Membrane | transporter |
| MIMAT0003236 | miR-571 (miRNAs w/seed GAGUUGG) | 2.230 | TargetScan Human | Moderate (predicted) | SLC6A20 | solute carrier family 6 member 20 | Plasma Membrane | transporter |
| MIMAT0003236 | miR-571 (miRNAs w/seed GAGUUGG) | 2.230 | TargetScan Human | Moderate (predicted) | SLC7A8 | solute carrier family 7 member 8 | Plasma Membrane | transporter |
| MIMAT0003236 | miR-571 (miRNAs w/seed GAGUUGG) | 2.230 | TargetScan Human | High (predicted) | SLN | sarcolipin | Cytoplasm | other |
| MIMAT0003236 | miR-571 (miRNAs w/seed GAGUUGG) | 2.230 | TargetScan Human | Moderate (predicted) | SMIM10 | small integral membrane protein 10 | Other | other |
| MIMAT0003236 | miR-571 (miRNAs w/seed GAGUUGG) | 2.230 | TargetScan Human | High (predicted) | SMIM14 | small integral membrane protein 14 | Cytoplasm | other |
| MIMAT0003236 | miR-571 (miRNAs w/seed GAGUUGG) | 2.230 | TargetScan Human | Moderate (predicted) | SNPH | syntaphilin | Plasma Membrane | other |
| MIMAT0003236 | miR-571 (miRNAs w/seed GAGUUGG) | 2.230 | TargetScan Human | Moderate (predicted) | SNX30 | sorting nexin family member 30 | Other | other |
| MIMAT0003236 | miR-571 (miRNAs w/seed GAGUUGG) | 2.230 | TargetScan Human | Moderate (predicted) | SNX31 | sorting nexin 31 | Other | other |
| MIMAT0003236 | miR-571 (miRNAs w/seed GAGUUGG) | 2.230 | TargetScan Human | Moderate (predicted) | SOGA3 | SOGA family member 3 | Other | other |
| MIMAT0003236 | miR-571 (miRNAs w/seed GAGUUGG) | 2.230 | TargetScan Human | Moderate (predicted) | SORD | sorbitol dehydrogenase | Cytoplasm | enzyme |
| MIMAT0003236 | miR-571 (miRNAs w/seed GAGUUGG) | 2.230 | TargetScan Human | Moderate (predicted) | SPIN2A/SPIN2B | spindlin family member 2A | Other | other |
| MIMAT0003236 | miR-571 (miRNAs w/seed GAGUUGG) | 2.230 | TargetScan Human | Moderate (predicted) | SPOCK1 | eonectin), cwcv and kazal like domains pr | Extracellular Space | other |
| MIMAT0003236 | miR-571 (miRNAs w/seed GAGUUGG) | 2.230 | TargetScan Human | Moderate (predicted) | SPSB1 | odine receptor domain and SOCS box co | Cytoplasm | other |
| MIMAT0003236 | miR-571 (miRNAs w/seed GAGUUGG) | 2.230 | TargetScan Human | Moderate (predicted) | SRSF3 | serine and arginine rich splicing factor 3 | Nucleus | other |
| MIMAT0003236 | miR-571 (miRNAs w/seed GAGUUGG) | 2.230 | TargetScan Human | High (predicted) | SS18L2 | SS18 like 2 | Other | other |
| MIMAT0003236 | miR-571 (miRNAs w/seed GAGUUGG) | 2.230 | TargetScan Human | Moderate (predicted) | ST6GALNAC4 | acetylgalactosaminide alpha-2,6-sialyltrans | Cytoplasm | enzyme |
| MIMAT0003236 | miR-571 (miRNAs w/seed GAGUUGG) | 2.230 | TargetScan Human | Moderate (predicted) | ST6GALNAC5 | acetylgalactosaminide alpha-2,6-sialyltrans | Cytoplasm | enzyme |
| MIMAT0003236 | miR-571 (miRNAs w/seed GAGUUGG) | 2.230 | TargetScan Human | Moderate (predicted) | ST7 | suppression of tumorigenicity 7 | Other | other |
| MIMAT0003236 | miR-571 (miRNAs w/seed GAGUUGG) | 2.230 | TargetScan Human | Moderate (predicted) | ST8SIA3 | a-N-acetyl-neuraminide alpha-2,8-sialyltra | Cytoplasm | enzyme |
| MIMAT0003236 | miR-571 (miRNAs w/seed GAGUUGG) | 2.230 | TargetScan Human | Moderate (predicted) | STK32B | serine/threonine kinase 32B | Other | kinase |
| MIMAT0003236 | miR-571 (miRNAs w/seed GAGUUGG) | 2.230 | TargetScan Human | High (predicted) | STK36 | serine/threonine kinase 36 | Cytoplasm | kinase |
| MIMAT0003236 | miR-571 (miRNAs w/seed GAGUUGG) | 2.230 | TargetScan Human | Moderate (predicted) | STX17 | syntaxin 17 | Plasma Membrane | transporter |
| MIMAT0003236 | miR-571 (miRNAs w/seed GAGUUGG) | 2.230 | TargetScan Human | Moderate (predicted) | SUMF1 | sulfatase modifying factor 1 | Cytoplasm | enzyme |

Sup. Table 3. List of 3699 targets of the seven dysregulated miRNAs obtained from three different target prediction databases,TargetScan TarBase and miRecords considering those predicted as moderate, high, or experimentally validated, respectively.

| | | | | | | | | |
|---|---|---|---|---|---|---|---|---|
| MIMAT0003236 | miR-571 (miRNAs w/seed GAGUUGG) | 2.230 | TargetScan Human | Moderate (predicted) | SUMO3 | small ubiquitin like modifier 3 | Nucleus | other |
| MIMAT0003236 | miR-571 (miRNAs w/seed GAGUUGG) | 2.230 | TargetScan Human | Moderate (predicted) | SV2A | synaptic vesicle glycoprotein 2A | Cytoplasm | other |
| MIMAT0003236 | miR-571 (miRNAs w/seed GAGUUGG) | 2.230 | TargetScan Human | Moderate (predicted) | SYNDIG1L | synapse differentiation inducing 1 like | Cytoplasm | other |
| MIMAT0003236 | miR-571 (miRNAs w/seed GAGUUGG) | 2.230 | TargetScan Human | Moderate (predicted) | SYNJ2 | synaptojanin 2 | Cytoplasm | phosphatase |
| MIMAT0003236 | miR-571 (miRNAs w/seed GAGUUGG) | 2.230 | TargetScan Human | Moderate (predicted) | SYNPO2 | synaptopodin 2 | Cytoplasm | other |
| MIMAT0003236 | miR-571 (miRNAs w/seed GAGUUGG) | 2.230 | TargetScan Human | Moderate (predicted) | SYP | synaptophysin | Cytoplasm | transporter |
| MIMAT0003236 | miR-571 (miRNAs w/seed GAGUUGG) | 2.230 | TargetScan Human | Moderate (predicted) | SYT14 | synaptotagmin 14 | Other | transporter |
| MIMAT0003236 | miR-571 (miRNAs w/seed GAGUUGG) | 2.230 | TargetScan Human | Moderate (predicted) | SYVN1 | synoviolin 1 | Cytoplasm | transporter |
| MIMAT0003236 | miR-571 (miRNAs w/seed GAGUUGG) | 2.230 | TargetScan Human | Moderate (predicted) | TAC4 | tachykinin precursor 4 | Extracellular Space | other |
| MIMAT0003236 | miR-571 (miRNAs w/seed GAGUUGG) | 2.230 | TargetScan Human | Moderate (predicted) | TANC2 | ptide repeat, ankyrin repeat and coiled-coil | Nucleus | transcription regulator |
| MIMAT0003236 | miR-571 (miRNAs w/seed GAGUUGG) | 2.230 | TargetScan Human | Moderate (predicted) | TAS1R1 | taste 1 receptor member 1 | Plasma Membrane | G-protein coupled receptor |
| MIMAT0003236 | miR-571 (miRNAs w/seed GAGUUGG) | 2.230 | TargetScan Human | Moderate (predicted) | TBC1D23 | TBC1 domain family member 23 | Cytoplasm | other |
| MIMAT0003236 | miR-571 (miRNAs w/seed GAGUUGG) | 2.230 | TargetScan Human | Moderate (predicted) | TBKBP1 | TBK1 binding protein 1 | Other | other |
| MIMAT0003236 | miR-571 (miRNAs w/seed GAGUUGG) | 2.230 | TargetScan Human | Moderate (predicted) | TCAF1 | TRPM8 channel associated factor 1 | Plasma Membrane | other |
| MIMAT0003236 | miR-571 (miRNAs w/seed GAGUUGG) | 2.230 | TargetScan Human | Moderate (predicted) | TCEA1 | transcription elongation factor A1 | Nucleus | transcription regulator |
| MIMAT0003236 | miR-571 (miRNAs w/seed GAGUUGG) | 2.230 | TargetScan Human | Moderate (predicted) | TCF7L2 | transcription factor 7 like 2 | Nucleus | transcription regulator |
| MIMAT0003236 | miR-571 (miRNAs w/seed GAGUUGG) | 2.230 | TargetScan Human | Moderate (predicted) | TCIM | anscriptional and immune response regula | Nucleus | other |
| MIMAT0003236 | miR-571 (miRNAs w/seed GAGUUGG) | 2.230 | TargetScan Human | High (predicted) | TCTN1 | tectonic family member 1 | Extracellular Space | other |
| MIMAT0003236 | miR-571 (miRNAs w/seed GAGUUGG) | 2.230 | TargetScan Human | Moderate (predicted) | TEX35 | testis expressed 35 | Nucleus | other |
| MIMAT0003236 | miR-571 (miRNAs w/seed GAGUUGG) | 2.230 | TargetScan Human | Moderate (predicted) | TFRC | transferrin receptor | Plasma Membrane | transporter |
| MIMAT0003236 | miR-571 (miRNAs w/seed GAGUUGG) | 2.230 | TargetScan Human | Moderate (predicted) | TIMM8B | ase of inner mitochondrial membrane 8 ho | Cytoplasm | transporter |
| MIMAT0003236 | miR-571 (miRNAs w/seed GAGUUGG) | 2.230 | TargetScan Human | Moderate (predicted) | TIMP2 | TIMP metallopeptidase inhibitor 2 | Extracellular Space | other |
| MIMAT0003236 | miR-571 (miRNAs w/seed GAGUUGG) | 2.230 | TargetScan Human | Moderate (predicted) | TLR4 | toll like receptor 4 | Plasma Membrane | transmembrane receptor |
| MIMAT0003236 | miR-571 (miRNAs w/seed GAGUUGG) | 2.230 | TargetScan Human | Moderate (predicted) | TM6SF2 | transmembrane 6 superfamily member 2 | Plasma Membrane | other |
| MIMAT0003236 | miR-571 (miRNAs w/seed GAGUUGG) | 2.230 | TargetScan Human | Moderate (predicted) | TMCC3 | nsmembrane and coiled-coil domain famil | Cytoplasm | other |
| MIMAT0003236 | miR-571 (miRNAs w/seed GAGUUGG) | 2.230 | TargetScan Human | High (predicted) | TMED2 | transmembrane p24 trafficking protein 2 | Cytoplasm | transporter |
| MIMAT0003236 | miR-571 (miRNAs w/seed GAGUUGG) | 2.230 | TargetScan Human | Moderate (predicted) | TMEM144 | transmembrane protein 144 | Other | other |
| MIMAT0003236 | miR-571 (miRNAs w/seed GAGUUGG) | 2.230 | TargetScan Human | Moderate (predicted) | TMEM154 | transmembrane protein 154 | Other | other |
| MIMAT0003236 | miR-571 (miRNAs w/seed GAGUUGG) | 2.230 | TargetScan Human | Moderate (predicted) | TMEM159 | transmembrane protein 159 | Other | other |
| MIMAT0003236 | miR-571 (miRNAs w/seed GAGUUGG) | 2.230 | TargetScan Human | Moderate (predicted) | TMEM181 | transmembrane protein 181 | Other | other |
| MIMAT0003236 | miR-571 (miRNAs w/seed GAGUUGG) | 2.230 | TargetScan Human | Moderate (predicted) | TMEM184A | transmembrane protein 184A | Cytoplasm | other |
| MIMAT0003236 | miR-571 (miRNAs w/seed GAGUUGG) | 2.230 | TargetScan Human | Moderate (predicted) | TMEM215 | transmembrane protein 215 | Other | other |
| MIMAT0003236 | miR-571 (miRNAs w/seed GAGUUGG) | 2.230 | TargetScan Human | Moderate (predicted) | TMEM229B | transmembrane protein 229B | Other | other |
| MIMAT0003236 | miR-571 (miRNAs w/seed GAGUUGG) | 2.230 | TargetScan Human | Moderate (predicted) | TMEM239 | transmembrane protein 239 | Other | other |
| MIMAT0003236 | miR-571 (miRNAs w/seed GAGUUGG) | 2.230 | TargetScan Human | Moderate (predicted) | TMEM253 | transmembrane protein 253 | Other | other |
| MIMAT0003236 | miR-571 (miRNAs w/seed GAGUUGG) | 2.230 | TargetScan Human | Moderate (predicted) | TMPRSS11D | transmembrane serine protease 11D | Extracellular Space | peptidase |
| MIMAT0003236 | miR-571 (miRNAs w/seed GAGUUGG) | 2.230 | TargetScan Human | Moderate (predicted) | TMUB2 | nembrane and ubiquitin like domain contai | Other | other |
| MIMAT0003236 | miR-571 (miRNAs w/seed GAGUUGG) | 2.230 | TargetScan Human | Moderate (predicted) | TNFRSF12A | TNF receptor superfamily member 12A | Plasma Membrane | transmembrane receptor |
| MIMAT0003236 | miR-571 (miRNAs w/seed GAGUUGG) | 2.230 | TargetScan Human | Moderate (predicted) | TNFRSF4 | TNF receptor superfamily member 4 | Plasma Membrane | transmembrane receptor |
| MIMAT0003236 | miR-571 (miRNAs w/seed GAGUUGG) | 2.230 | TargetScan Human | Moderate (predicted) | TOMM34 | nslocase of outer mitochondrial membrane | Cytoplasm | other |
| MIMAT0003236 | miR-571 (miRNAs w/seed GAGUUGG) | 2.230 | TargetScan Human | Moderate (predicted) | TP53I11 | tumor protein p53 inducible protein 11 | Other | other |
| MIMAT0003236 | miR-571 (miRNAs w/seed GAGUUGG) | 2.230 | TargetScan Human | Moderate (predicted) | TPTE | smembrane phosphatase with tensin homo | Plasma Membrane | phosphatase |
| MIMAT0003236 | miR-571 (miRNAs w/seed GAGUUGG) | 2.230 | TargetScan Human | Moderate (predicted) | TRAF3IP3 | TRAF3 interacting protein 3 | Other | other |
| MIMAT0003236 | miR-571 (miRNAs w/seed GAGUUGG) | 2.230 | TargetScan Human | Moderate (predicted) | TRAPPC3L | afficking protein particle complex subunit 3 | Other | other |
| MIMAT0003236 | miR-571 (miRNAs w/seed GAGUUGG) | 2.230 | TargetScan Human | Moderate (predicted) | TRAPPC4 | rafficking protein particle complex subunit | Cytoplasm | other |
| MIMAT0003236 | miR-571 (miRNAs w/seed GAGUUGG) | 2.230 | TargetScan Human | Moderate (predicted) | TREML1 | ering receptor expressed on myeloid cells | Plasma Membrane | other |
| MIMAT0003236 | miR-571 (miRNAs w/seed GAGUUGG) | 2.230 | TargetScan Human | Moderate (predicted) | TRIB2 | tribbles pseudokinase 2 | Plasma Membrane | kinase |
| MIMAT0003236 | miR-571 (miRNAs w/seed GAGUUGG) | 2.230 | TargetScan Human | Moderate (predicted) | TRIM26 | tripartite motif containing 26 | Cytoplasm | other |
| MIMAT0003236 | miR-571 (miRNAs w/seed GAGUUGG) | 2.230 | TargetScan Human | Moderate (predicted) | TRIM9 | tripartite motif containing 9 | Cytoplasm | enzyme |
| MIMAT0003236 | miR-571 (miRNAs w/seed GAGUUGG) | 2.230 | TargetScan Human | Moderate (predicted) | TRNP1 | TMF1 regulated nuclear protein 1 | Nucleus | other |
| MIMAT0003236 | miR-571 (miRNAs w/seed GAGUUGG) | 2.230 | TargetScan Human | Moderate (predicted) | TUBB6 | tubulin beta 6 class V | Cytoplasm | other |
| MIMAT0003236 | miR-571 (miRNAs w/seed GAGUUGG) | 2.230 | TargetScan Human | Moderate (predicted) | TUBD1 | tubulin delta 1 | Cytoplasm | other |
| MIMAT0003236 | miR-571 (miRNAs w/seed GAGUUGG) | 2.230 | TargetScan Human | Moderate (predicted) | TXK | TXK tyrosine kinase | Cytoplasm | kinase |
| MIMAT0003236 | miR-571 (miRNAs w/seed GAGUUGG) | 2.230 | TargetScan Human | High (predicted) | TYR | tyrosinase | Cytoplasm | enzyme |
| MIMAT0003236 | miR-571 (miRNAs w/seed GAGUUGG) | 2.230 | TargetScan Human | High (predicted) | TYROBP | membrane immune signaling adaptor TYF | Plasma Membrane | transmembrane receptor |
| MIMAT0003236 | miR-571 (miRNAs w/seed GAGUUGG) | 2.230 | TargetScan Human | High (predicted) | UAP1 | P-N-acetylglucosamine pyrophosphorylas | Nucleus | enzyme |
| MIMAT0003236 | miR-571 (miRNAs w/seed GAGUUGG) | 2.230 | TargetScan Human | Moderate (predicted) | UAP1L1 | N-acetylglucosamine pyrophosphorylase 1 | Other | other |
| MIMAT0003236 | miR-571 (miRNAs w/seed GAGUUGG) | 2.230 | TargetScan Human | Moderate (predicted) | UBD | ubiquitin D | Nucleus | other |
| MIMAT0003236 | miR-571 (miRNAs w/seed GAGUUGG) | 2.230 | TargetScan Human | Moderate (predicted) | UBE2D2 | ubiquitin conjugating enzyme E2 D2 | Cytoplasm | enzyme |
| MIMAT0003236 | miR-571 (miRNAs w/seed GAGUUGG) | 2.230 | TargetScan Human | Moderate (predicted) | UBE2L6 | ubiquitin conjugating enzyme E2 L6 | Cytoplasm | enzyme |
| MIMAT0003236 | miR-571 (miRNAs w/seed GAGUUGG) | 2.230 | TargetScan Human | Moderate (predicted) | UBE2S | ubiquitin conjugating enzyme E2 S | Nucleus | enzyme |
| MIMAT0003236 | miR-571 (miRNAs w/seed GAGUUGG) | 2.230 | TargetScan Human | Moderate (predicted) | UBXN2B | UBX domain protein 2B | Cytoplasm | other |
| MIMAT0003236 | miR-571 (miRNAs w/seed GAGUUGG) | 2.230 | TargetScan Human | Moderate (predicted) | UGCG | UDP-glucose ceramide glucosyltransferas | Cytoplasm | enzyme |
| MIMAT0003236 | miR-571 (miRNAs w/seed GAGUUGG) | 2.230 | TargetScan Human | Moderate (predicted) | ULK3 | unc-51 like kinase 3 | Cytoplasm | kinase |
| MIMAT0003236 | miR-571 (miRNAs w/seed GAGUUGG) | 2.230 | TargetScan Human | Moderate (predicted) | USP51 | ubiquitin specific peptidase 51 | Other | peptidase |
| MIMAT0003236 | miR-571 (miRNAs w/seed GAGUUGG) | 2.230 | TargetScan Human | Moderate (predicted) | VPS28 | VPS28 subunit of ESCRT-I | Cytoplasm | transporter |
| MIMAT0003236 | miR-571 (miRNAs w/seed GAGUUGG) | 2.230 | TargetScan Human | Moderate (predicted) | VPS45 | vacuolar protein sorting 45 homolog | Cytoplasm | transporter |
| MIMAT0003236 | miR-571 (miRNAs w/seed GAGUUGG) | 2.230 | TargetScan Human | Moderate (predicted) | VSX2 | visual system homeobox 2 | Nucleus | transcription regulator |
| MIMAT0003236 | miR-571 (miRNAs w/seed GAGUUGG) | 2.230 | TargetScan Human | Moderate (predicted) | VTI1A | e transport through interaction with t-SNAF | Plasma Membrane | transporter |
| MIMAT0003236 | miR-571 (miRNAs w/seed GAGUUGG) | 2.230 | TargetScan Human | Moderate (predicted) | WBP1 | WW domain binding protein 1 | Cytoplasm | other |
| MIMAT0003236 | miR-571 (miRNAs w/seed GAGUUGG) | 2.230 | TargetScan Human | Moderate (predicted) | WDFY1 | WD repeat and FYVE domain containing 1 | Cytoplasm | other |
| MIMAT0003236 | miR-571 (miRNAs w/seed GAGUUGG) | 2.230 | TargetScan Human | Moderate (predicted) | WFDC10A | WAP four-disulfide core domain 10A | Extracellular Space | other |
| MIMAT0003236 | miR-571 (miRNAs w/seed GAGUUGG) | 2.230 | TargetScan Human | Moderate (predicted) | WNT2B | Wnt family member 2B | Extracellular Space | other |
| MIMAT0003236 | miR-571 (miRNAs w/seed GAGUUGG) | 2.230 | TargetScan Human | Moderate (predicted) | WNT8B | Wnt family member 8B | Extracellular Space | other |
| MIMAT0003236 | miR-571 (miRNAs w/seed GAGUUGG) | 2.230 | TargetScan Human | Moderate (predicted) | XDH | xanthine dehydrogenase | Cytoplasm | enzyme |
| MIMAT0003236 | miR-571 (miRNAs w/seed GAGUUGG) | 2.230 | TargetScan Human | High (predicted) | XG | Xg glycoprotein (Xg blood group) | Plasma Membrane | other |
| MIMAT0003236 | miR-571 (miRNAs w/seed GAGUUGG) | 2.230 | TargetScan Human | Moderate (predicted) | YWHAE | genase/tryptophan 5-monooxygenase activ | Cytoplasm | other |
| MIMAT0003236 | miR-571 (miRNAs w/seed GAGUUGG) | 2.230 | TargetScan Human | Moderate (predicted) | ZBED2 | zinc finger BED-type containing 2 | Nucleus | transcription regulator |
| MIMAT0003236 | miR-571 (miRNAs w/seed GAGUUGG) | 2.230 | TargetScan Human | Moderate (predicted) | ZBTB34 | zinc finger and BTB domain containing 34 | Nucleus | transcription regulator |
| MIMAT0003236 | miR-571 (miRNAs w/seed GAGUUGG) | 2.230 | TargetScan Human | Moderate (predicted) | ZBTB42 | zinc finger and BTB domain containing 42 | Cytoplasm | transcription regulator |
| MIMAT0003236 | miR-571 (miRNAs w/seed GAGUUGG) | 2.230 | TargetScan Human | Moderate (predicted) | ZBTB7A | zinc finger and BTB domain containing 7A | Nucleus | transcription regulator |
| MIMAT0003236 | miR-571 (miRNAs w/seed GAGUUGG) | 2.230 | TargetScan Human | Moderate (predicted) | ZC4H2 | zinc finger C4H2-type containing | Nucleus | other |
| MIMAT0003236 | miR-571 (miRNAs w/seed GAGUUGG) | 2.230 | TargetScan Human | Moderate (predicted) | ZDHHC15 | nc finger DHHC-type palmitoyltransferase | Cytoplasm | enzyme |
| MIMAT0003236 | miR-571 (miRNAs w/seed GAGUUGG) | 2.230 | TargetScan Human | Moderate (predicted) | ZDHHC21 | nc finger DHHC-type palmitoyltransferase | Plasma Membrane | enzyme |
| MIMAT0003236 | miR-571 (miRNAs w/seed GAGUUGG) | 2.230 | TargetScan Human | Moderate (predicted) | ZFP69B | ZFP69 zinc finger protein B | Nucleus | transcription regulator |
| MIMAT0003236 | miR-571 (miRNAs w/seed GAGUUGG) | 2.230 | TargetScan Human | Moderate (predicted) | ZMAT3 | zinc finger matrin-type 3 | Nucleus | other |
| MIMAT0003236 | miR-571 (miRNAs w/seed GAGUUGG) | 2.230 | TargetScan Human | Moderate (predicted) | ZNF16 | zinc finger protein 16 | Nucleus | transcription regulator |
| MIMAT0003236 | miR-571 (miRNAs w/seed GAGUUGG) | 2.230 | TargetScan Human | Moderate (predicted) | ZNF286A | zinc finger protein 286A | Nucleus | transcription regulator |
| MIMAT0003236 | miR-571 (miRNAs w/seed GAGUUGG) | 2.230 | TargetScan Human | Moderate (predicted) | ZNF384 | zinc finger protein 384 | Nucleus | transcription regulator |
| MIMAT0003236 | miR-571 (miRNAs w/seed GAGUUGG) | 2.230 | TargetScan Human | Moderate (predicted) | ZNF391 | zinc finger protein 391 | Other | other |
| MIMAT0003236 | miR-571 (miRNAs w/seed GAGUUGG) | 2.230 | TargetScan Human | Moderate (predicted) | ZNF444 | zinc finger protein 444 | Nucleus | transcription regulator |
| MIMAT0003236 | miR-571 (miRNAs w/seed GAGUUGG) | 2.230 | TargetScan Human | Moderate (predicted) | ZNF471 | zinc finger protein 471 | Nucleus | transcription regulator |
| MIMAT0003236 | miR-571 (miRNAs w/seed GAGUUGG) | 2.230 | TargetScan Human | Moderate (predicted) | ZNF527 | zinc finger protein 527 | Nucleus | other |
| MIMAT0003236 | miR-571 (miRNAs w/seed GAGUUGG) | 2.230 | TargetScan Human | High (predicted) | ZNF544 | zinc finger protein 544 | Nucleus | transcription regulator |
| MIMAT0003236 | miR-571 (miRNAs w/seed GAGUUGG) | 2.230 | TargetScan Human | Moderate (predicted) | ZNF585A | zinc finger protein 585A | Nucleus | transcription regulator |
| MIMAT0003236 | miR-571 (miRNAs w/seed GAGUUGG) | 2.230 | TargetScan Human | Moderate (predicted) | ZNF655 | zinc finger protein 655 | Nucleus | transcription regulator |
| MIMAT0003236 | miR-571 (miRNAs w/seed GAGUUGG) | 2.230 | TargetScan Human | Moderate (predicted) | ZNF676 | zinc finger protein 676 | Nucleus | transcription regulator |
| MIMAT0003236 | miR-571 (miRNAs w/seed GAGUUGG) | 2.230 | TargetScan Human | Moderate (predicted) | ZNF697 | zinc finger protein 697 | Other | other |
| MIMAT0003236 | miR-571 (miRNAs w/seed GAGUUGG) | 2.230 | TargetScan Human | Moderate (predicted) | ZNF781 | zinc finger protein 781 | Other | other |
| MIMAT0003236 | miR-571 (miRNAs w/seed GAGUUGG) | 2.230 | TargetScan Human | Moderate (predicted) | ZSCAN30 | zinc finger and SCAN domain containing 3 | Other | other |
| MIMAT0003236 | miR-571 (miRNAs w/seed GAGUUGG) | 2.230 | TargetScan Human | Moderate (predicted) | ZWINT | ZW10 interacting kinetochore protein | Nucleus | other |
| MIMAT0000738 | miR-383-5p (miRNAs w/seed GAUCAGA) | 2.030 | TargetScan Human | Moderate (predicted) | ABCC11 | TP binding cassette subfamily C member | Plasma Membrane | transporter |
| MIMAT0000738 | miR-383-5p (miRNAs w/seed GAUCAGA) | 2.030 | TargetScan Human | Moderate (predicted) | ABHD10 | ydrolase domain containing 10, depalmito | Cytoplasm | enzyme |
| MIMAT0000738 | miR-383-5p (miRNAs w/seed GAUCAGA) | 2.030 | TargetScan Human | Moderate (predicted) | ACO1 | aconitase 1 | Cytoplasm | enzyme |
| MIMAT0000738 | miR-383-5p (miRNAs w/seed GAUCAGA) | 2.030 | TargetScan Human | Moderate (predicted) | ACO2 | aconitase 2 | Cytoplasm | enzyme |
| MIMAT0000738 | miR-383-5p (miRNAs w/seed GAUCAGA) | 2.030 | TargetScan Human | Moderate (predicted) | ADAM10 | ADAM metallopeptidase domain 10 | Plasma Membrane | peptidase |
| MIMAT0000738 | miR-383-5p (miRNAs w/seed GAUCAGA) | 2.030 | TargetScan Human | Moderate (predicted) | ADAM30 | ADAM metallopeptidase domain 30 | Plasma Membrane | peptidase |
| MIMAT0000738 | miR-383-5p (miRNAs w/seed GAUCAGA) | 2.030 | TargetScan Human | Moderate (predicted) | ADIPOR2 | adiponectin receptor 2 | Plasma Membrane | transmembrane receptor |
| MIMAT0000738 | miR-383-5p (miRNAs w/seed GAUCAGA) | 2.030 | TargetScan Human | Moderate (predicted) | ADM2 | adrenomedullin 2 | Extracellular Space | other |
| MIMAT0000738 | miR-383-5p (miRNAs w/seed GAUCAGA) | 2.030 | TargetScan Human | Moderate (predicted) | ADPGK | ADP dependent glucokinase | Plasma Membrane | kinase |
| MIMAT0000738 | miR-383-5p (miRNAs w/seed GAUCAGA) | 2.030 | TargetScan Human | High (predicted) | ADSS2 | adenylosuccinate synthase 2 | Cytoplasm | enzyme |
| MIMAT0000738 | miR-383-5p (miRNAs w/seed GAUCAGA) | 2.030 | TargetScan Human | Moderate (predicted) | AF011889.5 | | Other | other |
| MIMAT0000738 | miR-383-5p (miRNAs w/seed GAUCAGA) | 2.030 | TargetScan Human | High (predicted) | AFAP1L1 | actin filament associated protein 1 like 1 | Other | other |
| MIMAT0000738 | miR-383-5p (miRNAs w/seed GAUCAGA) | 2.030 | TargetScan Human | Moderate (predicted) | AGFG1 | ArfGAP with FG repeats 1 | Nucleus | other |
| MIMAT0000738 | miR-383-5p (miRNAs w/seed GAUCAGA) | 2.030 | TargetScan Human | Moderate (predicted) | AGO1 | argonaute RISC component 1 | Cytoplasm | translation regulator |
| MIMAT0000738 | miR-383-5p (miRNAs w/seed GAUCAGA) | 2.030 | TargetScan Human | Moderate (predicted) | AJAP1 | adherens junctions associated protein 1 | Plasma Membrane | other |
| MIMAT0000738 | miR-383-5p (miRNAs w/seed GAUCAGA) | 2.030 | TargetScan Human | Moderate (predicted) | AKAP10 | A-kinase anchoring protein 10 | Cytoplasm | other |
| MIMAT0000738 | miR-383-5p (miRNAs w/seed GAUCAGA) | 2.030 | TargetScan Human | Moderate (predicted) | AKAP12 | A-kinase anchoring protein 12 | Cytoplasm | transporter |
| MIMAT0000738 | miR-383-5p (miRNAs w/seed GAUCAGA) | 2.030 | TargetScan Human | Moderate (predicted) | AKAP17A | A-kinase anchoring protein 17A | Nucleus | other |
| MIMAT0000738 | miR-383-5p (miRNAs w/seed GAUCAGA) | 2.030 | TargetScan Human | Moderate (predicted) | AKAP7 | A-kinase anchoring protein 7 | Plasma Membrane | other |
| MIMAT0000738 | miR-383-5p (miRNAs w/seed GAUCAGA) | 2.030 | TargetScan Human | High (predicted) | AKR1B10 | aldo-keto reductase family 1 member B10 | Cytoplasm | enzyme |
| MIMAT0000738 | miR-383-5p (miRNAs w/seed GAUCAGA) | 2.030 | TargetScan Human | High (predicted) | AKR1B15 | aldo-keto reductase family 1 member B15 | Cytoplasm | enzyme |
| MIMAT0000738 | miR-383-5p (miRNAs w/seed GAUCAGA) | 2.030 | TargetScan Human | High (predicted) | ALG11 | ALG11 alpha-1,2-mannosyltransferase | Cytoplasm | enzyme |
| MIMAT0000738 | miR-383-5p (miRNAs w/seed GAUCAGA) | 2.030 | TargetScan Human | Moderate (predicted) | AMBRA1 | autophagy and beclin 1 regulator 1 | Cytoplasm | other |
| MIMAT0000738 | miR-383-5p (miRNAs w/seed GAUCAGA) | 2.030 | TargetScan Human | Moderate (predicted) | ANKDD1A | kyrin repeat and death domain containing | Other | other |
| MIMAT0000738 | miR-383-5p (miRNAs w/seed GAUCAGA) | 2.030 | TargetScan Human | Moderate (predicted) | ANKRD11 | ankyrin repeat domain 11 | Nucleus | other |
| MIMAT0000738 | miR-383-5p (miRNAs w/seed GAUCAGA) | 2.030 | TargetScan Human | Moderate (predicted) | ANKRD34C | ankyrin repeat domain 34C | Other | other |
| MIMAT0000738 | miR-383-5p (miRNAs w/seed GAUCAGA) | 2.030 | TargetScan Human | Moderate (predicted) | ANKUB1 | kyrin repeat and ubiquitin domain containir | Other | other |
| MIMAT0000738 | miR-383-5p (miRNAs w/seed GAUCAGA) | 2.030 | TargetScan Human | Moderate (predicted) | ANO5 | anoctamin 5 | Plasma Membrane | ion channel |
| MIMAT0000738 | miR-383-5p (miRNAs w/seed GAUCAGA) | 2.030 | TargetScan Human | Moderate (predicted) | ANTXRL | ANTXR like | Other | other |
| MIMAT0000738 | miR-383-5p (miRNAs w/seed GAUCAGA) | 2.030 | TargetScan Human | Moderate (predicted) | ANXA5 | annexin A5 | Plasma Membrane | transporter |
| MIMAT0000738 | miR-383-5p (miRNAs w/seed GAUCAGA) | 2.030 | TargetScan Human | Moderate (predicted) | AP3M1 | aptor related protein complex 3 subunit m | Cytoplasm | transporter |
| MIMAT0000738 | miR-383-5p (miRNAs w/seed GAUCAGA) | 2.030 | TargetScan Human | Moderate (predicted) | APEH | acylaminoacyl-peptide hydrolase | Cytoplasm | peptidase |
| MIMAT0000738 | miR-383-5p (miRNAs w/seed GAUCAGA) | 2.030 | TargetScan Human | Moderate (predicted) | APOBEC3B | rotein B mRNA editing enzyme catalytic su | Cytoplasm | enzyme |
| MIMAT0000738 | miR-383-5p (miRNAs w/seed GAUCAGA) | 2.030 | TargetScan Human | Moderate (predicted) | APOBEC3G | rotein B mRNA editing enzyme catalytic su | Nucleus | enzyme |
| MIMAT0000738 | miR-383-5p (miRNAs w/seed GAUCAGA) | 2.030 | TargetScan Human | Moderate (predicted) | APOL6 | apolipoprotein L6 | Extracellular Space | transporter |
| MIMAT0000738 | miR-383-5p (miRNAs w/seed GAUCAGA) | 2.030 | TargetScan Human | Moderate (predicted) | ARHGAP28 | Rho GTPase activating protein 28 | Cytoplasm | other |
| MIMAT0000738 | miR-383-5p (miRNAs w/seed GAUCAGA) | 2.030 | TargetScan Human | Moderate (predicted) | ARID2 | AT-rich interaction domain 2 | Nucleus | transcription regulator |
| MIMAT0000738 | miR-383-5p (miRNAs w/seed GAUCAGA) | 2.030 | TargetScan Human | Moderate (predicted) | CX5-GPRASP2/GPR | ein-coupled receptor associated sorting pr | Plasma Membrane | other |
| MIMAT0000738 | miR-383-5p (miRNAs w/seed GAUCAGA) | 2.030 | TargetScan Human | Moderate (predicted) | ARPC4-TTLL3 | ARPC4-TTLL3 readthrough | Other | other |
| MIMAT0000738 | miR-383-5p (miRNAs w/seed GAUCAGA) | 2.030 | TargetScan Human | Moderate (predicted) | AS3MT | arsenite methyltransferase | Cytoplasm | enzyme |
| MIMAT0000738 | miR-383-5p (miRNAs w/seed GAUCAGA) | 2.030 | TargetScan Human | Moderate (predicted) | ASB3/GPR75-ASB3 | ankyrin repeat and SOCS box containing 3 | Cytoplasm | transcription regulator |
| MIMAT0000738 | miR-383-5p (miRNAs w/seed GAUCAGA) | 2.030 | TargetScan Human | Moderate (predicted) | ATE1 | arginyltransferase 1 | Nucleus | enzyme |
| MIMAT0000738 | miR-383-5p (miRNAs w/seed GAUCAGA) | 2.030 | TargetScan Human | Moderate (predicted) | ATF7 | activating transcription factor 7 | Nucleus | transcription regulator |
| MIMAT0000738 | miR-383-5p (miRNAs w/seed GAUCAGA) | 2.030 | TargetScan Human | Moderate (predicted) | ATP13A3 | ATPase 13A3 | Extracellular Space | transporter |
| MIMAT0000738 | miR-383-5p (miRNAs w/seed GAUCAGA) | 2.030 | TargetScan Human | Moderate (predicted) | ATP6V1G2 | ATPase H+ transporting V1 subunit G2 | Cytoplasm | transporter |

Sup. Table 3. List of 3699 targets of the seven dysregulated miRNAs obtained from three different target prediction databases,TargetScan TarBase and miRecords considering those predicted as moderate, high, or experimentally validated, respectively.

| | | | | | | | | |
|---|---|---|---|---|---|---|---|---|
| MIMAT0000738 | miR-383-5p (miRNAs w/seed GAUCAGA) | 2.030 | TargetScan Human | Moderate (predicted) | ATP7B | ATPase copper transporting beta | Cytoplasm | transporter |
| MIMAT0000738 | miR-383-5p (miRNAs w/seed GAUCAGA) | 2.030 | TargetScan Human | Moderate (predicted) | ATP8A1 | ATPase phospholipid transporting 8A1 | Cytoplasm | transporter |
| MIMAT0000738 | miR-383-5p (miRNAs w/seed GAUCAGA) | 2.030 | TargetScan Human | High (predicted) | ATR | ATR serine/threonine kinase | Nucleus | kinase |
| MIMAT0000738 | miR-383-5p (miRNAs w/seed GAUCAGA) | 2.030 | TargetScan Human | High (predicted) | ATXN3 | ataxin 3 | Nucleus | peptidase |
| MIMAT0000738 | miR-383-5p (miRNAs w/seed GAUCAGA) | 2.030 | TargetScan Human | Moderate (predicted) | AZI2 | 5-azacytidine induced 2 | Cytoplasm | other |
| MIMAT0000738 | miR-383-5p (miRNAs w/seed GAUCAGA) | 2.030 | TargetScan Human | Moderate (predicted) | BCAP29 | B cell receptor associated protein 29 | Cytoplasm | transporter |
| MIMAT0000738 | miR-383-5p (miRNAs w/seed GAUCAGA) | 2.030 | TargetScan Human | Moderate (predicted) | BCL2 | BCL2 apoptosis regulator | Cytoplasm | transporter |
| MIMAT0000738 | miR-383-5p (miRNAs w/seed GAUCAGA) | 2.030 | TargetScan Human | Moderate (predicted) | BCL2L13 | BCL2 like 13 | Cytoplasm | other |
| MIMAT0000738 | miR-383-5p (miRNAs w/seed GAUCAGA) | 2.030 | TargetScan Human | Moderate (predicted) | BCL2L2 | BCL2 like 2 | Cytoplasm | other |
| MIMAT0000738 | miR-383-5p (miRNAs w/seed GAUCAGA) | 2.030 | TargetScan Human | Moderate (predicted) | BEND7 | BEN domain containing 7 | Cytoplasm | other |
| MIMAT0000738 | miR-383-5p (miRNAs w/seed GAUCAGA) | 2.030 | TargetScan Human | Moderate (predicted) | BLOC1S6 | esis of lysosomal organelles complex 1 su | Cytoplasm | other |
| MIMAT0000738 | miR-383-5p (miRNAs w/seed GAUCAGA) | 2.030 | TargetScan Human | High (predicted) | BRCC3 | RCA1/BRCA2-containing complex subuni | Nucleus | enzyme |
| MIMAT0000738 | miR-383-5p (miRNAs w/seed GAUCAGA) | 2.030 | TargetScan Human | Moderate (predicted) | C10orf105 | chromosome 10 open reading frame 105 | Other | other |
| MIMAT0000738 | miR-383-5p (miRNAs w/seed GAUCAGA) | 2.030 | TargetScan Human | High (predicted) | C10orf71 | chromosome 10 open reading frame 71 | Other | other |
| MIMAT0000738 | miR-383-5p (miRNAs w/seed GAUCAGA) | 2.030 | TargetScan Human | Moderate (predicted) | C11orf21 | chromosome 11 open reading frame 21 | Cytoplasm | other |
| MIMAT0000738 | miR-383-5p (miRNAs w/seed GAUCAGA) | 2.030 | TargetScan Human | Moderate (predicted) | C11orf87 | chromosome 11 open reading frame 87 | Other | other |
| MIMAT0000738 | miR-383-5p (miRNAs w/seed GAUCAGA) | 2.030 | TargetScan Human | Moderate (predicted) | C16orf54 | chromosome 16 open reading frame 54 | Extracellular Space | other |
| MIMAT0000738 | miR-383-5p (miRNAs w/seed GAUCAGA) | 2.030 | TargetScan Human | Moderate (predicted) | C16orf90 | chromosome 16 open reading frame 90 | Other | other |
| MIMAT0000738 | miR-383-5p (miRNAs w/seed GAUCAGA) | 2.030 | TargetScan Human | High (predicted) | C17orf50 | chromosome 17 open reading frame 50 | Other | other |
| MIMAT0000738 | miR-383-5p (miRNAs w/seed GAUCAGA) | 2.030 | TargetScan Human | Moderate (predicted) | C1QTNF7 | C1q and TNF related 7 | Extracellular Space | other |
| MIMAT0000738 | miR-383-5p (miRNAs w/seed GAUCAGA) | 2.030 | TargetScan Human | Moderate (predicted) | C20orf197 | omosome 20 putative open reading frame | Other | other |
| MIMAT0000738 | miR-383-5p (miRNAs w/seed GAUCAGA) | 2.030 | TargetScan Human | Moderate (predicted) | C21orf62 | chromosome 21 open reading frame 62 | Other | other |
| MIMAT0000738 | miR-383-5p (miRNAs w/seed GAUCAGA) | 2.030 | TargetScan Human | Moderate (predicted) | C2orf72 | chromosome 2 open reading frame 72 | Other | other |
| MIMAT0000738 | miR-383-5p (miRNAs w/seed GAUCAGA) | 2.030 | TargetScan Human | Moderate (predicted) | C5 | complement C5 | Extracellular Space | cytokine |
| MIMAT0000738 | miR-383-5p (miRNAs w/seed GAUCAGA) | 2.030 | TargetScan Human | Moderate (predicted) | C5orf22 | chromosome 5 open reading frame 22 | Other | other |
| MIMAT0000738 | miR-383-5p (miRNAs w/seed GAUCAGA) | 2.030 | TargetScan Human | Moderate (predicted) | C5orf51 | chromosome 5 open reading frame 51 | Other | other |
| MIMAT0000738 | miR-383-5p (miRNAs w/seed GAUCAGA) | 2.030 | TargetScan Human | Moderate (predicted) | C7orf57 | chromosome 7 open reading frame 57 | Other | other |
| MIMAT0000738 | miR-383-5p (miRNAs w/seed GAUCAGA) | 2.030 | TargetScan Human | Moderate (predicted) | C8orf37 | chromosome 8 open reading frame 37 | Cytoplasm | other |
| MIMAT0000738 | miR-383-5p (miRNAs w/seed GAUCAGA) | 2.030 | TargetScan Human | Moderate (predicted) | CA3 | carbonic anhydrase 3 | Cytoplasm | enzyme |
| MIMAT0000738 | miR-383-5p (miRNAs w/seed GAUCAGA) | 2.030 | TargetScan Human | Moderate (predicted) | CA5B | carbonic anhydrase 5B | Cytoplasm | enzyme |
| MIMAT0000738 | miR-383-5p (miRNAs w/seed GAUCAGA) | 2.030 | TargetScan Human | Moderate (predicted) | CALCA | calcitonin related polypeptide alpha | Plasma Membrane | other |
| MIMAT0000738 | miR-383-5p (miRNAs w/seed GAUCAGA) | 2.030 | TargetScan Human | Moderate (predicted) | CALD1 | caldesmon 1 | Cytoplasm | other |
| MIMAT0000738 | miR-383-5p (miRNAs w/seed GAUCAGA) | 2.030 | TargetScan Human | Moderate (predicted) | CAMKK2 | m/calmodulin dependent protein kinase ki | Cytoplasm | kinase |
| MIMAT0000738 | miR-383-5p (miRNAs w/seed GAUCAGA) | 2.030 | TargetScan Human | High (predicted) | CAPNS2 | calpain small subunit 2 | Cytoplasm | peptidase |
| MIMAT0000738 | miR-383-5p (miRNAs w/seed GAUCAGA) | 2.030 | TargetScan Human | High (predicted) | CAPS2 | calcyphosine 2 | Other | other |
| MIMAT0000738 | miR-383-5p (miRNAs w/seed GAUCAGA) | 2.030 | TargetScan Human | Moderate (predicted) | CARF | calcium responsive transcription factor | Nucleus | transcription regulator |
| MIMAT0000738 | miR-383-5p (miRNAs w/seed GAUCAGA) | 2.030 | TargetScan Human | Moderate (predicted) | CASP2 | caspase 2 | Cytoplasm | peptidase |
| MIMAT0000738 | miR-383-5p (miRNAs w/seed GAUCAGA) | 2.030 | TargetScan Human | Moderate (predicted) | CBLB | Cbl proto-oncogene B | Nucleus | enzyme |
| MIMAT0000738 | miR-383-5p (miRNAs w/seed GAUCAGA) | 2.030 | TargetScan Human | Moderate (predicted) | CBLN3 | cerebellin 3 precursor | Extracellular Space | other |
| MIMAT0000738 | miR-383-5p (miRNAs w/seed GAUCAGA) | 2.030 | TargetScan Human | Moderate (predicted) | CBX5 | chromobox 5 | Nucleus | transcription regulator |
| MIMAT0000738 | miR-383-5p (miRNAs w/seed GAUCAGA) | 2.030 | TargetScan Human | Moderate (predicted) | CCDC103 | coiled-coil domain containing 103 | Cytoplasm | other |
| MIMAT0000738 | miR-383-5p (miRNAs w/seed GAUCAGA) | 2.030 | TargetScan Human | Moderate (predicted) | CCDC15 | coiled-coil domain containing 15 | Cytoplasm | other |
| MIMAT0000738 | miR-383-5p (miRNAs w/seed GAUCAGA) | 2.030 | TargetScan Human | Moderate (predicted) | CCDC28B | coiled-coil domain containing 28B | Cytoplasm | other |
| MIMAT0000738 | miR-383-5p (miRNAs w/seed GAUCAGA) | 2.030 | TargetScan Human | Moderate (predicted) | CCDC3 | coiled-coil domain containing 3 | Cytoplasm | other |
| MIMAT0000738 | miR-383-5p (miRNAs w/seed GAUCAGA) | 2.030 | TargetScan Human | High (predicted) | CCN2 | cellular communication network factor 2 | Extracellular Space | growth factor |
| MIMAT0000738 | miR-383-5p (miRNAs w/seed GAUCAGA) | 2.030 | TargetScan Human | Moderate (predicted) | CCNT2 | cyclin T2 | Nucleus | transcription regulator |
| MIMAT0000738 | miR-383-5p (miRNAs w/seed GAUCAGA) | 2.030 | TargetScan Human | Moderate (predicted) | CCR2 | C-C motif chemokine receptor 2 | Plasma Membrane | G-protein coupled receptor |
| MIMAT0000738 | miR-383-5p (miRNAs w/seed GAUCAGA) | 2.030 | TargetScan Human | Moderate (predicted) | CD274 | CD274 molecule | Plasma Membrane | enzyme |
| MIMAT0000738 | miR-383-5p (miRNAs w/seed GAUCAGA) | 2.030 | TargetScan Human | High (predicted) | CD28 | CD28 molecule | Plasma Membrane | transmembrane receptor |
| MIMAT0000738 | miR-383-5p (miRNAs w/seed GAUCAGA) | 2.030 | TargetScan Human | Moderate (predicted) | CD300LF | CD300 molecule like family member f | Plasma Membrane | other |
| MIMAT0000738 | miR-383-5p (miRNAs w/seed GAUCAGA) | 2.030 | TargetScan Human | High (predicted) | CDC42SE1 | CDC42 small effector 1 | Plasma Membrane | other |
| MIMAT0000738 | miR-383-5p (miRNAs w/seed GAUCAGA) | 2.030 | TargetScan Human | Moderate (predicted) | CDC42SE2 | CDC42 small effector 2 | Plasma Membrane | other |
| MIMAT0000738 | miR-383-5p (miRNAs w/seed GAUCAGA) | 2.030 | TargetScan Human | Moderate (predicted) | CDH12 | cadherin 12 | Plasma Membrane | other |
| MIMAT0000738 | miR-383-5p (miRNAs w/seed GAUCAGA) | 2.030 | TargetScan Human | Moderate (predicted) | CDIPT | diacylglycerol--inositol 3-phosphatidyltrans | Cytoplasm | enzyme |
| MIMAT0000738 | miR-383-5p (miRNAs w/seed GAUCAGA) | 2.030 | TargetScan Human | Moderate (predicted) | CDV3 | CDV3 homolog | Cytoplasm | other |
| MIMAT0000738 | miR-383-5p (miRNAs w/seed GAUCAGA) | 2.030 | TargetScan Human | Moderate (predicted) | CEACAM8 | CEA cell adhesion molecule 8 | Plasma Membrane | other |
| MIMAT0000738 | miR-383-5p (miRNAs w/seed GAUCAGA) | 2.030 | TargetScan Human | Moderate (predicted) | CELF6 | CUGBP Elav-like family member 6 | Cytoplasm | other |
| MIMAT0000738 | miR-383-5p (miRNAs w/seed GAUCAGA) | 2.030 | TargetScan Human | Moderate (predicted) | CENPF | centromere protein F | Nucleus | other |
| MIMAT0000738 | miR-383-5p (miRNAs w/seed GAUCAGA) | 2.030 | TargetScan Human | Moderate (predicted) | CEP97 | centrosomal protein 97 | Cytoplasm | other |
| MIMAT0000738 | miR-383-5p (miRNAs w/seed GAUCAGA) | 2.030 | TargetScan Human | Moderate (predicted) | CFAP161 | cilia and flagella associated protein 161 | Other | other |
| MIMAT0000738 | miR-383-5p (miRNAs w/seed GAUCAGA) | 2.030 | TargetScan Human | Moderate (predicted) | CFAP61 | cilia and flagella associated protein 61 | Cytoplasm | other |
| MIMAT0000738 | miR-383-5p (miRNAs w/seed GAUCAGA) | 2.030 | TargetScan Human | Moderate (predicted) | CHD6 | romodomain helicase DNA binding protei | Nucleus | enzyme |
| MIMAT0000738 | miR-383-5p (miRNAs w/seed GAUCAGA) | 2.030 | TargetScan Human | Moderate (predicted) | CHRM5 | cholinergic receptor muscarinic 5 | Plasma Membrane | G-protein coupled receptor |
| MIMAT0000738 | miR-383-5p (miRNAs w/seed GAUCAGA) | 2.030 | TargetScan Human | Moderate (predicted) | CHST10 | carbohydrate sulfotransferase 10 | Cytoplasm | enzyme |
| MIMAT0000738 | miR-383-5p (miRNAs w/seed GAUCAGA) | 2.030 | TargetScan Human | Moderate (predicted) | CHST14 | carbohydrate sulfotransferase 14 | Cytoplasm | enzyme |
| MIMAT0000738 | miR-383-5p (miRNAs w/seed GAUCAGA) | 2.030 | TargetScan Human | Moderate (predicted) | CIRBP | cold inducible RNA binding protein | Nucleus | translation regulator |
| MIMAT0000738 | miR-383-5p (miRNAs w/seed GAUCAGA) | 2.030 | TargetScan Human | High (predicted) | CLCN2 | chloride voltage-gated channel 2 | Plasma Membrane | ion channel |
| MIMAT0000738 | miR-383-5p (miRNAs w/seed GAUCAGA) | 2.030 | TargetScan Human | Moderate (predicted) | CLDN12 | claudin 12 | Plasma Membrane | other |
| MIMAT0000738 | miR-383-5p (miRNAs w/seed GAUCAGA) | 2.030 | TargetScan Human | Moderate (predicted) | CLDN2 | claudin 2 | Plasma Membrane | other |
| MIMAT0000738 | miR-383-5p (miRNAs w/seed GAUCAGA) | 2.030 | TargetScan Human | High (predicted) | CLEC3A | C-type lectin domain family 3 member A | Other | other |
| MIMAT0000738 | miR-383-5p (miRNAs w/seed GAUCAGA) | 2.030 | TargetScan Human | Moderate (predicted) | CLEC5A | C-type lectin domain containing 5A | Plasma Membrane | other |
| MIMAT0000738 | miR-383-5p (miRNAs w/seed GAUCAGA) | 2.030 | TargetScan Human | Moderate (predicted) | CLIC5 | chloride intracellular channel 5 | Cytoplasm | ion channel |
| MIMAT0000738 | miR-383-5p (miRNAs w/seed GAUCAGA) | 2.030 | TargetScan Human | Moderate (predicted) | CMTR2 | cap methyltransferase 2 | Other | enzyme |
| MIMAT0000738 | miR-383-5p (miRNAs w/seed GAUCAGA) | 2.030 | TargetScan Human | High (predicted) | CNIH1 | ichon family AMPA receptor auxiliary prot | Plasma Membrane | other |
| MIMAT0000738 | miR-383-5p (miRNAs w/seed GAUCAGA) | 2.030 | TargetScan Human | Moderate (predicted) | CNNM1 | CBS domain divalent metal cation transpor | Plasma Membrane | other |
| MIMAT0000738 | miR-383-5p (miRNAs w/seed GAUCAGA) | 2.030 | TargetScan Human | High (predicted) | CNOT4 | CCR4-NOT transcription complex subunit | Cytoplasm | enzyme |
| MIMAT0000738 | miR-383-5p (miRNAs w/seed GAUCAGA) | 2.030 | TargetScan Human | Moderate (predicted) | CNTD1 | cyclin N-terminal domain containing 1 | Other | other |
| MIMAT0000738 | miR-383-5p (miRNAs w/seed GAUCAGA) | 2.030 | TargetScan Human | Moderate (predicted) | CNTLN | centlein | Cytoplasm | other |
| MIMAT0000738 | miR-383-5p (miRNAs w/seed GAUCAGA) | 2.030 | TargetScan Human | Moderate (predicted) | COA5 | cytochrome c oxidase assembly factor 5 | Cytoplasm | other |
| MIMAT0000738 | miR-383-5p (miRNAs w/seed GAUCAGA) | 2.030 | TargetScan Human | Moderate (predicted) | COMMD7 | COMM domain containing 7 | Cytoplasm | other |
| MIMAT0000738 | miR-383-5p (miRNAs w/seed GAUCAGA) | 2.030 | TargetScan Human | Moderate (predicted) | CORO1C | coronin 1C | Cytoplasm | other |
| MIMAT0000738 | miR-383-5p (miRNAs w/seed GAUCAGA) | 2.030 | TargetScan Human | Moderate (predicted) | COX7B | cytochrome c oxidase subunit 7B | Cytoplasm | enzyme |
| MIMAT0000738 | miR-383-5p (miRNAs w/seed GAUCAGA) | 2.030 | TargetScan Human | Moderate (predicted) | CPB2 | carboxypeptidase B2 | Extracellular Space | peptidase |
| MIMAT0000738 | miR-383-5p (miRNAs w/seed GAUCAGA) | 2.030 | TargetScan Human | Moderate (predicted) | CPNE3 | copine 3 | Cytoplasm | kinase |
| MIMAT0000738 | miR-383-5p (miRNAs w/seed GAUCAGA) | 2.030 | TargetScan Human | Moderate (predicted) | CREG2 | ellular repressor of E1A stimulated genes | Extracellular Space | enzyme |
| MIMAT0000738 | miR-383-5p (miRNAs w/seed GAUCAGA) | 2.030 | TargetScan Human | Moderate (predicted) | CSPG5 | chondroitin sulfate proteoglycan 5 | Extracellular Space | growth factor |
| MIMAT0000738 | miR-383-5p (miRNAs w/seed GAUCAGA) | 2.030 | TargetScan Human | High (predicted) | CTNNAL1 | catenin alpha like 1 | Plasma Membrane | other |
| MIMAT0000738 | miR-383-5p (miRNAs w/seed GAUCAGA) | 2.030 | TargetScan Human | Moderate (predicted) | CTSB | cathepsin B | Cytoplasm | peptidase |
| MIMAT0000738 | miR-383-5p (miRNAs w/seed GAUCAGA) | 2.030 | TargetScan Human | High (predicted) | CTSF | cathepsin F | Cytoplasm | peptidase |
| MIMAT0000738 | miR-383-5p (miRNAs w/seed GAUCAGA) | 2.030 | TargetScan Human | Moderate (predicted) | CYP27B1 | chrome P450 family 27 subfamily B memb | Cytoplasm | enzyme |
| MIMAT0000738 | miR-383-5p (miRNAs w/seed GAUCAGA) | 2.030 | TargetScan Human | Moderate (predicted) | CYP2U1 | ochrome P450 family 2 subfamily U memb | Cytoplasm | enzyme |
| MIMAT0000738 | miR-383-5p (miRNAs w/seed GAUCAGA) | 2.030 | TargetScan Human | High (predicted) | CYP3A5 | ochrome P450 family 3 subfamily A memb | Cytoplasm | enzyme |
| MIMAT0000738 | miR-383-5p (miRNAs w/seed GAUCAGA) | 2.030 | TargetScan Human | Moderate (predicted) | CYP4F11 | chrome P450 family 4 subfamily F membe | Cytoplasm | enzyme |
| MIMAT0000738 | miR-383-5p (miRNAs w/seed GAUCAGA) | 2.030 | TargetScan Human | Moderate (predicted) | DACH2 | dachshund family transcription factor 2 | Nucleus | transcription regulator |
| MIMAT0000738 | miR-383-5p (miRNAs w/seed GAUCAGA) | 2.030 | TargetScan Human | Moderate (predicted) | DCHS2 | dachsous cadherin-related 2 | Extracellular Space | other |
| MIMAT0000738 | miR-383-5p (miRNAs w/seed GAUCAGA) | 2.030 | TargetScan Human | High (predicted) | DCTN3 | dynactin subunit 3 | Nucleus | other |
| MIMAT0000738 | miR-383-5p (miRNAs w/seed GAUCAGA) | 2.030 | TargetScan Human | Moderate (predicted) | DCTN4 | dynactin subunit 4 | Nucleus | other |
| MIMAT0000738 | miR-383-5p (miRNAs w/seed GAUCAGA) | 2.030 | TargetScan Human | Moderate (predicted) | DCUN1D4 | ctive in cullin neddylation 1 domain contair | Nucleus | other |
| MIMAT0000738 | miR-383-5p (miRNAs w/seed GAUCAGA) | 2.030 | TargetScan Human | Moderate (predicted) | DCX | doublecortin | Cytoplasm | other |
| MIMAT0000738 | miR-383-5p (miRNAs w/seed GAUCAGA) | 2.030 | TargetScan Human | Moderate (predicted) | DDI2 | DNA damage inducible 1 homolog 2 | Plasma Membrane | transporter |
| MIMAT0000738 | miR-383-5p (miRNAs w/seed GAUCAGA) | 2.030 | TargetScan Human | High (predicted) | DDIAS | DNA damage induced apoptosis suppress | Cytoplasm | other |
| MIMAT0000738 | miR-383-5p (miRNAs w/seed GAUCAGA) | 2.030 | TargetScan Human | High (predicted) | DDX21 | DExD-box helicase 21 | Nucleus | enzyme |
| MIMAT0000738 | miR-383-5p (miRNAs w/seed GAUCAGA) | 2.030 | TargetScan Human | Moderate (predicted) | DEDD2 | death effector domain containing 2 | Nucleus | other |
| MIMAT0000738 | miR-383-5p (miRNAs w/seed GAUCAGA) | 2.030 | TargetScan Human | Moderate (predicted) | DEPTOR | P domain containing MTOR interacting pro | Cytoplasm | other |
| MIMAT0000738 | miR-383-5p (miRNAs w/seed GAUCAGA) | 2.030 | TargetScan Human | High (predicted) | DGAT2L6 | diacylglycerol O-acyltransferase 2 like 6 | Cytoplasm | enzyme |
| MIMAT0000738 | miR-383-5p (miRNAs w/seed GAUCAGA) | 2.030 | TargetScan Human | Moderate (predicted) | DHRS12 | dehydrogenase/reductase 12 | Nucleus | other |
| MIMAT0000738 | miR-383-5p (miRNAs w/seed GAUCAGA) | 2.030 | TargetScan Human | High (predicted) | DHRS13 | dehydrogenase/reductase 13 | Other | enzyme |
| MIMAT0000738 | miR-383-5p (miRNAs w/seed GAUCAGA) | 2.030 | TargetScan Human | Moderate (predicted) | DHX36 | DEAH-box helicase 36 | Cytoplasm | enzyme |
| MIMAT0000738 | miR-383-5p (miRNAs w/seed GAUCAGA) | 2.030 | TargetScan Human | Moderate (predicted) | DIABLO | diablo IAP-binding mitochondrial protein | Cytoplasm | other |
| MIMAT0000738 | miR-383-5p (miRNAs w/seed GAUCAGA) | 2.030 | TargetScan Human | Moderate (predicted) | DIO1 | iodothyronine deiodinase 1 | Cytoplasm | enzyme |
| MIMAT0000738 | miR-383-5p (miRNAs w/seed GAUCAGA) | 2.030 | TargetScan Human | Moderate (predicted) | DIO2 | iodothyronine deiodinase 2 | Cytoplasm | enzyme |
| MIMAT0000738 | miR-383-5p (miRNAs w/seed GAUCAGA) | 2.030 | TargetScan Human | Moderate (predicted) | DMRTB1 | RT like family B with proline rich C-termin | Nucleus | transcription regulator |
| MIMAT0000738 | miR-383-5p (miRNAs w/seed GAUCAGA) | 2.030 | TargetScan Human | Moderate (predicted) | DNAH10OS | ein axonemal heavy chain 10 opposite str | Other | other |
| MIMAT0000738 | miR-383-5p (miRNAs w/seed GAUCAGA) | 2.030 | TargetScan Human | Moderate (predicted) | DNAJB14 | heat shock protein family (Hsp40) membe | Cytoplasm | enzyme |
| MIMAT0000738 | miR-383-5p (miRNAs w/seed GAUCAGA) | 2.030 | TargetScan Human | Moderate (predicted) | DPY19L3 | dpy-19 like C-mannosyltransferase 3 | Other | other |
| MIMAT0000738 | miR-383-5p (miRNAs w/seed GAUCAGA) | 2.030 | TargetScan Human | Moderate (predicted) | DR1 | down-regulator of transcription 1 | Nucleus | transcription regulator |
| MIMAT0000738 | miR-383-5p (miRNAs w/seed GAUCAGA) | 2.030 | TargetScan Human | Moderate (predicted) | DSC3 | desmocollin 3 | Plasma Membrane | other |
| MIMAT0000738 | miR-383-5p (miRNAs w/seed GAUCAGA) | 2.030 | TargetScan Human | Moderate (predicted) | DSG3 | desmoglein 3 | Plasma Membrane | other |
| MIMAT0000738 | miR-383-5p (miRNAs w/seed GAUCAGA) | 2.030 | TargetScan Human | Moderate (predicted) | DTHD1 | death domain containing 1 | Other | other |
| MIMAT0000738 | miR-383-5p (miRNAs w/seed GAUCAGA) | 2.030 | TargetScan Human | Moderate (predicted) | DUS4L | dihydrouridine synthase 4 like | Other | other |
| MIMAT0000738 | miR-383-5p (miRNAs w/seed GAUCAGA) | 2.030 | TargetScan Human | Moderate (predicted) | E2F7 | E2F transcription factor 7 | Nucleus | transcription regulator |
| MIMAT0000738 | miR-383-5p (miRNAs w/seed GAUCAGA) | 2.030 | TargetScan Human | Moderate (predicted) | EBF1 | EBF transcription factor 1 | Nucleus | transcription regulator |
| MIMAT0000738 | miR-383-5p (miRNAs w/seed GAUCAGA) | 2.030 | TargetScan Human | Moderate (predicted) | EDA2R | ectodysplasin A2 receptor | Plasma Membrane | transmembrane receptor |
| MIMAT0000738 | miR-383-5p (miRNAs w/seed GAUCAGA) | 2.030 | TargetScan Human | Moderate (predicted) | EDARADD | EDAR associated death domain | Cytoplasm | other |
| MIMAT0000738 | miR-383-5p (miRNAs w/seed GAUCAGA) | 2.030 | TargetScan Human | Moderate (predicted) | EDN1 | endothelin 1 | Extracellular Space | cytokine |
| MIMAT0000738 | miR-383-5p (miRNAs w/seed GAUCAGA) | 2.030 | TargetScan Human | Moderate (predicted) | EDNRA | endothelin receptor type A | Plasma Membrane | transmembrane receptor |
| MIMAT0000738 | miR-383-5p (miRNAs w/seed GAUCAGA) | 2.030 | TargetScan Human | Moderate (predicted) | EEF1AKMT3 | EEF1A lysine methyltransferase 3 | Cytoplasm | enzyme |
| MIMAT0000738 | miR-383-5p (miRNAs w/seed GAUCAGA) | 2.030 | TargetScan Human | Moderate (predicted) | EEF2K | eukaryotic elongation factor 2 kinase | Cytoplasm | kinase |
| MIMAT0000738 | miR-383-5p (miRNAs w/seed GAUCAGA) | 2.030 | TargetScan Human | Moderate (predicted) | EIF4B | eukaryotic translation initiation factor 4B | Cytoplasm | translation regulator |
| MIMAT0000738 | miR-383-5p (miRNAs w/seed GAUCAGA) | 2.030 | TargetScan Human | Moderate (predicted) | EIF4G2 | karyotic translation initiation factor 4 gamm | Cytoplasm | translation regulator |
| MIMAT0000738 | miR-383-5p (miRNAs w/seed GAUCAGA) | 2.030 | TargetScan Human | Moderate (predicted) | ELL2 | elongation factor for RNA polymerase II 2 | Nucleus | transcription regulator |
| MIMAT0000738 | miR-383-5p (miRNAs w/seed GAUCAGA) | 2.030 | TargetScan Human | High (predicted) | ELMOD2 | ELMO domain containing 2 | Other | other |
| MIMAT0000738 | miR-383-5p (miRNAs w/seed GAUCAGA) | 2.030 | TargetScan Human | Moderate (predicted) | EMCN | endomucin | Extracellular Space | other |
| MIMAT0000738 | miR-383-5p (miRNAs w/seed GAUCAGA) | 2.030 | TargetScan Human | Moderate (predicted) | EPAS1 | endothelial PAS domain protein 1 | Nucleus | transcription regulator |
| MIMAT0000738 | miR-383-5p (miRNAs w/seed GAUCAGA) | 2.030 | TargetScan Human | Moderate (predicted) | EPG5 | opic P-granules autophagy protein 5 homo | Cytoplasm | other |
| MIMAT0000738 | miR-383-5p (miRNAs w/seed GAUCAGA) | 2.030 | TargetScan Human | Moderate (predicted) | EPM2A | EPM2A glucan phosphatase, laforin | Cytoplasm | phosphatase |
| MIMAT0000738 | miR-383-5p (miRNAs w/seed GAUCAGA) | 2.030 | TargetScan Human | Moderate (predicted) | EPM2AIP1 | EPM2A interacting protein 1 | Cytoplasm | other |
| MIMAT0000738 | miR-383-5p (miRNAs w/seed GAUCAGA) | 2.030 | TargetScan Human | Moderate (predicted) | ERBB4 | erb-b2 receptor tyrosine kinase 4 | Plasma Membrane | kinase |
| MIMAT0000738 | miR-383-5p (miRNAs w/seed GAUCAGA) | 2.030 | TargetScan Human | Moderate (predicted) | ERMP1 | endoplasmic reticulum metallopeptidase 1 | Cytoplasm | other |
| MIMAT0000738 | miR-383-5p (miRNAs w/seed GAUCAGA) | 2.030 | TargetScan Human | High (predicted) | EXO1 | exonuclease 1 | Nucleus | enzyme |
| MIMAT0000738 | miR-383-5p (miRNAs w/seed GAUCAGA) | 2.030 | TargetScan Human | Moderate (predicted) | FABP7 | fatty acid binding protein 7 | Cytoplasm | transporter |
| MIMAT0000738 | miR-383-5p (miRNAs w/seed GAUCAGA) | 2.030 | TargetScan Human | Moderate (predicted) | FAM117B | mily with sequence similarity 117 member | Other | other |
| MIMAT0000738 | miR-383-5p (miRNAs w/seed GAUCAGA) | 2.030 | TargetScan Human | Moderate (predicted) | FAM120B | family with sequence similarity 120B | Nucleus | other |
| MIMAT0000738 | miR-383-5p (miRNAs w/seed GAUCAGA) | 2.030 | TargetScan Human | Moderate (predicted) | FAM126B | mily with sequence similarity 126 member | Other | other |
| MIMAT0000738 | miR-383-5p (miRNAs w/seed GAUCAGA) | 2.030 | TargetScan Human | Moderate (predicted) | FAM155A | mily with sequence similarity 155 member | Other | other |
| MIMAT0000738 | miR-383-5p (miRNAs w/seed GAUCAGA) | 2.030 | TargetScan Human | Moderate (predicted) | FAM163A | mily with sequence similarity 163 member | Other | other |

Sup. Table 3. List of 3699 targets of the seven dysregulated miRNAs obtained from three different target prediction databases,TargetScan TarBase and miRecords considering those predicted as moderate, high, or experimentally validated, respectively.

| | | | | | | | | |
|---|---|---|---|---|---|---|---|---|
| MIMAT0000738 | miR-383-5p (miRNAs w/seed GAUCAGA) | 2.030 | TargetScan Human | Moderate (predicted) | FAM174A | mily with sequence similarity 174 member | Extracellular Space | other |
| MIMAT0000738 | miR-383-5p (miRNAs w/seed GAUCAGA) | 2.030 | TargetScan Human | Moderate (predicted) | FAM177B | mily with sequence similarity 177 member | Other | other |
| MIMAT0000738 | miR-383-5p (miRNAs w/seed GAUCAGA) | 2.030 | TargetScan Human | Moderate (predicted) | FAM216B | mily with sequence similarity 216 member | Other | other |
| MIMAT0000738 | miR-383-5p (miRNAs w/seed GAUCAGA) | 2.030 | TargetScan Human | Moderate (predicted) | FAM220A | mily with sequence similarity 220 member | Nucleus | other |
| MIMAT0000738 | miR-383-5p (miRNAs w/seed GAUCAGA) | 2.030 | TargetScan Human | Moderate (predicted) | M230E (includes othe | mily with sequence similarity 230 member | Extracellular Space | other |
| MIMAT0000738 | miR-383-5p (miRNAs w/seed GAUCAGA) | 2.030 | TargetScan Human | Moderate (predicted) | FAM43B | amily with sequence similarity 43 member | Other | other |
| MIMAT0000738 | miR-383-5p (miRNAs w/seed GAUCAGA) | 2.030 | TargetScan Human | Moderate (predicted) | FASLG | Fas ligand | Extracellular Space | cytokine |
| MIMAT0000738 | miR-383-5p (miRNAs w/seed GAUCAGA) | 2.030 | TargetScan Human | Moderate (predicted) | FBXO5 | F-box protein 5 | Nucleus | enzyme |
| MIMAT0000738 | miR-383-5p (miRNAs w/seed GAUCAGA) | 2.030 | TargetScan Human | Moderate (predicted) | FGF13 | fibroblast growth factor 13 | Extracellular Space | growth factor |
| MIMAT0000738 | miR-383-5p (miRNAs w/seed GAUCAGA) | 2.030 | TargetScan Human | High (predicted) | FGF19 | fibroblast growth factor 19 | Extracellular Space | growth factor |
| MIMAT0000738 | miR-383-5p (miRNAs w/seed GAUCAGA) | 2.030 | TargetScan Human | Moderate (predicted) | FGF7 | fibroblast growth factor 7 | Extracellular Space | growth factor |
| MIMAT0000738 | miR-383-5p (miRNAs w/seed GAUCAGA) | 2.030 | TargetScan Human | High (predicted) | FGF9 | fibroblast growth factor 9 | Extracellular Space | growth factor |
| MIMAT0000738 | miR-383-5p (miRNAs w/seed GAUCAGA) | 2.030 | TargetScan Human | Moderate (predicted) | FHL5 | four and a half LIM domains 5 | Nucleus | transcription regulator |
| MIMAT0000738 | miR-383-5p (miRNAs w/seed GAUCAGA) | 2.030 | TargetScan Human | Moderate (predicted) | FLRT2 | onectin leucine rich transmembrane prote | Plasma Membrane | other |
| MIMAT0000738 | miR-383-5p (miRNAs w/seed GAUCAGA) | 2.030 | TargetScan Human | Moderate (predicted) | FOS | roto-oncogene, AP-1 transcription factor s | Nucleus | transcription regulator |
| MIMAT0000738 | miR-383-5p (miRNAs w/seed GAUCAGA) | 2.030 | TargetScan Human | Moderate (predicted) | FOXN3 | forkhead box N3 | Nucleus | transcription regulator |
| MIMAT0000738 | miR-383-5p (miRNAs w/seed GAUCAGA) | 2.030 | TargetScan Human | Moderate (predicted) | FOXO3 | forkhead box O3 | Nucleus | transcription regulator |
| MIMAT0000738 | miR-383-5p (miRNAs w/seed GAUCAGA) | 2.030 | TargetScan Human | Moderate (predicted) | FSBP | fibrinogen silencer binding protein | Nucleus | other |
| MIMAT0000738 | miR-383-5p (miRNAs w/seed GAUCAGA) | 2.030 | TargetScan Human | Moderate (predicted) | FSTL1 | follistatin like 1 | Extracellular Space | other |
| MIMAT0000738 | miR-383-5p (miRNAs w/seed GAUCAGA) | 2.030 | TargetScan Human | Moderate (predicted) | FUBP3 | far upstream element binding protein 3 | Nucleus | transcription regulator |
| MIMAT0000738 | miR-383-5p (miRNAs w/seed GAUCAGA) | 2.030 | TargetScan Human | Moderate (predicted) | FUS | FUS RNA binding protein | Nucleus | transcription regulator |
| MIMAT0000738 | miR-383-5p (miRNAs w/seed GAUCAGA) | 2.030 | TargetScan Human | Moderate (predicted) | FUT1 | fucosyltransferase 1 (H blood group) | Cytoplasm | enzyme |
| MIMAT0000738 | miR-383-5p (miRNAs w/seed GAUCAGA) | 2.030 | TargetScan Human | Moderate (predicted) | FUT9 | fucosyltransferase 9 | Cytoplasm | enzyme |
| MIMAT0000738 | miR-383-5p (miRNAs w/seed GAUCAGA) | 2.030 | TargetScan Human | High (predicted) | FXYD4 | YD domain containing ion transport regulat | Plasma Membrane | ion channel |
| MIMAT0000738 | miR-383-5p (miRNAs w/seed GAUCAGA) | 2.030 | TargetScan Human | Moderate (predicted) | FYTTD1 | forty-two-three domain containing 1 | Nucleus | other |
| MIMAT0000738 | miR-383-5p (miRNAs w/seed GAUCAGA) | 2.030 | TargetScan Human | Moderate (predicted) | GAB1 | GRB2 associated binding protein 1 | Cytoplasm | other |
| MIMAT0000738 | miR-383-5p (miRNAs w/seed GAUCAGA) | 2.030 | TargetScan Human | Moderate (predicted) | GABRA2 | -aminobutyric acid type A receptor subuni | Plasma Membrane | ion channel |
| MIMAT0000738 | miR-383-5p (miRNAs w/seed GAUCAGA) | 2.030 | TargetScan Human | Moderate (predicted) | GABRA4 | -aminobutyric acid type A receptor subuni | Plasma Membrane | ion channel |
| MIMAT0000738 | miR-383-5p (miRNAs w/seed GAUCAGA) | 2.030 | TargetScan Human | Moderate (predicted) | GALNT1 | ypeptide N-acetylgalactosaminyltransferas | Cytoplasm | enzyme |
| MIMAT0000738 | miR-383-5p (miRNAs w/seed GAUCAGA) | 2.030 | TargetScan Human | Moderate (predicted) | GALNT10 | ypeptide N-acetylgalactosaminyltransferas | Cytoplasm | enzyme |
| MIMAT0000738 | miR-383-5p (miRNAs w/seed GAUCAGA) | 2.030 | TargetScan Human | High (predicted) | GALNT11 | ypeptide N-acetylgalactosaminyltransferas | Cytoplasm | enzyme |
| MIMAT0000738 | miR-383-5p (miRNAs w/seed GAUCAGA) | 2.030 | TargetScan Human | High (predicted) | GALNT13 | ypeptide N-acetylgalactosaminyltransferas | Cytoplasm | enzyme |
| MIMAT0000738 | miR-383-5p (miRNAs w/seed GAUCAGA) | 2.030 | TargetScan Human | Moderate (predicted) | GAREM1 | RB2 associated regulator of MAPK1 subtyp | Nucleus | other |
| MIMAT0000738 | miR-383-5p (miRNAs w/seed GAUCAGA) | 2.030 | TargetScan Human | Moderate (predicted) | GAS1 | growth arrest specific 1 | Plasma Membrane | other |
| MIMAT0000738 | miR-383-5p (miRNAs w/seed GAUCAGA) | 2.030 | TargetScan Human | High (predicted) | GAS2L3 | growth arrest specific 2 like 3 | Other | other |
| MIMAT0000738 | miR-383-5p (miRNAs w/seed GAUCAGA) | 2.030 | TargetScan Human | Moderate (predicted) | GAST | gastrin | Extracellular Space | other |
| MIMAT0000738 | miR-383-5p (miRNAs w/seed GAUCAGA) | 2.030 | TargetScan Human | High (predicted) | GATA6 | GATA binding protein 6 | Nucleus | transcription regulator |
| MIMAT0000738 | miR-383-5p (miRNAs w/seed GAUCAGA) | 2.030 | TargetScan Human | Moderate (predicted) | GATD3A/GATD3B | amidotransferase like class 1 domain con | Cytoplasm | other |
| MIMAT0000738 | miR-383-5p (miRNAs w/seed GAUCAGA) | 2.030 | TargetScan Human | Moderate (predicted) | GBP2 | guanylate binding protein 2 | Cytoplasm | enzyme |
| MIMAT0000738 | miR-383-5p (miRNAs w/seed GAUCAGA) | 2.030 | TargetScan Human | Moderate (predicted) | GCNT4 | glucosaminyl (N-acetyl) transferase 4 | Cytoplasm | enzyme |
| MIMAT0000738 | miR-383-5p (miRNAs w/seed GAUCAGA) | 2.030 | TargetScan Human | Moderate (predicted) | GDE1 | lycerophosphodiester phosphodiesterase | Plasma Membrane | enzyme |
| MIMAT0000738 | miR-383-5p (miRNAs w/seed GAUCAGA) | 2.030 | TargetScan Human | Moderate (predicted) | GDNF | glial cell derived neurotrophic factor | Extracellular Space | growth factor |
| MIMAT0000738 | miR-383-5p (miRNAs w/seed GAUCAGA) | 2.030 | TargetScan Human | Moderate (predicted) | GEMIN5 | gem nuclear organelle associated protein 5 | Nucleus | other |
| MIMAT0000738 | miR-383-5p (miRNAs w/seed GAUCAGA) | 2.030 | TargetScan Human | Moderate (predicted) | GFPT2 | tamine-fructose-6-phosphate transaminas | Cytoplasm | enzyme |
| MIMAT0000738 | miR-383-5p (miRNAs w/seed GAUCAGA) | 2.030 | TargetScan Human | Moderate (predicted) | GJA5 | gap junction protein alpha 5 | Plasma Membrane | transporter |
| MIMAT0000738 | miR-383-5p (miRNAs w/seed GAUCAGA) | 2.030 | TargetScan Human | Moderate (predicted) | GLDN | gliomedin | Cytoplasm | other |
| MIMAT0000738 | miR-383-5p (miRNAs w/seed GAUCAGA) | 2.030 | TargetScan Human | Moderate (predicted) | GLRB | glycine receptor beta | Plasma Membrane | ion channel |
| MIMAT0000738 | miR-383-5p (miRNAs w/seed GAUCAGA) | 2.030 | TargetScan Human | Moderate (predicted) | GLRX5 | glutaredoxin 5 | Cytoplasm | other |
| MIMAT0000738 | miR-383-5p (miRNAs w/seed GAUCAGA) | 2.030 | TargetScan Human | Moderate (predicted) | GMCL1 | erm cell-less 1, spermatogenesis associate | Nucleus | other |
| MIMAT0000738 | miR-383-5p (miRNAs w/seed GAUCAGA) | 2.030 | TargetScan Human | Moderate (predicted) | GMNC | geminin coiled-coil domain containing | Nucleus | other |
| MIMAT0000738 | miR-383-5p (miRNAs w/seed GAUCAGA) | 2.030 | TargetScan Human | Moderate (predicted) | GNPDA2 | glucosamine-6-phosphate deaminase 2 | Nucleus | enzyme |
| MIMAT0000738 | miR-383-5p (miRNAs w/seed GAUCAGA) | 2.030 | TargetScan Human | Moderate (predicted) | GP1BB | glycoprotein Ib platelet subunit beta | Plasma Membrane | other |
| MIMAT0000738 | miR-383-5p (miRNAs w/seed GAUCAGA) | 2.030 | TargetScan Human | Moderate (predicted) | GPALPP1 | GPALPP motifs containing 1 | Other | other |
| MIMAT0000738 | miR-383-5p (miRNAs w/seed GAUCAGA) | 2.030 | TargetScan Human | High (predicted) | GPD1L | glycerol-3-phosphate dehydrogenase 1 like | Cytoplasm | enzyme |
| MIMAT0000738 | miR-383-5p (miRNAs w/seed GAUCAGA) | 2.030 | TargetScan Human | Moderate (predicted) | GPR61 | G protein-coupled receptor 61 | Plasma Membrane | G-protein coupled receptor |
| MIMAT0000738 | miR-383-5p (miRNAs w/seed GAUCAGA) | 2.030 | TargetScan Human | Moderate (predicted) | GPR89A/GPR89B | G protein-coupled receptor 89B | Cytoplasm | ion channel |
| MIMAT0000738 | miR-383-5p (miRNAs w/seed GAUCAGA) | 2.030 | TargetScan Human | Moderate (predicted) | GPX6 | glutathione peroxidase 6 | Extracellular Space | enzyme |
| MIMAT0000738 | miR-383-5p (miRNAs w/seed GAUCAGA) | 2.030 | TargetScan Human | Moderate (predicted) | GRIN2B | mate ionotropic receptor NMDA type subu | Plasma Membrane | ion channel |
| MIMAT0000738 | miR-383-5p (miRNAs w/seed GAUCAGA) | 2.030 | TargetScan Human | Moderate (predicted) | GRWD1 | glutamate rich WD repeat containing 1 | Nucleus | other |
| MIMAT0000738 | miR-383-5p (miRNAs w/seed GAUCAGA) | 2.030 | TargetScan Human | Moderate (predicted) | GSTK1 | glutathione S-transferase kappa 1 | Cytoplasm | enzyme |
| MIMAT0000738 | miR-383-5p (miRNAs w/seed GAUCAGA) | 2.030 | TargetScan Human | Moderate (predicted) | GTF2E1 | general transcription factor IIE subunit 1 | Nucleus | transcription regulator |
| MIMAT0000738 | miR-383-5p (miRNAs w/seed GAUCAGA) | 2.030 | TargetScan Human | Moderate (predicted) | GVQW3 | GVQW motif containing 3 | Other | other |
| MIMAT0000738 | miR-383-5p (miRNAs w/seed GAUCAGA) | 2.030 | TargetScan Human | Moderate (predicted) | GYPA | glycophorin A (MNS blood group) | Plasma Membrane | other |
| MIMAT0000738 | miR-383-5p (miRNAs w/seed GAUCAGA) | 2.030 | TargetScan Human | Moderate (predicted) | H2AW | H2A.W histone | Nucleus | other |
| MIMAT0000738 | miR-383-5p (miRNAs w/seed GAUCAGA) | 2.030 | TargetScan Human | High (predicted) | HACD2 | 3-hydroxyacyl-CoA dehydratase 2 | Cytoplasm | phosphatase |
| MIMAT0000738 | miR-383-5p (miRNAs w/seed GAUCAGA) | 2.030 | TargetScan Human | Moderate (predicted) | HAS3 | hyaluronan synthase 3 | Plasma Membrane | enzyme |
| MIMAT0000738 | miR-383-5p (miRNAs w/seed GAUCAGA) | 2.030 | TargetScan Human | Moderate (predicted) | HAVCR1 | hepatitis A virus cellular receptor 1 | Plasma Membrane | other |
| MIMAT0000738 | miR-383-5p (miRNAs w/seed GAUCAGA) | 2.030 | TargetScan Human | Moderate (predicted) | HCAR1 | hydroxycarboxylic acid receptor 1 | Plasma Membrane | G-protein coupled receptor |
| MIMAT0000738 | miR-383-5p (miRNAs w/seed GAUCAGA) | 2.030 | TargetScan Human | High (predicted) | HDDC3 | HD domain containing 3 | Other | other |
| MIMAT0000738 | miR-383-5p (miRNAs w/seed GAUCAGA) | 2.030 | TargetScan Human | Moderate (predicted) | HELLS | helicase, lymphoid specific | Nucleus | enzyme |
| MIMAT0000738 | miR-383-5p (miRNAs w/seed GAUCAGA) | 2.030 | TargetScan Human | High (predicted) | HHLA2 | HERV-H LTR-associating 2 | Other | other |
| MIMAT0000738 | miR-383-5p (miRNAs w/seed GAUCAGA) | 2.030 | TargetScan Human | Moderate (predicted) | HIPK2 | homeodomain interacting protein kinase 2 | Nucleus | kinase |
| MIMAT0000738 | miR-383-5p (miRNAs w/seed GAUCAGA) | 2.030 | TargetScan Human | Moderate (predicted) | HLA-DOA | r histocompatibility complex, class II, DO a | Plasma Membrane | transmembrane receptor |
| MIMAT0000738 | miR-383-5p (miRNAs w/seed GAUCAGA) | 2.030 | TargetScan Human | Moderate (predicted) | HMGN2 | mobility group nucleosomal binding doma | Nucleus | other |
| MIMAT0000738 | miR-383-5p (miRNAs w/seed GAUCAGA) | 2.030 | TargetScan Human | Moderate (predicted) | HNRNPH1 | eterogeneous nuclear ribonucleoprotein H | Nucleus | other |
| MIMAT0000738 | miR-383-5p (miRNAs w/seed GAUCAGA) | 2.030 | TargetScan Human | High (predicted) | HNRNPH2 | eterogeneous nuclear ribonucleoprotein H | Nucleus | other |
| MIMAT0000738 | miR-383-5p (miRNAs w/seed GAUCAGA) | 2.030 | TargetScan Human | Moderate (predicted) | HOOK1 | hook microtubule tethering protein 1 | Cytoplasm | other |
| MIMAT0000738 | miR-383-5p (miRNAs w/seed GAUCAGA) | 2.030 | TargetScan Human | Moderate (predicted) | HOXD9 | homeobox D9 | Nucleus | transcription regulator |
| MIMAT0000738 | miR-383-5p (miRNAs w/seed GAUCAGA) | 2.030 | TargetScan Human | Moderate (predicted) | HPGD | 15-hydroxyprostaglandin dehydrogenase | Cytoplasm | enzyme |
| MIMAT0000738 | miR-383-5p (miRNAs w/seed GAUCAGA) | 2.030 | TargetScan Human | High (predicted) | HS3ST3B1 | ran sulfate-glucosamine 3-sulfotransferase | Cytoplasm | enzyme |
| MIMAT0000738 | miR-383-5p (miRNAs w/seed GAUCAGA) | 2.030 | TargetScan Human | Moderate (predicted) | HSPE1-MOB4 | HSPE1-MOB4 readthrough | Other | other |
| MIMAT0000738 | miR-383-5p (miRNAs w/seed GAUCAGA) | 2.030 | TargetScan Human | Moderate (predicted) | HTN3 | histatin 3 | Extracellular Space | other |
| MIMAT0000738 | miR-383-5p (miRNAs w/seed GAUCAGA) | 2.030 | TargetScan Human | Moderate (predicted) | HTR2C | 5-hydroxytryptamine receptor 2C | Plasma Membrane | G-protein coupled receptor |
| MIMAT0000738 | miR-383-5p (miRNAs w/seed GAUCAGA) | 2.030 | TargetScan Human | Moderate (predicted) | HTR3C | 5-hydroxytryptamine receptor 3C | Plasma Membrane | ion channel |
| MIMAT0000738 | miR-383-5p (miRNAs w/seed GAUCAGA) | 2.030 | TargetScan Human | Moderate (predicted) | HTR3E | 5-hydroxytryptamine receptor 3E | Plasma Membrane | transmembrane receptor |
| MIMAT0000738 | miR-383-5p (miRNAs w/seed GAUCAGA) | 2.030 | TargetScan Human | Moderate (predicted) | HUS1 | HUS1 checkpoint clamp component | Nucleus | kinase |
| MIMAT0000738 | miR-383-5p (miRNAs w/seed GAUCAGA) | 2.030 | TargetScan Human | High (predicted) | IARS1 | isoleucyl-tRNA synthetase 1 | Cytoplasm | enzyme |
| MIMAT0000738 | miR-383-5p (miRNAs w/seed GAUCAGA) | 2.030 | TargetScan Human | Moderate (predicted) | IFI30 | IFI30 lysosomal thiol reductase | Cytoplasm | enzyme |
| MIMAT0000738 | miR-383-5p (miRNAs w/seed GAUCAGA) | 2.030 | TargetScan Human | Moderate (predicted) | IFI44L | interferon induced protein 44 like | Nucleus | other |
| MIMAT0000738 | miR-383-5p (miRNAs w/seed GAUCAGA) | 2.030 | TargetScan Human | High (predicted) | IGLL1/IGLL5 | immunoglobulin lambda like polypeptide 1 | Plasma Membrane | other |
| MIMAT0000738 | miR-383-5p (miRNAs w/seed GAUCAGA) | 2.030 | TargetScan Human | Moderate (predicted) | IGSF1 | immunoglobulin superfamily member 1 | Plasma Membrane | other |
| MIMAT0000738 | miR-383-5p (miRNAs w/seed GAUCAGA) | 2.030 | TargetScan Human | High (predicted) | IL12A | interleukin 12A | Extracellular Space | cytokine |
| MIMAT0000738 | miR-383-5p (miRNAs w/seed GAUCAGA) | 2.030 | TargetScan Human | Moderate (predicted) | IL17A | interleukin 17A | Extracellular Space | cytokine |
| MIMAT0000738 | miR-383-5p (miRNAs w/seed GAUCAGA) | 2.030 | TargetScan Human | High (predicted) | IL17RD | interleukin 17 receptor D | Cytoplasm | other |
| MIMAT0000738 | miR-383-5p (miRNAs w/seed GAUCAGA) | 2.030 | TargetScan Human | Moderate (predicted) | IL17REL | interleukin 17 receptor E like | Other | other |
| MIMAT0000738 | miR-383-5p (miRNAs w/seed GAUCAGA) | 2.030 | TargetScan Human | High (predicted) | IMPG1 | interphotoreceptor matrix proteoglycan 1 | Extracellular Space | other |
| MIMAT0000738 | miR-383-5p (miRNAs w/seed GAUCAGA) | 2.030 | TargetScan Human | Moderate (predicted) | IPO5 | importin 5 | Nucleus | transporter |
| MIMAT0000738 | miR-383-5p (miRNAs w/seed GAUCAGA) | 2.030 | TargetScan Human | Moderate (predicted) | IPO7 | importin 7 | Nucleus | transporter |
| MIMAT0000738 | miR-383-5p (miRNAs w/seed GAUCAGA) | 2.030 | TargetScan Human | High (predicted) | IQCJ | IQ motif containing J | Other | other |
| MIMAT0000738 | miR-383-5p (miRNAs w/seed GAUCAGA) | 2.030 | TargetScan Human | Moderate (predicted) | IRF1 | interferon regulatory factor 1 | Nucleus | transcription regulator |
| MIMAT0000738 | miR-383-5p (miRNAs w/seed GAUCAGA) | 2.030 | TargetScan Human | Moderate (predicted) | ITGB1BP1 | integrin subunit beta 1 binding protein 1 | Plasma Membrane | other |
| MIMAT0000738 | miR-383-5p (miRNAs w/seed GAUCAGA) | 2.030 | TargetScan Human | Moderate (predicted) | ITGB3BP | integrin subunit beta 3 binding protein | Nucleus | other |
| MIMAT0000738 | miR-383-5p (miRNAs w/seed GAUCAGA) | 2.030 | TargetScan Human | Moderate (predicted) | ITM2C | integral membrane protein 2C | Cytoplasm | other |
| MIMAT0000738 | miR-383-5p (miRNAs w/seed GAUCAGA) | 2.030 | TargetScan Human | High (predicted) | JRKL | JRK like | Nucleus | other |
| MIMAT0000738 | miR-383-5p (miRNAs w/seed GAUCAGA) | 2.030 | TargetScan Human | Moderate (predicted) | KANK2 | KN motif and ankyrin repeat domains 2 | Nucleus | transcription regulator |
| MIMAT0000738 | miR-383-5p (miRNAs w/seed GAUCAGA) | 2.030 | TargetScan Human | High (predicted) | KANSL2 | KAT8 regulatory NSL complex subunit 2 | Nucleus | other |
| MIMAT0000738 | miR-383-5p (miRNAs w/seed GAUCAGA) | 2.030 | TargetScan Human | Moderate (predicted) | KBTBD4 | kelch repeat and BTB domain containing 4 | Other | other |
| MIMAT0000738 | miR-383-5p (miRNAs w/seed GAUCAGA) | 2.030 | TargetScan Human | Moderate (predicted) | KCNAB1 | gated channel subfamily A member regula | Plasma Membrane | ion channel |
| MIMAT0000738 | miR-383-5p (miRNAs w/seed GAUCAGA) | 2.030 | TargetScan Human | Moderate (predicted) | KCNE1 | oltage-gated channel subfamily E regulato | Plasma Membrane | ion channel |
| MIMAT0000738 | miR-383-5p (miRNAs w/seed GAUCAGA) | 2.030 | TargetScan Human | Moderate (predicted) | KCNE4 | oltage-gated channel subfamily E regulato | Plasma Membrane | ion channel |
| MIMAT0000738 | miR-383-5p (miRNAs w/seed GAUCAGA) | 2.030 | TargetScan Human | Moderate (predicted) | KCNG3 | voltage-gated channel modifier subfamily G | Cytoplasm | ion channel |
| MIMAT0000738 | miR-383-5p (miRNAs w/seed GAUCAGA) | 2.030 | TargetScan Human | Moderate (predicted) | KCNJ10 | inwardly rectifying channel subfamily J m | Plasma Membrane | ion channel |
| MIMAT0000738 | miR-383-5p (miRNAs w/seed GAUCAGA) | 2.030 | TargetScan Human | Moderate (predicted) | KCNJ6 | m inwardly rectifying channel subfamily J r | Plasma Membrane | ion channel |
| MIMAT0000738 | miR-383-5p (miRNAs w/seed GAUCAGA) | 2.030 | TargetScan Human | Moderate (predicted) | KCNK13 | m two pore domain channel subfamily K m | Plasma Membrane | ion channel |
| MIMAT0000738 | miR-383-5p (miRNAs w/seed GAUCAGA) | 2.030 | TargetScan Human | Moderate (predicted) | KCNN3 | m calcium-activated channel subfamily N r | Plasma Membrane | ion channel |
| MIMAT0000738 | miR-383-5p (miRNAs w/seed GAUCAGA) | 2.030 | TargetScan Human | Moderate (predicted) | KCTD4 | ium channel tetramerization domain conta | Other | ion channel |
| MIMAT0000738 | miR-383-5p (miRNAs w/seed GAUCAGA) | 2.030 | TargetScan Human | Moderate (predicted) | KDELR3 | endoplasmic reticulum protein retention rec | Cytoplasm | transporter |
| MIMAT0000738 | miR-383-5p (miRNAs w/seed GAUCAGA) | 2.030 | TargetScan Human | Moderate (predicted) | KDF1 | keratinocyte differentiation factor 1 | Plasma Membrane | other |
| MIMAT0000738 | miR-383-5p (miRNAs w/seed GAUCAGA) | 2.030 | TargetScan Human | Moderate (predicted) | KDM8 | lysine demethylase 8 | Nucleus | enzyme |
| MIMAT0000738 | miR-383-5p (miRNAs w/seed GAUCAGA) | 2.030 | TargetScan Human | High (predicted) | KIAA0087 | KIAA0087 lncRNA | Other | other |
| MIMAT0000738 | miR-383-5p (miRNAs w/seed GAUCAGA) | 2.030 | TargetScan Human | Moderate (predicted) | KIAA0100 | KIAA0100 | Extracellular Space | other |
| MIMAT0000738 | miR-383-5p (miRNAs w/seed GAUCAGA) | 2.030 | TargetScan Human | Moderate (predicted) | KIAA1549 | KIAA1549 | Plasma Membrane | other |
| MIMAT0000738 | miR-383-5p (miRNAs w/seed GAUCAGA) | 2.030 | TargetScan Human | Moderate (predicted) | KIF5B | kinesin family member 5B | Cytoplasm | other |
| MIMAT0000738 | miR-383-5p (miRNAs w/seed GAUCAGA) | 2.030 | TargetScan Human | Moderate (predicted) | KIRREL3 | irre like nephrin family adhesion molecule | Extracellular Space | other |
| MIMAT0000738 | miR-383-5p (miRNAs w/seed GAUCAGA) | 2.030 | TargetScan Human | High (predicted) | KLHDC10 | kelch domain containing 10 | Nucleus | other |
| MIMAT0000738 | miR-383-5p (miRNAs w/seed GAUCAGA) | 2.030 | TargetScan Human | Moderate (predicted) | KLLN | illin, p53 regulated DNA replication inhibito | Nucleus | other |
| MIMAT0000738 | miR-383-5p (miRNAs w/seed GAUCAGA) | 2.030 | TargetScan Human | Moderate (predicted) | KNTC1 | kinetochore associated 1 | Nucleus | other |
| MIMAT0000738 | miR-383-5p (miRNAs w/seed GAUCAGA) | 2.030 | TargetScan Human | Moderate (predicted) | KPNB1 | karyopherin subunit beta 1 | Nucleus | other |
| MIMAT0000738 | miR-383-5p (miRNAs w/seed GAUCAGA) | 2.030 | TargetScan Human | High (predicted) | KRTAP1-5 | keratin associated protein 1-5 | Cytoplasm | other |
| MIMAT0000738 | miR-383-5p (miRNAs w/seed GAUCAGA) | 2.030 | TargetScan Human | Moderate (predicted) | KRTAP9-3 | keratin associated protein 9-3 | Cytoplasm | other |
| MIMAT0000738 | miR-383-5p (miRNAs w/seed GAUCAGA) | 2.030 | TargetScan Human | Moderate (predicted) | KRTAP9-4 | keratin associated protein 9-4 | Cytoplasm | other |
| MIMAT0000738 | miR-383-5p (miRNAs w/seed GAUCAGA) | 2.030 | TargetScan Human | Moderate (predicted) | KRTAP9-6 | keratin associated protein 9-6 | Cytoplasm | other |
| MIMAT0000738 | miR-383-5p (miRNAs w/seed GAUCAGA) | 2.030 | TargetScan Human | Moderate (predicted) | KRTAP9-7 | keratin associated protein 9-7 | Cytoplasm | other |
| MIMAT0000738 | miR-383-5p (miRNAs w/seed GAUCAGA) | 2.030 | TargetScan Human | High (predicted) | KSR2 | kinase suppressor of ras 2 | Cytoplasm | kinase |
| MIMAT0000738 | miR-383-5p (miRNAs w/seed GAUCAGA) | 2.030 | TargetScan Human | Moderate (predicted) | KTI12 | KTI12 chromatin associated homolog | Other | other |
| MIMAT0000738 | miR-383-5p (miRNAs w/seed GAUCAGA) | 2.030 | TargetScan Human | Moderate (predicted) | LBP | lipopolysaccharide binding protein | Plasma Membrane | transporter |
| MIMAT0000738 | miR-383-5p (miRNAs w/seed GAUCAGA) | 2.030 | TargetScan Human | High (predicted) | LCE2C/LCE2D | late cornified envelope 2D | Other | other |
| MIMAT0000738 | miR-383-5p (miRNAs w/seed GAUCAGA) | 2.030 | TargetScan Human | Moderate (predicted) | LDHA | lactate dehydrogenase A | Cytoplasm | enzyme |
| MIMAT0000738 | miR-383-5p (miRNAs w/seed GAUCAGA) | 2.030 | TargetScan Human | Moderate (predicted) | LILRB4 | leukocyte immunoglobulin like receptor B4 | Plasma Membrane | other |
| MIMAT0000738 | miR-383-5p (miRNAs w/seed GAUCAGA) | 2.030 | TargetScan Human | High (predicted) | LINC01560 | ng intergenic non-protein coding RNA 156 | Other | other |
| MIMAT0000738 | miR-383-5p (miRNAs w/seed GAUCAGA) | 2.030 | TargetScan Human | Moderate (predicted) | LINC01565 | ng intergenic non-protein coding RNA 156 | Other | other |
| MIMAT0000738 | miR-383-5p (miRNAs w/seed GAUCAGA) | 2.030 | TargetScan Human | High (predicted) | LINC02313 | ng intergenic non-protein coding RNA 231 | Other | other |
| MIMAT0000738 | miR-383-5p (miRNAs w/seed GAUCAGA) | 2.030 | TargetScan Human | Moderate (predicted) | LIPN | lipase family member N | Extracellular Space | enzyme |
| MIMAT0000738 | miR-383-5p (miRNAs w/seed GAUCAGA) | 2.030 | TargetScan Human | Moderate (predicted) | LIPT2 | lipoyl(octanoyl) transferase 2 | Cytoplasm | enzyme |
| MIMAT0000738 | miR-383-5p (miRNAs w/seed GAUCAGA) | 2.030 | TargetScan Human | Moderate (predicted) | LIX1 | limb and CNS expressed 1 | Other | other |
| MIMAT0000738 | miR-383-5p (miRNAs w/seed GAUCAGA) | 2.030 | TargetScan Human | High (predicted) | LLCFC1 | LLLL and CFNLAS motif containing 1 | Other | other |

Sup. Table 3. List of 3699 targets of the seven dysregulated miRNAs obtained from three different target prediction databases,TargetScan TarBase and miRecords considering those predicted as moderate, high, or experimentally validated, respectively.

| | | | | | | | | |
|---|---|---|---|---|---|---|---|---|
| MIMAT0000738 | miR-383-5p (miRNAs w/seed GAUCAGA) | 2.030 | TargetScan Human | Moderate (predicted) | LMX1A | LIM homeobox transcription factor 1 alpha | Nucleus | transcription regulator |
| MIMAT0000738 | miR-383-5p (miRNAs w/seed GAUCAGA) | 2.030 | TargetScan Human | High (predicted) | LOC100132686 | uncharacterized LOC100132686 | Other | other |
| MIMAT0000738 | miR-383-5p (miRNAs w/seed GAUCAGA) | 2.030 | TargetScan Human | Moderate (predicted) | LOC100132813 | uncharacterized LOC100132813 | Other | other |
| MIMAT0000738 | miR-383-5p (miRNAs w/seed GAUCAGA) | 2.030 | TargetScan Human | High (predicted) | LOC440910 | uncharacterized LOC440910 | Other | other |
| MIMAT0000738 | miR-383-5p (miRNAs w/seed GAUCAGA) | 2.030 | TargetScan Human | Moderate (predicted) | LRGUK | ch repeats and guanylate kinase domain c | Cytoplasm | other |
| MIMAT0000738 | miR-383-5p (miRNAs w/seed GAUCAGA) | 2.030 | TargetScan Human | Moderate (predicted) | LRIG2 | e rich repeats and immunoglobulin like dor | Cytoplasm | other |
| MIMAT0000738 | miR-383-5p (miRNAs w/seed GAUCAGA) | 2.030 | TargetScan Human | Moderate (predicted) | LRRC74B | leucine rich repeat containing 74B | Other | other |
| MIMAT0000738 | miR-383-5p (miRNAs w/seed GAUCAGA) | 2.030 | TargetScan Human | Moderate (predicted) | LRRD1 | ine rich repeats and death domain contain | Other | other |
| MIMAT0000738 | miR-383-5p (miRNAs w/seed GAUCAGA) | 2.030 | TargetScan Human | Moderate (predicted) | LSM11 | LSM11, U7 small nuclear RNA associated | Nucleus | other |
| MIMAT0000738 | miR-383-5p (miRNAs w/seed GAUCAGA) | 2.030 | TargetScan Human | Moderate (predicted) | LSP1 | lymphocyte specific protein 1 | Cytoplasm | other |
| MIMAT0000738 | miR-383-5p (miRNAs w/seed GAUCAGA) | 2.030 | TargetScan Human | Moderate (predicted) | LTA | lymphotoxin alpha | Extracellular Space | cytokine |
| MIMAT0000738 | miR-383-5p (miRNAs w/seed GAUCAGA) | 2.030 | TargetScan Human | Moderate (predicted) | LUZP2 | leucine zipper protein 2 | Other | other |
| MIMAT0000738 | miR-383-5p (miRNAs w/seed GAUCAGA) | 2.030 | TargetScan Human | Moderate (predicted) | LYPD5 | LY6/PLAUR domain containing 5 | Other | other |
| MIMAT0000738 | miR-383-5p (miRNAs w/seed GAUCAGA) | 2.030 | TargetScan Human | Moderate (predicted) | MAFG | MAF bZIP transcription factor G | Nucleus | transcription regulator |
| MIMAT0000738 | miR-383-5p (miRNAs w/seed GAUCAGA) | 2.030 | TargetScan Human | High (predicted) | MAL2 | mal, T cell differentiation protein 2 | Plasma Membrane | transporter |
| MIMAT0000738 | miR-383-5p (miRNAs w/seed GAUCAGA) | 2.030 | TargetScan Human | High (predicted) | MALL | mal, T cell differentiation protein like | Plasma Membrane | other |
| MIMAT0000738 | miR-383-5p (miRNAs w/seed GAUCAGA) | 2.030 | TargetScan Human | Moderate (predicted) | MALRD1 | l and LDL receptor class A domain contair | Cytoplasm | other |
| MIMAT0000738 | miR-383-5p (miRNAs w/seed GAUCAGA) | 2.030 | TargetScan Human | Moderate (predicted) | MANBAL | mannosidase beta like | Other | other |
| MIMAT0000738 | miR-383-5p (miRNAs w/seed GAUCAGA) | 2.030 | TargetScan Human | Moderate (predicted) | MAPK10 | mitogen-activated protein kinase 10 | Cytoplasm | kinase |
| MIMAT0000738 | miR-383-5p (miRNAs w/seed GAUCAGA) | 2.030 | TargetScan Human | Moderate (predicted) | MBP | myelin basic protein | Extracellular Space | other |
| MIMAT0000738 | miR-383-5p (miRNAs w/seed GAUCAGA) | 2.030 | TargetScan Human | Moderate (predicted) | MBTD1 | mbt domain containing 1 | Other | other |
| MIMAT0000738 | miR-383-5p (miRNAs w/seed GAUCAGA) | 2.030 | TargetScan Human | High (predicted) | MDM4 | MDM4 regulator of p53 | Nucleus | transcription regulator |
| MIMAT0000738 | miR-383-5p (miRNAs w/seed GAUCAGA) | 2.030 | TargetScan Human | High (predicted) | MEAF6 | MYST/Esa1 associated factor 6 | Nucleus | other |
| MIMAT0000738 | miR-383-5p (miRNAs w/seed GAUCAGA) | 2.030 | TargetScan Human | Moderate (predicted) | MED19 | mediator complex subunit 19 | Nucleus | other |
| MIMAT0000738 | miR-383-5p (miRNAs w/seed GAUCAGA) | 2.030 | TargetScan Human | High (predicted) | MESD | mesoderm development LRP chaperone | Extracellular Space | other |
| MIMAT0000738 | miR-383-5p (miRNAs w/seed GAUCAGA) | 2.030 | TargetScan Human | High (predicted) | MEST | mesoderm specific transcript | Cytoplasm | peptidase |
| MIMAT0000738 | miR-383-5p (miRNAs w/seed GAUCAGA) | 2.030 | TargetScan Human | Moderate (predicted) | MFN1 | mitofusin 1 | Cytoplasm | enzyme |
| MIMAT0000738 | miR-383-5p (miRNAs w/seed GAUCAGA) | 2.030 | TargetScan Human | Moderate (predicted) | MFSD1 | jor facilitator superfamily domain containir | Cytoplasm | transporter |
| MIMAT0000738 | miR-383-5p (miRNAs w/seed GAUCAGA) | 2.030 | TargetScan Human | Moderate (predicted) | MFSD9 | jor facilitator superfamily domain containir | Other | transporter |
| MIMAT0000738 | miR-383-5p (miRNAs w/seed GAUCAGA) | 2.030 | TargetScan Human | Moderate (predicted) | MGA | MAX dimerization protein MGA | Nucleus | transcription regulator |
| MIMAT0000738 | miR-383-5p (miRNAs w/seed GAUCAGA) | 2.030 | TargetScan Human | Moderate (predicted) | MINAR1 | nbrane integral NOTCH2 associated recep | Plasma Membrane | other |
| MIMAT0000738 | miR-383-5p (miRNAs w/seed GAUCAGA) | 2.030 | TargetScan Human | High (predicted) | MLANA | melan-A | Plasma Membrane | other |
| MIMAT0000738 | miR-383-5p (miRNAs w/seed GAUCAGA) | 2.030 | TargetScan Human | Moderate (predicted) | MLPH | melanophilin | Cytoplasm | other |
| MIMAT0000738 | miR-383-5p (miRNAs w/seed GAUCAGA) | 2.030 | TargetScan Human | Moderate (predicted) | MMP24 | matrix metallopeptidase 24 | Plasma Membrane | peptidase |
| MIMAT0000738 | miR-383-5p (miRNAs w/seed GAUCAGA) | 2.030 | TargetScan Human | Moderate (predicted) | MMRN2 | multimerin 2 | Extracellular Space | other |
| MIMAT0000738 | miR-383-5p (miRNAs w/seed GAUCAGA) | 2.030 | TargetScan Human | Moderate (predicted) | MOB1B | MOB kinase activator 1B | Cytoplasm | other |
| MIMAT0000738 | miR-383-5p (miRNAs w/seed GAUCAGA) | 2.030 | TargetScan Human | High (predicted) | MOB3B | MOB kinase activator 3B | Other | other |
| MIMAT0000738 | miR-383-5p (miRNAs w/seed GAUCAGA) | 2.030 | TargetScan Human | Moderate (predicted) | MOB4 | MOB family member 4, phocein | Cytoplasm | other |
| MIMAT0000738 | miR-383-5p (miRNAs w/seed GAUCAGA) | 2.030 | TargetScan Human | Moderate (predicted) | MORN3 | MORN repeat containing 3 | Nucleus | other |
| MIMAT0000738 | miR-383-5p (miRNAs w/seed GAUCAGA) | 2.030 | TargetScan Human | Moderate (predicted) | MORN4 | MORN repeat containing 4 | Extracellular Space | other |
| MIMAT0000738 | miR-383-5p (miRNAs w/seed GAUCAGA) | 2.030 | TargetScan Human | Moderate (predicted) | MOSPD1 | motile sperm domain containing 1 | Other | other |
| MIMAT0000738 | miR-383-5p (miRNAs w/seed GAUCAGA) | 2.030 | TargetScan Human | High (predicted) | MREG | melanoregulin | Cytoplasm | other |
| MIMAT0000738 | miR-383-5p (miRNAs w/seed GAUCAGA) | 2.030 | TargetScan Human | Moderate (predicted) | MRM3 | mitochondrial rRNA methyltransferase 3 | Cytoplasm | enzyme |
| MIMAT0000738 | miR-383-5p (miRNAs w/seed GAUCAGA) | 2.030 | TargetScan Human | Moderate (predicted) | MROH1 | maestro heat like repeat family member 1 | Other | other |
| MIMAT0000738 | miR-383-5p (miRNAs w/seed GAUCAGA) | 2.030 | TargetScan Human | High (predicted) | MRPL27 | mitochondrial ribosomal protein L27 | Cytoplasm | other |
| MIMAT0000738 | miR-383-5p (miRNAs w/seed GAUCAGA) | 2.030 | TargetScan Human | Moderate (predicted) | MS4A10 | membrane spanning 4-domains A10 | Other | other |
| MIMAT0000738 | miR-383-5p (miRNAs w/seed GAUCAGA) | 2.030 | TargetScan Human | Moderate (predicted) | MS4A3 | membrane spanning 4-domains A3 | Plasma Membrane | other |
| MIMAT0000738 | miR-383-5p (miRNAs w/seed GAUCAGA) | 2.030 | TargetScan Human | Moderate (predicted) | MSL2 | MSL complex subunit 2 | Nucleus | other |
| MIMAT0000738 | miR-383-5p (miRNAs w/seed GAUCAGA) | 2.030 | TargetScan Human | Moderate (predicted) | MT1G | metallothionein 1G | Nucleus | other |
| MIMAT0000738 | miR-383-5p (miRNAs w/seed GAUCAGA) | 2.030 | TargetScan Human | High (predicted) | MT-ATP8 | ATP synthase F0 subunit 8 | Cytoplasm | enzyme |
| MIMAT0000738 | miR-383-5p (miRNAs w/seed GAUCAGA) | 2.030 | TargetScan Human | Moderate (predicted) | MTCP1 | mature T cell proliferation 1 | Cytoplasm | other |
| MIMAT0000738 | miR-383-5p (miRNAs w/seed GAUCAGA) | 2.030 | TargetScan Human | Moderate (predicted) | MTHFD2 | drogenase (NADP+ dependent) 2, methen | Cytoplasm | enzyme |
| MIMAT0000738 | miR-383-5p (miRNAs w/seed GAUCAGA) | 2.030 | TargetScan Human | Moderate (predicted) | MTMR6 | myotubularin related protein 6 | Cytoplasm | phosphatase |
| MIMAT0000738 | miR-383-5p (miRNAs w/seed GAUCAGA) | 2.030 | TargetScan Human | Moderate (predicted) | MTPN | myotrophin | Nucleus | transcription regulator |
| MIMAT0000738 | miR-383-5p (miRNAs w/seed GAUCAGA) | 2.030 | TargetScan Human | Moderate (predicted) | MTUS1 | microtubule associated scaffold protein 1 | Extracellular Space | other |
| MIMAT0000738 | miR-383-5p (miRNAs w/seed GAUCAGA) | 2.030 | TargetScan Human | Moderate (predicted) | MUC20 | mucin 20, cell surface associated | Plasma Membrane | other |
| MIMAT0000738 | miR-383-5p (miRNAs w/seed GAUCAGA) | 2.030 | TargetScan Human | Moderate (predicted) | MXRA7 | matrix remodeling associated 7 | Extracellular Space | other |
| MIMAT0000738 | miR-383-5p (miRNAs w/seed GAUCAGA) | 2.030 | TargetScan Human | Moderate (predicted) | MYT1L | myelin transcription factor 1 like | Nucleus | transcription regulator |
| MIMAT0000738 | miR-383-5p (miRNAs w/seed GAUCAGA) | 2.030 | TargetScan Human | Moderate (predicted) | NAV3 | neuron navigator 3 | Nucleus | other |
| MIMAT0000738 | miR-383-5p (miRNAs w/seed GAUCAGA) | 2.030 | TargetScan Human | Moderate (predicted) | NBEA | neurobeachin | Cytoplasm | other |
| MIMAT0000738 | miR-383-5p (miRNAs w/seed GAUCAGA) | 2.030 | TargetScan Human | High (predicted) | NCKAP1 | NCK associated protein 1 | Plasma Membrane | other |
| MIMAT0000738 | miR-383-5p (miRNAs w/seed GAUCAGA) | 2.030 | TargetScan Human | Moderate (predicted) | NCL | nucleolin | Nucleus | other |
| MIMAT0000738 | miR-383-5p (miRNAs w/seed GAUCAGA) | 2.030 | TargetScan Human | Moderate (predicted) | NDRG3 | NDRG family member 3 | Cytoplasm | other |
| MIMAT0000738 | miR-383-5p (miRNAs w/seed GAUCAGA) | 2.030 | TargetScan Human | Moderate (predicted) | NDUFA2 | ADH:ubiquinone oxidoreductase subunit A | Cytoplasm | enzyme |
| MIMAT0000738 | miR-383-5p (miRNAs w/seed GAUCAGA) | 2.030 | TargetScan Human | Moderate (predicted) | NFIA | nuclear factor I A | Nucleus | transcription regulator |
| MIMAT0000738 | miR-383-5p (miRNAs w/seed GAUCAGA) | 2.030 | TargetScan Human | Moderate (predicted) | NID2 | nidogen 2 | Extracellular Space | other |
| MIMAT0000738 | miR-383-5p (miRNAs w/seed GAUCAGA) | 2.030 | TargetScan Human | Moderate (predicted) | NNAT | neuronatin | Plasma Membrane | transporter |
| MIMAT0000738 | miR-383-5p (miRNAs w/seed GAUCAGA) | 2.030 | TargetScan Human | Moderate (predicted) | NPAT | lear protein, coactivator of histone transcri | Nucleus | transcription regulator |
| MIMAT0000738 | miR-383-5p (miRNAs w/seed GAUCAGA) | 2.030 | TargetScan Human | Moderate (predicted) | NR2F6 | clear receptor subfamily 2 group F membe | Nucleus | ligand-dependent nuclear receptor |
| MIMAT0000738 | miR-383-5p (miRNAs w/seed GAUCAGA) | 2.030 | TargetScan Human | Moderate (predicted) | NRG1 | neuregulin 1 | Plasma Membrane | growth factor |
| MIMAT0000738 | miR-383-5p (miRNAs w/seed GAUCAGA) | 2.030 | TargetScan Human | Moderate (predicted) | NRGN | neurogranin | Cytoplasm | other |
| MIMAT0000738 | miR-383-5p (miRNAs w/seed GAUCAGA) | 2.030 | TargetScan Human | Moderate (predicted) | NT5C1A | 5'-nucleotidase, cytosolic IA | Cytoplasm | phosphatase |
| MIMAT0000738 | miR-383-5p (miRNAs w/seed GAUCAGA) | 2.030 | TargetScan Human | Moderate (predicted) | NUDCD2 | NudC domain containing 2 | Cytoplasm | other |
| MIMAT0000738 | miR-383-5p (miRNAs w/seed GAUCAGA) | 2.030 | TargetScan Human | Moderate (predicted) | NUDT18 | nudix hydrolase 18 | Cytoplasm | enzyme |
| MIMAT0000738 | miR-383-5p (miRNAs w/seed GAUCAGA) | 2.030 | TargetScan Human | High (predicted) | NXPH1 | neurexophilin 1 | Extracellular Space | other |
| MIMAT0000738 | miR-383-5p (miRNAs w/seed GAUCAGA) | 2.030 | TargetScan Human | Moderate (predicted) | OIP5 | Opa interacting protein 5 | Nucleus | other |
| MIMAT0000738 | miR-383-5p (miRNAs w/seed GAUCAGA) | 2.030 | TargetScan Human | Moderate (predicted) | OPA3 | chondrial membrane lipid metabolism regu | Cytoplasm | other |
| MIMAT0000738 | miR-383-5p (miRNAs w/seed GAUCAGA) | 2.030 | TargetScan Human | High (predicted) | OPN3 | opsin 3 | Plasma Membrane | G-protein coupled receptor |
| MIMAT0000738 | miR-383-5p (miRNAs w/seed GAUCAGA) | 2.030 | TargetScan Human | Moderate (predicted) | OSBPL2 | oxysterol binding protein like 2 | Cytoplasm | transporter |
| MIMAT0000738 | miR-383-5p (miRNAs w/seed GAUCAGA) | 2.030 | TargetScan Human | High (predicted) | OTX2 | orthodenticle homeobox 2 | Nucleus | transcription regulator |
| MIMAT0000738 | miR-383-5p (miRNAs w/seed GAUCAGA) | 2.030 | TargetScan Human | Moderate (predicted) | PABPC3 | poly(A) binding protein cytoplasmic 3 | Cytoplasm | other |
| MIMAT0000738 | miR-383-5p (miRNAs w/seed GAUCAGA) | 2.030 | TargetScan Human | High (predicted) | PADI1 | peptidyl arginine deiminase 1 | Cytoplasm | enzyme |
| MIMAT0000738 | miR-383-5p (miRNAs w/seed GAUCAGA) | 2.030 | TargetScan Human | Moderate (predicted) | PAN3 | poly(A) specific ribonuclease subunit PAN3 | Cytoplasm | enzyme |
| MIMAT0000738 | miR-383-5p (miRNAs w/seed GAUCAGA) | 2.030 | TargetScan Human | Moderate (predicted) | PANK1 | pantothenate kinase 1 | Cytoplasm | kinase |
| MIMAT0000738 | miR-383-5p (miRNAs w/seed GAUCAGA) | 2.030 | TargetScan Human | Moderate (predicted) | PARG | poly(ADP-ribose) glycohydrolase | Cytoplasm | enzyme |
| MIMAT0000738 | miR-383-5p (miRNAs w/seed GAUCAGA) | 2.030 | TargetScan Human | Moderate (predicted) | PARP2 | poly(ADP-ribose) polymerase 2 | Nucleus | enzyme |
| MIMAT0000738 | miR-383-5p (miRNAs w/seed GAUCAGA) | 2.030 | TargetScan Human | High (predicted) | PATE4 | prostate and testis expressed 4 | Other | other |
| MIMAT0000738 | miR-383-5p (miRNAs w/seed GAUCAGA) | 2.030 | TargetScan Human | Moderate (predicted) | PATZ1 | OZ/BTB and AT hook containing zinc finge | Nucleus | transcription regulator |
| MIMAT0000738 | miR-383-5p (miRNAs w/seed GAUCAGA) | 2.030 | TargetScan Human | Moderate (predicted) | PCGF5 | polycomb group ring finger 5 | Cytoplasm | other |
| MIMAT0000738 | miR-383-5p (miRNAs w/seed GAUCAGA) | 2.030 | TargetScan Human | Moderate (predicted) | PCLAF | PCNA clamp associated factor | Nucleus | other |
| MIMAT0000738 | miR-383-5p (miRNAs w/seed GAUCAGA) | 2.030 | TargetScan Human | Moderate (predicted) | PCM1 | pericentriolar material 1 | Cytoplasm | other |
| MIMAT0000738 | miR-383-5p (miRNAs w/seed GAUCAGA) | 2.030 | TargetScan Human | Moderate (predicted) | PCSK2 | proprotein convertase subtilisin/kexin type | Extracellular Space | peptidase |
| MIMAT0000738 | miR-383-5p (miRNAs w/seed GAUCAGA) | 2.030 | TargetScan Human | Moderate (predicted) | PEG10 | paternally expressed 10 | Nucleus | other |
| MIMAT0000738 | miR-383-5p (miRNAs w/seed GAUCAGA) | 2.030 | TargetScan Human | High (predicted) | PERP | p53 apoptosis effector related to PMP22 | Plasma Membrane | other |
| MIMAT0000738 | miR-383-5p (miRNAs w/seed GAUCAGA) | 2.030 | TargetScan Human | High (predicted) | PET117 | PET117 cytochrome c oxidase chaperone | Cytoplasm | other |
| MIMAT0000738 | miR-383-5p (miRNAs w/seed GAUCAGA) | 2.030 | TargetScan Human | Moderate (predicted) | PEX11A | peroxisomal biogenesis factor 11 alpha | Cytoplasm | other |
| MIMAT0000738 | miR-383-5p (miRNAs w/seed GAUCAGA) | 2.030 | TargetScan Human | Moderate (predicted) | PEX13 | peroxisomal biogenesis factor 13 | Cytoplasm | transporter |
| MIMAT0000738 | miR-383-5p (miRNAs w/seed GAUCAGA) | 2.030 | TargetScan Human | Moderate (predicted) | PEX5L | peroxisomal biogenesis factor 5 like | Cytoplasm | ion channel |
| MIMAT0000738 | miR-383-5p (miRNAs w/seed GAUCAGA) | 2.030 | TargetScan Human | Moderate (predicted) | PFKM | phosphofructokinase, muscle | Cytoplasm | kinase |
| MIMAT0000738 | miR-383-5p (miRNAs w/seed GAUCAGA) | 2.030 | TargetScan Human | High (predicted) | PFN2 | profilin 2 | Cytoplasm | enzyme |
| MIMAT0000738 | miR-383-5p (miRNAs w/seed GAUCAGA) | 2.030 | TargetScan Human | Moderate (predicted) | PGD | phosphogluconate dehydrogenase | Cytoplasm | enzyme |
| MIMAT0000738 | miR-383-5p (miRNAs w/seed GAUCAGA) | 2.030 | TargetScan Human | Moderate (predicted) | PGM5 | phosphoglucomutase 5 | Cytoplasm | enzyme |
| MIMAT0000738 | miR-383-5p (miRNAs w/seed GAUCAGA) | 2.030 | TargetScan Human | Moderate (predicted) | PGPEP1 | pyroglutamyl-peptidase I | Cytoplasm | peptidase |
| MIMAT0000738 | miR-383-5p (miRNAs w/seed GAUCAGA) | 2.030 | TargetScan Human | Moderate (predicted) | PHACTR3 | phosphatase and actin regulator 3 | Nucleus | other |
| MIMAT0000738 | miR-383-5p (miRNAs w/seed GAUCAGA) | 2.030 | TargetScan Human | Moderate (predicted) | PIGU | hatidylinositol glycan anchor biosynthesis | Cytoplasm | enzyme |
| MIMAT0000738 | miR-383-5p (miRNAs w/seed GAUCAGA) | 2.030 | TargetScan Human | Moderate (predicted) | PIN4 | dylprolyl cis/trans isomerase, NIMA-interac | Nucleus | enzyme |
| MIMAT0000738 | miR-383-5p (miRNAs w/seed GAUCAGA) | 2.030 | TargetScan Human | Moderate (predicted) | PIP4K2C | atidylinositol-5-phosphate 4-kinase type 2 | Cytoplasm | kinase |
| MIMAT0000738 | miR-383-5p (miRNAs w/seed GAUCAGA) | 2.030 | TargetScan Human | Moderate (predicted) | PIP5KL1 | osphatidylinositol-4-phosphate 5-kinase lik | Plasma Membrane | kinase |
| MIMAT0000738 | miR-383-5p (miRNAs w/seed GAUCAGA) | 2.030 | TargetScan Human | Moderate (predicted) | PLA2G2D | phospholipase A2 group IID | Extracellular Space | enzyme |
| MIMAT0000738 | miR-383-5p (miRNAs w/seed GAUCAGA) | 2.030 | TargetScan Human | Moderate (predicted) | PLA2G3 | phospholipase A2 group III | Extracellular Space | enzyme |
| MIMAT0000738 | miR-383-5p (miRNAs w/seed GAUCAGA) | 2.030 | TargetScan Human | Moderate (predicted) | PLA2G4A | phospholipase A2 group IVA | Cytoplasm | enzyme |
| MIMAT0000738 | miR-383-5p (miRNAs w/seed GAUCAGA) | 2.030 | TargetScan Human | Moderate (predicted) | PLA2G4D | phospholipase A2 group IVD | Cytoplasm | enzyme |
| MIMAT0000738 | miR-383-5p (miRNAs w/seed GAUCAGA) | 2.030 | TargetScan Human | High (predicted) | PLAC1 | placenta enriched 1 | Plasma Membrane | other |
| MIMAT0000738 | miR-383-5p (miRNAs w/seed GAUCAGA) | 2.030 | TargetScan Human | Moderate (predicted) | PLD1 | phospholipase D1 | Cytoplasm | enzyme |
| MIMAT0000738 | miR-383-5p (miRNAs w/seed GAUCAGA) | 2.030 | TargetScan Human | Moderate (predicted) | PLEKHA1 | pleckstrin homology domain containing A1 | Cytoplasm | other |
| MIMAT0000738 | miR-383-5p (miRNAs w/seed GAUCAGA) | 2.030 | TargetScan Human | Moderate (predicted) | PLEKHB2 | pleckstrin homology domain containing B2 | Other | other |
| MIMAT0000738 | miR-383-5p (miRNAs w/seed GAUCAGA) | 2.030 | TargetScan Human | Moderate (predicted) | PLPPR1 | phospholipid phosphatase related 1 | Nucleus | other |
| MIMAT0000738 | miR-383-5p (miRNAs w/seed GAUCAGA) | 2.030 | TargetScan Human | Moderate (predicted) | POC1B | POC1 centriolar protein B | Cytoplasm | other |
| MIMAT0000738 | miR-383-5p (miRNAs w/seed GAUCAGA) | 2.030 | TargetScan Human | Moderate (predicted) | POGK | ransposable element derived with KRAB c | Nucleus | other |
| MIMAT0000738 | miR-383-5p (miRNAs w/seed GAUCAGA) | 2.030 | TargetScan Human | High (predicted) | POLE3 | NA polymerase epsilon 3, accessory subu | Nucleus | enzyme |
| MIMAT0000738 | miR-383-5p (miRNAs w/seed GAUCAGA) | 2.030 | TargetScan Human | Moderate (predicted) | POT1 | protection of telomeres 1 | Nucleus | other |
| MIMAT0000738 | miR-383-5p (miRNAs w/seed GAUCAGA) | 2.030 | TargetScan Human | Moderate (predicted) | PPEF1 | protein phosphatase with EF-hand domain | Extracellular Space | phosphatase |
| MIMAT0000738 | miR-383-5p (miRNAs w/seed GAUCAGA) | 2.030 | TargetScan Human | Moderate (predicted) | PPFIA2 | PTPRF interacting protein alpha 2 | Plasma Membrane | phosphatase |
| MIMAT0000738 | miR-383-5p (miRNAs w/seed GAUCAGA) | 2.030 | TargetScan Human | High (predicted) | PPIL6 | peptidylprolyl isomerase like 6 | Other | enzyme |
| MIMAT0000738 | miR-383-5p (miRNAs w/seed GAUCAGA) | 2.030 | TargetScan Human | Moderate (predicted) | PPP1CB | protein phosphatase 1 catalytic subunit bet | Cytoplasm | phosphatase |
| MIMAT0000738 | miR-383-5p (miRNAs w/seed GAUCAGA) | 2.030 | TargetScan Human | Moderate (predicted) | PPP1R10 | protein phosphatase 1 regulatory subunit 1 | Nucleus | other |
| MIMAT0000738 | miR-383-5p (miRNAs w/seed GAUCAGA) | 2.030 | TargetScan Human | Moderate (predicted) | PPP1R3C | protein phosphatase 1 regulatory subunit 3 | Cytoplasm | phosphatase |
| MIMAT0000738 | miR-383-5p (miRNAs w/seed GAUCAGA) | 2.030 | TargetScan Human | High (predicted) | PPP1R3D | protein phosphatase 1 regulatory subunit 3 | Cytoplasm | phosphatase |
| MIMAT0000738 | miR-383-5p (miRNAs w/seed GAUCAGA) | 2.030 | TargetScan Human | Moderate (predicted) | PRAMEF11 | PRAME family member 11 | Extracellular Space | other |
| MIMAT0000738 | miR-383-5p (miRNAs w/seed GAUCAGA) | 2.030 | TargetScan Human | Moderate (predicted) | AMEF4 (includes oth | PRAME family member 4 | Other | other |
| MIMAT0000738 | miR-383-5p (miRNAs w/seed GAUCAGA) | 2.030 | TargetScan Human | High (predicted) | PRDX3 | peroxiredoxin 3 | Cytoplasm | enzyme |
| MIMAT0000738 | miR-383-5p (miRNAs w/seed GAUCAGA) | 2.030 | TargetScan Human | Moderate (predicted) | PREX2 | sitol-3,4,5-trisphosphate dependent Rac e | Cytoplasm | other |
| MIMAT0000738 | miR-383-5p (miRNAs w/seed GAUCAGA) | 2.030 | TargetScan Human | Moderate (predicted) | PRKAG1 | nase AMP-activated non-catalytic subunit | Nucleus | kinase |
| MIMAT0000738 | miR-383-5p (miRNAs w/seed GAUCAGA) | 2.030 | TargetScan Human | Moderate (predicted) | PRR19 | proline rich 19 | Other | other |
| MIMAT0000738 | miR-383-5p (miRNAs w/seed GAUCAGA) | 2.030 | TargetScan Human | Moderate (predicted) | PRR3 | proline rich 3 | Other | other |
| MIMAT0000738 | miR-383-5p (miRNAs w/seed GAUCAGA) | 2.030 | TargetScan Human | Moderate (predicted) | PRRT1 | proline rich transmembrane protein 1 | Other | other |
| MIMAT0000738 | miR-383-5p (miRNAs w/seed GAUCAGA) | 2.030 | TargetScan Human | Moderate (predicted) | PRRX1 | paired related homeobox 1 | Nucleus | transcription regulator |
| MIMAT0000738 | miR-383-5p (miRNAs w/seed GAUCAGA) | 2.030 | TargetScan Human | Moderate (predicted) | PRTFDC1 | osphoribosyl transferase domain containin | Cytoplasm | enzyme |
| MIMAT0000738 | miR-383-5p (miRNAs w/seed GAUCAGA) | 2.030 | TargetScan Human | Moderate (predicted) | PSME4 | proteasome activator subunit 4 | Cytoplasm | other |
| MIMAT0000738 | miR-383-5p (miRNAs w/seed GAUCAGA) | 2.030 | TargetScan Human | High (predicted) | PSMG2 | proteasome assembly chaperone 2 | Nucleus | other |
| MIMAT0000738 | miR-383-5p (miRNAs w/seed GAUCAGA) | 2.030 | TargetScan Human | Moderate (predicted) | PSTK | phosphoseryl-tRNA kinase | Cytoplasm | kinase |
| MIMAT0000738 | miR-383-5p (miRNAs w/seed GAUCAGA) | 2.030 | TargetScan Human | High (predicted) | PYCARD | PYD and CARD domain containing | Cytoplasm | transcription regulator |
| MIMAT0000738 | miR-383-5p (miRNAs w/seed GAUCAGA) | 2.030 | TargetScan Human | Moderate (predicted) | PYCR1 | pyrroline-5-carboxylate reductase 1 | Cytoplasm | enzyme |
| MIMAT0000738 | miR-383-5p (miRNAs w/seed GAUCAGA) | 2.030 | TargetScan Human | Moderate (predicted) | RAB27B | RAB27B, member RAS oncogene family | Cytoplasm | enzyme |
| MIMAT0000738 | miR-383-5p (miRNAs w/seed GAUCAGA) | 2.030 | TargetScan Human | Moderate (predicted) | RAB3D | RAB3D, member RAS oncogene family | Cytoplasm | enzyme |
| MIMAT0000738 | miR-383-5p (miRNAs w/seed GAUCAGA) | 2.030 | TargetScan Human | Moderate (predicted) | RAB7A | RAB7A, member RAS oncogene family | Cytoplasm | enzyme |

Sup. Table 3. List of 3699 targets of the seven dysregulated miRNAs obtained from three different target prediction databases,TargetScan TarBase and miRecords considering those predicted as moderate, high, or experimentally validated, respectively.

| | | | | | | | | |
|---|---|---|---|---|---|---|---|---|
| MIMAT0000738 | miR-383-5p (miRNAs w/seed GAUCAGA) | 2.030 | TargetScan Human | Moderate (predicted) | RAB8A | RAB8A, member RAS oncogene family | Plasma Membrane | enzyme |
| MIMAT0000738 | miR-383-5p (miRNAs w/seed GAUCAGA) | 2.030 | TargetScan Human | Moderate (predicted) | RABGAP1 | RAB GTPase activating protein 1 | Cytoplasm | other |
| MIMAT0000738 | miR-383-5p (miRNAs w/seed GAUCAGA) | 2.030 | TargetScan Human | Moderate (predicted) | RAD51AP1 | RAD51 associated protein 1 | Nucleus | other |
| MIMAT0000738 | miR-383-5p (miRNAs w/seed GAUCAGA) | 2.030 | TargetScan Human | High (predicted) | RAD54B | RAD54 homolog B | Nucleus | enzyme |
| MIMAT0000738 | miR-383-5p (miRNAs w/seed GAUCAGA) | 2.030 | TargetScan Human | Moderate (predicted) | RAG2 | recombination activating 2 | Nucleus | enzyme |
| MIMAT0000738 | miR-383-5p (miRNAs w/seed GAUCAGA) | 2.030 | TargetScan Human | Moderate (predicted) | RALB | RAS like proto-oncogene B | Cytoplasm | enzyme |
| MIMAT0000738 | miR-383-5p (miRNAs w/seed GAUCAGA) | 2.030 | TargetScan Human | Moderate (predicted) | RALGPS1 | GEF with PH domain and SH3 binding mo | Cytoplasm | other |
| MIMAT0000738 | miR-383-5p (miRNAs w/seed GAUCAGA) | 2.030 | TargetScan Human | High (predicted) | RAP2A | RAP2A, member of RAS oncogene family | Plasma Membrane | enzyme |
| MIMAT0000738 | miR-383-5p (miRNAs w/seed GAUCAGA) | 2.030 | TargetScan Human | Moderate (predicted) | RAPGEF2 | Rap guanine nucleotide exchange factor 2 | Cytoplasm | other |
| MIMAT0000738 | miR-383-5p (miRNAs w/seed GAUCAGA) | 2.030 | TargetScan Human | Moderate (predicted) | RASAL2 | RAS protein activator like 2 | Extracellular Space | other |
| MIMAT0000738 | miR-383-5p (miRNAs w/seed GAUCAGA) | 2.030 | TargetScan Human | Moderate (predicted) | RASEF | RAS and EF-hand domain containing | Cytoplasm | other |
| MIMAT0000738 | miR-383-5p (miRNAs w/seed GAUCAGA) | 2.030 | TargetScan Human | Moderate (predicted) | RASL10B | RAS like family 10 member B | Other | enzyme |
| MIMAT0000738 | miR-383-5p (miRNAs w/seed GAUCAGA) | 2.030 | TargetScan Human | High (predicted) | RASSF8 | Ras association domain family member 8 | Extracellular Space | other |
| MIMAT0000738 | miR-383-5p (miRNAs w/seed GAUCAGA) | 2.030 | TargetScan Human | High (predicted) | RBBP9 | RB binding protein 9, serine hydrolase | Nucleus | other |
| MIMAT0000738 | miR-383-5p (miRNAs w/seed GAUCAGA) | 2.030 | TargetScan Human | Moderate (predicted) | RBM15B | RNA binding motif protein 15B | Nucleus | other |
| MIMAT0000738 | miR-383-5p (miRNAs w/seed GAUCAGA) | 2.030 | TargetScan Human | Moderate (predicted) | RBM26 | RNA binding motif protein 26 | Other | other |
| MIMAT0000738 | miR-383-5p (miRNAs w/seed GAUCAGA) | 2.030 | TargetScan Human | Moderate (predicted) | RBM3 | RNA binding motif protein 3 | Cytoplasm | other |
| MIMAT0000738 | miR-383-5p (miRNAs w/seed GAUCAGA) | 2.030 | TargetScan Human | Moderate (predicted) | RBM7 | RNA binding motif protein 7 | Nucleus | other |
| MIMAT0000738 | miR-383-5p (miRNAs w/seed GAUCAGA) | 2.030 | TargetScan Human | High (predicted) | RBMS1 | binding motif single stranded interacting pr | Nucleus | other |
| MIMAT0000738 | miR-383-5p (miRNAs w/seed GAUCAGA) | 2.030 | TargetScan Human | Moderate (predicted) | RCAN2 | regulator of calcineurin 2 | Other | other |
| MIMAT0000738 | miR-383-5p (miRNAs w/seed GAUCAGA) | 2.030 | TargetScan Human | High (predicted) | RCBTB2 | RCC1 and BTB domain containing protein | Cytoplasm | other |
| MIMAT0000738 | miR-383-5p (miRNAs w/seed GAUCAGA) | 2.030 | TargetScan Human | Moderate (predicted) | RCSD1 | RCSD domain containing 1 | Other | other |
| MIMAT0000738 | miR-383-5p (miRNAs w/seed GAUCAGA) | 2.030 | TargetScan Human | Moderate (predicted) | RDH12 | retinol dehydrogenase 12 | Cytoplasm | enzyme |
| MIMAT0000738 | miR-383-5p (miRNAs w/seed GAUCAGA) | 2.030 | TargetScan Human | Moderate (predicted) | REEP5 | receptor accessory protein 5 | Extracellular Space | transporter |
| MIMAT0000738 | miR-383-5p (miRNAs w/seed GAUCAGA) | 2.030 | TargetScan Human | Moderate (predicted) | RETREG1 | reticulophagy regulator 1 | Cytoplasm | other |
| MIMAT0000738 | miR-383-5p (miRNAs w/seed GAUCAGA) | 2.030 | TargetScan Human | Moderate (predicted) | RGS4 | regulator of G protein signaling 4 | Cytoplasm | enzyme |
| MIMAT0000738 | miR-383-5p (miRNAs w/seed GAUCAGA) | 2.030 | TargetScan Human | High (predicted) | RHAG | Rh associated glycoprotein | Plasma Membrane | peptidase |
| MIMAT0000738 | miR-383-5p (miRNAs w/seed GAUCAGA) | 2.030 | TargetScan Human | Moderate (predicted) | RHOBTB1 | Rho related BTB domain containing 1 | Cytoplasm | enzyme |
| MIMAT0000738 | miR-383-5p (miRNAs w/seed GAUCAGA) | 2.030 | TargetScan Human | Moderate (predicted) | RIPPLY3 | ripply transcriptional repressor 3 | Nucleus | other |
| MIMAT0000738 | miR-383-5p (miRNAs w/seed GAUCAGA) | 2.030 | TargetScan Human | High (predicted) | RMND5A | uired for meiotic nuclear division 5 homolo | Nucleus | enzyme |
| MIMAT0000738 | miR-383-5p (miRNAs w/seed GAUCAGA) | 2.030 | TargetScan Human | Moderate (predicted) | RNASE13 | ibonuclease A family member 13 (inactive | Other | other |
| MIMAT0000738 | miR-383-5p (miRNAs w/seed GAUCAGA) | 2.030 | TargetScan Human | High (predicted) | RNF141 | ring finger protein 141 | Other | enzyme |
| MIMAT0000738 | miR-383-5p (miRNAs w/seed GAUCAGA) | 2.030 | TargetScan Human | Moderate (predicted) | RNPEP | arginyl aminopeptidase | Cytoplasm | peptidase |
| MIMAT0000738 | miR-383-5p (miRNAs w/seed GAUCAGA) | 2.030 | TargetScan Human | High (predicted) | ROBO3 | roundabout guidance receptor 3 | Plasma Membrane | transmembrane receptor |
| MIMAT0000738 | miR-383-5p (miRNAs w/seed GAUCAGA) | 2.030 | TargetScan Human | Moderate (predicted) | ROGDI | rogdi atypical leucine zipper | Nucleus | other |
| MIMAT0000738 | miR-383-5p (miRNAs w/seed GAUCAGA) | 2.030 | TargetScan Human | Moderate (predicted) | RORB | RAR related orphan receptor B | Nucleus | ligand-dependent nuclear receptor |
| MIMAT0000738 | miR-383-5p (miRNAs w/seed GAUCAGA) | 2.030 | TargetScan Human | Moderate (predicted) | RORC | RAR related orphan receptor C | Nucleus | ligand-dependent nuclear receptor |
| MIMAT0000738 | miR-383-5p (miRNAs w/seed GAUCAGA) | 2.030 | TargetScan Human | High (predicted) | RP11 1167A192 | | Other | other |
| MIMAT0000738 | miR-383-5p (miRNAs w/seed GAUCAGA) | 2.030 | TargetScan Human | Moderate (predicted) | RP11 126H74 | | Other | other |
| MIMAT0000738 | miR-383-5p (miRNAs w/seed GAUCAGA) | 2.030 | TargetScan Human | High (predicted) | RP11 770J14 | | Other | other |
| MIMAT0000738 | miR-383-5p (miRNAs w/seed GAUCAGA) | 2.030 | TargetScan Human | High (predicted) | RP11 80A151 | | Other | other |
| MIMAT0000738 | miR-383-5p (miRNAs w/seed GAUCAGA) | 2.030 | TargetScan Human | Moderate (predicted) | RP2 | RP2 activator of ARL3 GTPase | Cytoplasm | enzyme |
| MIMAT0000738 | miR-383-5p (miRNAs w/seed GAUCAGA) | 2.030 | TargetScan Human | Moderate (predicted) | RP5 856G12 | | Other | other |
| MIMAT0000738 | miR-383-5p (miRNAs w/seed GAUCAGA) | 2.030 | TargetScan Human | High (predicted) | RPL36A-HNRNPH2 | RPL36A-HNRNPH2 readthrough | Other | other |
| MIMAT0000738 | miR-383-5p (miRNAs w/seed GAUCAGA) | 2.030 | TargetScan Human | Moderate (predicted) | RPL41 | ribosomal protein L41 | Cytoplasm | other |
| MIMAT0000738 | miR-383-5p (miRNAs w/seed GAUCAGA) | 2.030 | TargetScan Human | High (predicted) | RPRD1B | ation of nuclear pre-mRNA domain contain | Nucleus | other |
| MIMAT0000738 | miR-383-5p (miRNAs w/seed GAUCAGA) | 2.030 | TargetScan Human | Moderate (predicted) | RPS6 | ribosomal protein S6 | Cytoplasm | other |
| MIMAT0000738 | miR-383-5p (miRNAs w/seed GAUCAGA) | 2.030 | TargetScan Human | Moderate (predicted) | RPS6KC1 | ribosomal protein S6 kinase C1 | Cytoplasm | kinase |
| MIMAT0000738 | miR-383-5p (miRNAs w/seed GAUCAGA) | 2.030 | TargetScan Human | High (predicted) | RSBN1 | round spermatid basic protein 1 | Nucleus | other |
| MIMAT0000738 | miR-383-5p (miRNAs w/seed GAUCAGA) | 2.030 | TargetScan Human | Moderate (predicted) | RSPH3 | radial spoke head 3 | Extracellular Space | other |
| MIMAT0000738 | miR-383-5p (miRNAs w/seed GAUCAGA) | 2.030 | TargetScan Human | Moderate (predicted) | RXFP4 | relaxin family peptide/INSL5 receptor 4 | Plasma Membrane | G-protein coupled receptor |
| MIMAT0000738 | miR-383-5p (miRNAs w/seed GAUCAGA) | 2.030 | TargetScan Human | Moderate (predicted) | S100A13 | S100 calcium binding protein A13 | Cytoplasm | other |
| MIMAT0000738 | miR-383-5p (miRNAs w/seed GAUCAGA) | 2.030 | TargetScan Human | High (predicted) | SAMD12 | sterile alpha motif domain containing 12 | Other | other |
| MIMAT0000738 | miR-383-5p (miRNAs w/seed GAUCAGA) | 2.030 | TargetScan Human | Moderate (predicted) | SARAF | perated calcium entry associated regulator | Cytoplasm | other |
| MIMAT0000738 | miR-383-5p (miRNAs w/seed GAUCAGA) | 2.030 | TargetScan Human | Moderate (predicted) | SASS6 | SAS-6 centriolar assembly protein | Cytoplasm | other |
| MIMAT0000738 | miR-383-5p (miRNAs w/seed GAUCAGA) | 2.030 | TargetScan Human | High (predicted) | SCD | stearoyl-CoA desaturase | Cytoplasm | enzyme |
| MIMAT0000738 | miR-383-5p (miRNAs w/seed GAUCAGA) | 2.030 | TargetScan Human | High (predicted) | SCN3B | odium voltage-gated channel beta subunit | Plasma Membrane | ion channel |
| MIMAT0000738 | miR-383-5p (miRNAs w/seed GAUCAGA) | 2.030 | TargetScan Human | Moderate (predicted) | SCOC | short coiled-coil protein | Cytoplasm | other |
| MIMAT0000738 | miR-383-5p (miRNAs w/seed GAUCAGA) | 2.030 | TargetScan Human | Moderate (predicted) | SDE2 | SDE2 telomere maintenance homolog | Nucleus | other |
| MIMAT0000738 | miR-383-5p (miRNAs w/seed GAUCAGA) | 2.030 | TargetScan Human | Moderate (predicted) | SEC61B | SEC61 translocon subunit beta | Cytoplasm | transporter |
| MIMAT0000738 | miR-383-5p (miRNAs w/seed GAUCAGA) | 2.030 | TargetScan Human | Moderate (predicted) | SEC63 | C63 homolog, protein translocation regula | Cytoplasm | transporter |
| MIMAT0000738 | miR-383-5p (miRNAs w/seed GAUCAGA) | 2.030 | TargetScan Human | Moderate (predicted) | SEL1L | 1L adaptor subunit of ERAD E3 ubiquitin l | Cytoplasm | other |
| MIMAT0000738 | miR-383-5p (miRNAs w/seed GAUCAGA) | 2.030 | TargetScan Human | Moderate (predicted) | SEPSECS | sphoserine) tRNA:Sec (selenocysteine) tR | Cytoplasm | enzyme |
| MIMAT0000738 | miR-383-5p (miRNAs w/seed GAUCAGA) | 2.030 | TargetScan Human | High (predicted) | SERINC5 | serine incorporator 5 | Plasma Membrane | transporter |
| MIMAT0000738 | miR-383-5p (miRNAs w/seed GAUCAGA) | 2.030 | TargetScan Human | Moderate (predicted) | SERP2 | ciated endoplasmic reticulum protein famil | Other | other |
| MIMAT0000738 | miR-383-5p (miRNAs w/seed GAUCAGA) | 2.030 | TargetScan Human | Moderate (predicted) | SFTPB | surfactant protein B | Extracellular Space | other |
| MIMAT0000738 | miR-383-5p (miRNAs w/seed GAUCAGA) | 2.030 | TargetScan Human | Moderate (predicted) | SGMS2 | sphingomyelin synthase 2 | Plasma Membrane | enzyme |
| MIMAT0000738 | miR-383-5p (miRNAs w/seed GAUCAGA) | 2.030 | TargetScan Human | Moderate (predicted) | SH2D4A | SH2 domain containing 4A | Cytoplasm | other |
| MIMAT0000738 | miR-383-5p (miRNAs w/seed GAUCAGA) | 2.030 | TargetScan Human | Moderate (predicted) | SHISA3 | shisa family member 3 | Other | other |
| MIMAT0000738 | miR-383-5p (miRNAs w/seed GAUCAGA) | 2.030 | TargetScan Human | Moderate (predicted) | SHMT2 | serine hydroxymethyltransferase 2 | Cytoplasm | enzyme |
| MIMAT0000738 | miR-383-5p (miRNAs w/seed GAUCAGA) | 2.030 | TargetScan Human | Moderate (predicted) | SHTN1 | shootin 1 | Plasma Membrane | other |
| MIMAT0000738 | miR-383-5p (miRNAs w/seed GAUCAGA) | 2.030 | TargetScan Human | Moderate (predicted) | SIM1 | SIM bHLH transcription factor 1 | Nucleus | transcription regulator |
| MIMAT0000738 | miR-383-5p (miRNAs w/seed GAUCAGA) | 2.030 | TargetScan Human | Moderate (predicted) | SKAP1 | src kinase associated phosphoprotein 1 | Cytoplasm | kinase |
| MIMAT0000738 | miR-383-5p (miRNAs w/seed GAUCAGA) | 2.030 | TargetScan Human | Moderate (predicted) | SLAMF1 | g lymphocytic activation molecule family m | Plasma Membrane | transmembrane receptor |
| MIMAT0000738 | miR-383-5p (miRNAs w/seed GAUCAGA) | 2.030 | TargetScan Human | Moderate (predicted) | SLC10A4 | solute carrier family 10 member 4 | Plasma Membrane | transporter |
| MIMAT0000738 | miR-383-5p (miRNAs w/seed GAUCAGA) | 2.030 | TargetScan Human | Moderate (predicted) | SLC12A1 | solute carrier family 12 member 1 | Plasma Membrane | transporter |
| MIMAT0000738 | miR-383-5p (miRNAs w/seed GAUCAGA) | 2.030 | TargetScan Human | Moderate (predicted) | SLC22A11 | solute carrier family 22 member 11 | Plasma Membrane | transporter |
| MIMAT0000738 | miR-383-5p (miRNAs w/seed GAUCAGA) | 2.030 | TargetScan Human | Moderate (predicted) | SLC22A5 | solute carrier family 22 member 5 | Plasma Membrane | transporter |
| MIMAT0000738 | miR-383-5p (miRNAs w/seed GAUCAGA) | 2.030 | TargetScan Human | Moderate (predicted) | SLC25A29 | solute carrier family 25 member 29 | Cytoplasm | transporter |
| MIMAT0000738 | miR-383-5p (miRNAs w/seed GAUCAGA) | 2.030 | TargetScan Human | High (predicted) | SLC25A40 | solute carrier family 25 member 40 | Cytoplasm | other |
| MIMAT0000738 | miR-383-5p (miRNAs w/seed GAUCAGA) | 2.030 | TargetScan Human | Moderate (predicted) | SLC2A2 | solute carrier family 2 member 2 | Plasma Membrane | transporter |
| MIMAT0000738 | miR-383-5p (miRNAs w/seed GAUCAGA) | 2.030 | TargetScan Human | Moderate (predicted) | SLC2A5 | solute carrier family 2 member 5 | Plasma Membrane | transporter |
| MIMAT0000738 | miR-383-5p (miRNAs w/seed GAUCAGA) | 2.030 | TargetScan Human | High (predicted) | SLC30A8 | solute carrier family 30 member 8 | Cytoplasm | transporter |
| MIMAT0000738 | miR-383-5p (miRNAs w/seed GAUCAGA) | 2.030 | TargetScan Human | High (predicted) | SLC35A3 | solute carrier family 35 member A3 | Cytoplasm | transporter |
| MIMAT0000738 | miR-383-5p (miRNAs w/seed GAUCAGA) | 2.030 | TargetScan Human | High (predicted) | SLC35D2 | solute carrier family 35 member D2 | Cytoplasm | transporter |
| MIMAT0000738 | miR-383-5p (miRNAs w/seed GAUCAGA) | 2.030 | TargetScan Human | Moderate (predicted) | SLC35F1 | solute carrier family 35 member F1 | Other | other |
| MIMAT0000738 | miR-383-5p (miRNAs w/seed GAUCAGA) | 2.030 | TargetScan Human | Moderate (predicted) | SLC37A3 | solute carrier family 37 member 3 | Cytoplasm | transporter |
| MIMAT0000738 | miR-383-5p (miRNAs w/seed GAUCAGA) | 2.030 | TargetScan Human | Moderate (predicted) | SLC5A9 | solute carrier family 5 member 9 | Plasma Membrane | transporter |
| MIMAT0000738 | miR-383-5p (miRNAs w/seed GAUCAGA) | 2.030 | TargetScan Human | Moderate (predicted) | SLC6A12 | solute carrier family 6 member 12 | Plasma Membrane | transporter |
| MIMAT0000738 | miR-383-5p (miRNAs w/seed GAUCAGA) | 2.030 | TargetScan Human | Moderate (predicted) | SLC9A3R1 | SLC9A3 regulator 1 | Plasma Membrane | other |
| MIMAT0000738 | miR-383-5p (miRNAs w/seed GAUCAGA) | 2.030 | TargetScan Human | Moderate (predicted) | SLFN13 | schlafen family member 13 | Nucleus | enzyme |
| MIMAT0000738 | miR-383-5p (miRNAs w/seed GAUCAGA) | 2.030 | TargetScan Human | Moderate (predicted) | SLMAP | sarcolemma associated protein | Plasma Membrane | other |
| MIMAT0000738 | miR-383-5p (miRNAs w/seed GAUCAGA) | 2.030 | TargetScan Human | High (predicted) | SMC1B | structural maintenance of chromosomes 1 | Nucleus | transporter |
| MIMAT0000738 | miR-383-5p (miRNAs w/seed GAUCAGA) | 2.030 | TargetScan Human | Moderate (predicted) | SMC2 | structural maintenance of chromosomes 2 | Nucleus | transporter |
| MIMAT0000738 | miR-383-5p (miRNAs w/seed GAUCAGA) | 2.030 | TargetScan Human | High (predicted) | SMIM15 | small integral membrane protein 15 | Other | other |
| MIMAT0000738 | miR-383-5p (miRNAs w/seed GAUCAGA) | 2.030 | TargetScan Human | Moderate (predicted) | SMYD1 | SET and MYND domain containing 1 | Nucleus | transcription regulator |
| MIMAT0000738 | miR-383-5p (miRNAs w/seed GAUCAGA) | 2.030 | TargetScan Human | Moderate (predicted) | SNX24 | sorting nexin 24 | Other | transporter |
| MIMAT0000738 | miR-383-5p (miRNAs w/seed GAUCAGA) | 2.030 | TargetScan Human | Moderate (predicted) | SNX31 | sorting nexin 31 | Other | other |
| MIMAT0000738 | miR-383-5p (miRNAs w/seed GAUCAGA) | 2.030 | TargetScan Human | Moderate (predicted) | SOCS2 | suppressor of cytokine signaling 2 | Cytoplasm | other |
| MIMAT0000738 | miR-383-5p (miRNAs w/seed GAUCAGA) | 2.030 | TargetScan Human | Moderate (predicted) | SORCS1 | in related VPS10 domain containing recep | Plasma Membrane | transporter |
| MIMAT0000738 | miR-383-5p (miRNAs w/seed GAUCAGA) | 2.030 | TargetScan Human | Moderate (predicted) | SOS2 | Ras/Rho guanine nucleotide exchange fa | Cytoplasm | other |
| MIMAT0000738 | miR-383-5p (miRNAs w/seed GAUCAGA) | 2.030 | TargetScan Human | Moderate (predicted) | SOX13 | SRY-box transcription factor 13 | Nucleus | transcription regulator |
| MIMAT0000738 | miR-383-5p (miRNAs w/seed GAUCAGA) | 2.030 | TargetScan Human | Moderate (predicted) | SP1 | Sp1 transcription factor | Nucleus | transcription regulator |
| MIMAT0000738 | miR-383-5p (miRNAs w/seed GAUCAGA) | 2.030 | TargetScan Human | Moderate (predicted) | SP6 | Sp6 transcription factor | Nucleus | transcription regulator |
| MIMAT0000738 | miR-383-5p (miRNAs w/seed GAUCAGA) | 2.030 | TargetScan Human | Moderate (predicted) | SPEF2 | sperm flagellar 2 | Cytoplasm | other |
| MIMAT0000738 | miR-383-5p (miRNAs w/seed GAUCAGA) | 2.030 | TargetScan Human | High (predicted) | SPP2 | secreted phosphoprotein 2 | Extracellular Space | other |
| MIMAT0000738 | miR-383-5p (miRNAs w/seed GAUCAGA) | 2.030 | TargetScan Human | Moderate (predicted) | SPRY1 | sprouty RTK signaling antagonist 1 | Cytoplasm | other |
| MIMAT0000738 | miR-383-5p (miRNAs w/seed GAUCAGA) | 2.030 | TargetScan Human | High (predicted) | SPRY3 | sprouty RTK signaling antagonist 3 | Plasma Membrane | other |
| MIMAT0000738 | miR-383-5p (miRNAs w/seed GAUCAGA) | 2.030 | TargetScan Human | Moderate (predicted) | SPTLC3 | e palmitoyltransferase long chain base sub | Cytoplasm | enzyme |
| MIMAT0000738 | miR-383-5p (miRNAs w/seed GAUCAGA) | 2.030 | TargetScan Human | Moderate (predicted) | SRGAP2 | LIT-ROBO Rho GTPase activating protein | Cytoplasm | other |
| MIMAT0000738 | miR-383-5p (miRNAs w/seed GAUCAGA) | 2.030 | TargetScan Human | High (predicted) | SRSF2 | serine and arginine rich splicing factor 2 | Nucleus | transcription regulator |
| MIMAT0000738 | miR-383-5p (miRNAs w/seed GAUCAGA) | 2.030 | TargetScan Human | Moderate (predicted) | SSBP3 | single stranded DNA binding protein 3 | Nucleus | transcription regulator |
| MIMAT0000738 | miR-383-5p (miRNAs w/seed GAUCAGA) | 2.030 | TargetScan Human | Moderate (predicted) | SST | somatostatin | Extracellular Space | other |
| MIMAT0000738 | miR-383-5p (miRNAs w/seed GAUCAGA) | 2.030 | TargetScan Human | Moderate (predicted) | ST8SIA3 | a-N-acetyl-neuraminide alpha-2,8-sialyltra | Cytoplasm | enzyme |
| MIMAT0000738 | miR-383-5p (miRNAs w/seed GAUCAGA) | 2.030 | TargetScan Human | Moderate (predicted) | STEAP2 | STEAP2 metalloreductase | Plasma Membrane | enzyme |
| MIMAT0000738 | miR-383-5p (miRNAs w/seed GAUCAGA) | 2.030 | TargetScan Human | Moderate (predicted) | STK26 | serine/threonine kinase 26 | Nucleus | kinase |
| MIMAT0000738 | miR-383-5p (miRNAs w/seed GAUCAGA) | 2.030 | TargetScan Human | Moderate (predicted) | STRAP | ne/threonine kinase receptor associated pr | Plasma Membrane | other |
| MIMAT0000738 | miR-383-5p (miRNAs w/seed GAUCAGA) | 2.030 | TargetScan Human | Moderate (predicted) | STRBP | spermatid perinuclear RNA binding protein | Cytoplasm | other |
| MIMAT0000738 | miR-383-5p (miRNAs w/seed GAUCAGA) | 2.030 | TargetScan Human | High (predicted) | STRN3 | striatin 3 | Nucleus | transcription regulator |
| MIMAT0000738 | miR-383-5p (miRNAs w/seed GAUCAGA) | 2.030 | TargetScan Human | Moderate (predicted) | STX12 | syntaxin 12 | Cytoplasm | other |
| MIMAT0000738 | miR-383-5p (miRNAs w/seed GAUCAGA) | 2.030 | TargetScan Human | High (predicted) | STYXL1 | serine/threonine/tyrosine interacting like 1 | Cytoplasm | phosphatase |
| MIMAT0000738 | miR-383-5p (miRNAs w/seed GAUCAGA) | 2.030 | TargetScan Human | High (predicted) | SUFU | JFU negative regulator of hedgehog signal | Nucleus | transcription regulator |
| MIMAT0000738 | miR-383-5p (miRNAs w/seed GAUCAGA) | 2.030 | TargetScan Human | Moderate (predicted) | SULT2A1 | sulfotransferase family 2A member 1 | Cytoplasm | enzyme |
| MIMAT0000738 | miR-383-5p (miRNAs w/seed GAUCAGA) | 2.030 | TargetScan Human | High (predicted) | SV2B | synaptic vesicle glycoprotein 2B | Plasma Membrane | transporter |
| MIMAT0000738 | miR-383-5p (miRNAs w/seed GAUCAGA) | 2.030 | TargetScan Human | Moderate (predicted) | SV2C | synaptic vesicle glycoprotein 2C | Plasma Membrane | other |
| MIMAT0000738 | miR-383-5p (miRNAs w/seed GAUCAGA) | 2.030 | TargetScan Human | Moderate (predicted) | SYF2 | SYF2 pre-mRNA splicing factor | Nucleus | other |
| MIMAT0000738 | miR-383-5p (miRNAs w/seed GAUCAGA) | 2.030 | TargetScan Human | Moderate (predicted) | SYT2 | synaptotagmin 2 | Cytoplasm | transporter |
| MIMAT0000738 | miR-383-5p (miRNAs w/seed GAUCAGA) | 2.030 | TargetScan Human | Moderate (predicted) | SYTL2 | synaptotagmin like 2 | Cytoplasm | other |
| MIMAT0000738 | miR-383-5p (miRNAs w/seed GAUCAGA) | 2.030 | TargetScan Human | Moderate (predicted) | TADA1 | transcriptional adaptor 1 | Nucleus | transcription regulator |
| MIMAT0000738 | miR-383-5p (miRNAs w/seed GAUCAGA) | 2.030 | TargetScan Human | Moderate (predicted) | TAF4B | ATA-box binding protein associated factor | Nucleus | transcription regulator |
| MIMAT0000738 | miR-383-5p (miRNAs w/seed GAUCAGA) | 2.030 | TargetScan Human | High (predicted) | TAF9B | ATA-box binding protein associated factor | Nucleus | transcription regulator |
| MIMAT0000738 | miR-383-5p (miRNAs w/seed GAUCAGA) | 2.030 | TargetScan Human | Moderate (predicted) | TAOK3 | TAO kinase 3 | Cytoplasm | kinase |
| MIMAT0000738 | miR-383-5p (miRNAs w/seed GAUCAGA) | 2.030 | TargetScan Human | Moderate (predicted) | TBC1D22B | TBC1 domain family member 22B | Other | other |
| MIMAT0000738 | miR-383-5p (miRNAs w/seed GAUCAGA) | 2.030 | TargetScan Human | Moderate (predicted) | TBC1D28 | TBC1 domain family member 28 | Other | other |
| MIMAT0000738 | miR-383-5p (miRNAs w/seed GAUCAGA) | 2.030 | TargetScan Human | High (predicted) | TBCK | TBC1 domain containing kinase | Cytoplasm | kinase |
| MIMAT0000738 | miR-383-5p (miRNAs w/seed GAUCAGA) | 2.030 | TargetScan Human | High (predicted) | TBX19 | T-box transcription factor 19 | Nucleus | transcription regulator |
| MIMAT0000738 | miR-383-5p (miRNAs w/seed GAUCAGA) | 2.030 | TargetScan Human | Moderate (predicted) | TECRL | trans-2,3-enoyl-CoA reductase like | Cytoplasm | other |
| MIMAT0000738 | miR-383-5p (miRNAs w/seed GAUCAGA) | 2.030 | TargetScan Human | Moderate (predicted) | TEKT1 | tektin 1 | Cytoplasm | other |
| MIMAT0000738 | miR-383-5p (miRNAs w/seed GAUCAGA) | 2.030 | TargetScan Human | Moderate (predicted) | TENT4B | terminal nucleotidyltransferase 4B | Nucleus | enzyme |
| MIMAT0000738 | miR-383-5p (miRNAs w/seed GAUCAGA) | 2.030 | TargetScan Human | Moderate (predicted) | TEX261 | testis expressed 261 | Extracellular Space | other |
| MIMAT0000738 | miR-383-5p (miRNAs w/seed GAUCAGA) | 2.030 | TargetScan Human | Moderate (predicted) | TEX36 | testis expressed 36 | Other | other |
| MIMAT0000738 | miR-383-5p (miRNAs w/seed GAUCAGA) | 2.030 | TargetScan Human | Moderate (predicted) | TFAP2A | transcription factor AP-2 alpha | Nucleus | transcription regulator |
| MIMAT0000738 | miR-383-5p (miRNAs w/seed GAUCAGA) | 2.030 | TargetScan Human | Moderate (predicted) | TFR2 | transferrin receptor 2 | Plasma Membrane | transporter |
| MIMAT0000738 | miR-383-5p (miRNAs w/seed GAUCAGA) | 2.030 | TargetScan Human | Moderate (predicted) | TGOLN2 | trans-golgi network protein 2 | Cytoplasm | other |
| MIMAT0000738 | miR-383-5p (miRNAs w/seed GAUCAGA) | 2.030 | TargetScan Human | High (predicted) | THAP1 | THAP domain containing 1 | Nucleus | transcription regulator |
| MIMAT0000738 | miR-383-5p (miRNAs w/seed GAUCAGA) | 2.030 | TargetScan Human | Moderate (predicted) | THAP10 | THAP domain containing 10 | Other | other |

Sup. Table 3. List of 3699 targets of the seven dysregulated miRNAs obtained from three different target prediction databases,TargetScan TarBase and miRecords considering those predicted as moderate, high, or experimentally validated, respectively.

| | | | | | | | | |
|---|---|---|---|---|---|---|---|---|
| MIMAT0000738 | miR-383-5p (miRNAs w/seed GAUCAGA) | 2.030 | TargetScan Human | Moderate (predicted) | THBS1 | thrombospondin 1 | Extracellular Space | other |
| MIMAT0000738 | miR-383-5p (miRNAs w/seed GAUCAGA) | 2.030 | TargetScan Human | Moderate (predicted) | THTPA | thiamine triphosphatase | Cytoplasm | phosphatase |
| MIMAT0000738 | miR-383-5p (miRNAs w/seed GAUCAGA) | 2.030 | TargetScan Human | Moderate (predicted) | TIRAP | TIR domain containing adaptor protein | Cytoplasm | other |
| MIMAT0000738 | miR-383-5p (miRNAs w/seed GAUCAGA) | 2.030 | TargetScan Human | Moderate (predicted) | TLX1NB | TLX1 neighbor | Other | other |
| MIMAT0000738 | miR-383-5p (miRNAs w/seed GAUCAGA) | 2.030 | TargetScan Human | Moderate (predicted) | TMED9 | transmembrane p24 trafficking protein 9 | Cytoplasm | transporter |
| MIMAT0000738 | miR-383-5p (miRNAs w/seed GAUCAGA) | 2.030 | TargetScan Human | Moderate (predicted) | TMEM117 | transmembrane protein 117 | Cytoplasm | other |
| MIMAT0000738 | miR-383-5p (miRNAs w/seed GAUCAGA) | 2.030 | TargetScan Human | Moderate (predicted) | TMEM138 | transmembrane protein 138 | Extracellular Space | other |
| MIMAT0000738 | miR-383-5p (miRNAs w/seed GAUCAGA) | 2.030 | TargetScan Human | High (predicted) | TMEM154 | transmembrane protein 154 | Other | other |
| MIMAT0000738 | miR-383-5p (miRNAs w/seed GAUCAGA) | 2.030 | TargetScan Human | Moderate (predicted) | TMEM156 | transmembrane protein 156 | Other | other |
| MIMAT0000738 | miR-383-5p (miRNAs w/seed GAUCAGA) | 2.030 | TargetScan Human | Moderate (predicted) | TMEM161B | transmembrane protein 161B | Other | other |
| MIMAT0000738 | miR-383-5p (miRNAs w/seed GAUCAGA) | 2.030 | TargetScan Human | Moderate (predicted) | TMEM168 | transmembrane protein 168 | Cytoplasm | other |
| MIMAT0000738 | miR-383-5p (miRNAs w/seed GAUCAGA) | 2.030 | TargetScan Human | Moderate (predicted) | TMEM169 | transmembrane protein 169 | Other | other |
| MIMAT0000738 | miR-383-5p (miRNAs w/seed GAUCAGA) | 2.030 | TargetScan Human | Moderate (predicted) | TMEM182 | transmembrane protein 182 | Other | other |
| MIMAT0000738 | miR-383-5p (miRNAs w/seed GAUCAGA) | 2.030 | TargetScan Human | Moderate (predicted) | TMEM187 | transmembrane protein 187 | Cytoplasm | other |
| MIMAT0000738 | miR-383-5p (miRNAs w/seed GAUCAGA) | 2.030 | TargetScan Human | High (predicted) | TMEM199 | transmembrane protein 199 | Cytoplasm | other |
| MIMAT0000738 | miR-383-5p (miRNAs w/seed GAUCAGA) | 2.030 | TargetScan Human | Moderate (predicted) | TMEM212 | transmembrane protein 212 | Other | other |
| MIMAT0000738 | miR-383-5p (miRNAs w/seed GAUCAGA) | 2.030 | TargetScan Human | High (predicted) | TMEM251 | transmembrane protein 251 | Other | other |
| MIMAT0000738 | miR-383-5p (miRNAs w/seed GAUCAGA) | 2.030 | TargetScan Human | Moderate (predicted) | TMEM41B | transmembrane protein 41B | Cytoplasm | other |
| MIMAT0000738 | miR-383-5p (miRNAs w/seed GAUCAGA) | 2.030 | TargetScan Human | Moderate (predicted) | TMEM45B | transmembrane protein 45B | Extracellular Space | other |
| MIMAT0000738 | miR-383-5p (miRNAs w/seed GAUCAGA) | 2.030 | TargetScan Human | Moderate (predicted) | TMEM78 | transmembrane protein 78 | Other | other |
| MIMAT0000738 | miR-383-5p (miRNAs w/seed GAUCAGA) | 2.030 | TargetScan Human | Moderate (predicted) | TMEM98 | transmembrane protein 98 | Cytoplasm | other |
| MIMAT0000738 | miR-383-5p (miRNAs w/seed GAUCAGA) | 2.030 | TargetScan Human | Moderate (predicted) | TMIGD3 | mbrane and immunoglobulin domain cont | Plasma Membrane | other |
| MIMAT0000738 | miR-383-5p (miRNAs w/seed GAUCAGA) | 2.030 | TargetScan Human | Moderate (predicted) | TMPRSS3 | transmembrane serine protease 3 | Plasma Membrane | peptidase |
| MIMAT0000738 | miR-383-5p (miRNAs w/seed GAUCAGA) | 2.030 | TargetScan Human | Moderate (predicted) | TNFAIP8 | TNF alpha induced protein 8 | Cytoplasm | other |
| MIMAT0000738 | miR-383-5p (miRNAs w/seed GAUCAGA) | 2.030 | TargetScan Human | High (predicted) | TNFSF12-TNFSF13 | TNFSF12-TNFSF13 readthrough | Other | other |
| MIMAT0000738 | miR-383-5p (miRNAs w/seed GAUCAGA) | 2.030 | TargetScan Human | High (predicted) | TNFSF13 | TNF superfamily member 13 | Extracellular Space | cytokine |
| MIMAT0000738 | miR-383-5p (miRNAs w/seed GAUCAGA) | 2.030 | TargetScan Human | Moderate (predicted) | TNFSF14 | TNF superfamily member 14 | Extracellular Space | cytokine |
| MIMAT0000738 | miR-383-5p (miRNAs w/seed GAUCAGA) | 2.030 | TargetScan Human | Moderate (predicted) | TNFSF15 | TNF superfamily member 15 | Extracellular Space | cytokine |
| MIMAT0000738 | miR-383-5p (miRNAs w/seed GAUCAGA) | 2.030 | TargetScan Human | Moderate (predicted) | TNIP3 | TNFAIP3 interacting protein 3 | Cytoplasm | other |
| MIMAT0000738 | miR-383-5p (miRNAs w/seed GAUCAGA) | 2.030 | TargetScan Human | Moderate (predicted) | TOP2A | DNA topoisomerase II alpha | Nucleus | enzyme |
| MIMAT0000738 | miR-383-5p (miRNAs w/seed GAUCAGA) | 2.030 | TargetScan Human | Moderate (predicted) | TP53INP2 | umor protein p53 inducible nuclear protein | Nucleus | other |
| MIMAT0000738 | miR-383-5p (miRNAs w/seed GAUCAGA) | 2.030 | TargetScan Human | Moderate (predicted) | TRIM10 | tripartite motif containing 10 | Cytoplasm | other |
| MIMAT0000738 | miR-383-5p (miRNAs w/seed GAUCAGA) | 2.030 | TargetScan Human | Moderate (predicted) | TRIM14 | tripartite motif containing 14 | Cytoplasm | other |
| MIMAT0000738 | miR-383-5p (miRNAs w/seed GAUCAGA) | 2.030 | TargetScan Human | High (predicted) | TRIM22 | tripartite motif containing 22 | Cytoplasm | transcription regulator |
| MIMAT0000738 | miR-383-5p (miRNAs w/seed GAUCAGA) | 2.030 | TargetScan Human | Moderate (predicted) | TRIM27 | tripartite motif containing 27 | Nucleus | transcription regulator |
| MIMAT0000738 | miR-383-5p (miRNAs w/seed GAUCAGA) | 2.030 | TargetScan Human | Moderate (predicted) | TRIM44 | tripartite motif containing 44 | Cytoplasm | other |
| MIMAT0000738 | miR-383-5p (miRNAs w/seed GAUCAGA) | 2.030 | TargetScan Human | High (predicted) | TRIM6 | tripartite motif containing 6 | Cytoplasm | enzyme |
| MIMAT0000738 | miR-383-5p (miRNAs w/seed GAUCAGA) | 2.030 | TargetScan Human | Moderate (predicted) | TRIM9 | tripartite motif containing 9 | Cytoplasm | enzyme |
| MIMAT0000738 | miR-383-5p (miRNAs w/seed GAUCAGA) | 2.030 | TargetScan Human | Moderate (predicted) | TRIML1 | tripartite motif family like 1 | Other | other |
| MIMAT0000738 | miR-383-5p (miRNAs w/seed GAUCAGA) | 2.030 | TargetScan Human | Moderate (predicted) | TRIO | rio Rho guanine nucleotide exchange facto | Cytoplasm | kinase |
| MIMAT0000738 | miR-383-5p (miRNAs w/seed GAUCAGA) | 2.030 | TargetScan Human | Moderate (predicted) | TRMT10B | tRNA methyltransferase 10B | Cytoplasm | enzyme |
| MIMAT0000738 | miR-383-5p (miRNAs w/seed GAUCAGA) | 2.030 | TargetScan Human | Moderate (predicted) | TRMT1L | tRNA methyltransferase 1 like | Other | enzyme |
| MIMAT0000738 | miR-383-5p (miRNAs w/seed GAUCAGA) | 2.030 | TargetScan Human | Moderate (predicted) | TSPAN12 | tetraspanin 12 | Plasma Membrane | other |
| MIMAT0000738 | miR-383-5p (miRNAs w/seed GAUCAGA) | 2.030 | TargetScan Human | High (predicted) | TTC1 | tetratricopeptide repeat domain 1 | Cytoplasm | other |
| MIMAT0000738 | miR-383-5p (miRNAs w/seed GAUCAGA) | 2.030 | TargetScan Human | Moderate (predicted) | TTC22 | tetratricopeptide repeat domain 22 | Other | other |
| MIMAT0000738 | miR-383-5p (miRNAs w/seed GAUCAGA) | 2.030 | TargetScan Human | Moderate (predicted) | TTC30B | tetratricopeptide repeat domain 30B | Extracellular Space | other |
| MIMAT0000738 | miR-383-5p (miRNAs w/seed GAUCAGA) | 2.030 | TargetScan Human | Moderate (predicted) | TTLL3 | tubulin tyrosine ligase like 3 | Extracellular Space | enzyme |
| MIMAT0000738 | miR-383-5p (miRNAs w/seed GAUCAGA) | 2.030 | TargetScan Human | High (predicted) | TUBA1A | tubulin alpha 1a | Cytoplasm | other |
| MIMAT0000738 | miR-383-5p (miRNAs w/seed GAUCAGA) | 2.030 | TargetScan Human | Moderate (predicted) | UBA5 | ubiquitin like modifier activating enzyme 5 | Cytoplasm | enzyme |
| MIMAT0000738 | miR-383-5p (miRNAs w/seed GAUCAGA) | 2.030 | TargetScan Human | Moderate (predicted) | UBAC1 | UBA domain containing 1 | Nucleus | other |
| MIMAT0000738 | miR-383-5p (miRNAs w/seed GAUCAGA) | 2.030 | TargetScan Human | Moderate (predicted) | UBL3 | ubiquitin like 3 | Cytoplasm | other |
| MIMAT0000738 | miR-383-5p (miRNAs w/seed GAUCAGA) | 2.030 | TargetScan Human | High (predicted) | UBL4B | ubiquitin like 4B | Other | other |
| MIMAT0000738 | miR-383-5p (miRNAs w/seed GAUCAGA) | 2.030 | TargetScan Human | Moderate (predicted) | UBR7 | uitin protein ligase E3 component n-recog | Other | enzyme |
| MIMAT0000738 | miR-383-5p (miRNAs w/seed GAUCAGA) | 2.030 | TargetScan Human | Moderate (predicted) | UBXN10 | UBX domain protein 10 | Extracellular Space | other |
| MIMAT0000738 | miR-383-5p (miRNAs w/seed GAUCAGA) | 2.030 | TargetScan Human | Moderate (predicted) | UMODL1 | uromodulin like 1 | Cytoplasm | other |
| MIMAT0000738 | miR-383-5p (miRNAs w/seed GAUCAGA) | 2.030 | TargetScan Human | Moderate (predicted) | UPF3B | B regulator of nonsense mediated mRNA | Nucleus | transporter |
| MIMAT0000738 | miR-383-5p (miRNAs w/seed GAUCAGA) | 2.030 | TargetScan Human | Moderate (predicted) | UPP2 | uridine phosphorylase 2 | Cytoplasm | enzyme |
| MIMAT0000738 | miR-383-5p (miRNAs w/seed GAUCAGA) | 2.030 | TargetScan Human | Moderate (predicted) | VEGFA | vascular endothelial growth factor A | Extracellular Space | growth factor |
| MIMAT0000738 | miR-383-5p (miRNAs w/seed GAUCAGA) | 2.030 | TargetScan Human | Moderate (predicted) | VEZT | tin, adherens junctions transmembrane pr | Plasma Membrane | other |
| MIMAT0000738 | miR-383-5p (miRNAs w/seed GAUCAGA) | 2.030 | TargetScan Human | High (predicted) | VPS35 | VPS35 retromer complex component | Cytoplasm | transporter |
| MIMAT0000738 | miR-383-5p (miRNAs w/seed GAUCAGA) | 2.030 | TargetScan Human | High (predicted) | VPS52 | VPS52 subunit of GARP complex | Cytoplasm | other |
| MIMAT0000738 | miR-383-5p (miRNAs w/seed GAUCAGA) | 2.030 | TargetScan Human | Moderate (predicted) | VPS54 | VPS54 subunit of GARP complex | Extracellular Space | other |
| MIMAT0000738 | miR-383-5p (miRNAs w/seed GAUCAGA) | 2.030 | TargetScan Human | Moderate (predicted) | VPS72 | vacuolar protein sorting 72 homolog | Nucleus | transcription regulator |
| MIMAT0000738 | miR-383-5p (miRNAs w/seed GAUCAGA) | 2.030 | TargetScan Human | Moderate (predicted) | VSTM2A | set and transmembrane domain containing | Extracellular Space | other |
| MIMAT0000738 | miR-383-5p (miRNAs w/seed GAUCAGA) | 2.030 | TargetScan Human | Moderate (predicted) | VTCN1 | t domain containing T cell activation inhib | Plasma Membrane | other |
| MIMAT0000738 | miR-383-5p (miRNAs w/seed GAUCAGA) | 2.030 | TargetScan Human | Moderate (predicted) | VWC2 | on Willebrand factor C domain containing | Extracellular Space | other |
| MIMAT0000738 | miR-383-5p (miRNAs w/seed GAUCAGA) | 2.030 | TargetScan Human | Moderate (predicted) | WAC | W domain containing adaptor with coiled-c | Nucleus | other |
| MIMAT0000738 | miR-383-5p (miRNAs w/seed GAUCAGA) | 2.030 | TargetScan Human | Moderate (predicted) | WASF3 | WASP family member 3 | Cytoplasm | other |
| MIMAT0000738 | miR-383-5p (miRNAs w/seed GAUCAGA) | 2.030 | TargetScan Human | Moderate (predicted) | WBP2 | WW domain binding protein 2 | Cytoplasm | transcription regulator |
| MIMAT0000738 | miR-383-5p (miRNAs w/seed GAUCAGA) | 2.030 | TargetScan Human | Moderate (predicted) | WDR44 | WD repeat domain 44 | Cytoplasm | other |
| MIMAT0000738 | miR-383-5p (miRNAs w/seed GAUCAGA) | 2.030 | TargetScan Human | Moderate (predicted) | WDR7 | WD repeat domain 7 | Cytoplasm | other |
| MIMAT0000738 | miR-383-5p (miRNAs w/seed GAUCAGA) | 2.030 | TargetScan Human | Moderate (predicted) | WIPI2 | repeat domain, phosphoinositide interacti | Cytoplasm | other |
| MIMAT0000738 | miR-383-5p (miRNAs w/seed GAUCAGA) | 2.030 | TargetScan Human | High (predicted) | WNK4 | WNK lysine deficient protein kinase 4 | Plasma Membrane | kinase |
| MIMAT0000738 | miR-383-5p (miRNAs w/seed GAUCAGA) | 2.030 | TargetScan Human | High (predicted) | WNT16 | Wnt family member 16 | Extracellular Space | other |
| MIMAT0000738 | miR-383-5p (miRNAs w/seed GAUCAGA) | 2.030 | TargetScan Human | Moderate (predicted) | WNT2 | Wnt family member 2 | Extracellular Space | cytokine |
| MIMAT0000738 | miR-383-5p (miRNAs w/seed GAUCAGA) | 2.030 | TargetScan Human | Moderate (predicted) | XKRX | XK related X-linked | Other | other |
| MIMAT0000738 | miR-383-5p (miRNAs w/seed GAUCAGA) | 2.030 | TargetScan Human | Moderate (predicted) | YIPF5 | Yip1 domain family member 5 | Cytoplasm | other |
| MIMAT0000738 | miR-383-5p (miRNAs w/seed GAUCAGA) | 2.030 | TargetScan Human | Moderate (predicted) | YIPF7 | Yip1 domain family member 7 | Other | other |
| MIMAT0000738 | miR-383-5p (miRNAs w/seed GAUCAGA) | 2.030 | TargetScan Human | High (predicted) | YOD1 | YOD1 deubiquitinase | Cytoplasm | enzyme |
| MIMAT0000738 | miR-383-5p (miRNAs w/seed GAUCAGA) | 2.030 | TargetScan Human | High (predicted) | YWHAH | ygenase/tryptophan 5-monooxygenase ac | Cytoplasm | transcription regulator |
| MIMAT0000738 | miR-383-5p (miRNAs w/seed GAUCAGA) | 2.030 | TargetScan Human | Moderate (predicted) | ZBTB33 | zinc finger and BTB domain containing 33 | Nucleus | transcription regulator |
| MIMAT0000738 | miR-383-5p (miRNAs w/seed GAUCAGA) | 2.030 | TargetScan Human | Moderate (predicted) | ZC2HC1A | zinc finger C2HC-type containing 1A | Other | other |
| MIMAT0000738 | miR-383-5p (miRNAs w/seed GAUCAGA) | 2.030 | TargetScan Human | Moderate (predicted) | ZC3H12B | zinc finger CCCH-type containing 12B | Other | other |
| MIMAT0000738 | miR-383-5p (miRNAs w/seed GAUCAGA) | 2.030 | TargetScan Human | Moderate (predicted) | ZC3H12C | zinc finger CCCH-type containing 12C | Other | other |
| MIMAT0000738 | miR-383-5p (miRNAs w/seed GAUCAGA) | 2.030 | TargetScan Human | Moderate (predicted) | ZDHHC17 | nc finger DHHC-type palmitoyltransferase | Cytoplasm | enzyme |
| MIMAT0000738 | miR-383-5p (miRNAs w/seed GAUCAGA) | 2.030 | TargetScan Human | Moderate (predicted) | ZFHX3 | zinc finger homeobox 3 | Nucleus | transcription regulator |
| MIMAT0000738 | miR-383-5p (miRNAs w/seed GAUCAGA) | 2.030 | TargetScan Human | Moderate (predicted) | ZFP41 | ZFP41 zinc finger protein | Nucleus | transcription regulator |
| MIMAT0000738 | miR-383-5p (miRNAs w/seed GAUCAGA) | 2.030 | TargetScan Human | Moderate (predicted) | ZHX1 | zinc fingers and homeoboxes 1 | Nucleus | transcription regulator |
| MIMAT0000738 | miR-383-5p (miRNAs w/seed GAUCAGA) | 2.030 | TargetScan Human | Moderate (predicted) | ZIC1 | Zic family member 1 | Nucleus | transcription regulator |
| MIMAT0000738 | miR-383-5p (miRNAs w/seed GAUCAGA) | 2.030 | TargetScan Human | Moderate (predicted) | ZIC3 | Zic family member 3 | Nucleus | transcription regulator |
| MIMAT0000738 | miR-383-5p (miRNAs w/seed GAUCAGA) | 2.030 | TargetScan Human | Moderate (predicted) | ZIK1 | inc finger protein interacting with K protein | Other | other |
| MIMAT0000738 | miR-383-5p (miRNAs w/seed GAUCAGA) | 2.030 | TargetScan Human | Moderate (predicted) | ZKSCAN8 | inc finger with KRAB and SCAN domains | Nucleus | transcription regulator |
| MIMAT0000738 | miR-383-5p (miRNAs w/seed GAUCAGA) | 2.030 | TargetScan Human | Moderate (predicted) | ZMAT4 | zinc finger matrin-type 4 | Nucleus | other |
| MIMAT0000738 | miR-383-5p (miRNAs w/seed GAUCAGA) | 2.030 | TargetScan Human | Moderate (predicted) | ZNF185 | zinc finger protein 185 with LIM domain | Plasma Membrane | other |
| MIMAT0000738 | miR-383-5p (miRNAs w/seed GAUCAGA) | 2.030 | TargetScan Human | Moderate (predicted) | ZNF300 | zinc finger protein 300 | Nucleus | transcription regulator |
| MIMAT0000738 | miR-383-5p (miRNAs w/seed GAUCAGA) | 2.030 | TargetScan Human | Moderate (predicted) | ZNF331 | zinc finger protein 331 | Nucleus | transcription regulator |
| MIMAT0000738 | miR-383-5p (miRNAs w/seed GAUCAGA) | 2.030 | TargetScan Human | High (predicted) | ZNF410 | zinc finger protein 410 | Nucleus | transcription regulator |
| MIMAT0000738 | miR-383-5p (miRNAs w/seed GAUCAGA) | 2.030 | TargetScan Human | Moderate (predicted) | ZNF460 | zinc finger protein 460 | Nucleus | transcription regulator |
| MIMAT0000738 | miR-383-5p (miRNAs w/seed GAUCAGA) | 2.030 | TargetScan Human | Moderate (predicted) | ZNF496 | zinc finger protein 496 | Nucleus | transcription regulator |
| MIMAT0000738 | miR-383-5p (miRNAs w/seed GAUCAGA) | 2.030 | TargetScan Human | Moderate (predicted) | ZNF557 | zinc finger protein 557 | Nucleus | transcription regulator |
| MIMAT0000738 | miR-383-5p (miRNAs w/seed GAUCAGA) | 2.030 | TargetScan Human | Moderate (predicted) | ZNF581 | zinc finger protein 581 | Nucleus | transcription regulator |
| MIMAT0000738 | miR-383-5p (miRNAs w/seed GAUCAGA) | 2.030 | TargetScan Human | Moderate (predicted) | ZNF677 | zinc finger protein 677 | Other | other |
| MIMAT0000738 | miR-383-5p (miRNAs w/seed GAUCAGA) | 2.030 | TargetScan Human | Moderate (predicted) | ZNF681 | zinc finger protein 681 | Other | other |
| MIMAT0000738 | miR-383-5p (miRNAs w/seed GAUCAGA) | 2.030 | TargetScan Human | Moderate (predicted) | ZNF765 | zinc finger protein 765 | Other | other |
| MIMAT0000738 | miR-383-5p (miRNAs w/seed GAUCAGA) | 2.030 | TargetScan Human | Moderate (predicted) | ZNF774 | zinc finger protein 774 | Other | other |
| MIMAT0000738 | miR-383-5p (miRNAs w/seed GAUCAGA) | 2.030 | TargetScan Human | Moderate (predicted) | ZNF790 | zinc finger protein 790 | Other | other |
| MIMAT0000738 | miR-383-5p (miRNAs w/seed GAUCAGA) | 2.030 | TargetScan Human | High (predicted) | ZNF814 | zinc finger protein 814 | Other | other |
| MIMAT0000738 | miR-383-5p (miRNAs w/seed GAUCAGA) | 2.030 | TargetScan Human | Moderate (predicted) | ZNF829 | zinc finger protein 829 | Other | other |
| MIMAT0000738 | miR-383-5p (miRNAs w/seed GAUCAGA) | 2.030 | TargetScan Human | Moderate (predicted) | ZNF83 | zinc finger protein 83 | Nucleus | transcription regulator |
| MIMAT0000738 | miR-383-5p (miRNAs w/seed GAUCAGA) | 2.030 | TargetScan Human | Moderate (predicted) | ZSCAN23 | inc finger and SCAN domain containing 2 | Cytoplasm | transcription regulator |
| MIMAT0000738 | miR-383-5p (miRNAs w/seed GAUCAGA) | 2.030 | TargetScan Human | Moderate (predicted) | ZXDB | zinc finger X-linked duplicated B | Nucleus | transcription regulator |
| MIMAT0003948 | miR-4712-5p (and other miRNAs w/seed CCAGUAC) | -15.630 | TargetScan Human | Moderate (predicted) | A4GNT | alpha-1,4-N-acetylglucosaminyltransferas | Cytoplasm | enzyme |
| MIMAT0003948 | miR-4712-5p (and other miRNAs w/seed CCAGUAC) | -15.630 | TargetScan Human | Moderate (predicted) | AADACL3 | arylacetamide deacetylase like 3 | Other | other |
| MIMAT0003948 | miR-4712-5p (and other miRNAs w/seed CCAGUAC) | -15.630 | TargetScan Human | Moderate (predicted) | ABHD12B | abhydrolase domain containing 12B | Other | peptidase |
| MIMAT0003948 | miR-4712-5p (and other miRNAs w/seed CCAGUAC) | -15.630 | TargetScan Human | Moderate (predicted) | ABI1 | abl interactor 1 | Cytoplasm | other |
| MIMAT0003948 | miR-4712-5p (and other miRNAs w/seed CCAGUAC) | -15.630 | TargetScan Human | High (predicted) | AC0040769 | | Other | other |
| MIMAT0003948 | miR-4712-5p (and other miRNAs w/seed CCAGUAC) | -15.630 | TargetScan Human | High (predicted) | AC005008.2 | | Other | other |
| MIMAT0003948 | miR-4712-5p (and other miRNAs w/seed CCAGUAC) | -15.630 | TargetScan Human | Moderate (predicted) | ACBD5 | acyl-CoA binding domain containing 5 | Nucleus | other |
| MIMAT0003948 | miR-4712-5p (and other miRNAs w/seed CCAGUAC) | -15.630 | TargetScan Human | Moderate (predicted) | ADAM22 | ADAM metallopeptidase domain 22 | Plasma Membrane | peptidase |
| MIMAT0003948 | miR-4712-5p (and other miRNAs w/seed CCAGUAC) | -15.630 | TargetScan Human | High (predicted) | ADGRV1 | adhesion G protein-coupled receptor V1 | Plasma Membrane | G-protein coupled receptor |
| MIMAT0003948 | miR-4712-5p (and other miRNAs w/seed CCAGUAC) | -15.630 | TargetScan Human | Moderate (predicted) | AFF3 | AF4/FMR2 family member 3 | Nucleus | transcription regulator |
| MIMAT0003948 | miR-4712-5p (and other miRNAs w/seed CCAGUAC) | -15.630 | TargetScan Human | High (predicted) | AGER | nced glycosylation end-product specific re | Plasma Membrane | transmembrane receptor |
| MIMAT0003948 | miR-4712-5p (and other miRNAs w/seed CCAGUAC) | -15.630 | TargetScan Human | Moderate (predicted) | AGFG1 | ArfGAP with FG repeats 1 | Nucleus | other |
| MIMAT0003948 | miR-4712-5p (and other miRNAs w/seed CCAGUAC) | -15.630 | TargetScan Human | High (predicted) | AGTR2 | angiotensin II receptor type 2 | Plasma Membrane | G-protein coupled receptor |
| MIMAT0003948 | miR-4712-5p (and other miRNAs w/seed CCAGUAC) | -15.630 | TargetScan Human | Moderate (predicted) | ALG10B | ALG10 alpha-1,2-glucosyltransferase B | Plasma Membrane | transporter |
| MIMAT0003948 | miR-4712-5p (and other miRNAs w/seed CCAGUAC) | -15.630 | TargetScan Human | High (predicted) | AMIGO2 | adhesion molecule with Ig like domain 2 | Plasma Membrane | other |
| MIMAT0003948 | miR-4712-5p (and other miRNAs w/seed CCAGUAC) | -15.630 | TargetScan Human | Moderate (predicted) | ANHX | anomalous homeobox | Other | other |
| MIMAT0003948 | miR-4712-5p (and other miRNAs w/seed CCAGUAC) | -15.630 | TargetScan Human | Moderate (predicted) | ANKAR | ankyrin and armadillo repeat containing | Nucleus | transcription regulator |
| MIMAT0003948 | miR-4712-5p (and other miRNAs w/seed CCAGUAC) | -15.630 | TargetScan Human | Moderate (predicted) | ANKRD23 | ankyrin repeat domain 23 | Nucleus | other |
| MIMAT0003948 | miR-4712-5p (and other miRNAs w/seed CCAGUAC) | -15.630 | TargetScan Human | Moderate (predicted) | ANKRD40 | ankyrin repeat domain 40 | Other | other |
| MIMAT0003948 | miR-4712-5p (and other miRNAs w/seed CCAGUAC) | -15.630 | TargetScan Human | Moderate (predicted) | ANP32A | ic nuclear phosphoprotein 32 family meml | Cytoplasm | other |
| MIMAT0003948 | miR-4712-5p (and other miRNAs w/seed CCAGUAC) | -15.630 | TargetScan Human | Moderate (predicted) | AP3M2 | aptor related protein complex 3 subunit m | Cytoplasm | transporter |
| MIMAT0003948 | miR-4712-5p (and other miRNAs w/seed CCAGUAC) | -15.630 | TargetScan Human | Moderate (predicted) | AQP4 | aquaporin 4 | Plasma Membrane | transporter |
| MIMAT0003948 | miR-4712-5p (and other miRNAs w/seed CCAGUAC) | -15.630 | TargetScan Human | High (predicted) | ARHGAP12 | Rho GTPase activating protein 12 | Cytoplasm | other |
| MIMAT0003948 | miR-4712-5p (and other miRNAs w/seed CCAGUAC) | -15.630 | TargetScan Human | Moderate (predicted) | ARPC2 | actin related protein 2/3 complex subunit 2 | Cytoplasm | other |
| MIMAT0003948 | miR-4712-5p (and other miRNAs w/seed CCAGUAC) | -15.630 | TargetScan Human | Moderate (predicted) | ARPP19 | cAMP regulated phosphoprotein 19 | Cytoplasm | transporter |
| MIMAT0003948 | miR-4712-5p (and other miRNAs w/seed CCAGUAC) | -15.630 | TargetScan Human | High (predicted) | ASAH2 | N-acylsphingosine amidohydrolase 2 | Cytoplasm | enzyme |
| MIMAT0003948 | miR-4712-5p (and other miRNAs w/seed CCAGUAC) | -15.630 | TargetScan Human | Moderate (predicted) | ASAH2B | N-acylsphingosine amidohydrolase 2B | Cytoplasm | other |
| MIMAT0003948 | miR-4712-5p (and other miRNAs w/seed CCAGUAC) | -15.630 | TargetScan Human | Moderate (predicted) | ASAP3 | with SH3 domain, ankyrin repeat and PH d | Plasma Membrane | other |
| MIMAT0003948 | miR-4712-5p (and other miRNAs w/seed CCAGUAC) | -15.630 | TargetScan Human | Moderate (predicted) | ASPA | aspartoacylase | Cytoplasm | enzyme |
| MIMAT0003948 | miR-4712-5p (and other miRNAs w/seed CCAGUAC) | -15.630 | TargetScan Human | Moderate (predicted) | ATP6V0C | ATPase H+ transporting V0 subunit c | Cytoplasm | transporter |
| MIMAT0003948 | miR-4712-5p (and other miRNAs w/seed CCAGUAC) | -15.630 | TargetScan Human | Moderate (predicted) | BACE1 | beta-secretase 1 | Cytoplasm | peptidase |
| MIMAT0003948 | miR-4712-5p (and other miRNAs w/seed CCAGUAC) | -15.630 | TargetScan Human | Moderate (predicted) | BAZ1A | omodomain adjacent to zinc finger domain | Nucleus | other |
| MIMAT0003948 | miR-4712-5p (and other miRNAs w/seed CCAGUAC) | -15.630 | TargetScan Human | Moderate (predicted) | BCAN | brevican | Extracellular Space | other |
| MIMAT0003948 | miR-4712-5p (and other miRNAs w/seed CCAGUAC) | -15.630 | TargetScan Human | Moderate (predicted) | BICC1 | BicC family RNA binding protein 1 | Other | other |
| MIMAT0003948 | miR-4712-5p (and other miRNAs w/seed CCAGUAC) | -15.630 | TargetScan Human | Moderate (predicted) | BMP6 | bone morphogenetic protein 6 | Extracellular Space | growth factor |

Sup. Table 3. List of 3699 targets of the seven dysregulated miRNAs obtained from three different target prediction databases,TargetScan TarBase and miRecords considering those predicted as moderate, high, or experimentally validated, respectively.

| | | | | | | | | |
|---|---|---|---|---|---|---|---|---|
| MIMAT0003948 | miR-4712-5p (and other miRNAs w/seed CCAGUAC) | -15.630 | TargetScan Human | Moderate (predicted) | BNIP2 | BCL2 interacting protein 2 | Cytoplasm | other |
| MIMAT0003948 | miR-4712-5p (and other miRNAs w/seed CCAGUAC) | -15.630 | TargetScan Human | Moderate (predicted) | BTD | biotinidase | Extracellular Space | enzyme |
| MIMAT0003948 | miR-4712-5p (and other miRNAs w/seed CCAGUAC) | -15.630 | TargetScan Human | Moderate (predicted) | BTF3L4 | basic transcription factor 3 like 4 | Other | other |
| MIMAT0003948 | miR-4712-5p (and other miRNAs w/seed CCAGUAC) | -15.630 | TargetScan Human | High (predicted) | BTG1 | BTG anti-proliferation factor 1 | Nucleus | transcription regulator |
| MIMAT0003948 | miR-4712-5p (and other miRNAs w/seed CCAGUAC) | -15.630 | TargetScan Human | Moderate (predicted) | C11orf42 | chromosome 11 open reading frame 42 | Other | other |
| MIMAT0003948 | miR-4712-5p (and other miRNAs w/seed CCAGUAC) | -15.630 | TargetScan Human | High (predicted) | C12orf42 | chromosome 12 open reading frame 42 | Other | other |
| MIMAT0003948 | miR-4712-5p (and other miRNAs w/seed CCAGUAC) | -15.630 | TargetScan Human | Moderate (predicted) | C17orf102 | chromosome 17 open reading frame 102 | Other | other |
| MIMAT0003948 | miR-4712-5p (and other miRNAs w/seed CCAGUAC) | -15.630 | TargetScan Human | Moderate (predicted) | C1orf131 | chromosome 1 open reading frame 131 | Nucleus | other |
| MIMAT0003948 | miR-4712-5p (and other miRNAs w/seed CCAGUAC) | -15.630 | TargetScan Human | Moderate (predicted) | C4orf17 | chromosome 4 open reading frame 17 | Other | other |
| MIMAT0003948 | miR-4712-5p (and other miRNAs w/seed CCAGUAC) | -15.630 | TargetScan Human | Moderate (predicted) | C8orf44-SGK3/SGK3 | /glucocorticoid regulated kinase family me | Cytoplasm | kinase |
| MIMAT0003948 | miR-4712-5p (and other miRNAs w/seed CCAGUAC) | -15.630 | TargetScan Human | Moderate (predicted) | CAPN1 | calpain 1 | Cytoplasm | peptidase |
| MIMAT0003948 | miR-4712-5p (and other miRNAs w/seed CCAGUAC) | -15.630 | TargetScan Human | Moderate (predicted) | CARM1 | tivator associated arginine methyltransfera | Nucleus | transcription regulator |
| MIMAT0003948 | miR-4712-5p (and other miRNAs w/seed CCAGUAC) | -15.630 | TargetScan Human | Moderate (predicted) | CARNS1 | carnosine synthase 1 | Cytoplasm | enzyme |
| MIMAT0003948 | miR-4712-5p (and other miRNAs w/seed CCAGUAC) | -15.630 | TargetScan Human | Moderate (predicted) | CAV2 | caveolin 2 | Plasma Membrane | other |
| MIMAT0003948 | miR-4712-5p (and other miRNAs w/seed CCAGUAC) | -15.630 | TargetScan Human | Moderate (predicted) | CBFB | core-binding factor subunit beta | Nucleus | transcription regulator |
| MIMAT0003948 | miR-4712-5p (and other miRNAs w/seed CCAGUAC) | -15.630 | TargetScan Human | Moderate (predicted) | CCDC102B | coiled-coil domain containing 102B | Extracellular Space | other |
| MIMAT0003948 | miR-4712-5p (and other miRNAs w/seed CCAGUAC) | -15.630 | TargetScan Human | Moderate (predicted) | CCDC122 | coiled-coil domain containing 122 | Other | other |
| MIMAT0003948 | miR-4712-5p (and other miRNAs w/seed CCAGUAC) | -15.630 | TargetScan Human | Moderate (predicted) | CCDC39 | coiled-coil domain containing 39 | Cytoplasm | other |
| MIMAT0003948 | miR-4712-5p (and other miRNAs w/seed CCAGUAC) | -15.630 | TargetScan Human | High (predicted) | CCDC60 | coiled-coil domain containing 60 | Other | other |
| MIMAT0003948 | miR-4712-5p (and other miRNAs w/seed CCAGUAC) | -15.630 | TargetScan Human | Moderate (predicted) | CCDC85A | coiled-coil domain containing 85A | Other | other |
| MIMAT0003948 | miR-4712-5p (and other miRNAs w/seed CCAGUAC) | -15.630 | TargetScan Human | Moderate (predicted) | CCND2 | cyclin D2 | Nucleus | other |
| MIMAT0003948 | miR-4712-5p (and other miRNAs w/seed CCAGUAC) | -15.630 | TargetScan Human | Moderate (predicted) | CCNG2 | cyclin G2 | Nucleus | other |
| MIMAT0003948 | miR-4712-5p (and other miRNAs w/seed CCAGUAC) | -15.630 | TargetScan Human | Moderate (predicted) | CCNO | cyclin O | Nucleus | other |
| MIMAT0003948 | miR-4712-5p (and other miRNAs w/seed CCAGUAC) | -15.630 | TargetScan Human | Moderate (predicted) | CCNT1 | cyclin T1 | Nucleus | transcription regulator |
| MIMAT0003948 | miR-4712-5p (and other miRNAs w/seed CCAGUAC) | -15.630 | TargetScan Human | Moderate (predicted) | CCNY | cyclin Y | Nucleus | other |
| MIMAT0003948 | miR-4712-5p (and other miRNAs w/seed CCAGUAC) | -15.630 | TargetScan Human | Moderate (predicted) | CCT7 | chaperonin containing TCP1 subunit 7 | Cytoplasm | other |
| MIMAT0003948 | miR-4712-5p (and other miRNAs w/seed CCAGUAC) | -15.630 | TargetScan Human | Moderate (predicted) | CCZ1/CCZ1B | B, vacuolar protein trafficking and biogen | Cytoplasm | other |
| MIMAT0003948 | miR-4712-5p (and other miRNAs w/seed CCAGUAC) | -15.630 | TargetScan Human | Moderate (predicted) | CD247 | CD247 molecule | Plasma Membrane | transmembrane receptor |
| MIMAT0003948 | miR-4712-5p (and other miRNAs w/seed CCAGUAC) | -15.630 | TargetScan Human | Moderate (predicted) | CD2BP2 | CD2 cytoplasmic tail binding protein 2 | Cytoplasm | other |
| MIMAT0003948 | miR-4712-5p (and other miRNAs w/seed CCAGUAC) | -15.630 | TargetScan Human | Moderate (predicted) | CDC42EP3 | CDC42 effector protein 3 | Cytoplasm | other |
| MIMAT0003948 | miR-4712-5p (and other miRNAs w/seed CCAGUAC) | -15.630 | TargetScan Human | Moderate (predicted) | CDKAL1 | 5 regulatory subunit associated protein 1 l | Cytoplasm | enzyme |
| MIMAT0003948 | miR-4712-5p (and other miRNAs w/seed CCAGUAC) | -15.630 | TargetScan Human | Moderate (predicted) | CDKL4 | cyclin dependent kinase like 4 | Nucleus | kinase |
| MIMAT0003948 | miR-4712-5p (and other miRNAs w/seed CCAGUAC) | -15.630 | TargetScan Human | High (predicted) | CDR1 | cerebellar degeneration related protein 1 | Other | other |
| MIMAT0003948 | miR-4712-5p (and other miRNAs w/seed CCAGUAC) | -15.630 | TargetScan Human | Moderate (predicted) | CEACAM18 | CEA cell adhesion molecule 18 | Other | other |
| MIMAT0003948 | miR-4712-5p (and other miRNAs w/seed CCAGUAC) | -15.630 | TargetScan Human | Moderate (predicted) | CEBPZOS | CEBPZ opposite strand | Other | other |
| MIMAT0003948 | miR-4712-5p (and other miRNAs w/seed CCAGUAC) | -15.630 | TargetScan Human | Moderate (predicted) | CFAP70 | cilia and flagella associated protein 70 | Cytoplasm | other |
| MIMAT0003948 | miR-4712-5p (and other miRNAs w/seed CCAGUAC) | -15.630 | TargetScan Human | Moderate (predicted) | CHMP4B | charged multivesicular body protein 4B | Cytoplasm | other |
| MIMAT0003948 | miR-4712-5p (and other miRNAs w/seed CCAGUAC) | -15.630 | TargetScan Human | Moderate (predicted) | CHSY3 | chondroitin sulfate synthase 3 | Cytoplasm | enzyme |
| MIMAT0003948 | miR-4712-5p (and other miRNAs w/seed CCAGUAC) | -15.630 | TargetScan Human | High (predicted) | CIB4 | alcium and integrin binding family member | Other | other |
| MIMAT0003948 | miR-4712-5p (and other miRNAs w/seed CCAGUAC) | -15.630 | TargetScan Human | Moderate (predicted) | CISD2 | CDGSH iron sulfur domain 2 | Cytoplasm | other |
| MIMAT0003948 | miR-4712-5p (and other miRNAs w/seed CCAGUAC) | -15.630 | TargetScan Human | High (predicted) | CKS1B | CDC28 protein kinase regulatory subunit 1 | Other | kinase |
| MIMAT0003948 | miR-4712-5p (and other miRNAs w/seed CCAGUAC) | -15.630 | TargetScan Human | High (predicted) | CLEC4A | C-type lectin domain family 4 member A | Plasma Membrane | transmembrane receptor |
| MIMAT0003948 | miR-4712-5p (and other miRNAs w/seed CCAGUAC) | -15.630 | TargetScan Human | Moderate (predicted) | CLEC4M | C-type lectin domain family 4 member M | Plasma Membrane | other |
| MIMAT0003948 | miR-4712-5p (and other miRNAs w/seed CCAGUAC) | -15.630 | TargetScan Human | Moderate (predicted) | CNDP1 | carnosine dipeptidase 1 | Cytoplasm | peptidase |
| MIMAT0003948 | miR-4712-5p (and other miRNAs w/seed CCAGUAC) | -15.630 | TargetScan Human | High (predicted) | CNEP1R1 | clear envelope phosphatase 1 regulatory s | Nucleus | other |
| MIMAT0003948 | miR-4712-5p (and other miRNAs w/seed CCAGUAC) | -15.630 | TargetScan Human | Moderate (predicted) | CNOT6L | CR4-NOT transcription complex subunit 6 | Cytoplasm | enzyme |
| MIMAT0003948 | miR-4712-5p (and other miRNAs w/seed CCAGUAC) | -15.630 | TargetScan Human | Moderate (predicted) | CNR1 | cannabinoid receptor 1 | Plasma Membrane | G-protein coupled receptor |
| MIMAT0003948 | miR-4712-5p (and other miRNAs w/seed CCAGUAC) | -15.630 | TargetScan Human | Moderate (predicted) | CNTF | ciliary neurotrophic factor | Extracellular Space | cytokine |
| MIMAT0003948 | miR-4712-5p (and other miRNAs w/seed CCAGUAC) | -15.630 | TargetScan Human | Moderate (predicted) | CNTN4 | contactin 4 | Plasma Membrane | enzyme |
| MIMAT0003948 | miR-4712-5p (and other miRNAs w/seed CCAGUAC) | -15.630 | TargetScan Human | Moderate (predicted) | COL24A1 | collagen type XXIV alpha 1 chain | Extracellular Space | other |
| MIMAT0003948 | miR-4712-5p (and other miRNAs w/seed CCAGUAC) | -15.630 | TargetScan Human | Moderate (predicted) | COLCA1 | colorectal cancer associated 1 | Other | other |
| MIMAT0003948 | miR-4712-5p (and other miRNAs w/seed CCAGUAC) | -15.630 | TargetScan Human | Moderate (predicted) | COLQ | like tail subunit of asymmetric acetylcholir | Extracellular Space | other |
| MIMAT0003948 | miR-4712-5p (and other miRNAs w/seed CCAGUAC) | -15.630 | TargetScan Human | High (predicted) | COX11 | ochrome c oxidase copper chaperone CO | Cytoplasm | enzyme |
| MIMAT0003948 | miR-4712-5p (and other miRNAs w/seed CCAGUAC) | -15.630 | TargetScan Human | Moderate (predicted) | CPEB2 | asmic polyadenylation element binding pr | Cytoplasm | translation regulator |
| MIMAT0003948 | miR-4712-5p (and other miRNAs w/seed CCAGUAC) | -15.630 | TargetScan Human | High (predicted) | CPEB3 | asmic polyadenylation element binding pr | Cytoplasm | translation regulator |
| MIMAT0003948 | miR-4712-5p (and other miRNAs w/seed CCAGUAC) | -15.630 | TargetScan Human | High (predicted) | CRNN | cornulin | Cytoplasm | other |
| MIMAT0003948 | miR-4712-5p (and other miRNAs w/seed CCAGUAC) | -15.630 | TargetScan Human | Moderate (predicted) | CSGALNACT1 | roitin sulfate N-acetylgalactosaminyltransfe | Cytoplasm | enzyme |
| MIMAT0003948 | miR-4712-5p (and other miRNAs w/seed CCAGUAC) | -15.630 | TargetScan Human | Moderate (predicted) | CSRNP3 | cysteine and serine rich nuclear protein 3 | Nucleus | transcription regulator |
| MIMAT0003948 | miR-4712-5p (and other miRNAs w/seed CCAGUAC) | -15.630 | TargetScan Human | Moderate (predicted) | CTPS1 | CTP synthase 1 | Nucleus | enzyme |
| MIMAT0003948 | miR-4712-5p (and other miRNAs w/seed CCAGUAC) | -15.630 | TargetScan Human | Moderate (predicted) | CXCL11 | C-X-C motif chemokine ligand 11 | Extracellular Space | cytokine |
| MIMAT0003948 | miR-4712-5p (and other miRNAs w/seed CCAGUAC) | -15.630 | TargetScan Human | Moderate (predicted) | CXorf65 | chromosome X open reading frame 65 | Other | other |
| MIMAT0003948 | miR-4712-5p (and other miRNAs w/seed CCAGUAC) | -15.630 | TargetScan Human | Moderate (predicted) | CYB561D1 | cytochrome b561 family member D1 | Other | other |
| MIMAT0003948 | miR-4712-5p (and other miRNAs w/seed CCAGUAC) | -15.630 | TargetScan Human | Moderate (predicted) | CYB5D2 | cytochrome b5 domain containing 2 | Extracellular Space | other |
| MIMAT0003948 | miR-4712-5p (and other miRNAs w/seed CCAGUAC) | -15.630 | TargetScan Human | High (predicted) | CYP3A5 | ochrome P450 family 3 subfamily A memb | Cytoplasm | enzyme |
| MIMAT0003948 | miR-4712-5p (and other miRNAs w/seed CCAGUAC) | -15.630 | TargetScan Human | Moderate (predicted) | DCAF12L1 | DDB1 and CUL4 associated factor 12 like | Extracellular Space | other |
| MIMAT0003948 | miR-4712-5p (and other miRNAs w/seed CCAGUAC) | -15.630 | TargetScan Human | Moderate (predicted) | DCAF4L1 | DDB1 and CUL4 associated factor 4 like 1 | Other | other |
| MIMAT0003948 | miR-4712-5p (and other miRNAs w/seed CCAGUAC) | -15.630 | TargetScan Human | High (predicted) | DCN | decorin | Extracellular Space | other |
| MIMAT0003948 | miR-4712-5p (and other miRNAs w/seed CCAGUAC) | -15.630 | TargetScan Human | High (predicted) | DCUN1D1 | ctive in cullin neddylation 1 domain contair | Nucleus | other |
| MIMAT0003948 | miR-4712-5p (and other miRNAs w/seed CCAGUAC) | -15.630 | TargetScan Human | Moderate (predicted) | DCUN1D3 | ctive in cullin neddylation 1 domain contair | Cytoplasm | other |
| MIMAT0003948 | miR-4712-5p (and other miRNAs w/seed CCAGUAC) | -15.630 | TargetScan Human | Moderate (predicted) | DEUP1 | deuterosome assembly protein 1 | Other | other |
| MIMAT0003948 | miR-4712-5p (and other miRNAs w/seed CCAGUAC) | -15.630 | TargetScan Human | Moderate (predicted) | DHTKD1 | genase E1 and transketolase domain con | Cytoplasm | enzyme |
| MIMAT0003948 | miR-4712-5p (and other miRNAs w/seed CCAGUAC) | -15.630 | TargetScan Human | Moderate (predicted) | DIAPH3 | diaphanous related formin 3 | Cytoplasm | enzyme |
| MIMAT0003948 | miR-4712-5p (and other miRNAs w/seed CCAGUAC) | -15.630 | TargetScan Human | Moderate (predicted) | DLG1 | discs large MAGUK scaffold protein 1 | Plasma Membrane | kinase |
| MIMAT0003948 | miR-4712-5p (and other miRNAs w/seed CCAGUAC) | -15.630 | TargetScan Human | Moderate (predicted) | DNER | delta/notch like EGF repeat containing | Plasma Membrane | transmembrane receptor |
| MIMAT0003948 | miR-4712-5p (and other miRNAs w/seed CCAGUAC) | -15.630 | TargetScan Human | Moderate (predicted) | DTX1 | deltex E3 ubiquitin ligase 1 | Nucleus | transcription regulator |
| MIMAT0003948 | miR-4712-5p (and other miRNAs w/seed CCAGUAC) | -15.630 | TargetScan Human | Moderate (predicted) | DYNC1LI2 | nein cytoplasmic 1 light intermediate chai | Cytoplasm | other |
| MIMAT0003948 | miR-4712-5p (and other miRNAs w/seed CCAGUAC) | -15.630 | TargetScan Human | High (predicted) | ECI2 | enoyl-CoA delta isomerase 2 | Cytoplasm | enzyme |
| MIMAT0003948 | miR-4712-5p (and other miRNAs w/seed CCAGUAC) | -15.630 | TargetScan Human | Moderate (predicted) | EDA2R | ectodysplasin A2 receptor | Plasma Membrane | transmembrane receptor |
| MIMAT0003948 | miR-4712-5p (and other miRNAs w/seed CCAGUAC) | -15.630 | TargetScan Human | Moderate (predicted) | EEF1AKMT2 | EEF1A lysine methyltransferase 2 | Cytoplasm | enzyme |
| MIMAT0003948 | miR-4712-5p (and other miRNAs w/seed CCAGUAC) | -15.630 | TargetScan Human | Moderate (predicted) | EFHC1 | EF-hand domain containing 1 | Cytoplasm | other |
| MIMAT0003948 | miR-4712-5p (and other miRNAs w/seed CCAGUAC) | -15.630 | TargetScan Human | Moderate (predicted) | EIF2AK4 | yotic translation initiation factor 2 alpha kir | Cytoplasm | kinase |
| MIMAT0003948 | miR-4712-5p (and other miRNAs w/seed CCAGUAC) | -15.630 | TargetScan Human | High (predicted) | EIF4G3 | karyotic translation initiation factor 4 gamm | Cytoplasm | translation regulator |
| MIMAT0003948 | miR-4712-5p (and other miRNAs w/seed CCAGUAC) | -15.630 | TargetScan Human | Moderate (predicted) | ELL2 | elongation factor for RNA polymerase II 2 | Nucleus | transcription regulator |
| MIMAT0003948 | miR-4712-5p (and other miRNAs w/seed CCAGUAC) | -15.630 | TargetScan Human | Moderate (predicted) | ELOVL4 | ELOVL fatty acid elongase 4 | Cytoplasm | enzyme |
| MIMAT0003948 | miR-4712-5p (and other miRNAs w/seed CCAGUAC) | -15.630 | TargetScan Human | Moderate (predicted) | EMX2 | empty spiracles homeobox 2 | Nucleus | transcription regulator |
| MIMAT0003948 | miR-4712-5p (and other miRNAs w/seed CCAGUAC) | -15.630 | TargetScan Human | Moderate (predicted) | EPB41L5 | rythrocyte membrane protein band 4.1 like | Plasma Membrane | other |
| MIMAT0003948 | miR-4712-5p (and other miRNAs w/seed CCAGUAC) | -15.630 | TargetScan Human | Moderate (predicted) | EPC2 | enhancer of polycomb homolog 2 | Other | other |
| MIMAT0003948 | miR-4712-5p (and other miRNAs w/seed CCAGUAC) | -15.630 | TargetScan Human | Moderate (predicted) | EPHB1 | EPH receptor B1 | Plasma Membrane | kinase |
| MIMAT0003948 | miR-4712-5p (and other miRNAs w/seed CCAGUAC) | -15.630 | TargetScan Human | Moderate (predicted) | EVC | EvC ciliary complex subunit 1 | Cytoplasm | other |
| MIMAT0003948 | miR-4712-5p (and other miRNAs w/seed CCAGUAC) | -15.630 | TargetScan Human | Moderate (predicted) | FAM169A | mily with sequence similarity 169 member | Other | other |
| MIMAT0003948 | miR-4712-5p (and other miRNAs w/seed CCAGUAC) | -15.630 | TargetScan Human | Moderate (predicted) | FAM170A | mily with sequence similarity 170 member | Nucleus | other |
| MIMAT0003948 | miR-4712-5p (and other miRNAs w/seed CCAGUAC) | -15.630 | TargetScan Human | Moderate (predicted) | FAM76A | amily with sequence similarity 76 member | Nucleus | other |
| MIMAT0003948 | miR-4712-5p (and other miRNAs w/seed CCAGUAC) | -15.630 | TargetScan Human | Moderate (predicted) | FBXW7 | F-box and WD repeat domain containing 7 | Nucleus | enzyme |
| MIMAT0003948 | miR-4712-5p (and other miRNAs w/seed CCAGUAC) | -15.630 | TargetScan Human | Moderate (predicted) | FCRL5 | Fc receptor like 5 | Plasma Membrane | other |
| MIMAT0003948 | miR-4712-5p (and other miRNAs w/seed CCAGUAC) | -15.630 | TargetScan Human | Moderate (predicted) | FGD2 | YVE, RhoGEF and PH domain containing | Cytoplasm | other |
| MIMAT0003948 | miR-4712-5p (and other miRNAs w/seed CCAGUAC) | -15.630 | TargetScan Human | Moderate (predicted) | FGF14 | fibroblast growth factor 14 | Extracellular Space | growth factor |
| MIMAT0003948 | miR-4712-5p (and other miRNAs w/seed CCAGUAC) | -15.630 | TargetScan Human | Moderate (predicted) | FHAD1 | ad associated phosphopeptide binding do | Other | other |
| MIMAT0003948 | miR-4712-5p (and other miRNAs w/seed CCAGUAC) | -15.630 | TargetScan Human | Moderate (predicted) | FLI1 | i-1 proto-oncogene, ETS transcription fact | Nucleus | transcription regulator |
| MIMAT0003948 | miR-4712-5p (and other miRNAs w/seed CCAGUAC) | -15.630 | TargetScan Human | Moderate (predicted) | FLJ40288 | uncharacterized FLJ40288 | Other | other |
| MIMAT0003948 | miR-4712-5p (and other miRNAs w/seed CCAGUAC) | -15.630 | TargetScan Human | Moderate (predicted) | FMO1 | in containing dimethylaniline monoxygena | Cytoplasm | enzyme |
| MIMAT0003948 | miR-4712-5p (and other miRNAs w/seed CCAGUAC) | -15.630 | TargetScan Human | Moderate (predicted) | FOXA3 | forkhead box A3 | Nucleus | transcription regulator |
| MIMAT0003948 | miR-4712-5p (and other miRNAs w/seed CCAGUAC) | -15.630 | TargetScan Human | Moderate (predicted) | GAB1 | GRB2 associated binding protein 1 | Cytoplasm | other |
| MIMAT0003948 | miR-4712-5p (and other miRNAs w/seed CCAGUAC) | -15.630 | TargetScan Human | Moderate (predicted) | GBP6 | guanylate binding protein family member 6 | Cytoplasm | enzyme |
| MIMAT0003948 | miR-4712-5p (and other miRNAs w/seed CCAGUAC) | -15.630 | TargetScan Human | Moderate (predicted) | GCLC | glutamate-cysteine ligase catalytic subuni | Cytoplasm | enzyme |
| MIMAT0003948 | miR-4712-5p (and other miRNAs w/seed CCAGUAC) | -15.630 | TargetScan Human | High (predicted) | GCSAML | inal center associated signaling and motili | Other | other |
| MIMAT0003948 | miR-4712-5p (and other miRNAs w/seed CCAGUAC) | -15.630 | TargetScan Human | Moderate (predicted) | GEMIN8 | gem nuclear organelle associated protein 8 | Cytoplasm | other |
| MIMAT0003948 | miR-4712-5p (and other miRNAs w/seed CCAGUAC) | -15.630 | TargetScan Human | High (predicted) | GKN2 | gastrokine 2 | Extracellular Space | other |
| MIMAT0003948 | miR-4712-5p (and other miRNAs w/seed CCAGUAC) | -15.630 | TargetScan Human | Moderate (predicted) | GLB1L3 | galactosidase beta 1 like 3 | Other | enzyme |
| MIMAT0003948 | miR-4712-5p (and other miRNAs w/seed CCAGUAC) | -15.630 | TargetScan Human | Moderate (predicted) | GLCCI1 | glucocorticoid induced 1 | Cytoplasm | other |
| MIMAT0003948 | miR-4712-5p (and other miRNAs w/seed CCAGUAC) | -15.630 | TargetScan Human | Moderate (predicted) | GLYAT | glycine-N-acyltransferase | Cytoplasm | enzyme |
| MIMAT0003948 | miR-4712-5p (and other miRNAs w/seed CCAGUAC) | -15.630 | TargetScan Human | High (predicted) | GMFB | glia maturation factor beta | Cytoplasm | growth factor |
| MIMAT0003948 | miR-4712-5p (and other miRNAs w/seed CCAGUAC) | -15.630 | TargetScan Human | Moderate (predicted) | LGA6L6 (includes oth | golgin A6 family like 6 | Other | other |
| MIMAT0003948 | miR-4712-5p (and other miRNAs w/seed CCAGUAC) | -15.630 | TargetScan Human | Moderate (predicted) | LGA6L9 (includes oth | golgin A6 family like 9 | Other | other |
| MIMAT0003948 | miR-4712-5p (and other miRNAs w/seed CCAGUAC) | -15.630 | TargetScan Human | Moderate (predicted) | GORASP1 | golgi reassembly stacking protein 1 | Cytoplasm | other |
| MIMAT0003948 | miR-4712-5p (and other miRNAs w/seed CCAGUAC) | -15.630 | TargetScan Human | Moderate (predicted) | GPD1L | glycerol-3-phosphate dehydrogenase 1 like | Cytoplasm | enzyme |
| MIMAT0003948 | miR-4712-5p (and other miRNAs w/seed CCAGUAC) | -15.630 | TargetScan Human | Moderate (predicted) | GPR50 | G protein-coupled receptor 50 | Plasma Membrane | G-protein coupled receptor |
| MIMAT0003948 | miR-4712-5p (and other miRNAs w/seed CCAGUAC) | -15.630 | TargetScan Human | Moderate (predicted) | GRIA2 | amate ionotropic receptor AMPA type subu | Plasma Membrane | ion channel |
| MIMAT0003948 | miR-4712-5p (and other miRNAs w/seed CCAGUAC) | -15.630 | TargetScan Human | Moderate (predicted) | GSDMA | gasdermin A | Cytoplasm | other |
| MIMAT0003948 | miR-4712-5p (and other miRNAs w/seed CCAGUAC) | -15.630 | TargetScan Human | Moderate (predicted) | GTDC1 | lycosyltransferase like domain containing | Other | other |
| MIMAT0003948 | miR-4712-5p (and other miRNAs w/seed CCAGUAC) | -15.630 | TargetScan Human | Moderate (predicted) | GTF3C1 | general transcription factor IIIC subunit 1 | Nucleus | transcription regulator |
| MIMAT0003948 | miR-4712-5p (and other miRNAs w/seed CCAGUAC) | -15.630 | TargetScan Human | Moderate (predicted) | GTF3C2 | general transcription factor IIIC subunit 2 | Nucleus | transcription regulator |
| MIMAT0003948 | miR-4712-5p (and other miRNAs w/seed CCAGUAC) | -15.630 | TargetScan Human | Moderate (predicted) | HAVCR1 | hepatitis A virus cellular receptor 1 | Plasma Membrane | other |
| MIMAT0003948 | miR-4712-5p (and other miRNAs w/seed CCAGUAC) | -15.630 | TargetScan Human | Moderate (predicted) | HEBP1 | heme binding protein 1 | Cytoplasm | other |
| MIMAT0003948 | miR-4712-5p (and other miRNAs w/seed CCAGUAC) | -15.630 | TargetScan Human | Moderate (predicted) | HKDC1 | hexokinase domain containing 1 | Cytoplasm | kinase |
| MIMAT0003948 | miR-4712-5p (and other miRNAs w/seed CCAGUAC) | -15.630 | TargetScan Human | High (predicted) | HLA-DOA | r histocompatibility complex, class II, DO a | Plasma Membrane | transmembrane receptor |
| MIMAT0003948 | miR-4712-5p (and other miRNAs w/seed CCAGUAC) | -15.630 | TargetScan Human | Moderate (predicted) | HOXA10-HOXA9 | | Other | other |
| MIMAT0003948 | miR-4712-5p (and other miRNAs w/seed CCAGUAC) | -15.630 | TargetScan Human | Moderate (predicted) | HOXA9 | homeobox A9 | Nucleus | transcription regulator |
| MIMAT0003948 | miR-4712-5p (and other miRNAs w/seed CCAGUAC) | -15.630 | TargetScan Human | Moderate (predicted) | HPGD | 15-hydroxyprostaglandin dehydrogenase | Cytoplasm | enzyme |
| MIMAT0003948 | miR-4712-5p (and other miRNAs w/seed CCAGUAC) | -15.630 | TargetScan Human | Moderate (predicted) | HS2ST1 | heparan sulfate 2-O-sulfotransferase 1 | Cytoplasm | enzyme |
| MIMAT0003948 | miR-4712-5p (and other miRNAs w/seed CCAGUAC) | -15.630 | TargetScan Human | Moderate (predicted) | HSBP1 | heat shock factor binding protein 1 | Nucleus | other |
| MIMAT0003948 | miR-4712-5p (and other miRNAs w/seed CCAGUAC) | -15.630 | TargetScan Human | Moderate (predicted) | HSPA4 | eat shock protein family A (Hsp70) membe | Cytoplasm | other |
| MIMAT0003948 | miR-4712-5p (and other miRNAs w/seed CCAGUAC) | -15.630 | TargetScan Human | Moderate (predicted) | HTN1 | histatin 1 | Plasma Membrane | other |
| MIMAT0003948 | miR-4712-5p (and other miRNAs w/seed CCAGUAC) | -15.630 | TargetScan Human | Moderate (predicted) | HTN3 | histatin 3 | Extracellular Space | other |
| MIMAT0003948 | miR-4712-5p (and other miRNAs w/seed CCAGUAC) | -15.630 | TargetScan Human | Moderate (predicted) | HUS1 | HUS1 checkpoint clamp component | Nucleus | kinase |
| MIMAT0003948 | miR-4712-5p (and other miRNAs w/seed CCAGUAC) | -15.630 | TargetScan Human | Moderate (predicted) | HYPK | huntingtin interacting protein K | Cytoplasm | other |
| MIMAT0003948 | miR-4712-5p (and other miRNAs w/seed CCAGUAC) | -15.630 | TargetScan Human | Moderate (predicted) | IARS2 | soleucyl-tRNA synthetase 2, mitochondria | Cytoplasm | enzyme |
| MIMAT0003948 | miR-4712-5p (and other miRNAs w/seed CCAGUAC) | -15.630 | TargetScan Human | Moderate (predicted) | IDH3G | hydrogenase (NAD(+)) 3 non-catalytic sub | Cytoplasm | enzyme |
| MIMAT0003948 | miR-4712-5p (and other miRNAs w/seed CCAGUAC) | -15.630 | TargetScan Human | Moderate (predicted) | IGF2BP2 | lin like growth factor 2 mRNA binding prot | Cytoplasm | translation regulator |
| MIMAT0003948 | miR-4712-5p (and other miRNAs w/seed CCAGUAC) | -15.630 | TargetScan Human | Moderate (predicted) | IHH | Indian hedgehog signaling molecule | Extracellular Space | enzyme |
| MIMAT0003948 | miR-4712-5p (and other miRNAs w/seed CCAGUAC) | -15.630 | TargetScan Human | Moderate (predicted) | IKZF4 | IKAROS family zinc finger 4 | Nucleus | transcription regulator |
| MIMAT0003948 | miR-4712-5p (and other miRNAs w/seed CCAGUAC) | -15.630 | TargetScan Human | Moderate (predicted) | IL11 | interleukin 11 | Extracellular Space | cytokine |
| MIMAT0003948 | miR-4712-5p (and other miRNAs w/seed CCAGUAC) | -15.630 | TargetScan Human | Moderate (predicted) | IL31RA | interleukin 31 receptor A | Plasma Membrane | transmembrane receptor |
| MIMAT0003948 | miR-4712-5p (and other miRNAs w/seed CCAGUAC) | -15.630 | TargetScan Human | Moderate (predicted) | IL36RN | interleukin 36 receptor antagonist | Extracellular Space | cytokine |
| MIMAT0003948 | miR-4712-5p (and other miRNAs w/seed CCAGUAC) | -15.630 | TargetScan Human | Moderate (predicted) | INHBA | inhibin subunit beta A | Extracellular Space | growth factor |
| MIMAT0003948 | miR-4712-5p (and other miRNAs w/seed CCAGUAC) | -15.630 | TargetScan Human | High (predicted) | INO80D | INO80 complex subunit D | Nucleus | other |
| MIMAT0003948 | miR-4712-5p (and other miRNAs w/seed CCAGUAC) | -15.630 | TargetScan Human | Moderate (predicted) | IP6K2 | inositol hexakisphosphate kinase 2 | Cytoplasm | kinase |

Sup. Table 3. List of 3699 targets of the seven dysregulated miRNAs obtained from three different target prediction databases,TargetScan TarBase and miRecords considering those predicted as moderate, high, or experimentally validated, respectively.

| | | | | | | | | |
|---|---|---|---|---|---|---|---|---|
| MIMAT0003948 | miR-4712-5p (and other miRNAs w/seed CCAGUAC) | -15.630 | TargetScan Human | Moderate (predicted) | IRF2 | interferon regulatory factor 2 | Nucleus | transcription regulator |
| MIMAT0003948 | miR-4712-5p (and other miRNAs w/seed CCAGUAC) | -15.630 | TargetScan Human | High (predicted) | IST1 | IST1 factor associated with ESCRT-III | Cytoplasm | other |
| MIMAT0003948 | miR-4712-5p (and other miRNAs w/seed CCAGUAC) | -15.630 | TargetScan Human | Moderate (predicted) | ITPRIPL2 | ITPRIP like 2 | Other | other |
| MIMAT0003948 | miR-4712-5p (and other miRNAs w/seed CCAGUAC) | -15.630 | TargetScan Human | Moderate (predicted) | KAT2B | lysine acetyltransferase 2B | Nucleus | transcription regulator |
| MIMAT0003948 | miR-4712-5p (and other miRNAs w/seed CCAGUAC) | -15.630 | TargetScan Human | High (predicted) | KCNJ1 | m inwardly rectifying channel subfamily J r | Plasma Membrane | ion channel |
| MIMAT0003948 | miR-4712-5p (and other miRNAs w/seed CCAGUAC) | -15.630 | TargetScan Human | High (predicted) | KCNJ6 | m inwardly rectifying channel subfamily J r | Plasma Membrane | ion channel |
| MIMAT0003948 | miR-4712-5p (and other miRNAs w/seed CCAGUAC) | -15.630 | TargetScan Human | Moderate (predicted) | KCNK1 | m two pore domain channel subfamily K m | Plasma Membrane | ion channel |
| MIMAT0003948 | miR-4712-5p (and other miRNAs w/seed CCAGUAC) | -15.630 | TargetScan Human | High (predicted) | KCNMB2 | m-activated channel subfamily M regulato | Plasma Membrane | ion channel |
| MIMAT0003948 | miR-4712-5p (and other miRNAs w/seed CCAGUAC) | -15.630 | TargetScan Human | Moderate (predicted) | KICS2 | KICSTOR subunit 2 | Plasma Membrane | other |
| MIMAT0003948 | miR-4712-5p (and other miRNAs w/seed CCAGUAC) | -15.630 | TargetScan Human | Moderate (predicted) | KLF14 | Kruppel like factor 14 | Nucleus | transcription regulator |
| MIMAT0003948 | miR-4712-5p (and other miRNAs w/seed CCAGUAC) | -15.630 | TargetScan Human | Moderate (predicted) | KLHL42 | kelch like family member 42 | Nucleus | enzyme |
| MIMAT0003948 | miR-4712-5p (and other miRNAs w/seed CCAGUAC) | -15.630 | TargetScan Human | Moderate (predicted) | KLRC3 | killer cell lectin like receptor C3 | Plasma Membrane | transmembrane receptor |
| MIMAT0003948 | miR-4712-5p (and other miRNAs w/seed CCAGUAC) | -15.630 | TargetScan Human | Moderate (predicted) | KNTC1 | kinetochore associated 1 | Nucleus | other |
| MIMAT0003948 | miR-4712-5p (and other miRNAs w/seed CCAGUAC) | -15.630 | TargetScan Human | Moderate (predicted) | KRT75 | keratin 75 | Cytoplasm | other |
| MIMAT0003948 | miR-4712-5p (and other miRNAs w/seed CCAGUAC) | -15.630 | TargetScan Human | Moderate (predicted) | KRTAP13-2 | keratin associated protein 13-2 | Cytoplasm | other |
| MIMAT0003948 | miR-4712-5p (and other miRNAs w/seed CCAGUAC) | -15.630 | TargetScan Human | High (predicted) | KRTAP3-2 | keratin associated protein 3-2 | Cytoplasm | other |
| MIMAT0003948 | miR-4712-5p (and other miRNAs w/seed CCAGUAC) | -15.630 | TargetScan Human | Moderate (predicted) | LATS2 | large tumor suppressor kinase 2 | Nucleus | kinase |
| MIMAT0003948 | miR-4712-5p (and other miRNAs w/seed CCAGUAC) | -15.630 | TargetScan Human | Moderate (predicted) | LBR | lamin B receptor | Nucleus | enzyme |
| MIMAT0003948 | miR-4712-5p (and other miRNAs w/seed CCAGUAC) | -15.630 | TargetScan Human | Moderate (predicted) | LCOR | and dependent nuclear receptor corepress | Nucleus | transcription regulator |
| MIMAT0003948 | miR-4712-5p (and other miRNAs w/seed CCAGUAC) | -15.630 | TargetScan Human | High (predicted) | LDAH | lipid droplet associated hydrolase | Cytoplasm | enzyme |
| MIMAT0003948 | miR-4712-5p (and other miRNAs w/seed CCAGUAC) | -15.630 | TargetScan Human | High (predicted) | LGALS3BP | galectin 3 binding protein | Plasma Membrane | transmembrane receptor |
| MIMAT0003948 | miR-4712-5p (and other miRNAs w/seed CCAGUAC) | -15.630 | TargetScan Human | Moderate (predicted) | LIMK2 | LIM domain kinase 2 | Cytoplasm | kinase |
| MIMAT0003948 | miR-4712-5p (and other miRNAs w/seed CCAGUAC) | -15.630 | TargetScan Human | Moderate (predicted) | LIN28A | lin-28 homolog A | Cytoplasm | other |
| MIMAT0003948 | miR-4712-5p (and other miRNAs w/seed CCAGUAC) | -15.630 | TargetScan Human | Moderate (predicted) | LINC00239 | ong intergenic non-protein coding RNA 23 | Other | other |
| MIMAT0003948 | miR-4712-5p (and other miRNAs w/seed CCAGUAC) | -15.630 | TargetScan Human | Moderate (predicted) | LINC01560 | ong intergenic non-protein coding RNA 15 | Other | other |
| MIMAT0003948 | miR-4712-5p (and other miRNAs w/seed CCAGUAC) | -15.630 | TargetScan Human | Moderate (predicted) | LINC02108 | ong intergenic non-protein coding RNA 21 | Other | other |
| MIMAT0003948 | miR-4712-5p (and other miRNAs w/seed CCAGUAC) | -15.630 | TargetScan Human | Moderate (predicted) | LPGAT1 | lysophosphatidylglycerol acyltransferase 1 | Cytoplasm | enzyme |
| MIMAT0003948 | miR-4712-5p (and other miRNAs w/seed CCAGUAC) | -15.630 | TargetScan Human | Moderate (predicted) | LPIN1 | lipin 1 | Nucleus | phosphatase |

Sup. Table 3. List of 3699 targets of the seven dysregulated miRNAs obtained from three different target prediction databases,TargetScan TarBase and miRecords considering those predicted as moderate, high, or experimentally validated, respectively.

| | | | | | | | | |
|---|---|---|---|---|---|---|---|---|
| MIMAT0003948 | miR-4712-5p (and other miRNAs w/seed CCAGUAC) | -15.630 | TargetScan Human | Moderate (predicted) | LRFN5 | ch repeat and fibronectin type III domain cc | Nucleus | other |
| MIMAT0003948 | miR-4712-5p (and other miRNAs w/seed CCAGUAC) | -15.630 | TargetScan Human | Moderate (predicted) | LRP6 | LDL receptor related protein 6 | Plasma Membrane | transmembrane receptor |
| MIMAT0003948 | miR-4712-5p (and other miRNAs w/seed CCAGUAC) | -15.630 | TargetScan Human | Moderate (predicted) | LRRC38 | leucine rich repeat containing 38 | Plasma Membrane | other |
| MIMAT0003948 | miR-4712-5p (and other miRNAs w/seed CCAGUAC) | -15.630 | TargetScan Human | Moderate (predicted) | LRRC58 | leucine rich repeat containing 58 | Other | other |
| MIMAT0003948 | miR-4712-5p (and other miRNAs w/seed CCAGUAC) | -15.630 | TargetScan Human | Moderate (predicted) | LUZP4 | leucine zipper protein 4 | Nucleus | other |
| MIMAT0003948 | miR-4712-5p (and other miRNAs w/seed CCAGUAC) | -15.630 | TargetScan Human | High (predicted) | LUZP6 | leucine zipper protein 6 | Other | other |
| MIMAT0003948 | miR-4712-5p (and other miRNAs w/seed CCAGUAC) | -15.630 | TargetScan Human | Moderate (predicted) | LYPD5 | LY6/PLAUR domain containing 5 | Other | other |
| MIMAT0003948 | miR-4712-5p (and other miRNAs w/seed CCAGUAC) | -15.630 | TargetScan Human | Moderate (predicted) | LYRM2 | LYR motif containing 2 | Cytoplasm | other |
| MIMAT0003948 | miR-4712-5p (and other miRNAs w/seed CCAGUAC) | -15.630 | TargetScan Human | High (predicted) | MAGT1 | magnesium transporter 1 | Plasma Membrane | enzyme |
| MIMAT0003948 | miR-4712-5p (and other miRNAs w/seed CCAGUAC) | -15.630 | TargetScan Human | High (predicted) | MAOA | monoamine oxidase A | Cytoplasm | enzyme |
| MIMAT0003948 | miR-4712-5p (and other miRNAs w/seed CCAGUAC) | -15.630 | TargetScan Human | Moderate (predicted) | MAP3K1 | ogen-activated protein kinase kinase kinas | Cytoplasm | kinase |
| MIMAT0003948 | miR-4712-5p (and other miRNAs w/seed CCAGUAC) | -15.630 | TargetScan Human | Moderate (predicted) | MARCKS | stoylated alanine rich protein kinase C sub | Plasma Membrane | other |
| MIMAT0003948 | miR-4712-5p (and other miRNAs w/seed CCAGUAC) | -15.630 | TargetScan Human | Moderate (predicted) | MARK1 | microtubule affinity regulating kinase 1 | Cytoplasm | kinase |
| MIMAT0003948 | miR-4712-5p (and other miRNAs w/seed CCAGUAC) | -15.630 | TargetScan Human | High (predicted) | MATN2 | matrilin 2 | Extracellular Space | other |
| MIMAT0003948 | miR-4712-5p (and other miRNAs w/seed CCAGUAC) | -15.630 | TargetScan Human | Moderate (predicted) | MCCC2 | methylcrotonoyl-CoA carboxylase 2 | Cytoplasm | enzyme |
| MIMAT0003948 | miR-4712-5p (and other miRNAs w/seed CCAGUAC) | -15.630 | TargetScan Human | Moderate (predicted) | MDFIC | MyoD family inhibitor domain containing | Nucleus | other |
| MIMAT0003948 | miR-4712-5p (and other miRNAs w/seed CCAGUAC) | -15.630 | TargetScan Human | Moderate (predicted) | MDM4 | MDM4 regulator of p53 | Nucleus | transcription regulator |
| MIMAT0003948 | miR-4712-5p (and other miRNAs w/seed CCAGUAC) | -15.630 | TargetScan Human | Moderate (predicted) | MED20 | mediator complex subunit 20 | Nucleus | transcription regulator |
| MIMAT0003948 | miR-4712-5p (and other miRNAs w/seed CCAGUAC) | -15.630 | TargetScan Human | High (predicted) | MED6 | mediator complex subunit 6 | Nucleus | transcription regulator |
| MIMAT0003948 | miR-4712-5p (and other miRNAs w/seed CCAGUAC) | -15.630 | TargetScan Human | High (predicted) | MESP1 | esoderm posterior bHLH transcription facto | Nucleus | transcription regulator |
| MIMAT0003948 | miR-4712-5p (and other miRNAs w/seed CCAGUAC) | -15.630 | TargetScan Human | Moderate (predicted) | METTL15 | methyltransferase like 15 | Other | other |
| MIMAT0003948 | miR-4712-5p (and other miRNAs w/seed CCAGUAC) | -15.630 | TargetScan Human | Moderate (predicted) | MFSD10 | or facilitator superfamily domain containin | Nucleus | transporter |
| MIMAT0003948 | miR-4712-5p (and other miRNAs w/seed CCAGUAC) | -15.630 | TargetScan Human | High (predicted) | MINDY4 | MINDY lysine 48 deubiquitinase 4 | Other | other |
| MIMAT0003948 | miR-4712-5p (and other miRNAs w/seed CCAGUAC) | -15.630 | TargetScan Human | Moderate (predicted) | MLEC | malectin | Plasma Membrane | other |
| MIMAT0003948 | miR-4712-5p (and other miRNAs w/seed CCAGUAC) | -15.630 | TargetScan Human | High (predicted) | MNX1 | motor neuron and pancreas homeobox 1 | Nucleus | transcription regulator |
| MIMAT0003948 | miR-4712-5p (and other miRNAs w/seed CCAGUAC) | -15.630 | TargetScan Human | Moderate (predicted) | MOG | myelin oligodendrocyte glycoprotein | Extracellular Space | other |
| MIMAT0003948 | miR-4712-5p (and other miRNAs w/seed CCAGUAC) | -15.630 | TargetScan Human | Moderate (predicted) | MRC1 | mannose receptor C-type 1 | Plasma Membrane | transmembrane receptor |
| MIMAT0003948 | miR-4712-5p (and other miRNAs w/seed CCAGUAC) | -15.630 | TargetScan Human | High (predicted) | MRPL15 | mitochondrial ribosomal protein L15 | Cytoplasm | other |
| MIMAT0003948 | miR-4712-5p (and other miRNAs w/seed CCAGUAC) | -15.630 | TargetScan Human | Moderate (predicted) | MRPS27 | mitochondrial ribosomal protein S27 | Cytoplasm | other |
| MIMAT0003948 | miR-4712-5p (and other miRNAs w/seed CCAGUAC) | -15.630 | TargetScan Human | Moderate (predicted) | MSMO1 | methylsterol monooxygenase 1 | Cytoplasm | enzyme |
| MIMAT0003948 | miR-4712-5p (and other miRNAs w/seed CCAGUAC) | -15.630 | TargetScan Human | Moderate (predicted) | MSTO1 | itochondrial distribution and morphology r | Cytoplasm | other |
| MIMAT0003948 | miR-4712-5p (and other miRNAs w/seed CCAGUAC) | -15.630 | TargetScan Human | Moderate (predicted) | MTMR1 | myotubularin related protein 1 | Cytoplasm | phosphatase |
| MIMAT0003948 | miR-4712-5p (and other miRNAs w/seed CCAGUAC) | -15.630 | TargetScan Human | High (predicted) | MTPN | myotrophin | Nucleus | transcription regulator |
| MIMAT0003948 | miR-4712-5p (and other miRNAs w/seed CCAGUAC) | -15.630 | TargetScan Human | Moderate (predicted) | MTX3 | metaxin 3 | Other | other |
| MIMAT0003948 | miR-4712-5p (and other miRNAs w/seed CCAGUAC) | -15.630 | TargetScan Human | Moderate (predicted) | MX1 | MX dynamin like GTPase 1 | Cytoplasm | enzyme |
| MIMAT0003948 | miR-4712-5p (and other miRNAs w/seed CCAGUAC) | -15.630 | TargetScan Human | Moderate (predicted) | MYBL1 | MYB proto-oncogene like 1 | Nucleus | transcription regulator |
| MIMAT0003948 | miR-4712-5p (and other miRNAs w/seed CCAGUAC) | -15.630 | TargetScan Human | Moderate (predicted) | MYOM3 | myomesin 3 | Other | other |
| MIMAT0003948 | miR-4712-5p (and other miRNAs w/seed CCAGUAC) | -15.630 | TargetScan Human | Moderate (predicted) | NAMPT | nicotinamide phosphoribosyltransferase | Extracellular Space | cytokine |
| MIMAT0003948 | miR-4712-5p (and other miRNAs w/seed CCAGUAC) | -15.630 | TargetScan Human | Moderate (predicted) | NCAM2 | neural cell adhesion molecule 2 | Plasma Membrane | other |
| MIMAT0003948 | miR-4712-5p (and other miRNAs w/seed CCAGUAC) | -15.630 | TargetScan Human | Moderate (predicted) | NECAB1 | -terminal EF-hand calcium binding protein | Cytoplasm | other |
| MIMAT0003948 | miR-4712-5p (and other miRNAs w/seed CCAGUAC) | -15.630 | TargetScan Human | Moderate (predicted) | NEU3 | neuraminidase 3 | Plasma Membrane | enzyme |
| MIMAT0003948 | miR-4712-5p (and other miRNAs w/seed CCAGUAC) | -15.630 | TargetScan Human | High (predicted) | NKAIN4 | m/potassium transporting ATPase interac | Other | other |
| MIMAT0003948 | miR-4712-5p (and other miRNAs w/seed CCAGUAC) | -15.630 | TargetScan Human | Moderate (predicted) | NMT2 | N-myristoyltransferase 2 | Cytoplasm | enzyme |
| MIMAT0003948 | miR-4712-5p (and other miRNAs w/seed CCAGUAC) | -15.630 | TargetScan Human | Moderate (predicted) | NPAT | lear protein, coactivator of histone transcri | Nucleus | transcription regulator |
| MIMAT0003948 | miR-4712-5p (and other miRNAs w/seed CCAGUAC) | -15.630 | TargetScan Human | Moderate (predicted) | NPPC | natriuretic peptide C | Extracellular Space | other |
| MIMAT0003948 | miR-4712-5p (and other miRNAs w/seed CCAGUAC) | -15.630 | TargetScan Human | High (predicted) | NRP1 | neuropilin 1 | Plasma Membrane | transmembrane receptor |
| MIMAT0003948 | miR-4712-5p (and other miRNAs w/seed CCAGUAC) | -15.630 | TargetScan Human | High (predicted) | NUDCD1 | NudC domain containing 1 | Nucleus | other |
| MIMAT0003948 | miR-4712-5p (and other miRNAs w/seed CCAGUAC) | -15.630 | TargetScan Human | Moderate (predicted) | NUP153 | nucleoporin 153 | Nucleus | other |
| MIMAT0003948 | miR-4712-5p (and other miRNAs w/seed CCAGUAC) | -15.630 | TargetScan Human | Moderate (predicted) | NXPH1 | neurexophilin 1 | Extracellular Space | other |
| MIMAT0003948 | miR-4712-5p (and other miRNAs w/seed CCAGUAC) | -15.630 | TargetScan Human | Moderate (predicted) | NXT2 | nuclear transport factor 2 like export factor | Nucleus | transporter |
| MIMAT0003948 | miR-4712-5p (and other miRNAs w/seed CCAGUAC) | -15.630 | TargetScan Human | Moderate (predicted) | OAF | out at first homolog | Cytoplasm | other |
| MIMAT0003948 | miR-4712-5p (and other miRNAs w/seed CCAGUAC) | -15.630 | TargetScan Human | Moderate (predicted) | OGT | ked N-acetylglucosamine (GlcNAc) transf | Cytoplasm | enzyme |
| MIMAT0003948 | miR-4712-5p (and other miRNAs w/seed CCAGUAC) | -15.630 | TargetScan Human | Moderate (predicted) | OR10A2 | ctory receptor family 10 subfamily A memb | Plasma Membrane | G-protein coupled receptor |
| MIMAT0003948 | miR-4712-5p (and other miRNAs w/seed CCAGUAC) | -15.630 | TargetScan Human | Moderate (predicted) | OSGIN2 | e stress induced growth inhibitor family m | Other | other |
| MIMAT0003948 | miR-4712-5p (and other miRNAs w/seed CCAGUAC) | -15.630 | TargetScan Human | Moderate (predicted) | P2RY1 | purinergic receptor P2Y1 | Plasma Membrane | G-protein coupled receptor |
| MIMAT0003948 | miR-4712-5p (and other miRNAs w/seed CCAGUAC) | -15.630 | TargetScan Human | Moderate (predicted) | PAAF1 | proteasomal ATPase associated factor 1 | Other | other |
| MIMAT0003948 | miR-4712-5p (and other miRNAs w/seed CCAGUAC) | -15.630 | TargetScan Human | Moderate (predicted) | PACRG-AS3 | PACRG antisense RNA 3 | Other | other |
| MIMAT0003948 | miR-4712-5p (and other miRNAs w/seed CCAGUAC) | -15.630 | TargetScan Human | Moderate (predicted) | PAN2 | poly(A) specific ribonuclease subunit PAN | Cytoplasm | peptidase |
| MIMAT0003948 | miR-4712-5p (and other miRNAs w/seed CCAGUAC) | -15.630 | TargetScan Human | Moderate (predicted) | PAQR8 | ogestin and adipoQ receptor family membe | Plasma Membrane | other |
| MIMAT0003948 | miR-4712-5p (and other miRNAs w/seed CCAGUAC) | -15.630 | TargetScan Human | Moderate (predicted) | PBK | PDZ binding kinase | Cytoplasm | kinase |
| MIMAT0003948 | miR-4712-5p (and other miRNAs w/seed CCAGUAC) | -15.630 | TargetScan Human | High (predicted) | PCDHB3 | protocadherin beta 3 | Plasma Membrane | other |
| MIMAT0003948 | miR-4712-5p (and other miRNAs w/seed CCAGUAC) | -15.630 | TargetScan Human | Moderate (predicted) | PCSK5 | proprotein convertase subtilisin/kexin type | Extracellular Space | peptidase |
| MIMAT0003948 | miR-4712-5p (and other miRNAs w/seed CCAGUAC) | -15.630 | TargetScan Human | Moderate (predicted) | PDCD1LG2 | programmed cell death 1 ligand 2 | Plasma Membrane | enzyme |
| MIMAT0003948 | miR-4712-5p (and other miRNAs w/seed CCAGUAC) | -15.630 | TargetScan Human | Moderate (predicted) | PDIK1L | PDLIM1 interacting kinase 1 like | Nucleus | kinase |
| MIMAT0003948 | miR-4712-5p (and other miRNAs w/seed CCAGUAC) | -15.630 | TargetScan Human | Moderate (predicted) | PGF | placental growth factor | Extracellular Space | growth factor |
| MIMAT0003948 | miR-4712-5p (and other miRNAs w/seed CCAGUAC) | -15.630 | TargetScan Human | Moderate (predicted) | PHACTR2 | phosphatase and actin regulator 2 | Other | other |
| MIMAT0003948 | miR-4712-5p (and other miRNAs w/seed CCAGUAC) | -15.630 | TargetScan Human | Moderate (predicted) | PHF20 | PHD finger protein 20 | Nucleus | transcription regulator |
| MIMAT0003948 | miR-4712-5p (and other miRNAs w/seed CCAGUAC) | -15.630 | TargetScan Human | Moderate (predicted) | PHTF1 | putative homeodomain transcription factor | Nucleus | transcription regulator |
| MIMAT0003948 | miR-4712-5p (and other miRNAs w/seed CCAGUAC) | -15.630 | TargetScan Human | Moderate (predicted) | PIGH | hatidylinositol glycan anchor biosynthesis | Cytoplasm | enzyme |
| MIMAT0003948 | miR-4712-5p (and other miRNAs w/seed CCAGUAC) | -15.630 | TargetScan Human | Moderate (predicted) | PIGO | hatidylinositol glycan anchor biosynthesis | Cytoplasm | enzyme |
| MIMAT0003948 | miR-4712-5p (and other miRNAs w/seed CCAGUAC) | -15.630 | TargetScan Human | Moderate (predicted) | PIP4K2A | hatidylinositol-5-phosphate 4-kinase type | Cytoplasm | kinase |
| MIMAT0003948 | miR-4712-5p (and other miRNAs w/seed CCAGUAC) | -15.630 | TargetScan Human | Moderate (predicted) | PITRM1 | pitrilysin metallopeptidase 1 | Cytoplasm | peptidase |
| MIMAT0003948 | miR-4712-5p (and other miRNAs w/seed CCAGUAC) | -15.630 | TargetScan Human | Moderate (predicted) | PKD1L2 | polycystin 1 like 2 (gene/pseudogene) | Other | ion channel |
| MIMAT0003948 | miR-4712-5p (and other miRNAs w/seed CCAGUAC) | -15.630 | TargetScan Human | Moderate (predicted) | PKD2 | stin 2, transient receptor potential cation c | Plasma Membrane | ion channel |
| MIMAT0003948 | miR-4712-5p (and other miRNAs w/seed CCAGUAC) | -15.630 | TargetScan Human | Moderate (predicted) | PLEKHA5 | pleckstrin homology domain containing A5 | Cytoplasm | other |
| MIMAT0003948 | miR-4712-5p (and other miRNAs w/seed CCAGUAC) | -15.630 | TargetScan Human | Moderate (predicted) | PLIN3 | perilipin 3 | Cytoplasm | other |
| MIMAT0003948 | miR-4712-5p (and other miRNAs w/seed CCAGUAC) | -15.630 | TargetScan Human | Moderate (predicted) | PLXDC2 | plexin domain containing 2 | Extracellular Space | other |
| MIMAT0003948 | miR-4712-5p (and other miRNAs w/seed CCAGUAC) | -15.630 | TargetScan Human | Moderate (predicted) | POGLUT3 | protein O-glucosyltransferase 3 | Other | enzyme |
| MIMAT0003948 | miR-4712-5p (and other miRNAs w/seed CCAGUAC) | -15.630 | TargetScan Human | Moderate (predicted) | POGZ | transposable element derived with ZNF d | Nucleus | enzyme |
| MIMAT0003948 | miR-4712-5p (and other miRNAs w/seed CCAGUAC) | -15.630 | TargetScan Human | High (predicted) | POMT2 | protein O-mannosyltransferase 2 | Cytoplasm | enzyme |
| MIMAT0003948 | miR-4712-5p (and other miRNAs w/seed CCAGUAC) | -15.630 | TargetScan Human | High (predicted) | POP7 | OP7 homolog, ribonuclease P/MRP subu | Nucleus | enzyme |
| MIMAT0003948 | miR-4712-5p (and other miRNAs w/seed CCAGUAC) | -15.630 | TargetScan Human | Moderate (predicted) | PPP1CC | otein phosphatase 1 catalytic subunit gam | Nucleus | phosphatase |
| MIMAT0003948 | miR-4712-5p (and other miRNAs w/seed CCAGUAC) | -15.630 | TargetScan Human | Moderate (predicted) | PPP3CA | rotein phosphatase 3 catalytic subunit alp | Cytoplasm | phosphatase |
| MIMAT0003948 | miR-4712-5p (and other miRNAs w/seed CCAGUAC) | -15.630 | TargetScan Human | Moderate (predicted) | PPP4R2 | protein phosphatase 4 regulatory subunit | Cytoplasm | other |
| MIMAT0003948 | miR-4712-5p (and other miRNAs w/seed CCAGUAC) | -15.630 | TargetScan Human | Moderate (predicted) | PRIMA1 | proline rich membrane anchor 1 | Plasma Membrane | other |
| MIMAT0003948 | miR-4712-5p (and other miRNAs w/seed CCAGUAC) | -15.630 | TargetScan Human | Moderate (predicted) | PRR13 | proline rich 13 | Nucleus | other |
| MIMAT0003948 | miR-4712-5p (and other miRNAs w/seed CCAGUAC) | -15.630 | TargetScan Human | Moderate (predicted) | PSENEN | esenilin enhancer, gamma-secretase subu | Plasma Membrane | peptidase |
| MIMAT0003948 | miR-4712-5p (and other miRNAs w/seed CCAGUAC) | -15.630 | TargetScan Human | Moderate (predicted) | PSG5 | pregnancy specific beta-1-glycoprotein 5 | Extracellular Space | other |
| MIMAT0003948 | miR-4712-5p (and other miRNAs w/seed CCAGUAC) | -15.630 | TargetScan Human | Moderate (predicted) | PSMD9 | proteasome 26S subunit, non-ATPase 9 | Cytoplasm | transcription regulator |
| MIMAT0003948 | miR-4712-5p (and other miRNAs w/seed CCAGUAC) | -15.630 | TargetScan Human | Moderate (predicted) | PSMG2 | proteasome assembly chaperone 2 | Nucleus | other |
| MIMAT0003948 | miR-4712-5p (and other miRNAs w/seed CCAGUAC) | -15.630 | TargetScan Human | Moderate (predicted) | PTGES3 | prostaglandin E synthase 3 | Cytoplasm | enzyme |
| MIMAT0003948 | miR-4712-5p (and other miRNAs w/seed CCAGUAC) | -15.630 | TargetScan Human | Moderate (predicted) | PTPN1 | tein tyrosine phosphatase non-receptor ty | Cytoplasm | phosphatase |
| MIMAT0003948 | miR-4712-5p (and other miRNAs w/seed CCAGUAC) | -15.630 | TargetScan Human | Moderate (predicted) | PTX3 | pentraxin 3 | Extracellular Space | other |
| MIMAT0003948 | miR-4712-5p (and other miRNAs w/seed CCAGUAC) | -15.630 | TargetScan Human | Moderate (predicted) | RAB23 | RAB23, member RAS oncogene family | Cytoplasm | enzyme |
| MIMAT0003948 | miR-4712-5p (and other miRNAs w/seed CCAGUAC) | -15.630 | TargetScan Human | Moderate (predicted) | RAB27B | RAB27B, member RAS oncogene family | Cytoplasm | enzyme |
| MIMAT0003948 | miR-4712-5p (and other miRNAs w/seed CCAGUAC) | -15.630 | TargetScan Human | Moderate (predicted) | RAB8B | RAB8B, member RAS oncogene family | Cytoplasm | enzyme |
| MIMAT0003948 | miR-4712-5p (and other miRNAs w/seed CCAGUAC) | -15.630 | TargetScan Human | High (predicted) | RAC2 | Rac family small GTPase 2 | Cytoplasm | enzyme |
| MIMAT0003948 | miR-4712-5p (and other miRNAs w/seed CCAGUAC) | -15.630 | TargetScan Human | Moderate (predicted) | RANBP9 | RAN binding protein 9 | Nucleus | other |
| MIMAT0003948 | miR-4712-5p (and other miRNAs w/seed CCAGUAC) | -15.630 | TargetScan Human | Moderate (predicted) | RAP1GDS1 | ap1 GTPase-GDP dissociation stimulator | Cytoplasm | other |
| MIMAT0003948 | miR-4712-5p (and other miRNAs w/seed CCAGUAC) | -15.630 | TargetScan Human | Moderate (predicted) | RASSF3 | Ras association domain family member 3 | Other | other |
| MIMAT0003948 | miR-4712-5p (and other miRNAs w/seed CCAGUAC) | -15.630 | TargetScan Human | Moderate (predicted) | RBM25 | RNA binding motif protein 25 | Nucleus | other |
| MIMAT0003948 | miR-4712-5p (and other miRNAs w/seed CCAGUAC) | -15.630 | TargetScan Human | High (predicted) | RCAN2 | regulator of calcineurin 2 | Other | other |
| MIMAT0003948 | miR-4712-5p (and other miRNAs w/seed CCAGUAC) | -15.630 | TargetScan Human | High (predicted) | RCHY1 | finger and CHY zinc finger domain contain | Nucleus | enzyme |
| MIMAT0003948 | miR-4712-5p (and other miRNAs w/seed CCAGUAC) | -15.630 | TargetScan Human | Moderate (predicted) | RCN2 | reticulocalbin 2 | Cytoplasm | other |
| MIMAT0003948 | miR-4712-5p (and other miRNAs w/seed CCAGUAC) | -15.630 | TargetScan Human | Moderate (predicted) | RCOR2 | REST corepressor 2 | Nucleus | transcription regulator |
| MIMAT0003948 | miR-4712-5p (and other miRNAs w/seed CCAGUAC) | -15.630 | TargetScan Human | Moderate (predicted) | RMI1 | RecQ mediated genome instability 1 | Nucleus | other |
| MIMAT0003948 | miR-4712-5p (and other miRNAs w/seed CCAGUAC) | -15.630 | TargetScan Human | Moderate (predicted) | RNF122 | ring finger protein 122 | Cytoplasm | enzyme |
| MIMAT0003948 | miR-4712-5p (and other miRNAs w/seed CCAGUAC) | -15.630 | TargetScan Human | Moderate (predicted) | RNF125 | ring finger protein 125 | Cytoplasm | enzyme |
| MIMAT0003948 | miR-4712-5p (and other miRNAs w/seed CCAGUAC) | -15.630 | TargetScan Human | Moderate (predicted) | RNF166 | ring finger protein 166 | Other | other |
| MIMAT0003948 | miR-4712-5p (and other miRNAs w/seed CCAGUAC) | -15.630 | TargetScan Human | Moderate (predicted) | RNF2 | ring finger protein 2 | Nucleus | transcription regulator |
| MIMAT0003948 | miR-4712-5p (and other miRNAs w/seed CCAGUAC) | -15.630 | TargetScan Human | Moderate (predicted) | RNLS | renalase, FAD dependent amine oxidase | Extracellular Space | enzyme |
| MIMAT0003948 | miR-4712-5p (and other miRNAs w/seed CCAGUAC) | -15.630 | TargetScan Human | Moderate (predicted) | RPAP1 | RNA polymerase II associated protein 1 | Other | other |
| MIMAT0003948 | miR-4712-5p (and other miRNAs w/seed CCAGUAC) | -15.630 | TargetScan Human | Moderate (predicted) | RPL21 | ribosomal protein L21 | Cytoplasm | other |
| MIMAT0003948 | miR-4712-5p (and other miRNAs w/seed CCAGUAC) | -15.630 | TargetScan Human | Moderate (predicted) | RPL36 | ribosomal protein L36 | Cytoplasm | other |
| MIMAT0003948 | miR-4712-5p (and other miRNAs w/seed CCAGUAC) | -15.630 | TargetScan Human | Moderate (predicted) | RRAGB | Ras related GTP binding B | Cytoplasm | enzyme |
| MIMAT0003948 | miR-4712-5p (and other miRNAs w/seed CCAGUAC) | -15.630 | TargetScan Human | High (predicted) | RSAD1 | cal S-adenosyl methionine domain contain | Cytoplasm | other |
| MIMAT0003948 | miR-4712-5p (and other miRNAs w/seed CCAGUAC) | -15.630 | TargetScan Human | Moderate (predicted) | RXFP1 | relaxin family peptide receptor 1 | Plasma Membrane | G-protein coupled receptor |
| MIMAT0003948 | miR-4712-5p (and other miRNAs w/seed CCAGUAC) | -15.630 | TargetScan Human | Moderate (predicted) | RYBP | RING1 and YY1 binding protein | Nucleus | transcription regulator |
| MIMAT0003948 | miR-4712-5p (and other miRNAs w/seed CCAGUAC) | -15.630 | TargetScan Human | High (predicted) | RYR1 | ryanodine receptor 1 | Cytoplasm | ion channel |
| MIMAT0003948 | miR-4712-5p (and other miRNAs w/seed CCAGUAC) | -15.630 | TargetScan Human | High (predicted) | S100A7A | S100 calcium binding protein A7A | Cytoplasm | other |
| MIMAT0003948 | miR-4712-5p (and other miRNAs w/seed CCAGUAC) | -15.630 | TargetScan Human | Moderate (predicted) | S1PR3 | sphingosine-1-phosphate receptor 3 | Plasma Membrane | G-protein coupled receptor |
| MIMAT0003948 | miR-4712-5p (and other miRNAs w/seed CCAGUAC) | -15.630 | TargetScan Human | High (predicted) | SAMD3 | sterile alpha motif domain containing 3 | Other | other |
| MIMAT0003948 | miR-4712-5p (and other miRNAs w/seed CCAGUAC) | -15.630 | TargetScan Human | High (predicted) | SAMD7 | sterile alpha motif domain containing 7 | Other | other |
| MIMAT0003948 | miR-4712-5p (and other miRNAs w/seed CCAGUAC) | -15.630 | TargetScan Human | High (predicted) | SBSN | suprabasin | Cytoplasm | other |
| MIMAT0003948 | miR-4712-5p (and other miRNAs w/seed CCAGUAC) | -15.630 | TargetScan Human | High (predicted) | SCG2 | secretogranin II | Extracellular Space | cytokine |
| MIMAT0003948 | miR-4712-5p (and other miRNAs w/seed CCAGUAC) | -15.630 | TargetScan Human | Moderate (predicted) | SCP2 | sterol carrier protein 2 | Cytoplasm | transporter |
| MIMAT0003948 | miR-4712-5p (and other miRNAs w/seed CCAGUAC) | -15.630 | TargetScan Human | Moderate (predicted) | SEC11A | 11 homolog A, signal peptidase complex s | Cytoplasm | peptidase |
| MIMAT0003948 | miR-4712-5p (and other miRNAs w/seed CCAGUAC) | -15.630 | TargetScan Human | Moderate (predicted) | SELENOH | selenoprotein H | Nucleus | other |
| MIMAT0003948 | miR-4712-5p (and other miRNAs w/seed CCAGUAC) | -15.630 | TargetScan Human | Moderate (predicted) | SEPHS2 | selenophosphate synthetase 2 | Cytoplasm | enzyme |
| MIMAT0003948 | miR-4712-5p (and other miRNAs w/seed CCAGUAC) | -15.630 | TargetScan Human | Moderate (predicted) | SERF2 | small EDRK-rich factor 2 | Other | other |
| MIMAT0003948 | miR-4712-5p (and other miRNAs w/seed CCAGUAC) | -15.630 | TargetScan Human | High (predicted) | SERPINB10 | serpin family B member 10 | Cytoplasm | other |
| MIMAT0003948 | miR-4712-5p (and other miRNAs w/seed CCAGUAC) | -15.630 | TargetScan Human | Moderate (predicted) | SFXN2 | sideroflexin 2 | Cytoplasm | transporter |
| MIMAT0003948 | miR-4712-5p (and other miRNAs w/seed CCAGUAC) | -15.630 | TargetScan Human | Moderate (predicted) | SH2D1A | SH2 domain containing 1A | Cytoplasm | other |
| MIMAT0003948 | miR-4712-5p (and other miRNAs w/seed CCAGUAC) | -15.630 | TargetScan Human | Moderate (predicted) | SH2D5 | SH2 domain containing 5 | Plasma Membrane | other |
| MIMAT0003948 | miR-4712-5p (and other miRNAs w/seed CCAGUAC) | -15.630 | TargetScan Human | Moderate (predicted) | SH3RF2 | SH3 domain containing ring finger 2 | Extracellular Space | enzyme |
| MIMAT0003948 | miR-4712-5p (and other miRNAs w/seed CCAGUAC) | -15.630 | TargetScan Human | Moderate (predicted) | SHISAL2B | shisa like 2B | Other | other |
| MIMAT0003948 | miR-4712-5p (and other miRNAs w/seed CCAGUAC) | -15.630 | TargetScan Human | Moderate (predicted) | SIM1 | SIM bHLH transcription factor 1 | Nucleus | transcription regulator |
| MIMAT0003948 | miR-4712-5p (and other miRNAs w/seed CCAGUAC) | -15.630 | TargetScan Human | High (predicted) | SIRT7 | sirtuin 7 | Nucleus | enzyme |
| MIMAT0003948 | miR-4712-5p (and other miRNAs w/seed CCAGUAC) | -15.630 | TargetScan Human | Moderate (predicted) | SLC12A8 | solute carrier family 12 member 8 | Other | transporter |
| MIMAT0003948 | miR-4712-5p (and other miRNAs w/seed CCAGUAC) | -15.630 | TargetScan Human | Moderate (predicted) | SLC17A6 | solute carrier family 17 member 6 | Plasma Membrane | transporter |
| MIMAT0003948 | miR-4712-5p (and other miRNAs w/seed CCAGUAC) | -15.630 | TargetScan Human | Moderate (predicted) | SLC25A24 | solute carrier family 25 member 24 | Cytoplasm | transporter |
| MIMAT0003948 | miR-4712-5p (and other miRNAs w/seed CCAGUAC) | -15.630 | TargetScan Human | Moderate (predicted) | SLC25A6 | solute carrier family 25 member 6 | Cytoplasm | transporter |
| MIMAT0003948 | miR-4712-5p (and other miRNAs w/seed CCAGUAC) | -15.630 | TargetScan Human | Moderate (predicted) | SLC27A3 | solute carrier family 27 member 3 | Cytoplasm | transporter |
| MIMAT0003948 | miR-4712-5p (and other miRNAs w/seed CCAGUAC) | -15.630 | TargetScan Human | Moderate (predicted) | SLC30A9 | solute carrier family 30 member 9 | Nucleus | transporter |

Sup. Table 3. List of 3699 targets of the seven dysregulated miRNAs obtained from three different target prediction databases,TargetScan TarBase and miRecords considering those predicted as moderate, high, or experimentally validated, respectively.

| | | | | | | | | |
|---|---|---|---|---|---|---|---|---|
| MIMAT0003948 | miR-4712-5p (and other miRNAs w/seed CCAGUAC) | -15.630 | TargetScan Human | Moderate (predicted) | SLC35D1 | solute carrier family 35 member D1 | Cytoplasm | transporter |
| MIMAT0003948 | miR-4712-5p (and other miRNAs w/seed CCAGUAC) | -15.630 | TargetScan Human | Moderate (predicted) | SLC39A14 | solute carrier family 39 member 14 | Plasma Membrane | transporter |
| MIMAT0003948 | miR-4712-5p (and other miRNAs w/seed CCAGUAC) | -15.630 | TargetScan Human | Moderate (predicted) | SLC46A3 | solute carrier family 46 member 3 | Extracellular Space | transporter |
| MIMAT0003948 | miR-4712-5p (and other miRNAs w/seed CCAGUAC) | -15.630 | TargetScan Human | Moderate (predicted) | SLC5A3 | solute carrier family 5 member 3 | Plasma Membrane | transporter |
| MIMAT0003948 | miR-4712-5p (and other miRNAs w/seed CCAGUAC) | -15.630 | TargetScan Human | Moderate (predicted) | SMN1/SMN2 | survival of motor neuron 1, telomeric | Nucleus | other |
| MIMAT0003948 | miR-4712-5p (and other miRNAs w/seed CCAGUAC) | -15.630 | TargetScan Human | Moderate (predicted) | SNW1 | SNW domain containing 1 | Nucleus | transcription regulator |
| MIMAT0003948 | miR-4712-5p (and other miRNAs w/seed CCAGUAC) | -15.630 | TargetScan Human | Moderate (predicted) | SOX2 | SRY-box transcription factor 2 | Nucleus | transcription regulator |
| MIMAT0003948 | miR-4712-5p (and other miRNAs w/seed CCAGUAC) | -15.630 | TargetScan Human | Moderate (predicted) | SP4 | Sp4 transcription factor | Nucleus | transcription regulator |
| MIMAT0003948 | miR-4712-5p (and other miRNAs w/seed CCAGUAC) | -15.630 | TargetScan Human | Moderate (predicted) | SPATA5 | spermatogenesis associated 5 | Cytoplasm | other |
| MIMAT0003948 | miR-4712-5p (and other miRNAs w/seed CCAGUAC) | -15.630 | TargetScan Human | Moderate (predicted) | SPDYA | dy/RINGO cell cycle regulator family mem | Nucleus | other |
| MIMAT0003948 | miR-4712-5p (and other miRNAs w/seed CCAGUAC) | -15.630 | TargetScan Human | Moderate (predicted) | SPHK2 | sphingosine kinase 2 | Cytoplasm | kinase |
| MIMAT0003948 | miR-4712-5p (and other miRNAs w/seed CCAGUAC) | -15.630 | TargetScan Human | Moderate (predicted) | SPIN4 | spindlin family member 4 | Other | other |
| MIMAT0003948 | miR-4712-5p (and other miRNAs w/seed CCAGUAC) | -15.630 | TargetScan Human | Moderate (predicted) | SRSF10 | serine and arginine rich splicing factor 10 | Nucleus | other |
| MIMAT0003948 | miR-4712-5p (and other miRNAs w/seed CCAGUAC) | -15.630 | TargetScan Human | Moderate (predicted) | STAC2 | SH3 and cysteine rich domain 2 | Plasma Membrane | other |
| MIMAT0003948 | miR-4712-5p (and other miRNAs w/seed CCAGUAC) | -15.630 | TargetScan Human | High (predicted) | STC1 | stanniocalcin 1 | Extracellular Space | kinase |
| MIMAT0003948 | miR-4712-5p (and other miRNAs w/seed CCAGUAC) | -15.630 | TargetScan Human | High (predicted) | STK17A | serine/threonine kinase 17a | Nucleus | kinase |
| MIMAT0003948 | miR-4712-5p (and other miRNAs w/seed CCAGUAC) | -15.630 | TargetScan Human | High (predicted) | STK38L | serine/threonine kinase 38 like | Cytoplasm | kinase |
| MIMAT0003948 | miR-4712-5p (and other miRNAs w/seed CCAGUAC) | -15.630 | TargetScan Human | High (predicted) | STMN1 | stathmin 1 | Cytoplasm | other |
| MIMAT0003948 | miR-4712-5p (and other miRNAs w/seed CCAGUAC) | -15.630 | TargetScan Human | Moderate (predicted) | STRN3 | striatin 3 | Nucleus | transcription regulator |
| MIMAT0003948 | miR-4712-5p (and other miRNAs w/seed CCAGUAC) | -15.630 | TargetScan Human | High (predicted) | SUCNR1 | succinate receptor 1 | Plasma Membrane | G-protein coupled receptor |
| MIMAT0003948 | miR-4712-5p (and other miRNAs w/seed CCAGUAC) | -15.630 | TargetScan Human | Moderate (predicted) | SYT14 | synaptotagmin 14 | Other | transporter |
| MIMAT0003948 | miR-4712-5p (and other miRNAs w/seed CCAGUAC) | -15.630 | TargetScan Human | Moderate (predicted) | TAF7 | ATA-box binding protein associated factor | Nucleus | transcription regulator |
| MIMAT0003948 | miR-4712-5p (and other miRNAs w/seed CCAGUAC) | -15.630 | TargetScan Human | Moderate (predicted) | TBC1D13 | TBC1 domain family member 13 | Cytoplasm | other |
| MIMAT0003948 | miR-4712-5p (and other miRNAs w/seed CCAGUAC) | -15.630 | TargetScan Human | High (predicted) | TBCA | tubulin folding cofactor A | Cytoplasm | other |
| MIMAT0003948 | miR-4712-5p (and other miRNAs w/seed CCAGUAC) | -15.630 | TargetScan Human | Moderate (predicted) | TCAP | titin-cap | Cytoplasm | other |
| MIMAT0003948 | miR-4712-5p (and other miRNAs w/seed CCAGUAC) | -15.630 | TargetScan Human | High (predicted) | TCL1B | TCL1 family AKT coactivator B | Other | other |
| MIMAT0003948 | miR-4712-5p (and other miRNAs w/seed CCAGUAC) | -15.630 | TargetScan Human | High (predicted) | TDP1 | tyrosyl-DNA phosphodiesterase 1 | Nucleus | enzyme |
| MIMAT0003948 | miR-4712-5p (and other miRNAs w/seed CCAGUAC) | -15.630 | TargetScan Human | Moderate (predicted) | TERB2 | nere repeat binding bouquet formation pro | Nucleus | other |
| MIMAT0003948 | miR-4712-5p (and other miRNAs w/seed CCAGUAC) | -15.630 | TargetScan Human | Moderate (predicted) | TEX35 | testis expressed 35 | Nucleus | other |
| MIMAT0003948 | miR-4712-5p (and other miRNAs w/seed CCAGUAC) | -15.630 | TargetScan Human | Moderate (predicted) | TFAP2C | transcription factor AP-2 gamma | Nucleus | transcription regulator |
| MIMAT0003948 | miR-4712-5p (and other miRNAs w/seed CCAGUAC) | -15.630 | TargetScan Human | Moderate (predicted) | TFRC | transferrin receptor | Plasma Membrane | transporter |
| MIMAT0003948 | miR-4712-5p (and other miRNAs w/seed CCAGUAC) | -15.630 | TargetScan Human | Moderate (predicted) | TGDS | TDP-glucose 4,6-dehydratase | Extracellular Space | enzyme |
| MIMAT0003948 | miR-4712-5p (and other miRNAs w/seed CCAGUAC) | -15.630 | TargetScan Human | Moderate (predicted) | THAP4 | THAP domain containing 4 | Nucleus | transcription regulator |
| MIMAT0003948 | miR-4712-5p (and other miRNAs w/seed CCAGUAC) | -15.630 | TargetScan Human | Moderate (predicted) | TIFA | nteracting protein with forkhead associated | Cytoplasm | other |
| MIMAT0003948 | miR-4712-5p (and other miRNAs w/seed CCAGUAC) | -15.630 | TargetScan Human | High (predicted) | TLNRD1 | talin rod domain containing 1 | Cytoplasm | other |
| MIMAT0003948 | miR-4712-5p (and other miRNAs w/seed CCAGUAC) | -15.630 | TargetScan Human | Moderate (predicted) | TLR7 | toll like receptor 7 | Plasma Membrane | transmembrane receptor |
| MIMAT0003948 | miR-4712-5p (and other miRNAs w/seed CCAGUAC) | -15.630 | TargetScan Human | Moderate (predicted) | TMCC2 | nsmembrane and coiled-coil domain famil | Extracellular Space | other |
| MIMAT0003948 | miR-4712-5p (and other miRNAs w/seed CCAGUAC) | -15.630 | TargetScan Human | Moderate (predicted) | TMEM132C | transmembrane protein 132C | Other | other |
| MIMAT0003948 | miR-4712-5p (and other miRNAs w/seed CCAGUAC) | -15.630 | TargetScan Human | High (predicted) | TMEM178A | transmembrane protein 178A | Cytoplasm | other |
| MIMAT0003948 | miR-4712-5p (and other miRNAs w/seed CCAGUAC) | -15.630 | TargetScan Human | Moderate (predicted) | TMEM212 | transmembrane protein 212 | Other | other |
| MIMAT0003948 | miR-4712-5p (and other miRNAs w/seed CCAGUAC) | -15.630 | TargetScan Human | Moderate (predicted) | TMEM74B | transmembrane protein 74B | Other | other |
| MIMAT0003948 | miR-4712-5p (and other miRNAs w/seed CCAGUAC) | -15.630 | TargetScan Human | High (predicted) | TNFRSF10B | TNF receptor superfamily member 10b | Plasma Membrane | transmembrane receptor |
| MIMAT0003948 | miR-4712-5p (and other miRNAs w/seed CCAGUAC) | -15.630 | TargetScan Human | High (predicted) | TNN | tenascin N | Plasma Membrane | other |
| MIMAT0003948 | miR-4712-5p (and other miRNAs w/seed CCAGUAC) | -15.630 | TargetScan Human | Moderate (predicted) | TOM1 | arget of myb1 membrane trafficking protei | Cytoplasm | transporter |
| MIMAT0003948 | miR-4712-5p (and other miRNAs w/seed CCAGUAC) | -15.630 | TargetScan Human | Moderate (predicted) | TOP1MT | DNA topoisomerase I mitochondrial | Cytoplasm | enzyme |
| MIMAT0003948 | miR-4712-5p (and other miRNAs w/seed CCAGUAC) | -15.630 | TargetScan Human | Moderate (predicted) | TRIAP1 | TP53 regulated inhibitor of apoptosis 1 | Cytoplasm | other |
| MIMAT0003948 | miR-4712-5p (and other miRNAs w/seed CCAGUAC) | -15.630 | TargetScan Human | Moderate (predicted) | TRIM49D1/TRIM49D2 | tripartite motif containing 49D1 | Other | other |
| MIMAT0003948 | miR-4712-5p (and other miRNAs w/seed CCAGUAC) | -15.630 | TargetScan Human | Moderate (predicted) | TRIM5 | tripartite motif containing 5 | Cytoplasm | enzyme |
| MIMAT0003948 | miR-4712-5p (and other miRNAs w/seed CCAGUAC) | -15.630 | TargetScan Human | High (predicted) | TRPS1 | transcriptional repressor GATA binding 1 | Nucleus | transcription regulator |
| MIMAT0003948 | miR-4712-5p (and other miRNAs w/seed CCAGUAC) | -15.630 | TargetScan Human | Moderate (predicted) | TSPYL5 | TSPY like 5 | Other | other |
| MIMAT0003948 | miR-4712-5p (and other miRNAs w/seed CCAGUAC) | -15.630 | TargetScan Human | Moderate (predicted) | TTL | tubulin tyrosine ligase | Cytoplasm | enzyme |
| MIMAT0003948 | miR-4712-5p (and other miRNAs w/seed CCAGUAC) | -15.630 | TargetScan Human | Moderate (predicted) | TXNDC12 | thioredoxin domain containing 12 | Cytoplasm | enzyme |
| MIMAT0003948 | miR-4712-5p (and other miRNAs w/seed CCAGUAC) | -15.630 | TargetScan Human | Moderate (predicted) | UNC13B | unc-13 homolog B | Cytoplasm | other |
| MIMAT0003948 | miR-4712-5p (and other miRNAs w/seed CCAGUAC) | -15.630 | TargetScan Human | Moderate (predicted) | VAMP5 | vesicle associated membrane protein 5 | Plasma Membrane | transporter |
| MIMAT0003948 | miR-4712-5p (and other miRNAs w/seed CCAGUAC) | -15.630 | TargetScan Human | Moderate (predicted) | VASH2 | vasohibin 2 | Cytoplasm | peptidase |
| MIMAT0003948 | miR-4712-5p (and other miRNAs w/seed CCAGUAC) | -15.630 | TargetScan Human | Moderate (predicted) | VAT1 | vesicle amine transport 1 | Plasma Membrane | transporter |
| MIMAT0003948 | miR-4712-5p (and other miRNAs w/seed CCAGUAC) | -15.630 | TargetScan Human | Moderate (predicted) | VCAM1 | vascular cell adhesion molecule 1 | Plasma Membrane | transmembrane receptor |
| MIMAT0003948 | miR-4712-5p (and other miRNAs w/seed CCAGUAC) | -15.630 | TargetScan Human | Moderate (predicted) | VHLL | VHL like | Other | other |
| MIMAT0003948 | miR-4712-5p (and other miRNAs w/seed CCAGUAC) | -15.630 | TargetScan Human | Moderate (predicted) | VTA1 | vesicle trafficking 1 | Cytoplasm | other |
| MIMAT0003948 | miR-4712-5p (and other miRNAs w/seed CCAGUAC) | -15.630 | TargetScan Human | Moderate (predicted) | WARS2 | yptophanyl tRNA synthetase 2, mitochondr | Cytoplasm | enzyme |
| MIMAT0003948 | miR-4712-5p (and other miRNAs w/seed CCAGUAC) | -15.630 | TargetScan Human | Moderate (predicted) | WDFY1 | WD repeat and FYVE domain containing 1 | Cytoplasm | other |
| MIMAT0003948 | miR-4712-5p (and other miRNAs w/seed CCAGUAC) | -15.630 | TargetScan Human | Moderate (predicted) | WDR55 | WD repeat domain 55 | Nucleus | other |
| MIMAT0003948 | miR-4712-5p (and other miRNAs w/seed CCAGUAC) | -15.630 | TargetScan Human | High (predicted) | XAF1 | XIAP associated factor 1 | Nucleus | other |
| MIMAT0003948 | miR-4712-5p (and other miRNAs w/seed CCAGUAC) | -15.630 | TargetScan Human | Moderate (predicted) | XK | X-linked Kx blood group | Plasma Membrane | transporter |
| MIMAT0003948 | miR-4712-5p (and other miRNAs w/seed CCAGUAC) | -15.630 | TargetScan Human | Moderate (predicted) | XXbac-BPG32J3.22 | | Other | other |
| MIMAT0003948 | miR-4712-5p (and other miRNAs w/seed CCAGUAC) | -15.630 | TargetScan Human | High (predicted) | YAE1 | YAE1 maturation factor of ABCE1 | Other | other |
| MIMAT0003948 | miR-4712-5p (and other miRNAs w/seed CCAGUAC) | -15.630 | TargetScan Human | Moderate (predicted) | YIF1B | acting factor homolog B, membrane traffick | Cytoplasm | other |
| MIMAT0003948 | miR-4712-5p (and other miRNAs w/seed CCAGUAC) | -15.630 | TargetScan Human | Moderate (predicted) | YWHAE | genase/tryptophan 5-monooxygenase activ | Cytoplasm | other |
| MIMAT0003948 | miR-4712-5p (and other miRNAs w/seed CCAGUAC) | -15.630 | TargetScan Human | Moderate (predicted) | ZBED2 | zinc finger BED-type containing 2 | Nucleus | transcription regulator |
| MIMAT0003948 | miR-4712-5p (and other miRNAs w/seed CCAGUAC) | -15.630 | TargetScan Human | Moderate (predicted) | ZC3H7A | zinc finger CCCH-type containing 7A | Extracellular Space | other |
| MIMAT0003948 | miR-4712-5p (and other miRNAs w/seed CCAGUAC) | -15.630 | TargetScan Human | Moderate (predicted) | ZCCHC2 | zinc finger CCHC-type containing 2 | Cytoplasm | other |
| MIMAT0003948 | miR-4712-5p (and other miRNAs w/seed CCAGUAC) | -15.630 | TargetScan Human | High (predicted) | ZDHHC11 | zinc finger DHHC-type containing 11 | Cytoplasm | enzyme |
| MIMAT0003948 | miR-4712-5p (and other miRNAs w/seed CCAGUAC) | -15.630 | TargetScan Human | High (predicted) | ZDHHC11B | zinc finger DHHC-type containing 11B | Other | other |
| MIMAT0003948 | miR-4712-5p (and other miRNAs w/seed CCAGUAC) | -15.630 | TargetScan Human | Moderate (predicted) | ZFAND3 | zinc finger AN1-type containing 3 | Other | other |
| MIMAT0003948 | miR-4712-5p (and other miRNAs w/seed CCAGUAC) | -15.630 | TargetScan Human | Moderate (predicted) | ZFAND5 | zinc finger AN1-type containing 5 | Nucleus | other |
| MIMAT0003948 | miR-4712-5p (and other miRNAs w/seed CCAGUAC) | -15.630 | TargetScan Human | Moderate (predicted) | ZFP69B | ZFP69 zinc finger protein B | Nucleus | transcription regulator |
| MIMAT0003948 | miR-4712-5p (and other miRNAs w/seed CCAGUAC) | -15.630 | TargetScan Human | Moderate (predicted) | ZFP90 | ZFP90 zinc finger protein | Nucleus | transcription regulator |
| MIMAT0003948 | miR-4712-5p (and other miRNAs w/seed CCAGUAC) | -15.630 | TargetScan Human | High (predicted) | ZG16B | zymogen granule protein 16B | Cytoplasm | other |
| MIMAT0003948 | miR-4712-5p (and other miRNAs w/seed CCAGUAC) | -15.630 | TargetScan Human | Moderate (predicted) | ZKSCAN3 | inc finger with KRAB and SCAN domains | Nucleus | transcription regulator |
| MIMAT0003948 | miR-4712-5p (and other miRNAs w/seed CCAGUAC) | -15.630 | TargetScan Human | Moderate (predicted) | ZMAT3 | zinc finger matrin-type 3 | Nucleus | other |
| MIMAT0003948 | miR-4712-5p (and other miRNAs w/seed CCAGUAC) | -15.630 | TargetScan Human | Moderate (predicted) | ZNF101 | zinc finger protein 101 | Nucleus | transcription regulator |
| MIMAT0003948 | miR-4712-5p (and other miRNAs w/seed CCAGUAC) | -15.630 | TargetScan Human | High (predicted) | ZNF107 | zinc finger protein 107 | Nucleus | transcription regulator |
| MIMAT0003948 | miR-4712-5p (and other miRNAs w/seed CCAGUAC) | -15.630 | TargetScan Human | High (predicted) | ZNF117 | zinc finger protein 117 | Nucleus | transcription regulator |
| MIMAT0003948 | miR-4712-5p (and other miRNAs w/seed CCAGUAC) | -15.630 | TargetScan Human | High (predicted) | ZNF12 | zinc finger protein 12 | Nucleus | transcription regulator |
| MIMAT0003948 | miR-4712-5p (and other miRNAs w/seed CCAGUAC) | -15.630 | TargetScan Human | Moderate (predicted) | ZNF14 | zinc finger protein 14 | Nucleus | transcription regulator |
| MIMAT0003948 | miR-4712-5p (and other miRNAs w/seed CCAGUAC) | -15.630 | TargetScan Human | Moderate (predicted) | ZNF154 | zinc finger protein 154 | Nucleus | transcription regulator |
| MIMAT0003948 | miR-4712-5p (and other miRNAs w/seed CCAGUAC) | -15.630 | TargetScan Human | High (predicted) | ZNF195 | zinc finger protein 195 | Nucleus | transcription regulator |
| MIMAT0003948 | miR-4712-5p (and other miRNAs w/seed CCAGUAC) | -15.630 | TargetScan Human | Moderate (predicted) | ZNF208 | zinc finger protein 208 | Nucleus | transcription regulator |
| MIMAT0003948 | miR-4712-5p (and other miRNAs w/seed CCAGUAC) | -15.630 | TargetScan Human | Moderate (predicted) | ZNF215 | zinc finger protein 215 | Nucleus | transcription regulator |
| MIMAT0003948 | miR-4712-5p (and other miRNAs w/seed CCAGUAC) | -15.630 | TargetScan Human | Moderate (predicted) | ZNF23 | zinc finger protein 23 | Nucleus | transcription regulator |
| MIMAT0003948 | miR-4712-5p (and other miRNAs w/seed CCAGUAC) | -15.630 | TargetScan Human | High (predicted) | ZNF233 | zinc finger protein 233 | Nucleus | transcription regulator |
| MIMAT0003948 | miR-4712-5p (and other miRNAs w/seed CCAGUAC) | -15.630 | TargetScan Human | Moderate (predicted) | ZNF256 | zinc finger protein 256 | Nucleus | transcription regulator |
| MIMAT0003948 | miR-4712-5p (and other miRNAs w/seed CCAGUAC) | -15.630 | TargetScan Human | Moderate (predicted) | ZNF260 | zinc finger protein 260 | Nucleus | transcription regulator |
| MIMAT0003948 | miR-4712-5p (and other miRNAs w/seed CCAGUAC) | -15.630 | TargetScan Human | High (predicted) | ZNF286A | zinc finger protein 286A | Nucleus | transcription regulator |
| MIMAT0003948 | miR-4712-5p (and other miRNAs w/seed CCAGUAC) | -15.630 | TargetScan Human | High (predicted) | ZNF30 | zinc finger protein 30 | Nucleus | transcription regulator |
| MIMAT0003948 | miR-4712-5p (and other miRNAs w/seed CCAGUAC) | -15.630 | TargetScan Human | High (predicted) | ZNF300 | zinc finger protein 300 | Nucleus | transcription regulator |
| MIMAT0003948 | miR-4712-5p (and other miRNAs w/seed CCAGUAC) | -15.630 | TargetScan Human | Moderate (predicted) | ZNF302 | zinc finger protein 302 | Nucleus | transcription regulator |
| MIMAT0003948 | miR-4712-5p (and other miRNAs w/seed CCAGUAC) | -15.630 | TargetScan Human | Moderate (predicted) | ZNF415 | zinc finger protein 415 | Nucleus | transcription regulator |
| MIMAT0003948 | miR-4712-5p (and other miRNAs w/seed CCAGUAC) | -15.630 | TargetScan Human | Moderate (predicted) | ZNF471 | zinc finger protein 471 | Nucleus | transcription regulator |
| MIMAT0003948 | miR-4712-5p (and other miRNAs w/seed CCAGUAC) | -15.630 | TargetScan Human | Moderate (predicted) | ZNF514 | zinc finger protein 514 | Nucleus | transcription regulator |
| MIMAT0003948 | miR-4712-5p (and other miRNAs w/seed CCAGUAC) | -15.630 | TargetScan Human | High (predicted) | ZNF525 | zinc finger protein 525 | Nucleus | transcription regulator |
| MIMAT0003948 | miR-4712-5p (and other miRNAs w/seed CCAGUAC) | -15.630 | TargetScan Human | High (predicted) | ZNF528 | zinc finger protein 528 | Other | other |
| MIMAT0003948 | miR-4712-5p (and other miRNAs w/seed CCAGUAC) | -15.630 | TargetScan Human | Moderate (predicted) | ZNF544 | zinc finger protein 544 | Nucleus | transcription regulator |
| MIMAT0003948 | miR-4712-5p (and other miRNAs w/seed CCAGUAC) | -15.630 | TargetScan Human | High (predicted) | ZNF589 | zinc finger protein 589 | Nucleus | transcription regulator |
| MIMAT0003948 | miR-4712-5p (and other miRNAs w/seed CCAGUAC) | -15.630 | TargetScan Human | Moderate (predicted) | ZNF606 | zinc finger protein 606 | Nucleus | transcription regulator |
| MIMAT0003948 | miR-4712-5p (and other miRNAs w/seed CCAGUAC) | -15.630 | TargetScan Human | Moderate (predicted) | ZNF611 | zinc finger protein 611 | Other | other |
| MIMAT0003948 | miR-4712-5p (and other miRNAs w/seed CCAGUAC) | -15.630 | TargetScan Human | High (predicted) | ZNF616 | zinc finger protein 616 | Nucleus | transcription regulator |
| MIMAT0003948 | miR-4712-5p (and other miRNAs w/seed CCAGUAC) | -15.630 | TargetScan Human | Moderate (predicted) | ZNF621 | zinc finger protein 621 | Nucleus | transcription regulator |
| MIMAT0003948 | miR-4712-5p (and other miRNAs w/seed CCAGUAC) | -15.630 | TargetScan Human | High (predicted) | ZNF676 | zinc finger protein 676 | Nucleus | transcription regulator |
| MIMAT0003948 | miR-4712-5p (and other miRNAs w/seed CCAGUAC) | -15.630 | TargetScan Human | Moderate (predicted) | ZNF701 | zinc finger protein 701 | Nucleus | transcription regulator |
| MIMAT0003948 | miR-4712-5p (and other miRNAs w/seed CCAGUAC) | -15.630 | TargetScan Human | Moderate (predicted) | ZNF705A | zinc finger protein 705A | Nucleus | transcription regulator |
| MIMAT0003948 | miR-4712-5p (and other miRNAs w/seed CCAGUAC) | -15.630 | TargetScan Human | High (predicted) | ZNF728 | zinc finger protein 728 | Other | other |
| MIMAT0003948 | miR-4712-5p (and other miRNAs w/seed CCAGUAC) | -15.630 | TargetScan Human | Moderate (predicted) | ZNF732 | zinc finger protein 732 | Other | other |
| MIMAT0003948 | miR-4712-5p (and other miRNAs w/seed CCAGUAC) | -15.630 | TargetScan Human | Moderate (predicted) | ZNF737 | zinc finger protein 737 | Other | other |
| MIMAT0003948 | miR-4712-5p (and other miRNAs w/seed CCAGUAC) | -15.630 | TargetScan Human | Moderate (predicted) | ZNF764 | zinc finger protein 764 | Other | other |
| MIMAT0003948 | miR-4712-5p (and other miRNAs w/seed CCAGUAC) | -15.630 | TargetScan Human | Moderate (predicted) | ZNF772 | zinc finger protein 772 | Other | other |
| MIMAT0003948 | miR-4712-5p (and other miRNAs w/seed CCAGUAC) | -15.630 | TargetScan Human | Moderate (predicted) | ZNF780B | zinc finger protein 780B | Other | other |
| MIMAT0003948 | miR-4712-5p (and other miRNAs w/seed CCAGUAC) | -15.630 | TargetScan Human | Moderate (predicted) | ZNF791 | zinc finger protein 791 | Other | other |
| MIMAT0003948 | miR-4712-5p (and other miRNAs w/seed CCAGUAC) | -15.630 | TargetScan Human | High (predicted) | ZNF813 | zinc finger protein 813 | Other | other |
| MIMAT0003948 | miR-4712-5p (and other miRNAs w/seed CCAGUAC) | -15.630 | TargetScan Human | Moderate (predicted) | ZNF829 | zinc finger protein 829 | Other | other |
| MIMAT0003948 | miR-4712-5p (and other miRNAs w/seed CCAGUAC) | -15.630 | TargetScan Human | High (predicted) | ZNF83 | zinc finger protein 83 | Nucleus | transcription regulator |
| MIMAT0003948 | miR-4712-5p (and other miRNAs w/seed CCAGUAC) | -15.630 | TargetScan Human | Moderate (predicted) | ZNF845 | zinc finger protein 845 | Other | other |
| MIMAT0003948 | miR-4712-5p (and other miRNAs w/seed CCAGUAC) | -15.630 | TargetScan Human | Moderate (predicted) | ZNF875 | zinc finger protein 875 | Nucleus | transcription regulator |
| MIMAT0003948 | miR-4712-5p (and other miRNAs w/seed CCAGUAC) | -15.630 | TargetScan Human | Moderate (predicted) | ZNF90 | zinc finger protein 90 | Nucleus | transcription regulator |
| MIMAT0003948 | miR-4712-5p (and other miRNAs w/seed CCAGUAC) | -15.630 | TargetScan Human | Moderate (predicted) | ZNF99 | zinc finger protein 99 | Other | other |
| MIMAT0003948 | miR-4712-5p (and other miRNAs w/seed CCAGUAC) | -15.630 | TargetScan Human | Moderate (predicted) | ZNRF2 | zinc and ring finger 2 | Cytoplasm | enzyme |

Supplementary Table 4. List of IDs and names of all molecules shown in the chord diagram.

| Gene Symbol or Function/Pathway name | Synonym(s) | Entrez Gene Name | Location | Family | Entrez Gene ID for Human | Entrez Gene ID for Mouse | Entrez Gene ID for Rat |
|---|---|---|---|---|---|---|---|
| Apelin Cardiac Fibroblast Signaling Pathway | | | Other | canonical pathway | | | |
| Apelin Endothelial Signaling Pathway | | | Other | canonical pathway | | | |
| Apelin Liver Signaling Pathway | | | Other | canonical pathway | | | |
| Apelin Muscle Signaling Pathway | | | Other | canonical pathway | | | |
| Apelin Pancreas Signaling Pathway | | | Other | canonical pathway | | | |
| APLNR | A, Agt, AGTRL1, Apelin Receptor, APJ, APJR, Apj receptor, HG11, msr/apj | apelin receptor | Plasma Membrane | G-protein coupled receptor | 187 | 23796 | 83518 |
| CAMK4 | A430110E23Rik, AI666733, Ca2+-CaM kinase IV, calcium/calmodulin-dependent protein kinase IV, CaM, caMK, Camk4,alpha, Camk4,α, CaMK-GR, CaMKI, CAM KINASE4, CaMK IV, CaMKIV/Gr, Ccdpk, D18Bwg0362e, RATCCDPK | calcium/calmodulin dependent protein kinase IV | Nucleus | kinase | 814 | 12326 | 25050 |
| COL1A1 | Alpha1-1 Collagen, alpha 1(I) COLLAGEN, alpha 1 (I) PROCOLLAGEN, Alpha1 type i collagen, Alpha1 Type I Procollagen, CAFYD, Co, Col, COL1, Col1a, Cola-1, COL I, COLIA1, COLLAGEN 1 alpha1, Collagen alpha1, COLLAGEN alpha1 (I), Collagen alpha 1(I) chain precursor, Collagen I, Collagen i-alpha, COLLAGEN I alpha1, Collagen i-α, Collagen Type1 Alpha1, Collagen type I, COLLAGEN type I alpha 1, collagen, type I, alpha 1, collagen type I alpha 1 chain, Collagen type I pro α 1, COLLAGEN type I α 1, collagen, type I, α 1, collagen type I α 1 chain, Collagen α1(I), Collagen α 1(I) chain precursor, EDSARTH1, EDSC, Mov-, Mov-13, OI1, OI2, OI3, OI4, PREPRO COLLAGEN alpha1(I), PROCOLLAGEN 1alpha1, PROCOLLAGEN 1(I), Procollagen alpha1 (1), PROCOLLAGEN alpha1(I), Procollagen alpha1 type I, PROCOLLAGEN-IALPHA1, Procollagen type 1, Procollagen α 1(i), type1 alpha 1 COLLAGEN, type1 alpha1 PROCOLLAGEN, type1 α 1 COLLAGEN, Type I (alpha 1) procollagen, Type I (α 1) procollagen, α 1(I) COLLAGEN, α 1 (I) PROCOLLAGEN | collagen type I alpha 1 chain | Extracellular Space | other | 1277 | 12842 | 29393 |
| COL1A2 | AA960264, AI325291, alpha2(I) COLLAGEN, alpha 2(I) COLLAGEN precursor, alpha2(I) PROCOLLAGEN, Co, Col, Col1, Col1 alpha 2, Col1 α 2, Cola-2, COLLAGEN 1 alpha2, COLLAGEN alpha 2(I), COLLAGEN alpha 2(I) CHAIN precursor, COLLAGEN alpha2 type1, COLLAGEN I, COLLAGEN I alpha2, COLLAGEN type1 alpha2, COLLAGEN type I alpha2, collagen, type I, alpha 2, collagen type I alpha 2 chain, COLLAGEN type I PRO alpha 2, COLLAGEN type I PRO α 2, collagen, type I, α 2, collagen type I α 2 chain, COLLAGEN α 2(I), COLLAGEN α 2(I) CHAIN precursor, EDSARTH2, EDSCV, LOC100911218, OI4, oim, Pro-alpha2(i)collagen, PROCOLLAGEN alpha2(I), Procollagen type1 alpha2 chain, Procollagen type 1, prepro-alpha2 chain, α 2(I) COLLAGEN precursor | collagen type I alpha 2 chain | Extracellular Space | other | 1278 | 12843 | 84352 |
| COL5A3 | Alpha4(v) collagen, collagen, type V, alpha 3, collagen type V alpha 3 chain, collagen, type V, α 3, collagen type V α 3 chain | collagen type V alpha 3 chain | Extracellular Space | other | 50509 | 53867 | 60379 |
| CREBBP | AW558298, CB, CBP, CBP/p300, CREB binding protein, KAT, KAT3A, MKHK1, p300/CBP, RSTS, RSTS1, RTS | CREB binding protein | Nucleus | transcription regulator | 1387 | 12914 | 54244 |
| CTGF | AMPHIROGULIN, Ccn, cellular communication network factor 2, CTGF, CTGF isoform 1, CTGRP, Fibroblast-inducible secreted, Fis, Fisp12, Hcs2, HCS24, IGFBP8, IGFBP-RP2, NOV2, Tissue growth factor | cellular communication network factor 2 | Extracellular Space | growth factor | 1490 | 14219 | 64032 |
| EDN1 | ARCND3, endothelin 1, ET-1, Eth-1, HDLCQ7, PPET1, PPET, preproET, QME | endothelin 1 | Extracellular Space | cytokine | 1906 | 13614 | 24323 |
| EDN3 | endothelin 3, ET-3, HSCR4, ls, PPET3, preproET-3, RGD1564825, tmgc48, WS4B | endothelin 3 | Extracellular Space | other | 1908 | 13616 | 366270 |
| FOS | AP-1, c-f, C-FOS, D12Rfj1, D12Rfj, FBJ osteosarcoma oncogene, Fos proto-oncogene, AP-1 transcription factor subunit, p55 | Fos proto-oncogene, AP-1 transcription factor subunit | Nucleus | transcription regulator | 2353 | 14281 | 314322 |
| Gna13 | AU024132, AU043124, G13, Galp, GALPHA13, G protein alpha 13, G protein subunit alpha 13, G protein subunit α 13, G protein α 13, guanine nucleotide binding protein, alpha 13, guanine nucleotide binding protein, α 13, G α 13 | G protein subunit alpha 13 | Plasma Membrane | enzyme | 10672 | 14674 | 303634 |
| GNAI2 | C76432, G, Gal, Galphai2, GI2, GI2 alpha, GI2 α, Gia, Gialpha-2, Gi, Alpha2 Subunit, GIP, Giα-2, Gna, GNAI2B, G protein subunit alpha i2, G protein subunit α i2, G protein α I2, guanine nucleotide binding, guanine nucleotide binding protein (G protein), alpha inhibiting 2, guanine nucleotide binding protein (G protein), α inhibiting 2, G α I2, H LUCA15.1, H LUCA16.1, I2, LOC102642216 | G protein subunit alpha i2 | Plasma Membrane | enzyme | 2771 | 14678 | 81664 |
| GNAO1 | a, alphaO, AW050213, DEE17, EIEE17, Ga, G-ALPHA-o, GAO2, GNAO, GNAO2, Go, Go2 α, Go alpha subunit 2, Go α, Go α subunit 2, G protein alpha O subunit, G protein ao, G protein O, G protein subunit alpha o1, G protein subunit α o1, G-protein α o, G protein α o1, G protein α O subunit, guanine nucleotide binding protein, alpha O, guanine nucleotide binding protein, α O, G zero, G-α-o, HLA-DOB1, NEDIM, RATBPGTPC | G protein subunit alpha o1 | Plasma Membrane | enzyme | 2775 | 14681 | 50664 |
| HIF1A | AA959795, bHLHe7, bHLHe78, HIF-1, HIF1-ALPHA, HIF-1alpha (hydroxylated), HIF-1-α, HIF-1α (hydroxylated), Hypoxia inducible factor 1 alpha subunit, hypoxia inducible factor 1, alpha subunit, hypoxia inducible factor 1 subunit alpha, hypoxia inducible factor 1 subunit α, Hypoxia inducible factor 1 α subunit, hypoxia inducible factor 1, α subunit, MO, MOP1, PASD8 | hypoxia inducible factor 1 subunit alpha | Nucleus | transcription regulator | 3091 | 15251 | 29560 |
| IRS1 | ENSMUSG00000022591, G972, G972R, HIRS-1, insulin receptor substrate 1, IR, IRS1IRM | insulin receptor substrate 1 | Cytoplasm | enzyme | 3667 | 16367 | 25467 |
| JUN | Activator protein 1, AP-1, API-1, c-Fos, c-ju, cJUN, Junc, jun proto-oncogene, Jun proto-oncogene, AP-1 transcription factor subunit, LOC100288387, LOC100291417, LOC100293034, p39, v-Jun, V-jun Avian Sarcoma Virus 17 Oncogene Homolog, V-jun Sarcoma Virus 17 Oncogene Homolog | Jun proto-oncogene, AP-1 transcription factor subunit | Nucleus | transcription regulator | 3725 | 16476 | 24516 |
| KRAS | AI929937, CFC2, c-Ki-ras, c-Ki-ras2, c-Ki-ras p21, C-K-RAS, K-, Ki, KI-RAS, KI-RAS4B, Kirsten rat sarcoma viral oncogene homolog, Kr, Kra, KRAS1, KRAS2, K-RAS2A, K-RAS2B, K-RAS B, KRAS proto-oncogene, GTPase, NS, NS3, OES, p21, p21B, p21ras, RALD, RAS, RASK2 | KRAS proto-oncogene, GTPase | Cytoplasm | enzyme | 3845 | 16653 | 24525 |
| MAP2K4 | JNKK, JNKK1, MAPK/ERK KINASE-1, MAPKK4, MEK4, mitogen-activated protein kinase kinase 4, MKK4, PRKMK4, SAPKK-1, Sek, SEK1, Ser, SERK1, SKK1 | mitogen-activated protein kinase kinase 4 | Cytoplasm | kinase | 6416 | 26398 | 287398 |
| MAPK10 | C230008H04RIK, JNK, JNK3, JNK3A, JNK3B1, JNK3B2, Jun kinase, mitogen-activated protein kinase 10, p493F1, p493F12, p54bSAPK, PRKM10, SAPb, SAPK1b, Sapk1-β, SAPK(beta), SAPKC, Sapk β, SAPK(β), Ser, Serk2 | mitogen-activated protein kinase 10 | Cytoplasm | kinase | 5602 | 26414 | 25272 |
| MAPK12 | AW123708, Erk, ERK3, ERK-6, LOC100509672, mitogen-activated protein kinase 12, P38ga, P38GAMMA, P38 MAPK gamma, P38 MAP KINASE gamma, P38 MAP KINASE γ, p38 MAPK γ, P38 γ, Prkm, PRKM12, Sa, SAPK-3 | mitogen-activated protein kinase 12 | Cytoplasm | kinase | 6300 | 29857 | 60352 |
| miR-199a-5p (and other miRNAs w/seed CCAGUGU) | hsa-miR-199a, hsa-mir-199a-1-5p, hsa-mir-199a-2-5p, hsa-miR-199a-5p, hsa-miR-199b, hsa-miR-199b-5p, hsa-miR-199-s, Mir199-5p, miR-199a-5p, miR-199a-5p(2), miR-199b, miR-199b*, miR-199b-5p, mmu-miR-199a, mmu-miR-199a-5p, mmu-miR-199b, mmu-miR-199b*, mmu-miR-199b-5p, rno-miR-199a, rno-miR-199a-5p | | Cytoplasm | mature microRNA | | | |
| miR-30c-5p (and other miRNAs w/seed GUAAACA) | hsa-miR-30a, hsa-miR-30a-5p, hsa-miR-30b, hsa-miR-30b-5p, hsa-miR-30c, hsa-miR-30c-5p, hsa-miR-30d, hsa-miR-30d-5p, hsa-miR-30e, hsa-miR-30e-5p, miR-200, mir-30, miR-30-5p, miR-30a, miR-30a-5p, miR-30b, MIR30B-5p, miR-30c, MIR30C-5p, miR-30d, miR-30e, MIR30E-5P, miR-384-5p, Mirn30a-5p, MIRN30E-5P, mmu-miR-30a, mmu-miR-30a-5p, mmu-miR-30b, mmu-miR-30b-5p, mmu-miR-30c, mmu-miR-30c-5p, mmu-miR-30d, mmu-miR-30d-5p, mmu-miR-30e, mmu-miR-30e-5p, mmu-miR-384-5p, rno-miR-30a, rno-miR-30a-5p, rno-miR-30b, rno-miR-30b-5p, rno-miR-30c, rno-miR-30c-5p, rno-miR-30d, rno-miR-30d-5p, rno-miR-30e, rno-miR-30e-5p, rno-miR-384-5p | | Cytoplasm | mature microRNA | | | |
| miR-383-5p (miRNAs w/seed GAUCAGA) | hsa-miR-383, hsa-miR-383-5p, miR-383, mmu-miR-383, mmu-miR-383-5p | | Cytoplasm | mature microRNA | | | |
| miR-4712-5p (and other miRNAs w/seed CCAGUAC) | hsa-miR-4712-5p, hsa-miR-770-5p, miR-770-5p | | Cytoplasm | mature microRNA | | | |
| miR-548c-3p (miRNAs w/seed AAAAAUC) | hsa-miR-548c, hsa-miR-548c-3p, miR-548c-3p | | Cytoplasm | mature microRNA | | | |
| miR-571 (miRNAs w/seed GAGUUGG) | hsa-miR-571, miR-571 | | Cytoplasm | mature microRNA | | | |
| NFKB1 | CVID12, EBP-1, KBF1, NF-k, NF-kappa-B, NF-kappa-B1, NFkappaB1 p105, Nf kappa b DNA binding subunit, NF-kappabeta, NFkappaB-p50, NF KAPPA B subunit P50, NF-kB, NFKB-p105, NFKB subunit P105/P50, NF-κ-B, NF-κ-B1, NFκB1 p105, Nf κ b DNA binding subunit, NFκB-p50, NF κ B subunit P50, NUCLEAR FACTOR KAPPA B DNA binding subunit, nuclear factor kappa B subunit 1, nuclear factor of kappa light polypeptide gene enhancer in B cells 1, p105, nuclear factor of κ light polypeptide gene enhancer in B cells 1, p105, NUCLEAR FACTOR κ B DNA binding subunit, nuclear factor κ B subunit 1, p105 Nfkb, p5, p50 NF-kappaB, P50 nf κ b, p50/p1, P50/P105 | nuclear factor kappa B subunit 1 | Nucleus | transcription regulator | 4790 | 18033 | 81736 |
| PIK3CD | 2410099E07Rik, 2610208K16RIK, APDS, AW545373, IMD14, p110D, p110de, P110DELTA, P110 δ, phosphatidylinositol-4,5-bisphosphate 3-kinase catalytic subunit delta, phosphatidylinositol-4,5-bisphosphate 3-kinase, catalytic subunit delta, phosphatidylinositol-4,5-bisphosphate 3-kinase catalytic subunit δ, phosphatidylinositol-4,5-bisphosphate 3-kinase, catalytic subunit δ, PI3K, PI3K-delta, PI3Kδ | phosphatidylinositol-4,5-bisphosphate 3-kinase catalytic subunit delta | Cytoplasm | kinase | 5293 | 18707 | 366508 |
| PIK3R6 | BB220380, C17orf38, HsT41028, p84, p84 PI3K regulatory subunit, p84 PIKAP, p87, p87PIKAP, p87(PIKAP), phosphoinositide-3-kinase regulatory subunit 5, phosphoinositide-3-kinase regulatory subunit 6, phosphoinositide-3-kinase, regulatory subunit 6, RGD1560850 | phosphoinositide-3-kinase regulatory subunit 6 | Cytoplasm | kinase | 146850 | 104709 | 497932 |
| PPARGC1A | A830037N07RIK, Gm11133, LEM6, LRPGC1, peroxisome proliferative activated receptor, gamma, coactivator 1 alpha, peroxisome proliferative activated receptor, γ, coactivator 1 α, Peroxisome Proliferator-Activating Receptor Gamma Coactivator 1 Alpha, Peroxisome Proliferator-Activating Receptor γ Coactivator 1alpha, Peroxisome Proliferator-Activating Receptor γ Coactivator 1 α, Pgc, PGC-1, PGC1A, PGC-1alpha, PGC-1(alpha), Pgc-1alpha-a, PGC-1v, PGC-1α, PGC-1(α), Pgco1, PGCvf, PGCvf-1, PPARGC1, PPARGC-1-alpha, PPARGC-1-α, PPARG coactivator 1 alpha, PPARG coactivator 1 α, ppar γ coactivator 1 | PPARG coactivator 1 alpha | Nucleus | transcription regulator | 10891 | 19017 | 83516 |
| PRKAA2 | 2310008I11Rik, A830082D05, AMPK, AMPK2, AMPKa2, AMPK alpha2, Ampk catalytic subunit alpha2, Hmg CoA Rk, PRKAA, protein kinase, AMP-activated, alpha 2 catalytic subunit, protein kinase AMP-activated catalytic subunit alpha 2, protein kinase AMP-activated catalytic subunit α 2, protein kinase, AMP-activated, α 2 catalytic subunit | protein kinase AMP-activated catalytic subunit alpha 2 | Cytoplasm | kinase | 5563 | 108079 | 78975 |
| PRKAB2 | 5730553K21Rik, AMPK beta2, AW049591, BB124140, protein kinase, AMP-activated, beta 2 non-catalytic subunit, protein kinase AMP-activated non-catalytic subunit beta 2, protein kinase AMP-activated non-catalytic subunit β 2, protein kinase, AMP-activated, β 2 non-catalytic subunit | protein kinase AMP-activated non-catalytic subunit beta 2 | Cytoplasm | kinase | 5565 | 108097 | 64562 |

Supplementary Table 4. List of IDs and names of all molecules shown in the chord diagram.

| | | | | | | | |
|---|---|---|---|---|---|---|---|
| PRKAG1 | AA571379, AMPKG, Ampk gamma, AMPK gamma1, Ampk γ, Ampk γ 1, BB036179, Pr, Prkaac, Prkga1, protein kinase, AMP-activated, gamma 1 non-catalytic subunit, protein kinase AMP-activated non-catalytic subunit gamma 1, protein kinase AMP-activated non-catalytic subunit γ 1, protein kinase, AMP-activated, γ 1 non-catalytic subunit | protein kinase AMP-activated non-catalytic subunit gamma 1 | Nucleus | kinase | 5571 | 19082 | 25520 |
| PRKAG2 | 2410051C13RIK, AAKG2, AAKG, AI854673, AMPK gamma2, CMH6, H91620p, protein kinase AMP-activated non-catalytic subunit gamma 2, protein kinase AMP-activated non-catalytic subunit γ 2, protein kinase, AMP-activated, gamma 2 non-catalytic subunit, protein kinase, AMP-activated, γ 2 non-catalytic subunit, WPWS | protein kinase AMP-activated non-catalytic subunit gamma 2 | Cytoplasm | kinase | 51422 | 108099 | 373545 |
| PRKCG | PKC, PKCC, PKCG, PKCga, PKC-gamma, PKCI, PKCI(3), PKC-γ, Prk, Prkc, Prkcc, protein kinase C gamma, protein kinase C, gamma, protein kinase C γ, protein kinase C, γ, RATPKCI, SCA14 | protein kinase C gamma | Cytoplasm | kinase | 5582 | 18752 | 24681 |
| PRKCI | 2310021H13Rik, AI427505, APKC, aPKC IOTA/LAMBDA, aPKCl, aPKC lambda, aPKC ι/λ, aPKC λ, DXS1179E, mKIAA4165, nPKC-iota, nPKC-ι, Pk, PKCI, PKC IOTA, Pkcl, PKCla, PKC LAMBDA, Pkc lamda, Pkc lamda/iota, Pkc lamda/ι, PKC-ι, PKC λ, Pr, PRKCL, protein kinase C iota, protein kinase C, iota, protein kinase C ι, protein kinase C, ι | protein kinase C iota | Cytoplasm | kinase | 5584 | 18759 | 84006 |
| RALB | 5730472O18Rik, dRalb, RAS like proto-oncogene B, v-ral simian leukaemia viral oncogene B, v-ral simian leukemia viral oncogene B | RAS like proto-oncogene B | Cytoplasm | enzyme | 5899 | 64143 | 116546 |
| RAP1A | AI848598, C21KG, G-22K, Krev, KREV-1, R, RAP1, RAP1A, member of RAS oncogene family, RAS-related protein 1a, SMGP21 | RAP1A, member of RAS oncogene family | Cytoplasm | enzyme | 5906 | 109905 | 295347 |
| RAP1B | 2810443E11Rik, K-REV, RAL1B, Rap1, RAP1B, member of RAS oncogene family, RAS related protein 1b | RAP1B, member of RAS oncogene family | Cytoplasm | enzyme | 5908 | 215449 | 171337 |
| RAP2A | 5830461H18RIK, K-REV, RAP2, RAP2A, member of RAS oncogene family, RAS related protein 2a, RbBP-30 | RAP2A, member of RAS oncogene family | Plasma Membrane | enzyme | 5911 | 76108 | |
| RAP2B | 4021402C18Rik, AA408554, RAP2B, member of RAS oncogene family | RAP2B, member of RAS oncogene family | Plasma Membrane | enzyme | 5912 | 74012 | 170923 |
| REL | c-R, C-Rel, HIVEN86A, REL proto-oncogene, NF-kB subunit, reticuloendotheliosis oncogene | REL proto-oncogene, NF-kB subunit | Nucleus | transcription regulator | 5966 | 19696 | 305584 |
| SP1 | 1110003E12RIK, AA450830, AI845540, Sp1-1, Sp1 transcription factor, Sp1 (trans spliced isoform), Trans-acting transcription factor 1 | Sp1 transcription factor | Nucleus | transcription regulator | 6667 | 20683 | 24790 |
| TGFB2 | BB105277, Cetermin, G-TSF, LDS4, Polyergin, Tgfb, Tgf-beta, TGFBETA2, Tgf-β, TGF-β2, transforming growth factor beta 2, transforming growth factor, beta 2, transforming growth factor β 2, transforming growth factor, β 2 | transforming growth factor beta 2 | Extracellular Space | growth factor | 7042 | 21808 | 81809 |
| VCAM1 | CD106, INCAM-100, vascular cell adhesion molecule 1, Vcam, VCAM1B, VECAM1 | vascular cell adhesion molecule 1 | Plasma Membrane | transmembrane receptor | 7412 | 22329 | 25361 |
